# Switching Residues: A Platform for the Synthesis of Fidaxomicin Antibiotics

**DOI:** 10.1002/anie.202419095

**Published:** 2024-12-04

**Authors:** Erik Jung, Tizian Griesser, Jordan Costafrolaz, Ondine Duverger, Yves Mattenberger, Silvia Dittmann, Andrea Dorst, Alexander Major, David Dailler, Daniel Schäfle, Susanne Sievers, Konstantin Brodolin, Patrick H. Viollier, Peter Sander, Karl Gademann

**Affiliations:** ^1^ Department of Chemistry University of Zurich 8057 Zürich Switzerland; ^2^ Institute of Medical Microbiology University of Zurich Zurich Switzerland; ^3^ Department of Microbiology and Molecular Medicine Faculty of Medicine University of Geneva Geneva Switzerland; ^4^ Institut de Recherche en Infectiologie de Montpellier Univ. Montpellier CNRS Montpellier 34293 France; ^5^ Department of Microbial Physiology and Molecular Biology Institute of Microbiology Center for Functional Genomics of Microbes University of Greifswald Greifswald Germany; ^6^ Institute of Medical Microbiology University of Zurich Zurich Switzerland National Reference Laboratory for Mycobacteria University of Zurich Zurich Switzerland

**Keywords:** Natural products, antibiotics, semisynthesis, site-selective catalysis, fidaxomicin

## Abstract

Peripheral modification is often the main approach to optimize natural products for improved biological activity or desired physicochemical properties. This procedure inevitably increases molecular weight, often accompanied by undesired increased lipophilicity. Removing structural elements from natural products is not always tolerated. This is also the case for the antibiotic fidaxomicin (Fdx), where every structural component has been shown to be crucial for antibiotic activity. In this work, we demonstrate how the residue switching approach can maintain biological activity of Fdx derivatives by replacing the rhamnoside‐dichlorohomoorsellinate moiety of Fdx with smaller, more polar building blocks. We used palladium‐catalysed allylic substitution to selectively install *N*‐nucleophiles on the core of Fdx. The new derivatives were designed to mimic the binding of Fdx to the bacterial RNA polymerase. Evaluation against *Mycobacterium tuberculosis*, *Clostridioides difficile*, and the Gram‐negative model organism *Caulobacter crescentus* demonstrated that the newly introduced residues can restore antibiotic activity, which was further supported by on‐target RNA polymerase assays. We combined the allylic substitution with an organocatalysed novioside acylation protocol to enable the functionalisation of two vectors on Fdx in one pot. This platform greatly expands the accessible chemical space for Fdx derivatives and enables the future development of systemic Fdx antibiotics.

## Graphical abstract

**Switching residues**: Adding new substituents to natural products increases their molecular weight and often their lipophilicity. We developed a Pd‐catalysed protocol to directly replace the rhamnoside‐arene fragment of the antibiotic fidaxomicin with simple amines in 1 step from the unprotected natural product. This enabled direct access to Fdx derivatives of reduced complexity with activity against RNA polymerase and various bacteria.

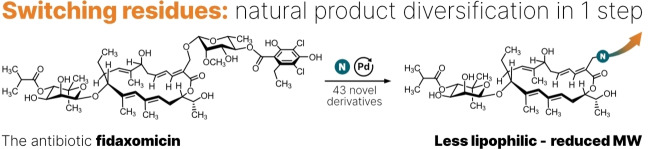

## Introduction

**Figure 1:**
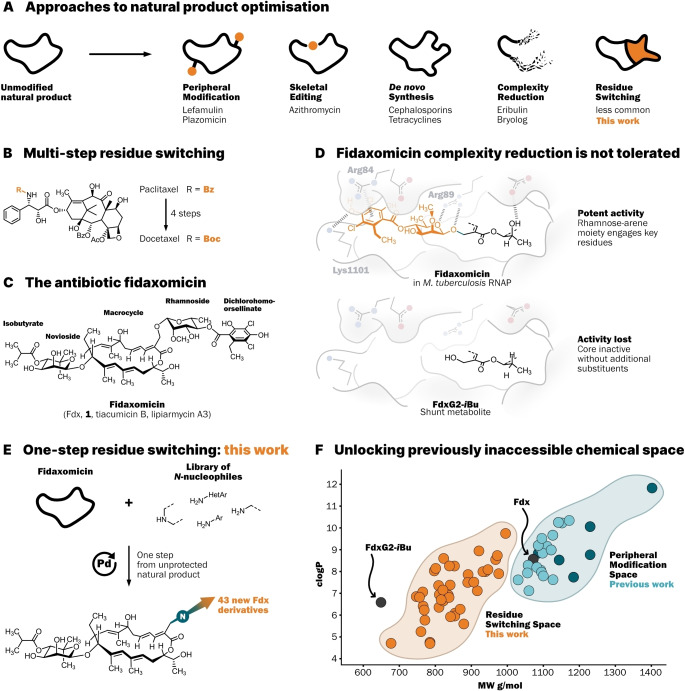
(**A**) Examples for different approaches to natural product optimisation (**B**) Switching residues on natural products usually requires multiple steps. (**C**) Structure of the glycosylated polyketide antibiotic fidaxomicin. (**D**) Complexity reduction is not a viable strategy for Fdx derivatisation due to loss of activity. Simplified representation of cryo‐EM structure of Fdx bound to RNAP (PDB: 6FBV).^[12]^ (**E**) A one‐step residue switching platform to access Fdx derivatives with reduced weight and lipophilicity. (**F**) Derivatives obtained through residue switching can reach previously inaccessible physicochemical space. Compounds synthesised in this work (orange), acyl derivatives^[13]^ (light blue), arene alkylation[^14^, ^15^] (dark blue). clogP was calculated using *OSIRIS DataWarrior 6.1.0*.^[16]^

For decades, natural products have served as a crucial source of lead structures for drug development. The natural product itself can turn out to be sufficiently potent, safe, and bioavailable to treat disease (e.g. vancomycin, paclitaxel, all‐*trans* retinoic acid, dextran, among many others).^[1]^ However, modification of the natural product structure is often necessary to improve physicochemical properties, spectrum of activity, or counter resistance (Figure 1A). Antibiotic discovery relies heavily on natural products and great efforts to modify their structure have led to dozens of approved drugs such as the cephalosporins or tetracyclines.[^1^, ^2^] The structural complexity of a natural product dictates which types of modifications are feasible for drug development. Often, the most accessible strategy is peripheral modification,^[3]^ wherein new moieties are added to existing reactive functional groups (recently approved examples include Lefamulin^[4]^ and Plazomicin^[5]^). In contrast, de novo synthesis is only possible after significant investment into the development of practical synthetic routes (e.g. Eravacycline).[^6^, ^7^] Complexity reduction of natural products can be a powerful approach if parts of the structure are known not to contribute to biological activity. The resulting structurally less complex targets are then much more synthetically accessible (e.g. Eribulin).[^8^, ^9^] If complexity reduction diminishes activity, then new substituents have to be introduced at the site of truncation to recover activity and modulate physicochemical properties. We term this less common approach residue switching. A notable example of residue switching is Docetaxel, which was prepared either from Paclitaxel in 4 steps or from 10‐deacetylbaccatin III in 5 steps, switching the naturally occurring Bz‐group to a Boc‐group (Figure 1B).[^10^, ^11^]

The natural product antibiotic fidaxomicin (Fdx, **1**, tiacumicin B, lipiarmycin A3) is approved for the treatment of gastrointestinal *Clostridioides difficile* infections (Figure 1C).[^17^, ^18^, ^19^] Fdx also shows potent in vitro activity against other Gram‐positive bacteria such as *Mycobacterium tuberculosis*.^[20]^ However, poor systemic absorption of Fdx precludes treatment of systemic infections by pathogens such as *M. tuberculosis*.^[21]^ Therefore, rationally designed derivatives with improved physicochemical properties that are informed by a detailed structural understanding are needed to unlock fidaxomicin's potential to treat tuberculosis (TB). The groups of Ebright and Campbell independently elucidated cryo‐EM structures of Fdx in complex with *M. tuberculosis* (*Mtb*) RNA polymerase (RNAP).[^12^, ^22^] These structures revealed that the dichlorohomoorsellinate engages in hydrogen bonding with β‐Lys1101 and a cation‐π interaction with β′‐Arg84. The rhamnoside is engaged in hydrogen bonding with β′‐Arg89. The latter interaction was shown to be essential in a transcription assay, since the IC_50_ of Fdx against the mutant RNAP^R89A^ increased>400‐fold. Removal of the dichlorohomoorsellinate moiety also reduced RNAP inhibition>400‐fold.^[12]^ Biosynthetic intermediates lacking the rhamnoside‐arene fragment, such as the shunt metabolite FdxG2‐*i*Bu, were also inactive against *Staphylococcus aureus*, underscoring the importance of this structural element (Figure 1D).^[23]^

A likely reason for the lack of new Fdx derivatives in the first four decades since its discovery in 1972 could lie in its structural complexity. Fdx contains two reactive phenolic hydroxy groups, five aliphatic hydroxy groups, two allylic β‐glucosides, and a labile isobutyrate moiety. Therefore, both total synthesis and selective semisynthetic modification of Fdx are extremely challenging.^[19]^ In 2015, the groups of Altmann, Gademann, and Zhu independently described total syntheses of the Fdx aglycon.[^24^, ^25^, ^26^] In the same year, the Gademann group achieved the first total synthesis of the fully glycosylated natural product.[^25^, ^27^] Since then, Roulland and co‐workers have also achieved the total synthesis of Fdx.^[28]^ The lessons learned in total synthesis have been paramount for the development of semisynthetic methods for the modification of Fdx.^[27]^

Previous efforts in our group have focused on site‐selective peripheral modification, which can retain antibiotic activity.[^13^, ^14^, ^15^] However, the addition of new substituents leads to an undesirable increase in molecular mass and little control over the overall physicochemical properties of the derivatives. Only major changes to the structure of Fdx could lead to the drastic physicochemical property shifts needed to achieve systemic availability. Extensive studies on the biosynthesis of Fdx, the biological activity of shunt metabolites, and synthetic studies revealed that every structural component of Fdx is essential for antimicrobial activity.[^13^, ^23^, ^29^, ^30^, ^31^] Consequently, reduction of complexity is not a feasible strategy for the development of new Fdx antibiotics (Figure 1D). *Only removal of structural components with concomitant introduction of new moieties (residue switching) could affect binding and physicochemical properties while also reducing or maintaining molecular mass*. Distinct from previous multi‐step residue switching approaches for other natural products, we developed a method to replace the rhamnoside‐dichlorohomoorsellinate residue of unprotected Fdx directly in one step through selective palladium catalysis (Figure 1E). By using various *N*‐nucleophiles, we synthesised a total of 43 new Fdx derivatives.

To illustrate how the residue switching approach unlocks previously inaccessible physicochemical space, we plotted calculated lipophilicity (clogP) against molecular weight (MW) of Fdx (grey), FdxG2‐*i*Bu (grey), previously synthesised Fdx derivatives via the peripheral modification approach (acyl derivatives^[13]^ in light blue, arene alkylation[^14^, ^15^] in dark blue), and the new compounds derived from the residue switching approach (orange) (Figure 1F). It is immediately apparent that peripheral modification generally leads to an increase in molecular weight, while also increasing lipophilicity. In contrast, residue switching delivers derivatives that are generally smaller and less lipophilic than parent Fdx, which is desirable for our future goal of developing systemically available Fdx antibiotics.^[32]^

## Results and Discussion

**Scheme 1:**
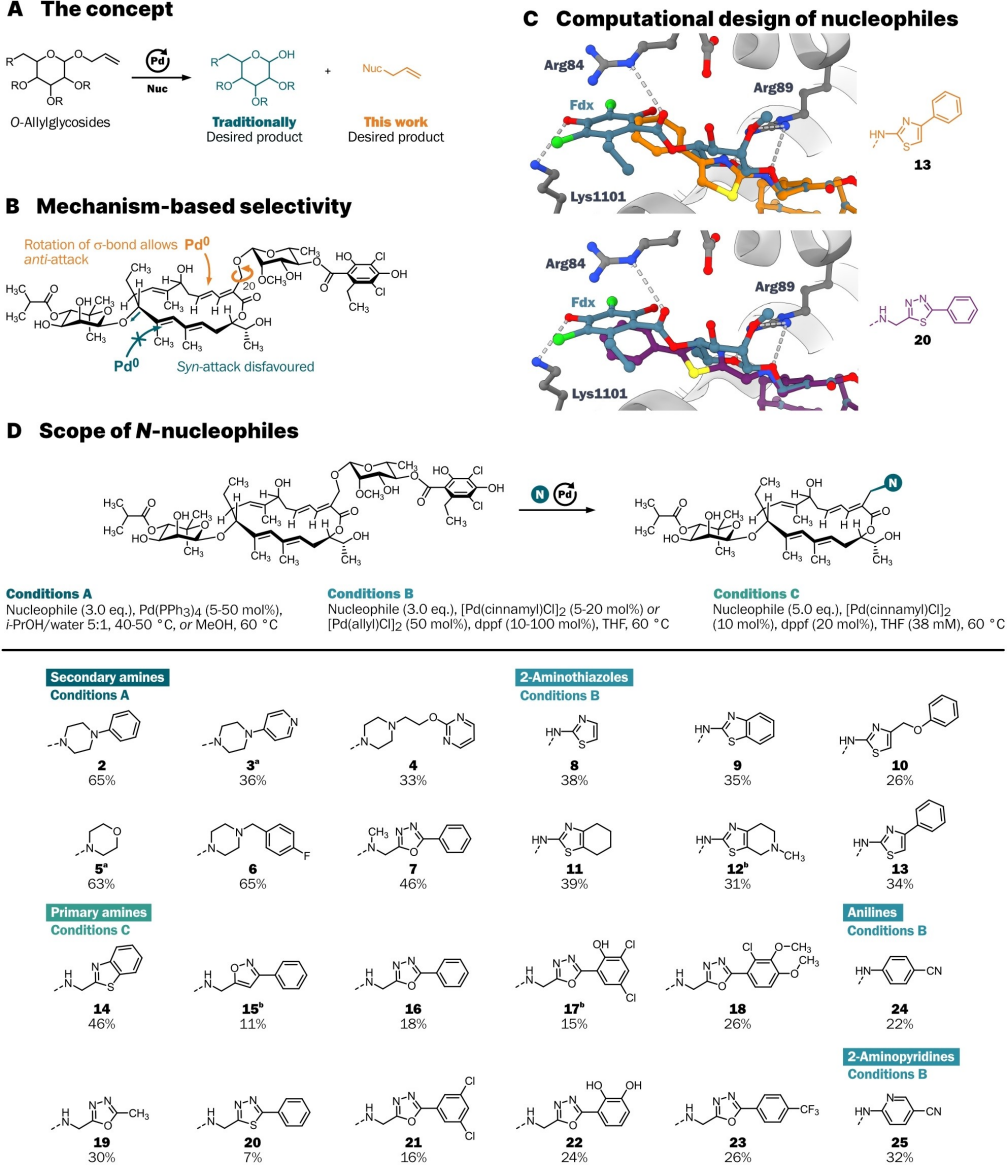
(**A**) Allylic glycosides can be functional handles for natural product derivatisation. (**B**) Mechanistic explanation for the excellent selectivity for substitution of the rhamnose. (**C**) Three‐dimensional representations of energy‐minimised structures of 2‐aminothiazole **13** (orange) and aminomethyl thiadiazole **20** (purple), each with Fdx (blue) in the cryo‐EM structure of *M. tuberculosis* RNAP by Ebright and co‐workers (PDB: 6FBV).^[12]^ (**D**) Scope of *N*‐nucleophiles for the Pd‐catalysed allylic substitution of the allylic rhamnoside of Fdx. [a] Mixture with 2’‐isobutyrate. [b] Obtained as the formate salt.

We set out to develop a synthetic platform that allows the facile introduction of *N*‐nucleophiles to displace the rhamnoside‐dichlorohomoorsellinate fragment of Fdx. *O*‐Allyl glycosides are usually seen as protected sugars,[^33^, ^34^, ^35^] yet those glycosides can also be seen as leaving groups and functional handles (Scheme 1A). The two allylic glycosides of Fdx could therefore serve as derivatisation points through formal nucleophilic displacement. Our group previously demonstrated the palladium‐mediated substitution of the rhamnoside‐dichlorohomoorsellinate fragment by activated *C*‐nucleophiles, with limited diversity of accessible derivatives (3 nucleophiles).^[13]^ The observed perfect selectivity for displacement of the rhamnoside over the novioside stems from the preference of Pd to operate via an *anti*‐*anti* mechanism.^[36]^ The backside of the π‐system adjacent to the noviose is shielded from *anti*‐attack of palladium by the rigid macrocycle. The rhamnoside is attached to the primary C20‐OH group, which allows σ‐bond rotation to position the glycosidic bond *anti* to the peripheral attack of palladium (see Scheme 1B, *Supplementary* Scheme 1). Exploiting this selectivity and expanding the method to *N*‐nucleophiles would allow the use of the vast range of easily accessible *N*‐nucleophile building blocks, rapidly accessing a library of new Fdx derivatives.

In developing our method, we considered that Fdx is prone to decomposition (loss of noviose) and rearrangements (ester migration, double bond isomerisation) upon exposure to heat or even mildly basic or acidic conditions (see *Supplementary* Scheme 2).^[27]^ Due to their close structural similarity, the resulting side products are extremely difficult to separate chromatographically. The displacement of challenging allylic leaving groups such as alcohols (including glycosides) by Pd can be promoted by polar solvents, which served as a starting point for our optimization.[^37^, ^38^, ^39^] After extensive screening, we found that the use of THF or alcohol/water mixtures, in combination with Pd(PPh_3_)_4_ or [Pd(cinnamyl)Cl]_2_/dppf at 40–60 °C efficiently displaces the rhamnoside‐dichlorohomoorsellinate residue of Fdx with the model nucleophiles *N*‐methylpiperazine and 2‐aminobenzothiazole (see *Supplementary* Scheme 3, *Supporting Information Table* 1). The use of benzylamine resulted in the unexpected formation of Bn‐N‐(Fdx)_2_ dimers, as substitution of the resulting secondary amine with another equivalent of Fdx is possible. Increasing the equivalents of benzylamine to 5.0 eq. and diluting the reaction to 38 mM significantly reduced the formation of this side product (see *Supporting Information Table* 2). The formation of the linear substitution product and the identity of common by‐products were assigned based on 2D NMR spectroscopy (see *Supplementary* Figures 1–3). Prior to purification, the reaction mixtures were treated with 3‐mercaptopropyl‐functionalised silica gel, scavenging palladium to prevent potential interference in the biological evaluation.[^40^, ^41^]

**Scheme 2:**
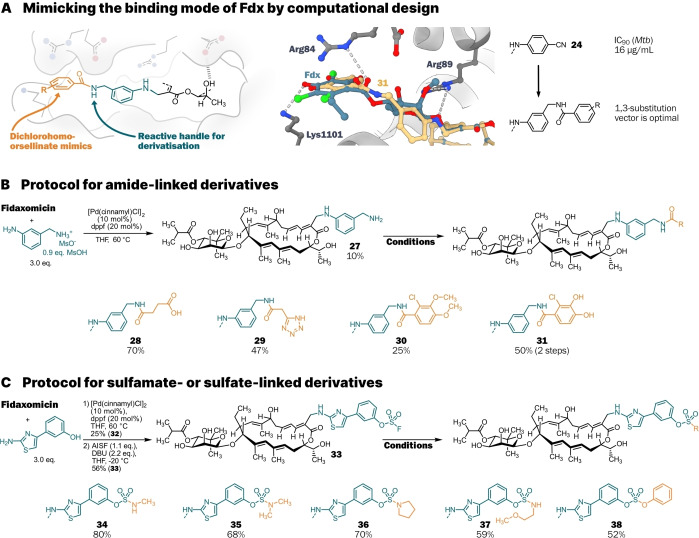
(**A**) Design of derivatives to mimic the arene of Fdx leads to structural evolution of aniline **24**. Three‐dimensional representation of energy‐minimised structure of aniline **31** (beige) with Fdx (blue) in the cryo‐EM structure of *Mtb* RNAP by Ebright and co‐workers (PDB: 6FBV).^[12]^ (**B**) Synthesis of amides **28**–**31** via late‐stage amidation of primary amine **27**. (**C**) Synthesis of sulfamate‐ and sulfate‐linked derivatives **34**–**38** via late‐stage SuFEx with fluorosulfate **33**. AISF=[4‐(acetylamino)phenyl]imidodisulfuryl difluoride.

**Scheme 3:**
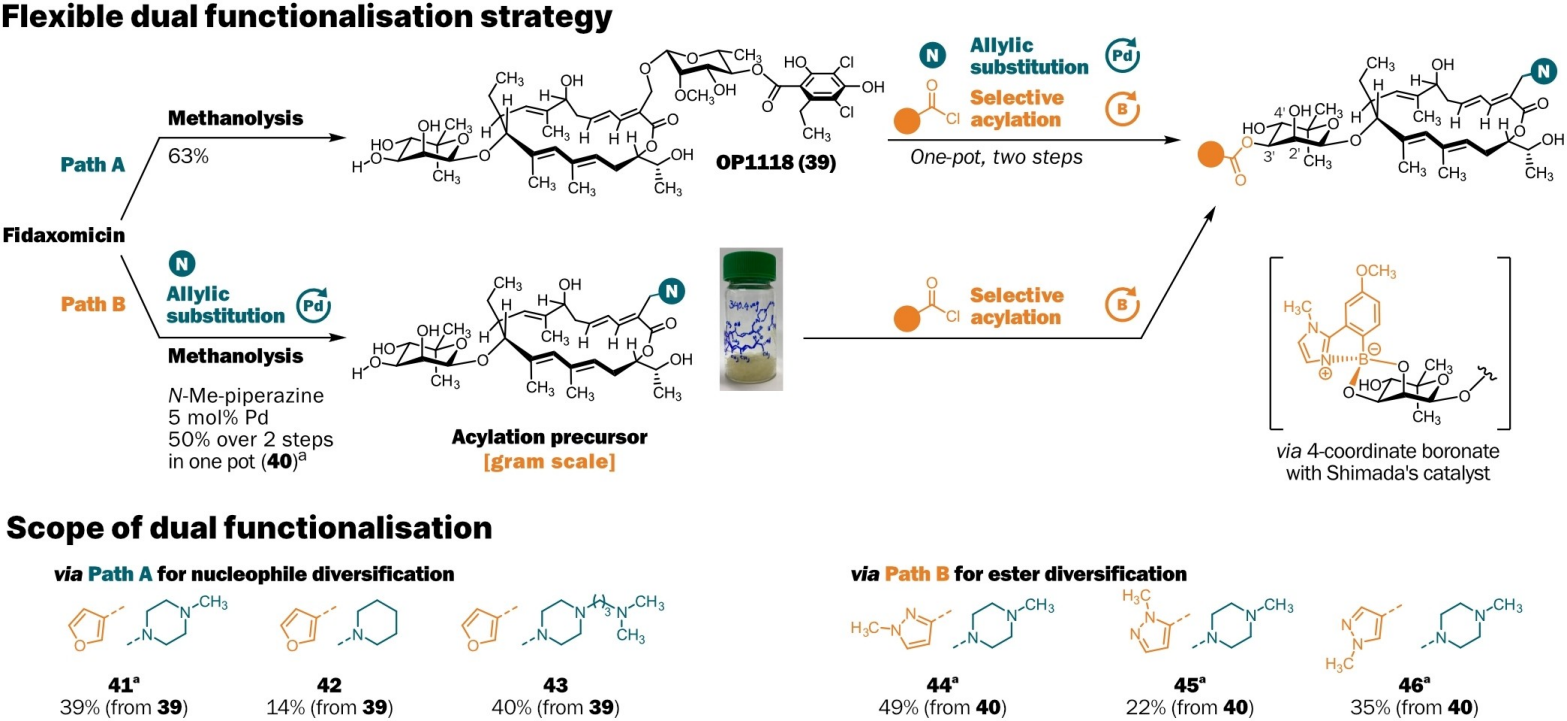
One‐pot dual functionalisation strategy. [a] Obtained as the formate salt.

**Figure 2:**
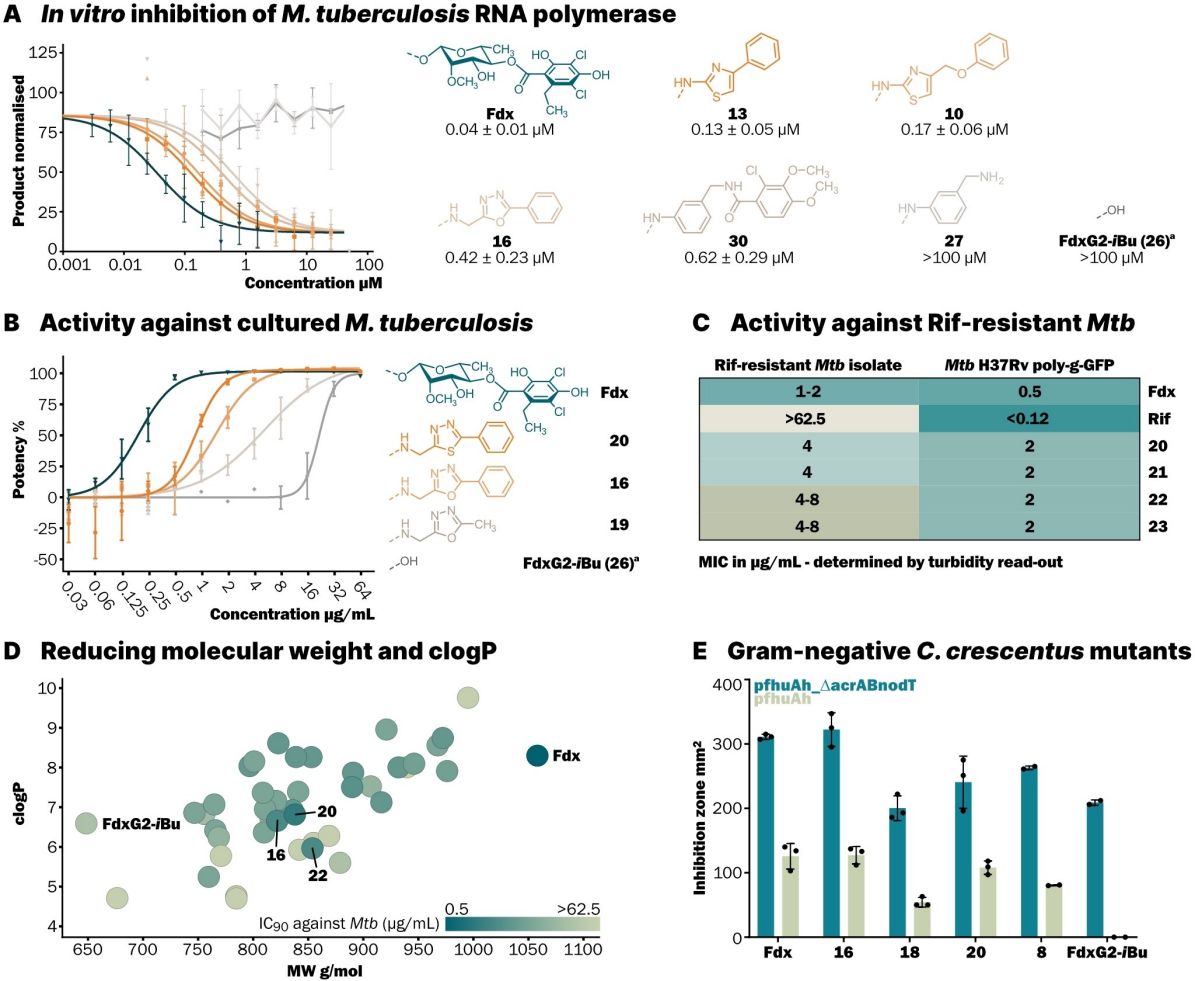
(**A**) IC_50_ determination by fluorescent run‐off transcription assay with *Mtb* RNAP. Inhibitor concentration plotted against normalised response and fitted using non‐linear regression with three parameters and shared top and bottom values for all data sets (*GraphPad Prism 10.1.2*). Performed in triplicate (*n*=3), centerline and error bars represent mean±standard deviation (s.d.). IC_50_ values given in μM±s.d. (**B**) Growth inhibition of *M. tuberculosis* H37Rv poly‐g‐GFP determined by fluorescent read‐out. Inhibitor concentration plotted against normalised response and fitted using non‐linear regression with variable slope and four parameters, lower bound was constrained to 0 (*GraphPad Prism 10.1.2*). Performed in triplicate (*n*=3), centerline and error bars represent mean±s.d. (**C**) Minimal inhibitory concentrations against a Rif‐resistant *Mtb* isolate and *M. tuberculosis* H37Rv poly‐g‐GFP determined by turbidity read‐out. (**D**) Physicochemical property plot (clogP vs. MW). clogP was calculated using *OSIRIS DataWarrior 6.1.0*.^[16]^ Colour coding corresponds to potency against *Mtb* (IC_90_) as determined in panel B. (**E**) Activity against *C. crescentus* pfhuA_ΔacrAB‐nodT and *C. crescentus* pfhuA determined by Kirby‐Bauer disk diffusion method. Inhibition zone was measured with *ImageJ 1.8.0*. Performed in triplicate for **Fdx**, **16**, **18**, and **20** (*n*=3) and in duplicate for **8** and FdxG2‐*i*Bu (*n*=2), centerline and error bars represent mean±s.d. [a] The shunt metabolite FdxG2‐*i*Bu also lacks the C18‐OH group of Fdx as it is installed later in the biosynthesis.

We selected further *N*‐nucleophiles based on their predicted ability to mimic crucial RNAP contacts of Fdx, such as that of the rhamnose to β′‐Arg89, based on a cryo‐EM structure of Fdx bound to RNAP.^[12]^ Using energy minimisation with the MAB force field in moloc,^[42]^ we designed different linker structures such as 4‐substituted 2‐aminothiazoles, aminomethyl‐substituted heterocycles, anilines, and 2‐aminopyridines (Scheme 1C, Scheme 1D). Analogues of derivatives, such as the aminomethyl oxadiazole series, were synthesised via multi‐step synthesis (see *Supplementary Schemes 4–8*). The allylic substitution on Fdx was also achieved on gram scale using 5 mol % Pd(PPh_3_)_4_, demonstrating the scalability of this method. Thus, a robust protocol for the introduction of *N*‐nucleophiles into Fdx was established and the synthesis of 24 Fdx derivatives was achieved (Scheme 1D).

The synthesis of building blocks and the separation of closely eluting side products were the main bottlenecks of our allylic displacement protocol. To circumvent this, we envisioned the installation of a reactive handle through allylic substitution to readily introduce new substituents at a late stage and simplify purification. Two approaches were established: Amide bond formation from a primary amine and sulfamate/sulfate formation from a fluorosulfate. For amide formation, an aniline was chosen as a linker since the aniline **24** showed activity against *M. tuberculosis*. Computational design suggested that an aminomethyl moiety *meta* to the aniline would position benzamides in the RNAP in the same location as the arene residue of Fdx (Scheme 2A). Several benzylamine protecting groups were evaluated for compatibility with Fdx and the reaction conditions.

2‐(Trimethylsilyl)ethyloxycarbonyl‐ (Teoc) and nitroveratryl‐oxycarbonyl‐ (Nvoc) protected amines were introduced to Fdx and successfully deprotected, however separation of the benzylamine **27** from deprotection reagents proved difficult. In the end, using the mesylate salt of 3‐(aminomethyl)aniline was sufficient to suppress primary amine reactivity and yielded **27** in one step. Succinamide **28** and the tetrazole **29** were chosen for their potential to form salt bridges with the basic residues at the entrance of the Fdx binding site.[^14^, ^43^] The catechol **31** mimics the dichlorohomoorsellinate moiety of Fdx and corresponds to the siderophore moiety of the 5^th^ generation cephalosporin antibiotic Cefiderocol.^[44]^ Succinamide **28** was obtained rapidly with succinic anhydride in 70 % yield. Amides **29**–**31** were obtained quickly after HATU‐mediated coupling with the respective carboxylic acids (Scheme 2B).

S^VI^‐F Electrophiles have been established as connecting hubs for the SuFEx (Sulfur Fluoride Exchange) click reaction framework.[^45^, ^46^] Through reaction with an SO_2_F‐transfer reagent, phenols can be turned into fluorosulfate electrophiles for late‐stage diversification. As free phenols are compatible with our allylic substitution protocol, we selected 3‐(2‐aminothiazol‐4‐yl)phenol as the nucleophile, resulting in phenol **32**. After screening SO_2_F transfer reagents, base, solvent, and temperature, the use of AISF in combination with DBU in THF at −20 °C gave fluorosulfate **33** in 56 % yield.^[47]^ Subsequently, we obtained the sulfamates **34**–**37** from fluorosulfate **33** and various amines through HOBt‐mediated SuFEx in 59–80 % yield.^[48]^ The sulfate **38** was synthesized in 52 % yield through reaction with phenol, HMDS, and BTMG (Scheme 2C).^[49]^ With these approaches we have demonstrated the utility and flexibility of reactive handles for rapid access to Fdx derivatives.

To gain better control over the physicochemical properties of the derivatives, it would be desirable to modify more than one site of Fdx at once. To achieve this, we combined our established selective acylation protocol^[13]^ with the newly developed allylic substitution. Using Shimada's boronic acid catalyst,^[50]^ it is possible to selectively introduce acyl groups to the 3’‐OH group of OP1118 (the deacylated metabolite of Fdx, **39**) from readily available acid chlorides. We found that allylic substitution in MeOH, followed by a solvent swap to 1,4‐dioxane, enabled the introduction of both a secondary amine at the C20‐position as well as an acyl group on the 3’‐OH of the novioside in one pot in 14–40 % yield over both steps for the furans **41**–**43** (Scheme 3, path A, *Supplementary* Scheme 9). Alternatively, allylic substitution of Fdx on gram scale in MeOH, followed by methanolysis of the isobutyrate in one‐pot through addition of potassium carbonate, gave the acylation precursor **40** in 50 % yield over two steps. Precursor **40** could then be acylated to yield the three pyrazoles **44**–**46** in 22‐49 % yield (Scheme 3, path B). If diversification of the nucleophile is desired, path A should be taken, and path B for diversification of the ester moiety. The combination of the palladium‐catalysed site‐selective substitution and the organocatalysed site‐selective acylation is a powerful method to gain control over two functionalisation vectors on Fdx at the same time. In total, we synthesised 43 new Fdx derivatives for evaluation of their biological activities.

With robust synthetic access to new Fdx derivatives, we investigated if these compounds of reduced complexity could still retain antibiotic activity. The shunt metabolite FdxG2‐*i*Bu (**26**), which can be obtained from a glycosyltransferase knock‐out producer strain, represents complexity reduction of Fdx without replacement substituents on the core.[^23^, ^51^] We first determined the ability of Fdx, its shunt metabolite FdxG2‐*i*Bu, and representative derivatives to inhibit *M. tuberculosis* (*Mtb*) RNAP using a fluorescent run‐off transcription assay (Figure 2A). Truncation of the rhamnoside‐dichlorohomoorsellinic fragment leads to complete loss of inhibitory activity against *M. tuberculosis* RNAP (Fdx IC_50_ 0.04±0.01 μM vs. FdxG2‐*i*Bu IC_50_>100 μM). Therefore, the main challenge we faced was regaining activity by mimicking how the rhamnoside‐arene fragment binds to RNAP through careful design of the nucleophiles. Because even if the potency of Fdx is not entirely restored, this may be outweighed by the potential improvements in physicochemical properties owed to a smaller molecular size of the derivatives. We were delighted to see that 2‐aminothiazoles **13** (IC_50_ 0.13±0.05 μM) and **10** (IC_50_ 0.17±0.06 μM) inhibit RNAP within an order of magnitude of Fdx, which is remarkable considering the 2‐aminothiazoles are drastically less complex than the rhamnoside‐dichlorohomoorsellinate moiety. Notable is also the increase in RNAP inhibition from amine **27** (IC_50_>100 μM) to benzamide **30** (IC_50_ 0.62±0.29 μM), which may be due to additional interactions created by the dichlorohomoorsellinate mimic.

Next, we investigated whether the observed on‐target activity also translated to cultured *M. tuberculosis*. Growth inhibition was determined by fluorescent read‐out of a

*M. tuberculosis* strain expressing GFP.^[52]^ Within the aminomethyl oxa‐/thiadiazole series, an increase in activity against *Mtb* was observed with increasing length of the introduced nucleophile (Figure 2B). Swapping phenyl for methyl substitution on the oxadiazole (**19**→**16**) improved activity against *Mtb* (IC_90_ 4 μg/mL (inhibitory concentration needed for 90 % inhibition of fluorescence as proxy for growth inhibition)). The thiadiazole **20**, which features increased substituent angles on the heterocycle, leads to further improved antibiotic activity (IC_90_ 2 μg/mL). For the most potent 2‐aminothiazole **10** an IC_90_ value of 8 μg/mL was observed. Fdx derivatives could substitute Rifampicin (Rif) in the treatment of infections caused by Rif‐resistant *M. tuberculosis* strains, since Fdx is also an RNAP inhibitor with no Rif binding site overlap. Encouragingly, the previously observed activities also translated to a Rif‐resistant *Mtb* isolate, as representative aminomethyl oxa‐/thiadiazole derivatives (**20**–**23**) showed MIC values of up to 4 μg/mL (compared to Fdx MIC 1‐2 μg/mL) (Figure 2C). Taken together, these derivatives restore activity lost by Fdx truncation to the same order of magnitude as Fdx (e.g. Fdx IC_90_ 0.5 μg/mL vs. **20** IC_90_ 2 μg/mL). Potent derivatives were obtained with a molecular weight reduction of more than 200 g/mol and lipophilicity reduction of more than two clogP units (Figure 2D). With further development, these derivatives could therefore fulfil physicochemical requirements that are not met by Fdx or other previously described derivatives. In addition, we evaluated the derivatives against *Clostridioides difficile*, revealing 2‐aminothiazole **10** (MIC 1 μg/mL) and 2‐aminopyridine **25** (MIC 1 μg/mL) as the most potent derivatives (see *Supporting Information Table* 3).

Since Fdx is not active against most Gram‐negative (GN) bacteria, one of our goals is expanding its spectrum of activity. For entry into GN bacteria, generally, smaller and more polar compounds are preferred.[^32^, ^53^] The presence of primary amines can also be beneficial to compound accumulation in GN bacteria.^[54]^ Previously, it was established that Fdx lacks GN activity due to poor cell permeation and partially due to efflux.[^14^, ^55^] This was elucidated using mutant strains of the GN model organism *Caulobacter crescentus* that harbours an aqueous (ungated) protein pore in the outer membrane (p*fhuA* ^*hyp*^) leading to a permeable envelope and/or deletion in the *acrAB‐nodT* genes (Δ*acrAB*‐*nodT*) which impairs antibiotic efflux.[^56^, ^57^, ^58^, ^59^] We evaluated our new derivatives against all three mutant *C. crescentus* strains as well as the wild type. Fdx displayed an inhibition zone of 311±4.0 mm^2^ against Δ*acrAB*‐*nodT*+p*fhuA* ^*hyp*^ cells. To our delight, the oxadiazole **16** (322±26 mm^2^) was as active as Fdx against Δ*acrAB*‐*nodT*+p*fhuA* ^*hyp*^ cells, suggesting a similar level of *C. crescentus* RNAP inhibition (Figure 2E, see *Supporting Information Table* 3 for full data). The derivatives obtainable via the residue switching platform approach the “600 Dalton” threshold that has been observed for compounds that are able to traverse the outer membrane of GN bacteria.[^32^, ^60^, ^61^] Evaluation against the Δ*acrAB*‐*nodT*, p*fhuA* ^*hyp*^, and WT strains of *C. crescentus* showed, that despite the reduced size and increased polarity of the derivatives, envelope permeation, and to a smaller extent efflux, is still limiting activity against GN bacteria. As some of our derivatives (**16**, **20**, and **18**, see Figure 2E and *Supporting Information Table* 3) are nearly or equally as potent as Fdx against permeable and efflux‐deficient strains of the GN *C. crescentus*, we hope that further optimisation to improve uptake will expand the spectrum of Fdx antibiotics to encompass GN bacteria.

## Conclusions

We developed a platform for the synthesis of new fidaxomicin derivatives. Using site‐selective palladium catalysis, it is now possible to replace the rhamnoside‐dichlorohomoorsellinate residue of Fdx by primary amines, secondary amines,

2‐aminothiazoles, anilines, and 2‐aminopyridines in one step from the unprotected natural product. This was demonstrated on gram scale, which underscores the suitability of this method to produce large amounts of the desired derivatives for biological characterisation. The introduction of reactive handles with the allylic substitution enabled rapid introduction of different carboxylic acids via amide bond formation and the introduction of different amines via SuFEx to the scaffold. Two vectors on Fdx were functionalized at once by combining the allylic substitution protocol with organocatalytic site‐selective acylation. In total, 43 new Fdx derivatives were synthesised and evaluated for their biological activities. Every structural element of Fdx has been shown to be essential for biological activity. Therefore, restoring antibiotic activity after it has been lost through truncation of the structure is a great challenge. We synthesized derivatives based on computational design to mimic Fdx binding to RNAP. Several of the new derivatives are potent inhibitors of *M. tuberculosis* RNAP and partially or fully restore activity against *M. tuberculosis*, Rif‐resistant *M. tuberculosis*, *C. difficile*, and mutant strains of *C. crescentus*. The new derivatives are less lipophilic and have lower molecular weight. As a result, they occupy previously inaccessible physicochemical space that is closer to the properties of approved antibiotics. The newly developed platform may therefore enable future development of the next generation of Fdx antibiotics.

## CRediT Statement

Erik Jung: Conceptualization, Methodology, Investigation, Formal analysis, Writing – Original Draft, Visualization, Project administration. Tizian Griesser: Methodology, Investigation, Formal analysis. Jordan Costafrolaz: Methodology, Investigation. Ondine Duverger: Investigation, Formal analysis. Yves Mattenberger: Methodology. Silvia Dittmann: Investigation, Formal analysis. Andrea Dorst: Investigation, Conceptualization. Alexander Major: Investigation. David Dailler: Investigation, Conceptualization. Daniel Schäfle: Methodology, Investigation, Formal analysis. Susanne Sievers: Formal analysis, Resources, Supervision, Project administration, Funding acquisition. Konstantin Brodolin: Formal analysis, Resources, Supervision, Project administration, Funding acquisition. Patrick H. Viollier: Resources, Supervision, Project administration, Funding acquisition. Peter Sander: Resources, Supervision, Project administration, Funding acquisition. Karl Gademann: Conceptualization, Formal analysis, Resources, Writing – Review & Editing, Supervision, Project administration, Funding acquisition.

## Supporting Information

Supporting Information contains supplementary Schemes, Figures, and Tables, experimental procedures, compound characterization, and NMR spectra. The authors have cited additional references in the *Supporting Information* (Ref. [62–74]).

## Conflict of Interests

The authors declare no competing financial interest.

1

## Supporting information

As a service to our authors and readers, this journal provides supporting information supplied by the authors. Such materials are peer reviewed and may be re‐organized for online delivery, but are not copy‐edited or typeset. Technical support issues arising from supporting information (other than missing files) should be addressed to the authors.

Supporting Information

## Data Availability

Additional data were deposited at zenodo (10.5281/zenodo.13884496).

## References

[1] D. J. Newman , G. M. Cragg , J. Nat. Prod. 2020, 83, 770–803. 32162523 10.1021/acs.jnatprod.9b01285

[2] E. Patridge , P. Gareiss , M. S. Kinch , D. Hoyer , Drug Discovery Today 2016, 21, 204–207. 25617672 10.1016/j.drudis.2015.01.009

[3] O. Robles , D. Romo , Nat. Prod. Rep. 2014, 31, 318–334. 24468713 10.1039/c3np70087a PMC4041598

[4] R. Novak , Ann. N. Y. Acad. Sci. 2011, 1241, 71–81. 22191527 10.1111/j.1749-6632.2011.06219.x

[5] B. Becker , M. A. Cooper , ACS Chem. Biol. 2013, 8, 105–115. 23110460 10.1021/cb3005116

[6] P. M. Wright , I. B. Seiple , A. G. Myers , Angew. Chem. Int. Ed. 2014, 53, 8840–8869. 10.1002/anie.201310843 PMC4536949 24990531

[7] F. Liu , A. G. Myers , Curr. Opin. Chem. Biol. 2016, 32, 48–57. 27043373 10.1016/j.cbpa.2016.03.011

[8] E. A. Crane , K. Gademann , Angew. Chem. Int. Ed. 2016, 55, 3882–3902. 10.1002/anie.201505863 PMC4797711 26833854

[9] M. J. Towle , K. A. Salvato , J. Budrow , B. F. Wels , G. Kuznetsov , K. K. Aalfs , S. Welsh , W. Zheng , B. M. Seletsky , M. H. Palme , G. J. Habgood , L. A. Singer , L. V. Di Pietro , Y. Wang , J. J. Chen , D. A. Quincy , A. Davis , K. Yoshimatsu , Y. Kishi , M. J. Yu , B. A. Littlefield , Cancer Res. 2001, 61, 1013–1021. 11221827

[10] B. Xue , J. Zhao , Y. Fan , S. Chen , W. Li , J. Chen , Z. Li , H. Wang , H. Kong , Chem. Biodiversity 2020, 17, e1900631. 10.1002/cbdv.201900631 31967396

[11] N. J. Sisti , C. A. Swindell , Method for Docetaxel Synthesis 1997, WO1997034866, A1.

[12] W. Lin , K. Das , D. Degen , A. Mazumder , D. Duchi , D. Wang , Y. W. Ebright , R. Y. Ebright , E. Sineva , M. Gigliotti , A. Srivastava , S. Mandal , Y. Jiang , Y. Liu , R. Yin , Z. Zhang , E. T. Eng , D. Thomas , S. Donadio , H. Zhang , C. Zhang , A. N. Kapanidis , R. H. Ebright , Mol. Cell 2018, 70, 60–71. 29606590 10.1016/j.molcel.2018.02.026 PMC6205224

[13] D. Dailler , A. Dorst , D. Schäfle , P. Sander , K. Gademann , Commun. Chem. 2021, 4, 59. 36697765 10.1038/s42004-021-00501-6 PMC9814943

[14] E. Jung , A. Kraimps , S. Dittmann , T. Griesser , J. Costafrolaz , Y. Mattenberger , S. Jurt , P. H. Viollier , P. Sander , S. Sievers , K. Gademann , ChemBioChem 2023, 24, e202300570. 37728121 10.1002/cbic.202300570

[15] A. Dorst , R. Berg , C. G. W. Gertzen , D. Schäfle , K. Zerbe , M. Gwerder , S. D. Schnell , P. Sander , H. Gohlke , K. Gademann , ACS Med. Chem. Lett. 2020, 11, 2414–2420. 33329763 10.1021/acsmedchemlett.0c00381 PMC7734799

[16] T. Sander , J. Freyss , M. von Korff , C. Rufener , J. Chem. Inf. Model. 2015, 55, 460–473. 25558886 10.1021/ci500588j

[17] European Medicines Agency, *Dificlir Assessment Report*, **2011**.

[18] W. Erb , J. Zhu , Nat. Prod. Rep. 2013, 30, 161–174. 23111588 10.1039/c2np20080e

[19] A. Dorst , K. Gademann , Helv. Chim. Acta 2020, 103, e2000038.

[20] E. J. C. Goldstein , F. Babakhani , D. M. Citron , Clin. Infect. Dis. 2012, 55, S143–S148. 22752863 10.1093/cid/cis339 PMC3388021

[21] Y. K. Shue , P. S. Sears , S. Shangle , R. B. Walsh , C. Lee , S. L. Gorbach , F. Okumu , R. A. Preston , Antimicrob. Agents Chemother. 2008, 52, 1391–1395. 18268081 10.1128/AAC.01045-07 PMC2292557

[22] H. Boyaci , J. Chen , M. Lilic , M. Palka , R. A. Mooney , R. Landick , S. A. Darst , E. A. Campbell , eLife 2018, 7, e34823. 29480804 10.7554/eLife.34823 PMC5837556

[23] Y. Xiao , S. Li , S. Niu , L. Ma , G. Zhang , H. Zhang , G. Zhang , J. Ju , C. Zhang , J. Am. Chem. Soc. 2011, 133, 1092–1105. 21186805 10.1021/ja109445q

[24] F. Glaus , K.-H. Altmann , Angew. Chem. Int. Ed. 2015, 54, 1937–1940. 10.1002/anie.201409510 25510439

[25] H. Miyatake-Ondozabal , E. Kaufmann , K. Gademann , Angew. Chem. Int. Ed. 2015, 54, 1933–1936. 10.1002/anie.201409464 25431322

[26] W. Erb , J.-M. Grassot , D. Linder , L. Neuville , J. Zhu , Angew. Chem. Int. Ed. 2015, 54, 1929–1932. 10.1002/anie.201409475 25422174

[27] H. Hattori , E. Kaufmann , H. Miyatake-Ondozabal , R. Berg , K. Gademann , J. Org. Chem. 2018, 83, 7180–7205. 29590752 10.1021/acs.joc.8b00101

[28] S. Norsikian , C. Tresse , M. François-Eude , L. Jeanne-Julien , G. Masson , V. Servajean , G. Genta-Jouve , J.-M. Beau , E. Roulland , Angew. Chem. Int. Ed. 2020, 59, 6612–6616. 10.1002/anie.202000231 32003915

[29] A. Dorst , E. Jung , K. Gademann , CHIMIA 2020, 74, 270–273. 32331545 10.2533/chimia.2020.270

[30] A. Dorst , I. S. Shchelik , D. Schäfle , P. Sander , K. Gademann , Helv. Chim. Acta 2020, 103, e2000130.

[31] I. Ferrara , G. A. Chesnokov , S. Dittmann , O. Blacque , S. Sievers , K. Gademann , JACS Au 2024, 4, 2267–2280. 38938792 10.1021/jacsau.4c00206 PMC11200244

[32] R. O'Shea , H. E. Moser , J. Med. Chem. 2008, 51, 2871–2878. 18260614 10.1021/jm700967e

[33] K. Nakayama , K. Uoto , K. Higashi , T. Soga , T. Kusama , Chem. Pharm. Bull. 1992, 40, 1718–1720. 10.1248/cpb.40.2909 1335843

[34] H. Tsukamoto , Y. Kondo , Synlett 2003, 1061–1063.

[35] M. Honda , H. Morita , I. Nagakura , J. Org. Chem. 1997, 62, 8932–8936.

[36] I. Stary , P. Kocovsky , J. Am. Chem. Soc. 1989, 111, 4981–4982.

[37] H. Tsukamoto , T. Suzuki , Y. Kondo , Synlett 2007, 2007, 3131–3136.

[38] X. Huo , M. Quan , G. Yang , X. Zhao , D. Liu , Y. Liu , W. Zhang , Org. Lett. 2014, 16, 1570–1573. 24621181 10.1021/ol5000988

[39] H. Kinoshita , H. Shinokubo , K. Oshima , Org. Lett. 2004, 6, 4085–4088. 15496105 10.1021/ol048207a

[40] M. Chatzopoulou , K. S. Madden , L. J. Bromhead , C. Greaves , T. J. Cogswell , S. Da Silva Pinto , S. R. G. Galan , I. Georgiou , M. S. Kennedy , A. Kennett , G. Apps , A. J. Russell , G. M. Wynne , ACS Med. Chem. Lett. 2022, 13, 262–270. 35173892 10.1021/acsmedchemlett.1c00638 PMC8842129

[41] T. Yamada , T. Matsuo , A. Ogawa , T. Ichikawa , Y. Kobayashi , H. Masuda , R. Miyamoto , H. Bai , K. Meguro , Y. Sawama , Y. Monguchi , H. Sajiki , Org. Process Res. Dev. 2019, 23, 462–469.

[42] P. R. Gerber , K. Müller , J. Comput.-Aided Mol. Des. 1995, 9, 251–268. 7561977 10.1007/BF00124456

[43] X. Cao , H. Boyaci , J. Chen , Y. Bao , R. Landick , E. A. Campbell , Nature 2022, 604, 541–545. 35388215 10.1038/s41586-022-04545-z PMC9635844

[44] T. Aoki , H. Yoshizawa , K. Yamawaki , K. Yokoo , J. Sato , S. Hisakawa , Y. Hasegawa , H. Kusano , M. Sano , H. Sugimoto , Y. Nishitani , T. Sato , M. Tsuji , R. Nakamura , T. Nishikawa , Y. Yamano , Eur. J. Med. Chem. 2018, 155, 847–868. 29960205 10.1016/j.ejmech.2018.06.014

[45] J. Dong , L. Krasnova , M. G. Finn , K. B. Sharpless , Angew. Chem. Int. Ed. 2014, 53, 9430–9448. 10.1002/anie.201309399 25112519

[46] T. Abdul Fattah , A. Saeed , F. Albericio , J. Fluorine Chem. 2018, 213, 87–112.

[47] H. Zhou , P. Mukherjee , R. Liu , E. Evrard , D. Wang , J. M. Humphrey , T. W. Butler , L. R. Hoth , J. B. Sperry , S. K. Sakata , C. J. Helal , C. W. Am Ende , Org. Lett. 2018, 20, 812–815. 29327935 10.1021/acs.orglett.7b03950

[48] M. Wei , D. Liang , X. Cao , W. Luo , G. Ma , Z. Liu , L. Li , Angew. Chem. Int. Ed. 2021, 60, 7397–7404. 10.1002/anie.202013976 33337566

[49] C. J. Smedley , J. A. Homer , T. L. Gialelis , A. S. Barrow , R. A. Koelln , J. E. Moses , Angew. Chem. Int. Ed. 2022, 61, e202112375. 10.1002/anie.202112375 PMC8867595 34755436

[50] N. Shimada , Y. Nakamura , T. Ochiai , K. Makino , Org. Lett. 2019, 21, 3789–3794. 31058511 10.1021/acs.orglett.9b01231

[51] E. Jung , M. Hunter , A. Dorst , A. Major , T. Teofilovic , R. Müller , K. Gademann , Helv. Chim. Acta 2024, e202400013.

[52] M. Dal Molin , P. Selchow , D. Schäfle , A. Tschumi , T. Ryckmans , S. Laage-Witt , P. Sander , J. Mol. Med. 2019, 97, 1601–1613. 31728550 10.1007/s00109-019-01840-7

[53] M. F. Richter , B. S. Drown , A. P. Riley , A. Garcia , T. Shirai , R. L. Svec , P. J. Hergenrother , Nature 2017, 545, 299–304. 28489819 10.1038/nature22308 PMC5737020

[54] K. A. Muñoz , P. J. Hergenrother , Acc. Chem. Res. 2021, 54, 1322–1333. 33635073 10.1021/acs.accounts.0c00895 PMC7969460

[55] A. Srivastava , M. Talaue , S. Liu , D. Degen , R. Y. Ebright , E. Sineva , A. Chakraborty , S. Y. Druzhinin , S. Chatterjee , J. Mukhopadhyay , Y. W. Ebright , A. Zozula , J. Shen , S. Sengupta , R. R. Niedfeldt , C. Xin , T. Kaneko , H. Irschik , R. Jansen , S. Donadio , N. Connell , R. H. Ebright , Curr. Opin. Microbiol. 2011, 14, 532–543. 21862392 10.1016/j.mib.2011.07.030 PMC3196380

[56] A. H. Delcour , Biochim. Biophys. Acta 2009, 1794, 808–816. 19100346 10.1016/j.bbapap.2008.11.005 PMC2696358

[57] V. Braun , J. Bacteriol. 2009, 191, 3431–3436. 19329642 10.1128/JB.00106-09 PMC2681897

[58] G. Krishnamoorthy , D. Wolloscheck , J. W. Weeks , C. Croft , V. V. Rybenkov , H. I. Zgurskaya , Antimicrob. Agents Chemother. 2016, 60, 7372–7381. 27697764 10.1128/AAC.01882-16 PMC5119019

[59] C. L. Kirkpatrick , P. H. Viollier , Chem. Biol. 2014, 21, 657–665. 24726830 10.1016/j.chembiol.2014.02.018

[60] D. G. Brown , T. L. May-Dracka , M. M. Gagnon , R. Tommasi , J. Med. Chem. 2014, 57, 10144–10161. 25402200 10.1021/jm501552x

[61] H. Nikaido , J. Biol. Chem. 1994, 269, 3905–3908. 7508433

[62] H. E. Gottlieb , V. Kotlyar , A. Nudelman , J. Org. Chem. 1997, 62, 7512–7515. 11671879 10.1021/jo971176v

[63] N. Srinivasan , A. Yurek-George , A. Ganesan , Mol. Diversity 2005, 9, 291–293. 10.1007/s11030-005-4386-8 16311804

[64] N. J. Liverton , R. A. Bednar , B. Bednar , J. W. Butcher , C. F. Claiborne , D. A. Claremon , M. Cunningham , A. G. DiLella , S. L. Gaul , B. E. Libby , E. A. Lyle , J. J. Lynch , J. A. McCauley , S. D. Mosser , K. T. Nguyen , G. L. Stump , H. Sun , H. Wang , J. Yergey , K. S. Koblan , J. Med. Chem. 2007, 50, 807–819. 17249648 10.1021/jm060983w

[65] M. Walter , Y. von Coburg , K. Isensee , K. Sander , X. Ligneau , J.-C. Camelin , J.-C. Schwartz , H. Stark , Bioorg. Med. Chem. Lett. 2010, 20, 5879–5882. 20728354 10.1016/j.bmcl.2010.07.098

[66] J. J. Crawford , D. F. Ortwine , B. Wei , W. B. Young , Heteroaryl Pyridone and Aza-Pyridone, Compounds as Inhibitors of Btk Activity 2013, WO2013067274A1.

[67] K.-C. Liu , J.-M. Fang , J.-T. Jan , T.-J. R. Cheng , S.-Y. Wang , S.-T. Yang , Y.-S. E. Cheng , C.-H. Wong , J. Med. Chem. 2012, 55, 8493–8501. 22963087 10.1021/jm3009844

[68] L. C. King , G. K. Ostrum , J. Org. Chem. 1964, 29, 3459–3461.

[69] M. Kücükdisli , H. Bel-Abed , D. Cirillo , W.-T. Lo , N.-L. Efrém , A. Horatscheck , L. Perepelittchenko , P. Prokofeva , T. A. L. Ehret , S. Radetzki , M. Neuenschwander , E. Specker , G. Médard , S. Müller , S. Wilhelm , B. Kuster , J. P. von Kries , V. Haucke , M. Nazaré , J. Med. Chem. 2023, 66, 14278–14302. 37819647 10.1021/acs.jmedchem.3c01319 PMC11151210

[70] Z. Morichaud , S. Trapani , R. K. Vishwakarma , L. Chaloin , C. Lionne , J. Lai-Kee-Him , P. Bron , K. Brodolin , Nat. Commun. 2023, 14, 484. 36717560 10.1038/s41467-023-36113-y PMC9886945

[71] Y. Hu , Z. Morichaud , S. Chen , J.-P. Leonetti , K. Brodolin , Nucleic Acids Res. 2012, 40, 6547–6557. 22570422 10.1093/nar/gks346 PMC3413145

[72] J. Bhat , R. Rane , S. M. Solapure , D. Sarkar , U. Sharma , M. N. Harish , S. Lamb , D. Plant , P. Alcock , S. Peters , S. Barde , R. K. Roy , J. Biomol. Screening 2006, 11, 968–976. 10.1177/1087057106291978 17021309

[73] C. Raynaud , K. G. Papavinasasundaram , R. A. Speight , B. Springer , P. Sander , E. C. Böttger , M. J. Colston , P. Draper , Mol. Microbiol. 2002, 46, 191–201. 12366842 10.1046/j.1365-2958.2002.03152.x

[74] U. Matt , P. Selchow , M. Dal Molin , S. Strommer , O. Sharif , K. Schilcher , F. Andreoni , A. Stenzinger , A. S. Zinkernagel , M. Zeitlinger , P. Sander , J. Nemeth , Int. J. Antimicrob. Agents 2017, 50, 55–62. 28506804 10.1016/j.ijantimicag.2017.02.022

