# Supplementary material for: Switching Residues: A Platform for the Synthesis of Fidaxomicin Antibiotics
Source: Angew Chem Int Ed Engl. 2024 Dec 4;64(7):e202419095. doi: 10.1002/anie.202419095 (PMC11811690; doi:10.1002/anie.202419095)

Angewandte Chemie
Eine Zeitschrift der Gesellschaft Deutscher Chemiker

Supporting Information

Switching Residues: A Platform for the Synthesis of Fidaxomicin Antibiotics

*E. Jung, T. Griesser, J. Costafrolaz, O. Duverger, Y. Mattenberger, S. Dittmann, A. Dorst, A. Major, D. Dailler, D. Schäfle, S. Sievers, K. Brodolin, P. H. Viollier, P. Sander, K. Gademann**

Table of Contents

1. Supplementary Figures, Schemes, and Tables 2
2. Synthesis 13

1.1 General Methods and Materials 13
1.2 General Procedures 15
1.3 Synthesis of *N*-Nucleophiles and Fidaxomicin Derivatives 17
- 1.3.1 General Considerations 17
- 1.3.2 Secondary Amines 20
- 1.3.3 Aminothiazoles 33
- 1.3.4 Primary Amines 47
- 1.3.5 Anilines and Aminopyridines 78
- 1.3.6 Amide-linked Derivatives 82
- 1.3.7 Sulfamate- and Sulfate-based derivatives 93
- 1.3.8 Derivatives Containing Noviose Modifications 109

1.4 Isolation of Shunt Metabolite 127
1.5 Biological Experiments 127
- 1.5.1 General procedure for determination of RNAP inhibition 127
- 1.5.2 General procedure for the determination of MIC values of *M. tuberculosis* 128
- 1.5.3 General procedure for the determination of MIC values of a Rifampicin-resistant *M. tuberculosis* isolate 129
- 1.5.4 General procedure for the determination of MIC values of *C. difficile* 130
- 1.5.5 Inhibition zone determination assay with *C. crescentus* 131

1.6 Molecular Modelling 131
1.7 References 132
1.8 NMR Spectra 134

1. Supplementary Figures, Schemes, and Tables

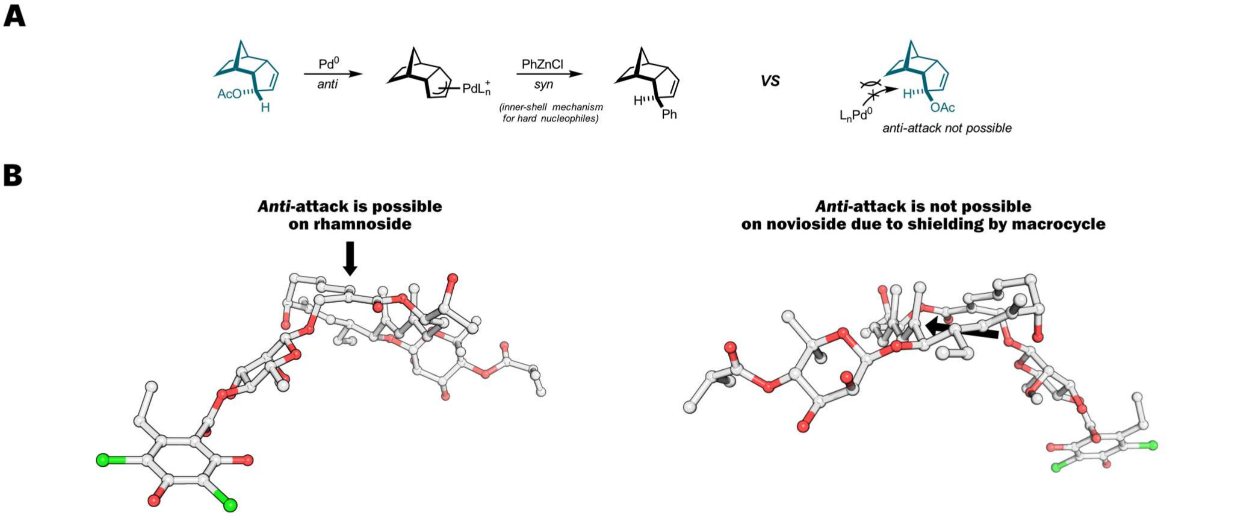

Supplementary Scheme 1. (A) Literature precedent for the preference of palladium to undergo *anti* addition to allyl acetates and steric shielding of the backface.[1] (B) The rhamnoside is located on a primary alcohol; this allows C-O σ-bond rotation for *anti*-attack of palladium. The backface of the π-system in conjugation with the novioside is shielded by the macrocycle.

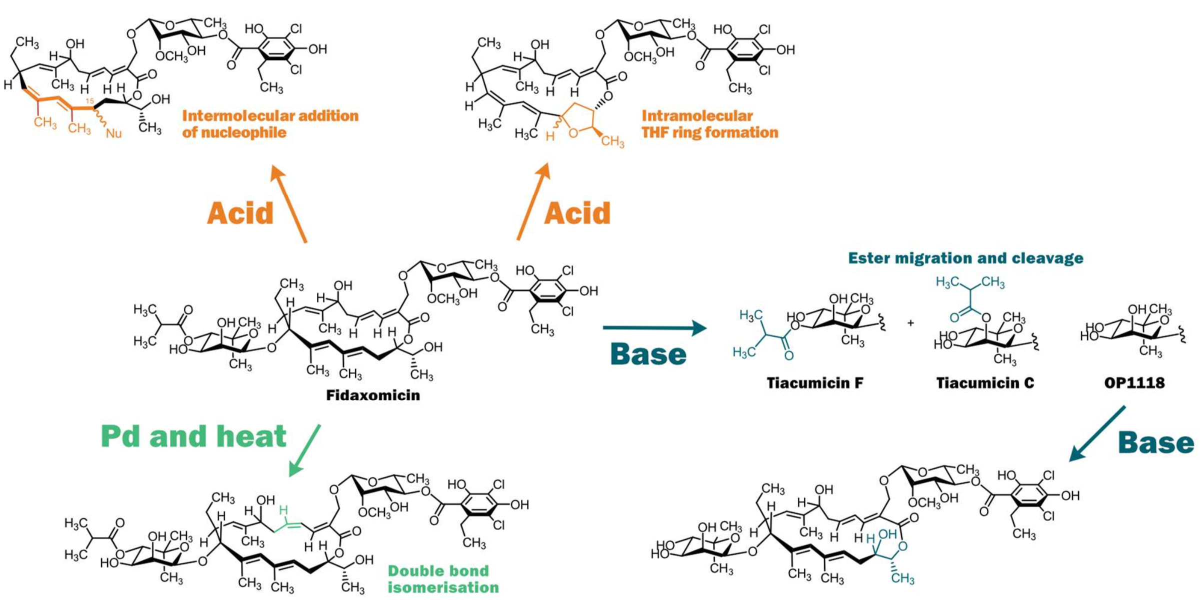

Supplementary Scheme 2. Known pathways of Fdx degradation.[2] Even mildly acidic or basic conditions can lead to rearrangements.

A

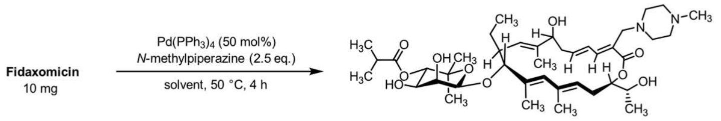

Entry	Solvent	Comment[a]
1	THF	10% conversion
2	*i*-PrOH	36% conversion
3	DMF	traces
4	DCE	traces
5	MeCN	traces
6	DMI	traces
7	HFIP	complex mixture
8	2,2,2-trifluoroethanol	complex mixture

[a]Conversion determined by UHPLC (detection at 270 nm).

B

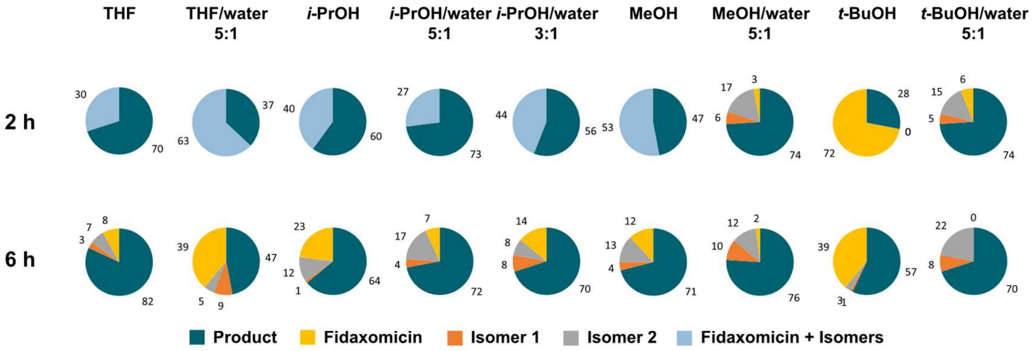

C

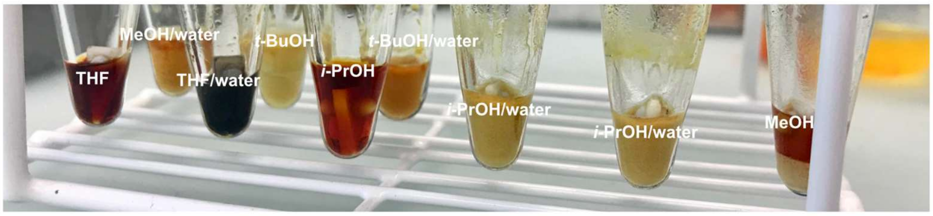

Supplementary Scheme 3. (A) Initial solvent screen for the allylic substitution on Fdx with secondary amines. (B) Detailed evaluation of THF and alcohols with and without water. THF without the addition of water is an excellent solvent for the reaction. All alcohols evaluated are productive solvents and benefit greatly from the addition of water in a ratio of 5:1. (C) Reactions in THF are generally homogenous. Alcohol/water mixtures are heterogeneous at high catalyst loadings.

Supplementary Table 1. Identification of optimal catalyst and ligand for 2-aminothiazoles.

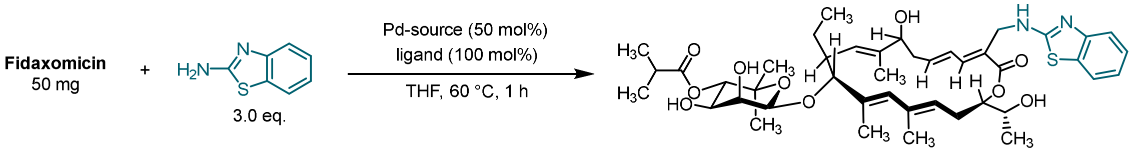

Entry	Pd-source	Ligand	Comment
1	[Pd(allyl)Cl]$_2$	dppf	58% conversion
**2**	**[Pd(cinnamyl)Cl]$_2$**	**dppf**	**67% conversion**
3	[Pd(allyl)Cl]$_2$	dppf-CF$_3$	44% conversion
4	[Pd(allyl)Cl]$_2$	dippf	3% conversion

[a]Conversion determined by UHPLC (detection at 270 nm).

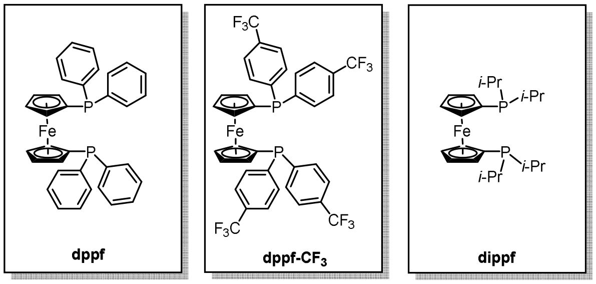

Supplementary Table 2. Optimization of reaction conditions to suppress dimer formation with primary amines. Both excess of nucleophile and high dilution suppress dimer formation. A concentration of 38 mM and 5.0 eq. of nucleophile were chosen as the optimal conditions.

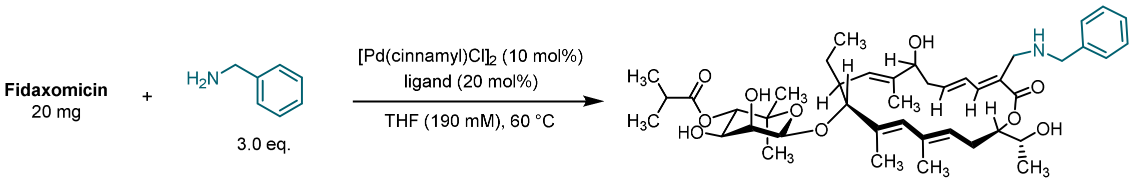

Entry	Deviation from above	15 min Conversion, P:D	90 min Conversion, P:D
1	none	43%, 5.0:1	91%, 2.2:1
2	1 mol% cat., 2 mol% dppf	4%	11%
3	1.2 eq. $BnNH_2$	28%, 1.6:1	52%, 1.1:1
**4**	**10 eq. $BnNH_2$**	**71%, 12.4:1**	**>99%, 13.7:1**
**5**	**38 mM**	**18%**	**57%, 4.4:1**

[a]Conversion determined by UHPLC (detection at 270 nm).

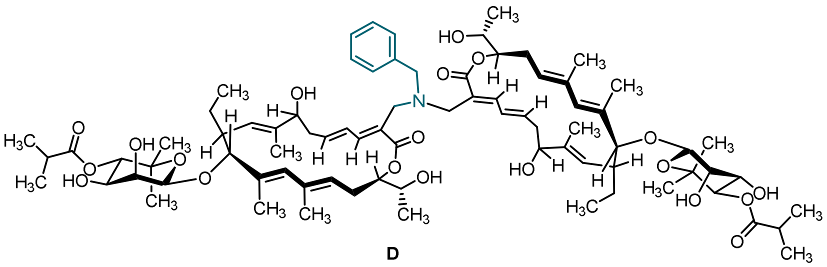

D

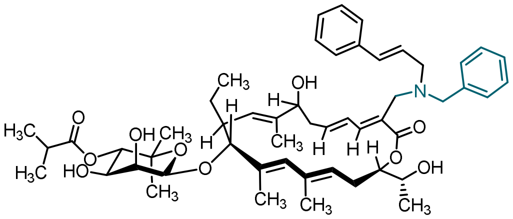

also observed

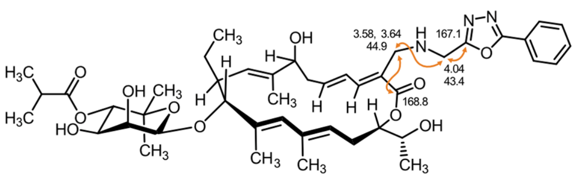

Supplementary Figure 1. 2D-NMR spectroscopy confirms that the linear product is obtained in the allylic substation with Fdx.

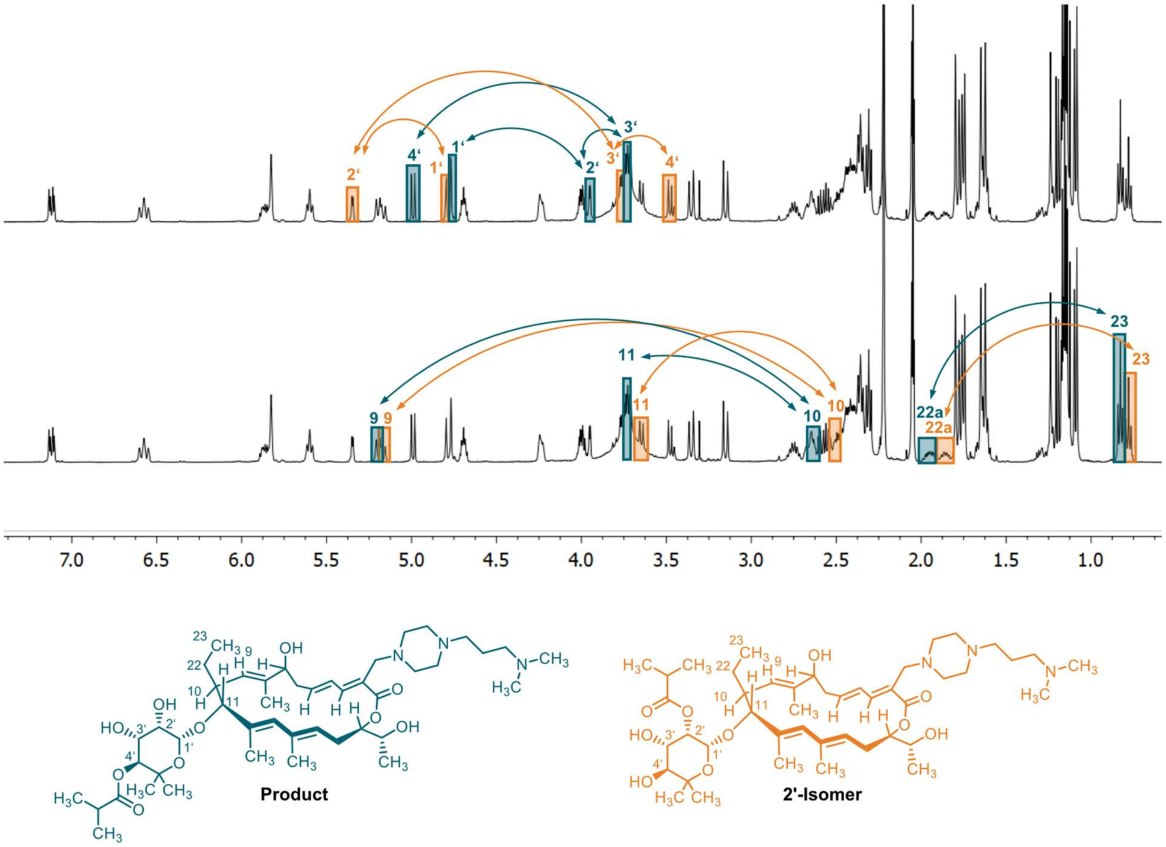

Supplementary Figure 2. Depending on nucleophile, temperature, and solvent, the isobutyrate moiety can migrate on the novioside. Shown is the ^{1}H spectrum of a mixture of desired 4'-product and the 2'-isomer. The arrows indicate observed COSY correlations.

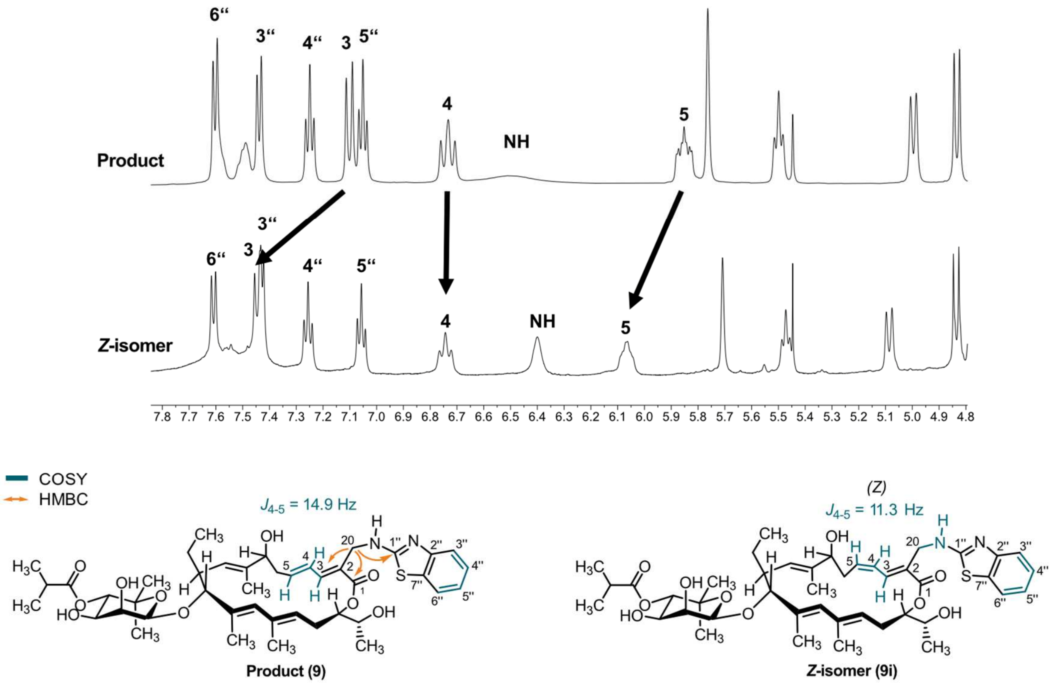

Supplementary Figure 3. At high temperatures and catalyst loadings isomerization of the C4-C5 double bond from *E* to *Z* is observed.

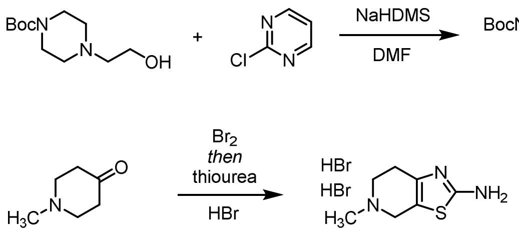

Supplementary Scheme 4. Synthesis of amine **4a** and 2-aminothiazole **12a**.

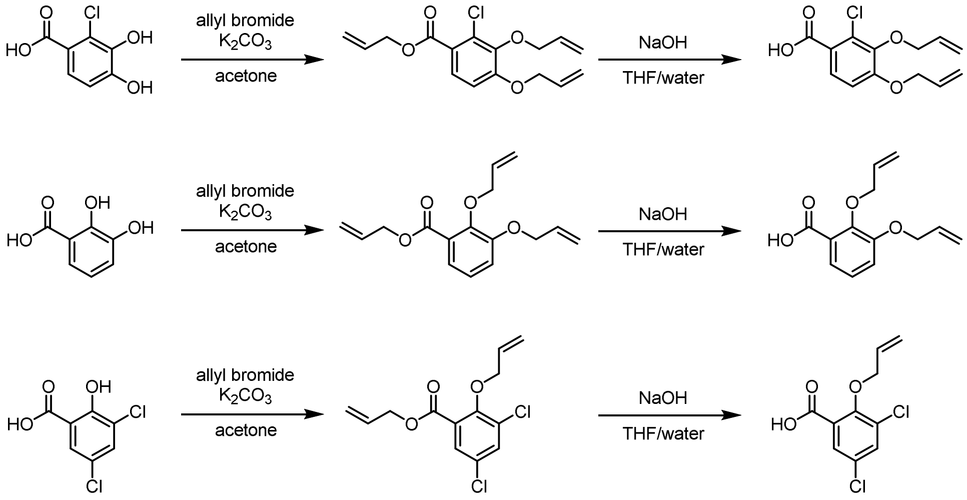

Supplementary Scheme 5. Synthesis of allyl protected phenol-containing benzoic acids.

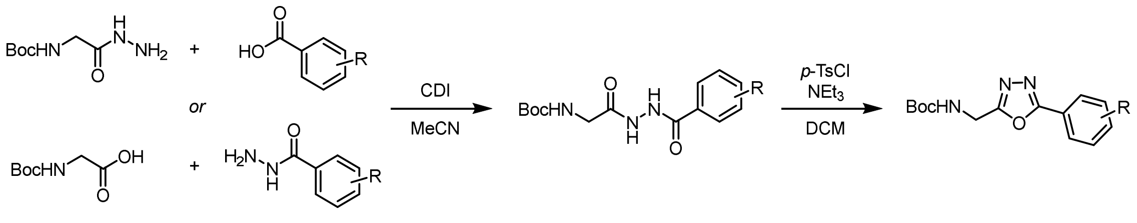

Supplementary Scheme 6. General synthetic approach to oxadiazoles from hydrazides and carboxylic acids.

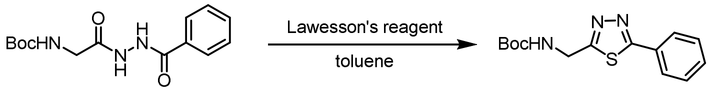

Supplementary Scheme 7. Synthesis of thiadiazoles.

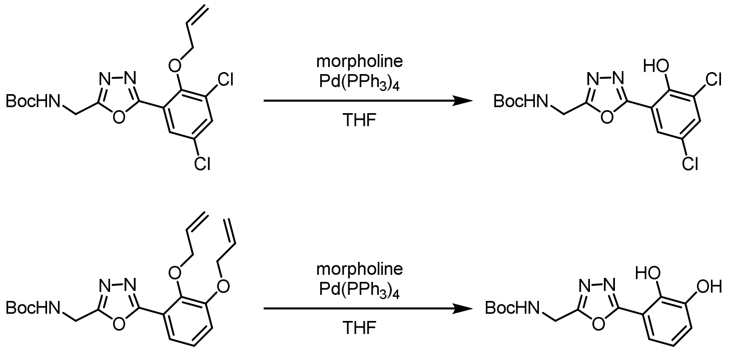

Supplementary Scheme 8. Deprotection of oxadiazoles containing allyl protecting groups.

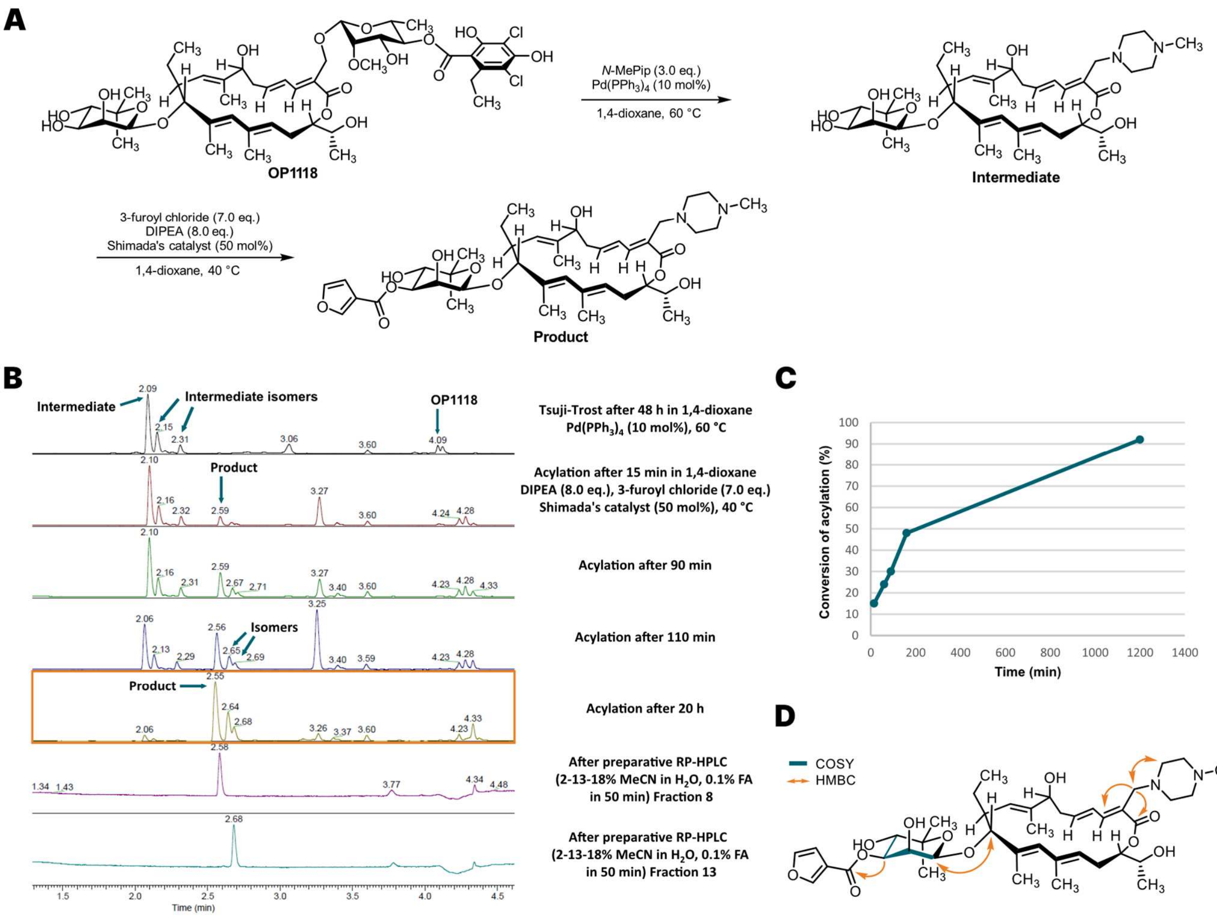

Supplementary Scheme 9. (A) One-pot allylic substitution and selective acylation of OP1118. High excess of base and acid chloride is required due to excess nucleophile and reactive dichlorohomoorsellinate by-product. In case of precious acid chlorides, this can be circumvented by isolation of the intermediate. (B) Reaction progress and purification of the one-pot dual functionalisation of OP1118. (C) Reaction progress of the acylation. Conversion determined by UHPLC (detection at 270 nm). (D) 2D-NMR experiments confirm the formation of the linear substitution product and selective 3’ -acylation.

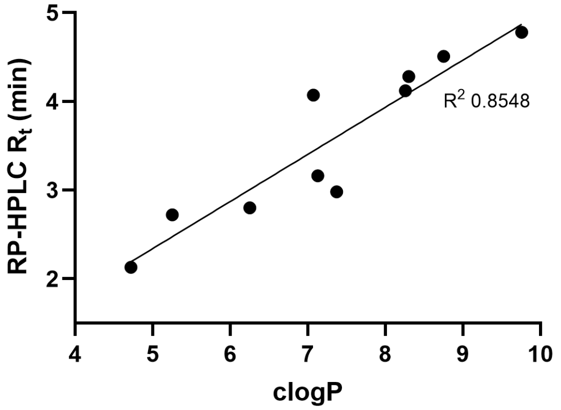

Supplementary Figure 4. Correlation of calculated lipophilicity (clogP, OSIRIS DataWarrior 6.1.0.) against RP-HPLC retention time (R_t in min, *Kinetex*® EVO C18; 1.7 µm; 100 Å, 50 x 2.1 mm; *Phenomenex, H2O/MeCN with 0.1% formic acid, 0-0.4 min 5% MeCN – 0.4-5.5 min 5-95% MeCN*). Derivatives containing basic functionality have lower retention times than predicted due to protonation by formic acid in mobile phase.

Supplementary Table 3. Biological evaluation of Fdx derivatives. *fhuAh* was expressed from plasmid. n.d. not determined, n.a. no activity observed at the tested concentration.

#	Compound	IC_{90} (µg/mL) *Mtb* H37Rv-poly-g-GFP Fluorescence read-out	MIC (µg/mL) *Mtb* H37Rv-poly-g-GFP Turbidity read-out	MIC (µg/mL) Rif-resistant *Mtb* isolate Turbidity read-out	IC_{50} (µM) *Mtb* RNAP Fluorescence read-out	MIC (µg/mL) *C. difficile*	Inhibition zone (mm^2) *C. crescentus* pfuhAh_ ΔacrABnodT	Inhibition zone (mm^2) *C. crescentus* pfuhAh
**Fdx**	**Fidaxomicin**	**0.5**	**0.5**	**1-2**	**0.04 ± 0.01**	**<0.0078**	**311 ± 4**	**126 ± 20**
**26**	**FdxG2-iBu**	**64**	**n.d.**	**n.d.**	**>100**	**4**	**209 ± 4[2]**	**n.a.[2]**
**Rif**	**Rifampicin**	n.d.	**<0.12**	**>62.5**	n.d.	n.d.	n.d.	n.d.
**2**	ERJ-138	16	n.d.	n.d.	n.d.	>32[1]	n.d.	n.d.
**3**	ERJ-139	16	n.d.	n.d.	n.d.	n.d.	n.d.	n.d.
**4**	ERJ-147	>64	n.d.	n.d.	n.d.	n.d.	n.d.	n.d.
**6**	ERJ-140	16	n.d.	n.d.	n.d.	16	n.d.	n.d.
**7**	ERJ-302	8	8	8-16	n.d.	16	n.d.	n.d.
**8**	ERJ-309	16	n.d.	n.d.	n.d.	4-8	263 ± 3[2]	81 ± 1[2]
**9**	ABt	8	4-8	8-16	n.d.	2	n.d.	n.d.
**10**	**ERJ-246**	**8**	**4-8**	**8-16**	**0.17 ± 0.06**	**1**	**n.d.**	**n.d.**
**11**	ERJ-322	16	n.d.	n.d.	n.d.	8-16	n.d.	n.d.
**12**	ERJ-194	32	n.d.	n.d.	n.d.	n.d.	n.d.	n.d.
**13**	**ERJ-318**	**8**	**8-16**	**8-16**	**0.13 ± 0.05**	**4**	**n.d.**	**n.d.**
**14**	ERJ-449	16	n.d.	n.d.	n.d.	>32	n.d.	n.d.
**15**	ERJ-541	16	n.d.	n.d.	n.d.	n.d.	n.d.	n.d.
**16**	**ERJ-301**	**4**	**4**	**4-8**	**0.42 ± 0.23**	**4-8**	**322 ± 26**	**127 ± 13**
**17**	ERJ-511	32	n.d.	n.d.	n.d.	2	n.d.	n.d.
**18**	ERJ-467	8	4	4-8	n.d.	8	200 ± 19	54 ± 8
**19**	ERJ-450	16	n.d.	n.d.	n.d.	32[1]	n.d.	n.d.
**20**	**ERJ-634**	**2**	**2**	**4**	**n.d.**	**n.d.**	**241 ± 40**	**108 ± 11**
**21**	ERJ-635	8	2	4	n.d.	4	73 ± 11	n.a.
**22**	ERJ-468	4	2	4-8	n.d.	8	n.d.	n.d.

**23**	ERJ-477	8	2	4-8	n.d.	4	n.d.	n.d.
**24**	ERJ-615	16	16	16-32	n.d.	4	n.d.	n.d.
**25**	ERJ-254	16	8	16-32	n.d.	1	n.d.	n.d.
**27**	ERJ-458	32	n.d.	n.d.	>100	>32[1]	119 ± 15	n.a.
**28**	ERJ-462	>64	n.d.	n.d.	n.d.	32	n.d.	n.d.
**29**	ERJ-465	64	n.d.	n.d.	n.d.	>32[1]	n.d.	n.d.
**30**	ERJ-404	16	n.d.	n.d.	0.62 ± 0.29	>32[1]	n.d.	n.d.
**31**	ERJ-420	>64	n.d.	n.d.	n.d.	32[1]	n.d.	n.d.
**32**	ERJ-688	4-8	n.d.	n.d.	n.d.	8	n.a.	n.a.
**33**	ERJ-710	8-16	n.d.	n.d.	n.d.	>32	n.a.	n.a.
**34**	ERJ-714	8	n.d.	n.d.	n.d.	1[1]	n.a.	n.a.
**35**	ERJ-713	8	n.d.	n.d.	n.d.	>32	n.a.	n.a.
**36**	ERJ-712	8	n.d.	n.d.	n.d.	>32	n.a.	n.a.
**37**	ERJ-715	8	n.d.	n.d.	n.d.	2	n.a.	n.a.
**38**	ERJ-716	>64	n.d.	n.d.	n.d.	>32	n.a.	n.a.
**41**	ERJ-192	>64	n.d.	n.d.	n.d.	n.d.	n.d.	n.d.
**42**	ERJ-153	>64	n.d.	n.d.	n.d.	n.d.	n.d.	n.d.
**43**	ERJ-188	32-64	n.d.	n.d.	n.d.	n.d.	n.d.	n.d.
**44**	ERJ-157	>64	n.d.	n.d.	n.d.	n.d.	n.d.	n.d.
**45**	ERJ-183	>64	n.d.	n.d.	n.d.	n.d.	n.d.	n.d.
**46**	ERJ-184	>64	n.d.	n.d.	n.d.	n.d.	n.d.	n.d.

[1]Tested in biological duplicates each with two technical replicates, all other derivatives tested in biological triplicates.
[2]Tested in duplicate.

2. Synthesis

1.1 General Methods and Materials

Unless otherwise stated, all chemicals were of reagent grade and purchased from *Sigma- Aldrich, Merck, Acros, Fluorochem, TCI, abcr, Fisher Scientific,* or *Honeywell*. Fidaxomicin was either isolated from cultures of *Actinoplanes deccanensis* (WT, ATCC 21983)[3] or purchased from commercial suppliers (*BOC Sciences* or *Biosynth Carbosynth*). DIPEA and TEA were distilled over calcium hydride in oven-dried glassware under nitrogen and stored under argon. Reactions were carried out under protecting gas (N_2 or Ar). Solvents for reactions were of p.a. grade. Evaporation of solvents *in vacuo* was carried out on a rotary evaporator at 40 °C (45-50 °C for mixtures containing water or DMF) bath temperature and appropriate pressure. Flash column chromatography was performed with silica gel (0.040-0.063 mm, *Merck*). Unless otherwise specified, reaction progress was monitored by thin layer chromatography (TLC). TLC was carried out on TLC silica gel 60 F_{254} (*Merck*). Compounds were visualised using UV, potassium permanganate solution, or CAM stain.

Ultra-high performance liquid chromatography coupled to mass spectrometry (UHPLC-MS): *Ultimate 3000 LC* instrument (*Thermo Fisher Scientific*) coupled to a triple quadrupole *Quantum Ultra EMR* MS (*Thermo Fisher Scientific*) using a reversed-phase column (*Kinetex*® EVO C18; 1.7 µm; 100 Å, 50 x 2.1 mm; *Phenomenex*). The LC was equipped with an *HPG-3400RS* pump, a *WPS-3000TRS* autosampler, a *TCC-3000RS* column oven and a Vanquish *DAD* detector (all *Thermo Fisher Scientific*). The following solvents were applied: H_2O + 0.1% HCOOH (A), MeCN + 0.1% HCOOH (B). Samples were prepared using HPLC grade solvents (MeCN, MeOH, H_2O) and filtered over a 4 mm syringe filter, PTFE (hydrophilic), pore size: 0.22 µm obtained from *BGB Analytik AG*. The MS was equipped with an H-ESI II ion source. The source temperature was 250 °C, the capillary temperature 270 °C and capillary voltage 3500 V, and datasets were acquired at resolution 0.7 on Q3 in centroid mode.

High performance liquid chromatography (HPLC): *Prominence* modular HPLC instrument (*Shimadzu*) coupled to an *SPD-20A* UV/Vis detector (*Shimadzu*) using a reversed-phase column (*Gemini NX* C18, 3 µm, 10 Å, 150 mm × 4.6 mm) for analytical HPLC, and a reversed-phase column (*Gemini NX* C18, 5 µm, 110 Å, 250 mm × 21.2 mm; for preparative HPLC. The LC was equipped with a *CBM-20A* system controller, *LC-20A* solvent delivery unit, a *DGU-20A* degassing unit, *FRC-10A* fraction collector (all *Shimadzu*). The following solvents were used: H_2O + 0.1% HCOOH (A), MeCN + 0.1% HCOOH (B). Solvent (A) was degassed by sonication prior to use.

Infrared spectra (IR): *SpectrumTwo FT-IR Spectrometer* (*Perkin–Elmer*) equipped with a Specac Golden GateTM *ATR* (attenuated total reflection) accessory; applied as thin films; 1/λ in cm^{-1}.

Nuclear magnetic resonance spectra (NMR): ^{1}H NMR spectra were recorded in $CDCl_3$, acetone-d_6, DMSO-d_6, methanol-d_4 or acetonitrile-d_3 on the instruments *Bruker AV-500* (500 MHz) or *AV-400* (400 MHz); chemical shift δ in ppm relative to solvent signals (δ = 7.26 ppm for $CDCl_3$, 2.05 ppm for acetone-d_6, 2.50 ppm for DMSO-d_6, 3.31 ppm for methanol-d_4 and 1.94 ppm for acetonitrile-d_3)[6], coupling constant *J* is given in Hz. ^{13}C NMR spectra were recorded in $CDCl_3$, acetone-d_6, DMSO-d_6, methanol-d_4 or acetonitrile-d_3 on the instruments *Bruker AV-500* (126 MHz) or *AV-400* (101 MHz); chemical shift δ in ppm relative to solvent signals (δ = 77.16 ppm for $CDCl_3$, 29.84 ppm for acetone-d_6, 39.52 ppm for DMSO-d_6, 49.00 ppm for methanol-d_4 and 1.32 ppm for acetonitrile-d_3)[4].

High-resolution-electrospray ionization mass spectra (HRMS): *On flow injection*: High-resolution mass spectra were acquired on a *QExactive* instrument (*ThermoFisher Scientific*, Bremen, Germany) equipped with a heated electrospray (ESI) ionization source and connected to a *Dionex Ultimate 3000* UHPLC system (*ThermoFischer Scientifics*, Germering, Germany). The samples were dissolved in MeOH at a concentration of ca. 50 µg mL^{-1} thereof 1 µL was injected on-flow with a *XRS* autosampler (*CTC*, Zwingen, Switzerland). The mobile phase (120 µL mL^{-1} flow rate) consisting of MeOH + 0.1% HCOOH or $MeCN/H_2O$ 2:8 + 0.1% HCOOH was chosen according to the solubility. Ion source parameters were set as follow: spray voltage 3.0 kV; capillary temperature 280 °C; sheath gas 30 L min^{-1}; aux gas 8 L min^{-1}; s-lens RF level 55.0; and aux gas temperature 250 °C. Full scan MS were acquired in the alternating (+)/(-)-ESI mode and over the ranges m/z = 80-1200, 133-2000, or 200-3000 at 70000 resolution (full width half maximum) and with automatic gain control (AGC) target of $3.00E^{+06}$. The maximum allowed ion transfer time (IT) was 30 ms. Masses were calibrated below 2 ppm accuracy between m/z = 130.06619 and 1621.96509 in the positive and between 265.14790 and 1779.96528 in the negative ESI mode using the *Pierce*® ESI calibration solutions (*ThermoFisher Scientific*, Rockford, USA). Additionally, contaminations of erucamide (m/z = 338.34174, (+)-ESI) and palmitic acid (m/z = 255.23295, (–)-ESI) were used as lock masses in (+)-and (–)-ESI, respectively.

1.2 General Procedures

General Procedure for the Allylic Substitution using Primary Amines (GP1)

To an HPLC vial was added fidaxomicin (**1**, 1.0 eq.), primary amine (5.0 eq.), $[Pd(cinnamyl)Cl]_2$ (10 mol%) and dppf (20 mol%). The vial was capped, evacuated and backfilled with nitrogen three times, and then dry THF (0.038 M) was added. The reaction was heated under stirring to 60 °C. Reaction progress was monitored by UHPLC-MS. After UHPLC-MS showed full conversion, the reaction mixture was cooled to RT, 3-mercaptopropyl-functionalised silica was added, and the mixture was stirred at RT until no Pd-complexes were observed by UHPLC-MS. The mixture was filtered through a pad of celite, eluted with DCM or MeOH (2 mL) and concentrated *in vacuo*. The resulting residue was dissolved in MeCN (1 mL) and eluted through a C18 SPE cartridge with MeCN (8 mL). The filtrate was concentrated *in vacuo* and the resulting mixture purified by preparative RP-HPLC to yield the product after concentration *in vacuo*.

General Procedure for the Removal of Boc Groups with Acid (GP2)

Boc-protected amines were deprotected according to literature procedure.[5]

The resulting amine trifluoroacetates, hydrochlorides, and hydrobromides were converted to the freebase prior to use in Tsuji-Trost reactions by stirring with Amberlyst A-21 resin in DCM/MeOH 1:1 for 30 min followed by filtration, washing with DCM and concentration *in vacuo* to yield the crude freebase. Amberlyst-A21 resin (100 g) was prepared by washing with MeOH (100 mL), stirring for 5 min and filtration. This was repeated with dry THF (100 mL) and dry DCM (100 mL). The resulting resin was dried under high-vacuum for 16 h and stored in the dark.

Alternatively, amine trifluoroacetates, hydrochlorides, and hydrobromides can be converted to the corresponding freebase by extraction, provided the amine is lipophilic enough to partition into the DCM layer and does not contain acidic protons. If applicable, this process generally gave better results than treatment with Amberlyst A-21. The amine salt was partitioned between DCM and a sodium bicarbonate solution (sat. aq.). The organic layer was separated, and the aqueous layer was extracted three times using DCM. The combined organic layers were dried over sodium sulfate, filtered, and concentrated *in vacuo* to yield the freebase.

General Procedure for the Thermal Removal of Boc Groups (GP3)

Boc-protected amines were dissolved in 1,2-dichlorobenzene (4.5 mL/mmol) in a microwave vial. Anisole (1.0 eq.) was added. The reaction mixture was heated in a microwave reactor (Monowave 450) to 120 °C for 10 min. The reaction mixture was diluted with EtOAc (10 mL), washed with

brine (10 mL), dried over sodium sulfate, filtered, and concentrated *in vacuo* to yield the primary amines which were directly used in the subsequent allylic substitution.

General Procedure for Allyl Deprotection (GP4)

The allyl protected compound and $Pd(PPh_3)_4$ (5-10 mol%) were dissolved in dry THF (0.2 M) under nitrogen and the stirred mixture was cooled to 0 °C. Morpholine (1.1 eq. per allyl group) was added to the solution and the reaction mixture was stirred at 0 °C until TLC or UHPLC-MS indicated full conversion (typically 0.5-3 h). The reaction mixture was diluted with a hydrochloric acid solution (1 M aq.), hydrochloric acid solution (0.1 M aq.), or an ammonium chloride solution (sat. aq.) depending on the acid sensitivity of the product. The aqueous layer was extracted with EtOAc, and the combined organic layers were washed with brine, dried over sodium sulfate, filtered, and concentrated *in vacuo*. The resulting residue was used directly in the next step or purified *via* flash column chromatography.

General Procedure for Oxadiazole Formation (GP5)

The acyl hydrazide was (1.0 eq.) dissolved in DCM (0.11 M) at 30 °C. Then, triethylamine (3.0 eq.) and *p*-toluenesulfonyl chloride (1.2 eq.) were added sequentially. The mixture was stirred at 30 °C for 4 h. The reaction mixture was diluted with water and extracted with EtOAc. The combined organic layers were washed with brine, dried over Na_2SO_4, filtered, and concentrated *in vacuo*. The resulting mixture was purified *via* flash column chromatography to yield the oxadiazole.

General Procedure for Sulfamates from Fluorosulfates (GP6)

Based on a modified literature procedure.[6] To a dry HPLC vial was added fluorosulfate (1.0 eq.) and HOBt (1.0 eq.). Dry DMSO (0.1 M) was added followed by amine (3.0-5.0 eq.). The reaction mixture was stirred at RT until full consumption of starting material was observed by UHPLC-MS. Then, the reaction mixture was diluted with one volume of MeCN and injected directly into a preparative RP-HPLC. The sulfamates were obtained after lyophilisation of pure fractions.

1.3 Synthesis of *N*-Nucleophiles and Fidaxomicin Derivatives

1.3.1 General Considerations

Three main sets of conditions are recommended depending on class of nucleophile:

Conditions A:

Nucleophile (3.0 eq.), $Pd(PPh_3)_4$ (5-50 mol%) in *i*-PrOH/H_2O 5:1 at 40-50 °C, in MeOH at 60 °C, or in THF at 40-60 °C.

Conditions B:

Nucleophile (3.0 eq.), $[Pd(cinnamyl)Cl]_2$ (5-20 mol%), dppf (10-40 mol%) in dry THF (190 mM) at 60 °C.

Conditions C:

Nucleophile (5.0 eq.), $[Pd(cinnamyl)Cl]_2$ (10 mol%), dppf (20 mol%), dry THF (38 mM) at 60 °C.

Conditions A are preferred for secondary amines, **Conditions B** are preferred for 2-aminothiazoles, anilines, and 2-aminopyridines. **Conditions C** should be used for primary amines to suppress dimer formation.

Setting up the reaction:

Solids are weighed into an appropriate glass vessel depending on scale (HPLC vial or microwave vial with PTFE septa). The septum is pierced with a needle connected to a Schlenk line. The reaction vessel is evacuated and backfilled with nitrogen or argon three times. Degassed solvents are added through the septum *via* needle, followed by the nucleophile (if liquid). The needle is removed, and the reaction vessel is placed in a pre-heated aluminium block or oil bath. The reaction should be stirred at fast speeds (ideally 1000 rpm). Reaction progress is best monitored by UHPLC-MS (at 270 nm) monitoring disappearance of starting material or stalling of reaction.

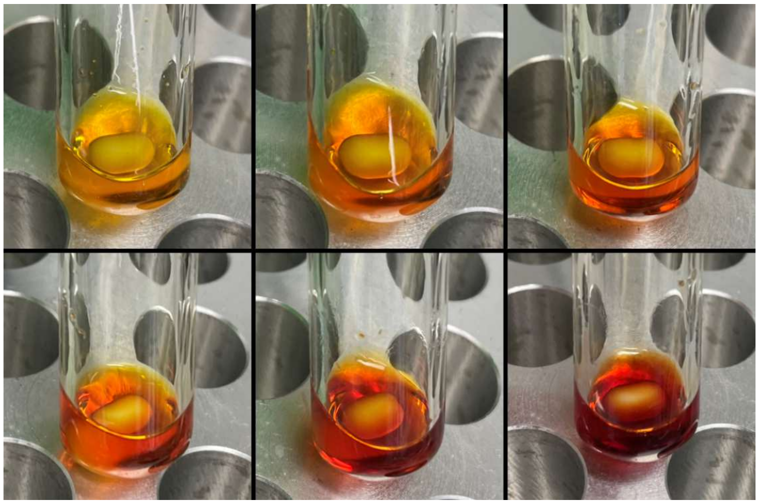

Supplementary Figure 5. Typical progression of reactions using [Pd(cinnamyl)Cl]$_2$. Colour and speed of colour change depends on the nucleophile used. Times shown: 0 min, 1 min, 2 min, 7 min, 54 min. 4 h 40 min.

Removal of palladium:

Once full conversion is reached the reaction mixture is diluted (with reaction solvent or other suitable solvent such as DCM, MeCN, THF). And >3.0 eq. of functionalised scavenging silica gel is added. Here, 3-mercaptopropyl functionalized silica gel was used but cysteine- or dimercaptotriazine-functionalised silica gel are also suitable. After stirring for ~2 h at RT (UHPLC can also be used to monitor presence of Pd-complexes) the suspension is filtered through silica gel and eluted with an appropriate solvent (*f.e.* DCM, MeCN, THF) and the filtrate is concentrated *in vacuo*.

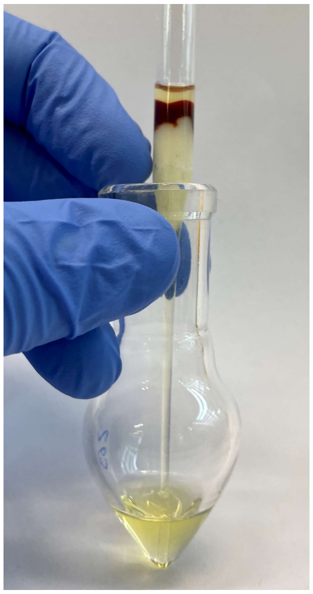

Supplementary Figure 6. Pasteur-pipette with cotton plug, silica gel, and 3-mercaptopropyl-functionalised silica gel.

1.3.2 Secondary Amines

Preparation of Amine 2

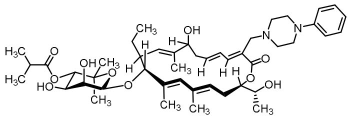

In a microwave vial a suspension of fidaxomicin (**1**, 50.0 mg, 47.3 µmol, 1.0 eq.), $Pd(PPh_3)_4$ (27.3 mg, 23.7 µmol, 50 mol%) and *N*-phenylpiperazine (23.0 mg, 0.142 mmol, 3.0 eq.) in *i*-PrOH/water 5:1 (0.6 mL) was sparged with nitrogen for 30 sec and the reaction headspace was purged with nitrogen for 1 min. The vial was capped and the reaction was heated to 40 °C. Reaction progress was monitored by UHPLC-MS. After 4 h the reaction mixture was cooled to RT, diluted with MeCN (2 mL) and eluted through a C18 SPE cartridge with MeCN (8 mL). The filtrate was concentrated *in vacuo* and the crude mixture purified by preparative RP-HPLC ([Gemini NX, C18, 5 µ, 110 Å, 250 mm × 21.2 mm, solvent A: H_2O + 0.1% HCOOH, solvent B: MeCN + 0.1% HCOOH, 20 mL/min; LC time program (min – %B): 5 min – 2%, 7 min – 25%, 40 min – 35%]) to yield, after lyophilisation, the *N*-phenylpiperazine **2** (t_R = 17.3 min, 24.7 mg, 30.5 µmol, 65%) as a colourless solid.

Analytical Data of Amine 2

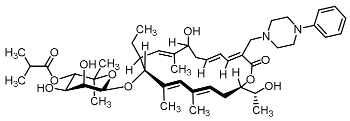

^{1}H NMR (400 MHz, acetone-*d$_6$*) δ 7.25 – 7.12 (m, 3H), 6.92 (d, *J* = 8.2 Hz, 2H), 6.76 (t, *J* = 7.3 Hz, 1H), 6.63 (dd, *J* = 15.0, 11.4 Hz, 1H), 5.94 – 5.86 (m, 1H), 5.85 – 5.81 (m, 1H), 5.66 – 5.57 (m, 1H), 5.20 (d, *J* = 10.7 Hz, 1H), 4.99 (d, *J* = 10.0 Hz, 1H), 4.77 (s, 1H), 4.74 – 4.68 (m, 1H), 4.25 (s, 1H), 4.06 – 3.98 (m, 1H), 3.95 (d, *J* = 3.1 Hz, 1H), 3.76 – 3.69 (m, 2H), 3.44 (d, *J* = 12.6 Hz, 1H), 3.23 (d, *J* = 12.6 Hz, 1H), 3.12 (t, *J* = 5.1 Hz, 4H), 3.08 – 2.80 (m, 2H), 2.81 – 2.71 (m, 1H), 2.71 – 2.63 (m, 2H), 2.62 – 2.49 (m, *J* = 5.1, 4.1 Hz, 4H), 2.49 – 2.37 (m, 1H), 1.99 – 1.91 (m, 1H), 1.81 (s, 3H), 1.76 (s, 3H), 1.65 (s, 3H), 1.27 – 1.22 (m, 1H), 1.22 – 1.10 (m, 12H), 1.09 (s, 3H), 0.83 (t, *J* = 7.4 Hz, 3H) ppm;

^{13}C NMR (126 MHz, acetone-*d$_6$*) δ 176.8, 168.9, 152.5, 143.9, 141.6, 136.9, 136.1, 136.0, 133.9, 129.7, 128.6, 126.8, 126.3, 124.0, 119.8, 116.5, 96.7, 93.3, 78.1, 75.7, 73.7, 72.9, 72.7, 70.1, 67.8, 53.73, 53.68, 49.7, 42.1, 37.2, 34.8, 28.7, 28.4, 26.5, 20.7, 19.4, 19.2, 18.6, 17.4, 15.2, 13.8, 11.2 ppm; **IR** (thin film): ν 3452w, 2974w, 2933w, 2875w, 1735m, 1698s, 1640w, 1600m, 1497w, 1454m, 1383m, 1346m, 1295m, 1243s, 1206m, 1147s, 1076s, 1032s, 1005s, 950w, 898m, 812w, 797w, 759m, 693m, 584w, 524w, 478w cm^{-1};

HRMS (ESI) for $C_{46}H_{69}O_{10}N_2^+$ $[M+H]^+$: calculated: 809.49467; found: 809.49504.

ERJ-138

Preparation of Amine 3

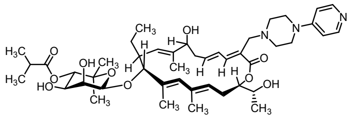

In a microwave vial suspension of fidaxomicin (**1**, 50.0 mg, 47.3 µmol, 1.0 eq.), $Pd(PPh_3)_4$ (27.3 mg, 23.7 µmol, 50 mol%) and 1-(pyridin-4-yl)piperazine (23.2 mg, 0.142 mmol, 3.0 eq.) in *i*-PrOH/water 5:1 (0.6 mL) was sparged with nitrogen for 30 sec and the reaction headspace was purged with nitrogen for 1 min. The vial was capped and the reaction was heated to 40 °C. Reaction progress was monitored by UHPLC-MS. After 19 h the reaction mixture was cooled to RT, diluted with MeCN (2 mL) and eluted through a C18 SPE cartridge with MeCN (8 mL). The filtrate was concentrated *in vacuo* and the crude mixture purified by preparative RP-HPLC ([Gemini NX, C18, 5 µ, 110 Å, 250 mm × 21.2 mm, solvent A: H_2O + 0.1% HCOOH, solvent B: MeCN + 0.1% HCOOH, 20 mL/min; LC time program (min – %B): 5 min – 2%, 7 min – 10%, 40 min – 20%]) to yield, after lyophilisation, the *N*-(pyridin-4-yl)piperazine **3** in an inseparable mixture with the corresponding 2'-ester (t_R = 24.0 min, 13.8 mg, 17.0 µmol, 36%) as a colourless solid.

Analytical Data of Amine 3

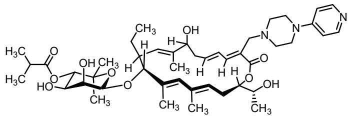

^{1}H NMR (400 MHz, acetone-*d$_6$*) δ 8.20 – 8.14 (m, 2H), 7.17 (d, *J* = 11.4 Hz, 1H), 6.82 – 6.74 (m, 2H), 6.62 (dd, *J* = 15.0, 11.4 Hz, 1H), 5.90 (ddd, *J* = 14.8, 9.5, 4.8 Hz, 1H), 5.83 (s, 1H), 5.62 (s, 1H), 5.20 (d, *J* = 10.8 Hz, 1H), 5.00 (d, *J* = 10.1 Hz, 1H), 4.77 (s, 1H), 4.71 (td, *J* = 6.0, 4.1 Hz, 1H), 4.25 (s, 1H), 4.05 – 3.98 (m, 1H), 3.96 (d, *J* = 3.3 Hz, 1H), 3.76 – 3.70 (m, 2H), 3.44 (d, *J* = 12.7 Hz, 1H), 3.35 – 3.31 (m, 6H), 3.24 (d, *J* = 12.6 Hz, 1H), 2.75 (dt, *J* = 13.8, 6.8 Hz, 1H), 2.70 – 2.63 (m, 1H), 2.60 – 2.37 (m, 7H), 2.00 – 1.91 (m, 1H), 1.80 (s, 3H), 1.75 (s, 3H), 1.65 (s, 3H), 1.19 – 1.11 (m, 12H), 1.09 (s, 3H), 0.83 (t, *J* = 7.4 Hz, 3H) ppm;

^{13}C NMR (126 MHz, acetone-*d$_6$*) δ 175.9, 167.9, 155.1, 149.5, 143.2, 141.0, 136.0, 135.2, 132.9, 127.7, 125.8, 125.1, 123.1, 108.3, 95.9, 92.4, 77.3, 74.8, 72.9, 72.0, 71.8, 69.2, 66.9, 52.7, 52.2, 45.9, 41.2, 36.3, 33.9, 29.7, 29.5, 27.8, 27.5, 25.6, 19.8, 18.5, 18.3, 17.7, 16.5, 14.3, 12.9, 10.3 ppm;

IR (thin film): ν 3406w, 2974m, 2932m, 2874m, 1735m, 1699s, 1642m, 1601s, 1544w, 1515m, 1453m, 1384m, 1295m, 1251s, 1208m, 1150m, 1080m, 1033m, 995s, 932w, 898w, 809m, 714w, 541w cm^{-1};

HRMS (ESI) for $C_{45}H_{68}O_{10}N_3^+$ $[M+H]^+$: calculated: 810.48992; found: 810.48940.

ERJ-139

Preparation of Amine 4

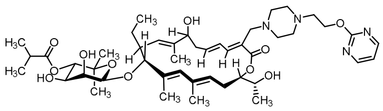

In a microwave vial a suspension of fidaxomicin (**1**, 50.0 mg, 47.3 µmol, 1.0 eq.), $Pd(PPh_3)_4$ (27.3 mg, 23.7 µmol, 50 mol%) and 2-(2-(piperazin-1-yl)ethoxy)pyrimidine (freebase obtained according to **GP2**, 29.6 mg, 0.142 mmol, 3.0 eq.) in *i*-PrOH/water 5:1 (0.6 mL) was sparged with nitrogen for 30 sec and the reaction headspace was purged with nitrogen for 1 min. The vial was capped and the reaction was heated to 40 °C. Reaction progress was monitored by UHPLC-MS. After 20 h the reaction mixture was cooled to RT, diluted with MeCN (2 mL) and eluted through a C18 SPE cartridge with MeCN (8 mL). The filtrate was concentrated *in vacuo* and the crude mixture purified by preparative RP-HPLC ([Gemini NX, C18, 5 µ, 110 Å, 250 mm × 21.2 mm, solvent A: H_2O + 0.1% HCOOH, solvent B: MeCN + 0.1% HCOOH, 20 mL/min; LC time program (min – %B): 5 min – 2%, 7 min – 20%, 40 min – 25%]) to yield, after lyophilisation, the pyrimidine **4** (t_R = 15.2 min, 13.4 mg, 15.7 µmol, 33%) as a brown solid.

Analytical Data of Amine 4

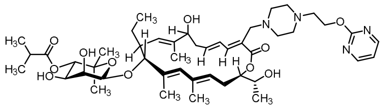

^{1}H NMR (500 MHz, acetone-d_6) δ 8.55 (d, *J* = 4.8 Hz, 2H), 7.12 (d, *J* = 11.4 Hz, 1H), 7.06 (t, *J* = 4.8 Hz, 1H), 6.58 (dd, *J* = 15.1, 11.4 Hz, 1H), 5.86 (dt, *J* = 10.1, 4.8 Hz, 1H), 5.83 (s, 1H), 5.60 (t, *J* = 8.2 Hz, 1H), 5.20 (dt, *J* = 10.6, 1.7 Hz, 1H), 4.99 (d, *J* = 10.1 Hz, 1H), 4.77 (d, *J* = 1.3 Hz, 1H), 4.69 (td, *J* = 6.1, 4.3 Hz, 1H), 4.43 (t, *J* = 6.0 Hz, 2H), 4.26 – 4.22 (m, 1H), 3.99 (t, *J* = 6.4 Hz, 1H), 3.95 (dd, *J* = 3.3, 1.2 Hz, 1H), 3.75 – 3.70 (m, 2H), 3.35 (d, *J* = 12.6 Hz, 1H), 3.14 (d, *J* = 12.6 Hz, 1H), 2.89 – 2.81 (m, 4H), 2.77 – 2.68 (m, 2H), 2.66 – 2.61 (m, 1H), 2.60 – 2.53 (m, 1H), 2.52 – 2.38 (m, 3H), 1.98 – 1.91 (m, 1H), 1.80 (s, 3H), 1.74 (s, 3H), 1.65 (s, 3H), 1.31 – 1.28 (m, 1H), 1.28 – 1.18 (m, 1H), 1.18 – 1.12 (m, 15H), 1.09 – 1.08 (m, 4H), 0.83 (t, *J* = 7.4 Hz, 3H) ppm;

^{13}C NMR (126 MHz, acetone-d_6) δ 176.8, 168.9, 166.2, 160.2, 143.7, 141.4, 136.9, 136.09, 136.05, 133.9, 128.7, 126.8, 126.5, 124.0, 116.0, 96.7, 93.3, 78.1, 75.7, 73.7, 72.9, 72.8, 70.1, 67.9, 65.7, 57.6, 54.4, 53.7, 42.1, 37.2, 34.8, 28.7, 28.5, 26.5, 20.7, 19.4, 19.2, 18.6, 17.4, 15.2, 13.8, 11.2 ppm;

IR (thin film): ν 3387m, 2973m, 2933m, 2876m, 1735m, 1699m, 1639m, 1578m, 1460m, 1428s, 1384m, 1369m, 1322m, 1249m, 1229m, 1201m, 1152s, 1077s, 1034s, 1009m, 950w, 898w, 810w, 799w, 719w, 685w, 641w, 582w, 525w, 479w, 467w, 456w cm^{-1};

HRMS (ESI) for $C_{46}H_{71}O_{11}N_4^+$ $[M+H]^+$: calculated: 855.51139; found: 855.51064.

ERJ-147

Preparation of *tert*-Butyl 4-(2-(pyrimidin-2-yloxy)ethyl)piperazine-1-carboxylate (4a)

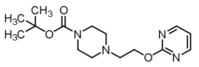

Prepared according to a literature procedure.[7] In a flame-dried flask under nitrogen a stirred solution of *tert*-butyl 4-(2-hydroxyethyl)piperazin-4-ium-1-carboxylate (311 mg, 1.35 mmol, 1.0 eq.) and 2-chloropyrimidine (155 mg, 1.35 mmol, 1.0 eq.) in DMF (7 mL) was cooled to 0 °C. A solution of NaHDMS (0.8 M in THF, 2.5 mL, 2.0 mmol, 1.5 eq.) was added dropwise. The reaction mixture turned bright orange, was allowed to warm to RT after 10 min and was then stirred at RT for 1 h. The reaction mixture was diluted with EtOAc (70 mL) and washed with a sodium bicarbonate solution (sat. aq., 50 mL), water (50 mL) and brine (50 mL). The organic layer was dried over sodium sulfate, filtered and concentrated *in vacuo*. The resulting residue was concentrated onto silica and purified using flash column chromatography (silica gel, 10% EEA (75:25:2 EtOAc:EtOH:NH_{3aq}) in pentane) to yield the pyrimidine **4a** (305 mg, 0.99 mmol, 73%) as a yellow oil.

Analytical Data of
***tert*-Butyl 4-(2-(pyrimidin-2-yloxy)ethyl)piperazine-1-carboxylate (4a)**

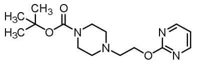

R_f = 0.25 (33% EEA (75:25:2 EtOAc:EtOH:NH_{3aq}) in pentane);

^{1}H NMR (400 MHz, $CDCl_3$) δ 8.49 (d, *J* = 4.8 Hz, 2H), 6.93 (t, *J* = 4.8 Hz, 1H), 4.54 (t, *J* = 5.8 Hz, 2H), 3.67 – 3.23 (m, 4H), 3.07 – 2.81 (m, 2H), 2.72 – 2.39 (m, 4H), 1.43 (s, 9H) ppm;

^{13}C NMR (101 MHz, $CDCl_3$) δ 165.0, 159.4, 154.7, 115.3, 79.9, 64.8, 56.9, 53.3, 43.4, 28.5 ppm;

IR (thin film): ν 3386w, 2975m, 2932m, 2864w, 2814w, 1693m, 1578m, 1565m, 1458m, 1424s, 1366m, 1320m, 1290m, 1246m, 1171m, 1130m, 1088m, 1026m, 1005m, 940w, 865w, 810m, 770m, 637w, 526w, 473w cm^{-1};

HRMS (ESI) for $C_{15}H_{24}O_3N_4Na^+$ $[M+Na]^+$: calculated: 331.17406; found: 331.17379.

ERJ-104

Preparation of Amine 5

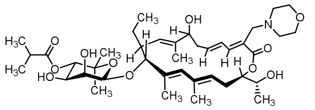

In an HPLC vial fidaxomicin (**1**, 50.0 mg, 47.3 µmol, 1.0 eq.) and $Pd(PPh_3)_4$ (5.7 mg, 4.7 µmol, 10 mol%) were evacuated and backfilled with N_2 three times. Then, *i*-PrOH/water 5:1 (0.6 mL, degassed by sparging with N_2) and morpholine (12.4 µL, 0.142 mmol, 3.0 eq.) were added sequentially. The stirred reaction mixture was heated to 50 °C. Reaction progress was monitored by UHPLC-MS. After 11 h the reaction mixture was cooled to RT, and 3-mercaptopropyl-functionalised silica gel was added. After stirring at RT for 2 h, the mixture was filtered through a plug of silica and eluted with DCM. The filtrate was concentrated *in vacuo* and the resulting residue diluted with MeCN (2 mL) and eluted through a C18 SPE cartridge with MeCN (8 mL). The filtrate was concentrated *in vacuo* and the mixture was purified twice by preparative RP-HPLC ([Gemini NX, C18, 5 µ, 110 Å, 250 mm × 21.2 mm, solvent A: H_2O + 0.1% HCOOH, solvent B: MeCN + 0.1% HCOOH, 18 mL/min; LC time program (min – %B): 5 min – 10%, 60 min – 30%]) to yield, after lyophilisation, the morpholine **3** in an inseparable mixture with the corresponding 2'-ester (t_R = 35.6 min, 21.7 mg, 29.6 µmol, 63%) as an off-white solid.

Analytical Data of Amine 5

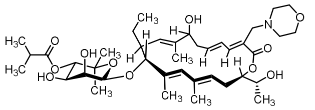

^{1}H NMR (500 MHz, acetone-*d$_6$*) δ 7.14 (d, *J* = 11.4 Hz, 1H), 6.59 (dd, *J* = 15.1, 11.3 Hz, 1H), 5.87 (ddd, *J* = 14.7, 9.5, 4.8 Hz, 1H), 5.83 (s, 1H), 5.60 (t, *J* = 8.2 Hz, 1H), 5.23 – 5.14 (m, 1H), 4.99 (d, *J* = 10.1 Hz, 1H), 4.77 (s, 1H), 4.73 – 4.66 (m, 1H), 4.28 – 4.23 (m, 1H), 4.03 – 3.97 (m, 2H), 3.78 – 3.69 (m, 1H), 3.59 – 3.49 (m, 4H), 3.35 (d, *J* = 12.6 Hz, 1H), 3.16 (d, *J* = 12.6 Hz, 1H), 2.74 (dt, *J* = 13.9, 6.8 Hz, 1H), 2.69 – 2.59 (m, 2H), 2.61 – 2.51 (m, 1H), 2.51 – 2.31 (m, 5H), 1.98 – 1.91 (m, 1H), 1.80 (d, *J* = 1.3 Hz, 3H), 1.74 (s, 3H), 1.65 (s, 3H), 1.31 – 1.18 (m, 3H), 1.16 (s, 12H), 1.08 (s, 3H), 0.83 (t, *J* = 7.4 Hz, 3H) ppm;

^{13}C NMR (126 MHz, acetone-*d$_6$*) δ 176.8, 168.8, 144.0, 141.6, 136.9, 136.1, 136.1, 133.9, 128.6, 126.7, 126.0, 124.0, 96.7, 93.3, 78.1, 75.7, 73.7, 72.9, 72.8, 70.2, 67.9, 67.5, 54.2, 54.1, 42.1, 37.2, 34.8, 28.7, 28.4, 26.5, 20.7, 19.4, 19.2, 18.6, 17.4, 15.2, 13.8, 11.2 ppm;

IR (thin film): ν 3453w, 2972m, 2930m, 2872w, 1737m, 1700m, 1641w, 1455w, 1405w, 1384w, 1369w, 1348w, 1333w, 1289w, 1254m, 1234m, 1207m, 1151s, 1115s, 1071s, 1034s, 1006s, 933w, 911w, 898w, 864w, 798w cm^{-1};

HRMS (ESI) for $C_{40}H_{64}O_{11}N^+$ $[M+H]^+$: calculated: 734.44739; found: 734.44724.

ERJ-636

Preparation of Amine 6

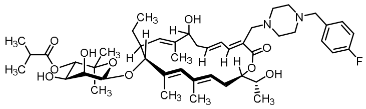

In a microwave vial equipped with a magnetic stirring bar a suspension of fidaxomicin (**1**, 50.0 mg, 47.3 µmol, 1.0 eq.), $Pd(PPh_3)_4$ (27.3 mg, 23.7 µmol, 50 mol%) and 1-(4-fluorobenzyl)piperazine (27.6 mg, 0.142 mmol, 3.0 eq.) in *i*-PrOH/water 5:1 (0.6 mL) was sparged with nitrogen for 30 sec and the reaction headspace was purged with nitrogen for 1 min. The vial was capped and the reaction was heated to 40 °C. Reaction progress was monitored by UHPLC-MS. After 4 h the reaction mixture was cooled to RT, diluted with MeCN (2 mL) and eluted through a C18 SPE cartridge with MeCN (8 mL). The filtrate was concentrated *in vacuo* and the crude mixture purified by preparative RP-HPLC ([Gemini NX, C18, 5 µ, 110 Å, 250 mm × 21.2 mm, solvent A: H_2O + 0.1% HCOOH, solvent B: MeCN + 0.1% HCOOH, 20 mL/min; LC time program (min – %B): 5 min – 2%, 7 min – 25%, 40 min – 35%]) to yield, after lyophilisation, the *N*-(*p*-fluorobenzyl)piperazine **6** (t_R = 15.4 min, 25.8 mg, 30.7 µmol, 65%) as a colourless solid.

Analytical Data of Amine 6

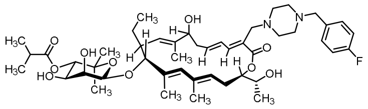

^{1}H NMR (400 MHz, acetone-d_6) δ 7.38 – 7.31 (m, 2H), 7.12 (d, J = 11.4 Hz, 1H), 7.10 – 7.01 (m, 2H), 6.57 (dd, J = 15.0, 11.4 Hz, 1H), 5.87 (dt, J = 10.4, 5.0 Hz, 1H), 5.82 (s, 1H), 5.60 – 5.56 (m, 1H), 5.22 – 5.17 (m, 1H), 4.99 (d, J = 10.1 Hz, 1H), 4.76 (d, J = 1.2 Hz, 1H), 4.69 (td, J = 6.1, 4.3 Hz, 1H), 4.26 – 4.22 (m, 1H), 3.99 (t, J = 6.4 Hz, 1H), 3.96 – 3.94 (m, 1H), 3.75 – 3.69 (m, 2H), 3.43 (s, 2H), 3.36 (d, J = 12.6 Hz, 1H), 3.15 (d, J = 12.6 Hz, 1H), 2.74 (dt, J = 13.8, 6.7 Hz, 1H), 2.65 (td, J = 9.9, 3.2 Hz, 1H), 2.56 (dt, J = 13.9, 6.9 Hz, 1H), 2.51 – 2.26 (m, 9H), 1.99 – 1.90 (m, 1H), 1.79 (d, J = 1.2 Hz, 3H), 1.73 (d, J = 1.3 Hz, 3H), 1.64 (s, 3H), 1.32 – 1.18 (m, 2H), 1.18 – 1.11 (m, 13H), 1.08 (s, 3H), 0.82 (t, J = 7.4 Hz, 3H) ppm;

^{13}C NMR (126 MHz, acetone-d_6) δ 176.8, 168.9, 162.7 (d, J = 242.5 Hz), 143.7, 141.5, 136.9, 136.1, 136.0, 135.7 (d, J = 3.1 Hz), 133.8, 131.4 (d, J = 8.0 Hz), 128.6, 126.7, 126.4, 124.0, 115.5 (d, J = 21.2 Hz), 96.7, 93.3, 78.1, 75.7, 73.7, 72.9, 72.7, 70.1, 67.8, 62.5, 53.8, 53.71, 53.68, 42.1, 37.2, 34.8, 28.7, 28.4, 26.5, 20.7, 19.4, 19.2, 18.6, 17.4, 15.2, 13.8, 11.2 ppm;

^{19}F NMR (376 MHz, acetone-d_6) δ -117.8 ppm;

IR (thin film): ν 3451w, 2974m, 2934m, 2876m, 2821w, 1736m, 1699s, 1640m, 1603w, 1509m, 1458m, 1384m, 1370m, 1345m, 1292m, 1247s, 1221s, 1149s, 1078s, 1034s, 1007s, 949w, 898m, 841m, 822w, 797w, 768w, 713w, 509w cm^{-1};

HRMS (ESI) for $C_{47}H_{70}O_{10}N_2F^+$ $[M+H]^+$: calculated: 841.50090; found: 841.50055.

ERJ-140

Preparation of Oxadiazole 7

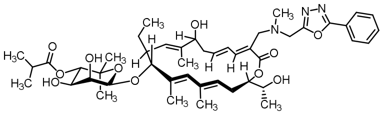

To an HPLC vial was added fidaxomicin (**1**, 100 mg, 94.5 µmol, 1.0 eq.), *N*-methyl-1-(5-phenyl-1,3,4-oxadiazol-2-yl)methanamine (53.6 mg, 0.284 mmol, 3.0 eq.), and $Pd(PPh_3)_4$ (10.9 mg, 9.5 µmol, 10 mol%). The vial was capped, evacuated and backfilled with nitrogen three times, and then dry MeOH (0.5 mL) was added. The reaction was heated under stirring to 60 °C. Reaction progress was monitored by UHPLC-MS. After 20 h the reaction mixture was cooled to RT, 3-mercaptopropyl-functionalised silica was added and the mixture was stirred at RT until no Pd-complexes were observed by UHPLC-MS. The reaction mixture was diluted with MeCN (2 mL) and eluted through a C18 SPE cartridge with MeCN (8 mL). The filtrate was concentrated *in vacuo* and the resulting crude mixture purified by preparative RP-HPLC ([Gemini NX, C18, 5 µ, 110 Å, 250 mm × 21.2 mm, solvent A: H_2O + 0.1% HCOOH, solvent B: MeCN + 0.1% HCOOH, 20 mL/min; LC time program (min – %B): 7 min – 20%, 60 min – 35%]) to yield, after lyophilization, the oxadiazole **7** (t_R = 38.7 min, 36.6 mg, 43.8 µmol, 46%) as an off-white solid.

Analytical Data of Oxadiazole 7

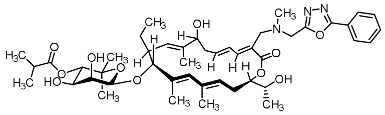

^{1}H NMR (500 MHz, acetone-*d$_6$*) δ 8.07 – 8.00 (m, 2H), 7.64 – 7.57 (m, 3H), 7.04 (d, *J* = 11.4 Hz, 1H), 6.65 – 6.56 (m, 1H), 5.86 – 5.74 (m, 2H), 5.57 – 5.49 (m, 1H), 5.16 (dt, *J* = 10.6, 1.6 Hz, 1H), 4.98 (d, *J* = 10.1 Hz, 1H), 4.75 (d, *J* = 1.3 Hz, 1H), 4.68 – 4.60 (m, 1H), 4.22 (s, 1H), 4.12 – 4.04 (m, 2H), 3.97 – 3.91 (m, 2H), 3.87 – 3.80 (m, 1H), 3.74 – 3.66 (m, 2H), 3.5, 3.4 (AB$_q$, 2H, J_{AB} = 12.9 Hz), 2.80 – 2.73 (m, 1H), 2.65 – 2.50 (m, 3H), 2.44 (dddd, *J* = 15.1, 9.7, 4.4, 1.0 Hz, 1H), 2.38 (s, 3H), 2.34 (ddd, *J* = 13.6, 8.5, 4.4 Hz, 1H), 2.00 – 1.89 (m, 1H), 1.75 (d, *J* = 1.4 Hz, 3H), 1.68 (d, *J* = 1.4 Hz, 3H), 1.64 – 1.60 (m, 3H), 1.25 – 1.17 (m, 1H), 1.18 – 1.11 (m, 12H), 1.07 (s, 3H), 0.81 (t, *J* = 7.4 Hz, 3H) ppm;

^{13}C NMR (126 MHz, acetone-d$_6$) δ 176.8, 168.6, 165.5, 165.2, 144.0, 141.9, 136.9, 136.3, 135.9, 134.0, 132.6, 130.0, 128.7, 127.6, 127.1, 126.3, 125.1, 124.0, 96.6, 93.3, 78.2, 75.7, 73.7, 73.0, 72.8, 70.2, 67.7, 52.4, 51.2, 43.0, 42.2, 37.3, 34.8, 28.7, 27.8, 26.4, 20.3, 19.4, 19.2, 18.6, 17.3, 15.3, 13.8, 11.2 ppm;

IR (thin film): ν 3428m, 2974m, 2933m, 2874m, 1736m, 1700s, 1640w, 1609w, 1553w, 1485w, 1450m, 1384m, 1369m, 1294w, 1247m, 1198m, 1148m, 1072s, 1033s, 1011m, 950w, 899w, 796w, 776w, 713m, 692w cm^{-1};

HRMS (ESI) for $C_{46}H_{66}O_{11}N_3^+$ $[M+H]^+$: calculated: 836.46919; found: 836.46960.

ERJ-302

1.3.3 Aminothiazoles

Preparation of Aminothiazole 8

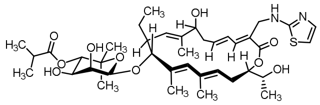

To an HPLC vial was added fidaxomicin (**1**, 100.0 mg, 94.5 µmol, 1.0 eq.), 2-aminothiazole (28.4 mg, 0.284 mmol, 3.0 eq.), [Pd(cinnamyl)Cl]$_2$ (4.9 mg, 9.5 µmol, 10 mol%) and dppf (10.5 mg, 18.9 µmol, 20 mol%). The vial was capped, evacuated and backfilled with nitrogen three times and then dry THF (0.5 mL) was added. The reaction was heated under stirring to 60 °C. Reaction progress was monitored by UHPLC-MS. After 2 h the reaction mixture was cooled to RT, 3-mercaptopropyl-functionalised silica was added, and the mixture was stirred at RT until no Pd-complexes were observed by UHPLC-MS. The reaction mixture was diluted with MeCN (2 mL) and eluted through a C18 SPE cartridge with MeCN (8 mL). The filtrate was concentrated *in vacuo* and the crude mixture purified by preparative RP-HPLC ([Gemini NX, C18, 5 µ, 110 Å, 250 mm × 21.2 mm, solvent A: H_2O + 0.1% HCOOH, solvent B: MeCN + 0.1% HCOOH, 20 mL/min; LC time program (min – %B): 7 min – 10%, 60 min – 25%]) to yield, after concentration *in vacuo*, the 2-aminothiazole **8** (t_R = 40.0 min, 26.5 mg, 35.5 µmol, 38%) as an off-white solid.

Analytical Data of Aminothiazole 8

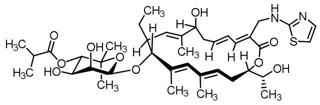

^{1}H NMR (500 MHz, acetone-*d$_6$*) δ 7.15 (d, *J* = 11.4 Hz, 1H), 7.04 (d, *J* = 3.7 Hz, 1H), 6.76 – 6.67 (m, 1H), 6.54 (d, *J* = 3.6 Hz, 1H), 5.91 (ddd, *J* = 14.6, 9.6, 4.6 Hz, 1H), 5.82 (s, 1H), 5.61 – 5.57 (m, 1H), 5.20 (dt, *J* = 10.6, 1.6 Hz, 1H), 4.99 (d, *J* = 10.1 Hz, 1H), 4.76 (d, *J* = 1.3 Hz, 1H), 4.70 (td, *J* = 5.9, 4.3 Hz, 1H), 4.30 – 4.21 (m, 3H), 4.07 – 3.98 (m, 1H), 3.96 (dd, *J* = 3.4, 1.2 Hz, 1H), 3.76 – 3.69 (m, 2H), 2.80 – 2.70 (m, 1H), 2.70 – 2.60 (m, 2H), 2.59 – 2.47 (m, 2H), 2.41 (ddd, *J* = 13.8, 8.9, 4.4 Hz, 1H), 1.99 – 1.89 (m, 1H), 1.79 (d, *J* = 1.3 Hz, 3H), 1.74 – 1.70 (m, 3H), 1.66 – 1.61 (m, 3H), 1.29 – 1.22 (m, 1H), 1.17 – 1.11 (m, 12H), 1.08 (s, 3H), 0.83 (t, *J* = 7.5 Hz, 3H) ppm;

^{13}C NMR (126 MHz, acetone-*d$_6$*) δ 176.8, 169.9, 168.3, 143.9, 142.5, 139.7, 136.8, 136.2, 136.0, 133.9, 128.6, 126.5, 126.0, 124.0, 107.1, 96.7, 93.3, 78.4, 75.7, 73.7, 72.9, 72.8, 70.1, 67.7, 42.1, 41.2, 37.3, 34.8, 28.7, 28.2, 26.5, 20.6, 19.4, 19.2, 18.6, 17.4, 15.2, 13.8, 11.2 ppm;

IR (thin film): ν 3387m, 2975m, 2933m, 2874m, 1692s, 1641m, 1540m, 1468m, 1385m, 1303m, 1254s, 1210s, 1152s, 1074s, 1033s, 898m, 796w, 688w, 613w, 527w, 462w cm^{-1};

HRMS (ESI) for $C_{39}H_{59}O_{10}N_2S^+$ $[M+H]^+$: calculated: 747.38849; found: 747.38783.

ERJ-231 ERJ-309b

Preparation of Aminothiazole 9 and Isomer 9i

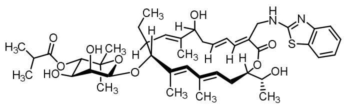
9

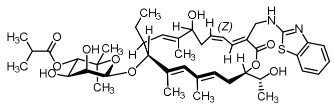
9i

To a microwave vial was added fidaxomicin (**1**, 100 mg, 94.5 µmol, 1.0 eq.), 2-aminobenzothiazole (42.6 mg, 0.284 mmol, 3.0 eq.), $[Pd(allyl)Cl]_2$ (17.3 mg, 47.3 µmol, 50 mol%) and dppf (52.4 mg, 94.5 µmol, 100 mol%). The vial was capped, evacuated and backfilled with nitrogen three times and then dry MeOH (0.5 mL) was added. The reaction was heated under stirring to 60 °C. Reaction progress was monitored by UHPLC-MS. After 16 h the reaction mixture was cooled to RT, diluted with MeCN (2 mL) and eluted through a C18 SPE cartridge with MeCN (8 mL). The filtrate was concentrated *in vacuo* and the crude mixture purified by preparative RP-HPLC ([Gemini NX, C18, 5 µ, 110 Å, 250 mm × 21.2 mm, solvent A: H_2O + 0.1% HCOOH, solvent B: MeCN + 0.1% HCOOH, 20 mL/min; LC time program (min – %B): 15 min – 30%, 100 min – 70%]) to yield, after concentration *in vacuo*, the 2-aminobenzothiazole **9** (t_R = 32.6 min, 26.0 mg, 33.1 µmol, 35%) as a red-brown solid and the *Z*-isomer **9i** (t_R =37.7 min, 9.6 mg, 12 µmol, 13%) as a brown solid.

Analytical Data of Aminothiazole 9

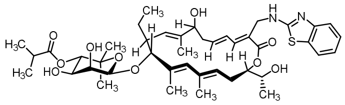

^{1}H NMR (500 MHz, acetonitrile-d_3) δ 8.24 (s, 1H), 7.60 (d, *J* = 7.9 Hz, 1H), 7.44 (d, *J* = 8.0 Hz, 1H), 7.25 (t, *J* = 7.7 Hz, 1H), 7.10 (d, *J* = 11.4 Hz, 1H), 7.05 (t, *J* = 7.6 Hz, 1H), 6.73 (dd, *J* = 14.9, 11.5 Hz, 1H), 6.50 (s, 1H, NH), 5.85 (ddd, *J* = 14.8, 10.0, 4.3 Hz, 1H), 5.76 (s, 1H), 5.50 (t, *J* = 8.3 Hz, 1H), 5.45 (s, 1H), 5.00 (d, *J* = 10.5 Hz, 1H), 4.83 (d, *J* = 10.1 Hz, 1H), 4.66 (q, *J* = 5.5 Hz, 1H), 4.60 (s, 1H), 4.49 (d, *J* = 33.1 Hz, 1H), 4.31 (q, *J* = 14.2 Hz, 2H), 4.17 (s, 1H), 4.00 – 3.95 (m, 1H), 3.83 (d, *J* = 3.2 Hz, 1H), 3.67 – 3.54 (m, 2H), 2.73 – 2.59 (m, 3H), 2.55 (p, *J* = 7.1 Hz, 1H), 2.50 – 2.40 (m, 1H), 2.37 – 2.25 (m, 1H), 1.76 – 1.70 (m, 6H), 1.61 (s, 3H), 1.21 – 1.15 (m, 1H), 1.15 – 1.10 (m, 9H), 1.06 (s, 3H), 1.04 (s, 3H), 0.82 (t, *J* = 7.4 Hz, 3H) ppm;

^{13}C NMR (126 MHz, acetonitrile-d_3) δ 177.5, 168.7, 167.2, 153.4, 144.1, 142.9, 137.2, 136.6, 135.8, 134.3, 131.7, 128.8, 127.2, 126.7, 125.8, 123.9, 122.4, 121.8, 119.5, 96.4, 93.1, 78.8, 75.6, 73.8, 73.1, 72.6, 70.0, 68.1, 42.3, 41.0, 37.4, 34.9, 28.6, 28.1, 26.5, 20.0, 19.4, 19.1, 18.6, 17.4, 15.4, 13.9, 11.3 ppm;

IR (thin film): ν 3676w, 3397w, 2973s, 2901m, 1692m, 1640w, 1599w, 1548m, 1455m, 1384m, 1252m, 1151m, 1067s, 1039s, 893w, 753w, 725w, 480w, 467w cm^{-1};

HRMS (APCI) for $C_{43}H_{61}O_{10}N_2S^+$ [M+H]$^+$: calculated: 797.40414; found: 797.40451.

ERJ-205

Analytical Data of 9i

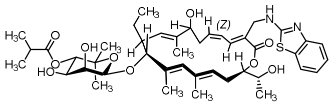

^{1}H NMR (500 MHz, acetonitrile-d_3) δ 7.61 (d, J = 7.8 Hz, 1H), 7.47 – 7.40 (m, 2H), 7.26 (t, J = 7.6 Hz, 1H), 7.06 (t, J = 7.5 Hz, 1H), 6.74 (t, J = 11.3 Hz, 1H), 6.40 (s, 1H, NH), 6.11 – 6.04 (m, 1H), 5.71 (s, 1H), 5.47 (t, J = 7.7 Hz, 1H), 5.09 (d, J = 10.6 Hz, 1H), 4.84 (d, J = 10.1 Hz, 1H), 4.80 – 4.74 (m, 1H), 4.63 (s, 1H), 4.41 – 4.34 (m, 2H), 4.01 – 3.94 (m, 2H), 3.85 (s, 1H), 3.64 (d, J = 9.6 Hz, 2H), 3.11 (s, 1H), 3.06 (s, 1H), 2.93 (s, 1H), 2.80 (s, 1H), 2.72 – 2.62 (m, 1H), 2.59 – 2.52 (m, 1H), 2.51 – 2.45 (m, 2H), 2.39 – 2.31 (m, 1H), 2.15 – 2.07 (m, 1H), 1.70 (s, 3H), 1.66 – 1.59 (m, 6H), 1.17 – 1.11 (m, 6H), 1.10 – 1.08 (m, 4H), 1.05 (s, 3H), 0.85 (t, J = 7.3 Hz, 3H) ppm;

^{13}C NMR (126 MHz, acetonitrile-d_3) δ 177.5, 168.0, 167.1, 153.5, 141.5, 139.7, 136.8, 136.4, 136.1, 133.1, 131.8, 128.4, 126.7, 125.5, 123.0, 122.4, 121.8, 119.5, 105.7, 96.6, 93.1, 78.9, 75.6, 74.0, 73.9, 72.4, 70.0, 67.7, 42.4, 40.9, 37.2, 34.9, 28.6, 28.4, 26.4, 20.2, 19.4, 19.1, 18.8, 17.8, 15.7, 13.8, 11.7 ppm;

IR (thin film): ν 3660w, 3383m, 2973m, 2929m, 1696m, 1635w, 1599m, 1548m, 1455m, 1407m, 1383m, 1314m, 1261m, 1228m, 1203m, 1148m, 1073s, 1037s, 879w, 795w, 753m, 725w, 698w, 625w, 513w cm^{-1};

HRMS (ESI) for $C_{43}H_{61}O_{10}N_2S^+$ $[M+H]^+$: calculated: 797.40414; found: 797.40365.

ERJ-205

Preparation of Aminothiazole 10

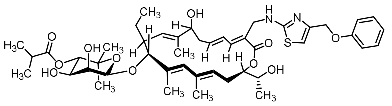

To an HPLC vial was added fidaxomicin (**1**, 100 mg, 94.5 µmol, 1.0 eq.), 4-(phenoxymethyl)thiazol-2-amine (58.8 mg, 0.284 mmol, 3.0 eq.), [Pd(cinnamyl)Cl]$_2$ (4.9 mg, 9.5 µmol, 10 mol%) and dppf (10.5 mg, 19 µmol, 20 mol%). The vial was capped, evacuated and backfilled with nitrogen three times and then dry THF (0.5 mL) was added. The reaction was heated under stirring to 60 °C. Reaction progress was monitored by UHPLC-MS. After 22 h the reaction mixture was cooled to RT, diluted with THF (1 mL), 3-mercaptopropyl-functionalised silica was added and the mixture was stirred at RT until no Pd-complexes were observed by UHPLC-MS. The mixture was filtered through a pad of celite, eluted with THF (2 mL) and concentrated *in vacuo*. The resulting residue was dissolved in MeCN (1 mL) and eluted through a C18 SPE cartridge with MeCN (8 mL). The filtrate was concentrated *in vacuo* and the resulting crude mixture purified by preparative RP-HPLC ([Gemini NX, C18, 5 µ, 110 Å, 250 mm × 21.2 mm, solvent A: H_2O + 0.1% HCOOH, solvent B: MeCN + 0.1% HCOOH, 20 mL/min; LC time program (min – %B): 7 min – 20%, 60 min – 40%]) to yield, after concentration *in vacuo*, the 2-aminothiazole **10** (t_R = 67.4 min, 20.9 mg, 24.5 µmol, 26%) as an off-white solid.

Analytical Data of Aminothiazole 10

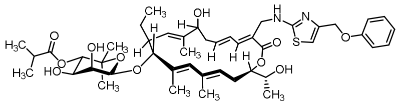

^{1}H NMR (500 MHz, acetone-d_6) δ 7.28 (dd, J = 8.7, 7.2 Hz, 2H), 7.17 (d, J = 11.4 Hz, 1H), 7.02 (d, J = 8.1 Hz, 2H), 6.93 (t, J = 7.3 Hz, 1H), 6.84 – 6.76 (m, 1H), 6.73 (t, J = 5.7 Hz, 1H), 6.53 (s, 1H), 5.91 (ddd, J = 14.6, 9.6, 4.5 Hz, 1H), 5.82 (s, 1H), 5.61 (t, J = 8.3 Hz, 1H), 5.20 (d, J = 10.5 Hz, 1H), 4.99 (d, J = 10.1 Hz, 1H), 4.95 (s, 2H), 4.76 (s, 1H), 4.71 (q, J = 5.3 Hz, 1H), 4.32 – 4.20 (m, 3H), 4.06 – 4.00 (m, 2H), 3.95 (d, J = 3.4 Hz, 1H), 3.84 (d, J = 3.7 Hz, 1H), 3.76 – 3.69 (m, 2H), 3.67 (d, J = 4.2 Hz, 1H), 3.28 (d, J = 9.3 Hz, 1H), 2.79 – 2.71 (m, 1H), 2.70 – 2.60 (m, 2H), 2.60 – 2.53 (m, 1H), 2.53 – 2.46 (m, 1H), 2.40 (ddd, J = 13.9, 9.0, 4.4 Hz, 1H), 1.94 (dtd, J = 15.0, 7.5, 3.1 Hz, 1H), 1.80 (s, 3H), 1.70 (s, 3H), 1.64 (s, 3H), 1.30 – 1.19 (m, 1H), 1.17 – 1.12 (m, 12H), 1.08 (s, 3H), 0.82 (t, J = 7.4 Hz, 3H) ppm;

^{13}C NMR (126 MHz, acetone-d_6) δ 176.8, 169.6, 168.4, 159.8, 149.3, 144.2, 142.5, 136.9, 136.2, 136.0, 133.9, 130.2, 128.8, 126.4, 125.9, 124.0, 121.5, 115.6, 104.4, 96.7, 93.3, 78.4, 75.7, 73.7, 72.9, 72.8, 70.2, 67.6, 67.0, 42.0, 41.1, 37.3, 34.8, 28.7, 28.2, 26.5, 20.6, 19.4, 19.2, 18.6, 17.4, 15.2, 13.8, 11.2 ppm;

IR (thin film): ν 3410w, 2975m, 2933m, 2875w, 1735m, 1693m, 1641w, 1599w, 1556m, 1496m, 1458m, 1385m, 1340m, 1299m, 1247s, 1213s, 1150m, 1079s, 1034s, 897w, 755m, 692m, 527w, 505w cm^{-1};

HRMS (ESI) for $C_{46}H_{65}O_{11}N_2S^+$ $[M+H]^+$: calculated: 853.43036; found: 853.42989.

ERJ-246

Preparation of Aminothiazole 11

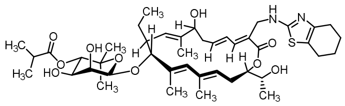

To an HPLC vial was added fidaxomicin (**1**, 100 mg, 94.5 µmol, 1.0 eq.), 4,5,6,7-tetrahydrobenzo[*d*]thiazol-2-amine (43.7 mg, 0.284 mmol, 3.0 eq.), $[Pd(cinnamyl)Cl]_2$ (4.9 mg, 9.5 µmol, 10 mol%) and dppf (10.5 mg, 18.9 µmol, 20 mol%). The vial was capped, evacuated and backfilled with nitrogen three times and then dry THF (0.5 mL) was added. The reaction was heated under stirring to 60 °C. Reaction progress was monitored by UHPLC-MS. After 3 h the reaction mixture was cooled to RT, 3-mercaptopropyl-functionalised silica was added and the mixture was stirred at RT until no Pd-complexes were observed by UHPLC-MS. The reaction mixture was diluted with MeCN (2 mL) and eluted through a C18 SPE cartridge with MeCN (8 mL). The filtrate was concentrated *in vacuo* and the crude mixture purified by preparative RP-HPLC ([Gemini NX, C18, 5 µ, 110 Å, 250 mm × 21.2 mm, solvent A: H_2O + 0.1% HCOOH, solvent B: MeCN + 0.1% HCOOH, 20 mL/min; LC time program (min – %B): 7 min – 15%, 60 min – 30%]) to yield, after concentration *in vacuo*, the 2-aminothiazole **11** (t_R = 37.7 min, 29.3 mg, 36.6 µmol, 39%) as an off-white solid.

Analytical Data of Aminothiazole 11

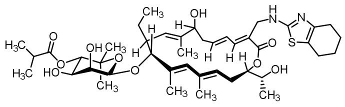

^{1}H NMR (500 MHz, acetone-d_6) δ 7.14 (d, J = 11.4 Hz, 1H), 6.75 (ddd, J = 13.7, 11.5, 1.7 Hz, 1H), 6.43 (br. s, 1H, NH), 5.90 (ddd, J = 14.7, 9.6, 4.6 Hz, 1H), 5.83 (s, 1H), 5.64 – 5.56 (m, 1H), 5.20 (dt, J = 10.7, 1.6 Hz, 1H), 5.00 (s, 1H), 4.76 (d, J = 1.3 Hz, 1H), 4.73 – 4.66 (m, 1H), 4.29 – 4.23 (m, 1H), 4.24 – 4.13 (m, 2H), 4.03 (p, J = 6.3 Hz, 1H), 3.95 (dd, J = 3.3, 1.3 Hz, 1H), 3.76 – 3.69 (m, 2H), 2.75 (dt, J = 13.9, 6.8 Hz, 1H), 2.72 – 2.60 (m, 2H), 2.60 – 2.43 (m, 6H), 2.40 (ddd, J = 13.8, 8.8, 4.3 Hz, 1H), 1.95 (dddd, J = 15.1, 13.1, 7.4, 3.1 Hz, 1H), 1.79 (d, J = 1.3 Hz, 3H), 1.76 (p, J = 3.0 Hz, 4H), 1.72 (d, J = 1.3 Hz, 3H), 1.65 (d, J = 1.3 Hz, 3H), 1.28 – 1.20 (m, 1H), 1.17 – 1.11 (m, 12H), 1.08 (s, 3H), 0.83 (t, J = 7.4 Hz, 3H) ppm;

^{13}C NMR (126 MHz, acetone-d_6) δ 176.8, 168.4, 166.6, 146.0, 143.9, 142.3, 136.8, 136.2, 136.0, 133.9, 128.8, 126.6, 126.1, 124.0, 115.9, 96.7, 93.3, 78.4, 75.7, 73.7, 72.9, 72.8, 70.1, 67.7, 42.1, 41.0, 37.3, 34.8, 28.7, 28.2, 27.4, 26.5, 24.4, 23.8, 23.6, 20.6, 19.4, 19.2, 18.6, 17.4, 15.2, 13.8, 11.2 ppm;

IR (thin film): ν 2974s, 2934s, 2874m, 1736m, 1694s, 1641m, 1539m, 1446m, 1385m, 1368m, 1298m, 1255s, 1202s, 1151s, 1074s, 1033s, 898w, 797w, 516w cm^{-1};

HRMS (ESI) for $C_{43}H_{65}O_{10}N_2S^+$ $[M+H]^+$: calculated: 801.43544; found: 801.43469.

ERJ-237, ERJ-322

Preparation of Aminothiazole 12

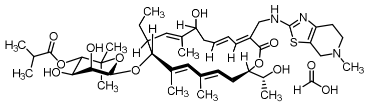

To an HPLC vial was added fidaxomicin (**1**, 50.0 mg, 47.3 µmol, 1.0 eq.), 5-methyl-4,5,6,7-tetrahydrothiazolo[5,4-*c*]pyridin-2-amine (24.0 mg, 0.142 mmol, 3.0 eq.), $[Pd(allyl)Cl]_2$ (8.7 mg, 23.7 µmol, 50 mol%) and dppf (26.2 mg, 47.3 µmol, 100 mol%). The vial was capped, evacuated and backfilled with nitrogen three times and then dry MeOH (0.25 mL) was added. The reaction was heated under stirring to 60 °C. Reaction progress was monitored by UHPLC-MS. After 16 h the reaction mixture was cooled to RT, diluted with MeCN (2 mL) and eluted through a C18 SPE cartridge with MeCN (8 mL). The filtrate was concentrated *in vacuo* and the crude mixture purified by preparative RP-HPLC ([Gemini NX, C18, 5 µ, 110 Å, 250 mm × 21.2 mm, solvent A: H_2O + 0.1% HCOOH, solvent B: MeCN + 0.1% HCOOH, 20 mL/min; LC time program (min – %B): 5 min – 2%, 7 min – 10%, 50 min – 16%]) to yield, after concentration *in vacuo*, the formate salt of the 2-aminothiazole **12** (t_R = 36.1 min, 12.8 mg, 14.9 µmol, 31%) as a yellow-brown solid.

Analytical Data of Aminothiazole 12

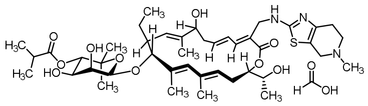

^{1}H NMR (500 MHz, acetone-*d₆*) δ 8.16 (s, 1H), 7.14 (d, *J* = 11.4 Hz, 1H), 6.79 – 6.69 (m, 1H), 6.57 (s, 1H, NH), 5.89 (td, *J* = 9.9, 4.9 Hz, 1H), 5.82 (s, 1H), 5.60 (t, *J* = 8.3 Hz, 1H), 5.19 (dt, *J* = 10.5, 1.6 Hz, 1H), 4.99 (d, *J* = 10.1 Hz, 1H), 4.78 – 4.75 (m, 1H), 4.69 (td, *J* = 5.9, 4.3 Hz, 1H), 4.25 (s, 1H), 4.25 – 4.15 (m, 2H), 4.02 (p, *J* = 6.3 Hz, 1H), 3.97 – 3.94 (m, 1H), 3.74 – 3.69 (m, 2H), 3.54 – 3.51 (m, 4H), 2.84 – 2.80 (m, 2H), 2.78 – 2.72 (m, 1H), 2.69 – 2.60 (m, 2H), 2.56 (p, *J* = 7.0 Hz, 1H), 2.50 – 2.45 (m, 4H), 2.43 – 2.36 (m, 1H), 1.98 – 1.91 (m, 1H), 1.81 – 1.78 (m, 3H), 1.72 – 1.70 (m, 3H), 1.66 – 1.63 (m, 4H), 1.27 – 1.21 (m, 1H), 1.18 – 1.13 (m, 15H), 1.08 (s, 3H), 0.83 (t, *J* = 7.4 Hz, 3H) ppm;

^{13}C NMR (126 MHz, acetone-*d₆*) δ 176.8, 168.4, 167.6, 163.2, 144.4, 144.1, 142.4, 136.8, 136.2, 136.0, 133.9, 128.8, 126.5, 126.0, 124.0, 112.3, 96.7, 93.3, 78.4, 75.7, 73.7, 72.9, 72.7, 70.1, 67.6, 52.9, 52.2, 44.7, 42.1, 41.0, 37.3, 34.8, 28.7, 28.2, 27.1, 26.5, 20.6, 19.4, 19.2, 18.6, 17.4, 15.2, 13.8, 11.2 ppm;

IR (thin film): ν 3676m, 3369w, 2988s, 2901s, 1693w, 1597w, 1406m, 1394m, 1382m, 1250m, 1076s, 1066s, 1057s, 893m, 600w cm^{-1};

HRMS (ESI) for $C_{43}H_{66}O_{10}N_3S^+$ [M+H]$^+$: calculated: 816.44634; found: 816.44682.

ERJ-194

Preparation of
5-Methyl-4,5,6,7-tetrahydrothiazolo[5,4-*c*]pyridin-2-amine dihydrobromide (12a)

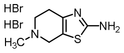

Prepared according to a literature procedure.[8] To a stirred solution of 1-methyl-4-piperidinone (1.16 mL, 10.0 mmol, 1.0 eq.) in hydrobromic acid (33% in AcOH, 12 mL), at 0 °C was added bromine (0.514 mL, 10.0 mmol, 1.0 eq.). The reaction mixture was allowed to warm to RT and was stirred for 20 min. Thiourea (761 mg, 10.0 mmol, 1.0 eq.) was added and the reaction mixture was stirred at RT for 30 min and then heated to 90 °C for 1.5 h. The reaction mixture was cooled to RT and concentrated *in vacuo*. The resulting residue was co-evaporated with EtOH (50 mL). The resulting crude mixture was recrystallised from EtOH/water to yield **12a** (385 mg, 1.16 mmol, 12%) as a colourless solid.

The freebase was obtained as described in **GP2**.

Analytical Data of
5-Methyl-4,5,6,7-tetrahydrothiazolo[5,4-*c*]pyridin-2-amine dihydrobromide (12a)

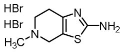

^{1}H NMR (400 MHz, DMSO-d_6) δ 6.67 (s, 2H), 3.33 (br. s, 2H), 2.63 (t, J = 5.8 Hz, 2H), 2.45 – 2.40 (m, 2H), 2.32 (s, 3H) ppm.

Analytical data are in agreement with the literature.[9]

ERJ-107

Preparation of Aminothiazole 13

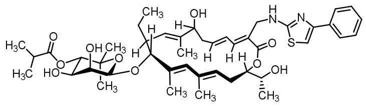

To an HPLC vial was added fidaxomicin (**1**, 80 mg, 75.6 µmol, 1.0 eq.), 2-amino-4-phenylthiazole (40.0 mg, 0.227 mmol, 3.0 eq.), $[Pd(cinnamyl)Cl]_2$ (3.9 mg, 7.6 µmol, 10 mol%) and dppf (8.4 mg, 15 µmol, 20 mol%). The vial was capped, evacuated and backfilled with nitrogen three times and then dry THF (0.4 mL) was added. The reaction was heated under stirring to 60 °C. Reaction progress was monitored by UHPLC-MS. After 5 h the reaction mixture was cooled to RT, 3-mercaptopropyl-functionalised silica was added and the mixture was stirred at RT until no Pd-complexes were observed by UHPLC-MS. The reaction mixture was diluted with MeCN (2 mL) and eluted through a C18 SPE cartridge with MeCN (8 mL). The filtrate was concentrated *in vacuo* and the crude mixture purified by preparative RP-HPLC ([Gemini NX, C18, 5 µ, 110 Å, 250 mm × 21.2 mm, solvent A: H_2O + 0.1% HCOOH, solvent B: MeCN + 0.1% HCOOH, 20 mL/min; LC time program (min – %B): 7 min – 30%, 60 min – 45%]) to yield, after concentration *in vacuo*, the 2-aminothiazole **13** (t_R = 52.5 min, 21.2 mg, 25.8 µmol, 34%) as an off-white solid.

Analytical Data of Aminothiazole 13

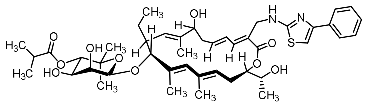

^{1}H NMR (500 MHz, acetone-*d6*) δ 7.94 – 7.91 (m, 2H), 7.37 (t, *J* = 7.7 Hz, 2H), 7.28 – 7.23 (m, 1H), 7.18 (d, *J* = 11.4 Hz, 1H), 6.99 – 6.91 (m, 2H), 6.86 (t, *J* = 5.8 Hz, 1H), 5.93 (ddd, *J* = 14.6, 9.5, 4.6 Hz, 1H), 5.82 (s, 1H), 5.61 (d, *J* = 3.9 Hz, 2H), 5.21 (dt, *J* = 10.7, 1.6 Hz, 1H), 4.99 (d, *J* = 10.2 Hz, 1H), 4.76 (d, *J* = 1.2 Hz, 1H), 4.72 (td, *J* = 5.9, 4.3 Hz, 1H), 4.45 (dd, *J* = 14.0, 5.7 Hz, 1H), 4.32 – 4.25 (m, 2H), 4.02 (p, *J* = 6.4 Hz, 1H), 3.95 (dd, *J* = 3.3, 1.2 Hz, 1H), 3.75 – 3.69 (m, 2H), 2.80 – 2.70 (m, 2H), 2.68 – 2.52 (m, 3H), 2.41 (ddd, *J* = 13.7, 8.9, 4.3 Hz, 1H), 1.98 – 1.91 (m, 1H), 1.79 (d, *J* = 1.4 Hz, 3H), 1.73 (d, *J* = 1.4 Hz, 3H), 1.65 (d, *J* = 1.3 Hz, 3H), 1.28 – 1.20 (m, 1H), 1.17 – 1.11 (m, 13H), 1.08 (s, 3H), 0.82 (t, *J* = 7.4 Hz, 3H) ppm;

^{13}C NMR (126 MHz, acetone-*d6*) δ 176.8, 168.9, 168.5, 151.6, 143.9, 142.2, 136.8, 136.3, 136.2, 136.0, 133.9, 129.2, 129.0, 128.0, 126.8, 126.6, 126.3, 124.0, 101.7, 96.7, 93.3, 78.4, 75.7, 73.7, 72.9, 72.8, 70.1, 67.7, 42.1, 40.9, 37.3, 34.8, 28.7, 28.2, 26.5, 20.6, 19.4, 19.2, 18.6, 17.4, 15.2, 13.8, 11.2 ppm;

IR (thin film): ν 3386m, 2974m, 2933m, 2874m, 1733m, 1691m, 1640m, 1602w, 1547m, 1524w, 1469w, 1444m, 1385m, 1369m, 1333m, 1299m, 1251m, 1207m, 1149s, 1072s, 1033s, 1008m, 950w, 898w, 772w, 703m cm^{-1};

HRMS (ESI) for $C_{45}H_{63}O_{10}N_2S^+$ $[M+H]^+$: calculated: 823.41979; found: 823.42023.

ERJ-238 ERJ-318b

1.3.4 Primary Amines

Preparation of Thiazole 14

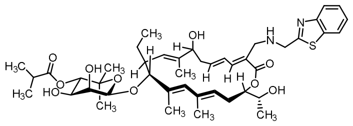

Prepared according to **GP1** at 94.5 µmol scale using benzo[*d*]thiazol-2-ylmethanamine (5.0 eq.) as the nucleophile. The residue was purified by preparative RP-HPLC ([Gemini NX, C18, 5 µ, 110 Å, 250 mm × 21.2 mm, solvent A: H_2O + 0.1% HCOOH, solvent B: MeCN + 0.1% HCOOH, 20 mL/min; LC time program (min – %B): 7 min – 20%, 60 min – 40%]) to yield, after concentration *in vacuo*, the thiazole **14** (t_R = 31.5 min, 35.0 mg, 43.2 µmol, 46%) as an off-white solid.

Analytical Data of Thiazole 14

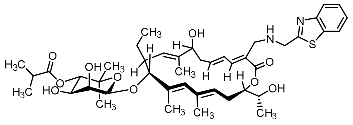

^{1}H NMR (500 MHz, $CDCl_3$) δ 8.00 – 7.89 (m, 2H), 7.51 – 7.36 (m, 2H), 7.19 (d, *J* = 11.5 Hz, 1H), 6.20 (dd, *J* = 14.8, 11.7 Hz, 1H), 5.89 (s, 1H), 5.81 (ddd, *J* = 15.0, 11.0, 4.0 Hz, 1H), 5.46 (dd, *J* = 10.5, 5.8 Hz, 1H), 5.15 (d, *J* = 10.7 Hz, 1H), 5.00 (d, *J* = 10.0 Hz, 1H), 4.68 (d, *J* = 15.5 Hz, 2H), 4.42 – 4.39 (m, 1H), 4.15 – 4.10 (m, 2H), 4.10 – 4.05 (m, 1H), 4.02 (d, *J* = 3.3 Hz, 1H), 3.69 – 3.65 (m, 2H), 3.63 – 3.59 (m, 1H), 3.54 – 3.46 (m, 1H), 2.85 – 2.76 (m, 1H), 2.75 – 2.67 (m, 1H), 2.66 – 2.57 (m, 2H), 2.33 (ddd, *J* = 15.0, 11.3, 4.9 Hz, 1H), 2.28 – 2.21 (m, 1H), 1.97 (s, 3H), 1.91 (ddd, *J* = 13.4, 7.5, 2.8 Hz, 1H), 1.83 (s, 3H), 1.68 (s, 3H), 1.27 – 1.22 (m, 1H), 1.23 – 1.17 (m, 9H), 1.15 (s, 3H), 1.10 (s, 3H), 0.86 (t, *J* = 7.4 Hz, 3H) ppm;

^{13}C NMR (126 MHz, $CDCl_3$) δ 177.3, 175.1, 170.0, 153.1, 143.5, 139.9, 136.5, 136.0, 135.3, 134.4, 134.4, 129.6, 128.3, 126.2, 125.3, 123.2, 122.5, 122.1, 94.7, 92.9, 79.8, 74.9, 73.4, 72.7, 71.6, 70.2, 69.0, 49.2, 43.4, 42.0, 37.6, 34.3, 28.5, 26.0, 19.2, 18.9, 18.5, 18.2, 17.0, 15.3, 13.8, 11.2 ppm;

IR (thin film): ν 3404w, 2975w, 2932w, 2874w, 1734m, 1690m, 1641w, 1456w, 1437m, 1384m, 1369m, 1346m, 1312m, 1252m, 1202m, 1148s, 1077s, 1032s, 977m, 949w, 897m, 842w, 797m, 761m, 732s, 701m, 584w, 505m, 463w, 431m cm^{-1};

HRMS (ESI) for $C_{44}H_{63}O_{10}N_2S^+$ $[M+H]^+$: calculated: 8811.41979; found: 8811.41931.

ERJ-449

Preparation of Oxadiazole 15

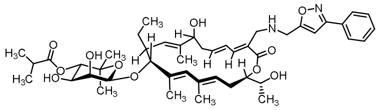

Prepared according to **GP1** At 94.5 µmol scale using (3-phenylisoxazol-5-yl)methanamine (5.0 eq.) as the nucleophile. The residue was purified by preparative RP-HPLC ([Gemini NX, C18, 5 µ, 110 Å, 250 mm × 21.2 mm, solvent A: H_2O + 0.1% HCOOH, solvent B: MeCN + 0.1% HCOOH, 20 mL/min; LC time program (min – %B): 27% isocratic]) to yield, after concentration *in vacuo*, the oxadiazole **15** (t_R = 18.1 min, 8.2 mg, 10 µmol, 11%) as an off-white solid.

Analytical Data of Oxadiazole 15

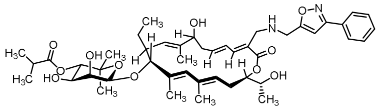

^{1}H NMR (500 MHz, $CDCl_3$) δ 7.83 – 7.73 (m, 2H), 7.51 – 7.40 (m, 3H), 6.97 (d, *J* = 11.2 Hz, 1H), 6.53 (s, 1H), 6.37 (t, *J* = 13.2 Hz, 1H), 5.76 (s, 1H), 5.74 – 5.69 (m, 1H), 5.42 – 5.37 (m, 1H), 5.02 – 4.94 (m, 2H), 4.66 (d, *J* = 9.9 Hz, 1H), 4.60 (s, 1H), 4.24 (s, 1H), 4.21 – 4.16 (m, 1H), 4.05 – 3.93 (m, 3H), 3.71 – 3.63 (m, 2H), 3.60 – 3.51 (m, 2H), 2.81 (dt, *J* = 14.5, 10.2 Hz, 1H), 2.72 – 2.55 (m, 3H), 2.50 – 2.40 (m, 1H), 2.24 – 2.18 (m, 1H), 1.91 – 1.85 (m, 1H), 1.83 (s, 3H), 1.75 (s, 3H), 1.65 (s, 3H), 1.26 – 1.23 (m, 1H), 1.22 – 1.16 (m, 9H), 1.13 (s, 3H), 1.07 (s, 3H), 0.82 (t, *J* = 7.3 Hz, 3H) ppm;

^{13}C NMR (126 MHz, $CDCl_3$) δ 177.3, 171.3, 169.1, 162.5, 142.7, 140.1, 136.6, 136.3, 134.4, 134.2, 130.3, 129.1, 129.0, 129.0, 128.2, 127.0, 126.1, 123.2, 100.6, 94.6, 92.5, 79.3, 74.8, 73.4, 72.7, 71.6, 70.2, 68.4, 44.4, 43.9, 41.9, 37.1, 34.3, 28.1, 27.5, 25.8, 19.2, 18.9, 18.5, 18.4, 16.9, 15.4, 13.7, 11.1 ppm;

IR (thin film): ν 3413w, 2976w, 2930w, 2875w, 1732m, 1688m, 1641m, 1608w, 1580w, 1469m, 1443m, 1407m, 1384m, 1369m, 1349m, 1299m, 1250m, 1202m, 1147m, 1074m, 1031s, 996m, 979m, 950m, 933m, 900m, 841m, 812m, 797m, 770m, 733s, 695s, 646m, 584m, 505m, 462m, 441m, 425m, 416m, 402m cm^{-1};

HRMS (ESI) for $C_{46}H_{65}O_{10}N_2^+$ $[M+H]^+$: calculated: 821.45829; found: 821.45797.

ERJ-541

Preparation of Oxadiazole 16

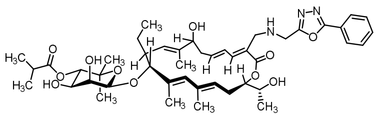

Prepared according to **GP1** at 94.5 µmol scale using (5-phenyl-1,3,4-oxadiazol-2-yl)methanamine (5.0 eq.) as the nucleophile. The residue was purified by preparative RP-HPLC ([Gemini NX, C18, 5 µ, 110 Å, 250 mm × 21.2 mm, solvent A: H_2O + 0.1% HCOOH, solvent B: MeCN + 0.1% HCOOH, 20 mL/min; LC time program (min – %B): 7 min – 15%, 60 min – 25%]) to yield, after concentration *in vacuo*, the oxadiazole **16** (t_R = 34.8 min, 14.2 mg, 17.0 µmol, 18%) as an off-white solid.

Analytical Data of Oxadiazole 16

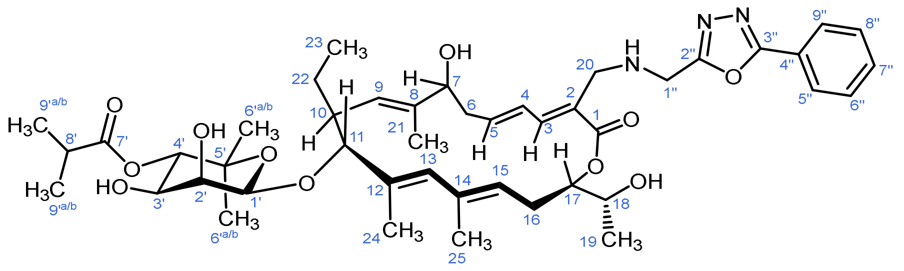

^{1}H NMR (500 MHz, acetone-d_6) δ 8.08 – 8.02 (m, 2H, arom. H), 7.65 – 7.57 (m, 3H, arom. H), 6.99 (d, J = 11.5 Hz, 1H, H3), 6.59 (dddd, J = 14.8, 11.6, 2.0, 0.9 Hz, 1H, H4), 5.82 (dt, J = 9.7, 4.7 Hz, 1H, H5), 5.78 (d, J = 2.9 Hz, 1H, H13), 5.43 (t, J = 8.3 Hz, 1H, H15), 5.19 (dt, J = 10.7, 1.5 Hz, 1H, H9), 4.98 (d, J = 10.1 Hz, 1H, H4'), 4.74 (d, J = 1.3 Hz, 1H, H1'), 4.62 (ddd, J = 7.0, 5.4, 4.2 Hz, 1H, H17), 4.22 (s, 1H, H7), 4.12 – 4.05 (m, 1H, H18), 4.04 (s, 2H, H1''), 3.95 (dd, J = 3.4, 1.3 Hz, 1H, H2'), 3.77 – 3.70 (m, 2H, H3'/H11), 3.64, 3.58 (AB_q, 2H, J_{AB} = 13.4 Hz, H20) 2.81 – 2.72 (m, 1H, H16b), 2.67 – 2.60 (m, 2H, H10/H6a), 2.56 (sept, J = 7.0 Hz, 1H, H8'), 2.50 – 2.41 (m, 1H, H6b), 2.29 (ddd, J = 14.0, 8.1, 4.2 Hz, 1H, H16a), 1.98 – 1.90 (m, 1H, H22a), 1.75 (d, J = 1.4 Hz, 3H, CH_3), 1.70 (d, J = 1.4 Hz, 3H, CH_3), 1.64 (t, J = 0.9 Hz, 3H, CH_3), 1.27 – 1.20 (m, 1H, H22b), 1.16 – 1.12 (m, 9H, H19/H6'^{a}/H6'^{b}), 1.11 (s, 3H, H9'$^{a/b}$), 1.06 (s, 3H, H9'$^{a/b}$), 0.82 (t, J = 7.5 Hz, 3H, H23) ppm;

^{13}C NMR (126 MHz, acetone-d_6) δ 176.8, 168.8, 167.1, 165.2, 143.1, 141.8, 136.9, 136.4, 135.7, 134.1, 132.6, 130.1, 128.6, 127.6, 127.5, 127.2, 125.1, 124.1, 96.6, 93.3, 78.3, 75.7, 73.1, 73.0, 72.7, 70.1, 67.7, 67.6, 44.9, 43.4, 42.2, 37.4, 34.8, 28.7, 27.6, 26.4, 20.2, 19.4, 19.2, 18.6, 17.3, 13.8, 11.2 ppm;

IR (thin film): ν 3432m, 2973m, 2927s, 2873m, 1737m, 1694s, 1641m, 1609w, 1554w, 1451m, 1384m, 1369m, 1295m, 1249m, 1207m, 1149m, 1071s, 1033s, 897m, 843w, 797w, 777w, 712m, 692m, 585w, 499w cm^{-1};

HRMS (ESI) for $C_{45}H_{64}O_{11}N_3^+$ $[M+H]^+$: calculated: 822.45354; found: 822.45412.

ERJ-301

Preparation of Oxadiazole 17

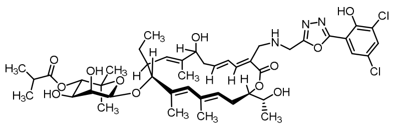

Prepared according to **GP1** At 30.2 µmol scale using 2-(5-(aminomethyl)-1,3,4-oxadiazol-2-yl)-4,6-dichlorophenol (4.8 eq.) as the nucleophile. The residue was purified by preparative RP-HPLC ([Gemini NX, C18, 5 µ, 110 Å, 250 mm × 21.2 mm, solvent A: H_2O + 0.1% HCOOH, solvent B: MeCN + 0.1% HCOOH, 20 mL/min; LC time program (min – %B): 7 min – 25%, 60 min – 45%]) to yield, after concentration *in vacuo*, the oxadiazole **17** (t_R = 43.2 min, 4.4 mg, 4.6 µmol, 15%) as an off-white solid.

Analytical Data of Oxadiazole 17

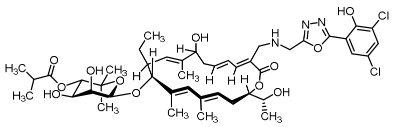

^{1}H NMR (500 MHz, $CDCl_3$) δ 7.66 (d, *J* = 2.5 Hz, 1H), 7.57 (d, *J* = 2.5 Hz, 1H), 6.82 (d, *J* = 11.5 Hz, 1H), 6.53 – 6.44 (m, 1H), 5.70 (ddd, *J* = 15.1, 10.9, 4.3 Hz, 1H), 5.65 (s, 1H), 5.05 – 4.95 (m, 2H), 4.91 (dd, *J* = 10.6, 5.7 Hz, 1H), 4.65 (s, 1H), 4.57 – 4.49 (m, 1H), 4.29 – 4.23 (m, 1H), 4.18 – 4.08 (m, 3H), 4.06 – 4.03 (m, 1H), 3.76 – 3.64 (m, 2H), 3.63 (s, 2H), 2.79 – 2.65 (m, 3H), 2.65 – 2.57 (m, 1H), 2.55 – 2.46 (m, 1H), 2.13 – 2.05 (m, 1H), 1.96 – 1.88 (m, 1H), 1.85 (s, 3H), 1.75 (s, 3H), 1.67 (s, 3H), 1.25 – 1.21 (m, 1H), 1.20 – 1.16 (m, 10H), 1.11 (s, 3H), 1.04 (s, 3H), 0.85 (t, *J* = 7.3 Hz, 3H) ppm;

^{13}C NMR (126 MHz, $CDCl_3$) δ 177.3, 169.4, 165.6, 163.0, 152.2, 142.5, 140.5, 136.7, 136.6, 134.6, 134.2, 133.7, 129.2, 128.1, 126.3, 125.1, 124.9, 123.1, 110.1, 94.5, 92.6, 79.6, 77.4, 74.9, 73.3, 73.1, 71.7, 70.2, 68.6, 44.9, 42.8, 41.9, 37.3, 34.3, 29.9, 28.1, 27.6, 19.2, 18.9, 18.4, 18.2, 16.8, 15.6, 13.7, 11.1 ppm;

IR (thin film): ν 3428w, 2974m, 2927m, 2874w, 2856w, 1735m, 1691m, 1641w, 1542w, 1468m, 1431m, 1385m, 1369m, 1296m, 1246s, 1192m, 1149m, 1078m, 1034s, 995m, 950w, 897m, 872m, 799m, 757m, 731m, 703w, 677w, 563w, 524w, 504w, 465w, 403w cm^{-1};

HRMS (ESI) for $C_{45}H_{62}O_{12}N_3Cl_2^+$ $[M+H]^+$: calculated: 906.37051; found: 906.37111.

ERJ-511

Preparation of Allyl 2-(allyloxy)-3,5-dichlorobenzoate (17a)

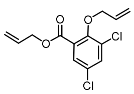

Carried out according to a modified literature procedure.[10] A suspension of 3,5-dichloro-2-hydroxybenzoic acid (1.00 g, 6.49 mmol, 1.0 eq.) and potassium carbonate (1.79 g, 13.0 mmol, 2.0 eq.) in dry acetone (16 mL) was heated in a microwave vial to 60 °C. Then, allyl bromide (1.13 mL, 13.0 mmol, 2.0 eq.) was added. The reaction was heated to 60 °C for 18 h. Then, the solution was cooled down to rt and diluted with water (10 mL) and extracted with EtOAc (mL). The combined organic layers were washed with brine, dried over Na_2SO_4, filtered, and concentrated *in vacuo*. This yielded the allyl 2-(allyloxy)-3,5-dichlorobenzoate (**17a**) (1.65 g, 5.74 mmol, 89%) as a brown oil.

Analytical Data of Allyl 2-(allyloxy)-3,5-dichlorobenzoate (17a)

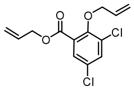

^{1}H NMR (500 MHz, $CDCl_3$) δ 7.71 – 7.67 (m, 1H), 7.56 – 7.52 (m, 1H), 6.11 (ddt, *J* = 16.6, 10.2, 5.8 Hz, 1H), 6.01 (ddt, *J* = 16.6, 10.3, 5.9 Hz, 1H), 5.46 – 5.36 (m, 2H), 5.35 – 5.25 (m, 2H), 4.84 – 4.78 (m, 2H), 4.58 – 4.53 (m, 2H) ppm;

^{13}C NMR (126 MHz, $CDCl_3$) δ 164.0, 153.5, 133.7, 133.0, 131.7, 130.9, 129.8, 129.4, 128.2, 119.4, 118.9, 75.7, 66.5 ppm;

IR (thin film): ν 3082w, 2957w, 1735m, 1649w, 1561w, 1441m, 1419m, 1406m, 1376w, 1357m, 1276s, 1231s, 1196m, 1135m, 1087m, 978s, 930m, 873m, 790m, 773m, 733w, 708w, 572w cm^{-1};

HRMS (ESI) for $C_{13}H_{12}O_3Cl_2Na^+$ $[M+Na]^+$: calculated: 309.00557; found: 309.00523.

AM-85

Preparation of 2-(Allyloxy)-3,5-dichlorobenzoic acid (17b)

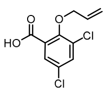

A solution of 2-(allyloxy)-3,5-dichlorobenzoate (1.6 g, 5.6 mmol) in THF (8.4 mL) and sodium hydroxide solution (2 M aq., 8.4 mL, 16.7 mmol, 3.0 eq.) was stirred for 12 h at 60 °C. The reaction was diluted with hydrochloric acid solution (1 M aq.) and extracted with EtOAc. The combined organic layers were washed with brine, dried over Na_2SO_4, filtered, and concentrated *in vacuo* to yield 2-(allyloxy)-3,5-dichlorobenzoic acid (**17b**) (0.78 g, 3.2 mmol, 57%) as a light-yellow solid.

Analytical Data of 2-(Allyloxy)-3,5-dichlorobenzoic acid (17b)

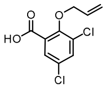

m.p.: 112 °C

^{1}H NMR (500 MHz, $CDCl_3$) δ 7.99 – 7.95 (m, 1H), 7.64 – 7.60 (m, 1H), 6.12 (ddt, *J* = 16.8, 10.2, 6.3 Hz, 1H), 5.46 (dt, *J* = 17.2, 1.4 Hz, 1H), 5.39 (d, *J* = 10.3 Hz, 1H), 4.69 (d, *J* = 6.3 Hz, 2H) ppm;

^{13}C NMR (126 MHz, $CDCl_3$) δ 165.2, 152.9, 135.3, 131.4, 131.1, 130.7, 129.9, 125.9, 121.6, 76.6 ppm;

IR (thin film): ν 2944m, 1695s, 1586w, 1557w, 1444m, 1418m, 1364m, 1295s, 1237s, 1155w, 1070w, 981m, 930m, 875m, 844w, 798m, 763w, 704m, 638w, 572w cm^{-1};

HRMS (ESI) for $C_{10}H_8O_3Cl_2Na^+$ $[M+Na]^+$: calculated: 268.97427; found: 268.97453.

AM-91

Preparation of *tert*-Butyl (2-(2-(2-(allyloxy)-3,5-dichlorobenzoyl)hydrazineyl)-2-oxoethyl)carbamate (17c)

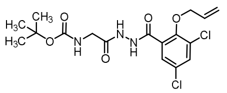

2-(Allyloxy)-3,4-dichlorobenzoic acid (0.50 g, 2.0 mmol, 1.0 eq.) and CDI (0.40 g, 2.5 mmol, 1.2 eq.) were added to acetonitrile (8 mL) and stirred for 30 min at RT. Afterwards *tert*-butyl-(2-hydrazineyl-2-oxoethyl)carbamate (0.38 g, 2.0 mmol, 1.0 eq.) was added. The solution was stirred for 12 h at rt. The solution was diluted with a hydrochloric acid solution (1 M aq., 10 mL) and extracted with EtOAc (3 × 10 mL). The combined organic layers were washed with water (5 mL), and brine (5 mL), dried over Na_2SO_4, filtered, and concentrated *in vacuo*. The crude was purified *via* column-chromatography (silica gel pentane/EtOAc 1:2) to yield *tert*-butyl (2-(2-(2-(allyloxy)-3,5-dichlorobenzoyl)hydrazineyl)-2-oxoethyl)carbamate (**17c**) as a white powder (0.50 g, 1.2 mmol, 59 %).

Analytical Data of *tert*-Butyl (2-(2-(2-(allyloxy)-3,5-dichlorobenzoyl)hydrazineyl)-2-oxoethyl)carbamate (17c)

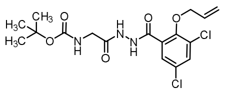

m.p.: 118 °C

^{1}H NMR (500 MHz, $CDCl_3$) δ 10.26 – 10.16 (m, 1H), 9.43 – 9.38 (m, 1H), 7.95 – 7.91 (m, 1H), 7.56 – 7.52 (m, 1H), 6.24 – 6.13 (m, 1H), 5.45 (dd, *J* = 16.9, 1.7 Hz, 1H), 5.35 (t, *J* = 10.6 Hz, 2H), 4.67 (d, *J* = 6.3 Hz, 2H), 3.99 (d, *J* = 6.0 Hz, 2H), 1.46 (s, 9H) ppm;

^{13}C NMR (126 MHz, $CDCl_3$) δ 166.9, 160.4, 156.4, 151.6, 134.0, 131.7, 130.7, 130.1, 129.8, 127.2, 121.4, 80.8, 76.2, 43.2, 28.4 ppm;

IR (thin film): ν 3082w, 2957w, 1735m, 1649w, 1561w, 1441m, 1419m, 1406m, 1376m, 1357m, 1276s, 1231s, 1196m, 1135m, 1087m, 978s, 930m, 873m, 790m, 773m, 733m, 708w, 572m cm^{-1};

HRMS (ESI) for $C_{17}H_{22}O_5N_3Cl_2^+$ $[M+H]^+$: calculated: 418.09310; found: 418.09268.

AM-96

Preparation of *tert*-Butyl ((5-(2-(allyloxy)-3,5-dichlorophenyl)-1,3,4-oxadiazol-2-yl)methyl)carbamate (17d)

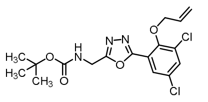

The cyclisation of **17c** was carried out according to **GP5**. The resulting residue was purified *via* flash column chromatography (silica gel, pentane/EtOAc 1:1) to yield *tert*-Butyl ((5-(2-(allyloxy)-3,5-dichlorophenyl)-1,3,4-oxadiazol-2-yl)methyl)carbamate (**17d**) (0.30 g, 0.75 mmol, 90%) as a colorless solid.

Analytical Data of *tert*-Butyl ((5-(2-(allyloxy)-3,5-dichlorophenyl)-1,3,4-oxadiazol-2-yl)methyl)carbamate (17d)

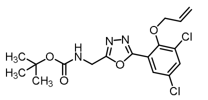

m.p.: 73 °C

^{1}H NMR (500 MHz, $CDCl_3$) δ 7.87 – 7.83 (m, 1H), 7.58 – 7.54 (m, 1H), 6.10 (ddt, J = 16.6, 10.4, 6.0 Hz, 1H), 5.44 – 5.36 (m, 1H), 5.31 – 5.25 (m, 1H), 5.22 (br. s, 1H), 4.66 – 4.62 (m, 2H), 4.59 (d, J = 6.0 Hz, 2H), 1.46 (s, 9H) ppm;

^{13}C NMR (126 MHz, $CDCl_3$) δ 164.6, 162.1, 155.5, 152.1, 133.5, 132.7, 131.2, 130.2, 128.4, 121.1, 119.4, 80.9, 75.5, 36.1, 28.4 ppm;

IR (thin film): ν 3345w, 3075w, 2979m, 2934w, 1716s, 1700s, 1648w, 1564m, 1513m, 1466m, 1447m, 1420m, 1392m, 1367m, 1315m, 1249s, 1164s, 1092m, 1082m, 1054m, 1033m, 977s, 935m, 863m, 790m, 773m, 738m, 709m, 658m, 608m, 571m, 550m, 535m, 462m, 446m, 425m cm^{-1};

HRMS (ESI) for $C_{17}H_{20}O_4N_3Cl_2^+$ $[M+H]^+$: calculated: 400.08254; found: 400.08232.

AM-109

Preparation of *tert*-Butyl ((5-(3,5-dichloro-2-hydroxyphenyl)-1,3,4-oxadiazol-2-yl)methyl)carbamate (17e)

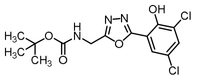

The deallylation was carried out according to **GP4** to yield *tert-b*utyl ((5-(3,5-dichloro-2-hydroxyphenyl)-1,3,4-oxadiazol-2-yl)methyl)carbamate (**17e**) (0.18 g, 0.50 mmol, 75%) as a yellow solid.

Analytical Data of *tert*-Butyl ((5-(3,5-dichloro-2-hydroxyphenyl)-1,3,4-oxadiazol-2-yl)methyl)carbamate (17e)

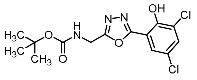

m.p.: 150 °C

^{1}H NMR (500 MHz, $CDCl_3$) δ 10.61 (br. s, 1H), 7.68 – 7.64 (m, 1H), 7.56 – 7.52 (m, 1H), 5.15 (br. s, 1H), 4.67 (d, *J* = 5.9 Hz, 2H), 1.49 (s, 9H) ppm;

^{13}C NMR (126 MHz, $CDCl_3$) δ 155.5, 152.4, 133.8, 125.0, 124.6, 123.8, 109.8, 81.2, 36.0, 28.4 ppm;

Note: One quaternary carbon was not observed or overlaps with another one.

IR (thin film): ν 3344w, 2979w, 1701s, 1621w, 1575w, 1541m, 1513m, 1482m, 1428m, 1392m, 1368m, 1246s, 1165s, 1056w, 992w, 936w, 864m, 804w, 756m, 730m, 657w, 563w cm^{-1};

HRMS (ESI) for $C_{14}H_{16}O_4N_3Cl_2^+$ $[M+H]^+$: calculated: 360.05124; found: 360.05086.

AM-111

Preparation of Oxadiazole 18

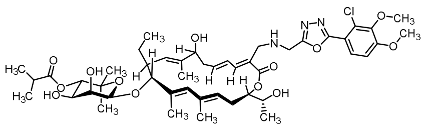

Prepared according to **GP1** at 56.7 µmol scale using (5-(2-chloro-3,4-dimethoxyphenyl)-1,3,4-oxadiazol-2-yl)methanamine (5.0 eq.) as the nucleophile. The residue was purified by preparative RP-HPLC ([Gemini NX, C18, 5 µ, 110 Å, 250 mm × 21.2 mm, solvent A: H_2O + 0.1% HCOOH, solvent B: MeCN + 0.1% HCOOH, 20 mL/min; LC time program (min – %B): 7 min – 20%, 60 min – 40%]) to yield, after concentration *in vacuo*, the oxadiazole **18** (t_R = 36.7 min, 13.5 mg, 14.7 µmol, 26%) as an off-white solid.

Analytical Data of Oxadiazole 18

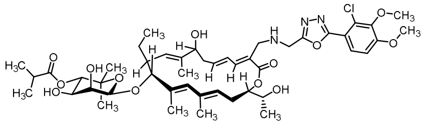

^{1}H NMR (500 MHz, $CDCl_3$) δ 7.77 – 7.63 (m, 1H), 6.97 – 6.93 (m, 1H), 6.90 (d, *J* = 11.6 Hz, 1H), 6.51 (dd, *J* = 14.8, 11.6 Hz, 1H), 5.73 (s, 1H), 5.72 – 5.66 (m, 1H), 5.23 – 5.15 (m, 1H), 5.06 (d, *J* = 10.7 Hz, 1H), 4.98 (d, *J* = 10.0 Hz, 1H), 4.64 – 4.61 (m, 1H), 4.60 (s, 1H), 4.25 (s, 1H), 4.14 (dq, *J* = 12.2, 5.6, 3.9 Hz, 1H), 4.06 – 4.03 (m, 2H), 4.02 – 4.00 (m, 1H), 3.95 (s, 3H), 3.90 (s, 3H), 3.69 – 3.64 (m, 2H), 3.64 – 3.60 (m, 1H), 3.52 (s, 1H), 2.84 – 2.74 (m, 1H), 2.70 – 2.55 (m, 3H), 2.54 – 2.45 (m, 1H), 2.16 – 2.09 (m, 1H), 1.93 – 1.88 (m, 1H), 1.86 (s, 3H), 1.75 (s, 3H), 1.65 (s, 3H), 1.28 – 1.21 (m, 1H), 1.18 (d, *J* = 7.0 Hz, 9H), 1.11 (s, 3H), 1.03 (s, 3H), 0.82 (t, *J* = 7.4 Hz, 3H) ppm;

^{13}C NMR (126 MHz, $CDCl_3$) δ 177.3, 169.3, 166.0, 163.3, 156.4, 146.6, 142.5, 140.0, 136.4, 136.3, 134.5, 134.1, 129.4, 128.4, 128.3, 126.8, 126.6, 123.3, 116.3, 110.7, 94.5, 92.4, 79.3, 76.8, 74.9, 73.4, 73.0, 71.6, 70.2, 68.6, 56.4, 44.7, 42.9, 41.9, 37.3, 34.3, 28.1, 27.6, 25.7, 19.2, 18.9, 18.4, 18.2, 16.9, 15.5, 13.7, 11.1 ppm;

IR (thin film): ν 3410w, 2975m, 2935m, 2876w, 1735m, 1692m, 1641w, 1594m, 1484s, 1455m, 1436m, 1412m, 1384m, 1370m, 1347w, 1296m, 1279m, 1251m, 1210m, 1148m, 1098m, 1074m, 1037s, 1008m, 898w, 813w, 798w, 763w, 732w, 675w cm^{-1};

HRMS (ESI) for $C_{47}H_{67}ClO_{13}N_3^+$ $[M+H]^+$: calculated: 916.43569; found: 916.43562.

ERJ-467

Preparation of *tert*-Butyl (2-(2-(2-chloro-3,4-dimethoxybenzoyl)hydrazineyl)-2-oxoethyl)carbamate (18a)

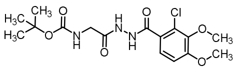

2-Chloro-3,4-dimethoxybenzoic acid (0.75 g, 3.5 mmol, 1.0 eq.) and CDI (0.63 g, 3.2 mmol, 1.2 eq.) were added to acetonitrile (8 mL) and stirred for 30 min at RT. Then, *tert*-butyl (2-hydrazineyl-2-oxoethyl)carbamate (0.60 g, 3.2 mmol, 1.0 eq.) was added. The solution was stirred for 12 h at RT. The solution was diluted with a hydrochloric acid solution (1 M aq., 10 mL), extracted with EtOAc (3 × 10 mL). The combined organic layers were washed with water (5 mL), brine (5 mL), dried over Na_2SO_4, filtered, and concentrated *in vacuo*. The mixture was purified *via* column chromatography (silica gel, pentane/EtOAc 1:2) to yield *tert*-butyl (2-(2-(2-chloro-3,4-dimethoxybenzoyl)hydrazineyl)-2-oxoethyl)carbamate (**18a**, 0.86 g, 2.2 mmol, 70 %) as a white solid.

Analytical Data of *tert*-Butyl (2-(2-(2-chloro-3,4-dimethoxybenzoyl)hydrazineyl)-2-oxoethyl)carbamate (18a)

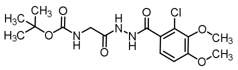

m.p.: 114 °C

^{1}H NMR (500 MHz, $CDCl_3$) δ 9.38 (br. s, 1H), 9.13 (br. s, 1H), 7.55 – 7.50 (m, 1H), 6.93 – 6.83 (m, 1H), 5.37 (br. s, 1H), 4.02 – 3.96 (m, 2H), 3.90 (s, 3H), 3.85 (s, 3H), 1.45 (s, 9H) ppm;

^{13}C NMR (126 MHz, $CDCl_3$) δ 166.9, 163.0, 156.3, 145.9, 126.6, 126.6, 124.4, 110.6, 80.8, 60.9, 56.3, 43.2, 28.4 ppm;

*Note: One quaternary carbon adjacent to a methoxy group was not observed or overlaps with another one (see relative intensity of quaternary carbons in ^{13}C NMR of compound **18b**).*

IR (thin film): ν 3261w, 2978w, 2938w, 1686s, 1591m, 1487s, 1405m, 1367m, 1297s, 1275s, 1223m, 1166s, 1042s, 945w, 916w, 861w, 815w, 779w, 732m, 670w cm^{-1};

HRMS (ESI) for $C_{16}H_{23}O_6N_3Cl^+$ $[M+H]^+$: calculated: 388.12699; found: 388.12624.

AM-88

Preparation of *tert*-Butyl ((5-(2-chloro-3,4-dimethoxyphenyl)-1,3,4-oxadiazol-2-yl)methyl)carbamate (18b)

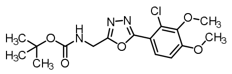

tert-Butyl-(2-(2-(2-chloro-3,4-dimethoxybenzoyl)hydrazineyl)-2-oxoethyl)carbamate (**18a,** 0.45 g, 1.2 mmol, 1.0 eq.) was dissolved in DCM (5.5 mL) at 30 °C. Afterwards triethylamine (0.49 mL, 3.5 mmol, 3.0 eq.) and *p*-toluenesulfonyl chloride (0.21 g, 1.4 mmol, 1.2 eq.) were added. The mixture was stirred at 30 °C for 4 h. The reaction mixture was diluted with water (5 mL) and then extracted with EtOAc (3 × 5 mL). The combined organic layers were washed with brine (5 mL), dried over Na_2SO_4, filtered, and concentrated *in vacuo*. The mixture was purified *via* column chromatography (silica gel, pentane/EtOAc 1:1) to yield *tert*-butyl ((5-(2-chloro-3,4-dimethoxyphenyl)-1,3,4-oxadiazol-2-yl)methyl)carbamate (**18b**, 0.33 g, 0.88 mmol, 70%) as a white powder.

Analytical Data of *tert*-Butyl ((5-(2-chloro-3,4-dimethoxyphenyl)-1,3,4-oxadiazol-2-yl)methyl)carbamate (18b)

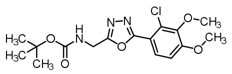

m.p.: 102 °C

^{1}H NMR (500 MHz, $CDCl_3$) δ 7.68 (d, *J* = 8.7 Hz, 1H), 6.93 (d, *J* = 8.8 Hz, 1H), 5.22 (s, 1H), 4.64 (d, *J* = 5.5 Hz, 2H), 3.94 (s, 3H), 3.89 (s, 3H), 1.47 (s, 9H) ppm;

^{13}C NMR (126 MHz, $CDCl_3$) δ 164.1, 163.9, 156.4, 155.5, 146.5, 128.4, 126.9, 116.3, 110.7, 80.7, 60.9, 56.4, 36.1, 28.4 ppm;

IR (thin film): ν 3350w, 2976w, 1713m, 1596m, 1484s, 1454m, 1413m, 1367m, 1285m, 1168m, 1042m, 1007m, 820w cm^{-1};

HRMS (ESI) for $C_{16}H_{21}O_5N_3Cl^+$ $[M+H]^+$: calculated: 370.11642; found: 370.11606.

AM-112

Preparation of Oxadiazole 19

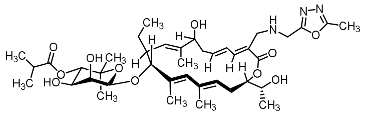

Prepared according to **GP1** at 94.5 µmol scale using (5-methyl-1,3,4-oxadiazol-2-yl)methanamine (5.0 eq.) as the nucleophile. The residue was purified by preparative RP-HPLC ([Gemini NX, C18, 5 µ, 110 Å, 250 mm × 21.2 mm, solvent A: H_2O + 0.1% HCOOH, solvent B: MeCN + 0.1% HCOOH, 20 mL/min; LC time program (min – %B): 7 min – 15%, 60 min – 40%]) to yield, after concentration *in vacuo*, the oxadiazole **19** (t_R = 28.4 min, 21.6 mg, 28.4 µmol, 30%) as an off-white solid.

Analytical Data of Oxadiazole 19

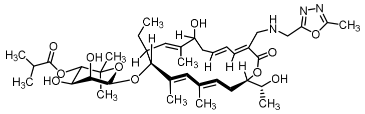

^{1}H NMR (500 MHz, $CDCl_3$) δ 7.04 (d, *J* = 11.5 Hz, 1H), 6.47 (dd, *J* = 14.8, 11.5 Hz, 1H), 5.85 (s, 1H), 5.78 (ddd, *J* = 15.1, 10.8, 4.4 Hz, 1H), 5.41 (dd, *J* = 10.4, 6.0 Hz, 1H), 5.03 – 4.96 (m, 2H), 4.67 – 4.63 (m, 2H), 4.29 – 4.23 (m, 1H), 4.17 – 4.10 (m, 1H), 4.01 (d, *J* = 3.3 Hz, 1H), 3.95, 3.89 (AB_q, 2H, J_{AB} = 15.5 Hz), 3.69 – 3.61 (m, 2H), 3.59 – 3.51 (m, 2H), 2.88 – 2.79 (m, 1H), 2.73 – 2.63 (m, 2H), 2.60 (p, *J* = 7.0 Hz, 1H), 2.56 – 2.47 (m, 4H), 2.21 (dt, *J* = 15.0, 4.6 Hz, 1H), 1.95 (s, 3H), 1.89 (ddd, *J* = 13.2, 7.6, 2.9 Hz, 1H), 1.81 (s, 3H), 1.66 (s, 3H), 1.24 – 1.17 (m, 10H), 1.14 (s, 3H), 1.09 (s, 3H), 0.82 (t, *J* = 7.4 Hz, 3H) ppm;

^{13}C NMR (126 MHz, $CDCl_3$) δ 177.3, 169.2, 165.8, 164.3, 142.8, 140.0, 136.5, 136.3, 134.4, 134.3, 129.3, 128.3, 126.2, 123.2, 94.7, 92.6, 79.4, 74.9, 73.4, 72.7, 71.6, 70.2, 68.6, 44.2, 42.4, 42.0, 37.2, 34.3, 28.2, 27.8, 25.9, 19.2, 18.9, 18.5, 18.3, 17.0, 15.4, 13.9, 11.2, 11.1 ppm;

IR (thin film): ν 3401w, 2975w, 2932w, 2874w, 1734m, 1691m, 1641w, 1595w, 1440w, 1384m, 1369m, 1347m, 1298m, 1252m, 1201m, 1148m, 1074m, 1032s, 978m, 950m, 897m, 841w, 797m, 732s, 701m, 583m, 503m, 463m, 419m cm^{-1};

HRMS (ESI) for $C_{40}H_{62}O_{11}N_3^+$ $[M+H]^+$: calculated: 760.43789; found: 760.43791.

ERJ-450

Preparation of Thiadiazole 20

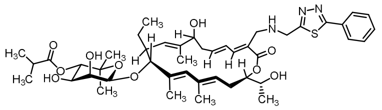

Prepared according to **GP1** At 50.0 µmol scale using (5-phenyl-1,3,4-thiadiazol-2-yl)methanamine (5.0 eq.) as the nucleophile. The residue was purified twice by preparative RP-HPLC ([Gemini NX, C18, 5 µ, 110 Å, 250 mm × 21.2 mm, solvent A: H_2O + 0.1% HCOOH, solvent B: MeCN + 0.1% HCOOH, 18 mL/min; LC time program (min – %B): 5 min – 25%, 60 min – 40%]) to yield, after concentration *in vacuo*, the thiadiazole **20** (t_R = 27.8 min, 3.0 mg, 3.6 µmol, 7%) as an off-white solid.

Analytical Data of Thiadiazole 20

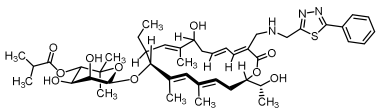

^{1}H NMR (500 MHz, $CDCl_3$) δ 7.98 – 7.93 (m, 2H), 7.52 – 7.47 (m, 3H), 7.04 (d, *J* = 11.5 Hz, 1H), 6.34 (dd, *J* = 14.8, 11.6 Hz, 1H), 5.82 – 5.73 (m, 2H), 5.37 (dt, *J* = 10.6, 4.5 Hz, 1H), 5.11 (d, *J* = 10.7 Hz, 1H), 4.99 (d, *J* = 10.0 Hz, 1H), 4.66 (dt, *J* = 10.8, 3.1 Hz, 1H), 4.59 (s, 1H), 4.34 – 4.30 (m, 1H), 4.18 – 4.14 (m, 2H), 4.14 – 4.08 (m, 1H), 4.00 (d, *J* = 3.4 Hz, 1H), 3.69 – 3.62 (m, 2H), 3.61 – 3.53 (m, 2H), 2.85 – 2.75 (m, 1H), 2.67 (ddt, *J* = 21.5, 15.6, 6.8 Hz, 2H), 2.59 (q, *J* = 7.0 Hz, 1H), 2.47 (ddd, *J* = 14.8, 11.0, 5.0 Hz, 1H), 2.21 (dd, *J* = 12.1, 7.3 Hz, 1H), 1.90 (s, 3H), 1.79 (s, 3H), 1.67 (s, 3H), 1.25 (s, 2H), 1.20 (dd, *J* = 10.4, 6.7 Hz, 9H), 1.13 (s, 3H), 1.05 (s, 3H), 0.84 (t, *J* = 7.4 Hz, 3H) ppm;

^{13}C NMR (126 MHz, $CDCl_3$) δ 177.3, 173.0, 169.7, 169.4, 143.0, 140.1, 136.5, 136.3, 134.4, 134.3, 131.3, 130.4, 129.4 (2 C), 128.3, 128.0, 126.2, 123.1, 94.6, 92.7, 79.7, 74.9, 73.4, 72.8, 71.6, 70.2, 68.9, 46.9, 44.1, 42.0, 37.5, 34.3, 28.2, 28.1, 25.9, 19.2, 18.9, 18.4, 18.3, 16.9, 15.4, 13.7, 11.2 ppm;

IR (thin film): ν 3676w, 3451w, 2973m, 2934, 1738m, 1692m, 1641w, 1458m, 1384m, 1252m, 1147m, 1067s, 1038s, 897w, 763w, 691w, 577w, 543w, 469w, 462w, 450w, 431m, 418w, 408m cm^{-1};

HRMS (ESI) for $C_{45}H_{64}O_{10}N_3S^+$ $[M+H]^+$: calculated: 838.43069; found: 838.43125.

ERJ-543, ERJ-634

Preparation of *tert*-Butyl (2-(2-benzoylhydrazineyl)-2-oxoethyl)carbamate (20a)

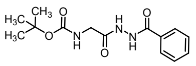

Benzoic acid (1.0 g, 5.3 mmol, 1.0 eq.) and CDI (1.1 g, 6.5 mmol, 1.2 eq.) were added to acetonitrile (18 mL) and stirred for 30 min at RT. Then, *tert*-butyl-(2-hydrazineyl-2-oxoethyl)carbamate (1.0 g, 5.3 mmol, 1.0 eq.) was added. The solution was stirred for 12 h at RT. The solution was diluted with a hydrochloric acid solution (1 M aq., 20 mL) and extracted with EtOAc (3 × 12 mL). The combined organic layers were washed with water (5 mL), and brine (10 mL), dried over Na_2SO_4, filtered, and concentrated *in vacuo*. The crude was purified *via* column chromatography (silica gel, pentane/EtOAc 3:2) to yield *tert*-butyl (2-(2-benzoylhydrazineyl)-2-oxoethyl)carbamate (**20a**, 0.83 g, 2.8 mmol, 54 %) as a white solid.

Analytical Data of *tert*-Butyl (2-(2-benzoylhydrazineyl)-2-oxoethyl)carbamate (20a)

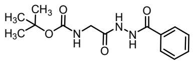

m.p.: 149 °C

^{1}H NMR (500 MHz, DMSO-*d*$_6$) δ 10.35 (s, 1H), 9.92 (s, 1H), 7.90 – 7.84 (m, 2H), 7.61 – 7.54 (m, 1H), 7.49 (m, 2H), 7.05 (m, 1H), 3.66 (d, J = 6.2 Hz, 2H), 1.39 (s, 9H) ppm;

^{13}C NMR (126 MHz, DMSO-*d*$_6$) δ 168.9, 165.4, 155.8, 132.5, 131.8, 128.4, 127.4, 78.0, 41.7, 28.2 ppm;

IR (thin film): ν 3402w, 3306w, 3255w, 1713m, 1696s, 1654s, 1528m, 1488m, 1409w, 1396w, 1371w, 1324w, 1281s, 1251s, 1162s, 1152s, 1056m, 1029w, 941w, 849w, 768w, 719s, 697m, 618w, 602w, 586w, 565w, 495w, 460w cm^{-1};

HRMS (ESI) for $C_{14}H_{20}O_4N_3^+$ $[M+H]^+$: calculated: 294.14483; found: 294.14428.

AM-126

Preparation of *tert*-Butyl ((5-phenyl-1,3,4-thiadiazol-2-yl)methyl)carbamate (20b)

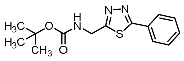

To a suspension of *tert*-butyl-(2-(2-benzoylhydrazineyl)-2-oxoethyl)carbamate (0.10 g, 0.34 mmol, 1.0 eq.) in toluene (8.5 mL), was added Lawesson's reagent (0.15 g, 0.38 mmol, 1.1 eq.), and the solution was heated to reflux for 1 h. The solvent was removed *in vacuo* and the residue was purified *via* column chromatography (silica gel, pentane/EtOAc 5:1) to yield *tert*-butyl ((5-phenyl-1,3,4-thiadiazol-2-yl)methyl)carbamate (**20b**, 78 mg, 0.27 mmol, 79%) as a light-yellow solid.

Analytical Data of *tert*-Butyl ((5-phenyl-1,3,4-thiadiazol-2-yl)methyl)carbamate (20b)

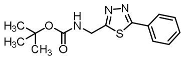

m.p.: 81 °C

^{1}H NMR (500 MHz, $CDCl_3$) δ 7.98 – 7.92 (m, 2H), 7.54 – 7.44 (m, 3H), 5.37 (br. s, 1H), 4.76 (s, 2H), 1.48 (s, 9H) ppm;

^{13}C NMR (126 MHz, $CDCl_3$) δ 170.1, 168.0, 155.8, 131.4 (d, *J* = 5.1 Hz), 130.1, 129.3, 128.1, 80.8, 39.9, 28.5 ppm;

IR (thin film): ν 3328w, 2978w, 1704s, 1514m, 1458m, 1428m, 1392w, 1367m, 1280m, 1251s, 1165s, 1044w, 981w, 933w, 861w, 763m, 690m cm^{-1};

HRMS (ESI) for $C_{14}H_{18}O_{12}N_3S^+$ $[M+H]^+$: calculated: 292.11142; found: 292.11120.

AM-129

Preparation of Oxadiazole 21

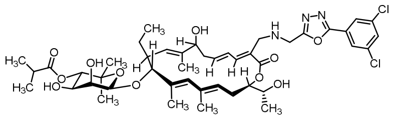

Prepared according to **GP1** at 60.0 µmol scale using (5-(3,5-dichlorophenyl)-1,3,4-oxadiazol-2-yl)methanamine (5.0 eq.) as the nucleophile. The residue was purified three times by preparative RP-HPLC (First run: [Gemini NX, C18, 5 µ, 110 Å, 250 mm × 21.2 mm, solvent A: H_2O + 0.1% HCOOH, solvent B: MeCN + 0.1% HCOOH, 18 mL/min; LC time program (min – %B): 5 min – 30%, 60 min – 60%, t_R = 29.0 min], second run: [Gemini NX, C18, 5 µ, 110 Å, 250 mm × 21.2 mm, solvent A: H_2O + 0.1% HCOOH, solvent B: MeCN + 0.1% HCOOH, 18 mL/min; LC time program (min – %B): 5 min – 35%, 60 min – 50%, t_R = 22.6 min], third run: [Gemini NX, C18, 5 µ, 110 Å, 250 mm × 21.2 mm, solvent A: H_2O + 0.1% HCOOH, solvent B: MeCN + 0.1% HCOOH, 18 mL/min; LC time program (min – %B): 5 min – 25%, 60 min – 45%, t_R = 45.4 min]) to yield, after concentration *in vacuo*, the oxadiazole **21** (8.3 mg, 9.3 µmol, 16%) as an off-white solid.

Analytical Data of Oxadiazole 21

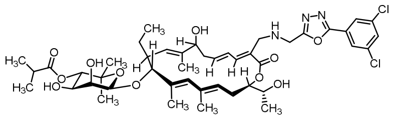

^{1}H NMR (500 MHz, $CDCl_3$) δ 7.93 – 7.89 (m, 2H), 7.58 – 7.53 (m, 1H), 6.76 (d, *J* = 11.6 Hz, 1H), 6.50 (dd, *J* = 14.8, 11.6 Hz, 1H), 5.69 (s, 1H), 5.69 – 5.63 (m, 1H), 5.09 (dd, *J* = 10.9, 5.6 Hz, 1H), 5.02 (d, *J* = 10.8 Hz, 1H), 4.98 (d, *J* = 10.0 Hz, 1H), 4.62 (s, 1H), 4.55 (dt, *J* = 10.9, 3.0 Hz, 1H), 4.29 – 4.24 (m, 1H), 4.24 – 4.16 (m, 1H), 4.12 – 3.97 (m, 3H), 3.70 – 3.56 (m, 4H), 2.87 – 2.76 (m, 1H), 2.73 – 2.64 (m, 2H), 2.64 – 2.56 (m, 1H), 2.52 (ddd, *J* = 14.6, 11.1, 4.9 Hz, 1H), 2.09 (dt, *J* = 15.1, 4.7 Hz, 1H), 1.95 – 1.86 (m, 1H), 1.75 (s, 3H), 1.73 (s, 3H), 1.66 (s, 3H), 1.29 – 1.21 (m, 1H), 1.20 – 1.17 (m, 9H), 1.11 (s, 3H), 1.04 (s, 3H), 0.83 (t, *J* = 7.4 Hz, 3H) ppm;

^{13}C NMR (126 MHz, $CDCl_3$) δ 177.3, 168.9, 167.2, 162.6, 141.9, 140.1, 136.7, 136.5, 136.2, 134.4, 134.2, 131.8, 129.3, 128.4, 127.0, 126.5, 125.4, 123.2, 94.5, 92.7, 79.0, 74.9, 73.3, 73.0, 71.7, 70.2, 68.3, 44.8, 42.9, 42.0, 37.3, 34.3, 28.1, 26.9, 25.8, 19.2, 18.9, 18.4, 18.2, 16.6, 15.5, 13.7, 11.1 ppm;

IR (thin film): ν 3451w, 2974m, 2932m, 2875w, 1737m, 1694s, 1641w, 1547w, 1443m, 1409w, 1385m, 1369m, 1348w, 1295w, 1254m, 1207m, 1148s, 1073s, 1035s, 995m, 979m, 896m, 867w, 805m, 729w, 670w, 410w cm^{-1};

HRMS (ESI) for $C_{45}H_{62}O_{11}N_3Cl_2^+$ $[M+H]^+$: calculated: 890.37559; found: 890.37716.

ERJ-471, ERJ-635

Preparation of *tert*-Butyl (2-(2-(3,5-dichlorobenzoyl)hydrazineyl)-2-oxoethyl)carbamate (21a)

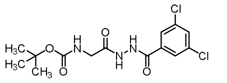

To a solution of *N*-Boc glycine in dry MeCN (2 mL) was added CDI (195 mg, 1.20 mmol, 1.2 eq.) at RT. The mixture was stirred for 15 min at RT after which 3,5-dichlorobenzohydrazide (209 mg, 1.00 mmol, 1.0 eq.) was added followed by dry DMF (2 mL). The reaction mixture was heated to 40 °C for 24 h. The reaction mixture was partitioned between EtOAc (20 mL) and a hydrochloric acid solution (1 M aq., 20 mL). The organic layer was separated, and the aqueous layer was extracted with EtOAc (2 × 15 mL). The combined organic layers were washed with water (20 mL) and brine (20 mL). The organic layer was then directly concentrated onto silica gel in vacuo and purified *via* flash column chromatography (silica gel, pentane/EtOAc 1:1 -> 1:2) to yield *tert*-butyl (2-(2-(3,5-dichlorobenzoyl)hydrazineyl)-2-oxoethyl)carbamate (**21a**, 123 mg, 0.340 mmol, 34%) as a colorless solid.

Note: Both starting material and product have extremely poor solubility in most organic solvents and may have to be transferred as suspensions.

Alternatively, the product can be obtained by coupling of 3,5-dichlorobenzoic acid (222 mg, 1.16 mmol, 1.1 eq.) in dry MeCN (2.5 mL) with CDI (206 mg, 1.27 mmol, 1.2 eq.), followed by addition of *tert*-butyl (2-hydrazineyl-2-oxoethyl)carbamate (200 mg, 1.06 mmol, 1.0 eq.). The reaction mixture was stirred at RT overnight and the resulting suspension was partitioned between a hydrochloric acid solution (1 M aq., 20 mL) and EtOAc (20 mL). The organic layer was separated, and the aqueous layer was extracted with EtOAc (2 × 20 mL). The combined organic layers were washed using water (20 mL) and brine (20 mL). The resulting turbid organic layer was concentrated *in vacuo* and the residue was triturated using hot DCM/MeOH and the filtrate was concentrated onto silica gel *in vacuo* and subsequently purified using flash column chromatography (silica gel, pentane/EtOAc 2:1 → 1:2) to yield *tert*-butyl (2-(2-(3,5-dichlorobenzoyl)hydrazineyl)-2-oxoethyl)carbamate (**21a**, 94.0 mg, 0.260 mmol, 25%) as a colorless solid.

Analytical Data of *tert*-Butyl (2-(2-(3,5-dichlorobenzoyl)hydrazineyl)-2-oxoethyl)carbamate (21a)

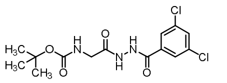

^{1}H NMR (500 MHz, DMSO-d_6) δ 10.61 (br. s, 1H), 10.06 (s, 1H), 7.91 – 7.85 (m, 2H), 7.10 – 7.04 (m, 1H), 3.66 (d, J = 6.3 Hz, 2H), 1.39 (s, 9H) ppm;

^{13}C NMR (126 MHz, DMSO-d_6) δ 168.8, 162.7, 155.8, 135.5, 134.5, 131.3, 126.2, 78.1, 41.7, 28.2 ppm;

IR (thin film): ν 3261m, 3076w, 3006w, 2980w, 2934w, 1779w, 1685s, 1567s, 1513s, 1453m, 1418m, 1392m, 1367m, 1325m, 1246s, 1164s, 1101w, 1051w, 1030w, 943w, 868m, 806m, 757w, 737m, 702w, 667m, 588w, 539w, 484w, 465w, 440w, 433w, 406w cm^{-1};

HRMS (ESI) for $C_{14}H_{17}O_4N_3Cl_2Na^+$ $[M+Na]^+$: calculated: 384.04883; found: 384.04866.

ERJ-622, ERJ-629

Preparation of
***tert*-Butyl ((5-(3,5-dichlorophenyl)-1,3,4-oxadiazol-2-yl)methyl)carbamate (21b)**

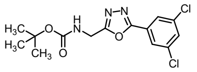

To *tert*-butyl (2-(2-(3,5-dichlorobenzoyl)hydrazineyl)-2-oxoethyl)carbamate (**21a**, 188 mg, 0.519 mmol, 1.0 eq.) in dry DCM (4.66 mL) was added triethylamine (0.22 mL, 1.56 mmol, 3.0 eq.) at RT, followed by *p*-toluenesulfonyl chloride (119 mg, 0.623 mmol, 1.2 eq.). The reaction mixture was stirred at RT for 4 h. The reaction mixture was partitioned between EtOAc (30 mL) and an ammonium chloride solution (sat. aq., 15 mL). The organic layer was separated, and the aqueous layer was extracted using EtOAc (2 × 15 mL). The combined organic layers were washed using a sodium bicarbonate solution (sat. aq., 20 mL), water (20 mL), brine (30 mL), dried over sodium sulfate, filtered, and concentrated *in vacuo*. The resulting residue was purified *via* flash column chromatography (silica gel, pentane/EtOAc 3:1) to yield *tert*-butyl ((5-(3,5-dichlorophenyl)-1,3,4-oxadiazol-2-yl)methyl)carbamate (**21b,** 111 mg, 0.322 mmol, 62%) as a colorless solid.

Analytical Data of
***tert*-Butyl ((5-(3,5-dichlorophenyl)-1,3,4-oxadiazol-2-yl)methyl)carbamat*e* (21b)**

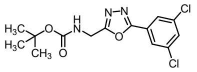

^{1}H NMR (500 MHz, $CDCl_3$) δ 7.97 – 7.90 (m, 2H), 7.55 – 7.50 (m, 1H), 5.24 – 5.13 (m, 1H), 4.64 (d, *J* = 6.1 Hz, 2H), 1.51 – 1.45 (m, 9H) ppm;

^{13}C NMR (126 MHz, $CDCl_3$) δ 164.7, 163.3, 155.5, 136.2, 131.9, 126.4, 125.3, 81.0, 36.1, 28.4 ppm;

IR (thin film): ν 3344w, 3077w, 2979w, 2934w, 1719s, 1567m, 1546s, 1518m, 1445m, 1410m, 1392m, 1367m, 1323w, 1281m, 1252m, 1215m, 1167s, 1105w, 1055w, 1034w, 981w, 937w, 864m, 806m, 726w, 669m, 605w, 579w, 545w cm^{-1};

HRMS (ESI) for $C_{14}H_{16}O_3N_3Cl_2^+$ $[M+H]^+$: calculated: 344.05632; found: 344.05577.

ERJ-469, ERJ-632

Preparation of Oxadiazole 22

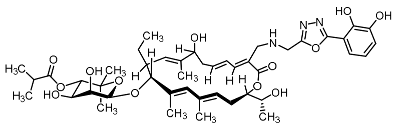

Prepared according to **GP1** at 23.6 µmol scale using 3-(5-(aminomethyl)-1,3,4-oxadiazol-2-yl)benzene-1,2-diol (5.0 eq.) as the nucleophile. The residue was purified by preparative RP-HPLC ([Gemini NX, C18, 5 µ, 110 Å, 250 mm × 21.2 mm, solvent A: H_2O + 0.1% HCOOH, solvent B: MeCN + 0.1% HCOOH, 20 mL/min; LC time program (min – %B): 7 min – 10%, 60 min – 40%]) to yield, after concentration *in vacuo*, the oxadiazole **22** (t_R = 40.9 min, 4.9 mg, 5.7 µmol, 24%) as an off-white solid.

Analytical Data of Oxadiazole 22

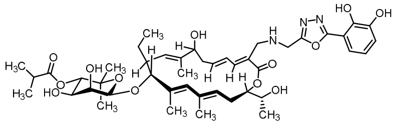

^{1}H NMR (500 MHz, $CDCl_3$) δ 7.25 – 7.23 (m, 1H), 7.12 (d, *J* = 7.9 Hz, 1H), 6.94 (t, *J* = 8.0 Hz, 1H), 6.83 (d, *J* = 11.5 Hz, 1H), 6.50 (t, *J* = 13.2 Hz, 1H), 5.68 (ddd, *J* = 15.8, 11.3, 4.6 Hz, 1H), 5.63 (s, 1H), 5.04 (d, *J* = 10.8 Hz, 1H), 5.00 (d, *J* = 9.9 Hz, 1H), 4.92 – 4.85 (m, 1H), 4.64 (s, 1H), 4.54 (d, *J* = 10.6 Hz, 1H), 4.26 (s, 1H), 4.16 – 4.03 (m, 4H), 3.71 (t, *J* = 10.2 Hz, 2H), 3.62 (d, *J* = 7.6 Hz, 2H), 3.53 (s, 1H), 2.74 – 2.56 (m, 4H), 2.55 – 2.46 (m, 1H), 2.10 – 2.01 (m, 1H), 2.00 – 1.89 (m, 1H), 1.85 (s, 3H), 1.75 (s, 3H), 1.66 (s, 3H), 1.24 (q, *J* = 8.1, 6.7 Hz, 1H), 1.20 – 1.15 (m, 9H), 1.13 (s, 3H), 1.05 (s, 3H), 0.85 (t, *J* = 7.4 Hz, 3H) ppm;

^{13}C NMR (126 MHz, $CDCl_3$) δ 177.3, 169.6, 165.1, 164.5, 145.5, 144.8, 142.5, 140.3, 136.7, 136.3, 134.5, 134.1, 129.4, 128.4, 126.4, 123.2, 120.8, 119.1, 117.7, 108.1, 94.5, 92.1, 79.8, 76.7, 74.9, 73.5, 73.1, 71.2, 70.2, 68.9, 45.0, 42.9, 41.9, 37.4, 34.3, 28.2, 25.8, 19.2, 18.9, 18.7, 18.1, 16.8, 15.6, 13.5, 11.1 δ ppm;

IR (thin film): ν 3397m, 2976m, 2931m, 2875w, 1733m, 1691m, 1640m, 1551w, 1473m, 1385m, 1370m, 1252s, 1211s, 1146s, 1074s, 1033s, 997m, 894m, 824w, 796w, 761w, 739m, 632w, 591w, 523w, 507w cm^{-1};

HRMS (ESI) for $C_{45}H_{64}O_{13}N_3^+$ $[M+H]^+$: calculated: 854.44337; found: 854.44295.

ERJ-468

Preparation of Allyl 2,3-bis(allyloxy)benzoate (22a)

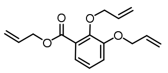

Carried out according to a modified literature procedure.[10] A suspension of 2,3-dihydroxybenzoic acid (1.00 g, 6.49 mmol, 1.0 eq.) and potassium carbonate (3.59 g, 25.9 mmol, 4.0 eq.) in dry acetone (16 mL) was heated in a microwave vial to 60 °C. Then, allyl bromide (2.26 mL, 25.9 mmol, 4.0 eq.) was added. The reaction was heated to 60 °C for 18 h. Then, the solution was cooled to RT and was diluted with water (10 mL) and extracted with EtOAc (3 × 10 mL). The combined organic layers were washed with brine, dried over Na_2SO_4, filtered, and concentrated *in vacuo*. This yielded the allyl 2,3-bis(allyloxy)benzoate (**22a**) as a brown oil (1.12 g, 4.02 mmol, 63%) which was used directly in the next step. An aliquot was purified using flash column chromatography to obtain an analytically pure sample.

Analytical Data of Allyl 2,3-bis(allyloxy)benzoate (22a)

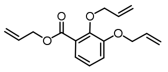

^{1}H NMR (500 MHz, $CDCl_3$) δ 7.39 – 7.31 (m, 1H), 7.10 – 7.02 (m, 2H), 6.18 – 5.97 (m, 3H), 5.47 – 5.18 (m, 6H), 4.81 (dd, *J* = 5.7, 1.6 Hz, 2H), 4.63 – 4.56 (m, 4H) ppm;

^{13}C NMR (101 MHz, $CDCl_3$) δ 166.2, 152.7, 148.4, 134.3, 133.1, 132.3, 126.9, 123.9, 122.8, 118.5, 118.0, 117.8, 117.8, 74.9, 70.1, 65.9 ppm;

IR (thin film): ν 3083w, 3020w, 2985w, 2938w, 2871w, 1728m, 1648w, 1581m, 1473m, 1421m, 1376w, 1359m, 1309m, 1261s, 1221m, 1186m, 1145m, 1088m, 1043m, 989s, 927m, 841w, 808w, 754m, 647w, 556w cm^{-1};

HRMS (ESI) for $C_{16}H_{19}O_4^+$ $[M+H]^+$: calculated: 275.12779; found: 275.12778.

AM-84

Preparation of 2,3-Bis(allyloxy)benzoic acid (22b)

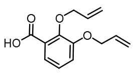

A solution of allyl 2,3-bis(allyloxy)benzoate (1.15 g, 4.19 mmol) in THF (3 mL) and a sodium hydroxide solution (1 M aq., 3 mL) was stirred for 12 h at 60 °C. The reaction was diluted with hydrochloric acid solution (1 M aq., 5 mL) and extracted with EtOAc (3 × 6 mL). The combined organic layers were washed with brine (5 mL), dried over Na_2SO_4, filtered, and concentrated *in vacuo* to yield 2,3-bis(allyloxy)benzoic acid (**22b**) as a brown solid (0.37 g, 1.6 mmol, 38%).

Analytical Data of 2,3-Bis(allyloxy)benzoic acid (22b)

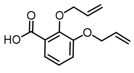

m.p.: 50 °C

^{1}H NMR (400 MHz, $CDCl_3$) δ 7.80 – 7.66 (m, 1H), 7.22 – 7.09 (m, 2H), 6.17 – 6.01 (m, 2H), 5.49 – 5.32 (m, 4H), 4.81 (d, *J* = 6.5 Hz, 2H), 4.63 (dt, *J* = 5.4, 1.5 Hz, 2H) ppm;

^{13}C NMR (101 MHz, $CDCl_3$) δ 165.4, 151.1, 147.0, 132.4, 131.8, 125.0, 124.5, 123.0, 121.6, 119.1, 118.6, 75.9, 70.2 ppm;

IR (thin film): ν 3084w, 1740m, 1695m, 1580m, 1475m, 1421m, 1391m, 1309m, 1263s, 1221m, 1206m, 1155w, 1087m, 1038m, 988m, 928m, 811m, 753m, 654w cm^{-1};

HRMS (ESI) for $C_{13}H_{15}O_4^+$ $[M+H]^+$: calculated: 235.09649; found: 235.09677.

AM-58, AM-90

Preparation of
***tert*-Butyl (2-(2-(2,3-bis(allyloxy)benzoyl)hydrazineyl)-2-oxoethyl)carbamate (22c)**

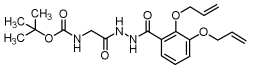

2,3-Bis(allyloxy)benzoic acid (0.30 g, 1.3 mmol, 1.0 eq.) and CDI (0.25 g, 1.6 mmol, 1.2 eq.) were added to acetonitrile (8 mL) and stirred for 30 min at RT. Then, *tert*-butyl (2-hydrazineyl-2-oxoethyl)carbamate (0.25 g, 1.3 mmol, 1.0 eq.) was added. The solution was stirred for 12 h at RT. The solution was diluted with a hydrochloric acid solution (1 M aq., 10 mL), extracted with EtOAc (3 × 10 mL), the combined organic layers were washed with water (5 mL), and brine (5 mL), dried over Na_2SO_4, filtered, and concentrated *in vacuo*. The crude was purified *via* column chromatography (silica gel, pentane/EtOAc 3:2) to yield *tert*-butyl (2-(2-(2,3-bis(allyloxy)benzoyl)hydrazineyl)-2-oxoethyl)carbamate (**22c**) as a white solid (0.47 g, 1.2 mmol, 90%).

Analytical Data of
***tert*-Butyl (2-(2-(2,3-bis(allyloxy)benzoyl)hydrazineyl)-2-oxoethyl)carbamate (22c)**

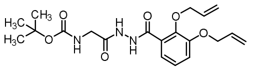

m.p.: 106 °C

^{1}H NMR (400 MHz, $CDCl_3$) 10.63 (s, 1H), 9.28 (s, 1H), 7.80 – 7.66 (m, 1H), 7.18 – 7.06 (m, 2H), 6.29 – 6.00 (m, 2H), 5.48 – 5.26 (m, 4H), 5.25 (s, 1H), 4.84 – 4.71 (m, 2H), 4.61 (ddt, *J* = 10.1, 5.3, 1.5 Hz, 2H), 3.99 (s, 2H), 1.46 (s, 9H) ppm;

Note: Some signals appear twice in the ^{1}H NMR due to presence of rotamers.

^{13}C NMR (101 MHz, $CDCl_3$) δ 151.7, 146.7, 132.9, 132.8, 132.4, 125.0, 124.6, 124.5, 124.5, 123.2, 121.6, 120.4, 119.0, 118.6, 118.2, 118.2, 75.9, 75.3, 70.1, 28.4 ppm;

IR (thin film): ν 3323w, 3163w, 2976m, 2927m, 2869m, 1711m, 1685m, 1651m, 1614s, 1576m, 1511m, 1479s, 1450s, 1423m, 1410m, 1390m, 1377m, 1368m, 1361m, 1305m, 1272s, 1258s, 1235m, 1216s, 1168s, 1100m, 1082m, 1063m, 1041m, 1023m, 993m, 975s, 938s, 924s, 871m, 845m, 805m, 792m, 772m, 751s, 669m, 587m, 565s, 499m, 456m cm^{-1};

HRMS (ESI) for $C_{20}H_{28}O_6N_3^+$ $[M+H]^+$: calculated: 406.19726; found: 406.19691.

AM-95

Preparation of

***tert*-Butyl ((5-(2,3-bis(allyloxy)phenyl)-1,3,4-oxadiazol-2-yl)methyl)carbamate (22d)**

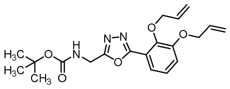

In a flame-dried microwave vial, *tert*-butyl (2-(2-(2,3-bis(allyloxy)benzoyl)hydrazineyl)-2-oxoethyl)carbamate (**22c**, 56.0 mg, 0.138 mmol, 1.0 eq.) was dissolved in dry DCM (1.24 mL) and triethylamine (58 µL, 0.41 mmol, 3.0 eq.) at RT. *p*-Toluenesulfonyl chloride (31.6 mg, 0.166 mmol, 1.2 eq.) was added and the mixture was stirred at RT for 3 h. The reaction mixture was partitioned between EtOAc (15 mL) and an ammonium chloride solution (sat. aq., 15 mL). The organic layer was separated, and the aqueous layer was extracted using EtOAc (2 × 10 mL). The combined organic layers were washed using a sodium bicarbonate solution (sat. aq., 15 mL), water (15 mL), brine (15 mL), dried over sodium sulfate, filtered, and concentrated *in vacuo*. The resulting residue was purified using flash column chromatography (silica gel, pentane/EtOAc 2:1) to yield the product (22.0 mg, 56.8 µmol, 41%).

Analytical Data of

***tert*-Butyl ((5-(2,3-bis(allyloxy)phenyl)-1,3,4-oxadiazol-2-yl)methyl)carbamate (22d)**

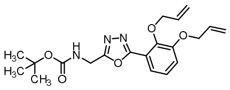

^{1}H NMR (500 MHz, $CDCl_3$) δ 7.54 – 7.49 (m, 1H), 7.17 – 7.10 (m, 1H), 7.10 – 7.05 (m, 1H), 6.17 – 6.02 (m, 2H), 5.44 (dt, *J* = 17.3, 1.5 Hz, 1H), 5.35 (dd, *J* = 17.2, 1.7 Hz, 1H), 5.32 (d, *J* = 1.4 Hz, 1H), 5.25 – 5.17 (m, 2H), 4.67 – 4.59 (m, 6H), 1.46 (s, 9H) ppm;

^{13}C NMR (126 MHz, $CDCl_3$) δ 164.0, 164.0, 155.5, 152.8, 147.2, 134.0, 132.9, 124.6, 122.1, 119.1, 118.2, 118.0, 117.6, 80.6, 74.8, 70.0, 36.2, 28.4 ppm;

IR (thin film): ν 3341w, 3083w, 2979w, 2932w, 2871w, 1714s, 1649w, 1574m, 1537m, 1512m, 1478s, 1453m, 1421m, 1392m, 1367m, 1305m, 1267s, 1250s, 1220s, 1164s, 1088m, 1053m, 1043m, 985s, 931s, 867m, 830w, 792m, 745m, 726m, 651m, 564m, 461m, 448m, 408w cm^{-1};

HRMS (ESI) for $C_{20}H_{26}O_5N_3^+$ $[M+H]^+$: calculated: 388.18670; found: 388.18623.

AM-28, AM-60, ERJ-630

Preparation of 3-(5-(Aminomethyl)-1,3,4-oxadiazol-2-yl)benzene-1,2-diol (22e)

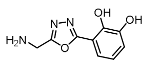

Deallylation of **22d** was carried out according to **GP4** The resulting residue was treated with TFA according to **GP2** to obtain crude 3-(5-(aminomethyl)-1,3,4-oxadiazol-2-yl)benzene-1,2-diol which was used directly in the following allylic substitution.

AM-32, AM-33

Preparation of Oxadiazole 23

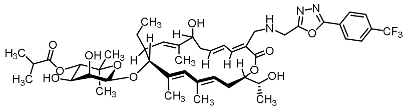

Prepared according to **GP1** At 28.0 µmol scale using (5-(4-(trifluoromethyl)phenyl)-1,3,4-oxadiazol-2-yl)methanamine (4.9 eq.) as the nucleophile. The residue was purified by preparative RP-HPLC ([Gemini NX, C18, 5 µ, 110 Å, 250 mm × 21.2 mm, solvent A: H_2O + 0.1% HCOOH, solvent B: MeCN + 0.1% HCOOH, 20 mL/min; LC time program (min – %B): 7 min – 30%, 60 min – 50%]) to yield, after concentration *in vacuo*, the oxadiazole **23** (t_R = 19.6 min, 6.4 mg, 7.2 µmol, 26%) as an off-white solid.

Analytical Data of Oxadiazole 23

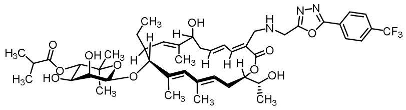

^{1}H NMR (500 MHz, $CDCl_3$) δ 8.23 – 8.10 (m, 2H), 7.82 – 7.75 (m, 2H), 6.88 (d, *J* = 11.5 Hz, 1H), 6.50 (dd, *J* = 14.8, 11.5 Hz, 1H), 5.75 (s, 1H), 5.67 (ddd, *J* = 15.2, 10.8, 4.4 Hz, 1H), 5.24 (dd, *J* = 10.5, 5.9 Hz, 1H), 5.03 – 4.96 (m, 2H), 4.62 (s, 1H), 4.60 – 4.58 (m, 1H), 4.26 (d, *J* = 4.4 Hz, 1H), 4.17 (d, *J* = 6.9 Hz, 1H), 4.13 – 3.99 (m, 3H), 3.68 – 3.60 (m, 4H), 3.59 – 3.42 (m, 1H), 2.81 (dt, *J* = 14.5, 10.5 Hz, 1H), 2.71 – 2.57 (m, 3H), 2.51 (ddd, *J* = 14.7, 10.8, 4.7 Hz, 1H), 2.15 (dt, *J* = 15.0, 4.3 Hz, 1H), 1.94 – 1.86 (m, 1H), 1.79 (s, 3H), 1.73 (s, 3H), 1.65 (s, 3H), 1.24 (d, *J* = 8.6 Hz, 1H), 1.19 (t, *J* = 6.8 Hz, 9H), 1.12 (d, *J* = 1.6 Hz, 3H), 1.06 (d, *J* = 1.6 Hz, 3H), 0.85 – 0.78 (m, 3H) ppm;

^{13}C NMR (126 MHz, $CDCl_3$) δ 177.3, 169.0, 166.8, 163.8, 142.3, 140.0, 136.6, 136.3, 134.3, 134.2, 133.7 (q, *J* = 33.0 Hz), 129.2, 128.3, 127.5, 127.0, 126.8, 126.2 (q, *J* = 3.8 Hz), 123.2, 94.6, 92.6, 79.2, 74.9, 73.4, 72.9, 71.6, 70.2, 68.5, 44.6, 42.8, 42.0, 37.2, 34.3, 28.1, 27.4, 25.8, 19.2, 18.9, 18.4, 18.3, 16.9, 15.4, 13.7, 11.1 ppm;

^{19}F NMR (471 MHz, $CDCl_3$) δ -63.1 ppm;

IR (thin film): ν 2975w, 2931w, 1735w, 1692m, 1641w, 1469w, 1458w, 1419w, 1385w, 1370w, 1324s, 1298w, 1249m, 1204m, 1169m, 1133s, 1086m, 1065s, 1034m, 1016m, 979m, 950w, 897w, 852m, 812w, 797w, 763w, 750w, 735w, 709w, 597w, 507w, 468w, 462w, 450w, 424w cm^{-1};

HRMS (ESI) for $C_{46}H_{63}O_{11}N_3F_3^+$ $[M+H]^+$: calculated: 890.44092; found: 890.44072.

ERJ-477

Preparation of *tert*-Butyl (2-oxo-2-(2-(4-(trifluoromethyl)benzoyl)hydrazineyl)ethyl) carbamate (23a)

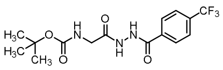

To a solution of *N*-Boc-glycine hydrazide (100 mg, 0.529 mmol, 1.0 eq.) in MeCN (0.53 mL) was added CDI (103 mg, 0.635 mmol, 1.2 eq.). The mixture was stirred for 15 min at RT, then 4-trifluoromethylbenzoic acid (101 mg, 0.529 mmol, 1.0 eq.) was added and the reaction stirred for 19 h at RT. The reaction mixture was partitioned between EtOAc (20 mL) and a hydrochloric acid solution (1 M aq., 20 mL). The organic layer was separated, and the aqueous layer was extracted using EtOAc (2 × 20 mL). The combined organic layers were washed with water (20 mL), brine (20 mL), dried over sodium sulfate, filtered, and concentrated *in vacuo*. The resulting residue was purified using flash column chromatography (silica gel, pentane/EtOAc 2:1 → 1:2) to yield *tert*-butyl (2-oxo-2-(2-(4-(trifluoromethyl)benzoyl)hydrazineyl)ethyl) carbamate (**23a**, 40.0 mg, 0.111 mmol, 21%) as a colorless solid.

Analytical Data of *tert*-Butyl (2-oxo-2-(2-(4-(trifluoromethyl)benzoyl)hydrazineyl)ethyl) carbamate (23a)

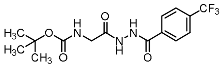

^{1}H NMR (500 MHz, methanol-*d4*) δ 8.09 – 7.99 (m, 2H), 7.83 – 7.76 (m, 2H), 3.88 (s, 2H), 1.46 (s, 9H) ppm;

^{13}C NMR (126 MHz, methanol-*d4*) δ 171.8, 167.8, 158.4, 137.3, 134.6 (q, J = 32.6 Hz), 129.5, 126.6 (q, J = 3.9 Hz), 125.2 (q, J = 271.5 Hz), 80.8, 43.3, 28.7 ppm;

^{19}F NMR (471 MHz, methanol-*d4*) δ -64.5 ppm;

IR (thin film): ν 3398w, 3271m, 2986w, 1711w, 1687s, 1612s, 1574m, 1516s, 1480m, 1427w, 1411w, 1392w, 1383w, 1369w, 1333m, 1321m, 1287m, 1255w, 1231m, 1200w, 1169s, 1119s, 1092w, 1067m, 1050w, 1031w, 1018m, 947w, 877w, 858m, 790w, 766w, 705w, 588m, 537m, 499m, 463w, 412w cm^{-1};

HRMS (ESI) for $C_{15}H_{18}O_4N_3F_3Na^+$ $[M+Na]^+$: calculated: 384.11416; found: 384.11372.

ERJ-623

Preparation of *tert*-Butyl ((5-(4-(trifluoromethyl)phenyl)-1,3,4-oxadiazol-2-yl)methyl) carbamate (23b)

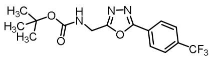

In a flame-dried microwave vial, *tert*-butyl (2-oxo-2-(2-(4-(trifluoromethyl)benzoyl) hydrazineyl)ethyl) carbamate (**23a**, 25.0 mg, 69.2 µmol, 1.0 eq.) was dissolved in dry DCM (0.44 mL). Triethylamine (29 µL, 0.21 mmol, 3.0 eq.) was added followed by *p*-toluenesulfonyl chloride (15.8 mg, 83.0 µmol, 1.2 eq.). The reaction mixture was stirred at RT for 1 h. The reaction mixture was partioned between EtOAc (20 mL) and an ammonium chloride solution (sat. aq., 20 mL). The organic layer was separated, and the aqueous layer was extracted with EtOAc (2 × 10 mL). The combined organic layers were washed with a sodium bicarbonate solution (sat. aq., 20 mL), water (20 mL), brine (20 mL), dried over sodium sulfate, filtered, and concentrated *in vacuo* to yield *tert*-butyl ((5-(4-(trifluoromethyl)phenyl)-1,3,4-oxadiazol-2-yl)methyl)carbamate (**23b**, 22.3 mg, 65.0 µmol, 94%) as a colorless solid.

Analytical Data of Preparation of *tert*-Butyl ((5-(4-(trifluoromethyl)phenyl)-1,3,4-oxadiazol-2-yl)methyl) carbamate (23b)

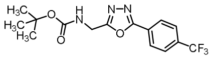

^{1}H NMR (500 MHz, $CDCl_3$) δ 8.19 – 8.14 (m, 2H), 7.80 – 7.74 (m, 2H), 5.24 (br. s, 1H), 4.66 (d, J = 6.0 Hz, 2H), 1.48 (s, 9H) ppm;

^{13}C NMR (126 MHz, $CDCl_3$) δ 164.7, 164.3, 155.6, 133.7 (q, J = 32.9 Hz), 127.4, 127.0, 126.3 (q, J = 3.7 Hz), 123.7 (q, J = 272.6 Hz), 80.9, 36.1, 28.4 ppm;

^{19}F NMR (471 MHz, $CDCl_3$) δ -63.1 ppm;

IR (thin film): ν 3340w, 2980w, 2932w, 1705m, 1562w, 1516m, 1507m, 1455w, 1420m, 1393w, 1368m, 1324s, 1282m, 1252m, 1218w, 1168s, 1129s, 1088m, 1066m, 1032w, 1016m, 937w, 851m, 781w, 764w, 750w, 707w, 659w, 633w, 597w, 469w cm^{-1};

HRMS (ESI) for $C_{15}H_{16}O_3N_3F_3Na^+$ $[M+Na]^+$: calculated: 366.10360; found: 366.10352.

ERJ-626

1.3.5 Anilines and Aminopyridines

Preparation of Aniline 24

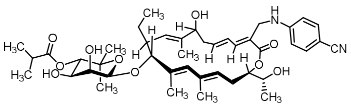

To an HPLC vial was added fidaxomicin (**1**, 100 mg, 94.5 µmol, 1.0 eq.), 4-aminobenzonitrile (33.5 mg, 0.284 mmol, 3.0 eq.), [Pd(cinnamyl)Cl]$_2$ (2.5 mg, 4.7 µmol, 5 mol%) and dppf (5.2 mg, 9.5 µmol, 10 mol%). The vial was capped, evacuated and backfilled with nitrogen three times, and then dry THF (0.5 mL) was added. The reaction was heated under stirring to 60 °C. Reaction progress was monitored by UHPLC-MS. After 6 h the reaction mixture was cooled to RT, diluted with THF (1 mL), 3-mercaptopropyl-functionalised silica was added and the mixture was stirred at RT until no Pd-complexes were observed by UHPLC-MS. The mixture was filtered through a pad of celite, eluted with DCM (2 mL) and concentrated *in vacuo*. The resulting residue was dissolved in MeCN (1 mL) and eluted through a C18 SPE cartridge with MeCN (8 mL). The filtrate was concentrated *in vacuo* and the resulting crude mixture purified by preparative RP-HPLC ([Gemini NX, C18, 5 µ, 110 Å, 250 mm × 21.2 mm, solvent A: H_2O + 0.1% HCOOH, solvent B: MeCN + 0.1% HCOOH, 18 mL/min; LC time program (min – %B): 7 min – 35%, 60 min – 55%]) to yield, after concentration *in vacuo*, the 2-aminopyridine **24** (t_R = 48.8 min, 15.9 mg, 20.8 µmol, 22%) as an off-white solid.

Analytical Data of Aniline 24

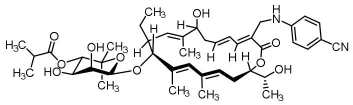

^{1}H NMR (500 MHz, acetone-d_6) δ 7.45 – 7.38 (m, 2H), 7.19 (d, J = 11.5 Hz, 1H), 6.80 – 6.72 (m, 2H), 6.71 – 6.60 (m, 1H), 5.96 (ddd, J = 14.6, 9.6, 4.6 Hz, 1H), 5.90 (t, J = 5.6 Hz, 1H), 5.81 (s, 1H), 5.62 – 5.56 (m, 1H), 5.23 – 5.17 (m, 1H), 4.99 (d, J = 10.1 Hz, 1H), 4.77 (s, 1H), 4.70 (q, J = 5.4 Hz, 1H), 4.30 – 4.24 (m, 1H), 4.19 – 4.04 (m, 2H), 4.03 – 3.97 (m, 1H), 3.97 – 3.94 (m, 1H), 3.81 (d, J = 3.7 Hz, 1H), 3.76 – 3.69 (m, 3H), 3.25 (d, J = 9.4 Hz, 1H), 2.79 – 2.59 (m, 3H), 2.59 – 2.53 (m, 1H), 2.52 – 2.46 (m, 1H), 2.41 (ddd, J = 13.8, 8.9, 4.4 Hz, 1H), 1.98 – 1.91 (m, 1H), 1.79 (s, 3H), 1.71 (s, 3H), 1.65 (s, 3H), 1.26 (ddd, J = 21.4, 10.2, 4.8 Hz, 1H), 1.16 – 1.11 (m, 13H), 1.08 (s, 3H), 0.83 (t, J = 7.4 Hz, 3H) ppm;

^{13}C NMR (126 MHz, acetone-d_6) δ 176.8, 168.2, 152.9, 144.2, 143.4, 136.9, 136.2, 136.1, 134.1, 133.8, 127.9, 126.5, 125.9, 124.1, 120.9, 113.3, 98.6, 96.8, 93.3, 78.4, 75.7, 73.7, 72.9, 72.8, 70.2, 67.7, 42.1, 39.5, 37.2, 34.8, 28.7, 28.3, 26.5, 20.6, 19.4, 19.2, 18.6, 17.4, 15.2, 13.8, 11.2 ppm;

IR (thin film): ν 3428m, 2975m, 2934m, 2876w, 2213m, 1696m, 1640w, 1607s, 1523m, 1469w, 1387m, 1337m, 1307w, 1241m, 1200m, 1173m, 1151m, 1075s, 1035s, 894w, 827w, 798w, 546w, 461w cm^{-1};

HRMS (ESI) for $C_{43}H_{61}O_{10}N_2^+$ $[M+H]^+$: calculated: 765.43207; found: 765.43115.

ERJ-255, ERJ-615

Preparation of Aminopyridine 25

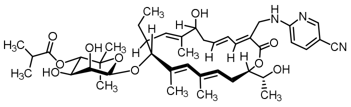

To an HPLC vial was added fidaxomicin (**1**, 100 mg, 94.5 µmol, 1.0 eq.), 2-amino-5-cyanopyridine (33.8 mg, 0.284 mmol, 3.0 eq.), [Pd(cinnamyl)Cl]$_2$ (9.8 mg, 19 µmol, 20 mol%) and dppf (21.0 mg, 37.8 µmol, 40 mol%). The vial was capped, evacuated and backfilled with nitrogen three times and then dry THF (0.5 mL) was added. The reaction was heated under stirring to 60 °C. Reaction progress was monitored by UHPLC-MS. After 16 h the reaction mixture was cooled to RT, diluted with THF (1 mL), 3-mercaptopropyl-functionalised silica was added and the mixture was stirred at RT until no Pd-complexes were observed by UHPLC-MS. The mixture was filtered through a pad of celite, eluted with THF (2 mL) and concentrated *in vacuo*. The resulting residue was dissolved in MeCN (1 mL) and eluted through a C18 SPE cartridge with MeCN (8 mL). The filtrate was concentrated *in vacuo* and the resulting crude mixture purified by preparative RP-HPLC ([Gemini NX, C18, 5 µ, 110 Å, 250 mm × 21.2 mm, solvent A: H_2O + 0.1% HCOOH, solvent B: MeCN + 0.1% HCOOH, 20 mL/min; LC time program (min – %B): 7 min – 20%, 60 min – 35%]) to yield, after concentration *in vacuo*, the 2-aminopyridine **25** (t_R = 65.9 min, 23.1 mg, 30.2 µmol, 32%) as a light brown solid.

Analytical Data of Aminopyridine 25

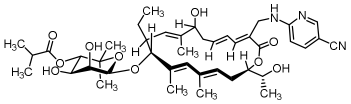

^{1}H NMR (500 MHz, acetone-*d$_6$*) δ 8.38 (d, *J* = 2.2 Hz, 1H), 7.61 (dd, *J* = 8.9, 2.3 Hz, 1H), 7.15 (d, *J* = 11.4 Hz, 1H), 6.84 – 6.72 (m, 2H), 6.68 (d, *J* = 8.8 Hz, 1H), 5.91 (ddd, *J* = 14.7, 9.7, 4.6 Hz, 1H), 5.80 (s, 1H), 5.59 (t, *J* = 8.3 Hz, 1H), 5.19 (dt, *J* = 10.6, 1.6 Hz, 1H), 4.99 (d, *J* = 10.1 Hz, 1H), 4.76 (d, *J* = 1.2 Hz, 1H), 4.69 (q, *J* = 5.5 Hz, 1H), 4.41 – 4.35 (m, 1H), 4.33 – 4.28 (m, 1H), 4.27 – 4.25 (m, 1H), 4.05 – 3.99 (m, 1H), 3.95 (d, *J* = 3.2 Hz, 1H), 3.75 – 3.68 (m, 2H), 3.29 (s, 1H), 2.84 (s, 2H), 2.79 – 2.70 (m, 1H), 2.70 – 2.60 (m, 2H), 2.60 – 2.45 (m, 2H), 2.40 (ddd, *J* = 13.9, 8.9, 4.4 Hz, 1H), 1.98 – 1.90 (m, 1H), 1.79 (d, *J* = 1.3 Hz, 3H), 1.68 (s, 3H), 1.65 (s, 3H), 1.30 – 1.19 (m, 1H), 1.16 – 1.11 (m, 12H), 1.08 (s, 3H), 0.82 (t, *J* = 7.4 Hz, 3H) ppm;

^{13}C NMR (126 MHz, acetone-*d$_6$*) δ 176.8, 168.4, 160.8, 153.7, 144.0, 142.5, 139.5, 136.9, 136.2, 136.0, 133.8, 128.7, 126.4, 126.0, 124.0, 119.2, 109.5, 96.9, 96.7, 93.3, 78.4, 75.7, 73.7, 72.9, 72.8, 70.1, 67.6, 42.0, 37.6, 37.3, 34.8, 28.7, 28.2, 26.5, 20.6, 19.4, 19.2, 18.6, 17.3, 15.2, 13.8, 11.2 ppm;

IR (thin film): ν 2975m, 2934m, 2876w, 2219w, 1732m, 1693m, 1641w, 1606s, 1517m, 1469w, 1386m, 1369m, 1299m, 1251m, 1211m, 1149m, 1074s, 1034s, 898w, 829w, 797w, 548w, 528w, 513w, 471w cm^{-1};

HRMS (ESI) for $C_{42}H_{60}O_{10}N_3^+$ $[M+H]^+$: calculated: 766.42732; found: 766.42662.

ERJ-254

1.3.6 Amide-linked Derivatives

Preparation of Amine 27

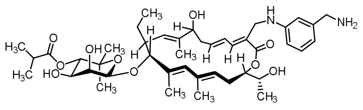

To an HPLC vial was added fidaxomicin (**1**, 400 mg, 0.378 mmol, 1.0 eq.), 3-(aminomethyl)anilinium methylsulfonate (0.9 eq. MsOH, 237 mg, 1.13 mmol, 3.0 eq.), $[Pd(cinnamyl)Cl]_2$ (19.6 mg, 37.8 µmol, 10 mol%) and dppf (43.2 mg, 75.6 µmol, 20 mol%). The vial was capped, evacuated and backfilled with nitrogen three times, and then dry THF (2.0 mL) was added. The reaction was heated under stirring to 60 °C. Reaction progress was monitored by UHPLC-MS. After 22 h the reaction mixture was cooled to RT, diluted with THF (2 mL), 3-mercaptopropyl-functionalised silica was added and the mixture was stirred at RT until no Pd-complexes were observed by UHPLC-MS. The mixture was filtered through a pad of celite, eluted with DCM (2 mL) and concentrated *in vacuo*. The resulting residue was dissolved in MeCN (3 mL) and eluted through a C18 SPE cartridge with MeCN (15 mL). The filtrate was concentrated *in vacuo* and the resulting crude mixture purified by preparative RP-HPLC ([Gemini NX, C18, 5 µ, 110 Å, 250 mm × 21.2 mm, solvent A: H_2O + 0.1% HCOOH, solvent B: MeCN + 0.1% HCOOH, 20 mL/min; LC time program (min – %B): 7 min – 15%, 60 min – 40%]) to yield, after concentration *in vacuo* the formate salt of the amine **27**. The salt was dissolved in DCM (5 mL) and washed with a sodium bicarbonate solution (sat. aq., 2 × 5 mL). The organic layer was dried over sodium sulfate, filtered, and concentrated *in vacuo* to yield the amine **27** (t_R = 27.6 min, 28.0 mg, 36.4 µmol, 10%) as an off-white solid.

Note: The use of 0.9 eq. MsOH in the preparation of the nucleophile salt is crucial since free benzylamine is required for catalyst activation.

Note: If the formate salt is not freebased, subsequent amide coupling partially leads to formylation of the primary amine.

Analytical Data of Amine 27

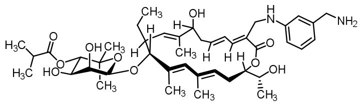

^{1}H NMR (500 MHz, $CDCl_3$) δ 7.13 – 7.09 (m, 1H), 7.06 (d, *J* = 11.9 Hz, 1H), 6.66 – 6.59 (m, 3H), 6.53 (d, *J* = 8.7 Hz, 1H), 5.85 – 5.75 (m, 2H), 5.43 (t, *J* = 8.2 Hz, 1H), 5.30 (d, *J* = 1.2 Hz, 1H), 5.02 – 4.92 (m, 2H), 4.68 (td, *J* = 8.0, 3.9 Hz, 1H), 4.64 (s, 1H), 4.24 (s, 1H), 4.12 – 3.95 (m, 4H), 3.78 (s, 2H), 3.70 – 3.58 (m, 2H), 2.70 (tdd, *J* = 20.5, 10.7, 6.5 Hz, 3H), 2.64 – 2.56 (m, 1H), 2.52 (ddd, *J* = 14.8, 10.6, 4.7 Hz, 1H), 2.30 – 2.19 (m, 1H), 1.89 (tt, *J* = 7.8, 3.8 Hz, 1H), 1.84 (s, 3H), 1.78 (s, 3H), 1.66 (s, 3H), 1.31 – 1.24 (m, 1H), 1.21 – 1.12 (m, 12H), 1.09 (s, 3H), 0.84 (s, 3H) ppm;

^{13}C NMR (126 MHz, $CDCl_3$) δ 177.3, 169.1, 148.3, 142.5, 140.3, 136.7, 136.2, 134.4, 134.3, 129.6, 128.9, 127.8, 126.8, 123.2, 117.2, 112.9, 112.6, 110.1, 94.7, 92.5, 79.3, 74.8, 73.5, 72.6, 71.5, 70.2, 68.7, 46.2, 41.8, 40.5, 37.0, 34.3, 28.3, 28.2, 25.9, 19.2, 18.9, 18.6, 18.5, 17.1, 15.4, 13.8, 11.1 ppm;

IR (thin film): ν 3388m, 2974m, 2931m, 2874m, 1733m, 1691s, 1640m, 1607m, 1469m, 1384m, 1344m, 1298m, 1258m, 1208m, 1151s, 1079s, 1034s, 1007m, 899m, 774w, 734w, 698w, 514w cm^{-1};

HRMS (ESI) for $C_{43}H_{65}O_{10}N_2^+$ $[M+H]^+$: calculated: 769.46337; found: 769.46407.

ERJ-458

Preparation of Amide 28

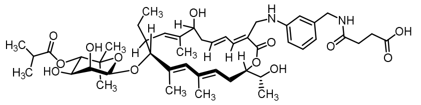

The amine **27** (3.8 mg, 4.9 µmol, 1.0 eq.) was dissolved in $CHCl_3$ (0.1 mL). Succinic anhydride (0.5 mg, 5.4 µmol, 1.1 eq.) was added and the reaction mixture was stirred at RT. Full conversion was observed after 1 min and the reaction mixture was evaporated under a stream of nitrogen. The residue was dissolved in MeCN (0.5 mL) and eluted through a C18 SPE cartridge with MeCN (4 mL). The filtrate was concentrated *in vacuo* and the resulting mixture purified by preparative RP-HPLC ([Gemini NX, C18, 5 µ, 110 Å, 250 mm × 21.2 mm, solvent A: H_2O + 0.1% HCOOH, solvent B: MeCN + 0.1% HCOOH, 20 mL/min; LC time program (min – %B): 7 min – 30%, 60 min – 50%]) to yield the amide **28** (t_R = 23.6 min, 3.0 mg, 3.5 µmol, 70%) as an off-white solid

Analytical Data of Amide 28

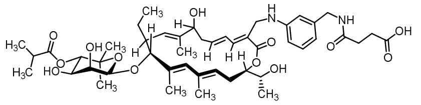

^{1}H NMR (500 MHz, $CDCl_3$) δ 7.13 – 7.02 (m, 2H), 6.66 – 6.52 (m, 4H), 6.34 – 6.28 (m, 1H), 5.83 (s, 1H), 5.81 – 5.73 (m, 1H), 5.41 (t, *J* = 7.9 Hz, 1H), 4.98 (d, *J* = 9.8 Hz, 1H), 4.94 (d, *J* = 10.9 Hz, 1H), 4.70 (d, *J* = 9.7 Hz, 1H), 4.64 (s, 1H), 4.46 – 4.29 (m, 2H), 4.28 (s, 1H), 4.07 – 4.00 (m, 3H), 3.69 – 3.57 (m, 2H), 2.76 – 2.45 (m, 11H), 2.22 (d, *J* = 14.7 Hz, 1H), 1.90 (d, *J* = 12.5 Hz, 1H), 1.86 (s, 3H), 1.78 (s, 3H), 1.66 (s, 3H), 1.16 (dd, *J* = 20.5, 8.4 Hz, 13H), 1.08 (s, 3H), 0.82 (t, *J* = 7.5 Hz, 3H) ppm;

^{13}C NMR (126 MHz, $CDCl_3$) δ 177.3, 174.7, 172.3, 169.5, 148.2, 142.8, 140.1, 139.3, 136.4, 136.3, 134.4, 134.4, 129.5, 129.3, 127.9, 126.4, 123.3, 117.8, 113.4, 112.9, 94.7, 92.4, 79.4, 74.8, 73.5, 72.7, 71.6, 70.2, 69.0, 43.8, 41.9, 40.6, 36.8, 34.3, 31.0, 29.7, 28.4, 28.1, 25.8, 19.2, 18.9, 18.5, 18.1, 17.0, 15.4, 13.8, 11.1 ppm;

IR (thin film): ν 3373w, 2975m, 2928m, 2875w, 1693s, 1642m, 1607m, 1545w, 1491w, 1469w, 1430m, 1385m, 1369m, 1347m, 1299m, 1260s, 1233s, 1209s, 1151s, 1078s, 1032s, 951w, 898m, 796w, 770w, 733m, 699m, 635w, 584w, 510w, 499w, 486w, 461w, 442w, 432w, 425w, 415w, 409w cm^{-1};

HRMS (ESI) for $C_{47}H_{69}O_{13}N_2^+$ $[M+H]^+$: calculated: 869.47942; found: 869.47986.

ERJ-462

Preparation of Amide 29

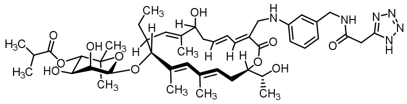

In an HPLC vial, 2-(1*H*-tetrazol-5-yl)acetic acid (0.9 mg, 6.9 µmol, 1.1 eq.) was dissolved in DMF (50 µL). DIPEA (2.2 µL, 13 µmol, 2.0 eq.) was added, followed by HATU (3.6 mg, 9.4 µmol, 1.5 eq.) and the mixture was stirred at RT for 5 min. Then, a solution of amine **27** (4.8 mg, 6.2 µmol, 1.0 eq.) in DMF (50 µL) was added dropwise *via* microliter syringe. Full conversion was observed after 5 min and the reaction mixture was partitioned between EtOAc (2 mL) and water (1 mL). The organic layer was separated, and the aqueous layer was extracted with EtOAc (3 × 1 mL). The combined organic layers were washed with brine (3 mL), dried over sodium sulfate, filtered, and concentrated *in vacuo*. The resulting residue was dissolved in MeCN (0.5 mL) and eluted through a C18 SPE cartridge with MeCN (6 mL). The filtrate was concentrated *in vacuo* and the resulting mixture purified by preparative RP-HPLC ([Gemini NX, C18, 5 µ, 110 Å, 250 mm × 21.2 mm, solvent A: H_2O + 0.1% HCOOH, solvent B: MeCN + 0.1% HCOOH, 20 mL/min; LC time program (min – %B): 7 min – 30%, 60 min – 50%]) to yield the amide **29** (t_R = 24.9 min, 2.6 mg, 3.0 µmol, 47%) as an off-white solid.

Analytical Data of Amide 29

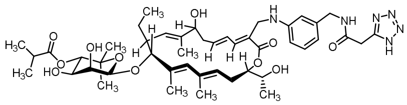

^{1}H NMR (500 MHz, acetone-d_6) δ 7.93 (br. s, 1H), 7.15 (d, J = 11.5 Hz, 1H), 7.02 (t, J = 7.8 Hz, 1H), 6.70 – 6.61 (m, 2H), 6.55 (t, J = 8.2 Hz, 1H), 5.91 (ddd, J = 14.7, 9.6, 4.6 Hz, 1H), 5.81 (s, 1H), 5.59 (t, J = 8.3 Hz, 1H), 5.20 (d, J = 10.5 Hz, 1H), 4.99 (d, J = 10.0 Hz, 1H), 4.76 (s, 1H), 4.70 (q, J = 5.6 Hz, 1H), 4.35 – 4.23 (m, 3H), 4.09 (s, 2H), 4.08 – 3.99 (m, 2H), 3.99 – 3.93 (m, 2H), 3.77 – 3.65 (m, 2H), 2.82 – 2.61 (m, 3H), 2.60 – 2.46 (m, 2H), 2.39 (ddd, J = 13.9, 8.9, 4.5 Hz, 1H), 1.93 (dt, J = 10.8, 2.8 Hz, 1H), 1.79 (s, 3H), 1.68 (s, 3H), 1.65 (s, 3H), 1.23 (dq, J = 15.8, 7.7 Hz, 1H), 1.17 – 1.11 (m, 13H), 1.08 (s, 3H), 0.82 (t, J = 7.4 Hz, 3H) ppm;

^{13}C NMR (126 MHz, acetone-d_6) δ 176.8, 168.6, 167.0, 149.8, 143.7, 142.6, 140.3, 136.8, 136.3, 136.0, 133.9, 129.8, 128.2, 126.9, 126.5, 124.0, 116.9, 112.9, 112.8, 96.7, 93.3, 78.3, 75.7, 73.8, 72.9, 72.8, 70.1, 67.7, 44.3, 44.2, 42.1, 40.4, 37.2, 29.2, 28.7, 28.2, 26.5, 20.6, 19.4, 19.2, 18.6, 17.4, 15.2, 13.8, 11.2 ppm;

Note: One quaternary carbon was not observed or overlaps with another one.

IR (thin film): ν 3358w, 2922s, 2852m, 1732m, 1675m, 1607m, 1548w, 1470w, 1384m, 1260m, 1233m, 1209m, 1148m, 1077s, 1032s, 898w, 797w, 765m, 750m, 700w, 524w, 424w, 409w cm^{-1};

HRMS (ESI) for $C_{46}H_{67}O_{11}N_6^+$ $[M+H]^+$: calculated: 879.48623; found: 879.48711.

ERJ-465

Preparation of Amide 30

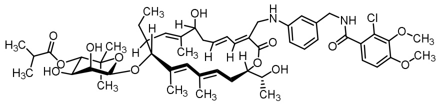

In an HPLC vial, 2-chloro-3,4-dimethoxybenzoic acid (2.9 mg, 13 µmol, 1.0 eq.) was dissolved in DMF (0.1 mL). DIPEA (4.6 µL, 26.6 µmol, 2.0 eq.) was added, followed by HATU (7.6 mg, 13 µmol, 1.5 eq.) and the mixture was stirred at RT for 5 min. Then, a solution of amine **27** (10.2 mg, 13.3 µmol, 1.0 eq.) in DMF (0.1 mL) was added dropwise *via* microliter syringe. Full conversion was observed after 1 min and the reaction mixture was partitioned between EtOAc (2 mL) and water (1 mL). The organic layer was separated, and the aqueous layer was extracted with EtOAc (3 × 1 mL). The combined organic layers were washed with brine (3 mL), dried over sodium sulfate, filtered, and concentrated *in vacuo*. The resulting residue was dissolved in MeCN (0.5 mL) and eluted through a C18 SPE cartridge with MeCN (6 mL). The filtrate was concentrated *in vacuo* and the resulting mixture purified by preparative RP-HPLC ([Gemini NX, C18, 5 µ, 110 Å, 250 mm × 21.2 mm, solvent A: H_2O + 0.1% HCOOH, solvent B: MeCN + 0.1% HCOOH, 20 mL/min; LC time program (min – %B): 7 min – 40%, 60 min – 70%]) to yield the amide **30** (t_R = 28.8 min, 3.2 mg, 3.3 µmol, 25%) as an off-white solid.

Analytical Data of Amide 30

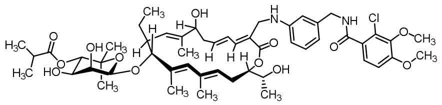

^{1}H NMR (500 MHz, $CDCl_3$) δ 7.52 – 7.45 (m, 1H), 7.18 – 7.10 (m, 1H), 7.09 – 7.04 (m, 1H), 6.90 – 6.84 (m, 1H), 6.72 – 6.62 (m, 2H), 6.59 – 6.54 (m, 1H), 5.84 – 5.73 (m, 2H), 5.43 (t, *J* = 8.1 Hz, 1H), 5.30 (s, 1H), 5.02 – 4.95 (m, 2H), 4.71 – 4.60 (m, 3H), 4.47 (dd, *J* = 14.7, 5.1 Hz, 1H), 4.23 (s, 1H), 4.14 – 3.93 (m, 4H), 3.90 (s, 3H), 3.85 (s, 3H), 3.69 – 3.59 (m, 2H), 2.76 – 2.55 (m, 4H), 2.49 (ddd, *J* = 14.9, 10.7, 4.7 Hz, 1H), 2.24 (dt, *J* = 11.5, 4.7 Hz, 1H), 1.88 (dd, *J* = 13.8, 6.7 Hz, 1H), 1.78 (s, 6H), 1.64 (s, 3H), 1.29 – 1.05 (m, 16H), 0.82 (t, *J* = 7.4 Hz, 3H) ppm;

^{13}C NMR (126 MHz, $CDCl_3$) δ 177.3, 169.1, 166.3, 155.5, 148.1, 145.7, 142.9, 140.8, 139.0, 136.4, 136.3, 134.3, 129.6, 128.7, 128.2, 127.4, 126.1, 126.0, 125.8, 123.2, 117.7, 113.8, 112.8, 110.6, 94.7, 92.5, 79.3, 74.9, 73.5, 72.6, 71.6, 70.2, 68.8, 60.8, 56.3, 44.6, 41.8, 40.6, 37.0, 34.3, 29.8, 28.4, 28.1, 25.9, 19.2, 18.9, 18.6, 18.5, 17.0, 15.4, 13.8, 11.1 ppm;

IR (thin film): ν 3393w, 2974w, 2932w, 2874w, 1733m, 1689m, 1642m, 1606m, 1592m, 1528w, 1487m, 1456m, 1402m, 1385m, 1368m, 1295m, 1268m, 1230m, 1209m, 1149m, 1072m, 1036s, 995m, 950w, 900w, 796w, 775w, 735m, 699w, 670w, 514w cm^{-1};

HRMS (ESI) for $C_{52}H_{72}O_{13}N_2Cl^+$ $[M+H]^+$: calculated: 967.47174; found: 967.47237.

ERJ-404

Preparation of Amide 31

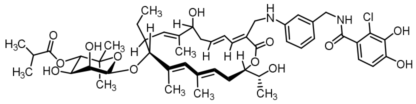

In an HPLC vial, 3,4-bis(allyloxy)-2-chlorobenzoic acid (**31b**, 0.9 mg, 6.9 µmol, 1.1 eq.) was dissolved in DMF (100 µL). DIPEA (6.3 µL, 36 µmol, 2.0 eq.) was added, followed by HATU (10.4 mg, 27.3 µmol, 1.5 eq.) and the mixture was stirred at RT for 5 min. Then, a solution of amine **27** (14.0 mg, 18.2 µmol, 1.0 eq.) in DMF (100 µL) was added dropwise *via* microliter syringe. After 30 min the reaction mixture was partitioned between EtOAc (2 mL) and water (1 mL). The organic layer was separated, and the aqueous layer was extracted with EtOAc (3 × 1 mL). The combined organic layers were washed with a lithium chloride solution (5% aq., 2 mL), brine (3 mL), dried over sodium sulfate, filtered, and concentrated *in vacuo*.

To the residue was added $Pd(PPh_3)_4$ (4.2 mg, 3.6 µL, 20 mol%) and the reaction vial was placed under vacuum and refilled with nitrogen (repeated three times). Dry THF (100 µL) was added, and the mixture cooled to 0 °C. Morpholine (3.5 µL, 40 µmol, 2.2 eq.) was added and after 20 min full conversion was reached. The reaction mixture was diluted with THF (1 mL), and 3-mercaptopropyl-functionalized silica gel was added. After stirring at RT, the mixture was filtered over celite and washed with DCM (5 mL). The filtrate was concentrated *in vacuo*, dissolved in MeCN (0.5 mL) and eluted through a C18 SPE cartridge with MeCN (4 mL). The filtrate was concentrated *in vacuo* and purified by preparative RP-HPLC ([Gemini NX, C18, 5 µ, 110 Å, 250 mm × 21.2 mm, solvent A: H_2O + 0.1% HCOOH, solvent B: MeCN + 0.1% HCOOH, 20 mL/min; LC time program (min – %B): 7 min – 30%, 60 min – 55%]) and lyophilized to yield the amide **31** (t_R = 31.3 min, 8.5 mg, 9.1 µmol, 50% *over 2 steps*) was obtained as a light-brown solid.

Analytical Data of Amide 31

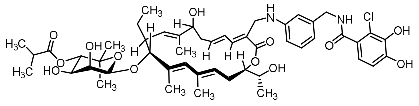

^{1}H NMR (500 MHz, acetone-d_6) δ 7.15 (d, J = 11.5 Hz, 1H), 7.04 (t, J = 7.7 Hz, 1H), 6.96 (d, J = 8.1 Hz, 1H), 6.82 (d, J = 8.2 Hz, 1H), 6.73 (s, 1H), 6.65 (t, J = 9.9 Hz, 2H), 6.56 (d, J = 8.2 Hz, 1H), 5.90 (ddd, J = 14.7, 9.7, 4.5 Hz, 1H), 5.80 (s, 1H), 5.64 – 5.57 (m, 2H), 5.19 (d, J = 10.6 Hz, 1H), 4.99 (d, J = 10.1 Hz, 1H), 4.76 (s, 1H), 4.69 (q, J = 5.5 Hz, 1H), 4.46 (qd, J = 14.9, 5.6 Hz, 2H), 4.24 (s, 1H), 4.08 (d, J = 13.4 Hz, 1H), 4.02 (d, J = 6.0 Hz, 2H), 3.97 – 3.91 (m, 2H), 3.87 – 3.78 (m, 4H), 3.76 – 3.67 (m, 1H), 2.74 (dt, J = 13.7, 6.8 Hz, 1H), 2.68 – 2.60 (m, 1H), 2.56 (hept, J = 7.0, 6.3 Hz, 1H), 2.52 – 2.44 (m, 1H), 2.38 (ddd, J = 13.8, 8.9, 4.4 Hz, 1H), 2.00 – 1.91 (m, 1H), 1.79 (s, 3H), 1.66 (s, 3H), 1.64 (s, 3H), 1.13 (d, J = 8.3 Hz, 13H), 1.08 (s, 3H), 0.83 (t, J = 7.6 Hz, 3H) ppm;

^{13}C NMR (126 MHz, acetone-d_6) δ 176.8, 168.5, 167.4, 149.8, 148.1, 143.6, 142.6, 141.0, 136.8, 136.3, 135.9, 133.9, 129.7, 128.2, 126.9, 126.5, 124.0, 120.8, 119.0, 116.9, 114.2, 112.9, 112.7, 96.7, 93.3, 78.3, 75.7, 73.8, 72.8, 70.1, 67.6, 49.8, 44.2, 44.1, 42.1, 40.5, 37.2, 34.8, 32.6, 28.7, 28.2, 26.5, 23.3, 20.6, 19.4, 19.2, 18.6, 17.3, 15.2, 14.4, 13.8, 11.2 ppm;

Note: The compound should be stored cold (-20 °C) and under nitrogen to avoid decomposition due to the sensitive catechol moiety. Extra signals are observed in the NMR spectra due to the presence of decomposition by-products and grease.

HRMS (ESI) for $C_{50}H_{68}O_{13}N_2Cl^+$ $[M+H]^+$: calculated: 939.44044; found: 939.44106.

ERJ-419, ERJ-420

Preparation of Allyl 3,4-bis(allyloxy)-2-chlorobenzoate (31a)

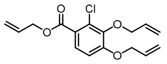

In a microwave vial, to a suspension of 2-chloro-3,4-dihydroxybenzoic acid (90% purity, 100 mg, 0.477 mmol, 1.0 eq.) and potassium carbonate (264 mg, 1.91 mmol, 4.0 eq.) was added dry acetone (1.2 mL). The vial was capped, and allyl bromide (0.17 mL, 1.9 mmol, 4.0 eq.) was added. The reaction mixture was stirred at 60 °C for 19 h. The reaction was allowed to cool to RT and was then partitioned between EtOAc (20 mL) and a hydrochloric acid solution (1 M aq., 10 mL). The organic layer was separated, and the aqueous layer was extracted with EtOAc (2 × 10 mL). The combined organic layers were washed with a sodium bicarbonate solution (sat. aq., 10 mL), brine (10 mL), dried over sodium sulfate, and concentrated *in vacuo*. The resulting residue was purified *via* flash column chromatography (silica gel, pentane/EtOAc 20:1) to yield allyl 3,4-bis(allyloxy)-2-chlorobenzoate (**31a**, 96 mg, 0.31 mmol, 65%) as a colorless oil.

Analytical Data of Allyl 3,4-bis(allyloxy)-2-chlorobenzoate (31a)

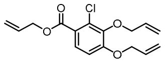

^{1}H NMR (500 MHz, $CDCl_3$) δ 7.68 – 7.63 (m, 1H), 6.85 – 6.80 (m, 1H), 6.13 (ddt, *J* = 16.7, 10.1, 6.0 Hz, 1H), 6.09 – 5.96 (m, 2H), 5.49 – 5.20 (m, 6H), 4.81 (dd, *J* = 5.6, 1.5 Hz, 2H), 4.66 – 4.61 (m, 2H), 4.56 (dd, *J* = 6.1, 1.4 Hz, 2H) ppm;

^{13}C NMR (126 MHz, $CDCl_3$) δ 165.1, 155.8, 145.4, 133.7, 132.3, 132.2, 129.9, 127.6, 123.0, 118.6, 118.6, 118.4, 111.1, 74.3, 69.8, 66.0 ppm;

IR (thin film): ν 3085w, 3020w, 2985w, 2938w, 2878w, 1725m, 1649w, 1586m, 1484m, 1454m, 1410m, 1358m, 1274s, 1262s, 1203m, 1166m, 1138m, 1099m, 1007s, 992s, 924s, 868w, 819m, 775m, 742m, 704w, 648w, 605w, 554w, 449w, 405w cm^{-1};

HRMS (ESI) for $C_{16}H_{18}O_4Cl^+$ $[M+H]^+$: calculated: 309.08881; found: 309.08899.

ERJ-398, ERJ-402, ERJ-618

Preparation of 3,4-Bis(allyloxy)-2-chlorobenzoic Acid (31b)

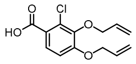

In a microwave vial, to a solution of 3,4-bis(allyloxy)-2-chlorobenzoate (**31a**, 74.8 mg, 0.242 mmol, 1.0 eq.) in THF (0.7 mL) was added a sodium hydroxide solution (1 M, 0.7 mL, 0.73 mmol, 3.0 eq.). The vial was capped, and the stirred reaction mixture was heated to 60 °C overnight. The reaction mixture was partitioned between a hydrochloric acid solution (1 M, 10 mL) and EtOAc (20 mL). The organic layer was separated, and the aqueous layer was extracted with EtOAc (3 × 10 mL). The combined organic layers were washed with brine (20 mL), dried over sodium sulfate, filtered, and concentrated *in vacuo* to yield 3,4-bis(allyloxy)-2-chlorobenzoic acid (**31b**, 61.5 mg, 0.229 mmol, 95%) as an off-white crystalline solid.

Analytical Data of 3,4-Bis(allyloxy)-2-chlorobenzoic Acid (31b)

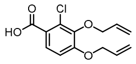

^{1}H NMR (500 MHz, $CDCl_3$) δ 7.86 – 7.80 (m, 1H), 6.89 – 6.83 (m, 1H), 6.21 – 6.10 (m, 1H), 6.10 – 6.01 (m, 1H), 5.51 – 5.23 (m, 4H), 4.68 – 4.65 (m, 2H), 4.57 (dd, *J* = 6.1, 1.5 Hz, 2H) ppm;

^{13}C NMR (126 MHz, $CDCl_3$) δ 169.6, 156.7, 145.5, 133.7, 132.2, 130.8, 128.9, 121.2, 118.7, 118.5, 111.0, 74.3, 69.8 ppm;

IR (thin film): ν 3086w, 2984w, 2930w, 2882w, 2795w, 2638w, 2571w, 1694s, 1670m, 1586s, 1488m, 1434m, 1412m, 1384w, 1359w, 1285s, 1222w, 1177w, 1148w, 1104w, 1008s, 991m, 917m, 870w, 832w, 819w, 782w, 772w, 741w, 705w, 568w, 417w cm^{-1};

HRMS (ESI) for $C_{13}H_{14}O_4Cl^+$ $[M+H]^+$: calculated: 269.05751; found: 269.05766.

ERJ-408, ERJ-619

1.3.7 Sulfamate- and Sulfate-based derivatives

Preparation of 32

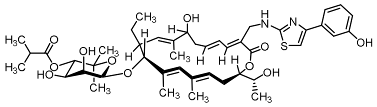

To a microwave vial was added fidaxomicin (**1**, 300 mg, 0.284 mmol, 1.0 eq.), 3-(2-aminothiazol-4-yl)phenol (164 mg, 0.852 mmol, 3.0 eq.), [Pd(cinnamyl)Cl]$_2$ (14.7 mg, 28.4 µmol, 10 mol%) and dppf (31.5 mg, 56.8 µmol, 20 mol%). The vial was capped, evacuated and backfilled with nitrogen three times and then dry THF (1.5 mL) was added. The reaction was placed in a heating block pre-heated to 60 °C, stirring at 1000 rpm. Reaction progress was monitored by UHPLC-MS. After 5 h the reaction mixture was cooled to RT, diluted with THF (2 mL), 3-mercaptopropyl-functionalised silica was added, and the mixture was stirred at RT until no Pd-complexes were observed by UHPLC-MS. The mixture was filtered through a pad of celite, eluted with DCM and MeOH and concentrated *in vacuo*. The resulting residue was purified by automated flash column chromatography (Biotage Isolera, 25 g silica gel, 30-70% EtOAc in hexane) to yield crude32 (~140 mg). This residue was dissolved in MeCN (2 mL) and purified by preparative RP-HPLC ([Gemini NX, C18, 5 µ, 110 Å, 250 mm × 21.2 mm, solvent A: H_2O + 0.1% HCOOH, solvent B: MeCN + 0.1% HCOOH, 18 mL/min; LC time program (min – %B): 7 min – 35%, 80 min – 80%]) to yield, after lyophilisation, the 2-aminothiazole **32** (t_R = 29.7 min, 60.2 mg, 71.7 µmol, 25%) as a colourless solid.

Analytical Data of 32

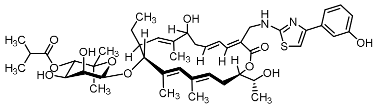

^{1}H NMR (500 MHz, acetone-d_6) δ 7.43 (dd, J = 2.5, 1.5 Hz, 1H), 7.38 (dt, J = 7.8, 1.2 Hz, 1H), 7.19 (d, J = 7.9 Hz, 1H), 7.17 (d, J = 11.2 Hz, 1H), 6.98 – 6.90 (m, 1H), 6.85 (s, 1H), 6.83 (t, J = 5.9 Hz, 1H), 6.75 (ddd, J = 8.0, 2.5, 0.9 Hz, 1H), 5.92 (ddd, J = 14.6, 9.6, 4.6 Hz, 1H), 5.82 (s, 1H), 5.63 – 5.58 (m, 1H), 5.20 (dt, J = 10.7, 1.6 Hz, 1H), 4.99 (d, J = 10.1 Hz, 1H), 4.76 (d, J = 1.2 Hz, 1H), 4.74 – 4.69 (m, 1H), 4.44 – 4.38 (m, 1H), 4.34 – 4.28 (m, 1H), 4.26 (s, 1H), 4.03 (p, J = 6.2 Hz, 1H), 3.96 – 3.92 (m, 1H), 3.74 – 3.69 (m, 2H), 2.79 – 2.73 (m, 0H), 2.72 – 2.69 (m, 1H), 2.68 – 2.60 (m, 1H), 2.59 – 2.52 (m, 2H), 2.40 (ddd, J = 13.8, 8.8, 4.4 Hz, 1H), 1.98 – 1.90 (m, 1H), 1.79 (d, J = 1.4 Hz, 3H), 1.73 (d, J = 1.4 Hz, 3H), 1.66 – 1.62 (m, 3H), 1.27 – 1.19 (m, 1H), 1.16 – 1.13 (m, 12H), 1.12 (s, 3H), 1.08 (s, 3H) ppm;

^{13}C NMR (126 MHz, acetone-d_6) δ 176.8, 168.7, 168.6, 158.4, 151.7, 144.0, 142.4, 137.7, 136.8, 136.3, 136.0, 133.9, 130.2, 129.0, 126.6, 126.2, 124.0, 118.1, 115.1, 113.9, 101.7, 96.7, 93.3, 78.5, 75.7, 73.7, 73.0, 72.8, 70.1, 67.8, 42.1, 40.9, 37.2, 34.8, 28.7, 28.2, 26.4, 20.5, 19.4, 19.2, 18.6, 17.4, 15.2, 13.8, 11.2 ppm;

IR (thin film): ν 3370w, 2976w, 2932w, 2875w, 1732w, 1685m, 1640w, 1601w, 1586w, 1550m, 1450m, 1385m, 1369m, 1343m, 1298m, 1251s, 1207s, 1153s, 1076s, 1032s, 998m, 950w, 897m, 794w, 734m, 717m, 605w, 584w, 523w, 499w, 467w, 447w, 435w, 423w, 415w, 407w, 403w cm^{-1};

HRMS (ESI) for $C_{45}H_{63}O_{11}N_2S^+$ [M+H]$^+$: calculated: 839.41471; found: 839.41437.

ERJ-688, ERJ-689, ERJ-703, ERJ-706

Preparation of 2-bromo-1-(3-hydroxyphenyl)ethan-1-one (32a)

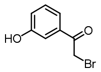

Prepared according to literature procedure.[11] A 50 mL three-necked flask was equipped with magnetic stirring bar, two stopper, and a reflux condenser equipped with a nitrogen bridge connected to a washing bottle filled with an aq. potassium hydroxide solution. Copper(II) bromide (2.12 g, 9.50 mmol, 0.95 eq.) was added to the flask, followed by dry EtOAc (8.3 mL). The stirred mixture was brought to reflux. 3-Hydroxyacetophenone (1.36 g, 10.0 mmol, 1.0 eq.) was suspended in hot, dry $CHCl_3$ (8.3 mL). EtOAc (ca. 3 mL) was added until all solids dissolved. This solution was added dropwise to the refluxing copper(II) bromide suspension. The stirred reaction mixture was heated to reflux for 21 h, during which a colour change from dark green to beige was observed. The reaction mixture was cooled to RT and filtered through a pad of celite and eluted with EtOAc until the filtrate was clear. The filtrate was concentrated *in vacuo* and the resulting residue was purified by flash column chromatography (Biotage Isolera, 40 g silica gel column, 5-22% EtOAc in hexane) to yield 2-bromo-1-(3-hydroxyphenyl)ethan-1-one (655 mg, 3.05 mmol, 30%) as a yellow solid.

Analytical Data of 2-bromo-1-(3-hydroxyphenyl)ethan-1-one (32a)

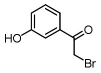

^{1}H NMR (400 MHz, $CDCl_3$) δ 7.57 – 7.52 (m, 1H), 7.51 – 7.49 (m, 1H), 7.41 – 7.35 (m, 1H), 7.14 – 7.10 (m, 1H), 5.38 (s, 1H), 4.45 (s, 2H) ppm.

Analytical data are in agreement with the literature.[12]

ERJ-686

Preparation of 3-(2-aminothiazol-4-yl)phenol (32b)

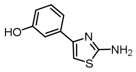

Synthesised according to a modified literature procedure.[12] To a 50 mL round-bottom flask equipped with a magnetic stirring bar was added 2-bromo-1-(3-hydroxyphenyl)ethan-1-one (655 mg, 3.05 mmol, 1.0 eq.), ethanol (15 mL) and thiourea (244 mg, 3.20 mmol, 1.05 eq.). The flask was equipped with a reflux condenser and heated to reflux for 2 h. After TLC indicated full conversion, the reaction mixture was cooled to RT and concentrated *in vacuo*. The resulting residue was treated with EtOAc (20 mL) and a sodium bicarbonate solution (sat. aq., 15 mL) and stirred until gas evolution ceased. The mixture was transferred into a separating funnel and the layers were separated. The aqueous layer was extracted with EtOAc (3 × 10 mL). The combined organic layers were washed with brine (20 mL), dried over sodium sulfate, filtered, and concentrated *in vacuo*. The resulting residue was purified by flash column chromatography (silica gel, MeOH in DCM 2-4%) to yield 3-(2-aminothiazol-4-yl)phenol (576 mg, 3.00 mmol, 98%) as a light brown solid.

Analytical Data of 3-(2-aminothiazol-4-yl)phenol (32b)

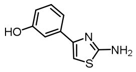

^{1}H NMR (400 MHz, DMSO-d_6) δ 9.38 – 9.31 (m, 1H), 7.24 – 7.17 (m, 2H), 7.13 (td, J = 8.0, 2.6 Hz, 1H), 7.00 (br. s, 2H), 6.92 – 6.85 (m, 1H), 6.71 – 6.58 (m, 1H) ppm.

Analytical data are in agreement with the literature.[12]

ERJ-687

Preparation of 33

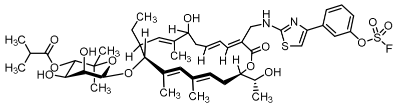

To a flame-dried HPLC vial was added phenol **32** (51.1 mg, 60.9 µmol, 1.0 eq.) and dry THF (200 µL). The stirred mixture was cooled to -20 °C using an NaCl-ice bath. Then a solution of DBU (20.0 µL, 0.134 mmol, 2.2 eq.) in dry THF (50 µL) was added dropwise, followed immediately by dropwise addition of a solution of 4-(acetylamino)phenyl]imidodisulfuryl difluoride (freshly recrystallised, 21.5 mg, 67.0 µmol, 1.1 eq.). The reaction mixture was allowed to warm to 0 °C over 2 h. The reaction was then partitioned between EtOAc (10 mL) and a hydrochloric acid solution (0.5 M aq., 5 mL). The organic layer was separated, and the aqueous layer was extracted with EtOAc (2 × 5 mL). The combined organic layers were washed with brine (20 mL), dried over sodium sulfate, filtered, and concentrated *in vacuo*. The resulting residue was dissolved in MeCN (1 mL) and eluted through a C18 SPE cartridge with MeCN (8 mL). The filtrate was concentrated *in vacuo* and the resulting crude mixture purified by preparative RP-HPLC ([Gemini NX, C18, 5 µ, 110 Å, 250 mm × 21.2 mm, solvent A: H_2O + 0.1% HCOOH, solvent B: MeCN + 0.1% HCOOH, 18 mL/min; LC time program (min – %B): 7 min – 50%, 60 min – 80%]) to yield, after lyophilisation, the fluorosulfate **33** (t_R = 31.1 min, 31.6 mg, 34.3 µmol, 56%, 69% brsm) as an off-white solid.

Analytical Data of 33

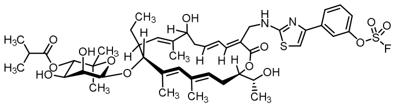

^{1}H NMR (500 MHz, acetone-d_6) δ 8.07 – 8.04 (m, 1H), 8.03 – 8.01 (m, 1H), 7.61 (t, J = 8.0 Hz, 1H), 7.42 (dd, J = 8.2, 2.6 Hz, 1H), 7.20 – 7.16 (m, 2H), 7.02 (t, J = 5.8 Hz, 1H), 6.93 – 6.86 (m, 1H), 5.94 (ddd, J = 14.7, 9.7, 4.5 Hz, 1H), 5.83 (s, 1H), 5.62 – 5.58 (m, 1H), 5.23 – 5.17 (m, 1H), 4.99 (d, J = 10.1 Hz, 1H), 4.78 – 4.75 (m, 1H), 4.73 – 4.69 (m, 1H), 4.47 – 4.42 (m, 1H), 4.33 – 4.26 (m, 1H), 4.04 – 4.00 (m, 1H), 3.96 – 3.93 (m, 1H), 3.82 (d, J = 3.6 Hz, 1H), 3.75 – 3.68 (m, 2H), 3.26 (d, J = 9.4 Hz, 1H), 2.80 (s, 3H), 2.77 – 2.70 (m, 2H), 2.67 – 2.59 (m, 1H), 2.59 – 2.52 (m, 2H), 2.41 (ddd, J = 13.9, 8.9, 4.4 Hz, 1H), 1.98 – 1.91 (m, 1H), 1.80 (d, J = 1.3 Hz, 3H), 1.73 (d, J = 1.4 Hz, 3H), 1.65 (d, J = 1.3 Hz, 3H), 1.28 – 1.19 (m, 1H), 1.16 – 1.12 (m, 12H), 1.08 (s, 3H), 0.82 (t, J = 7.4 Hz, 3H) ppm;

^{13}C NMR (126 MHz, acetone-d_6) δ 176.8, 169.2, 168.4, 151.5, 149.3, 143.9, 142.5, 139.1, 136.9, 136.3, 136.0, 133.9, 131.6, 128.8, 127.0, 126.6, 126.2, 124.0, 120.1, 119.0, 104.3, 96.7, 93.3, 78.5, 75.7, 73.7, 73.0, 72.8, 70.2, 67.7, 42.1, 41.0, 37.3, 34.8, 28.7, 28.2, 26.5, 20.6, 19.4, 19.2, 18.6, 17.4, 15.2, 13.8, 11.2 ppm;

^{19}F NMR (471 MHz, acetone-d_6) δ -75.2 ppm;

IR (thin film): ν 3407w, 2976w, 2933w, 2876w, 1732m, 1687m, 1642w, 1613w, 1553m, 1523w, 1448s, 1386m, 1369m, 1331m, 1302m, 1252m, 1234s, 1206s, 1147m, 1123m, 1072m, 1033s, 1004m, 995m, 976m, 951m, 899m, 854m, 815m, 796m, 771m, 735m, 716m, 682w, 657w, 635w, 571m, 545m, 529m, 503w, 461w, 447w, 439w, 431w cm^{-1};

HRMS (ESI) for $C_{45}H_{62}O_{13}N_2FS_2^+$ $[M+H]^+$: calculated: 921.36719; found: 921.36656.

ERJ-701, ERJ-705, ERJ-710

Preparation of 34

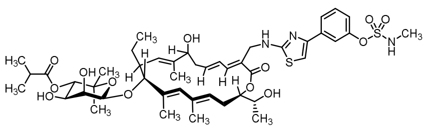

Prepared according to **GP6** on 9.8 µmol scale and a methylamine solution (2 M in THF, 24.4 µL, 48.8 µmol, 5.0 eq.). Once UHPLC-MS showed full conversion, MeCN (100 µL) was added and the mixture was injected into a preparative RP-HPLC ([Gemini NX, C18, 5 µ, 110 Å, 250 mm × 21.2 mm, solvent A: H_2O + 0.1% HCOOH, solvent B: MeCN + 0.1% HCOOH, 18 mL/min; LC time program (min – %B): 7 min – 50%, 60 min – 80%]) to yield, after lyophilisation, the methylsulfamate **34** (t_R = 19.2 min, 7.3 mg, 7.8 µmol, 80%) as an off-white solid.

Analytical Data of 34

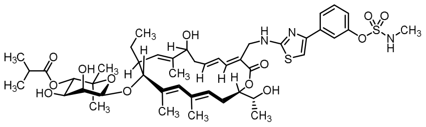

^{1}H NMR (500 MHz, acetone-d_6) δ 7.88 – 7.85 (m, 1H), 7.81 (t, *J* = 2.0 Hz, 1H), 7.44 (t, *J* = 8.0 Hz, 1H), 7.22 (dd, *J* = 8.0, 2.5 Hz, 1H), 7.18 (d, *J* = 11.4 Hz, 1H), 7.04 (s, 1H), 6.97 – 6.86 (m, 2H), 5.94 (ddd, *J* = 14.6, 9.6, 4.6 Hz, 1H), 5.82 (s, 1H), 5.62 – 5.58 (m, 1H), 5.22 – 5.18 (m, 1H), 4.99 (d, *J* = 10.1 Hz, 1H), 4.76 (d, *J* = 1.2 Hz, 1H), 4.72 (q, *J* = 5.5 Hz, 1H), 4.46 – 4.40 (m, 1H), 4.32 – 4.27 (m, 1H), 4.27 (s, 1H), 4.05 – 4.00 (m, 1H), 3.96 – 3.93 (m, 1H), 3.82 (d, *J* = 3.6 Hz, 1H), 3.75 – 3.70 (m, 2H), 3.68 (d, *J* = 4.2 Hz, 1H), 3.26 (d, *J* = 9.4 Hz, 1H), 2.91 (s, 3H), 2.80 (s, 2H), 2.78 – 2.70 (m, 2H), 2.68 – 2.60 (m, 1H), 2.60 – 2.50 (m, 2H), 2.41 (ddd, *J* = 13.9, 9.0, 4.4 Hz, 1H), 1.93 (m, 1H), 1.79 (d, *J* = 1.3 Hz, 3H), 1.73 (d, *J* = 1.4 Hz, 3H), 1.65 (d, *J* = 1.3 Hz, 3H), 1.28 – 1.19 (m, 1H), 1.17 – 1.11 (m, 12H), 1.08 (s, 3H), 0.82 (t, *J* = 7.4 Hz, 3H) ppm;

^{13}C NMR (126 MHz, acetone-d_6) δ 176.8, 169.1, 168.4, 151.7, 150.3, 143.9, 142.6, 138.2, 136.9, 136.9, 136.2, 136.0, 133.9, 130.6, 128.8, 126.6, 126.1, 125.0, 124.0, 121.5, 120.3, 103.1, 96.7, 93.3, 78.5, 75.7, 73.7, 73.0, 72.8, 70.2, 67.7, 42.1, 41.0, 37.3, 34.8, 28.7, 28.2, 26.5, 20.6, 19.4, 19.2, 18.6, 17.4, 15.2, 13.8, 11.2 ppm;

IR (thin film): ν 3387m, 2976m, 2933m, 2875w, 1726m, 1693m, 1641w, 1607w, 1551m, 1523w, 1468m, 1439m, 1385m, 1363m, 1308m, 1253m, 1211m, 1185s, 1142s, 1082s, 1033s, 1008m, 978m, 948m, 876m, 827m, 797m, 747m, 716w, 688w, 564m, 539w, 527w, 503w, 479w cm^{-1};

HRMS (ESI) for $C_{46}H_{66}O_{13}N_3S_2^+$ $[M+H]^+$: calculated: 932.40316; found: 932.40368.

ERJ-714

Preparation of 35

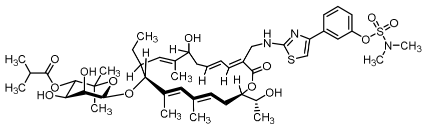

Prepared according to **GP6** on 10.4 µmol scale and a dimethylamine solution (2 M in THF, 20.8 µL, 41.6 µmol, 4.0 eq.). After 12 h, MeCN (100 µL) was added and the mixture was injected into a preparative RP-HPLC ([Gemini NX, C18, 5 µ, 110 Å, 250 mm × 21.2 mm, solvent A: H_2O + 0.1% HCOOH, solvent B: MeCN + 0.1% HCOOH, 18 mL/min; LC time program (min – %B): 7 min – 50%, 60 min – 80%]) to yield, after lyophilisation, the dimethylsulfamate **35** (t_R = 24.5 min, 6.7 mg, 7.1 µmol, 68%) as an off-white solid.

Analytical Data of 35

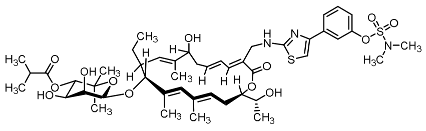

^{1}H NMR (500 MHz, acetone-d_6) δ 7.90 – 7.87 (m, 1H), 7.85 – 7.83 (m, 1H), 7.46 (t, J = 8.0 Hz, 1H), 7.24 (dd, J = 8.1, 2.5 Hz, 1H), 7.18 (d, J = 11.4 Hz, 1H), 7.06 (s, 1H), 6.93 – 6.85 (m, 2H), 5.94 (ddd, J = 14.7, 9.6, 4.6 Hz, 1H), 5.82 (s, 1H), 5.61 (t, J = 8.3 Hz, 1H), 5.21 (d, J = 10.6 Hz, 1H), 4.99 (d, J = 10.1 Hz, 1H), 4.76 (s, 1H), 4.72 (q, J = 5.5 Hz, 1H), 4.45 – 4.40 (m, 1H), 4.33 – 4.29 (m, 1H), 4.28 – 4.26 (m, 1H), 4.03 – 3.99 (m, 1H), 3.96 – 3.94 (m, 1H), 3.80 (d, J = 3.6 Hz, 1H), 3.73 – 3.70 (m, 2H), 3.65 (d, J = 4.2 Hz, 1H), 3.25 (d, J = 9.4 Hz, 1H), 2.99 (s, 6H), 2.79 – 2.78 (m, 3H, *Note: peak integration likely to high due to proximity to water peak*), 2.77 – 2.70 (m, 1H), 2.64 (qd, J = 9.7, 3.2 Hz, 1H), 2.59 – 2.52 (m, 2H), 2.42 (ddd, J = 13.9, 9.0, 4.4 Hz, 1H), 1.97 – 1.91 (m, 1H), 1.79 (d, J = 1.2 Hz, 3H), 1.73 (s, 3H), 1.64 (s, 3H), 1.24 (dt, J = 13.2, 7.7 Hz, 1H), 1.17 – 1.12 (m, 12H), 1.08 (s, 3H), 0.83 (t, J = 7.4 Hz, 3H) ppm;

^{13}C NMR (126 MHz, acetone-d_6) δ 176.8, 169.1, 168.4, 151.7, 150.2, 144.0, 142.6, 138.3, 136.9, 136.2, 136.1, 133.9, 130.7, 128.8, 126.6, 126.1, 125.0, 124.0, 121.3, 120.1, 103.2, 96.7, 93.3, 78.5, 75.7, 73.8, 73.0, 72.8, 70.2, 67.7, 42.1, 41.0, 39.0, 37.4, 34.8, 28.7, 28.2, 26.5, 20.6, 19.4, 19.2, 18.6, 17.4, 15.2, 13.8, 11.2 ppm;

IR (thin film): ν 3412w, 2975m, 2933m, 2875w, 1733m, 1695m, 1641w, 1607w, 1551m, 1523w, 1468m, 1440m, 1417m, 1372s, 1330m, 1306m, 1253m, 1210m, 1177s, 1139s, 1076m, 1033s, 1005m, 977m, 946m, 898m, 874m, 826m, 798m, 758m, 714w, 563m, 527w, 505w, 487w, 479w cm^{-1};

HRMS (ESI) for $C_{47}H_{68}O_{13}N_3S_2^+$ $[M+H]^+$: calculated: 946.41881; found: 946.41921.

ERJ-713

Preparation of 36

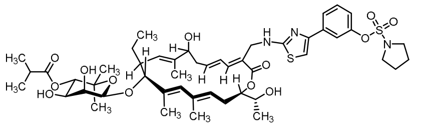

Prepared according to **GP6** on 8.7 µmol scale and pyrrolidine (2.1 µL, 26 µmol, 3.0 eq.). After 2 h, MeCN (100 µL) was added and the mixture was injected into a preparative RP-HPLC ([Gemini NX, C18, 5 µ, 110 Å, 250 mm × 21.2 mm, solvent A: H_2O + 0.1% HCOOH, solvent B: MeCN + 0.1% HCOOH, 18 mL/min; LC time program (min – %B): 7 min – 50%, 60 min – 80%]) to yield, after lyophilisation, the pyrrolidine-1-sulfonate **36** (t_R = 28.0 min, 5.9 mg, 6.1 µmol, 70%) as an off-white solid.

Analytical Data of 36

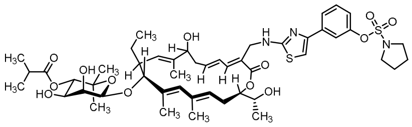

^{1}H NMR (500 MHz, acetone-d_6) δ 7.90 – 7.87 (m, 1H), 7.85 (t, *J* = 2.0 Hz, 1H), 7.45 (t, *J* = 7.9 Hz, 1H), 7.27 – 7.23 (m, 1H), 7.18 (d, *J* = 11.4 Hz, 1H), 7.06 (s, 1H), 6.93 (t, *J* = 5.7 Hz, 1H), 6.92 – 6.84 (m, 1H), 5.94 (ddd, *J* = 14.6, 9.6, 4.6 Hz, 1H), 5.82 (s, 1H), 5.61 (t, *J* = 8.3 Hz, 1H), 5.23 – 5.18 (m, 1H), 4.99 (d, *J* = 10.1 Hz, 1H), 4.78 – 4.75 (m, 1H), 4.72 (q, *J* = 5.4 Hz, 1H), 4.45 – 4.40 (m, 1H), 4.34 – 4.28 (m, 1H), 4.28 – 4.25 (m, 1H), 4.03 – 4.00 (m, 1H), 3.97 – 3.92 (m, 1H), 3.82 (d, *J* = 3.6 Hz, 1H), 3.73 – 3.70 (m, 1H), 3.66 (d, *J* = 4.2 Hz, 1H), 3.44 – 3.39 (m, 4H), 3.26 (d, *J* = 9.4 Hz, 1H), 2.80 (s, 2H), 2.77 – 2.71 (m, 2H), 2.68 – 2.61 (m, 1H), 2.59 – 2.51 (m, 2H), 2.42 (ddd, *J* = 13.9, 9.0, 4.4 Hz, 1H), 1.98 – 1.95 (m, 4H), 1.95 – 1.90 (m, 1H), 1.79 (s, 3H), 1.72 (s, 3H), 1.64 (s, 3H), 1.28 – 1.19 (m, 1H), 1.16 – 1.12 (m, 12H), 1.08 (s, 3H), 0.82 (t, *J* = 7.5 Hz, 3H) ppm;

^{13}C NMR (126 MHz, acetone-d_6) δ 176.8, 169.1, 168.4, 151.8, 150.2, 144.0, 142.7, 138.2, 136.9, 136.2, 136.0, 133.9, 130.6, 128.7, 126.5, 126.1, 124.9, 124.0, 121.5, 120.3, 103.1, 96.7, 93.3, 78.4, 75.7, 73.7, 72.9, 72.8, 70.1, 67.7, 50.1, 42.0, 41.0, 37.3, 34.8, 28.7, 28.2, 26.5, 26.3, 20.6, 19.4, 19.2, 18.6, 17.4, 15.2, 13.8, 11.2 ppm;

IR (thin film): ν 3414w, 2976m, 2932m, 2876w, 1733m, 1695s, 1641w, 1607w, 1579w, 1551m, 1522w, 1467m, 1440m, 1377s, 1338m, 1309m, 1250s, 1208s, 1173s, 1145s, 1079s, 1033s, 978m, 947m, 899m, 874m, 825m, 798m, 768m, 737w, 716w, 688w, 647w, 609m, 579w, 559m, 527w, 506w, 479w cm^{-1};

HRMS (ESI) for $C_{49}H_{70}O_{13}N_3S_2^+$ $[M+H]^+$: calculated: 972.43446; found: 972.43430.

ERJ-712

Preparation of 37

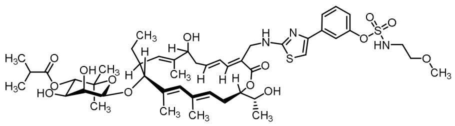

Prepared according to **GP6** on 6.4 µmol scale and 2-methoxyethylamine (2.8 µL, 32 µmol, 5.0 eq.). After 6 h, MeCN (70 µL) was added and the mixture was injected into a preparative RP-HPLC ([Gemini NX, C18, 5 µ, 110 Å, 250 mm × 21.2 mm, solvent A: H_2O + 0.1% HCOOH, solvent B: MeCN + 0.1% HCOOH, 18 mL/min; LC time program (min – %B): 7 min – 40%, 60 min – 70%]) to yield, after lyophilisation, the (2-methoxyethyl)sulfamate **37** (t_R = 35.8 min, 3.7 mg, 3.8 µmol, 59%) as an off-white solid.

Analytical Data of 37

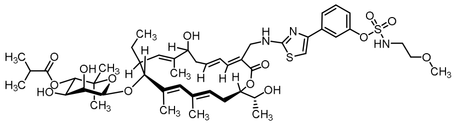

^{1}H NMR (500 MHz, acetone-d_6) δ 7.77 – 7.74 (m, 1H), 7.72 (t, *J* = 2.0 Hz, 1H), 7.32 (t, *J* = 7.9 Hz, 1H), 7.15 – 7.11 (m, 1H), 7.06 (d, *J* = 11.4 Hz, 1H), 6.91 (s, 1H), 6.82 – 6.75 (m, 2H), 5.81 (ddd, *J* = 14.6, 9.6, 4.6 Hz, 1H), 5.70 (s, 1H), 5.51 – 5.46 (m, 1H), 5.10 – 5.06 (m, 1H), 4.87 (d, *J* = 10.0 Hz, 1H), 4.64 (s, 1H), 4.60 (td, *J* = 5.7, 4.2 Hz, 1H), 4.34 – 4.28 (m, 1H), 4.22 – 4.16 (m, 1H), 4.16 – 4.13 (m, 1H), 3.93 – 3.89 (m, 1H), 3.84 – 3.80 (m, 1H), 3.70 (d, *J* = 3.6 Hz, 1H), 3.63 – 3.57 (m, 2H), 3.54 (d, *J* = 4.2 Hz, 1H), 3.42 (t, *J* = 5.5 Hz, 2H), 3.29 (t, *J* = 5.5 Hz, 2H), 3.18 (s, 3H), 3.13 (d, *J* = 9.4 Hz, 1H), 2.68 – 2.66 (m, 6H, *Note: peak integration likely to high due to proximity to water peak*), 2.65 – 2.59 (m, 1H), 2.56 – 2.48 (m, 1H), 2.48 – 2.40 (m, 2H), 2.29 (ddd, *J* = 13.8, 9.0, 4.5 Hz, 1H), 1.85 – 1.79 (m, 1H), 1.67 (d, *J* = 1.3 Hz, 3H), 1.60 (d, *J* = 1.4 Hz, 3H), 1.52 (s, 3H), 1.16 – 1.07 (m, 1H), 1.04 – 0.99 (m, 12H), 0.96 (s, 3H), 0.70 (t, *J* = 7.4 Hz, 3H) ppm;

^{13}C NMR (126 MHz, acetone-d_6) δ 175.9, 168.2, 167.5, 150.8, 149.5, 143.1, 141.7, 137.3, 136.0, 135.3, 135.2, 133.0, 129.6, 127.9, 125.6, 125.2, 124.1, 123.1, 120.7, 119.4, 102.2, 95.8, 92.4, 77.6, 74.8, 72.8, 72.1, 71.9, 70.5, 69.3, 66.8, 57.8, 43.7, 41.2, 40.1, 36.4, 33.9, 27.8, 27.3, 25.6, 19.7, 18.5, 18.3, 17.7, 16.5, 14.3, 12.9, 10.3 ppm;

IR (thin film): ν 3385w, 2975m, 2931m, 2875w, 1733m, 1688m, 1641w, 1607w, 1552m, 1466m, 1441m, 1384m, 1368s, 1307m, 1254m, 1210s, 1200s, 1182s, 1142s, 1084s, 1032s, 1009m, 977m, 948m, 897m, 876m, 827m, 799m, 745m, 716w, 688w, 637w, 564m, 524w, 469w, 465w cm^{-1};

HRMS (ESI) for $C_{48}H_{70}O_{14}N_3S_2^+$ $[M+H]^+$: calculated: 976.42937; found: 976.42921.

ERJ-715

Preparation of 38

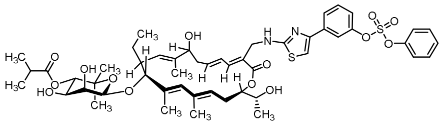

Based on a modified literature procedure.[13] Two HPLC vials were flame-dried. One was charged with fluorosulfate **33** (6.8 mg, 7.4 µmol, 1.0 eq.) and dry MeCN (50 µL). In the other vial phenol (0.8 mg, 7 µmol, 1.2 eq.) was dissolved in dry MeCN (30 µL). To the phenol solution were added successively a solution of hexamethyldisilane (freshly distilled under N_2, 1.9 µL, 8.9 µL, 1.2 eq.) in dry MeCN (10 µL) and a solution of 2-*tert*-butyl-1,1,3,3-tetramethylguanidine (0.18 µL, 0.89 µmol, 12 mol%). This mixture was then added dropwise to the stirred fluorosulfate solution at RT over 2 min. After 19 h, MeCN (100 µL) was added and the mixture was injected into a preparative RP-HPLC ([Gemini NX, C18, 5 µ, 110 Å, 250 mm × 21.2 mm, solvent A: H_2O + 0.1% HCOOH, solvent B: MeCN + 0.1% HCOOH, 18 mL/min; LC time program (min – %B): 7 min – 50%, 60 min – 80%]) to yield, after lyophilisation, the phenylsulfate **38** (t_R = 37.0 min, 3.8 mg, 3.8 µmol, 52%) as an off-white solid.

Analytical Data of 38

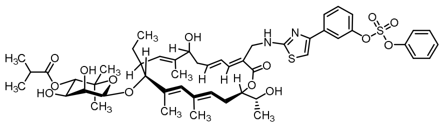

^{1}H NMR (500 MHz, acetone-d_6) δ 7.98 – 7.95 (m, 1H), 7.93 – 7.90 (m, 1H), 7.59 – 7.51 (m, 3H), 7.48 – 7.44 (m, 2H), 7.34 (ddd, *J* = 8.2, 2.6, 0.9 Hz, 1H), 7.18 (d, *J* = 11.4 Hz, 1H), 7.10 (s, 1H), 6.97 (t, *J* = 5.8 Hz, 1H), 6.92 – 6.84 (m, 1H), 5.93 (ddd, *J* = 14.7, 9.6, 4.5 Hz, 1H), 5.82 (s, 1H), 5.63 – 5.57 (m, 1H), 5.23 – 5.17 (m, 1H), 4.99 (d, *J* = 10.1 Hz, 1H), 4.76 (s, 1H), 4.71 (q, *J* = 5.4 Hz, 1H), 4.45 – 4.40 (m, 1H), 4.30 (dd, *J* = 14.0, 5.2 Hz, 1H), 4.26 – 4.23 (m, 1H), 4.03 – 4.01 (m, 1H), 3.96 – 3.94 (m, 1H), 3.82 (d, *J* = 3.6 Hz, 1H), 3.73 – 3.70 (m, 2H), 3.66 (d, *J* = 4.2 Hz, 1H), 3.26 (d, *J* = 9.4 Hz, 1H), 2.79 (m, 6H, *Note: peak integration likely to high due to proximity to water peak*), 2.77 – 2.69 (m, 2H), 2.66 – 2.60 (m, 1H), 2.59 – 2.50 (m, 2H), 2.41 (ddd, *J* = 13.8, 9.0, 4.4 Hz, 1H), 1.97 – 1.91 (m, 1H), 1.79 (d, *J* = 1.3 Hz, 3H), 1.72 (d, *J* = 1.4 Hz, 3H), 1.63 (d, *J* = 1.3 Hz, 3H), 1.24 (ddd, *J* = 13.4, 8.7, 7.3 Hz, 1H), 1.16 – 1.12 (m, 12H), 1.08 (s, 3H), 0.82 (t, *J* = 7.4 Hz, 3H) ppm;

^{13}C NMR (126 MHz, acetone-d_6) δ 176.8, 169.2, 168.4, 151.8, 151.5, 149.7, 144.0, 142.6, 138.7, 136.9, 136.2, 136.0, 133.9, 131.3, 131.2, 128.8, 128.7, 126.5, 126.1, 125.9, 124.0, 122.0, 120.2, 119.3, 103.7, 96.7, 93.3, 78.5, 75.7, 73.7, 72.9, 72.8, 70.2, 67.7, 42.0, 41.0, 37.3, 34.8, 28.7, 28.2, 26.5, 20.6, 19.4, 19.2, 18.6, 17.4, 15.2, 13.8, 11.2 ppm;

IR (thin film): ν 3416m, 2976m, 2932m, 2875w, 1733m, 1690m, 1641w, 1610w, 1551m, 1487m, 1471m, 1411m, 1331m, 1312m, 1253m, 1202s, 1146s, 1073m, 1033s, 1007m, 950m, 883s, 842m, 795m, 715w, 688w, 569m, 539w, 513w, 419w cm^{-1};

HRMS (ESI) for $C_{51}H_{67}O_{14}N_2S_2^+$ $[M+H]^+$: calculated: 995.40282; found: 995.40423.

ERJ-715

1.3.8 Derivatives Containing Noviose Modifications

Preparation of Shimada's Boronic Acid Catalyst

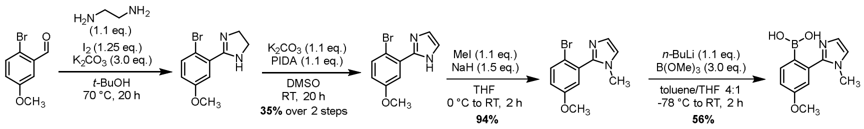

Prepared according to literature procedure.[14] In a flask under nitrogen ethylenediamine (1.72 mL, 25.6 mmol, 1.1 eq.), was added to a stirred solution of 2-bromo-5-methoxybenzaldehyde (5.00 g, 23.3 mmol, 1.0 eq.) in *t*-BuOH (117 mL). After 30 min, iodine (7.39 g, 29.1 mmol, 1.25 eq.) and potassium carbonate (9.66 g, 69.9 mmol, 3.0 eq.) were added. The reaction mixture was heated to 70 °C for 20 h. The reaction was quenched by addition of a sodium sulfate solution (sat. aq., 100 mL) and the resulting mixture was extracted with chloroform (3 × 100 mL). The combined organic layers were washed with a sodium bicarbonate solution (sat. aq., 60 mL) and brine (60 mL), dried over sodium sulfate, filtered, and concentrated *in vacuo* to yield the crude 2-(2-bromo-5-methoxyphenyl)-4,5-dihydro-1*H*-imidazole which was used in the next step without further purification.

To a stirred solution of the 2-(2-bromo-5-methoxyphenyl)-4,5-dihydro-1*H*-imidazole and potassium carbonate (3.54 g, 25.6 mmol, 1.1 eq.) in DMSO (233 mL) was added PIDA (8.26 g, 25.6 mmol, 1.1 eq.). The reaction mixture was stirred at RT for 20 h and then quenched by addition of a sodium bicarbonate solution (sat. aq., 150 mL). The resulting mixture was extracted with EtOAc (3 × 100 mL). The combined organic layers were washed with water (5 × 500 mL), brine (200 mL), dried over sodium sulfate, filtered and concentrated *in vacuo*. The resulting residue was recrystallised from chloroform to yield 2-(2-bromo-5-methoxyphenyl)-1*H*-imidazole (2.08 g, 8.22 mmol, 35% *over two steps*) as a colourless solid.

^{1}H NMR (400 MHz, $CDCl_3$) δ 7.63 (d, *J* = 3.2 Hz, 1H), 7.45 (d, *J* = 8.8 Hz, 1H), 7.19 (s, 2H), 6.78 (dd, *J* = 8.8, 3.2 Hz, 1H), 3.82 (s, 3H) ppm.

Analytical data are in agreement with the literature.[14]

In a flame-dried flask under nitrogen to a stirred suspension of sodium hydride (494 mg, 12.3 mmol, 1.5 eq.) in THF (40 mL) at 0 °C was added a solution of 2-(2-bromo-5-methoxyphenyl)-1*H*-imidazole (2.08 g, 8.23 mmol, 1.0 eq.) in THF (40 mL). The reaction mixture was stirred at 0 °C for 30 min, then iodomethane (0.564 mL, 9.05 mmol, 1.1 eq.) was added dropwise. The reaction mixture was allowed to warm to RT and was stirred for 1 h. The mixture was poured onto ice and extracted with EtOAc (3 × 80 mL). The combined organic layers were washed with water (50 mL), brine (50 mL), dried over sodium sulfate, filtered and concentrated *in vacuo*. The resulting residue was purified by flash column chromatography (silica gel, hexane/EtOAc 1:2) to yield 2-(2-bromo-5-methoxyphenyl)-1-methyl-1*H*-imidazole (2.07 g, 7.74 mmol, 94%) as a yellow oil.

^{1}H NMR (400 MHz, $CDCl_3$) δ 7.51 (d, *J* = 8.9 Hz, 1H), 7.15 (d, *J* = 1.3 Hz, 1H), 7.00 (d, *J* = 1.2 Hz, 1H), 6.98 (d, *J* = 3.1 Hz, 1H), 6.89 (dd, *J* = 8.8, 3.1 Hz, 1H), 3.80 (s, 3H), 3.54 (s, 3H) ppm.

Analytical data are in agreement with the literature.[14]

In a flame-dried flask under nitrogen to a stirred mixture of 2-(2-bromo-5-methoxyphenyl)-1-methyl-1*H*-imidazole (200 mg, 0.79 mmol, 1.0 eq.) in toluene/THF (4:1, 1.5 mL) at -78 °C was added dropwise *n*-BuLi (1.6 M in hexanes, 0.54 mL, 0.87 mmol, 1.1 eq.). After stirring at -78°C for 30 min, trimethyl borate (0.26 mL, 2.4 mmol, 3.0 eq.) was added dropwise, the reaction mixture was allowed to warm up to RT and was stirred for 1 h. Several drops of a sodium hydroxide solution (1 M aq.) were added, the mixture was stirred at RT and monitored for boronate ester disappearance by TLC. The mixture was concentrated in vacuo and purified by flash column chromatography (silica gel, 2-10% ammonia (7 M in MeOH) in DCM) to yield Shimada's catalyst (104 mg, 0.448 mmol, 57%) as a colourless solid.

^{1}H NMR (400 MHz, methanol-*d_4*) δ 7.46 (d, *J* = 8.0 Hz, 1H), 7.31 – 7.28 (m, 2H), 7.21 (s, 1H), 7.00 (dd, *J* = 8.1, 2.3 Hz, 1H), 4.06 (s, 3H), 3.87 (s, 3H) ppm;

^{11}B NMR (161 MHz, methanol-*d_4*) δ 7.05 ppm.

Analytical data are in agreement with the literature.[14]

Preparation of OP1118 (39)

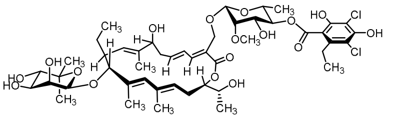

Prepared according to a literature procedure.[15] To a solution of fidaxomicin (**1**, 1.00 g, 0.95 mmol, 1.0 eq.) in methanol (10 mL) was added potassium carbonate (286 mg, 1.89 mmol, 2.0 eq.). The reaction mixture was stirred at RT for 2 h. The reaction was diluted with EtOAc (15 mL) and quenched by addition of an ammonium chloride solution (sat. aq., 30 mL). The organic layer was separated and the aqueous layer was extracted with EtOAc (4 × 50 mL). The combined organic layers were dried over sodium sulfate, filtered and concentrated *in vacuo*. The residue was dissolved in MeCN (1 mL) and eluted through a C18 SPE cartridge with MeCN (8 mL). The resulting solution was concentrated *in vacuo* and the crude mixture purified using preparative RP-HPLC ([Gemini NX, C18, 5 μ, 110 Å, 250 mm × 21.2 mm, solvent A: H_2O + 0.1% HCOOH, solvent B: MeCN + 0.1% HCOOH, 20 mL/min; LC time program (min – %B): 15 min – 30%, 100 min – 40%]) to yield, after lyophilisation, **OP1118** (**39**) (t_R = 38.6 min, 584 mg, 0.591 mmol, 63%) as a colourless solid.

Analytical Data of OP1118 (39)

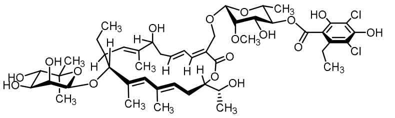

^{1}H NMR (400 MHz, acetone-*d6*) δ 7.24 (d, *J* = 11.4 Hz, 1H), 6.63 (dd, *J* = 15.0, 11.5 Hz, 1H), 5.96 (ddd, *J* = 14.6, 9.6, 4.6 Hz, 1H), 5.82 (s, 1H), 5.69 – 5.58 (m, 1H), 5.21 (d, *J* = 10.5 Hz, 1H), 5.10 (t, *J* = 9.7 Hz, 1H), 4.74 (dt, *J* = 6.6, 4.9 Hz, 1H), 4.71 – 4.66 (m, 2H), 4.60 (d, *J* = 11.4 Hz, 1H), 4.42 (d, *J* = 11.5 Hz, 1H), 4.26 (s, 1H), 4.08 – 3.97 (m, 1H), 3.92 – 3.87 (m, 1H), 3.81 (dd, *J* = 9.9, 3.4 Hz, 1H), 3.71 (d, *J* = 9.7 Hz, 1H), 3.66 – 3.55 (m, 2H), 3.55 – 3.47 (m, 5H), 3.00 (q, *J* = 7.4 Hz, 2H), 2.80 – 2.71 (m, 1H), 2.69 – 2.57 (m, 2H), 2.55 – 2.38 (m, 2H), 1.98 – 1.86 (m, 1H), 1.81 (s, 3H), 1.73 (s, 3H), 1.65 (s, 3H), 1.31 (d, *J* = 6.2 Hz, 3H), 1.25 – 1.16 (m, 11H), 1.07 (s, 3H), 0.82 (t, *J* = 7.4 Hz, 3H) ppm.

Analytical data are in agreement with the literature.[15]

ERJ-156

Preparation of Amine 40 and Isomer 40i

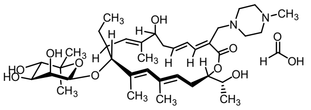 **40** 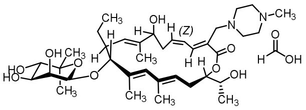 **40i**

To a microwave vial was added fidaxomicin (**1**, 1.00 g 0.95 mmol, 1.0 eq.) and $Pd(PPh_3)_4$ (54.6 mg, 47.3 µmol, 5 mol%). The vial was sealed, evacuated and backfilled with nitrogen three times and then dry MeOH (3.9 mL) was added, followed by 1-methylpiperazine (312 µL, 2.84 mmol, 3.0 eq.). After 48 h the reaction mixture was cooled to RT. Then potassium carbonate (131 mg, 0.946 mmol, 2.0 eq.) was added and the reaction mixture stirred for 1.5 h at RT. The mixture was diluted with EtOAc (40 mL) and water (40 mL). The organic layer was separated, and the aqueous layer was extracted with EtOAc (5 × 50 mL). The aqueous layer was saturated with NaCl and extracted with EtOAc (3 × 50 mL). The combined organic layers were washed with brine (50 mL), dried over sodium sulfate, filtered and concentrated *in vacuo*. The resulting residue was dissolved in MeCN (4 mL) and eluted through a C18 SPE cartridge with MeCN (60 mL). The resulting solution was concentrated *in vacuo* and the crude mixture purified by preparative RP-HPLC ([Gemini NX, C18, 5 µ, 110 Å, 250 mm × 21.2 mm, solvent A: H_2O + 0.1% HCOOH, solvent B: MeCN + 0.1% HCOOH, 20 mL/min; LC time program (min – %B): 5 min – 2%, 7 min – 6%, 50 min – 12%]) to yield, after concentration *in vacuo*, the formate salt of the *N*-methylpiperazine **40** (t_R = 17.7 min, 340.4 mg, 0.4709 mmol, 50%) as an off-white solid and the formate salt of the *Z*-isomer **40i** (t_R = 19.0 min, 53.0 mg, 75.6 µmol, 8%) as an off-white solid.

Analytical Data of Amine 40

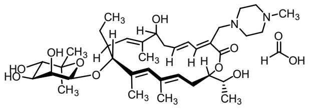

^{1}H NMR (500 MHz, acetone-d_6) 8.24 (s, 1H), 7.13 (d, J = 11.3 Hz, 1H), 6.62 – 6.51 (m, 1H), 5.87 (ddd, J = 14.7, 9.5, 4.7 Hz, 1H), 5.82 (s, 1H), 5.59 (t, J = 8.2 Hz, 1H), 5.23 – 5.16 (m, 1H), 4.69 (d, J = 4.0 Hz, 2H), 4.24 (d, J = 3.8 Hz, 1H), 4.01 (p, J = 6.3 Hz, 1H), 3.88 (dd, J = 2.9, 1.3 Hz, 1H), 3.69 (d, J = 9.8 Hz, 1H), 3.52 – 3.44 (m, 2H), 3.39 (d, J = 12.6 Hz, 1H), 3.20 (d, J = 12.7 Hz, 1H), 2.76 (dt, J = 14.1, 7.0 Hz, 1H), 2.70 – 2.58 (m, 2H), 2.57 – 2.34 (m, 11H), 2.27 (s, 3H), 1.94 (ddd, J = 13.2, 7.6, 3.1 Hz, 1H), 1.79 (d, J = 1.3 Hz, 3H), 1.77 – 1.74 (m, 3H), 1.67 – 1.62 (m, 3H), 1.28 – 1.20 (m, 1H), 1.20 (s, 3H), 1.16 (d, J = 6.3 Hz, 3H), 1.07 (s, 3H), 0.82 (t, J = 7.4 Hz, 3H) ppm;

^{13}C NMR (126 MHz, acetone-d$_6$) δ 168.9, 164.9, 143.9, 141.7, 136.8, 136.1, 136.0, 133.8, 128.6, 126.8, 126.1, 124.1, 96.7, 93.0, 78.2, 75.0, 74.7, 72.9, 72.6, 72.2, 67.7, 55.3, 53.4, 52.7, 45.5, 42.2, 37.2, 29.0, 28.3, 26.5, 20.6, 17.6, 17.4, 15.2, 13.8, 11.2 ppm;

IR (thin film): ν 3660w, 3384m, 2972m, 2931m, 1697m, 1639m, 1597s, 1459m, 1408m, 1379m, 1347m, 1298m, 1244m, 1207m, 1175m, 1161m, 1144m, 1077s, 1029s, 986m, 934w, 895m, 813w, 789m, 762w, 713w, 684w, 636w, 585w, 546w, 512w cm^{-1};

HRMS (ESI) for $C_{37}H_{61}O_9N_2^+$ [M+H]$^+$: calculated: 677.43716; found: 677.43740.

ERJ-192

Analytical Data of Isomer 40i

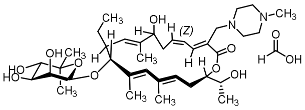

^{1}H NMR (500 MHz, acetone-d_6) δ 8.00 (s, 1H), 7.48 (d, *J* = 11.8 Hz, 1H), 6.62 – 6.52 (m, 1H), 6.09 – 6.00 (m, 1H), 5.75 (s, 1H), 5.54 (t, *J* = 7.7 Hz, 1H), 5.22 (d, *J* = 10.8 Hz, 1H), 4.79 (td, *J* = 6.4, 3.4 Hz, 1H), 4.69 (s, 1H), 4.05 – 3.98 (m, 3H), 3.90 (d, *J* = 2.7 Hz, 1H), 3.67 (d, *J* = 9.5 Hz, 1H), 3.53 – 3.41 (m, 8H), 3.35 (d, *J* = 12.5 Hz, 1H), 2.79 – 2.71 (m, 1H), 2.55 (s, 4H), 2.51 (s, 1H), 2.45 – 2.40 (m, 1H), 2.40 – 2.33 (m, 2H), 2.28 (s, 3H), 2.08 (d, *J* = 1.2 Hz, 3H), 2.00 – 1.90 (m, 1H), 1.75 (s, 3H), 1.69 (s, 3H), 1.68 (s, 3H), 1.21 – 1.17 (m, 4H), 1.14 (d, *J* = 6.4 Hz, 3H), 1.08 (s, 3H), 0.83 (t, *J* = 7.5 Hz, 3H) ppm;

^{13}C NMR (126 MHz, acetone-d_6) δ 168.3, 164.8, 161.2, 140.8, 139.7, 136.9, 136.5, 135.8, 132.7, 128.3, 125.4, 125.3, 123.3, 96.9, 93.0, 78.6, 75.0, 74.7, 74.1, 72.4, 72.2, 67.5, 56.0, 54.8, 54.5, 52.9, 51.3, 46.0, 45.5, 44.2, 42.4, 39.9, 37.0, 29.0, 28.5, 26.3, 20.6, 17.9, 17.8, 15.8, 13.8, 11.7 ppm;

IR (thin film): ν 3376m, 2972m, 2930m, 2111w, 1698m, 1597s, 1454m, 1377m, 1345m, 1310m, 1242s, 1204m, 1144m, 1076s, 1033s, 986m, 942w, 879w, 831w, 789w, 762w, 715w, 595w, 514w, 468w cm^{-1};

HRMS (ESI) for $C_{37}H_{61}O_9N_2^+$ $[M+H]^+$: calculated: 677.43716; found: 677.43722.

Preparation of Amine 41

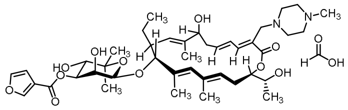

To an HPLC vial was added OP-1118 (**39**, 30.0 mg, 30.4 µmol, 1.0 eq.) and $Pd(PPh_3)_4$ (3.5 mg, 3.0 µmol, 10 mol%). The vial was capped, evacuated and backfilled with nitrogen three times and then dry MeOH (0.16 mL) was added, followed by 1-methylpiperazine (8.3 µL, 91 µmol, 3.0 eq.). The reaction was heated under stirring to 40 °C. Reaction progress was monitored by UHPLC-MS. After 48 h, the solvent was removed under a stream of nitrogen, followed by drying under high vacuum. Under ambient atmosphere Shimada's catalyst (3.5 mg, 15 µmol, 50 mol%) was added and the crude mixture was dissolved in dry 1,4-dioxane (0.16 mL) and DIPEA (47.7 µL, 0.274 mmol, 9.0 eq.). To the stirred mixture was added 3-furoyl chloride (40.1 mg, 0.213 mmol, 7.0 eq.) at 40 °C, the vial was capped and heated under stirring to 40 °C. Reaction progress was monitored by UHPLC-MS. After 20 h, the reaction mixture was cooled to RT, quenched by addition of a drop of MeOH and concentrated *in vacuo*. The residue was dissolved in MeCN (2 mL) and eluted through a C18 SPE cartridge with MeCN (8 mL). The filtrate was concentrated *in vacuo* and the crude mixture purified by preparative RP-HPLC ([Gemini NX, C18, 5 µ, 110 Å, 250 mm × 21.2 mm, solvent A: H_2O + 0.1% HCOOH, solvent B: MeCN + 0.1% HCOOH, 20 mL/min; LC time program (min – %B): 5 min – 2%, 7 min – 13%, 50 min – 18]) to yield, after concentration *in vacuo*, the formate salt of the *N*-methylpiperazine **41** (t_R = 21.5 min, 9.7 mg, 12 µmol, 39%) as an off-white solid.

Analytical Data of Amine 41

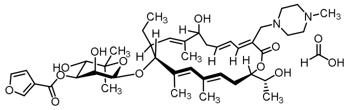

^{1}H NMR (500 MHz, acetone-d_6) δ 8.22 (dd, J = 1.6, 0.7 Hz, 1H), 8.17 (s, 1H), 7.66 (t, J = 1.8 Hz, 1H), 7.15 (d, J = 11.4 Hz, 1H), 6.80 (dd, J = 1.8, 0.8 Hz, 1H), 6.58 (dddd, J = 13.5, 11.4, 2.1, 1.1 Hz, 1H), 5.94 – 5.85 (m, 2H), 5.63 (t, J = 8.2 Hz, 1H), 5.20 (dt, J = 10.7, 1.5 Hz, 1H), 4.94 (dd, J = 10.3, 3.1 Hz, 1H), 4.83 (d, J = 1.2 Hz, 1H), 4.70 (td, J = 6.3, 4.2 Hz, 1H), 4.26 – 4.22 (m, 1H), 4.14 (dd, J = 3.1, 1.2 Hz, 1H), 4.03 (p, J = 6.3 Hz, 1H), 3.90 (d, J = 10.3 Hz, 1H), 3.78 (d, J = 9.7 Hz, 1H), 3.43 (d, J = 12.7 Hz, 1H), 3.24 (d, J = 12.6 Hz, 1H), 2.79 (dt, J = 14.3, 7.2 Hz, 1H), 2.70 – 2.51 (m, 12H), 2.52 – 2.38 (m, 1H), 2.37 (s, 3H), 1.96 – 1.90 (m, 1H), 1.81 (d, J = 1.3 Hz, 3H), 1.78 (d, J = 1.4 Hz, 3H), 1.66 – 1.64 (m, 3H), 1.26 (s, 3H), 1.24 – 1.20 (m, 1H), 1.18 – 1.16 (m, 6H), 0.81 (t, J = 7.4 Hz, 3H) ppm;

^{13}C NMR (101 MHz, acetone-d_6) δ 168.9, 163.4, 149.1, 145.1, 144.0, 141.8, 136.9, 136.2, 135.8, 134.1, 128.6, 127.0, 125.9, 124.0, 120.6, 110.7, 96.1, 93.1, 78.2, 75.6, 75.2, 72.9, 71.3, 70.7, 67.6, 55.0, 53.2, 52.2, 45.0, 42.2, 37.2, 28.9, 28.2, 26.5, 20.5, 17.8, 17.4, 15.3, 13.8, 11.2 ppm;

IR (thin film): ν 3375m, 2925m, 2854w, 1700s, 1639m, 1598m, 1508w, 1459m, 1378m, 1312s, 1244m, 1206m, 1159s, 1144s, 1080s, 1061s, 986s, 897w, 874m, 829w, 764m, 711w, 604w, 522w cm^{-1};

HRMS (APCI) for $C_{42}H_{63}O_{11}N_2^+$ $[M+H]^+$: calculated: 771.44264; found: 771.44220.

ERJ-153

Preparation of Amine 42

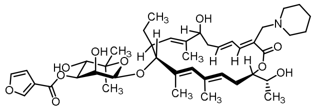

To an HPLC vial was added OP-1118 (**39**, 40.0 mg, 40.5 µmol, 1.0 eq.) and $Pd(PPh_3)_4$ (4.7 mg, 4.1 µmol, 10 mol%). The vial was capped, evacuated and backfilled with nitrogen three times and then dry MeOH (0.21 mL) was added, followed by piperidine (12.1 µL, 0.122 mmol, 3.0 eq.). The reaction was heated under stirring to 40 °C. Reaction progress was monitored by UHPLC-MS. After 17 h, the solvent was removed under a stream of nitrogen, followed by drying under high vacuum. Under ambient atmosphere Shimada's catalyst (4.7 mg, 20 µmol, 50 mol%) was added and the crude mixture was dissolved in dry 1,4-dioxane (0.21 mL) and DIPEA (49.4 µL, 0.284 mmol, 7.0 eq.). To the stirred mixture was added 3-furoyl chloride (38.2 mg, 0.203 mmol, 5.0 eq.) at 40 °C, the vial was capped and heated under stirring to 40 °C. Reaction progress was monitored by UHPLC-MS. After 90 min, DIPEA (14.1 µL, 0.0810 mmol, 2.0 eq.) and 3-furoyl chloride (15.3 mg, 0.0810 mmol, 2.0 eq.) were added. After 16 h, the reaction mixture was cooled to RT, quenched by addition of a drop of MeOH and concentrated *in vacuo*. The residue was dissolved in MeCN (2 mL) and eluted through a C18 SPE cartridge with MeCN (8 mL). The filtrate was concentrated *in vacuo* and the crude mixture purified by preparative RP-HPLC ([Gemini NX, C18, 5 µ, 110 Å, 250 mm × 21.2 mm, solvent A: H_2O + 0.1% HCOOH, solvent B: MeCN + 0.1% HCOOH, 20 mL/min; LC time program (min – %B): 5 min – 2%, 7 min – 15%, 50 min – 25%]) to yield, after concentration *in vacuo*, the piperidine **42** (t_R = 16.5 min, 4.3 mg, 5.7 µmol, 14%) as an off-white solid.

Analytical Data of Amine 42

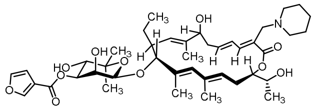

^{1}H NMR (500 MHz, acetone-d_6) δ 8.22 (d, J = 1.5 Hz, 1H), 7.66 (t, J = 1.7 Hz, 1H), 7.13 (d, J = 11.3 Hz, 1H), 6.80 (d, J = 1.8 Hz, 1H), 6.62 – 6.56 (m, 1H), 5.90 (s, 1H), 5.89 – 5.85 (m, 1H), 5.67 – 5.62 (m, 1H), 5.20 (dt, J = 10.6, 1.6 Hz, 1H), 4.94 (dd, J = 10.2, 3.1 Hz, 1H), 4.83 (s, 1H), 4.70 (td, J = 6.3, 4.3 Hz, 1H), 4.24 (d, J = 4.0 Hz, 1H), 4.14 (dd, J = 3.0, 1.2 Hz, 1H), 4.04 (p, J = 6.2 Hz, 1H), 3.90 (d, J = 10.3 Hz, 1H), 3.78 (d, J = 9.8 Hz, 1H), 3.40 – 3.35 (m, 1H), 3.22 – 3.15 (m, 1H), 2.78 (dt, J = 14.2, 7.1 Hz, 1H), 2.70 – 2.59 (m, 2H), 2.52 – 2.40 (m, 3H), 2.10 – 2.07 (m, 3H), 1.98 – 1.89 (m, 1H), 1.82 (s, 3H), 1.78 (s, 3H), 1.65 (s, 3H), 1.55 – 1.49 (m, 4H), 1.40 (p, J = 5.7 Hz, 2H), 1.32 – 1.28 (m, 1H), 1.26 (s, 3H), 1.17 (t, J = 3.1 Hz, 6H), 0.82 (t, J = 7.4 Hz, 3H) ppm;

^{13}C NMR (126 MHz, acetone-d_6) δ 168.9, 163.2, 149.1, 145.1, 143.8, 141.5, 136.9, 136.2, 135.8, 134.1, 128.7, 127.0, 126.3, 124.0, 120.6, 110.7, 96.1, 93.1, 78.2, 75.6, 75.2, 72.9, 71.3, 70.7, 67.7, 54.8, 54.3, 42.2, 37.2, 28.9, 28.4, 26.5, 26.5, 24.9, 20.5, 17.8, 17.4, 15.3, 13.8,
11.2 ppm;

IR (thin film): ν 3676m, 3449w, 2988s, 2901s, 1701w, 1406m, 1394m, 1250m, 1160m, 1066s, 1057s, 892m, 514w, 474w cm^{-1};

HRMS (ESI) for $C_{42}H_{62}O_{11}N^{+}$ $[M+H]^{+}$: calculated: 756.43174; found: 756.43160.

ERJ-188

Preparation of Amine 43

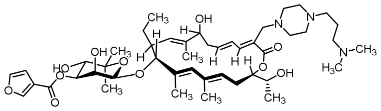

To an HPLC vial was added OP-1118 (**90**, 30.0 mg, 30.4 µmol, 1.0 eq.) and $Pd(PPh_3)_4$ (3.5 mg, 3.0 µmol, 10 mol%). The vial was capped, evacuated and backfilled with nitrogen three times and then dry MeOH (0.16 mL) was added, followed by *N*,*N*-dimethyl-3-(piperazin-1-yl)propan-1-amine (15.6 mg, 91.2 µmol, 3.0 eq.). The reaction was heated under stirring to 40 °C. Reaction progress was monitored by UHPLC-MS. After 19 h, the solvent was removed under a stream of nitrogen, followed by drying under high vacuum. Under ambient atmosphere Shimada's catalyst (3.5 mg, 15 µmol, 50 mol%) was added and the crude mixture was dissolved in dry 1,4-dioxane (0.16 mL) and DIPEA (47.7 µL, 0.274 mmol, 9.0 eq.). To the stirred mixture was added 3-furoyl chloride (40.1 mg, 0.213 mmol, 7.0 eq.) at 40 °C, the vial was capped and heated under stirring to 40 °C. Reaction progress was monitored by UHPLC-MS. After 2 h, the reaction mixture was cooled to RT, quenched by addition of a drop of MeOH and concentrated under *in vacuo*. The residue was dissolved in MeCN (2 mL) and eluted through a C18 SPE cartridge with MeCN (8 mL). The filtrate was concentrated *in vacuo* and the crude mixture purified by preparative RP-HPLC ([Gemini NX, C18, 5 µ, 110 Å, 250 mm 21.2 mm, solvent A: H_2O + 0.1% HCOOH, solvent B: MeCN + 0.1% HCOOH, 20 mL/min; LC time program (min – %B): 5 min – 2%, 7 min – 11%, 50 min – 16%]) to yield, after concentration *in vacuo*, the piperazine **43** (t_R = 28.9 min, 10.7 mg, 12.8 µmol, 40%) as an off-white solid.

Analytical Data of Amine 43

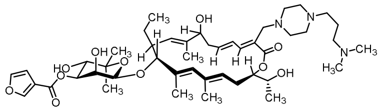

^{1}H NMR (500 MHz, acetone-d_6) δ 8.22 (d, *J* = 1.6 Hz, 1H), 7.66 (t, *J* = 1.8 Hz, 1H), 7.14 (d, *J* = 11.4 Hz, 1H), 6.80 (d, *J* = 1.9 Hz, 1H), 6.58 (dd, *J* = 15.1, 11.4 Hz, 1H), 5.93 – 5.83 (m, 2H), 5.67 – 5.60 (m, 2H), 5.20 (d, *J* = 10.5 Hz, 1H), 4.94 (dd, *J* = 10.2, 3.1 Hz, 1H), 4.83 (s, 1H), 4.27 – 4.21 (m, 1H), 4.15 (d, *J* = 3.1 Hz, 1H), 4.02 (p, *J* = 6.5, 6.0 Hz, 4H), 3.90 (d, *J* = 10.2 Hz, 1H), 3.78 (d, *J* = 9.7 Hz, 1H), 3.37 (d, *J* = 12.6 Hz, 1H), 3.18 (d, *J* = 12.6 Hz, 1H), 2.78 (dt, *J* = 14.1, 7.0 Hz, 1H), 2.70 – 2.61 (m, 2H), 2.60 – 2.54 (m, 1H), 2.53 – 2.40 (m, 12H), 2.38 (s, 6H), 1.97 – 1.89 (m, 1H), 1.81 (s, 3H), 1.77 (s, 3H), 1.72 (t, *J* = 7.3 Hz, 1H), 1.65 (s, 3H), 1.30 – 1.20 (m, 5H), 1.18 – 1.16 (m, 6H), 0.82 (t, *J* = 7.4 Hz, 3H) ppm;

^{13}C NMR (126 MHz, acetone-d_6) δ 168.9, 163.3, 149.1, 145.1, 143.8, 141.5, 136.9, 136.2, 135.8, 134.1, 128.7, 126.9, 126.3, 124.0, 120.5, 110.7, 96.1, 93.1, 78.1, 75.6, 75.2, 72.9, 71.3, 70.7, 67.8, 57.4, 56.5, 55.0, 53.6, 53.4, 49.7, 44.3, 42.1, 37.2, 28.9, 28.4, 26.5, 24.1, 20.6, 17.8, 17.4, 15.2, 13.8, 11.2 ppm;

IR (thin film): ν 3378w, 2933m, 2874w, 2825w, 1702s, 1639m, 1599m, 1507w, 1461m, 1402w, 1377m, 1312s, 1249m, 1207m, 1158s, 1080s, 1062s, 1004s, 988s, 897w, 874m, 827w, 764m, 711w, 604w, 523w, 464w cm^{-1};

HRMS (APCI) for $C_{46}H_{72}O_{11}N_3^+$ $[M+H]^+$: calculated: 842.51614; found: 842.51582.

ERJ-157

Preparation of Amine 44

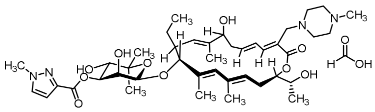

To an HPLC vial under ambient atmosphere was added **40** (20.0 mg, 29.5 µmol, 1.0 eq.) and Shimada's catalyst (3.4 mg, 15 µmol, 50 mol%). The crude mixture was dissolved in dry 1,4-dioxane (0.16 mL) and DIPEA (25.7 µL, 0.148 mmol, 5.0 eq.). To the stirred mixture was added 1-methyl-1*H*-pyrazole-3-carbonyl chloride (14.9 mg, 0.103 mmol, 3.5 eq.) at 40 °C, the vial was capped and heated under stirring to 40 °C. Reaction progress was monitored by UHPLC-MS. After 24 h the reaction mixture was cooled to RT, quenched by addition of a drop of MeOH and concentrated *in vacuo*. The residue was dissolved in MeCN (2 mL) and eluted through a C18 SPE cartridge with MeCN (8 mL). The filtrate was concentrated *in vacuo* and the crude mixture purified by preparative RP-HPLC ([Gemini NX, C18, 5 µ, 110 Å, 250 mm × 21.2 mm, solvent A: H_2O + 0.1% HCOOH, solvent B: MeCN + 0.1% HCOOH, 20 mL/min; LC time program (min – %B): 5 min – 2%, 7 min – 11%, 50 min – 16%]) to yield, after concentration *in vacuo*, the formate salt of the *N*-methylpiperazine **44** (t_R = 30.0 min, 12.0 mg, 14.5 µmol, 49%) as an off-white solid.

Analytical Data of Amine 44

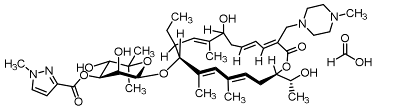

^{1}H NMR (500 MHz, acetone-d_6) δ 8.22 (s, 1H), 7.68 (dd, J = 6.2, 2.3 Hz, 1H), 7.14 (d, J = 11.4 Hz, 1H), 6.77 (d, J = 2.3 Hz, 1H), 6.58 (ddd, J = 15.1, 11.2, 1.7 Hz, 1H), 5.90 (d, J = 3.1 Hz, 1H), 5.90 – 5.84 (m, 1H), 5.65 (d, J = 8.5 Hz, 1H), 5.21 (dt, J = 10.5, 1.6 Hz, 1H), 4.97 (dd, J = 10.3, 3.1 Hz, 1H), 4.85 (d, J = 1.3 Hz, 1H), 4.71 (td, J = 6.2, 4.2 Hz, 1H), 4.24 (d, J = 4.2 Hz, 1H), 4.16 (dd, J = 3.1, 1.2 Hz, 1H), 4.02 (p, J = 6.3 Hz, 1H), 3.96 (d, J = 2.3 Hz, 3H), 3.94 (d, J = 10.3 Hz, 1H), 3.79 (d, J = 9.8 Hz, 1H), 3.38 (d, J = 12.7 Hz, 1H), 3.18 (d, J = 12.6 Hz, 1H), 2.78 (dt, J = 14.0, 6.9 Hz, 1H), 2.65 (dtt, J = 18.4, 9.8, 4.5 Hz, 2H), 2.46 (dddd, J = 23.0, 13.6, 9.0, 4.3 Hz, 10H), 2.22 (d, J = 3.0 Hz, 3H), 1.96 – 1.89 (m, 1H), 1.82 (d, J = 1.3 Hz, 3H), 1.77 (d, J = 1.4 Hz, 3H), 1.65 (d, J = 1.4 Hz, 3H), 1.27 (s, 3H), 1.26 – 1.20 (m, 1H), 1.21 – 1.15 (m, 6H), 0.81 (t, J = 7.4 Hz, 3H) ppm;

^{13}C NMR (126 MHz, acetone-d_6) δ 168.9, 164.6, 162.5, 144.1, 143.8, 141.5, 136.9, 136.2, 135.9, 134.0, 132.5, 128.6, 126.9, 126.3, 124.0, 109.7, 96.2, 93.0, 78.2, 75.5, 75.3, 72.9, 71.3, 70.8, 67.8, 55.6, 53.5, 53.1, 45.8, 42.1, 39.7, 37.2, 30.1, 28.9, 28.4, 26.5, 20.6, 17.8, 17.4, 15.2, 13.8, 11.2 ppm;

IR (thin film): ν 3385w, 2970w, 2930w, 2855w, 2807w, 1698s, 1640w, 1602w, 1459w, 1365m, 1312m, 1293w, 1241s, 1207m, 1148s, 1082m, 1061s, 1007s, 975m, 898w, 824w, 809w, 767m, 714w, 697w, 619w, 588w, 561w, 523w, 477w cm^{-1};

HRMS (ESI) for $C_{42}H_{65}O_{10}N_4^+$ $[M+H]^+$: calculated: 785.46952; found: 785.46895.

ERJ-183

Preparation of Amine 45

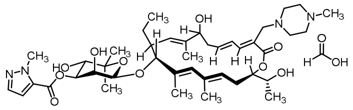

To an HPLC vial under ambient atmosphere was added **40** (20.0 mg, 29.5 µmol, 1.0 eq.) and Shimada's catalyst (3.4 mg, 15 µmol, 50 mol%). The crude mixture was dissolved in dry 1,4-dioxane (0.16 mL) and DIPEA (25.7 µL, 0.148 mmol, 5.0 eq.). To the stirred mixture was added 1-methyl-1*H*-pyrazole-5-carbonyl chloride (14.9 mg, 0.103 mmol, 3.5 eq.) at 40 °C, the vial was capped and heated under stirring to 40 °C. Reaction progress was monitored by UHPLC-MS. After 24 h the reaction mixture was cooled to RT, quenched by addition of a drop of MeOH and concentrated *in vacuo*. The residue was dissolved in MeCN (2 mL) and eluted through a C18 SPE cartridge with MeCN (8 mL). The filtrate was concentrated *in vacuo* and the crude mixture purified by preparative RP-HPLC ([Gemini NX, C18, 5 µ, 110 Å, 250 mm 21.2 mm, solvent A: H_2O + 0.1% HCOOH, solvent B: MeCN + 0.1% HCOOH, 20 mL/min; LC time program (min – %B): 5 min – 2%, 7 min – 11%, 50 min – 16%]) to yield, after concentration *in vacuo*, the formate salt of the *N*-methylpiperazine **45** (t_R = 32.8 min, 5.4 mg, 6.5 µmol, 22%) as an off-white solid.

Analytical Data of Amine 45

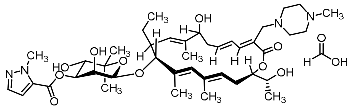

^{1}H NMR (500 MHz, acetone-d_6) δ 8.18 (s, 1H), 7.45 (d, *J* = 2.0 Hz, 1H), 7.13 (d, *J* = 11.4 Hz, 1H), 6.91 (d, *J* = 2.0 Hz, 1H), 6.62 – 6.54 (m, 1H), 5.92 – 5.89 (m, 2H), 5.89 – 5.83 (m, 1H), 5.67 – 5.62 (m, 1H), 5.21 (dt, *J* = 10.5, 1.5 Hz, 1H), 5.03 (dd, *J* = 10.2, 3.1 Hz, 1H), 4.86 (d, *J* = 1.2 Hz, 1H), 4.71 (td, *J* = 6.1, 4.2 Hz, 1H), 4.26 – 4.23 (m, 1H), 4.17 – 4.14 (m, 5H), 4.01 (p, *J* = 6.3 Hz, 1H), 3.94 (d, *J* = 10.2 Hz, 1H), 3.79 (d, *J* = 9.8 Hz, 1H), 3.36 (d, *J* = 12.6 Hz, 1H), 3.16 (d, *J* = 12.6 Hz, 1H), 2.77 (dt, *J* = 13.9, 6.8 Hz, 1H), 2.70 – 2.60 (m, 3H), 2.46 (m, 2H), 2.29 (d, *J* = 11.7 Hz, 1H), 2.17 (s, 3H), 1.96 – 1.91 (m, 1H), 1.82 (d, *J* = 1.3 Hz, 3H), 1.78 – 1.76 (m, 3H), 1.66 – 1.64 (m, 3H), 1.31 – 1.28 (m, 4H), 1.27 (s, 3H), 1.26 – 1.21 (m, 1H), 1.19 (s, 3H), 1.19 – 1.17 (m, 4H), 0.82 (t, *J* = 7.5 Hz, 3H) ppm;

^{13}C NMR (126 MHz, acetone-d_6) δ 168.9, 164.3, 160.1, 143.7, 141.4, 138.2, 136.9, 136.1, 135.9, 134.1, 128.7, 126.9, 126.5, 124.0, 112.3, 96.1, 93.1, 78.1, 76.2, 75.2, 72.9, 71.3, 70.7, 67.9, 55.8, 53.6, 53.4, 46.1, 42.1, 40.0, 37.2, 28.9, 28.5, 26.5, 20.7, 17.8, 17.4, 15.2, 13.8,
11.2 ppm;

IR (thin film): ν 2971m, 2933m, 2856m, 2808w, 1996w, 1701s, 1640m, 1602w, 1516w, 1459m, 1377m, 1320m, 1293m, 1248s, 1207m, 1163m, 1124s, 1083m, 1061s, 1007m, 978m, 932w, 898m, 811w, 765m, 708w, 653w, 557w, 524w, 490w, 456w cm^{-1};

HRMS (ESI) for $C_{42}H_{65}O_{10}N_4^+$ $[M+H]^+$: calculated: 785.46952; found: 785.46927.

ERJ-184

Preparation of Amine 46

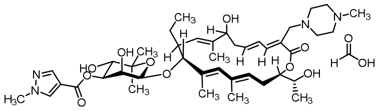

The formate salt **40** (40 mg) was partitioned between DCM (2 mL) and a sodium bicarbonate solution (sat. aq., 2 mL). The organic layer was separated, and the aqueous layer was extracted with DCM (2 × 2 mL), The combined organic layers were dried over sodium sulfate, filtered, and concentrated in vacuo to obtain the free amine of **40** (33.0 mg, 48.8 µmol).

To an HPLC vial under ambient atmosphere was added the free amine obtained from **40** (33.0 mg, 48.8 µmol, 1.0 eq.) and Shimada's catalyst (11.2 mg, 48.8 µmol, 100 mol%). The mixture was dissolved in dry 1,4-dioxane (140 µL) and DIPEA (43 µL, 0.24 mmol, 5.0 eq.) and cooled to 0 °C. To the stirred mixture was added a solution 1-methyl-1*H*-pyrazole-4-carbonyl chloride (24.7 mg, 0.171 mmol, 3.5 eq.) in dry 1,4-dioxane at 40 °C, the vial was capped and heated under stirring to 40 °C. Reaction progress was monitored by UHPLC-MS. After 24 h the reaction mixture was cooled to RT, quenched by addition of a drop of MeOH and concentrated *in vacuo*. The residue was dissolved in MeCN (2 mL) and eluted through a C18 SPE cartridge with MeCN (8 mL). The filtrate was concentrated *in vacuo* and the crude mixture purified by preparative RP-HPLC ([Gemini NX, C18, 5 µ, 110 Å, 250 mm 21.2 mm, solvent A: H_2O + 0.1% HCOOH, solvent B: MeCN + 0.1% HCOOH, 18 mL/min; LC time program (min – %B): 5 min – 10%, 60 min – 30%]) to yield, after concentration *in vacuo*, the formate salt of the *N*-methylpiperazine **46** (t_R = 33.1 min, 14.3 mg, 17.2 µmol, 35%) as an off-white solid.

Analytical Data of Amine 46

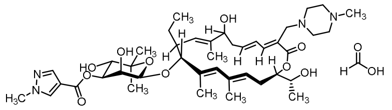

^{1}H NMR (500 MHz, acetone-d_6) δ 8.21 (s, 1H), 8.13 (s, 1H), 7.82 (s, 1H), 7.14 (d, J = 11.4 Hz, 1H), 6.58 (ddd, J = 14.9, 11.2, 1.7 Hz, 1H), 5.90 (s, 1H), 5.89 – 5.84 (m, 1H), 5.67 – 5.62 (m, 1H), 5.21 (dt, J = 10.5, 1.5 Hz, 1H), 4.91 (dd, J = 10.3, 3.1 Hz, 1H), 4.82 (d, J = 1.2 Hz, 1H), 4.71 (td, J = 6.1, 4.2 Hz, 1H), 4.26 – 4.22 (m, 1H), 4.13 (dd, J = 3.1, 1.2 Hz, 1H), 4.05 – 3.98 (m, 1H), 3.94 (s, 3H), 3.89 (d, J = 10.3 Hz, 1H), 3.78 (d, J = 9.8 Hz, 1H), 3.38 (d, J = 12.6 Hz, 1H), 3.18 (d, J = 12.6 Hz, 1H), 2.77 (dt, J = 14.1, 6.9 Hz, 1H), 2.70 – 2.60 (m, 2H), 2.45 (dddd, J = 22.3, 13.7, 9.1, 4.3 Hz, 10H), 2.22 (s, 3H), 1.92 (ddt, J = 13.0, 7.4, 3.7 Hz, 2H), 1.81 (d, J = 1.3 Hz, 3H), 1.77 (d, J = 1.4 Hz, 3H), 1.65 (d, J = 1.3 Hz, 3H), 1.26 (s, 3H), 1.24 – 1.19 (m, 1H), 1.18 (s, 3H), 0.82 (t, J = 7.5 Hz, 3H) ppm;

^{13}C NMR (126 MHz, acetone-d_6) δ 168.9, 164.5, 163.3, 143.8, 141.5, 141.4, 136.9, 136.2, 135.9, 134.8, 134.1, 128.7, 126.9, 126.3, 124.0, 115.7, 96.2, 93.1, 78.2, 75.2, 75.0, 73.0, 71.5, 70.9, 67.9, 55.6, 53.5, 45.8, 42.1, 39.3, 37.2, 28.9, 28.4, 26.5, 20.6, 17.8, 17.4, 15.2, 13.8, 11.2 ppm;

IR (thin film): ν 3405w, 2971w, 2934m, 2874w, 1699s, 1640w, 1601w, 1555m, 1456m, 1406m, 1377m, 1315m, 1294m, 1221s, 1162m, 1144m, 1119m, 1080m, 1062s, 1007s, 976m, 897m, 768m, 734m, 701w, 521w, 469w, 456w cm^{-1};

HRMS (ESI) for $C_{42}H_{65}O_{10}N_4^+$ $[M+H]^+$: calculated: 785.46952; found: 785.47006.

ERJ-186, ERJ-624

1.4 Isolation of Shunt Metabolite

Isolation of Shunt Metabolite FdxG2-*i*Bu (26)

FdxG2-*i*Bu (**26**) was isolated from cultures of *Actinoplanes deccanensis fdxG2*⁻ as described previously.[3]

1.5 Biological Experiments

1.5.1 General procedure for determination of RNAP inhibition

Protein expression and purification

M. tuberculosis RNAP core and RbpA were purified as described before.[16,17] The gene encoding for σ^A subunit was PCR-amplified from the *M. tuberculosis* H37Rv genomic DNA and cloned into pET28a under the *N*-terminal 6xHis tag. The Tobacco Etch Virus (TEV) protease site was inserted between the 6xHis tag and the σ^A gene. The 6xHis-tagged σ^A was expressed in *E. coli* BL21(DE3) and purified by Ni^{2+}-agarose affinity chromatography followed by TEV protease treatment and a second Ni^{2+}-agarose affinity chromatography.

Fluorescent transcription assay with UTPɣ-AmNS

Transcription assay was performed as described before with the following modifications.[18] Reactions were performed in 20 µl of 40 mM HEPES pH 8.0, 50 mM NaCl, 5 mM $MgCl_2$ and 20% DMSO (the assays tolerance for DMSO was established beforehand) in Nunc™ 384-Well black, Non-Treated, Flat-Bottom Microplate (*Thermo Scientific*™). 100 nM *M. tuberculosis* RNAP core, 500 nM σ^A and 500 nM RbpA were mixed and incubated for 5 min at 37 °C. Fidaxomicin and its derivatives in 20% DMSO were added at concentrations from 3 nM to 100 µM and incubated for 5 min at 37 °C. Then, 50 nM pGEM-T plasmid DNA carrying the *M. tuberculosis rrnA*P3 promoter was added and incubated at 37 °C for 10 min. Transcription was initiated by adding ATP, CTP, and GTP to a final concentration of 200 µM each and of 50 µM UTP γ-AmNS (*Jena Bioscience*). The microplate was transferred to a Spark 10M® (*TECAN*) fluorescence microplate reader. Transcription reactions were performed at 37 °C in triplicates. Fluorescence was measured every 10 min over 3 hours using $\lambda_{ex}/\lambda_{em}$ of 360/465 nm. For the negative control reactions, DNA was omitted. The positive control reactions were performed in 20% DMSO without inhibitor.

Analysis

Collected fluorescent values (F_i) were processed as follows. The recorded value of the first timepoint (time zero, F_0), was subtracted from each timepoint to get corrected values ($FC_i = F_i\text{-}F_0$). Next, for each timepoint (FC_i), the fluorescence value of the negative control reaction (FC_i^{DNA}) was subtracted and divided by the fluorescence value of the positive control reaction (FC_i^{DMSO}). Resulting normalized fluorescence values ($FC_i^N = (FC_i - FC_i^{DNA})/FC_i^{DMSO}$) recorded at 30 min of reaction were plotted as function of the inhibitor concentration. To calculate the half maximal inhibitory concentrations (IC_{50}) the FC_i^N values were fitted using non-linear regression with three parameters and shared top and bottom values for all data sets (GraphPad Prism 10.1.2).

1.5.2 General procedure for the determination of Inhibitory Concentration (IC_{90}) values of *M. tuberculosis*

IC_{90} determination was essentially conducted as described recently.[19] Briefly, the Green-Fluorescent Protein (GFP) expressing recombinant *Mycobacterium tuberculosis* H37Rv *rpsL*[20] transformed with pOLYG-Pr-GFP[21] was grown in Middlebrook 7H9-OADC with 0.05% Tween 80 until mid-log phase (optical density at 600 nm OD_{600} = 0.3 – 1.0), diluted to an OD_{600} of 0.04 and 20 µL of the suspension were added to an equal volume of 12-point 2-fold serial dilutions of the compounds in 7H9-OADC-Tween in 384-well plates in triplicates. Compound concentrations were in the range of 62.5 to 0.031 µg/mL. Fluorescence was measured immediately after inoculation (background) and after 10 days of incubation at 37 °C. Dose response curves were fitted with a 4-parameter log-normal model. P_{MIN} [-,-] and P_{MAX} [-, 120] are the minimum and the maximum, respectively, P_{Hill} [0,-] indicates the steepness, and EC_{50} [-,-] the log-back transformed Minimal Effective Concentration 50. The computational and statistical analysis was conducted with R (3.0.1 – 3.1.1; https://www.r-project.org/). Dose response curves were fitted with the ´drc´ package. The inhibitory potency I was calculated with the equation I = 100-[100(S-P)/(N-P)]. S is the sample's fluorescence while P and N derive from growth inhibition with the control drug (Kanamycin A) and solvent growth control measurements (DMSO 1.25% vol./vol.), respectively. A fluorescence reduction of 90% as compared to the no-drug control was reported as Inhibitory Concentration (IC_{90}).

1.5.3 General procedure for the determination of MIC values of a Rifampicin-resistant *M. tuberculosis* isolate

Preparation of stock solutions

Rifampicin was purchased from Sigma, Fidaxomicin was purchased from *BOC Sciences* or *Biosynth Carbosynth*. Stock solutions were prepared in suitable concentrations for the antibiotics/compounds to be tested, here 10 mg/ml solved in DMSO. The exact concentration was calculated based on the free base/active compound. Stock solutions were stored at -20 °C.

Preparation of bacterial cultures

M. tuberculosis (clinical isolate) rpoB mutant (S531L) and *M. tuberculosis* H37Rv GFP-reporter strain (poly-g-GFP, as comparator with rifampicin susceptible phenotype) were grown from frozen stock and passaged two times prior to the experiment. Cultures were grown until an OD_{600} of 0.3 was reached and subsequently diluted to final OD_{600} of 0.04.

Preparation of plates

A 96-well plate (*Greiner Bio-One*, U-bottom, CH) was prepared as followed: 90 µL of fresh 7H9 media was added to wells 2-12 and 177.7 µL 7H9 was added to well 1. Antibiotics/compounds were thawed at RT and 2.3 µL were added, at double the test concentration (125 µg/mL, final concentration 62.5 µg/mL). 90 µL were transferred from well 1 to well 2, mix by pipetting and in the similar matter continued till well 10. The last 90 µL were discarded. 90 µL of bacterial culture were added to wells 1-11. Experiment was carried out in biological triplicates. The plates were sealed and incubated for 14 days at 37 °C. After 14 days, turbidity was determined by eye. The lowest antibiotic concentration which prevented visible growth was recorded as the Minimal Inhibitory Concentration (MIC).

1.5.4 General procedure for the determination of MIC values of *C. difficile*

200 µL of a spore suspension of *C. difficile* 630 (DSM No. 27543; ATCC No. BAA-1382) was used for inoculation of 8 mL Brain Heart Infusion (BHI) (*Oxoid*; CM 1135) + 5 g/L yeast extract (*Roth* No. 2363.3) + 0.1% taurocholate (*Roth* No. 8149.3) and incubated overnight at 37 °C in an anaerobic workstation (95% N_2, 5% H_2; Whitley A35 HEPA by *Meintrup DWS*). 10 µL of the culture were used to start a second culture in BHI + 5 g/L yeast extract + 0.1% cysteine (*Thermo Scientific*; J63745.22) (freshly prepared). This second culture and a dilution series of it (five times dilution of 1:5) were incubated anaerobically at 37 °C overnight. This overnight culture not exceeding an OD_{600} of 2 was diluted down to an OD_{600} of 0.1 with BHI-S (BHI + 5 g/L yeast extract + 0.4% cysteine, pH 7.2) and 100 µL of it used to inoculate 96 well microtiter plates (MTP) (*TPP Switzerland*; Nr.92096) which were beforehand prepared as follows: Each substance to be tested was dissolved in DMSO (*Sigma*; D8418; for molecular biology) to 10 mg/mL and diluted in BHI-S to obtain a substance concentration of 64 µg/mL. To enhance solubility, the substances ERJ-714 and ERJ-715 were dissolved in DMSO to 5 mg/mL before dilution in BHI-S to a concentration of 64 µg/mL. 200 µL of this solution was used to load the first row of wells of the MTP. Using a multichannel pipette 100 µL were taken from first wells and loaded into second wells that were already provided with 100 µL BHI-S; 100 µL of second wells were provided into third wells that were already provided with 100 µL BHI-S, and so on until the last row of wells of the MTP; 100 µL of the last wells with substance concentrations of 0.125 µg/mL were discarded. Final culture volumes were thus 200 µL and the tested concentration range of substance 0.0625 µg/mL to 32 µg/mL. 200 µL of BHI-S served as negative control. 100 µL BHI-S + 100 µL of *C. difficile* culture without substance served as positive growth control (final OD_{600} of 0.6 – 0.7). MTPs were incubated anaerobically overnight at 37 °C and OD_{600} determined next day using a plate reader (*BioTek*; SynergyMx). Each substance unless specified was tested in at least three biological replicates each with two technical replicates. Substance concentrations suppressing growth to $OD_{600} < 0.15$ were reported as MIC.

1.5.5 Inhibition zone determination assay with *C. crescentus*

All the *C. crescentus* strains were cultivated in peptone-yeast extract medium (PYE) and incubated at 30 °C under aerobic conditions. All plasmids were maintained with 1 µg/ml of gentamicin (*Gibco*, #15710-049) in the media and induced with 0.5 mM IPTG (Isopropyl b-D-thiogalactoside, *Biosolve chimie*, #0016242362BS). Inhibition assay was carried out in a 14 cm diameter petri dish with 50 mL of 1.5% agar PYE, with an overlay of 12 mL of 0.375% agar PYE supplemented with 600 µL of overnight culture of the strain of interest, appropriate antibiotics and inducer. After polymerisation, paper disks (*Bio-rad* #1660468EDU) with 20 µg of each selected compound dissolved in DMSO were placed on the surface. After 24 h of incubation at 30 °C, the area with no visible growth was measured using ImageJ (version: Java 1.8.0_345 64bit).

1.6 Molecular Modelling

Modelling was based on the cryo-EM structure reported by Ebright and co-workers (PDB: 6FBV).[22] The structure of derivatives was built and posed manually by modifying the existing fidaxomicin ligand and then energy-minimised in moloc (Gerber molecular Design) using the MAB force field with stationary macrocycle and protein.[23]

1.7 References

[1] I. Stary, P. Kocovsky, *J. Am. Chem. Soc.* **1989**, *111*, 4981–4982.

[2] H. Hattori, E. Kaufmann, H. Miyatake-Ondozabal, R. Berg, K. Gademann, *J. Org. Chem.* **2018**, *83*, 7180–7205.

[3] E. Jung, M. Hunter, A. Dorst, A. Major, T. Teofilovic, R. Müller, K. Gademann, *Helv. Chim. Acta* **2024**, e202400013.

[4] H. E. Gottlieb, V. Kotlyar, A. Nudelman, *J. Org. Chem.* **1997**, *62*, 7512–7515.

[5] N. Srinivasan, A. Yurek-George, A. Ganesan, *Mol. Divers.* **2005**, *9*, 291–293.

[6] M. Wei, D. Liang, X. Cao, W. Luo, G. Ma, Z. Liu, L. Li, *Angew. Chem. Int. Ed.* **2021**, *60*, 7397–7404.

[7] N. J. Liverton, R. A. Bednar, B. Bednar, J. W. Butcher, C. F. Claiborne, D. A. Claremon, M. Cunningham, A. G. DiLella, S. L. Gaul, B. E. Libby, E. A. Lyle, J. J. Lynch, J. A. McCauley, S. D. Mosser, K. T. Nguyen, G. L. Stump, H. Sun, H. Wang, J. Yergey, K. S. Koblan, *J. Med. Chem.* **2007**, *50*, 807–819.

[8] M. Walter, Y. von Coburg, K. Isensee, K. Sander, X. Ligneau, J.-C. Camelin, J.-C. Schwartz, H. Stark, *Bioorg. Med. Chem. Lett.* **2010**, *20*, 5879–5882.

[9] J. J. Crawford, D. F. Ortwine, B. Wei, W. B. Young, *Heteroaryl Pyridone and Aza-Pyridone Compounds as Inhibitors of Btk Activity*, **2013**, WO2013067274A1.

[10] K.-C. Liu, J.-M. Fang, J.-T. Jan, T.-J. R. Cheng, S.-Y. Wang, S.-T. Yang, Y.-S. E. Cheng, C.-H. Wong, *J. Med. Chem.* **2012**, *55*, 8493–8501.

[11] L. C. King, G. K. Ostrum, *J. Org. Chem.* **1964**, *29*, 3459–3461.

[12] M. Kücükdisli, H. Bel-Abed, D. Cirillo, W.-T. Lo, N.-L. Efrém, A. Horatscheck, L. Perepelittchenko, P. Prokofeva, T. A. L. Ehret, S. Radetzki, M. Neuenschwander, E. Specker, G. Médard, S. Müller, S. Wilhelm, B. Kuster, J. P. von Kries, V. Haucke, M. Nazaré, *J. Med. Chem.* **2023**, *66*, 14278–14302.

[13] C. J. Smedley, J. A. Homer, T. L. Gialelis, A. S. Barrow, R. A. Koelln, J. E. Moses, *Angew. Chem. Int. Ed.* **2022**, *61*, e202112375.

[14] N. Shimada, Y. Nakamura, T. Ochiai, K. Makino, *Org. Lett.* **2019**, *21*, 3789–3794.

[15] D. Dailler, A. Dorst, D. Schäfle, P. Sander, K. Gademann, *Commun. Chem.* **2021**, *4:59*.

[16] Z. Morichaud, S. Trapani, R. K. Vishwakarma, L. Chaloin, C. Lionne, J. Lai-Kee-Him, P. Bron, K. Brodolin, *Nat. Commun.* **2023**, *14*, 484.

[17] Y. Hu, Z. Morichaud, S. Chen, J.-P. Leonetti, K. Brodolin, *Nucleic Acids Res.* **2012**, *40*, 6547–6557.

[18] J. Bhat, R. Rane, S. M. Solapure, D. Sarkar, U. Sharma, M. N. Harish, S. Lamb, D. Plant, P. Alcock, S. Peters, S. Barde, R. K. Roy, *J. Biomol. Screen.* **2006**, *11*, 968–976.

[19] M. Dal Molin, P. Selchow, D. Schäfle, A. Tschumi, T. Ryckmans, S. Laage-Witt, P. Sander, *J. Mol. Med.* **2019**, *97*, 1601–1613.

[20] C. Raynaud, K. G. Papavinasasundaram, R. A. Speight, B. Springer, P. Sander, E. C. Böttger, M. J. Colston, P. Draper, *Mol. Microbiol.* **2002**, *46*, 191–201.

[21] U. Matt, P. Selchow, M. Dal Molin, S. Strommer, O. Sharif, K. Schilcher, F. Andreoni, A. Stenzinger, A. S. Zinkernagel, M. Zeitlinger, P. Sander, J. Nemeth, *Int. J. Antimicrob. Agents* **2017**, *50*, 55–62.

[22] W. Lin, K. Das, D. Degen, A. Mazumder, D. Duchi, D. Wang, Y. W. Ebright, R. Y. Ebright, E. Sineva, M. Gigliotti, A. Srivastava, S. Mandal, Y. Jiang, Y. Liu, R. Yin, Z. Zhang, E. T. Eng, D. Thomas, S. Donadio, H. Zhang, C. Zhang, A. N. Kapanidis, R. H. Ebright, *Mol. Cell* **2018**, *70*, 60–71.

[23] P. R. Gerber, K. Müller, *J. Comput. Aided Mol. Des.* **1995**, *9*, 251–268.

1.8 NMR Spectra

^{1}H NMR spectrum of **2** (400 MHz, acetone-d_6)

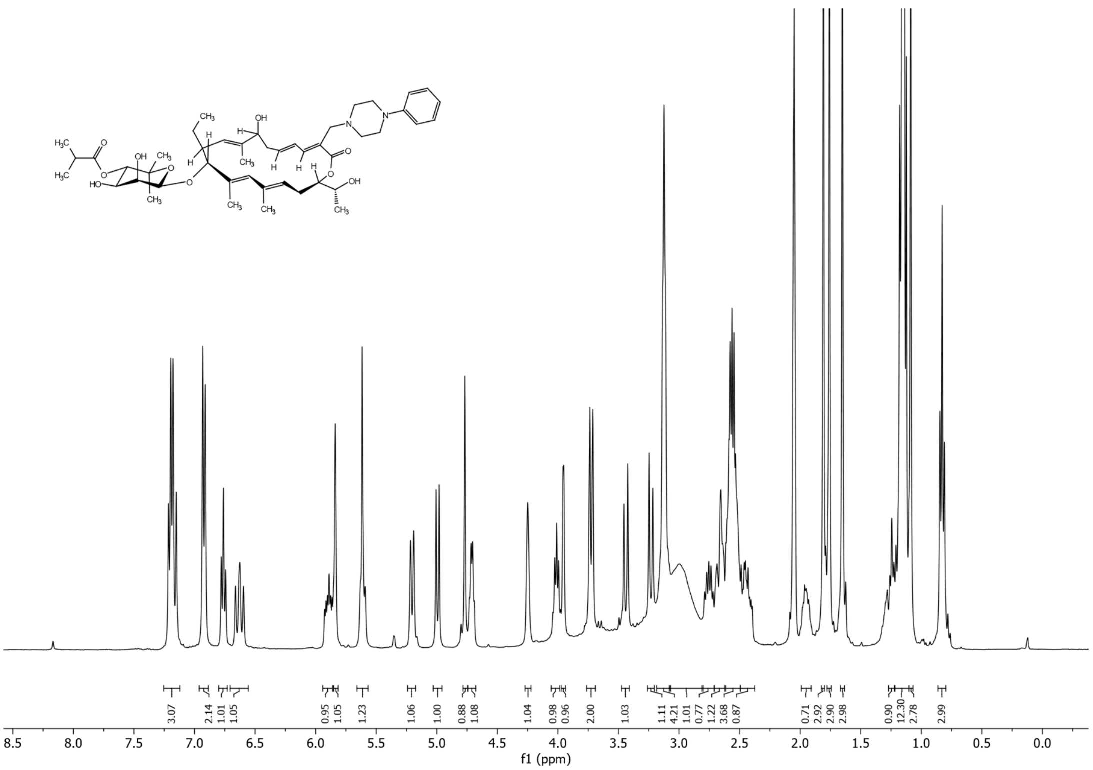

^{13}C NMR spectrum of **2** (126 MHz, acetone-d_6)

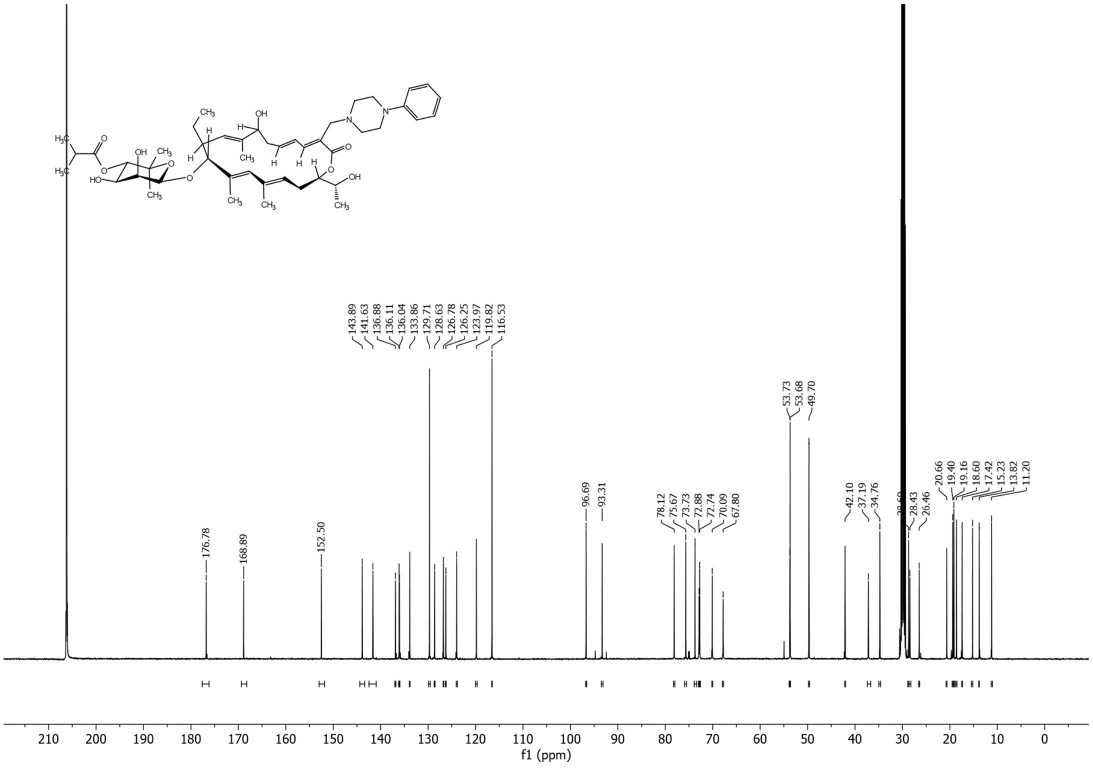

^{1}H NMR spectrum of **3** (400 MHz, acetone-d_6)

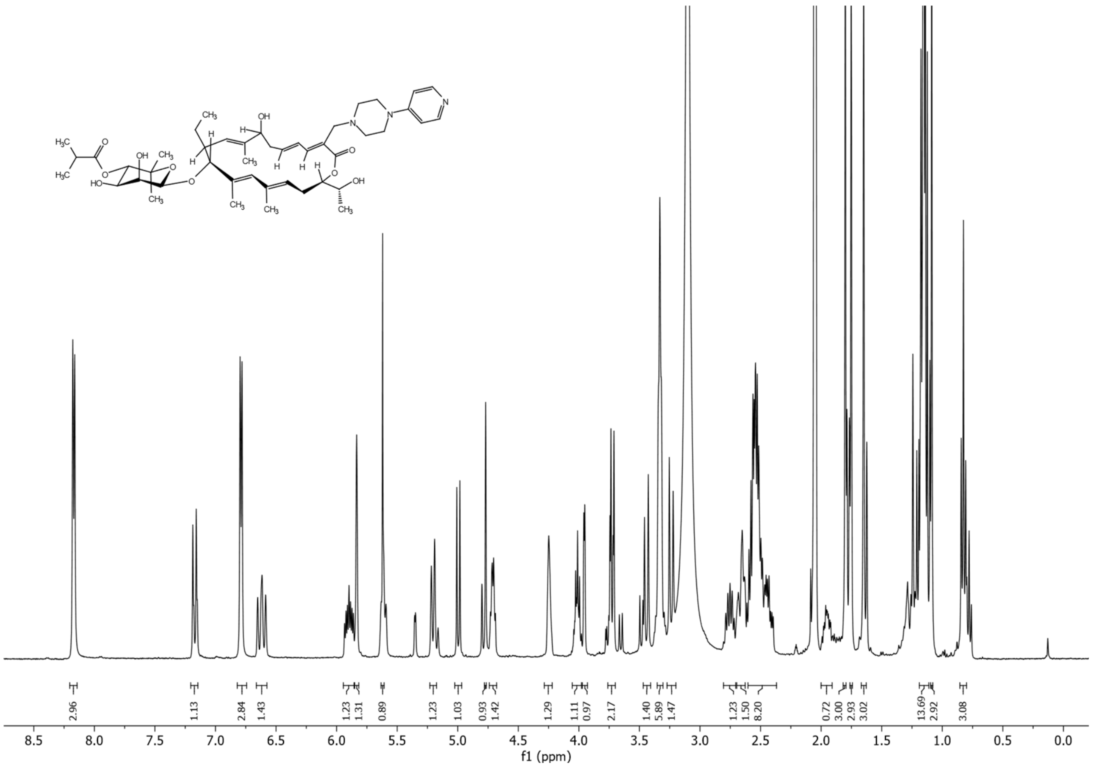

^{13}C NMR spectrum of **3** (126 MHz, acetone-d_6)

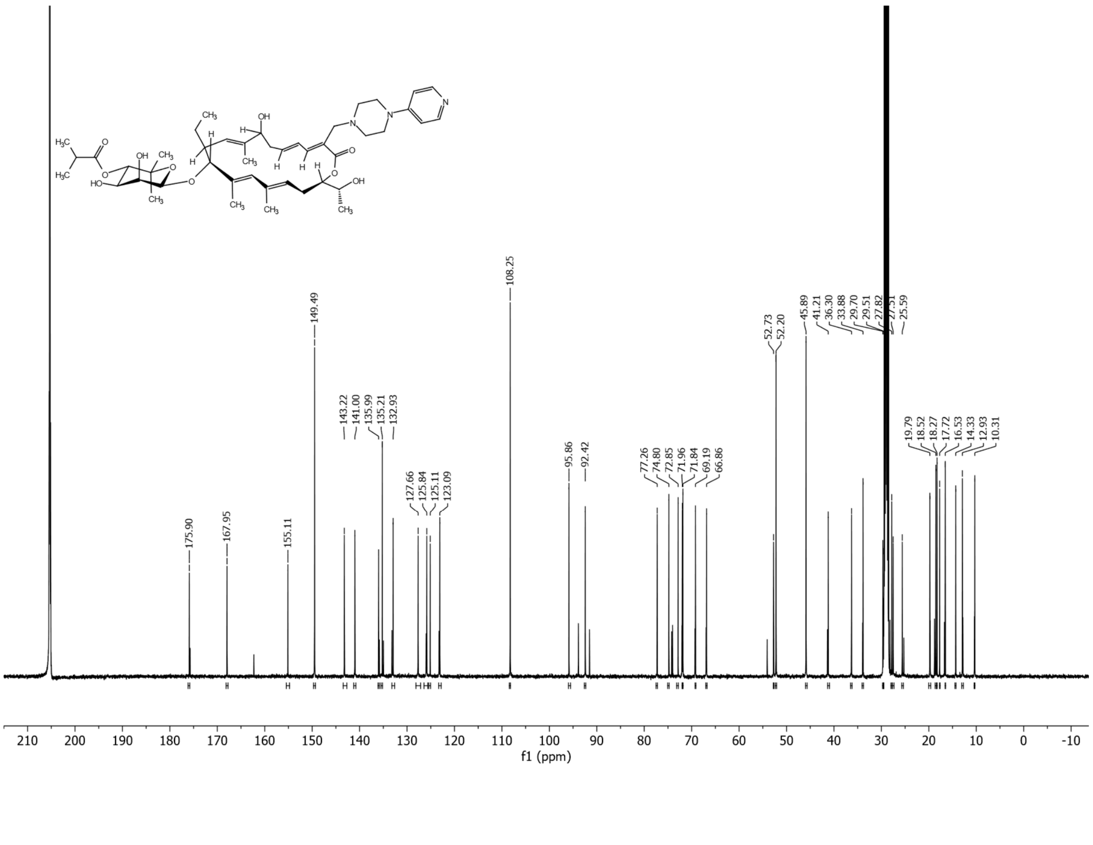

^{1}H NMR spectrum of **4** (500 MHz, acetone-d_6)

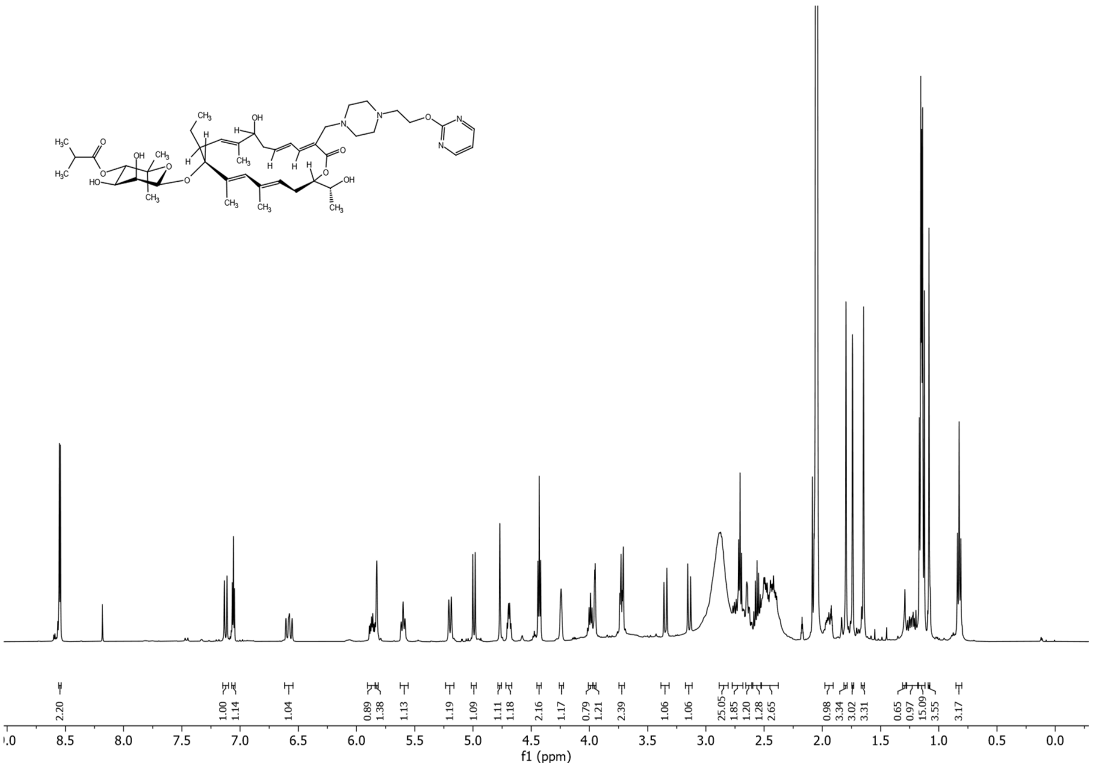

^{13}C NMR spectrum of **4** (126 MHz, acetone-d_6)

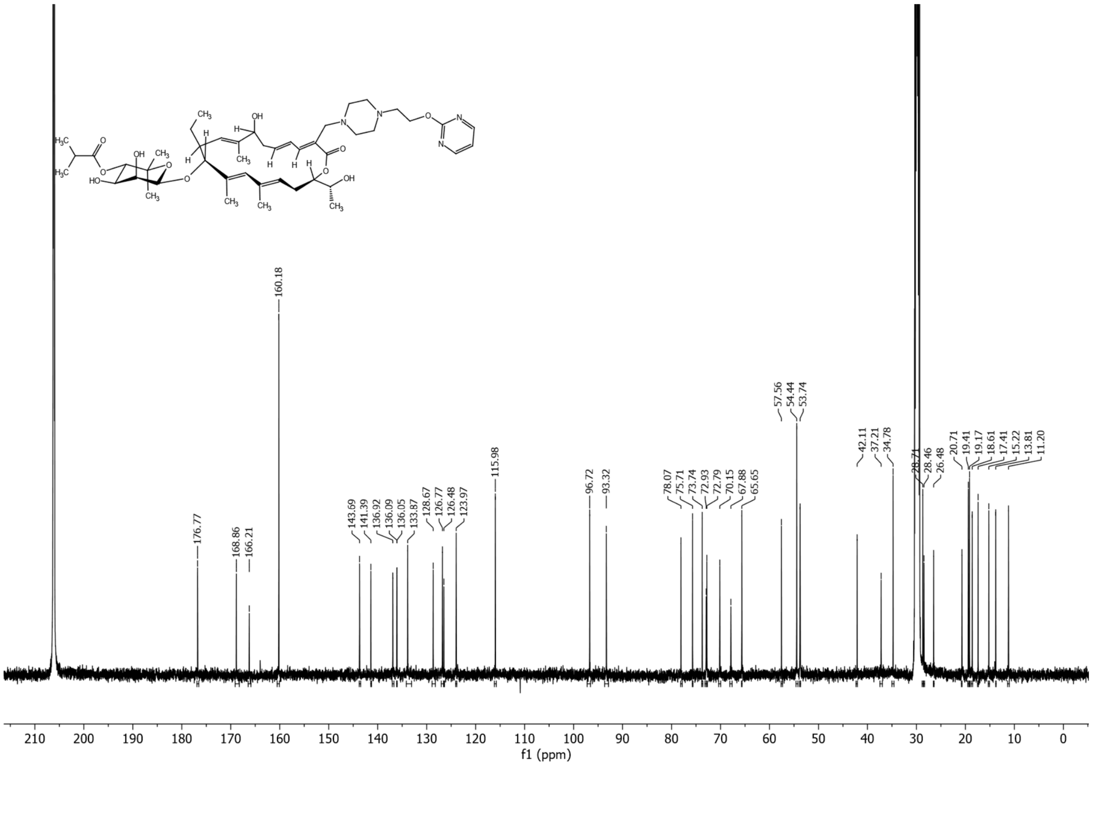

^{1}H NMR spectrum of **4a** (400 MHz, $CDCl_3$)

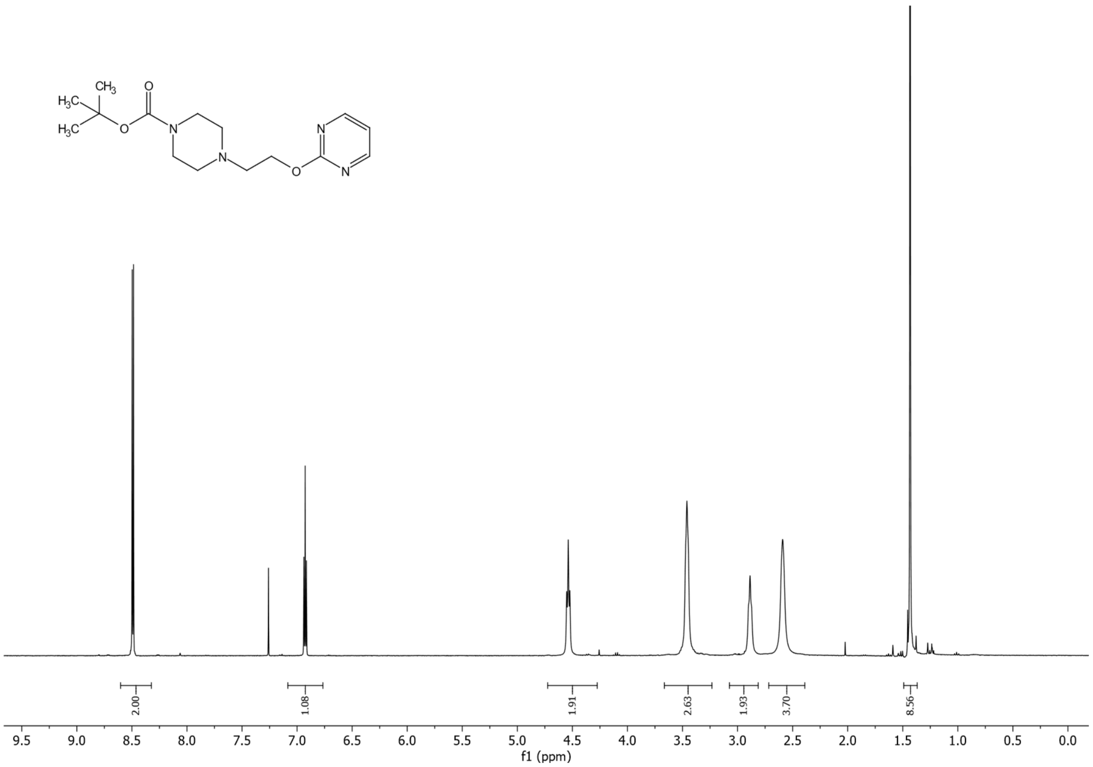

^{13}C NMR spectrum of **4a** (101 MHz, $CDCl_3$)

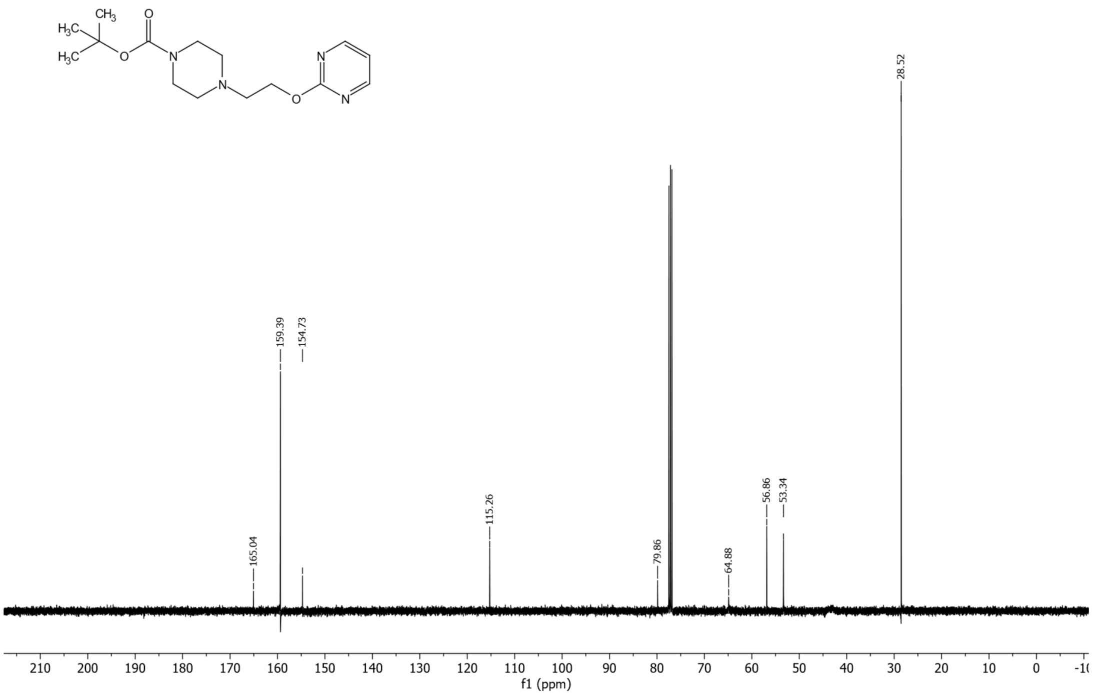

^{1}H NMR spectrum of **5** (500 MHz, acetone-d_6)

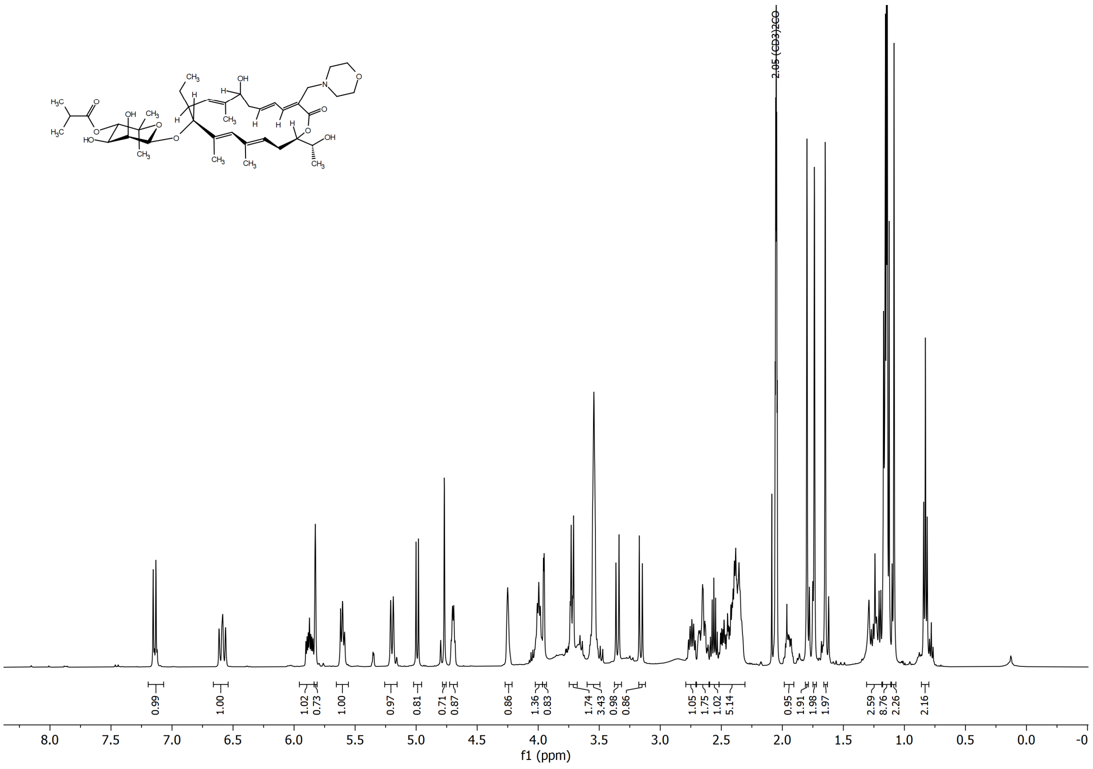

^{13}C NMR spectrum of **5** (126 MHz, acetone-d_6)

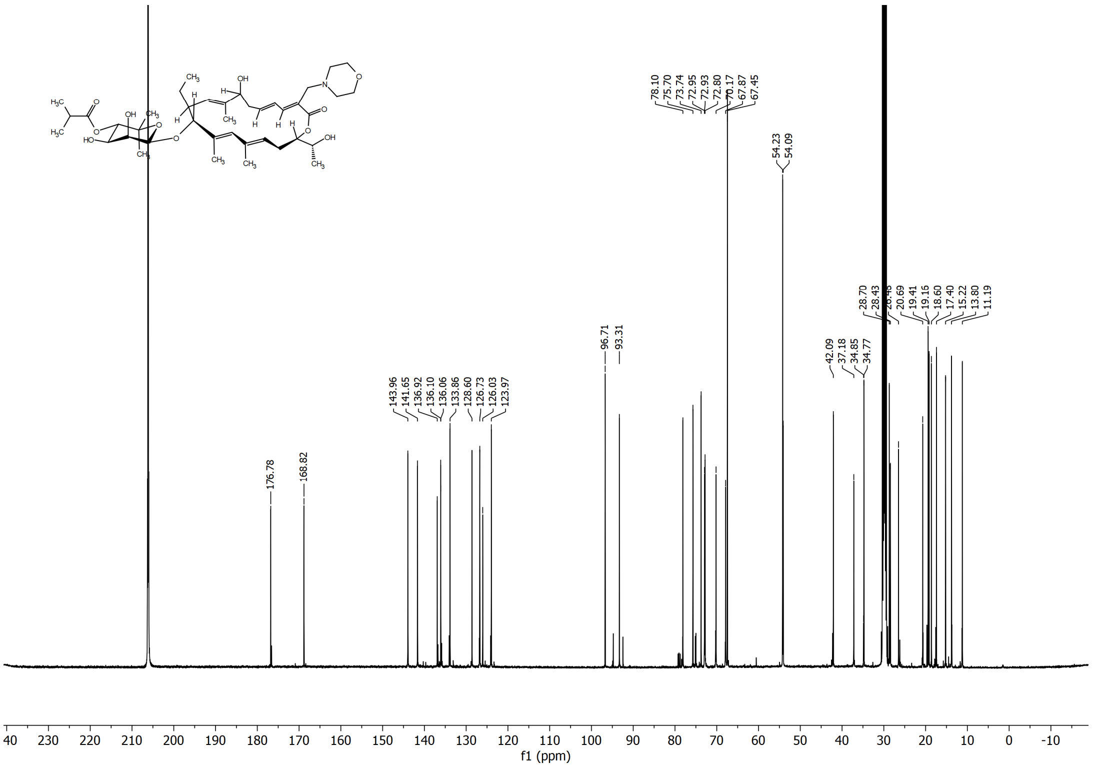

^{1}H NMR spectrum of **6** (400 MHz, acetone-d_6)

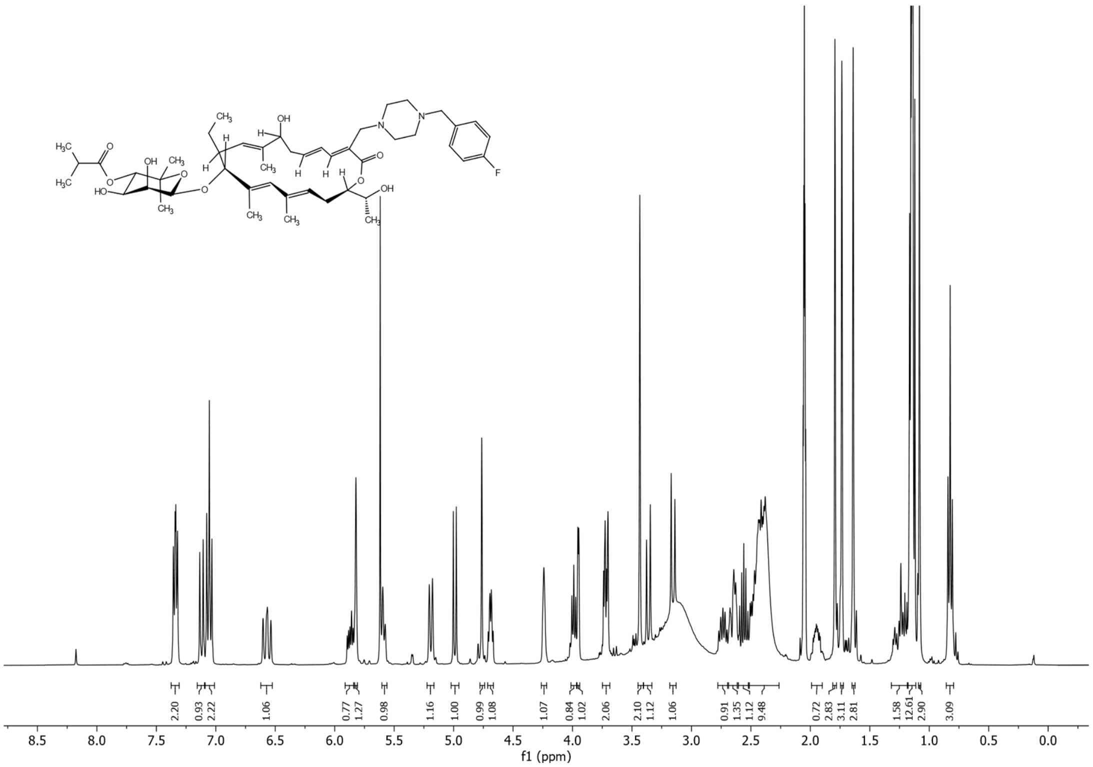

^{13}C NMR spectrum of **6** (126 MHz, acetone-d_6)

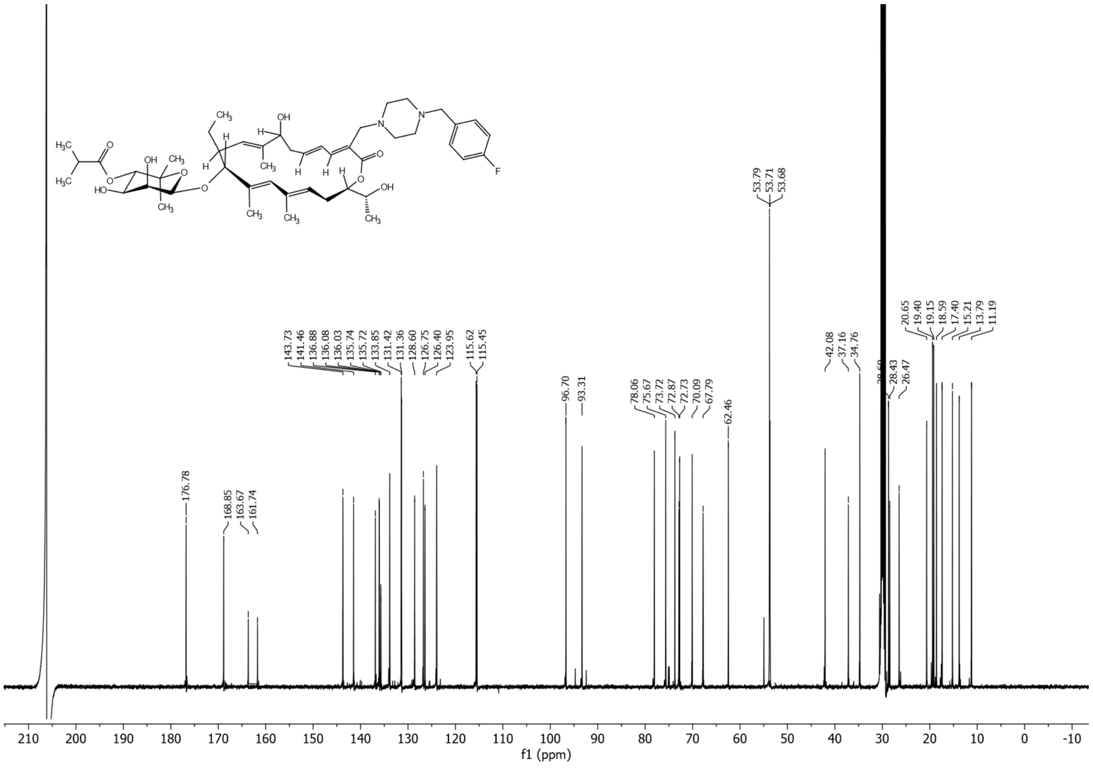

^{19}F NMR spectrum of **6** (376 MHz, acetone-d_6)

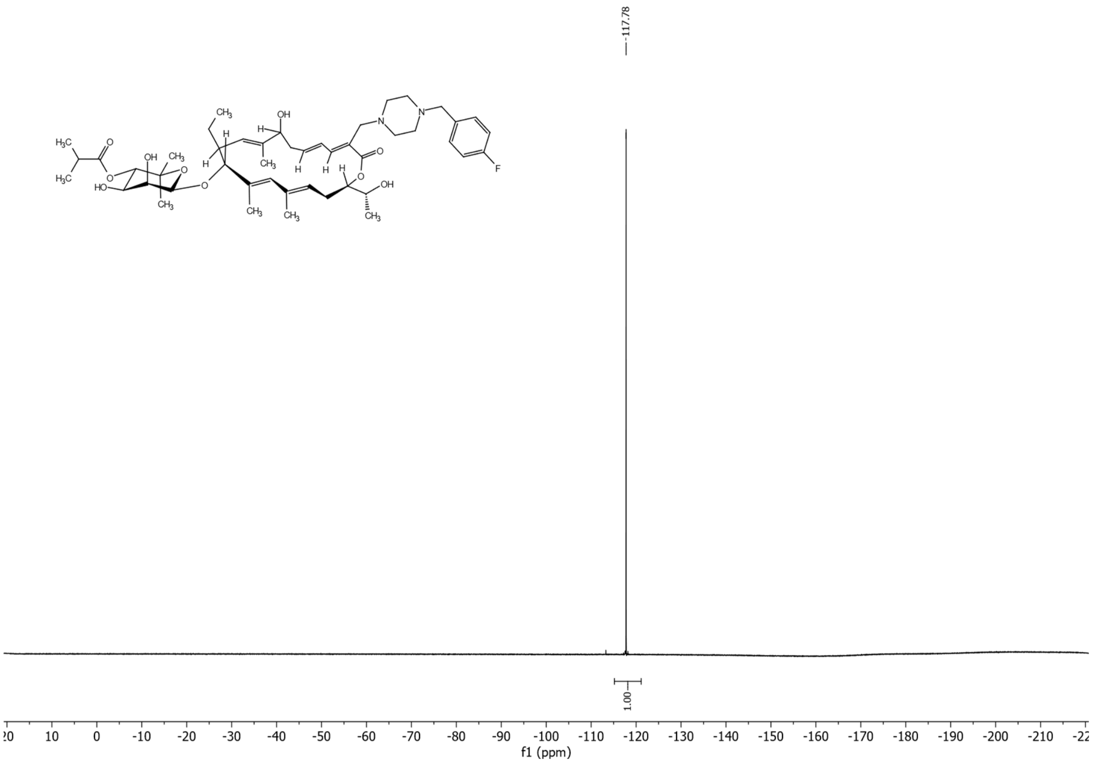

^{1}H NMR spectrum of **7** (500 MHz, acetone-d_6)

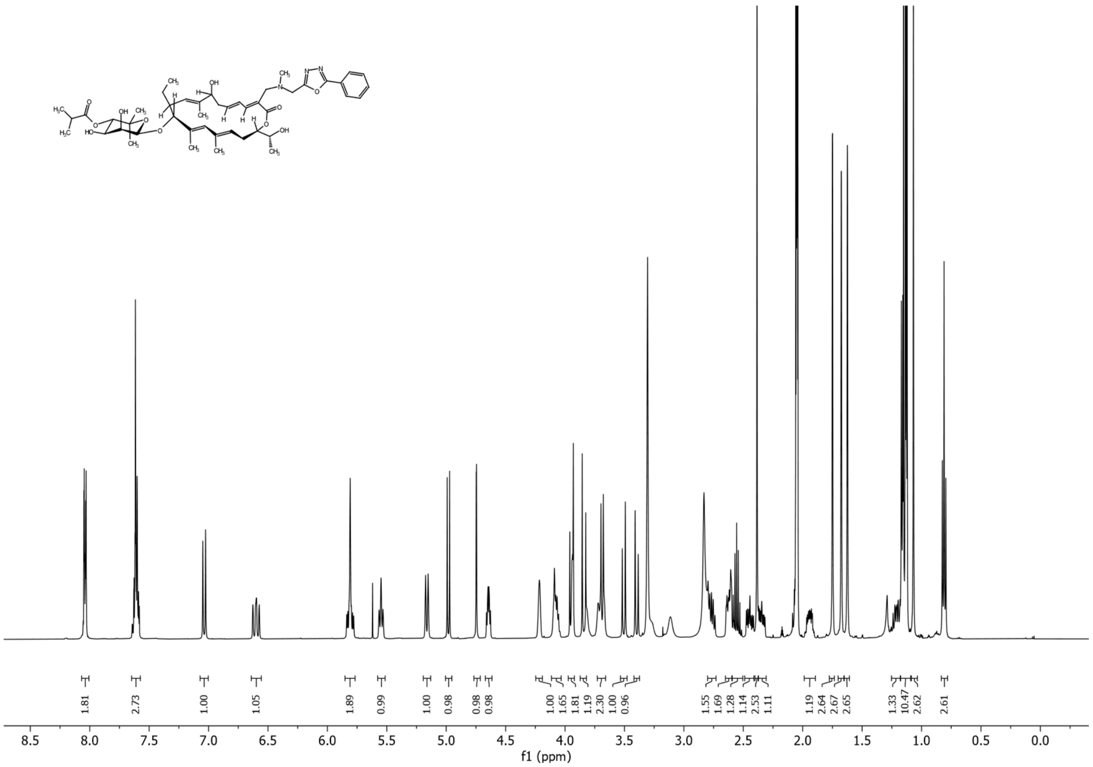

^{13}C NMR spectrum of **7** (126 MHz, acetone-d_6)

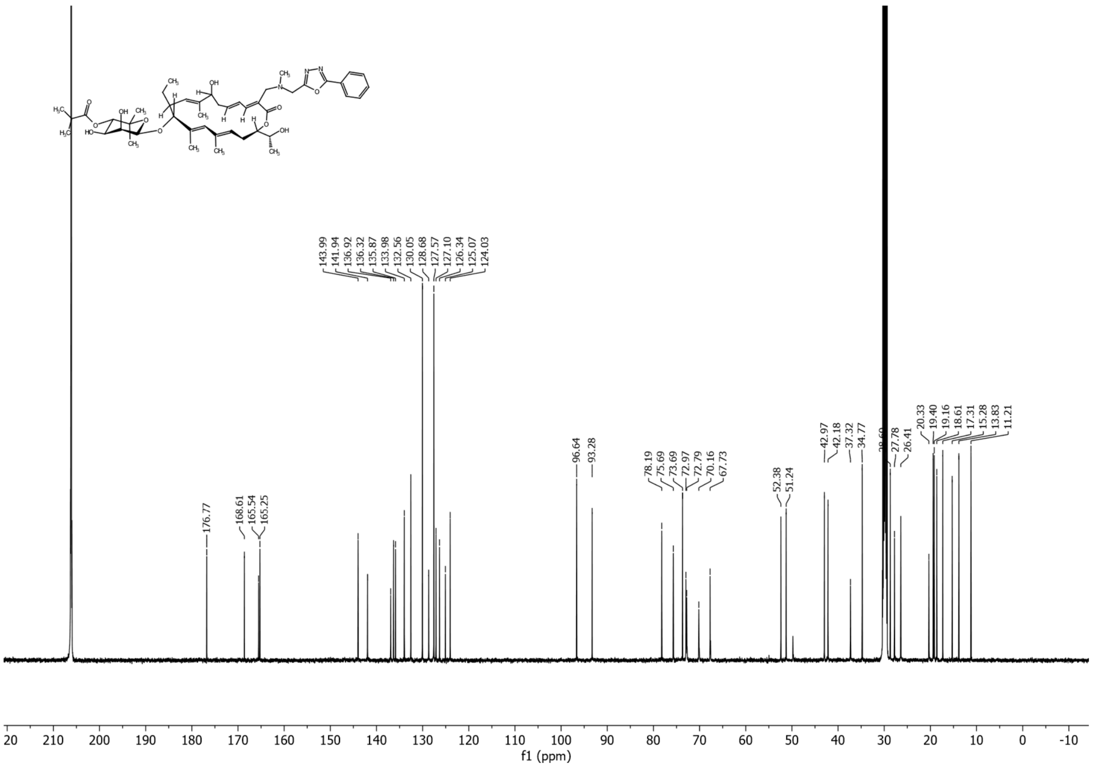

^{1}H NMR spectrum of **8** (500 MHz, acetone-d_6)

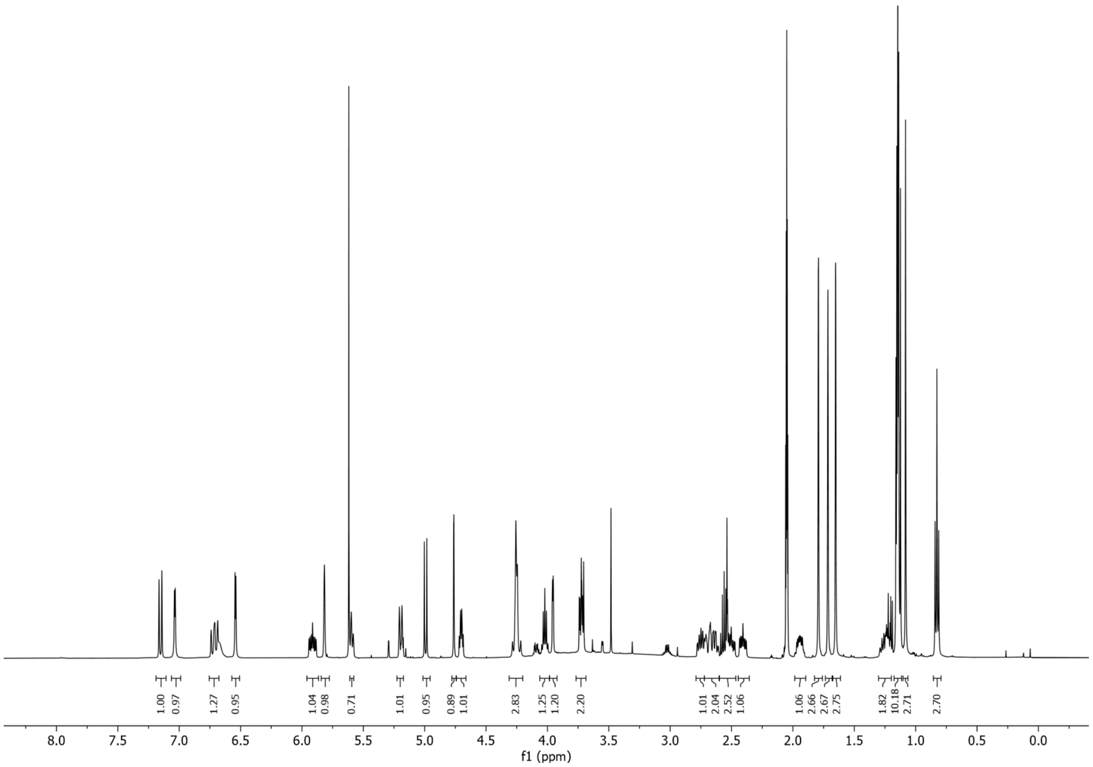

^{13}C NMR spectrum of **8** (126 MHz, acetone-d_6)

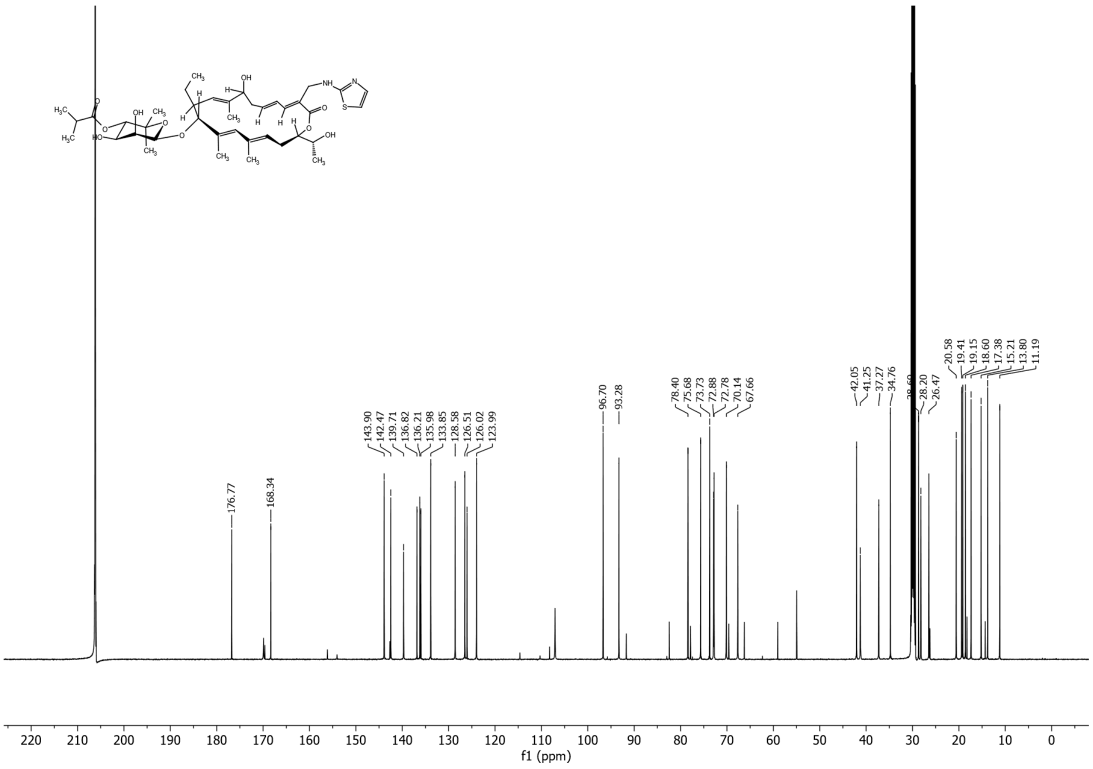

^{1}H NMR spectrum of **9** (500 MHz, acetonitrile-d_3)

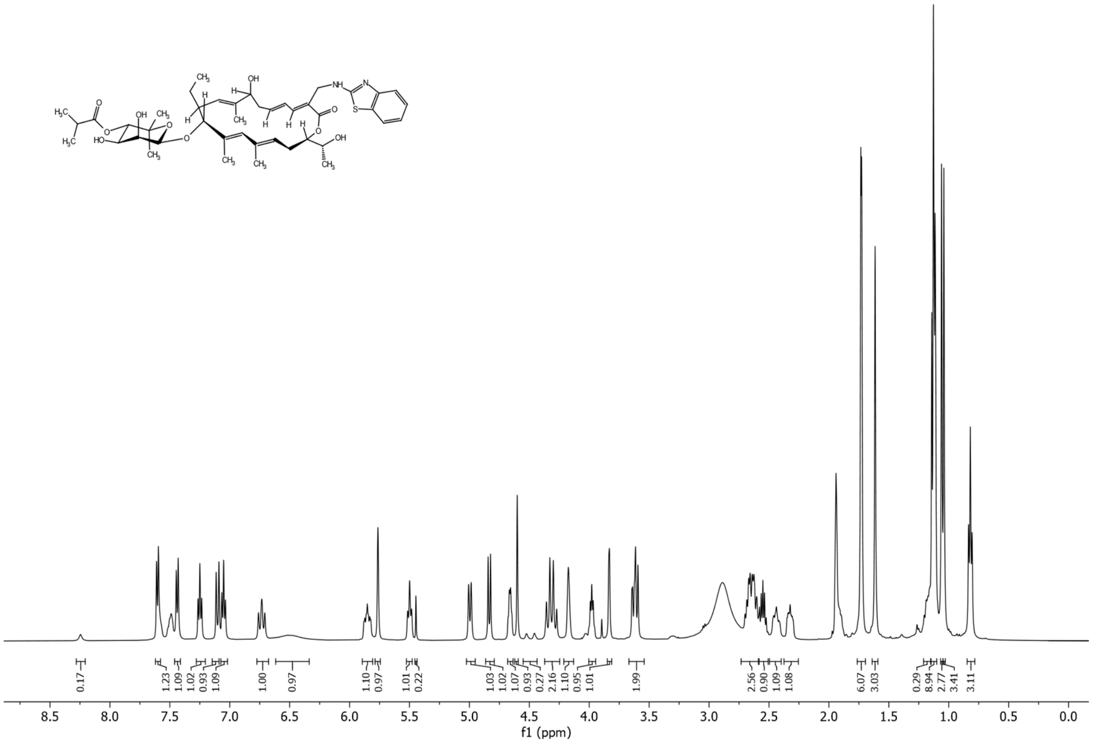

^{13}C NMR spectrum of **9** (126 MHz, acetonitrile-d_3)

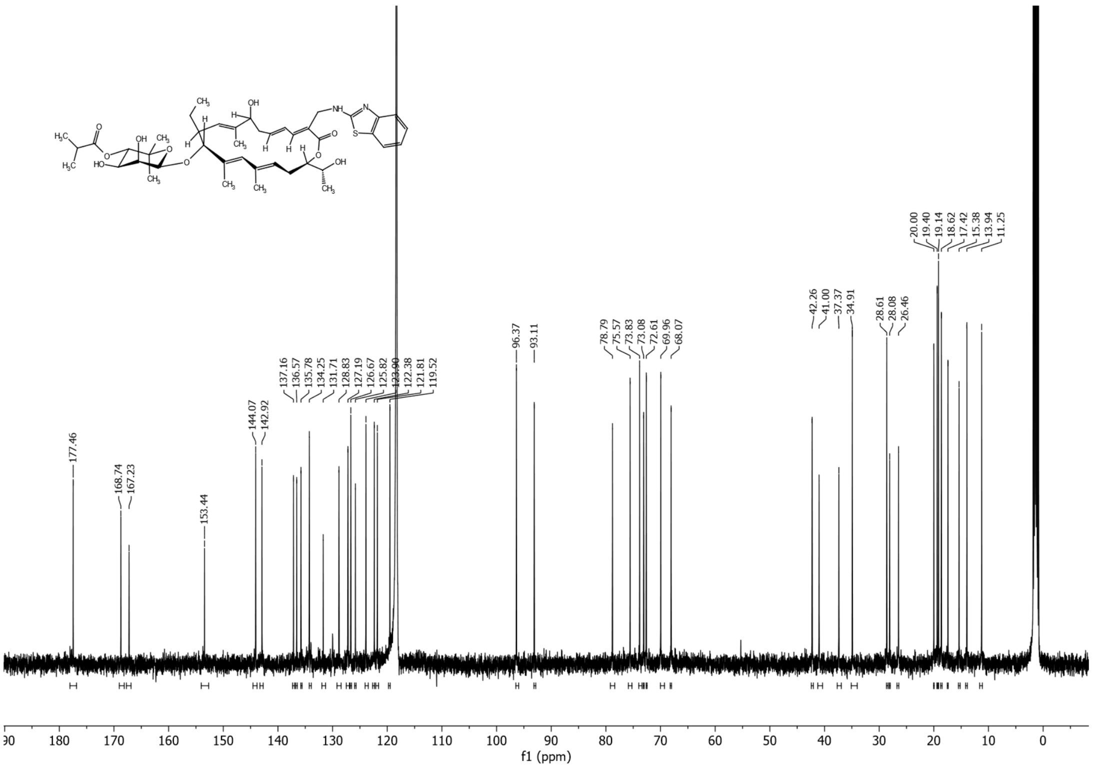

COSY spectrum of **9** (500 MHz, acetonitrile-d_3)

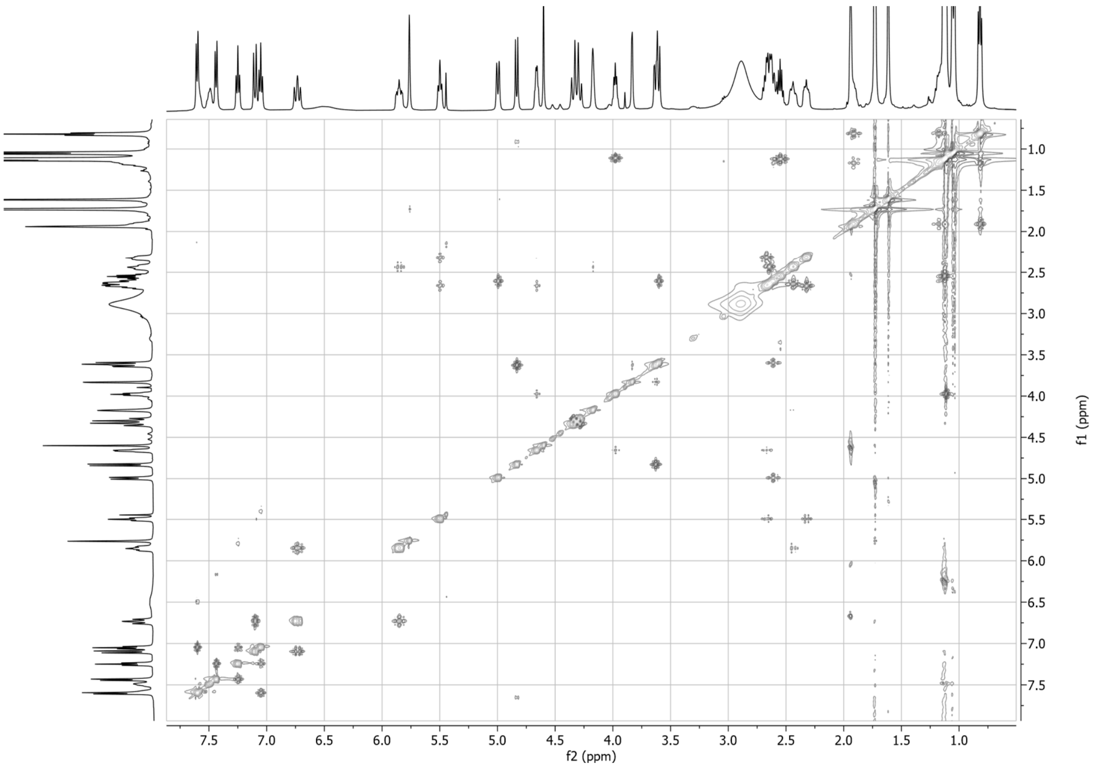

TOCSY spectrum of **9** (500 MHz, acetonitrile-d_3)

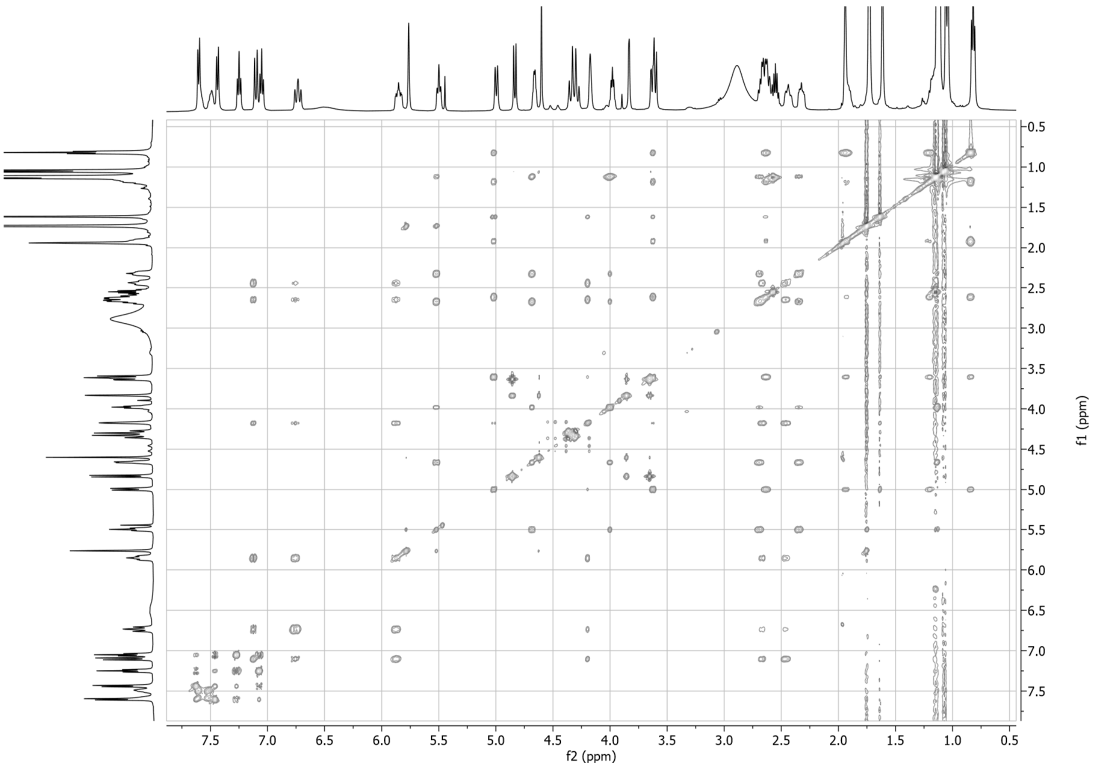

HSQC spectrum of **9** (500 MHz 126 MHz, acetonitrile-d_3)

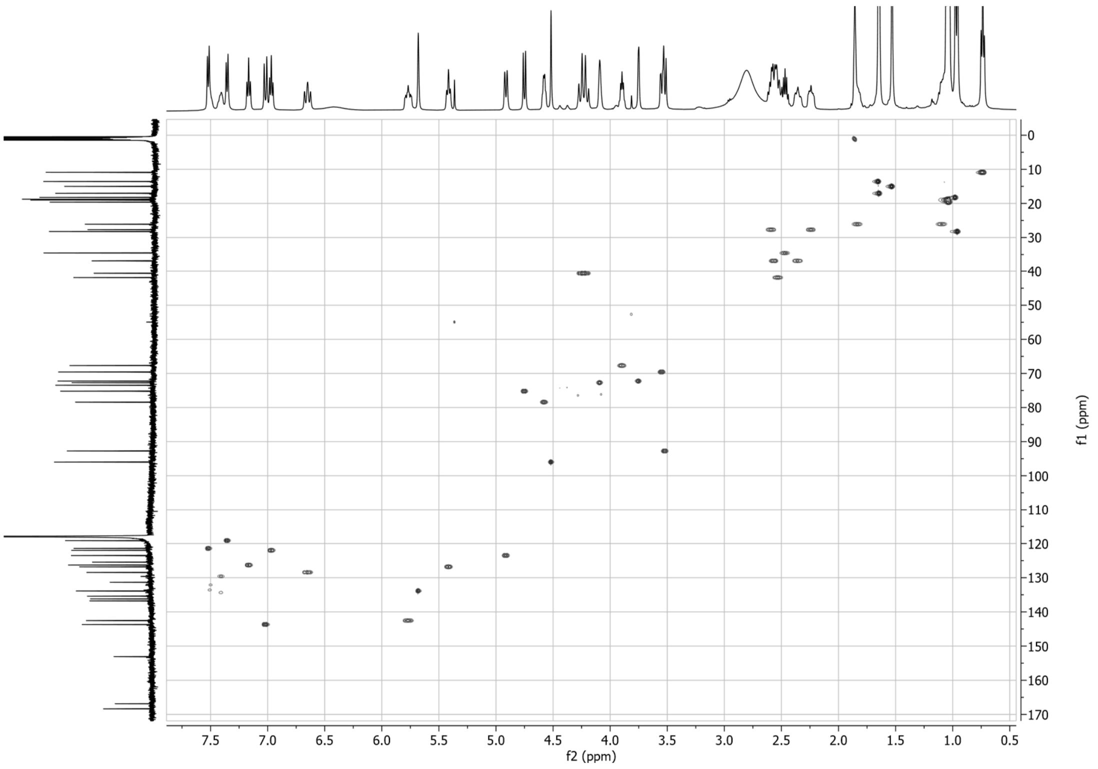

HMBC spectrum of **9** (500 MHz 126 MHz, acetonitrile-d_3)

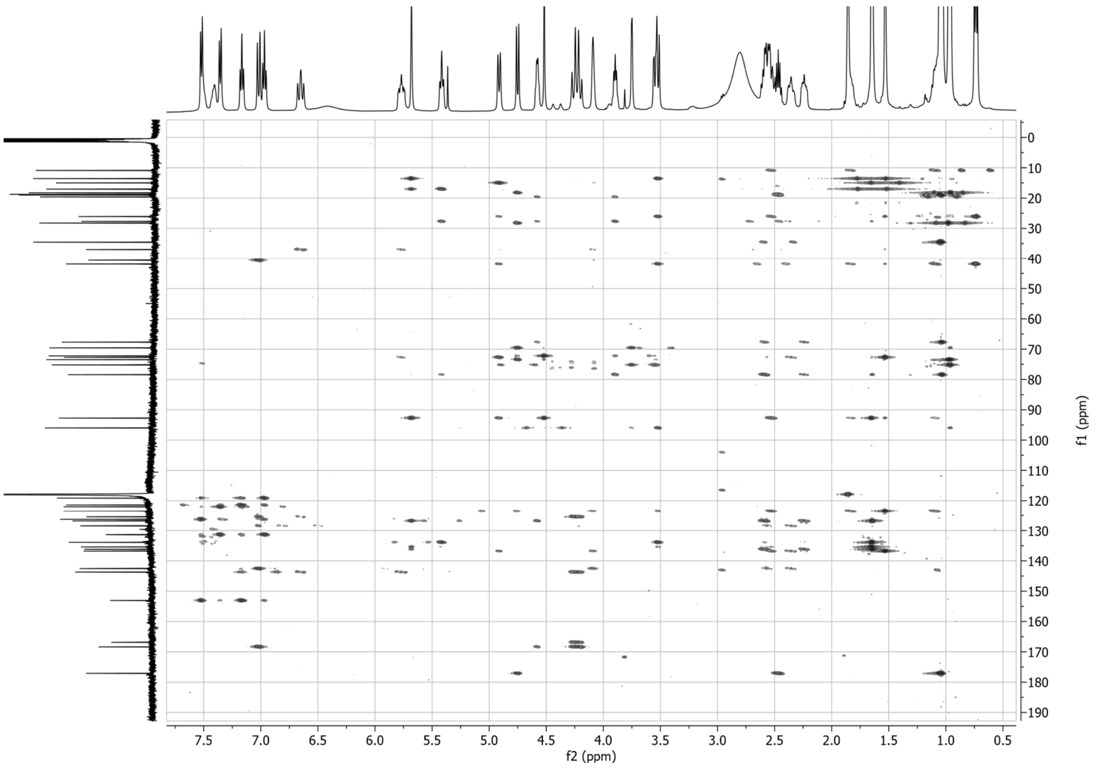

ROESY spectrum of **9** (500 MHz, acetonitrile-d_3)

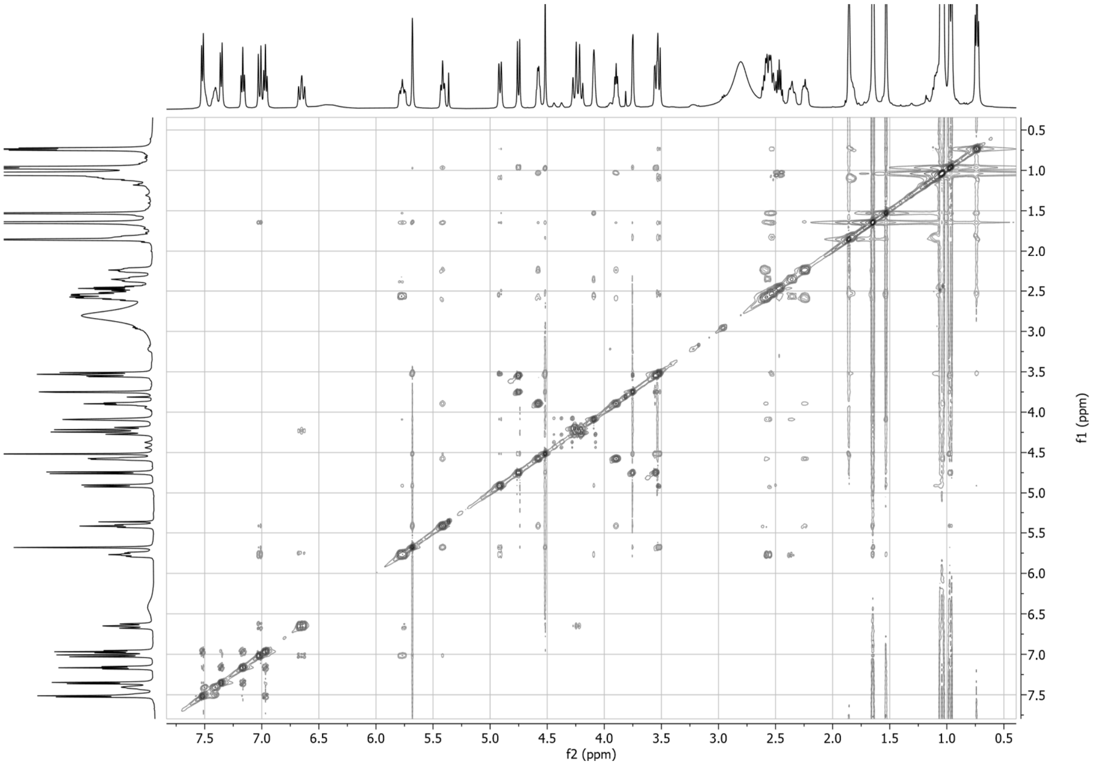

^{1}H NMR spectrum of **9i** (500 MHz, acetonitrile-d_3)

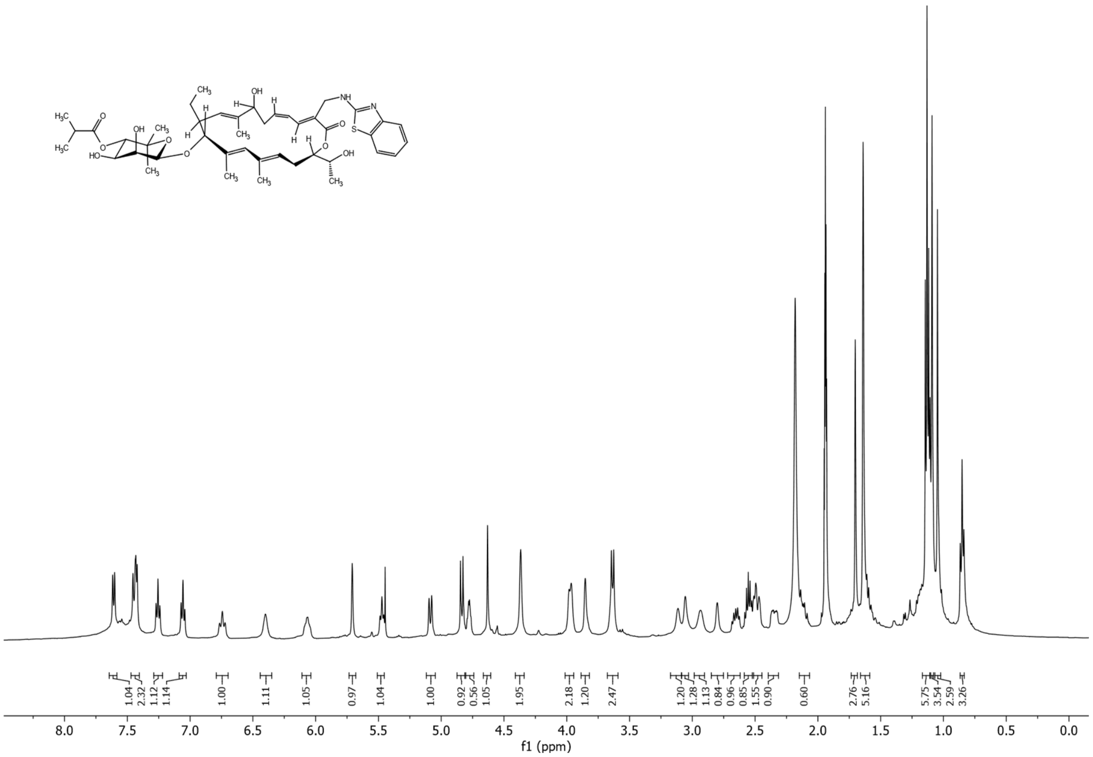

^{13}C NMR spectrum of **9i** (126 MHz, acetonitrile-d_3)

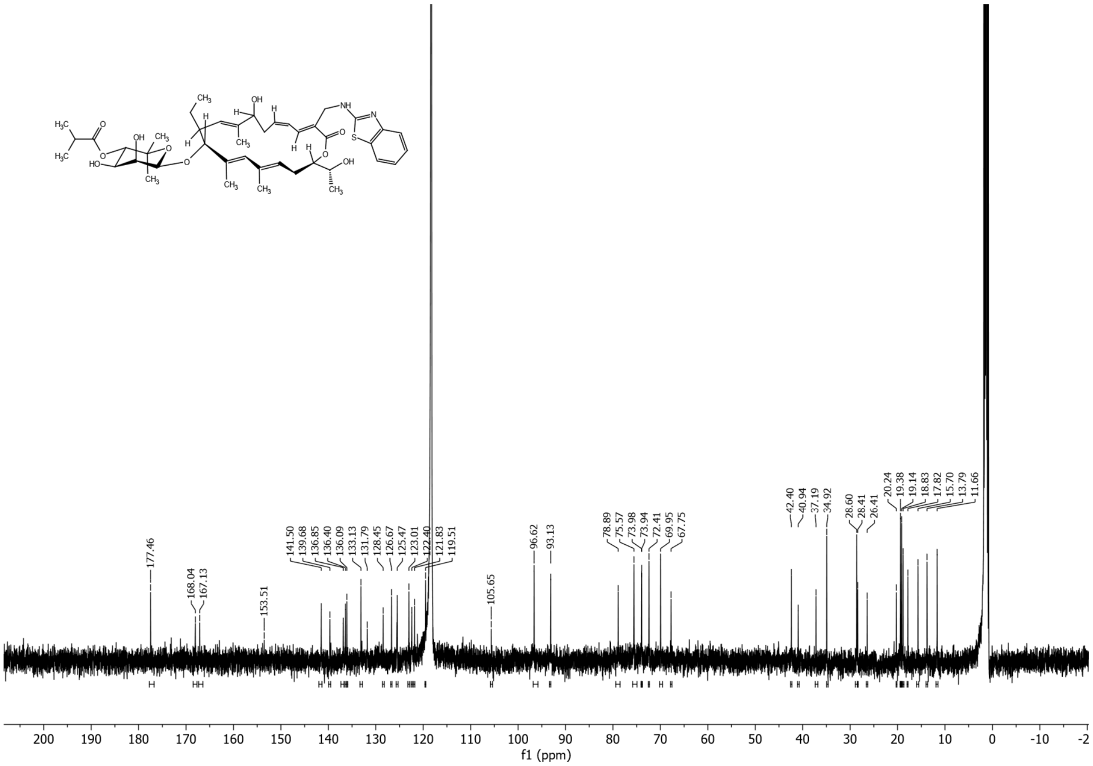

COSY spectrum of **9i** (500 MHz, acetonitrile-d_3)

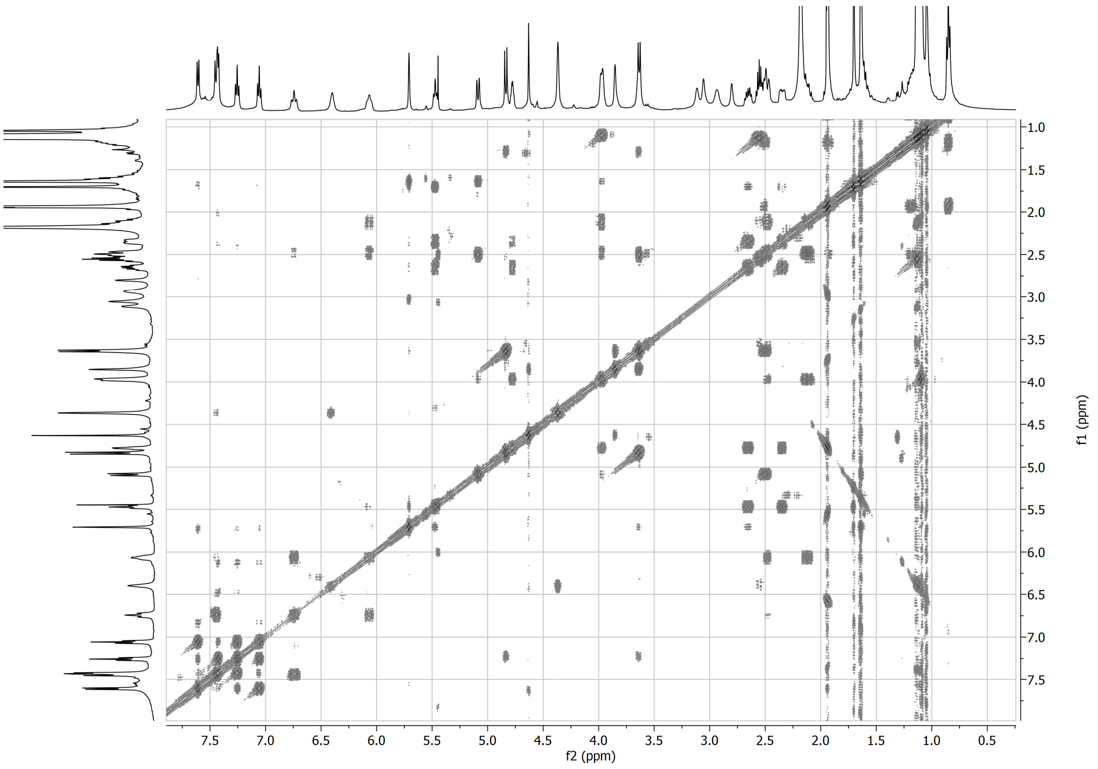

TOCSY spectrum of **9i** (500 MHz, acetonitrile-d_3)

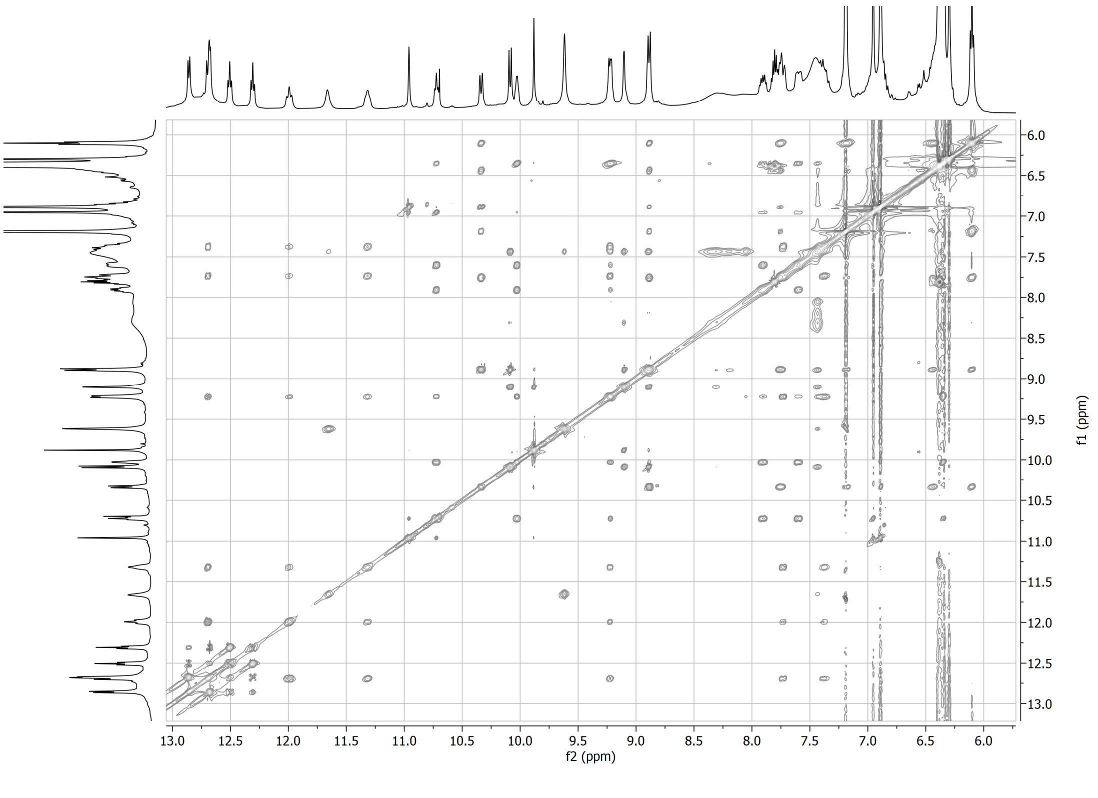

HSQC spectrum of **9i** (500 MHz 126 MHz, acetonitrile-d_3)

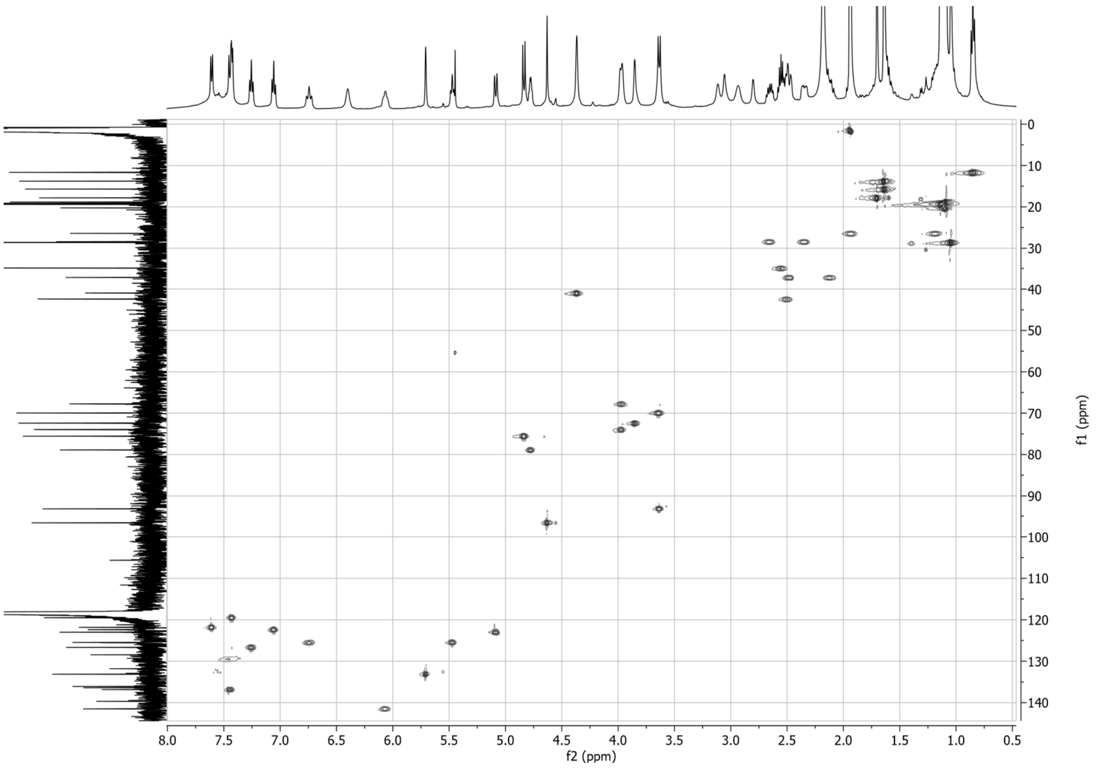

HMBC spectrum of **9i** (500 MHz 126 MHz, acetonitrile-d_3)

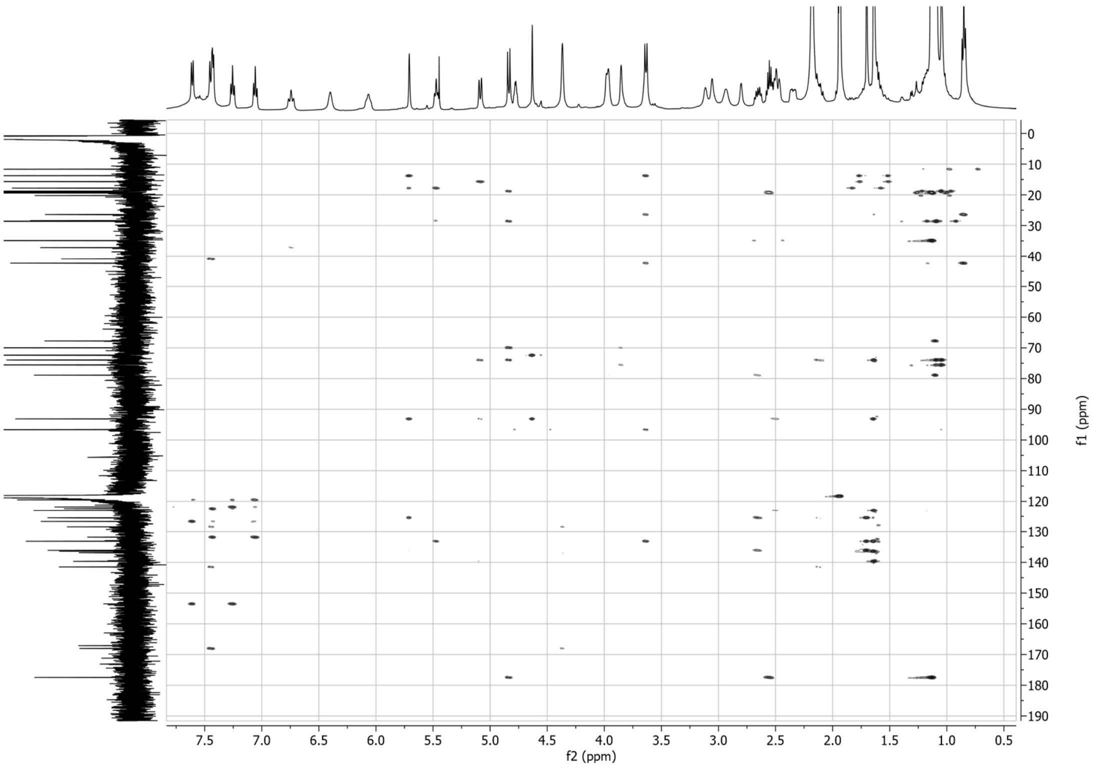

NOESY spectrum of **9i** (500 MHz, acetonitrile-d_3)

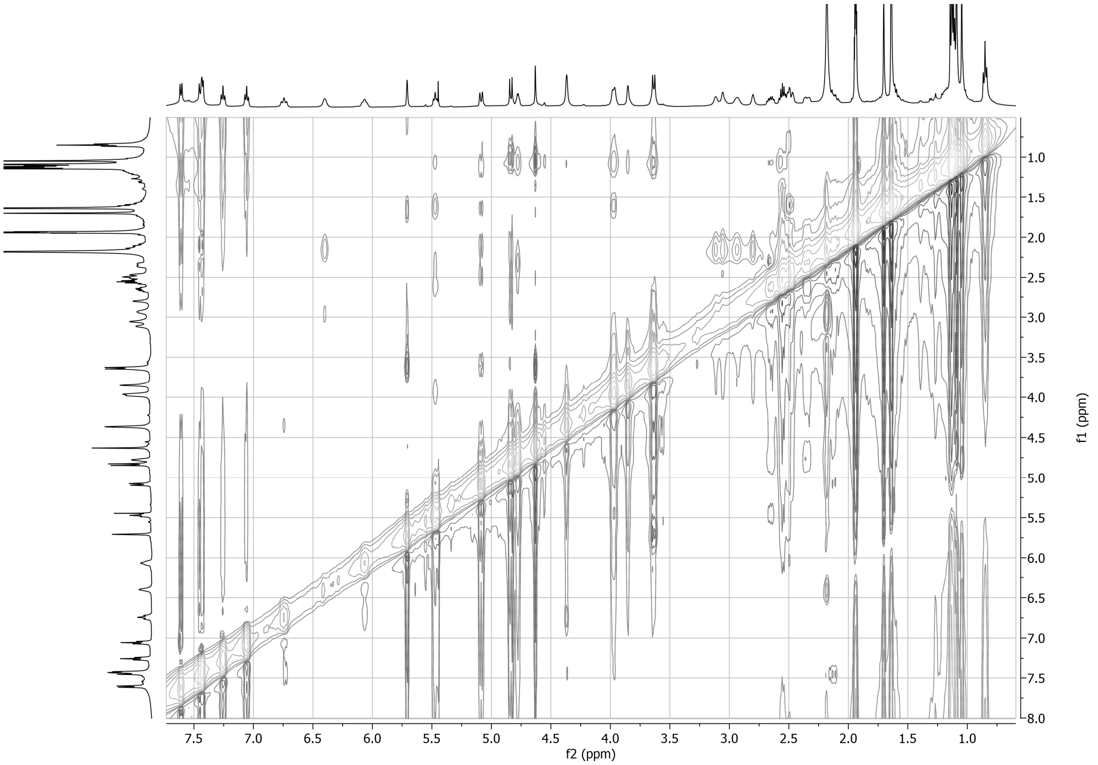

^{1}H NMR spectrum of **10** (500 MHz, acetone-d_6)

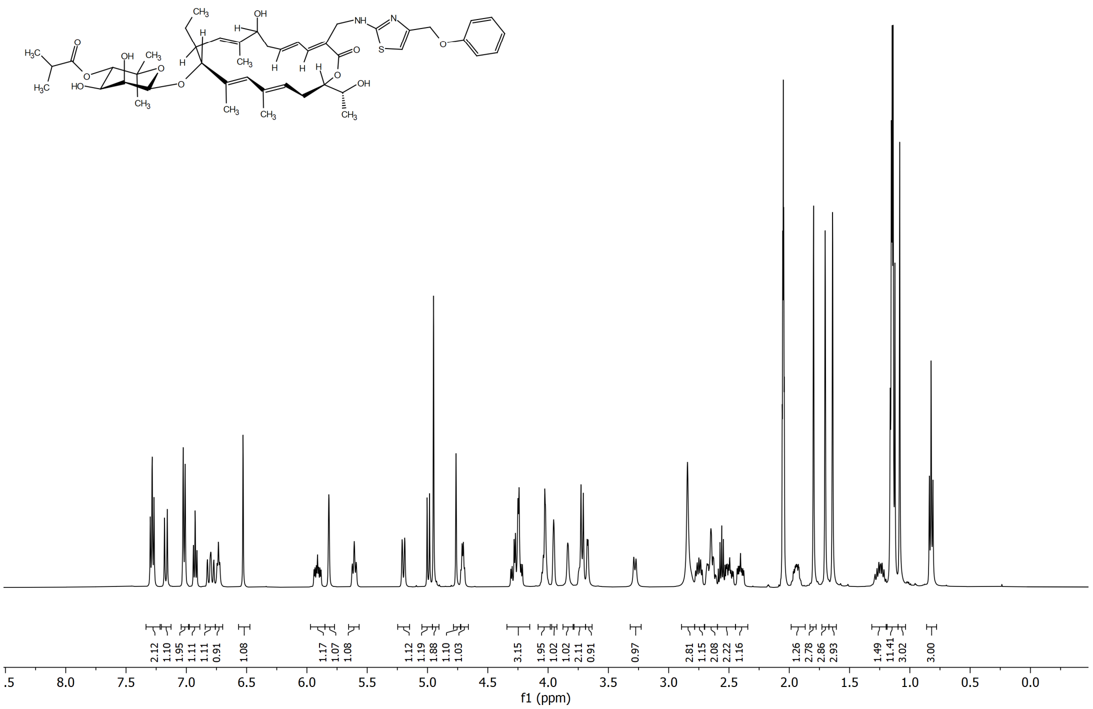

^{13}C NMR spectrum of **10** (126 MHz, acetone-d_6)

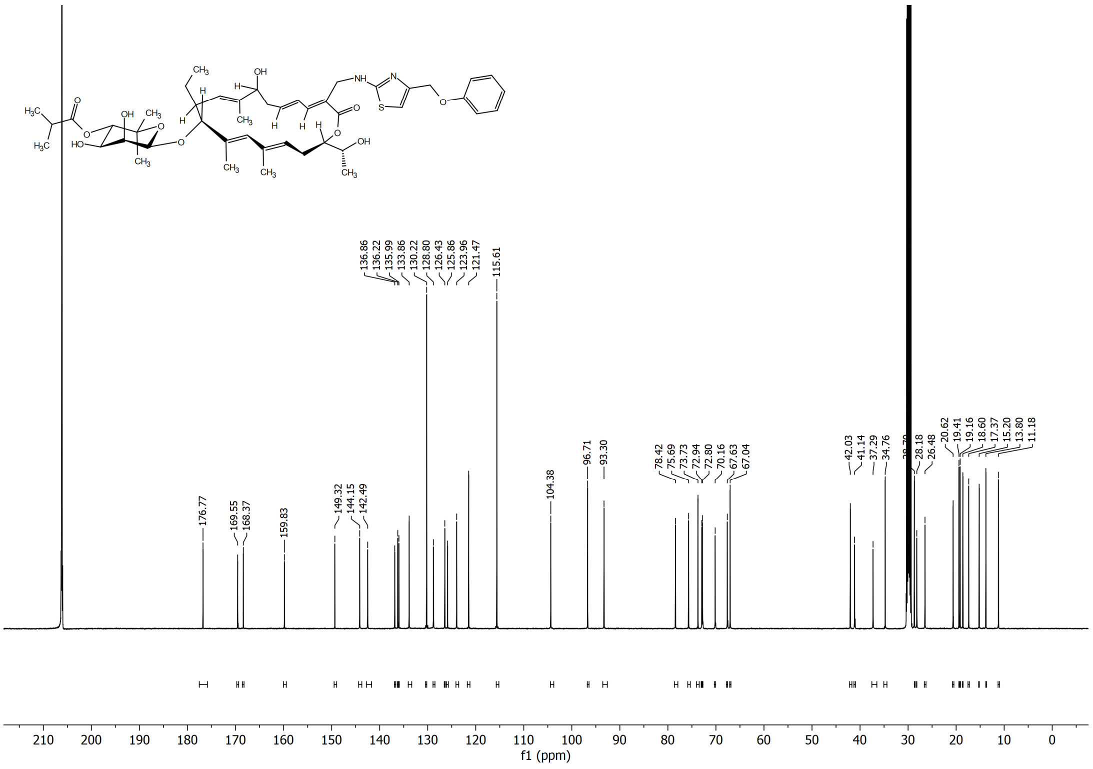

^{1}H NMR spectrum of **11** (500 MHz, acetone-d_6)

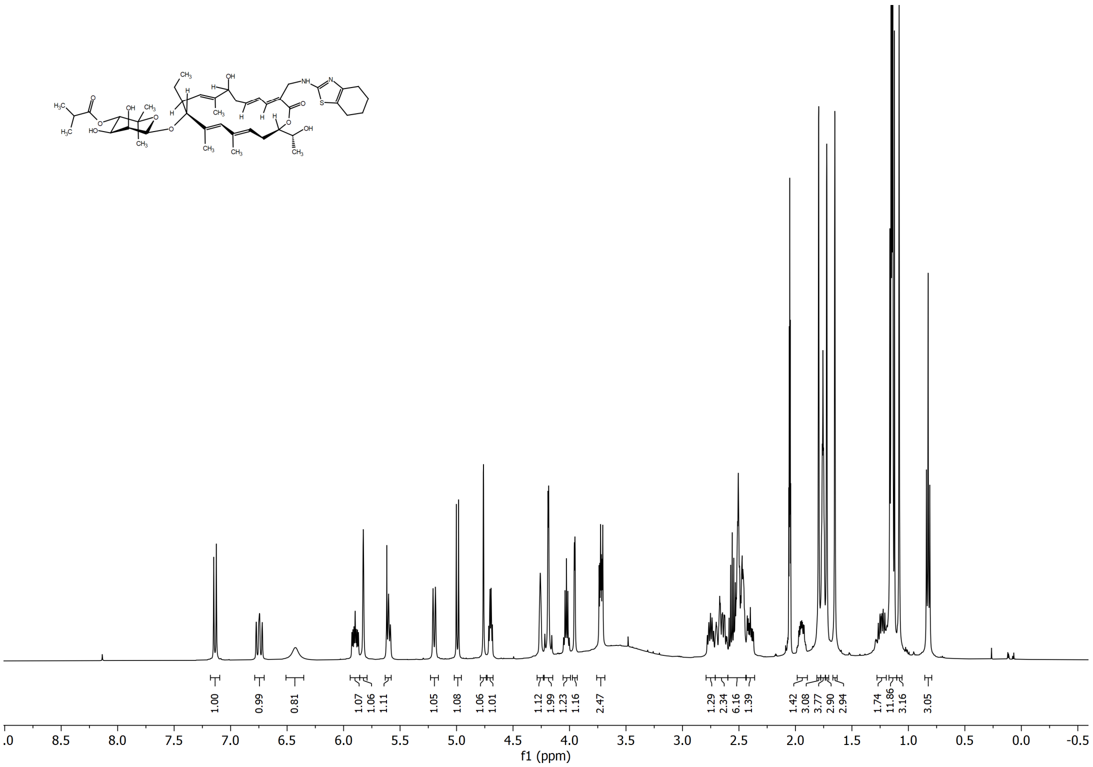

^{13}C NMR spectrum of **11** (126 MHz, acetone-d_6)

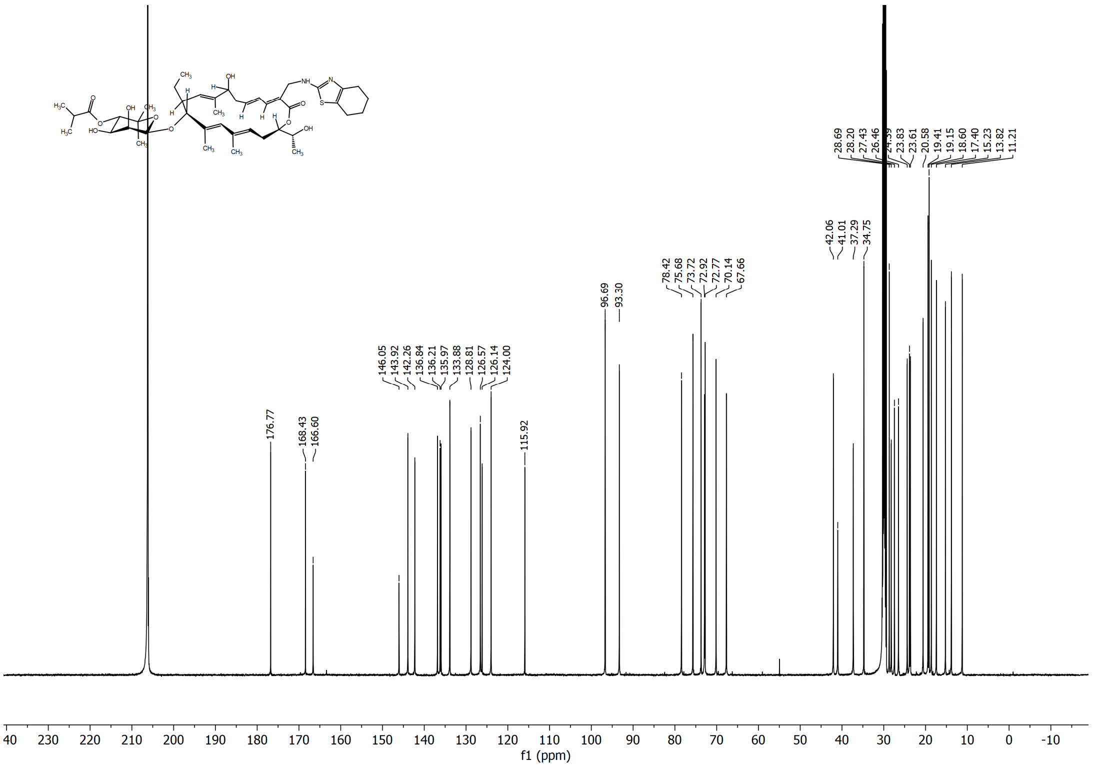

^{1}H NMR spectrum of **12** (500 MHz, acetone-d_6)

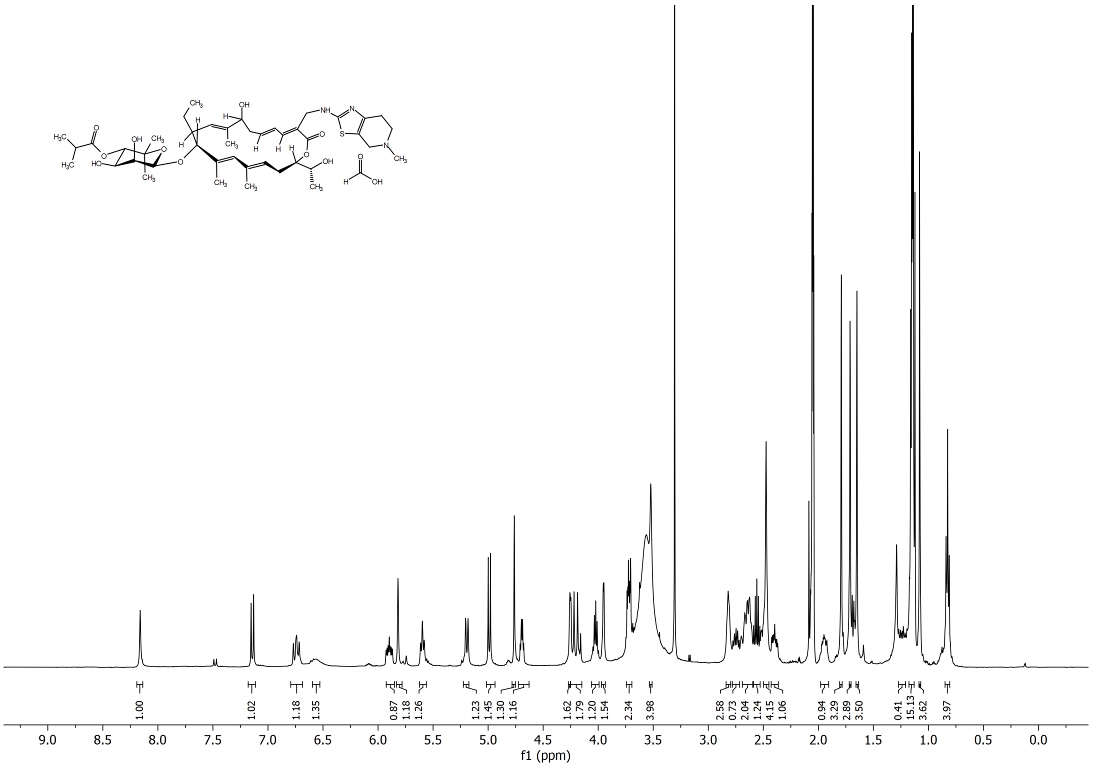

^{13}C NMR spectrum of **12** (126 MHz, acetone-d_6)

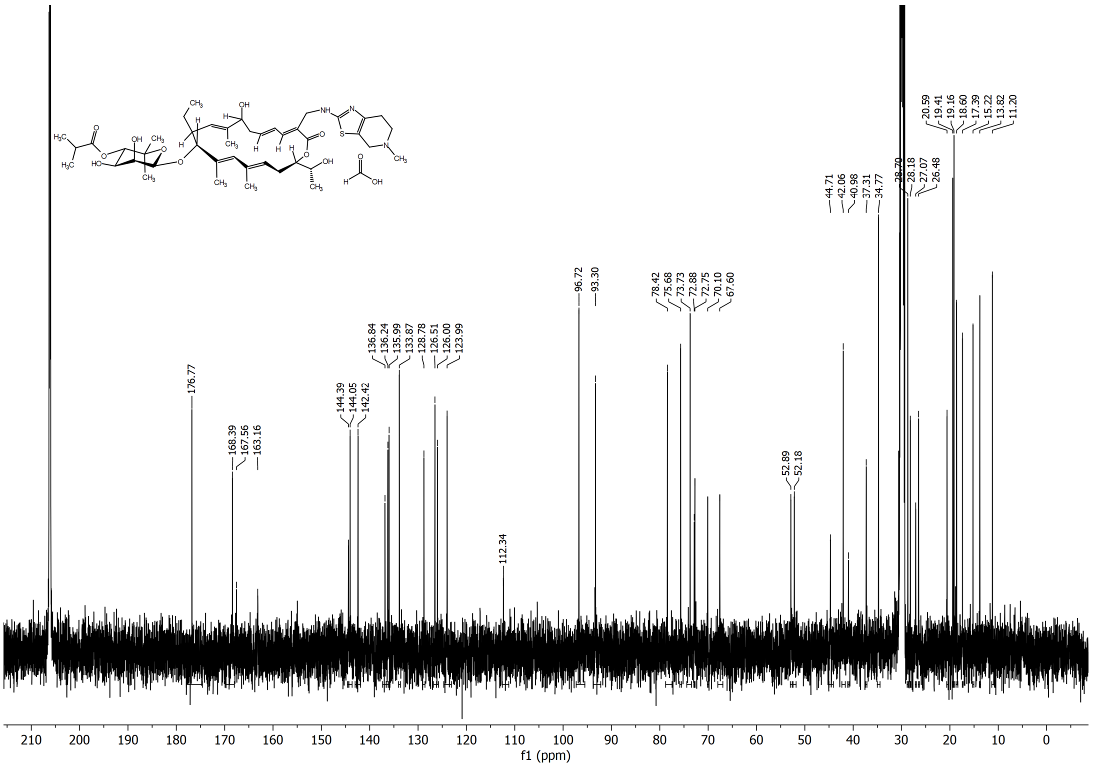

COSY spectrum of **12** (500 MHz, acetone-d_6)

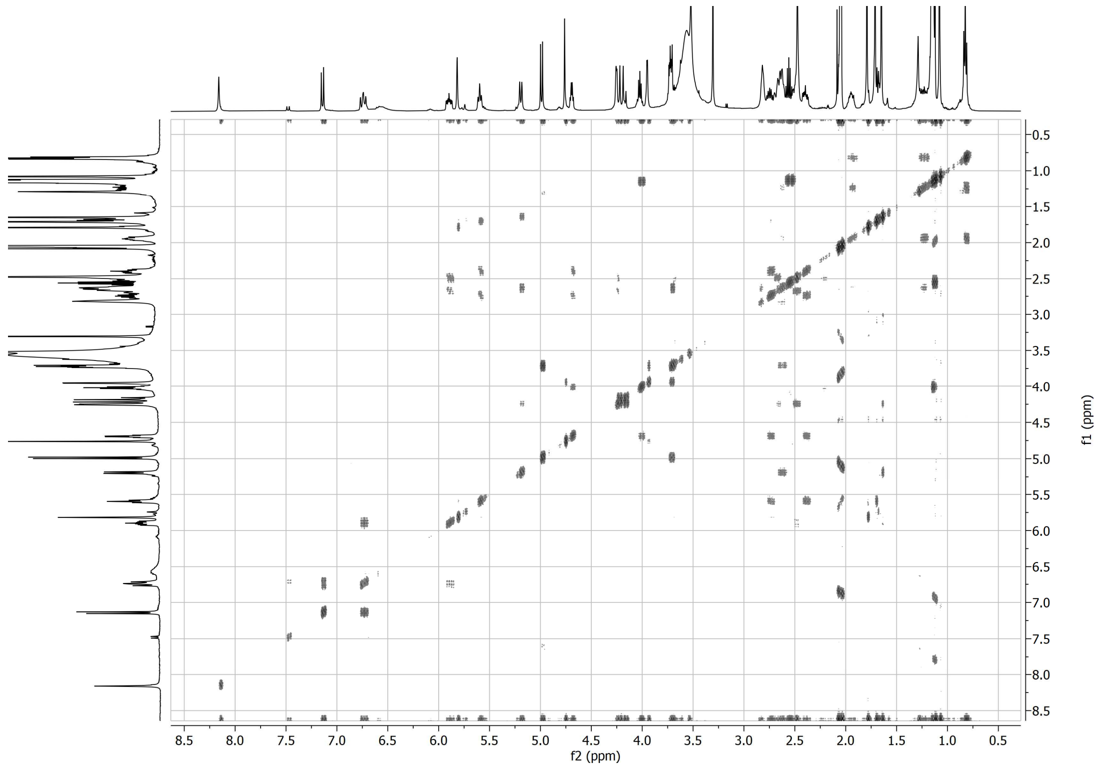

TOCSY spectrum of **12** (500 MHz, acetone-d_6)

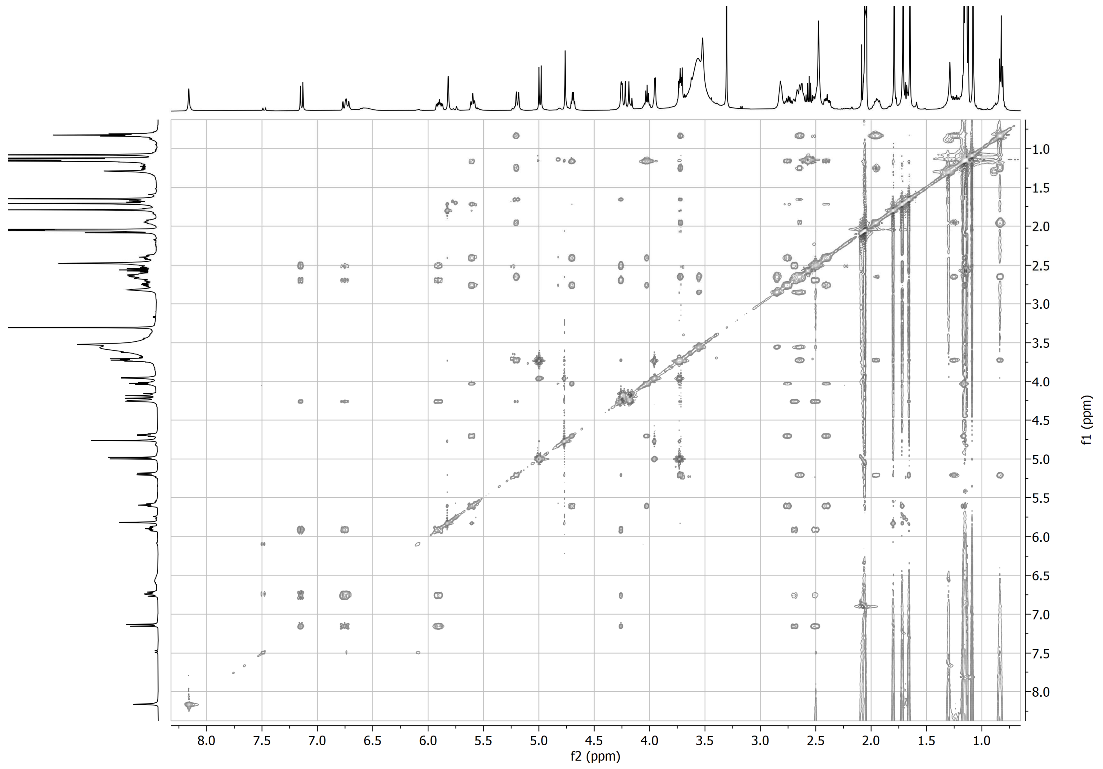

HSQC spectrum of **12** (500 MHz 126 MHz, acetone-d_6)

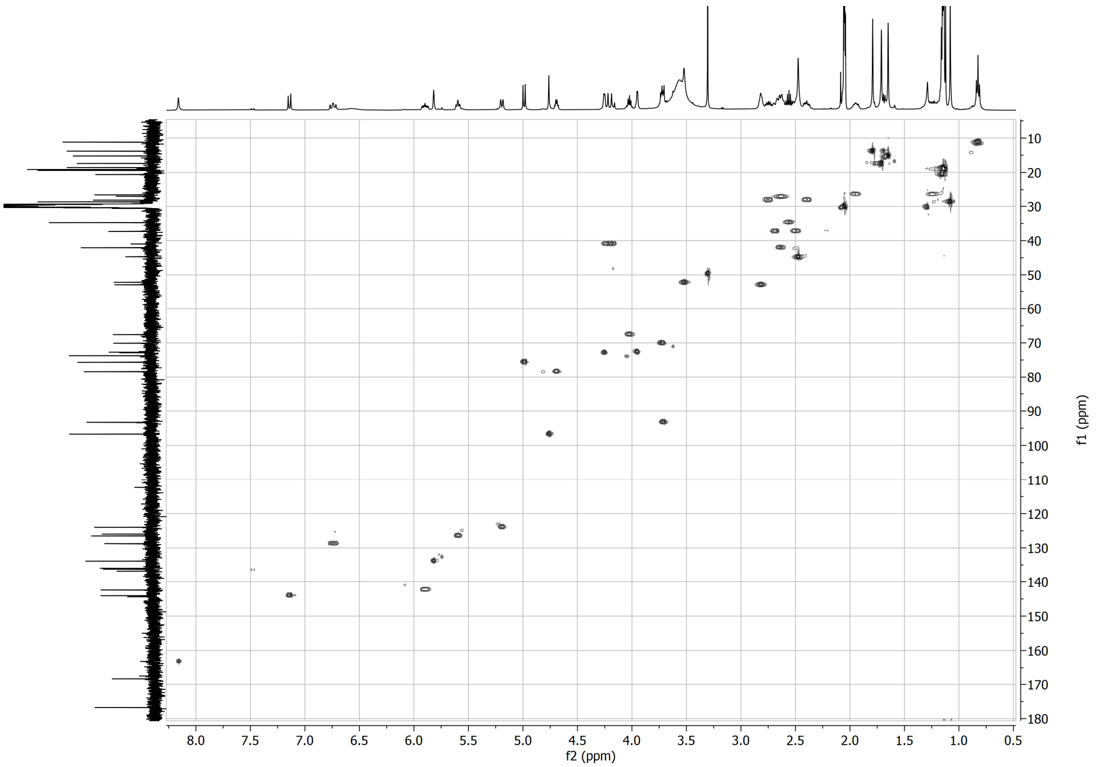

HMBC spectrum of **12** (500 MHz 126 MHz, acetone-d_6)

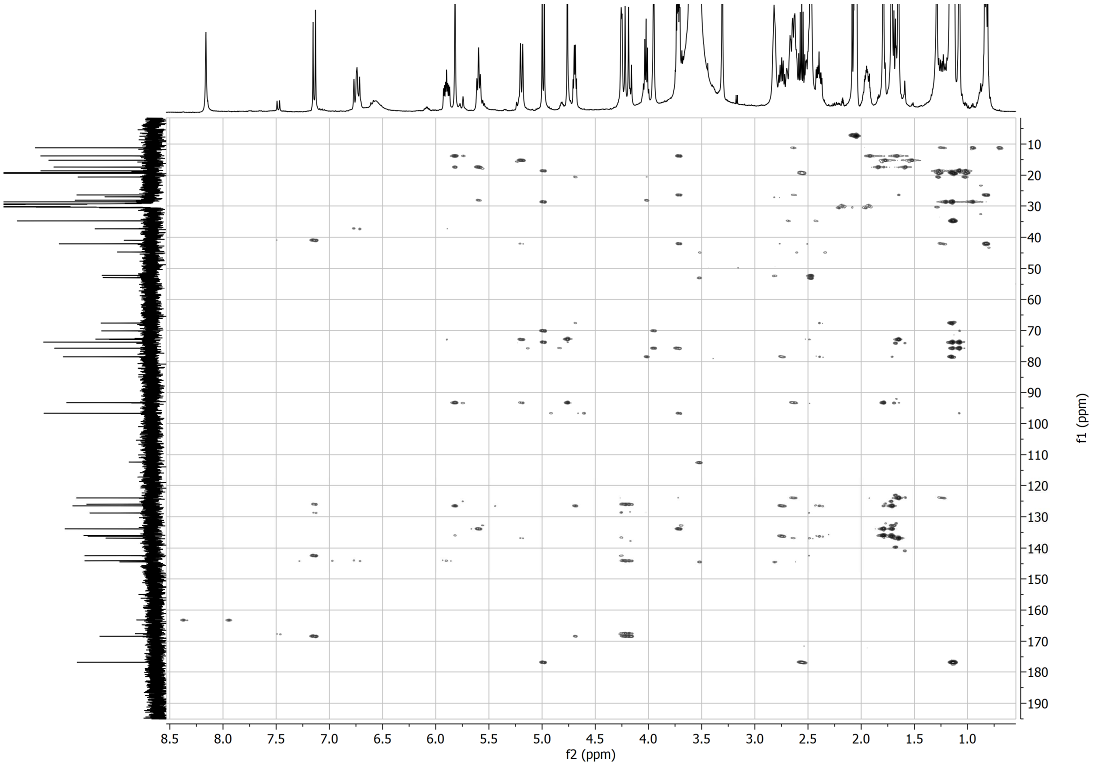

NOESY spectrum of **12** (500 MHz, acetone-d_6)

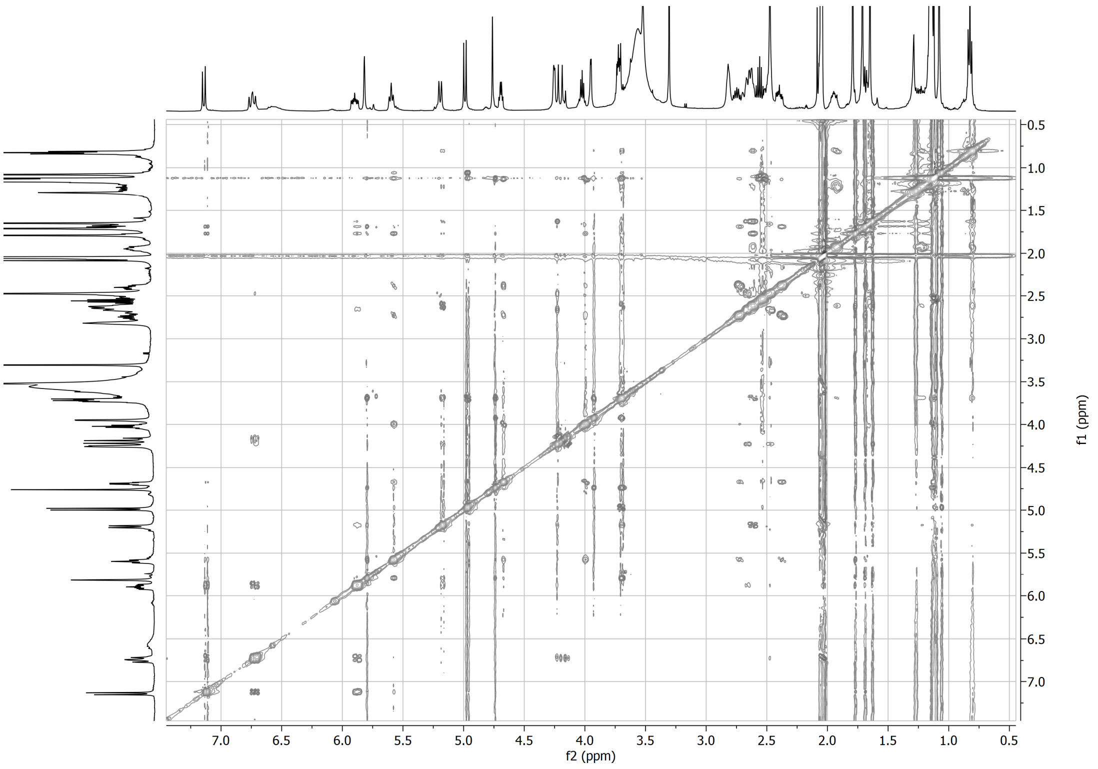

^{1}H NMR spectrum of **12a** (400 MHz, DMSO-d_6)

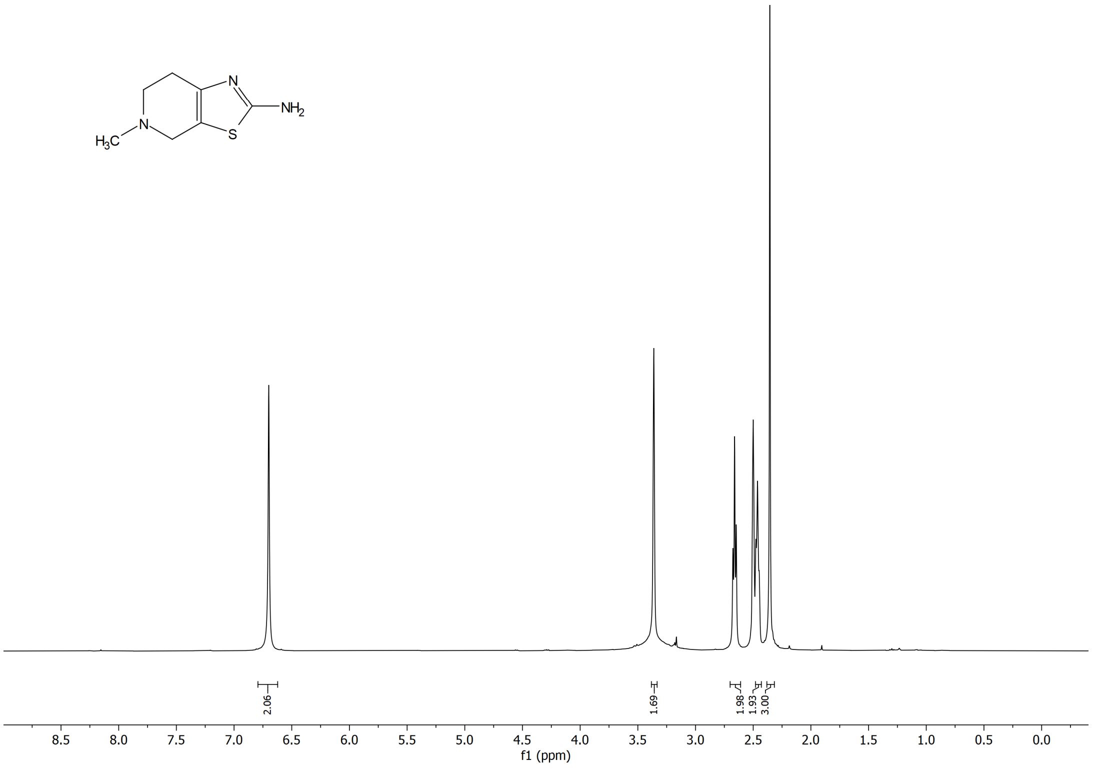

^{1}H NMR spectrum of **13** (500 MHz, acetone-d_6)

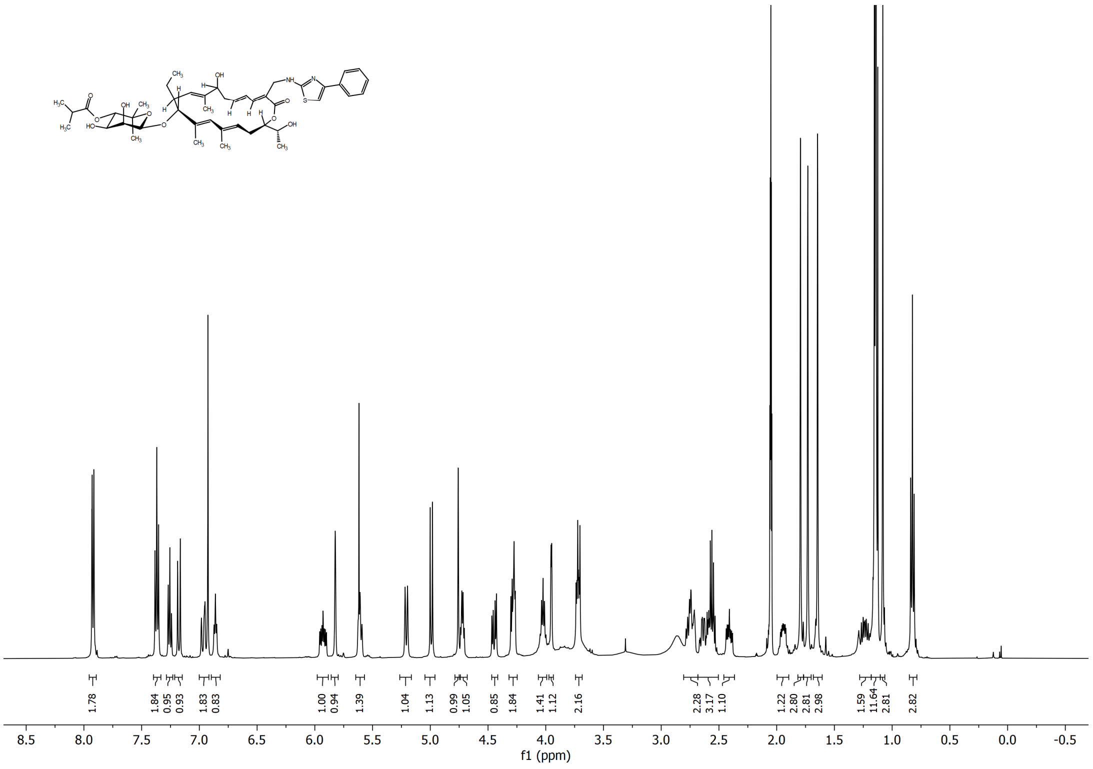

^{13}C NMR spectrum of **13** (126 MHz, acetone-d_6)

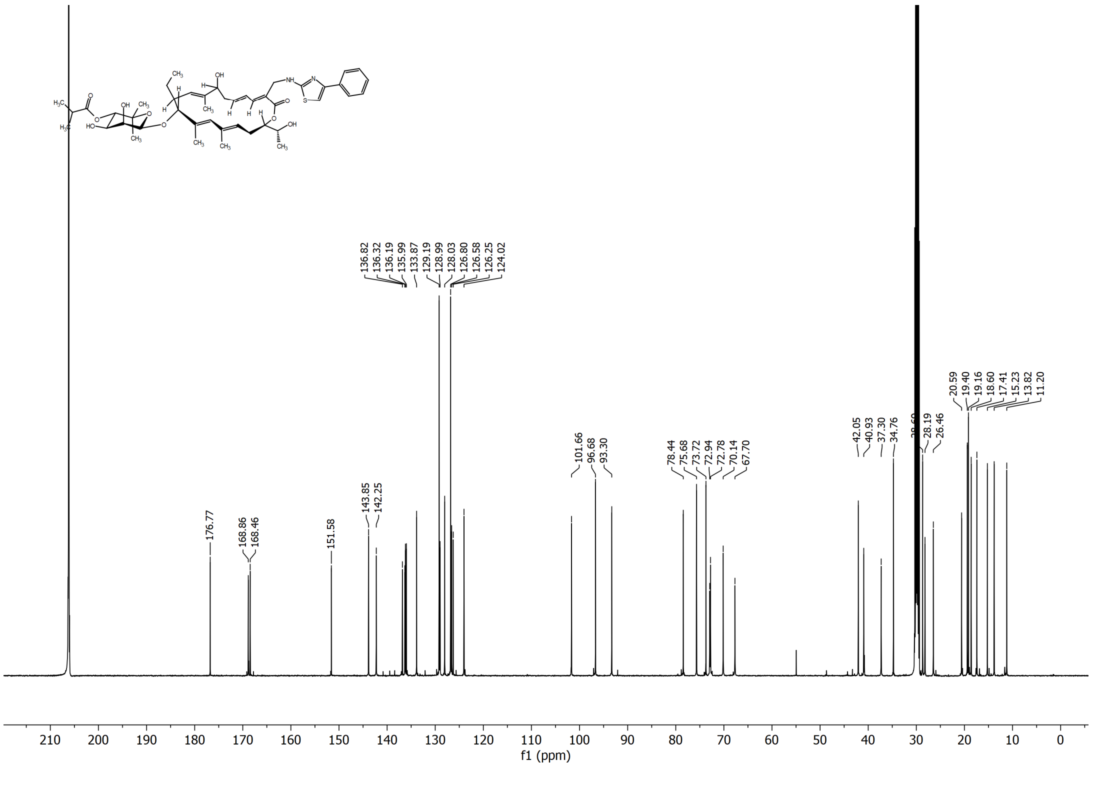

^{1}H NMR spectrum of **14** (500 MHz, $CDCl_3$)

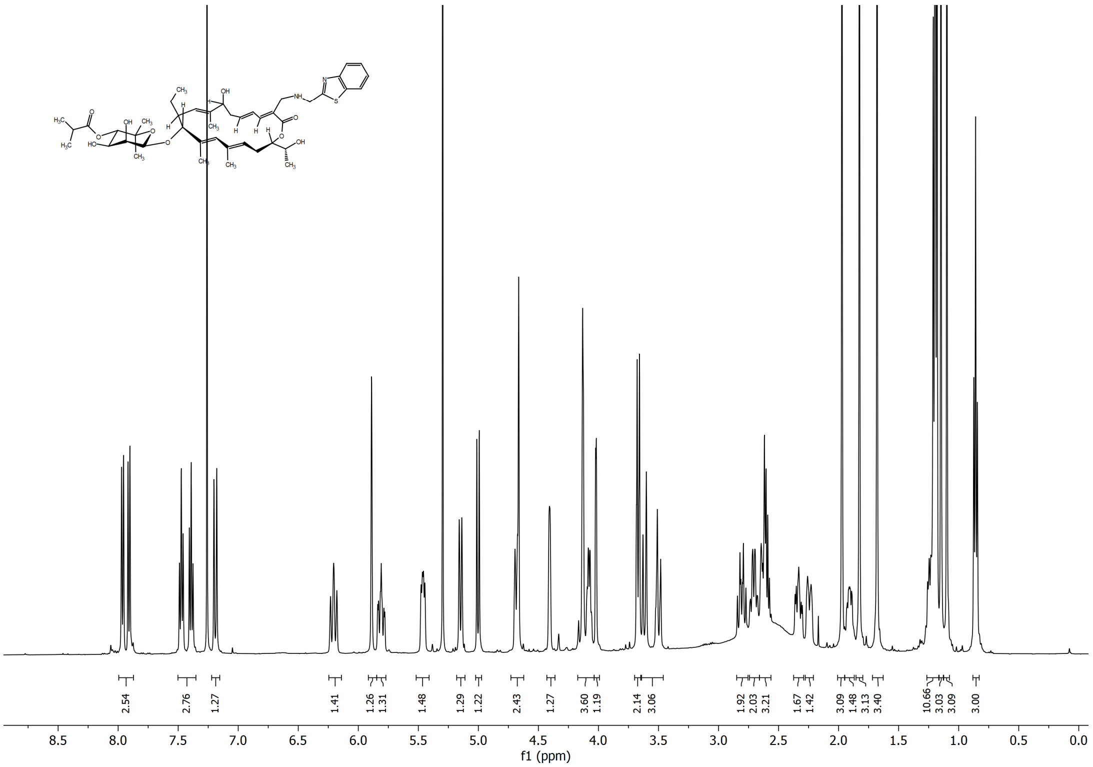

^{13}C NMR spectrum of **14** (126 MHz, $CDCl_3$)

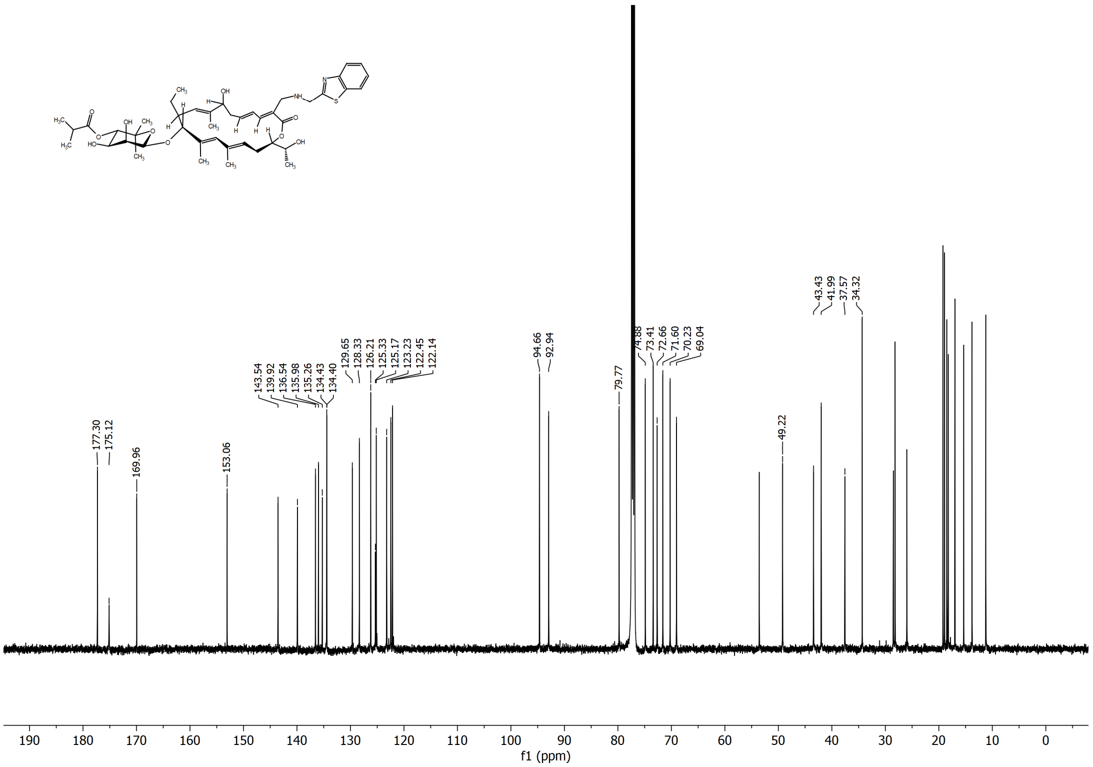

^{1}H NMR spectrum of **15** (500 MHz, $CDCl_3$)

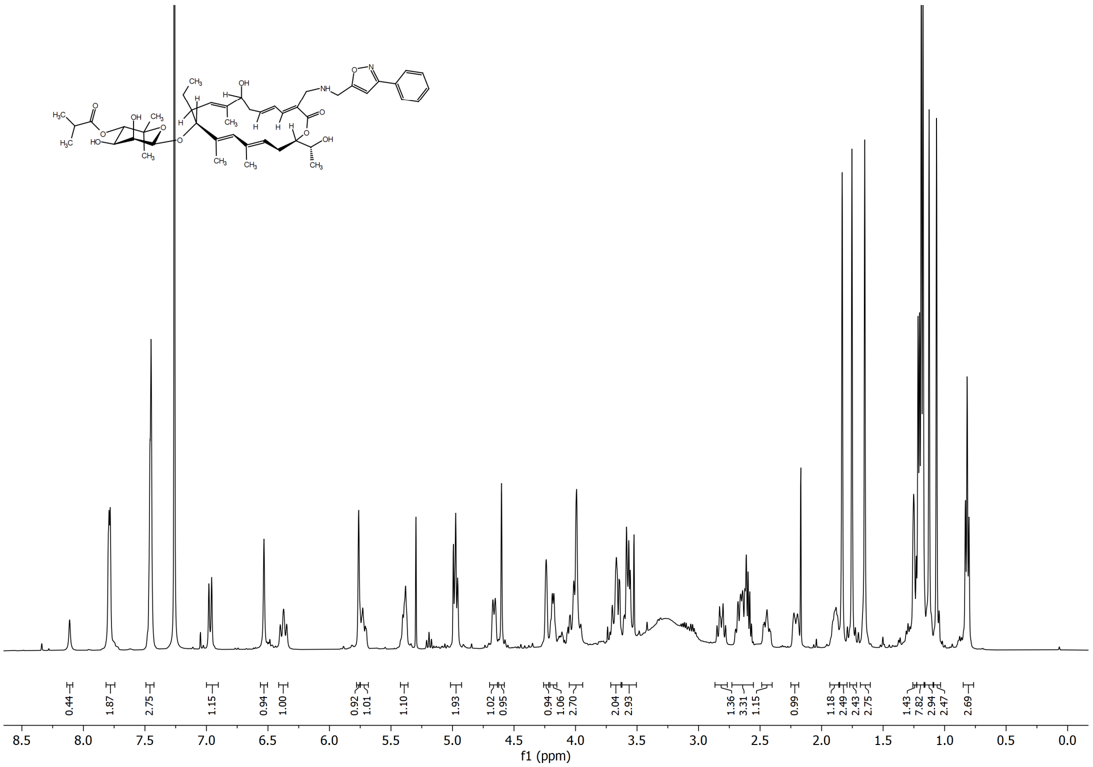

^{13}C NMR spectrum of **15** (126 MHz, $CDCl_3$)

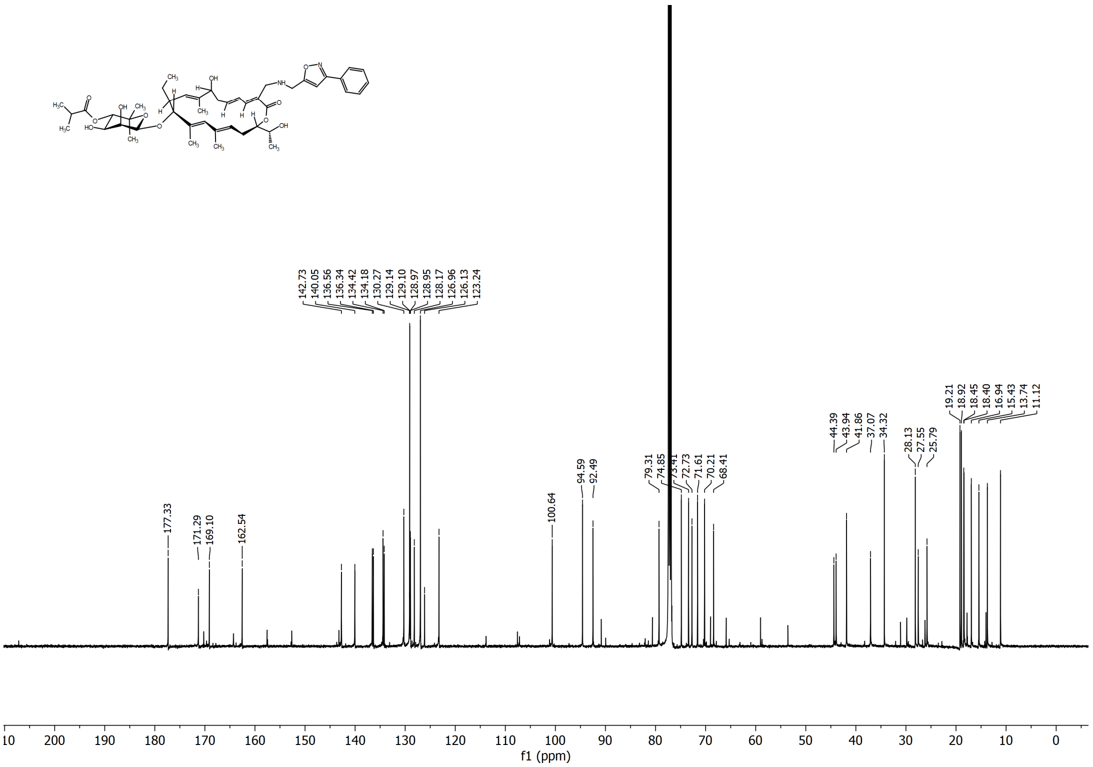

^{1}H NMR spectrum of **16** (500 MHz, acetone-d_6)

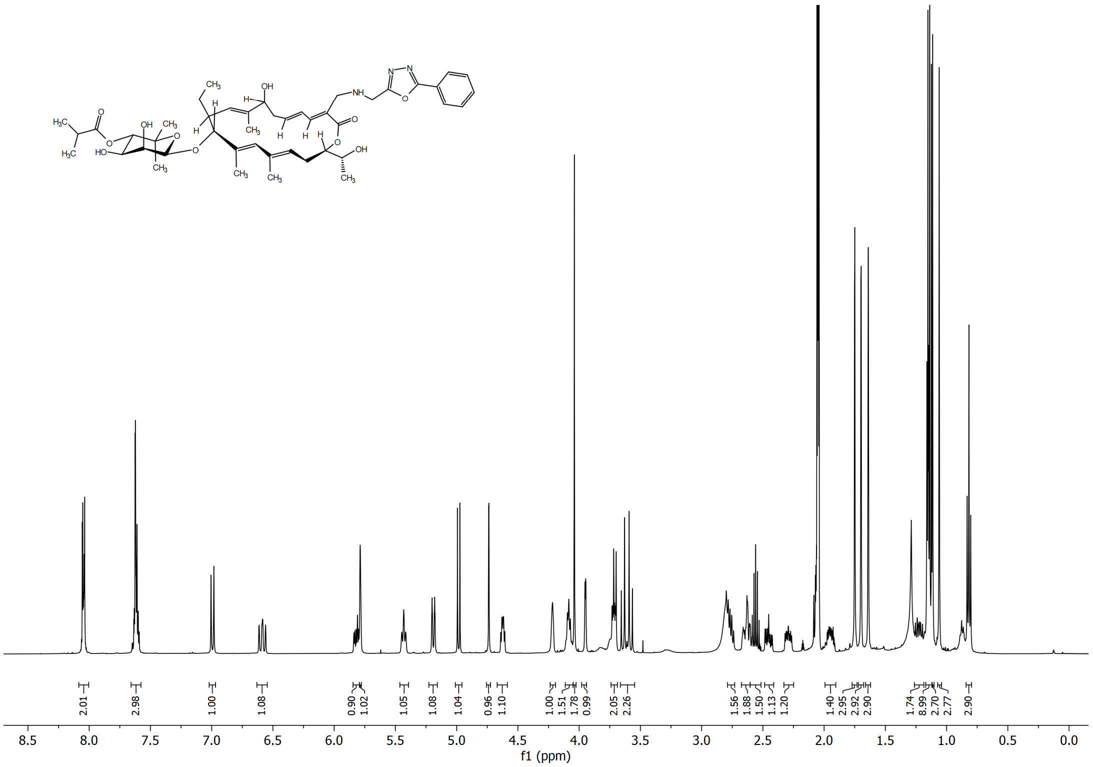

^{13}C NMR spectrum of **16** (126 MHz, acetone-d_6)

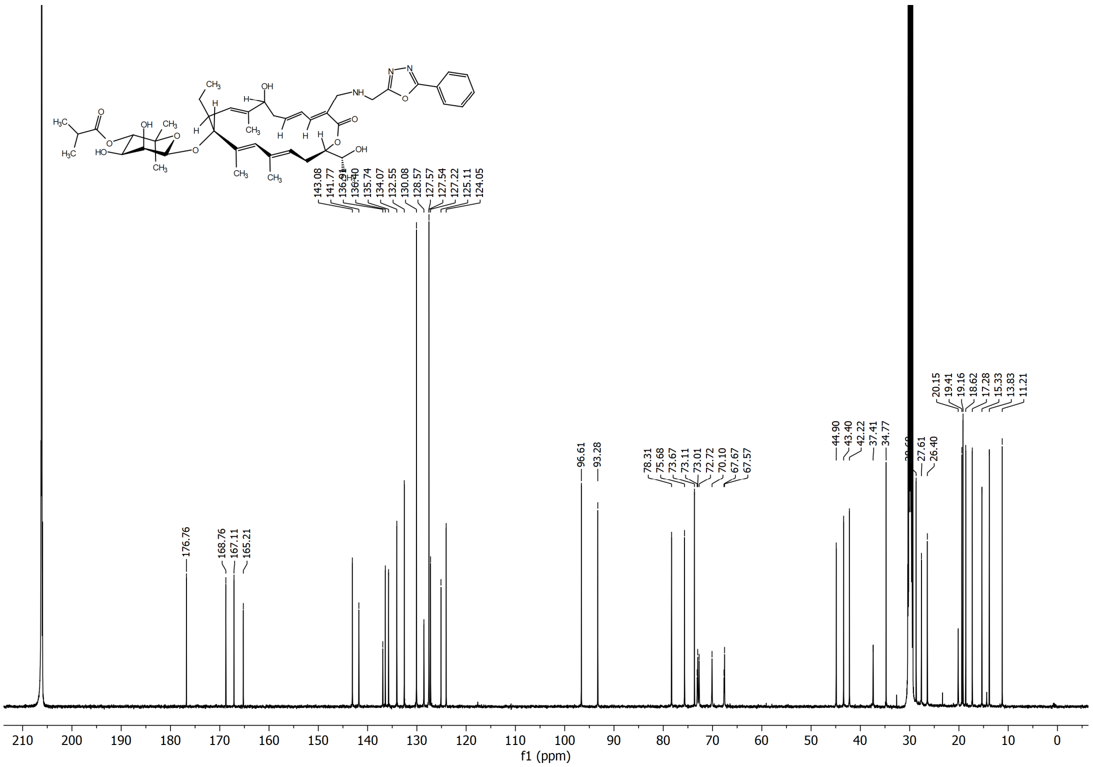

COSY spectrum of **16** (500 MHz, acetonitrile-d_3 + methanol-d_4)

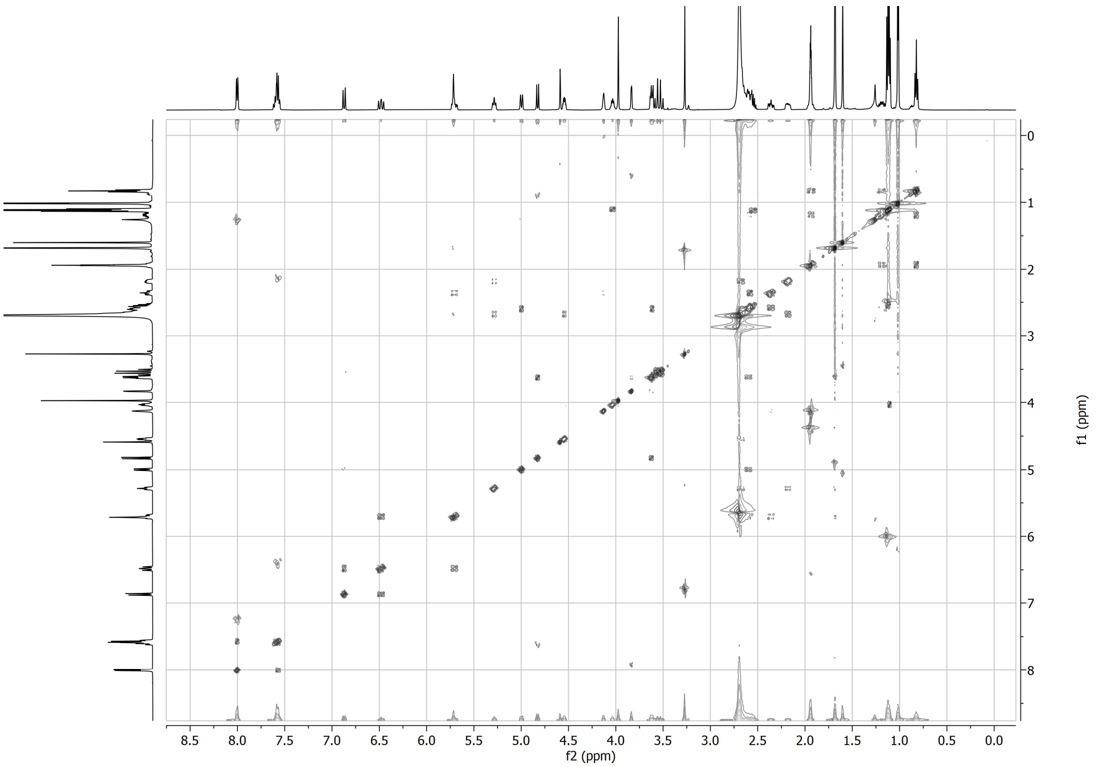

TOCSY spectrum of **16** (500 MHz, acetonitrile-d_3 + methanol-d_4)

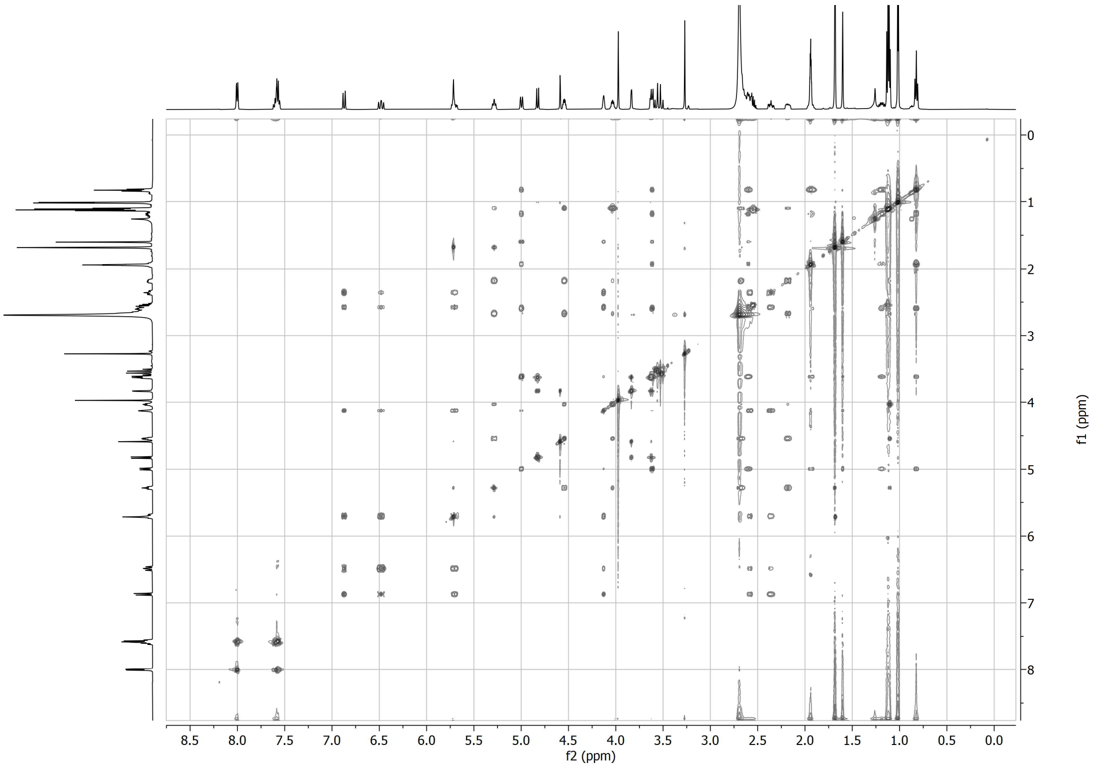

HSQC spectrum of **16** (500 MHz 126 MHz, acetonitrile-d_3 + methanol-d_4)

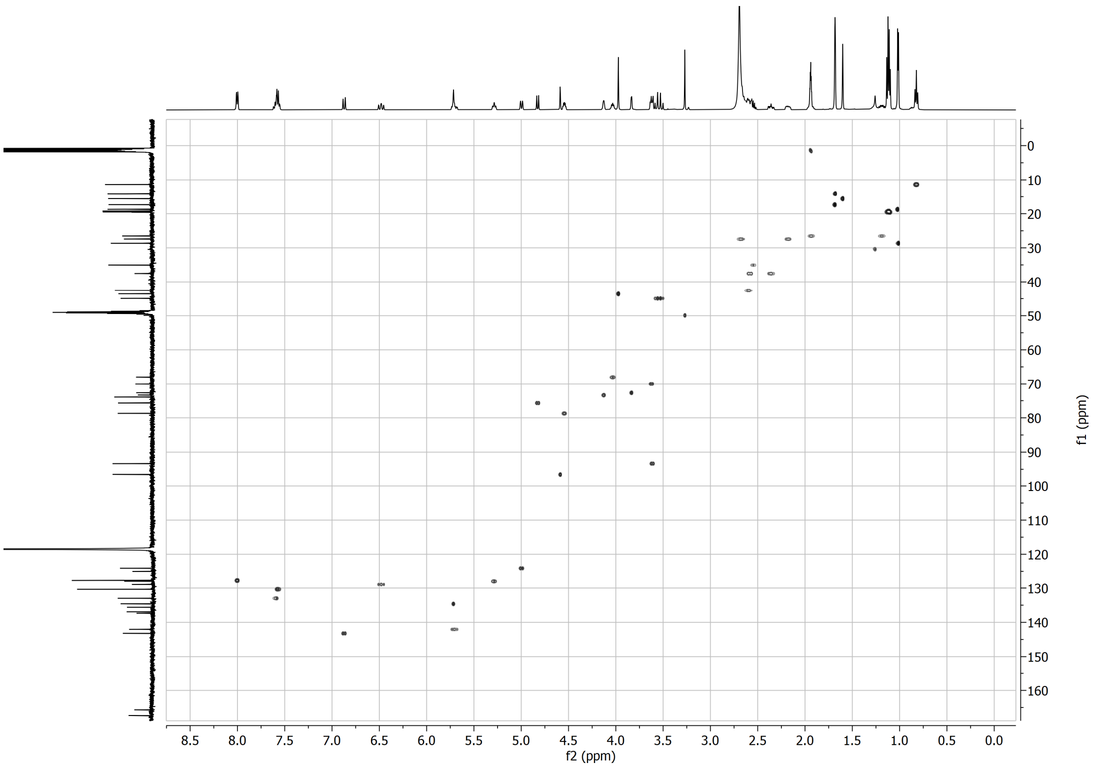

HMBC spectrum of **16** (500 MHz 126 MHz, acetonitrile-d_3 + methanol-d_4)

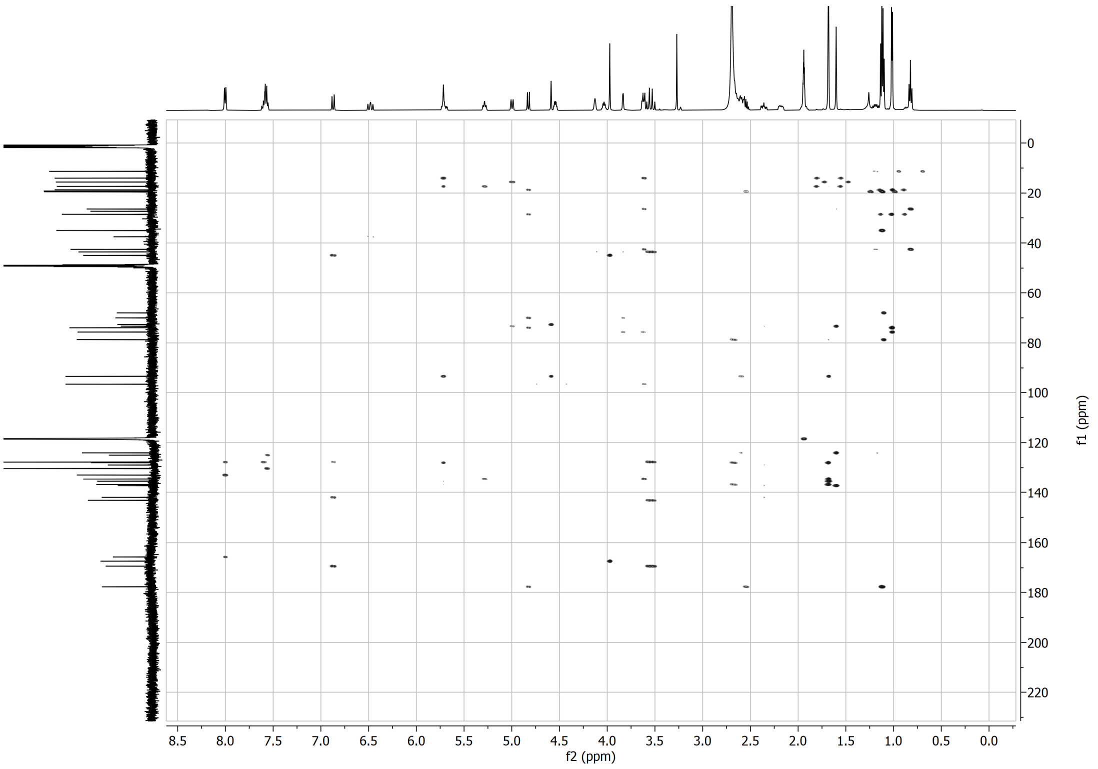

^{1}H NMR spectrum of **17** (500 MHz, $CDCl_3$)

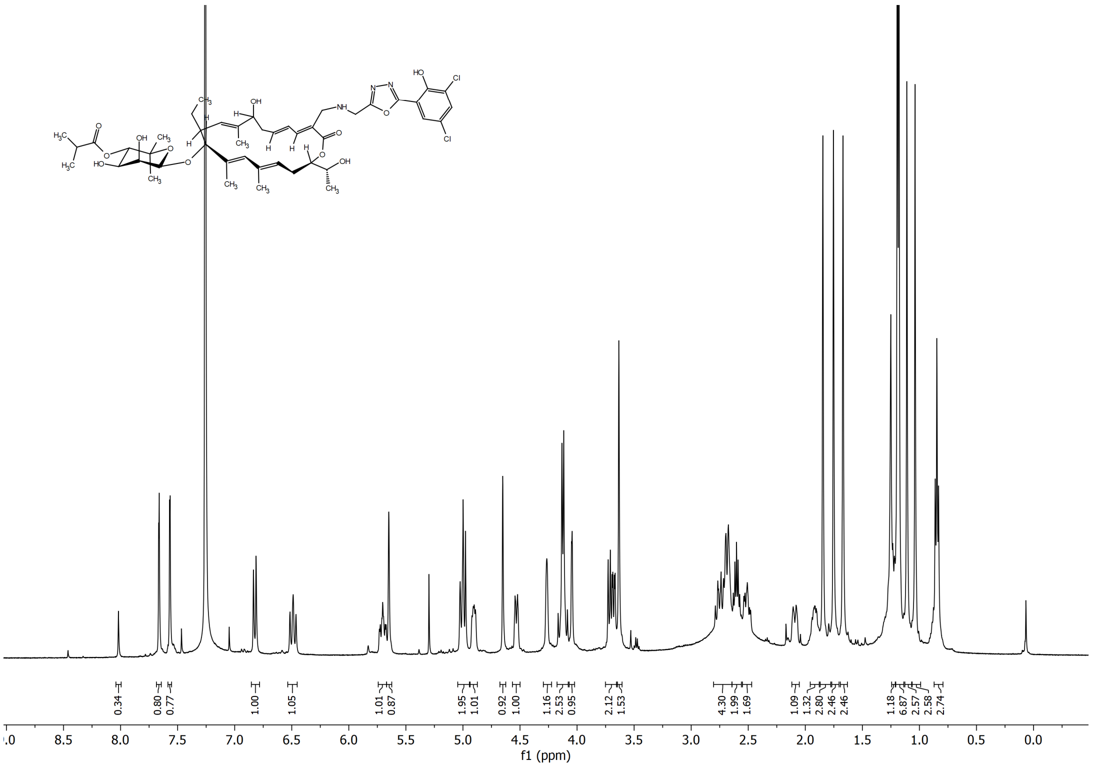

^{13}C NMR spectrum of **17** (126 MHz, $CDCl_3$)

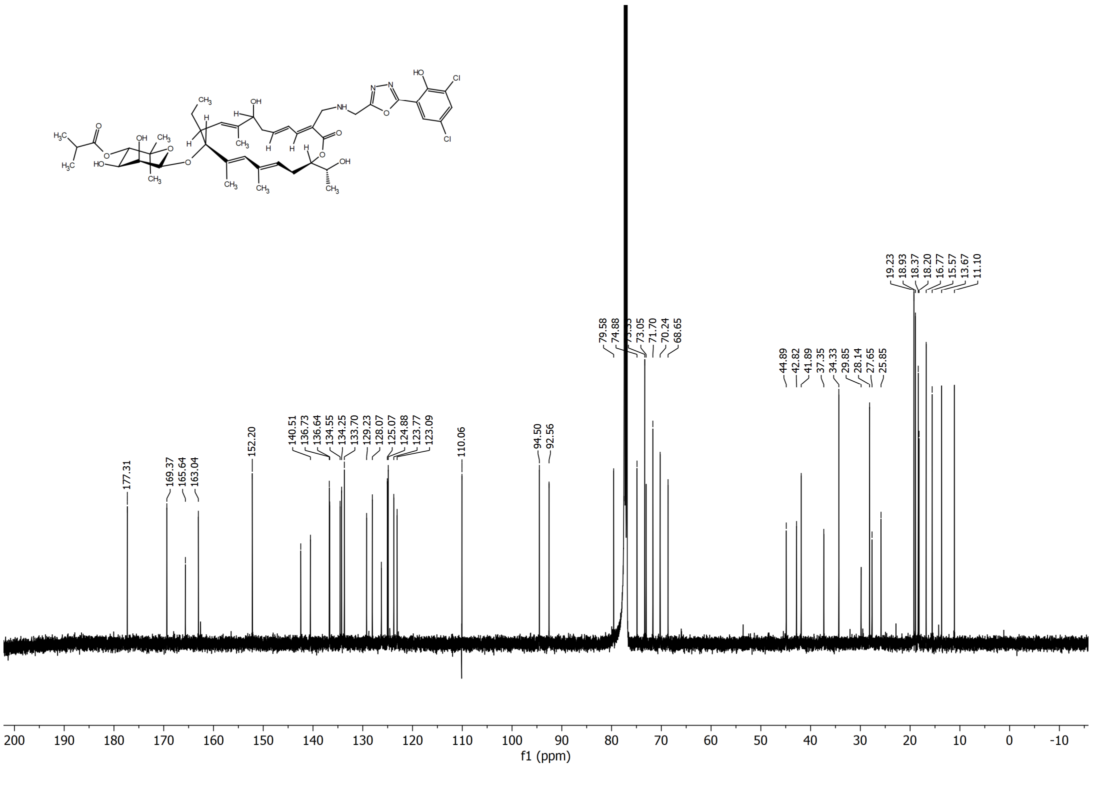

^{1}H NMR spectrum of **17a** (500 MHz, $CDCl_3$)

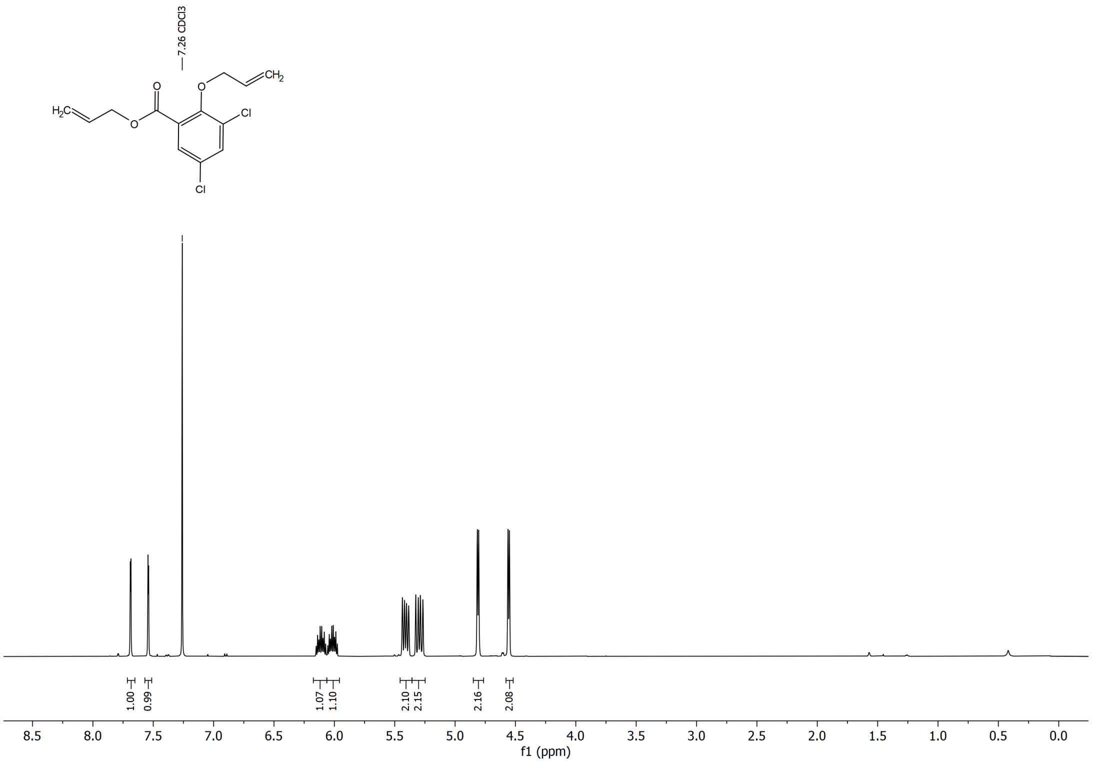

^{13}C NMR spectrum of **17a** (126 MHz, $CDCl_3$)

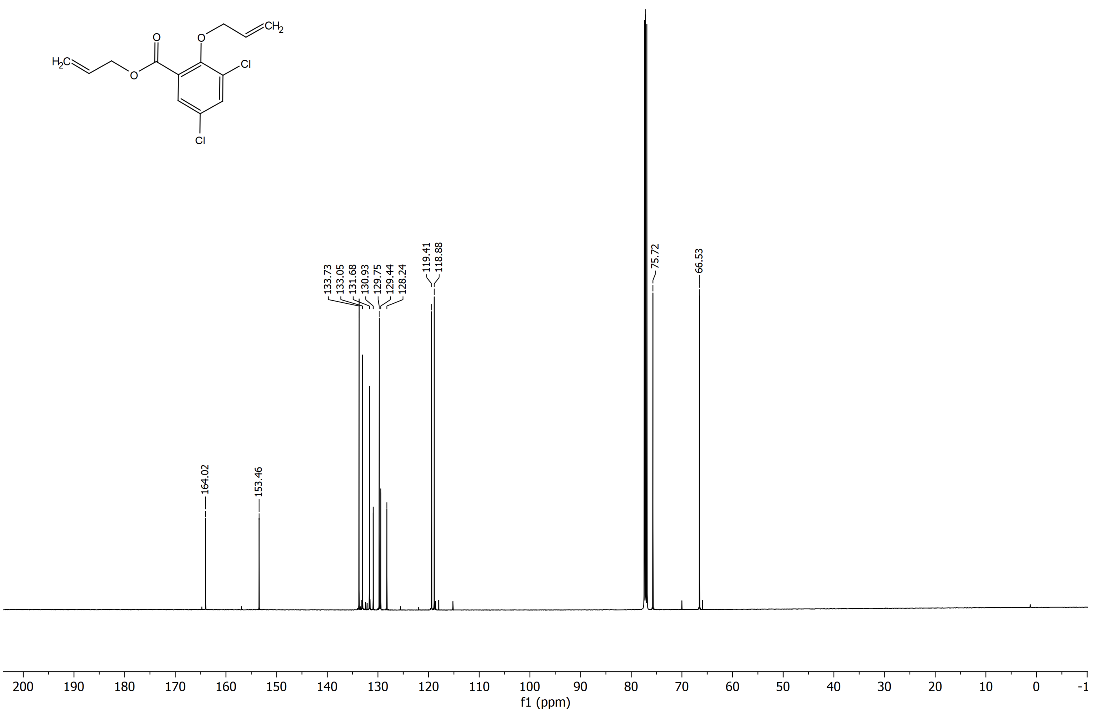

^{1}H NMR spectrum of **17b** (500 MHz, $CDCl_3$)

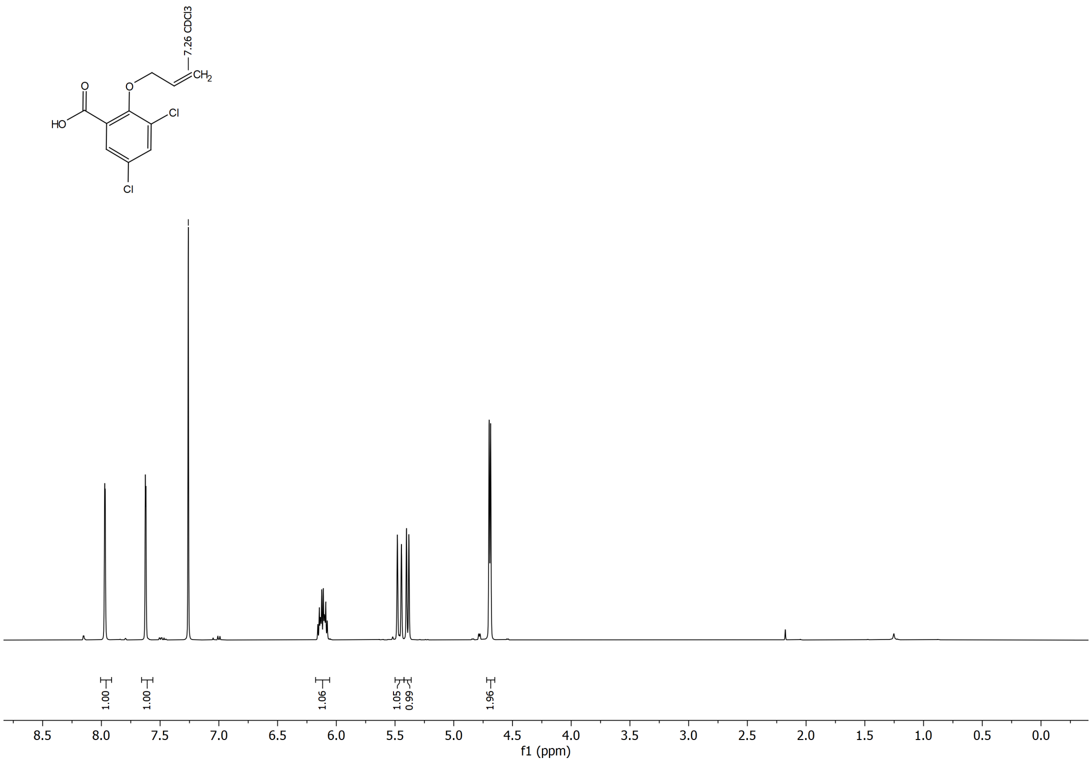

^{13}C NMR spectrum of **17b** (126 MHz, $CDCl_3$)

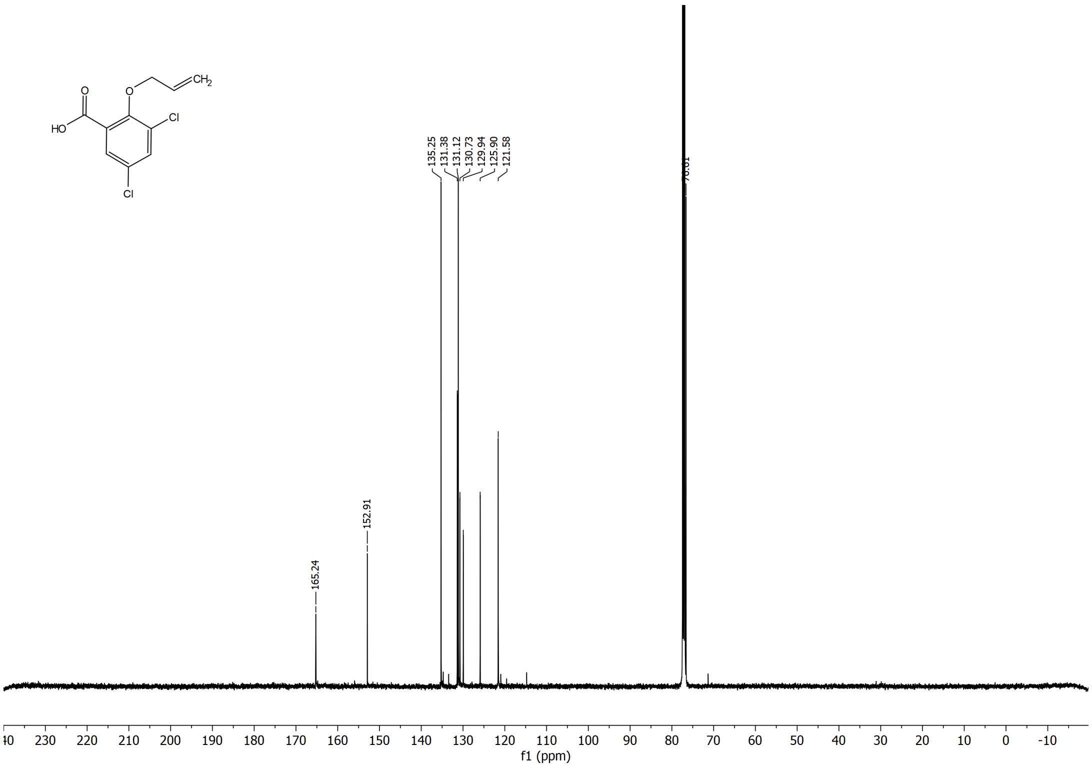

^{1}H NMR spectrum of **17c** (500 MHz, $CDCl_3$)

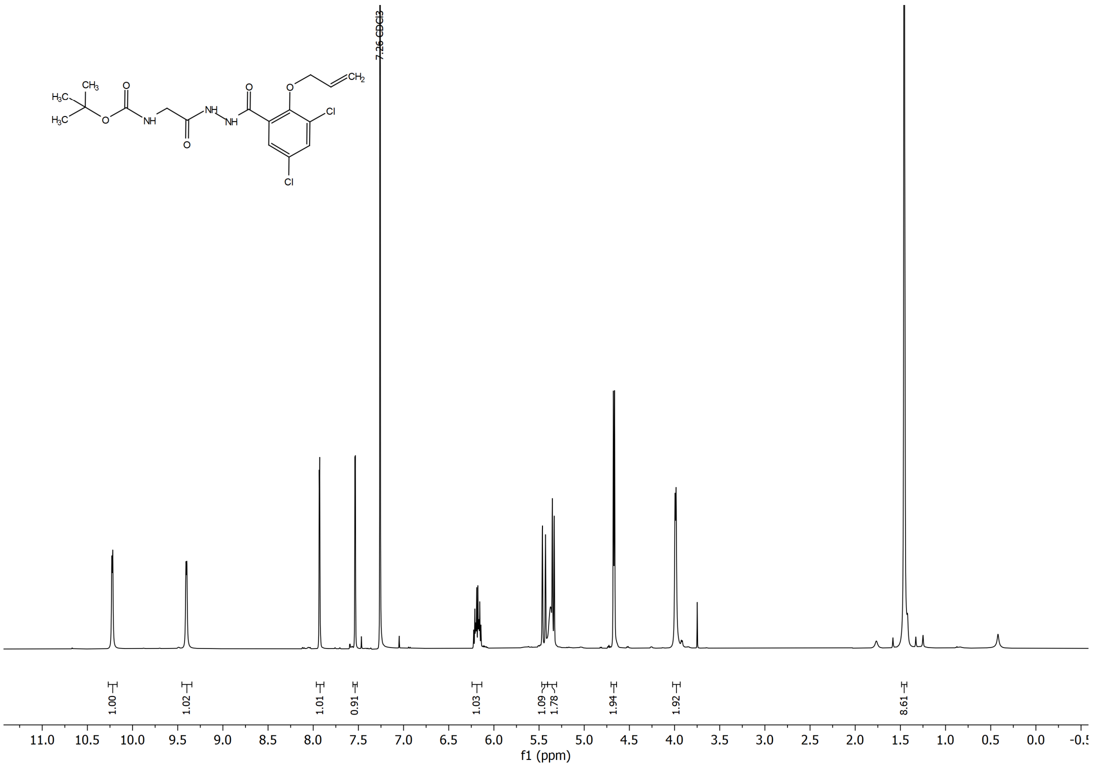

^{13}C NMR spectrum of **17c** (126 MHz, $CDCl_3$)

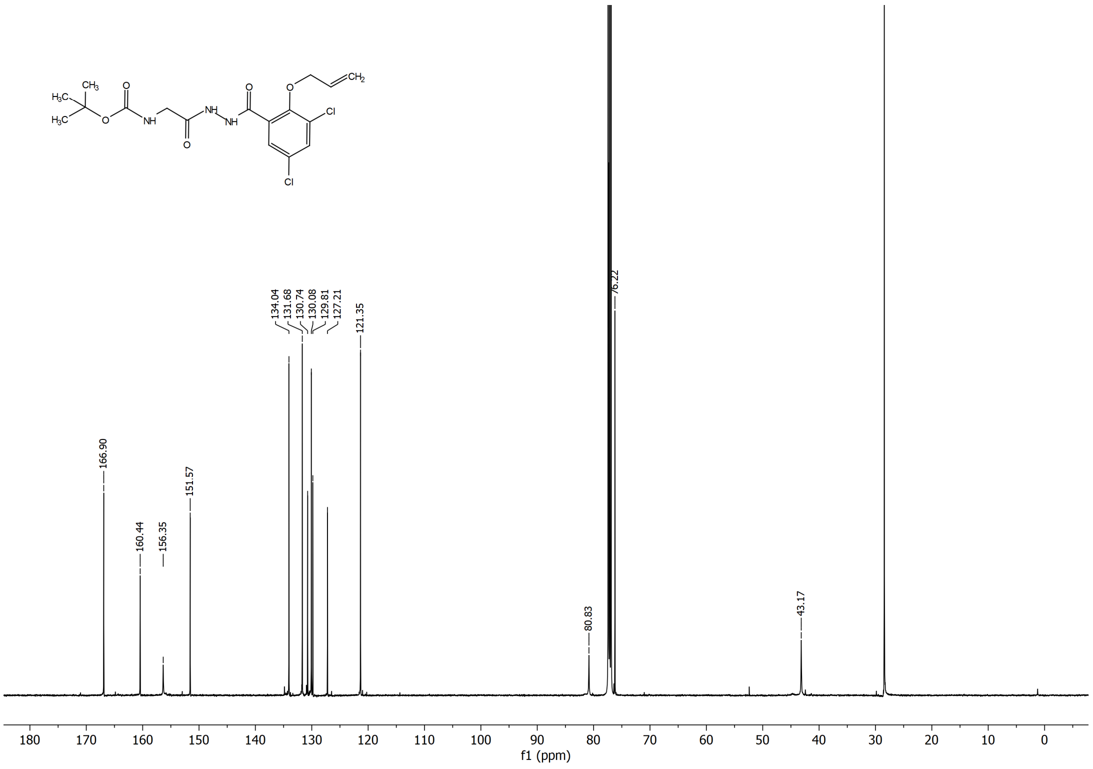

^{1}H NMR spectrum of **17d** (500 MHz, $CDCl_3$)

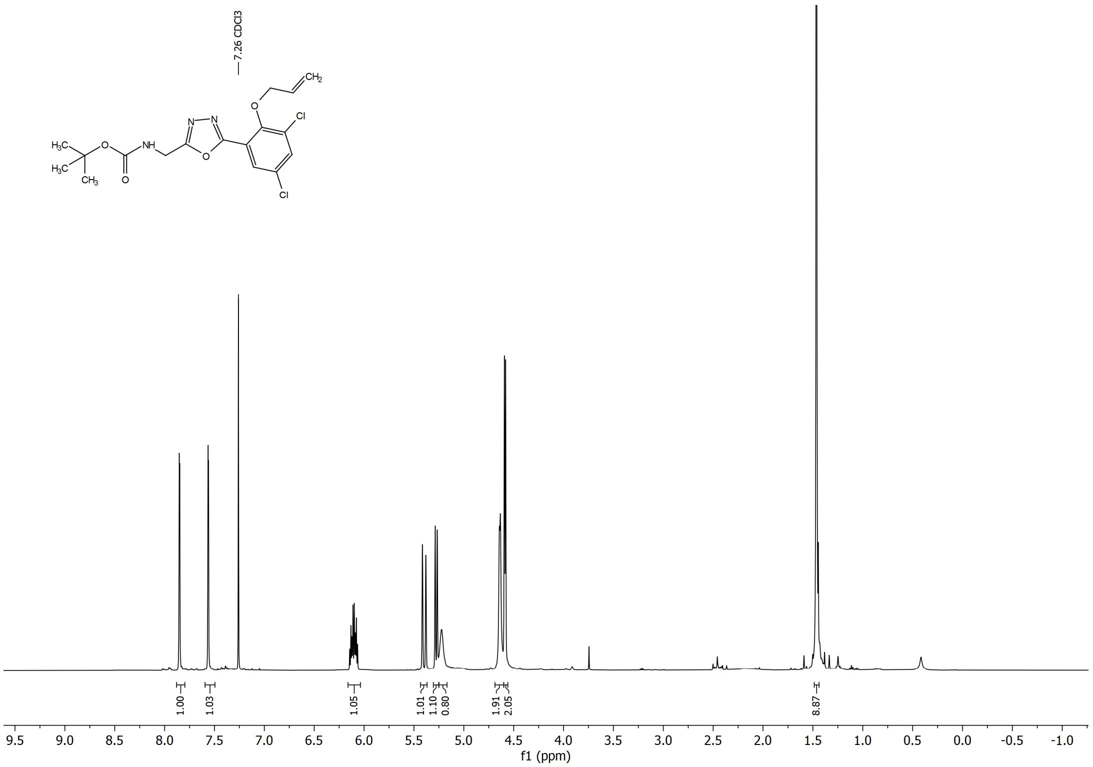

^{13}C NMR spectrum of **17d** (126 MHz, $CDCl_3$)

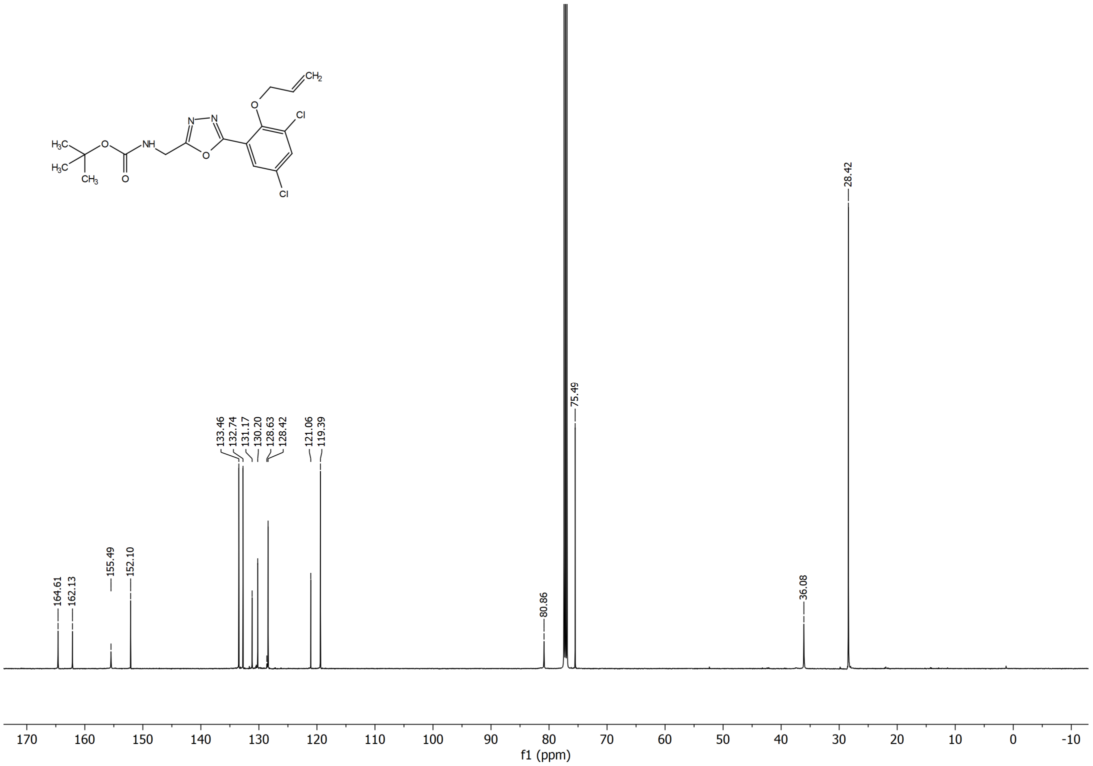

^{1}H NMR spectrum of **17e** (500 MHz, $CDCl_3$)

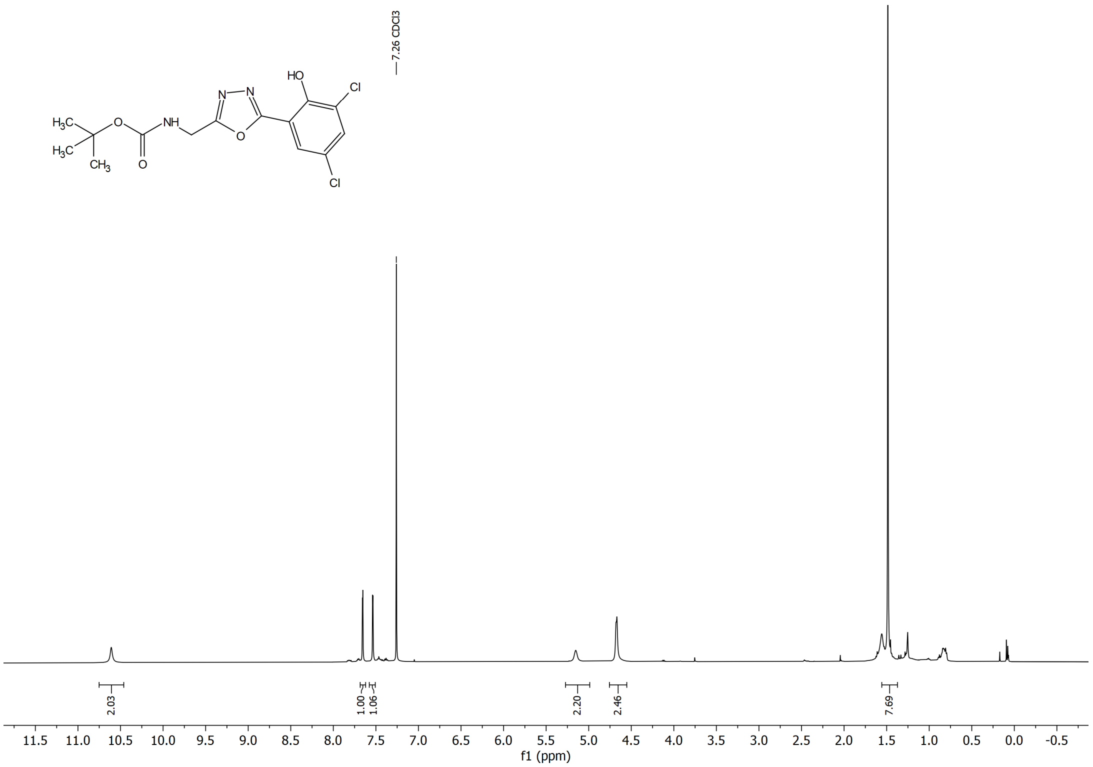

^{13}C NMR spectrum of **17e** (126 MHz, $CDCl_3$)

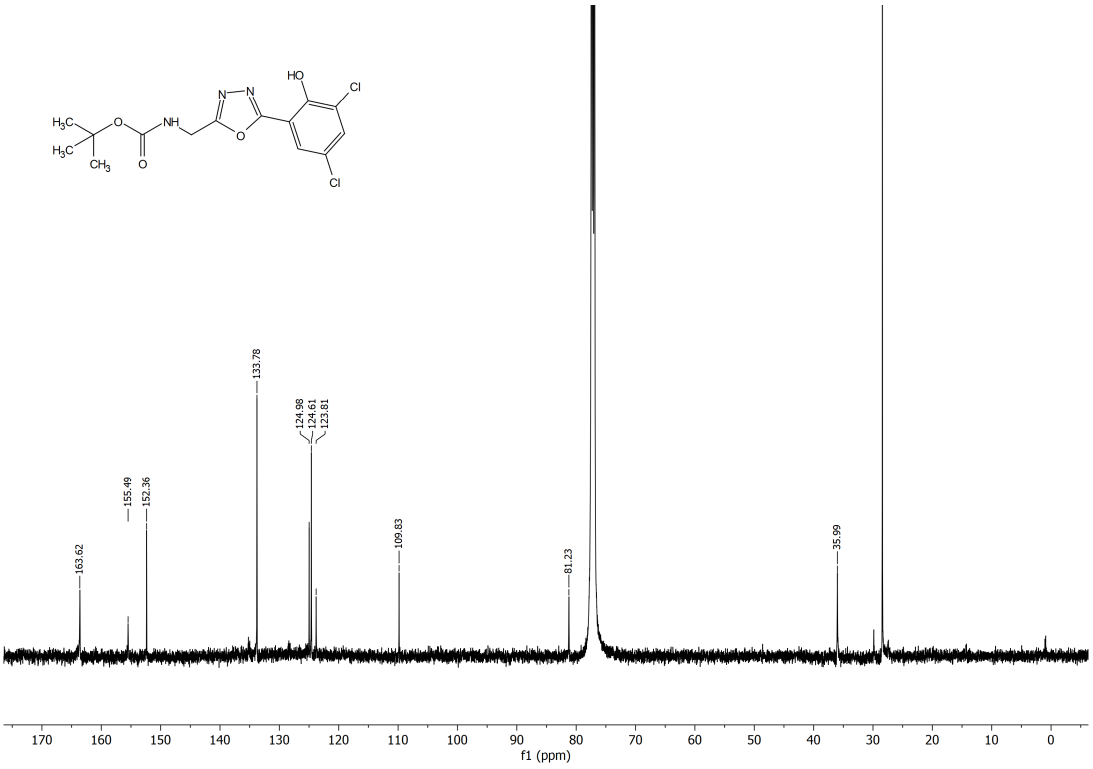

^{1}H NMR spectrum of **18** (500 MHz, $CDCl_3$)

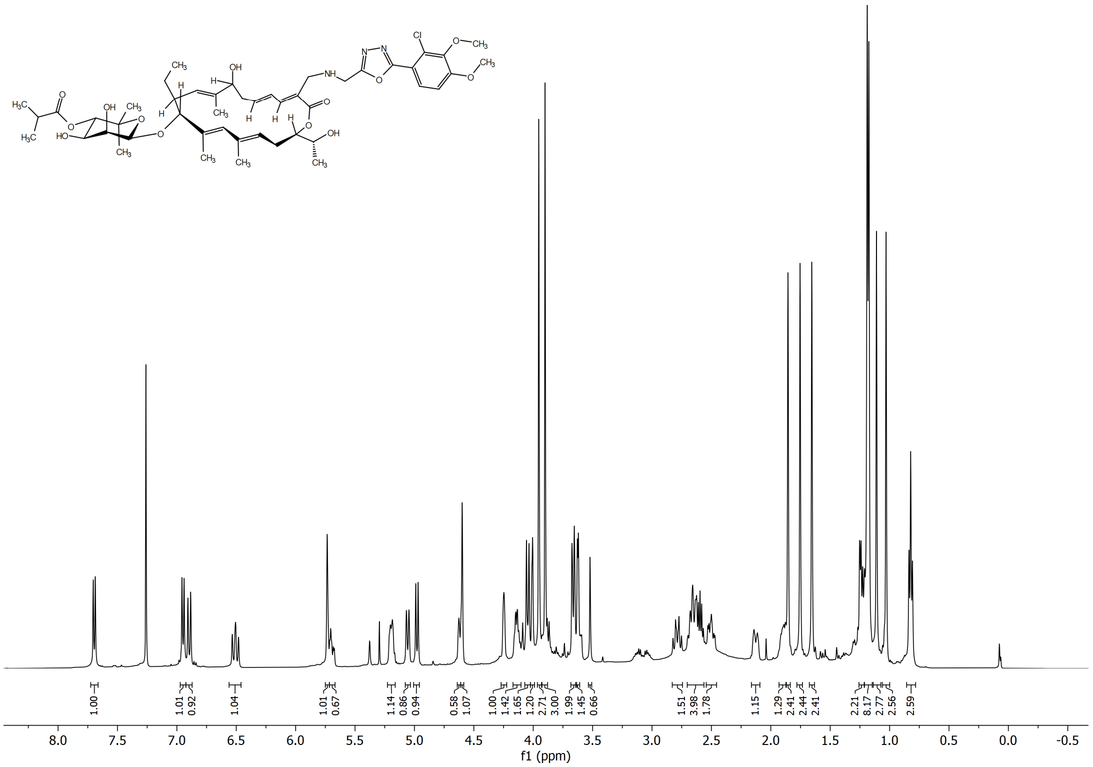

^{13}C NMR spectrum of **18** (126 MHz, $CDCl_3$)

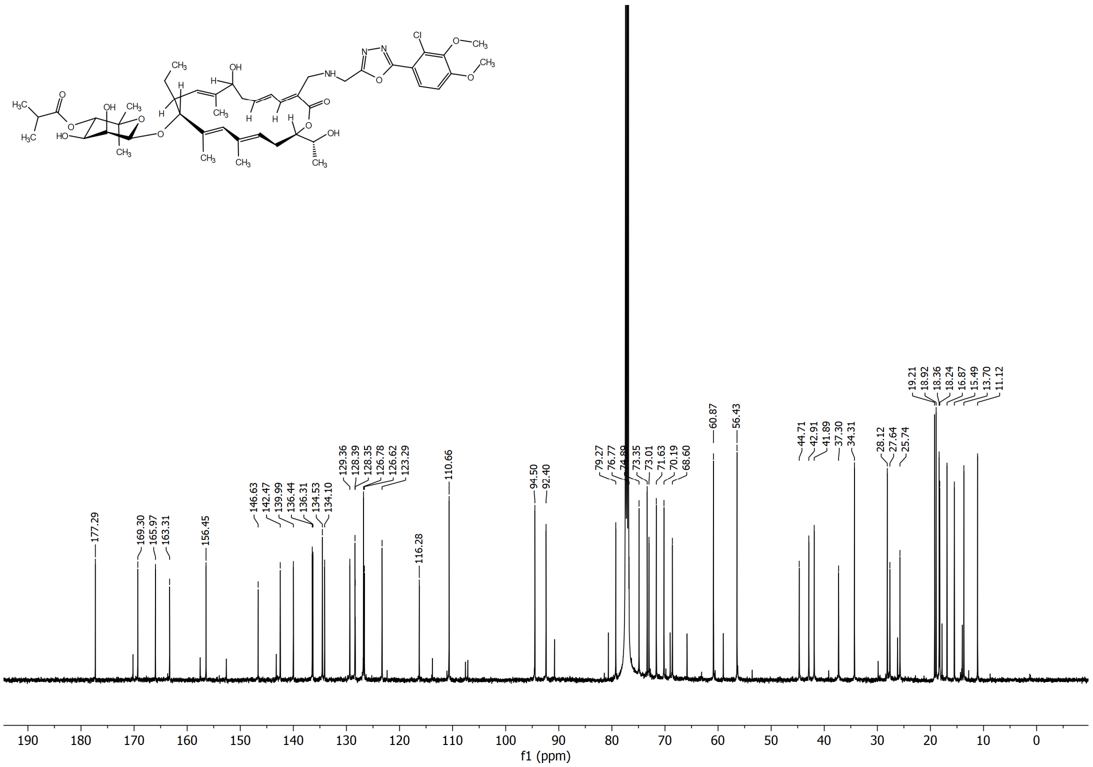

^{1}H NMR spectrum of **18a** (500 MHz, $CDCl_3$)

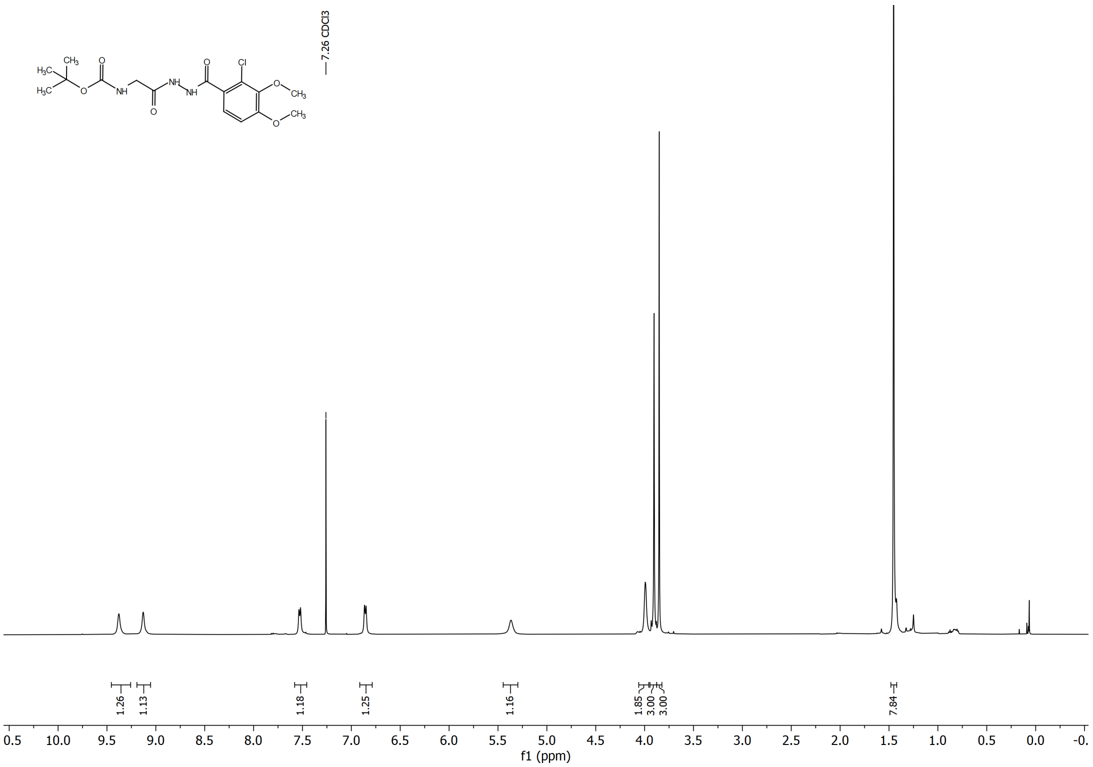

^{13}C NMR spectrum of **18a** (126 MHz, $CDCl_3$)

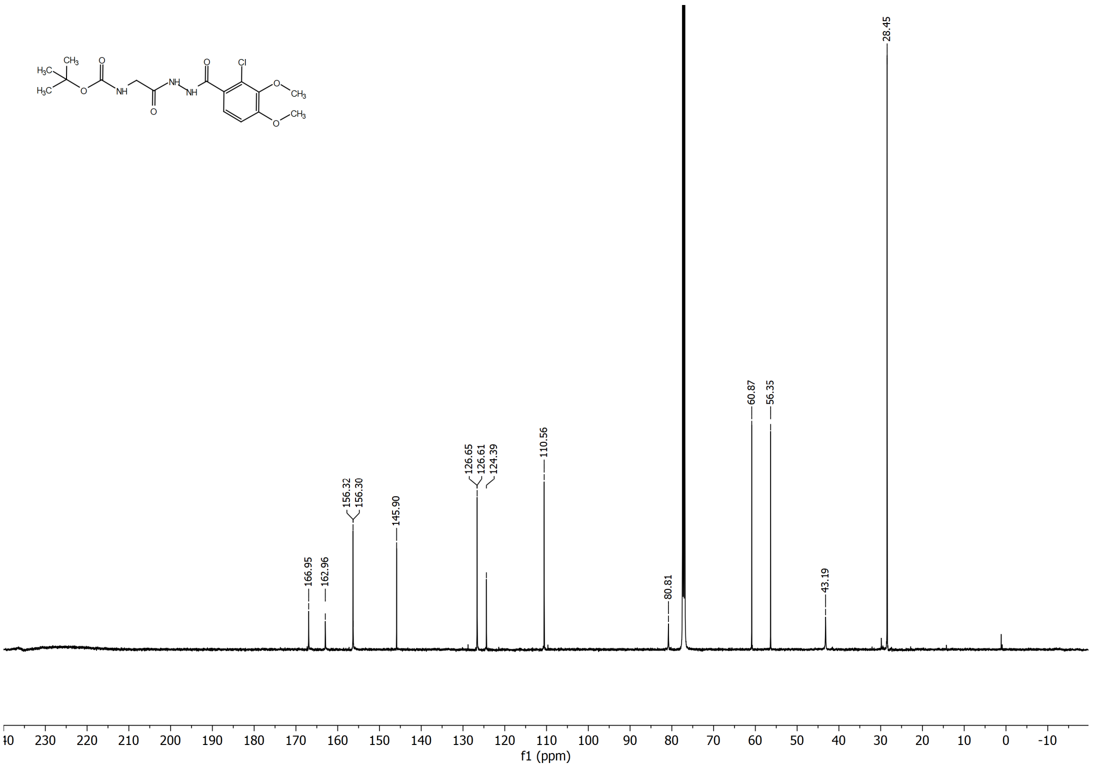

^{1}H NMR spectrum of **18b** (500 MHz, $CDCl_3$)

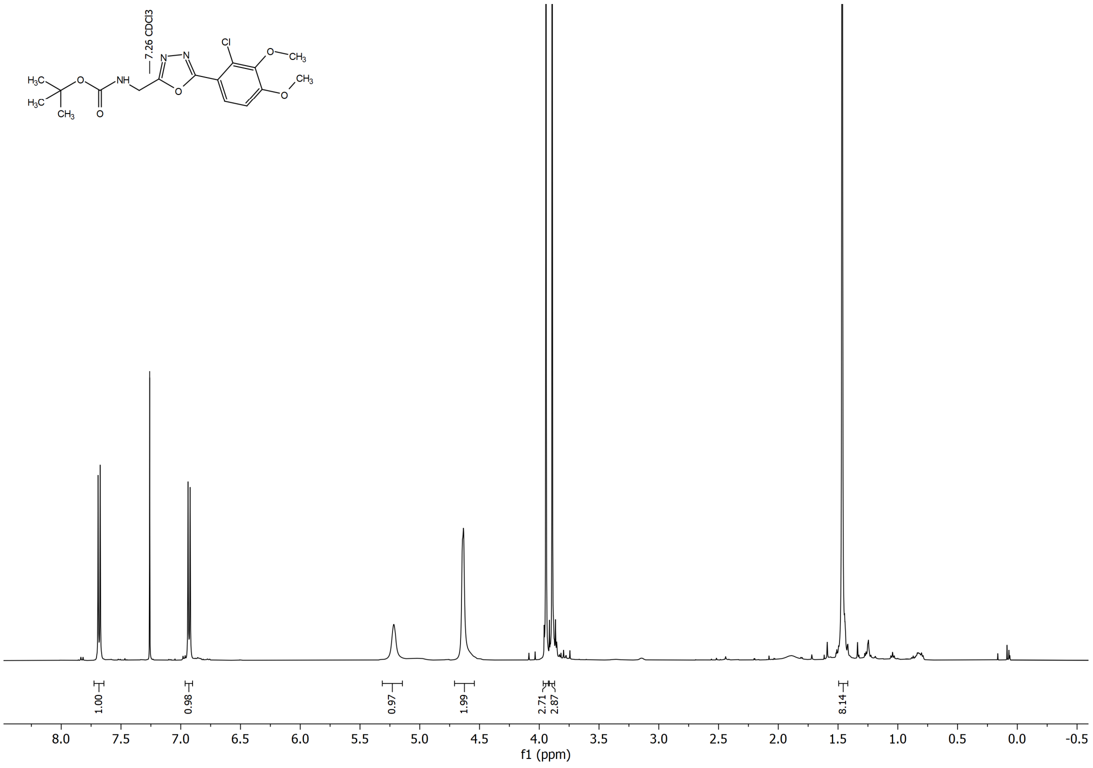

^{13}C NMR spectrum of **18b** (126 MHz, $CDCl_3$)

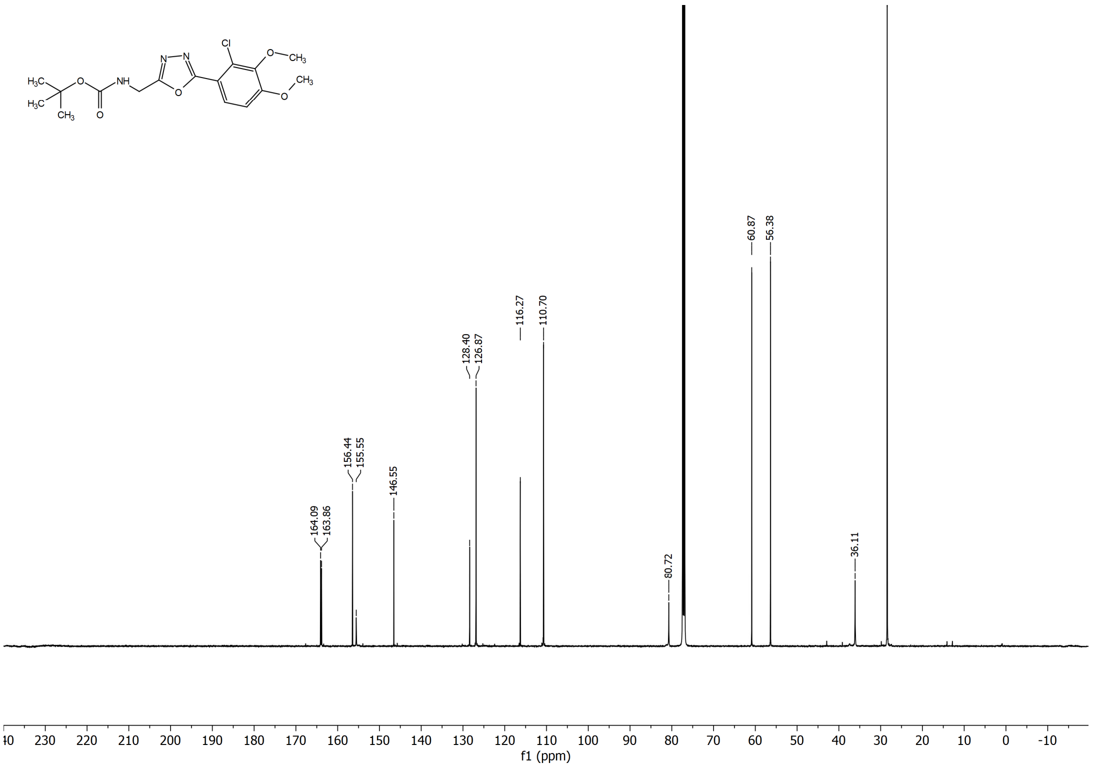

^{1}H NMR spectrum of **19** (500 MHz, $CDCl_3$)

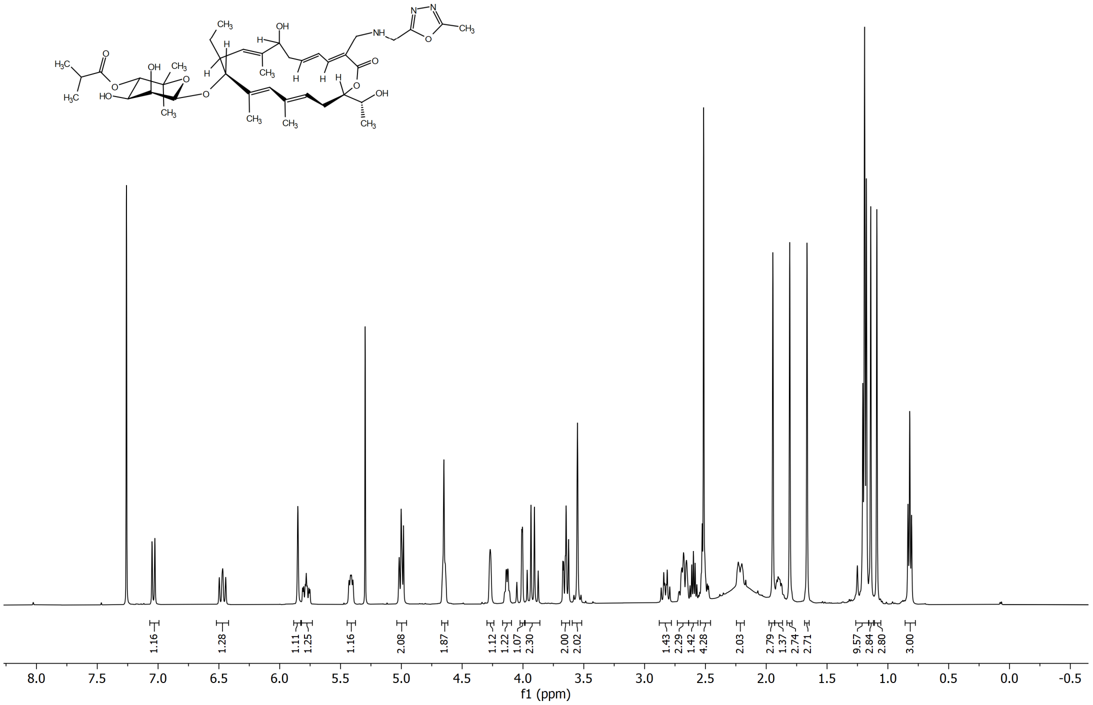

^{13}C NMR spectrum of **19** (126 MHz, $CDCl_3$)

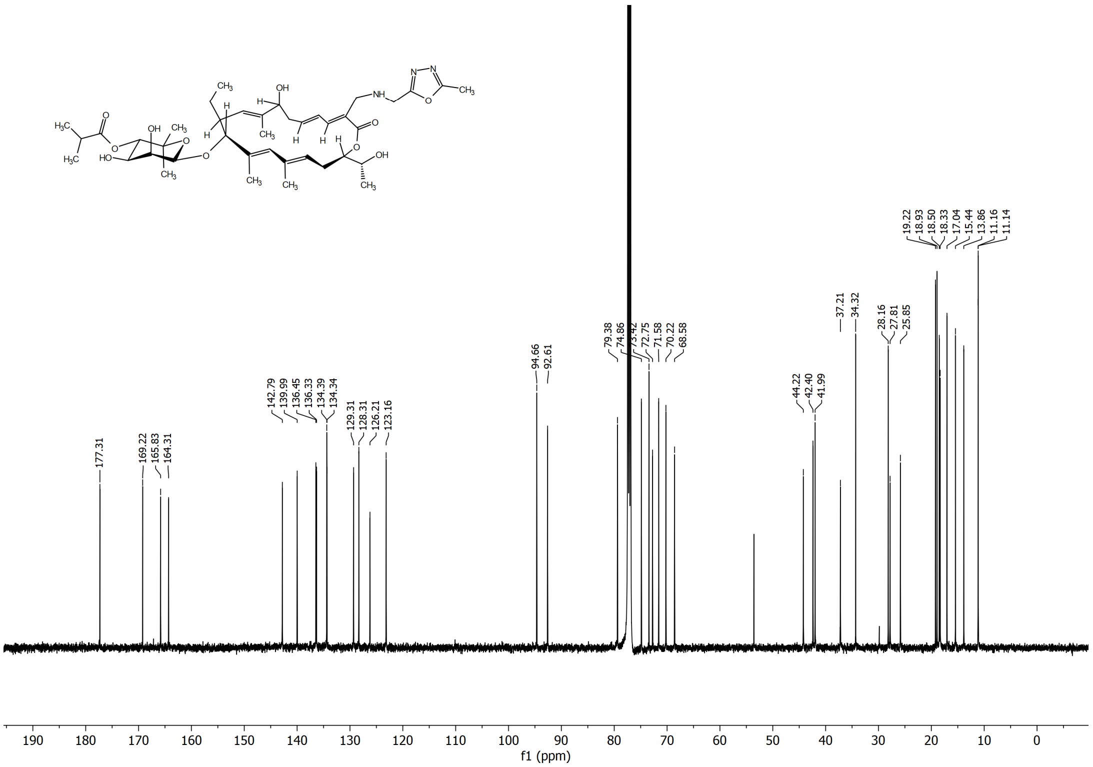

^{1}H NMR spectrum of **20** (500 MHz, $CDCl_3$)

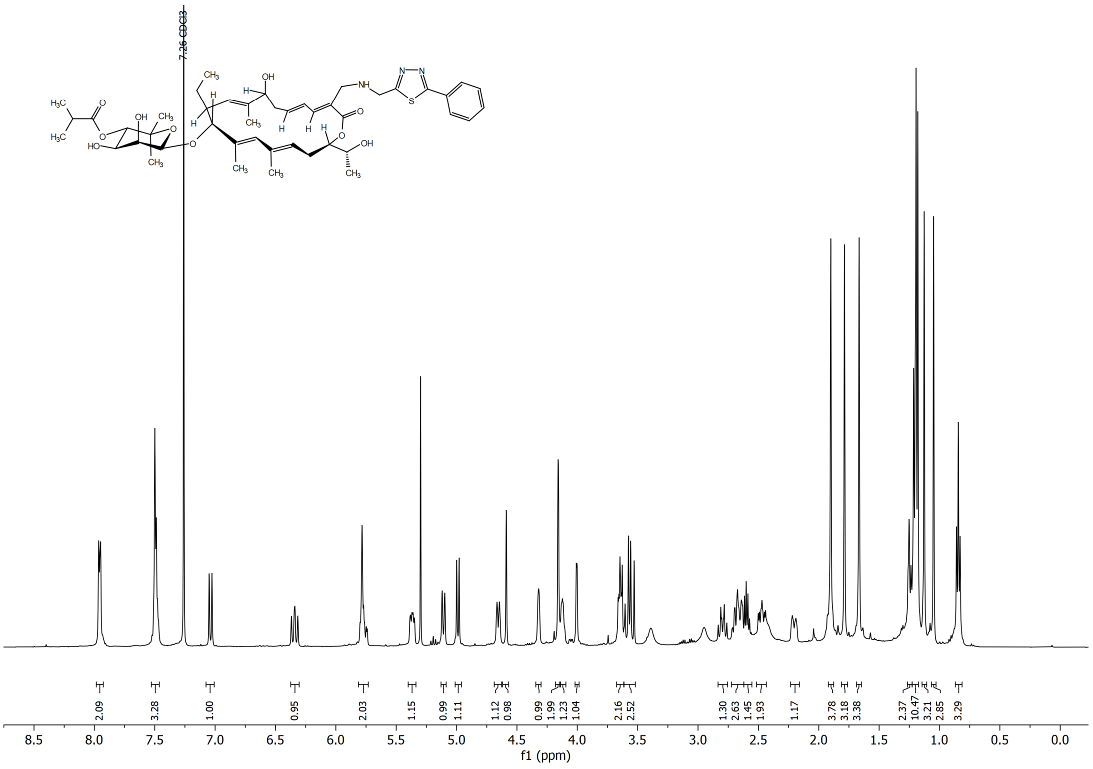

^{13}C NMR spectrum of **20** (126 MHz, $CDCl_3$)

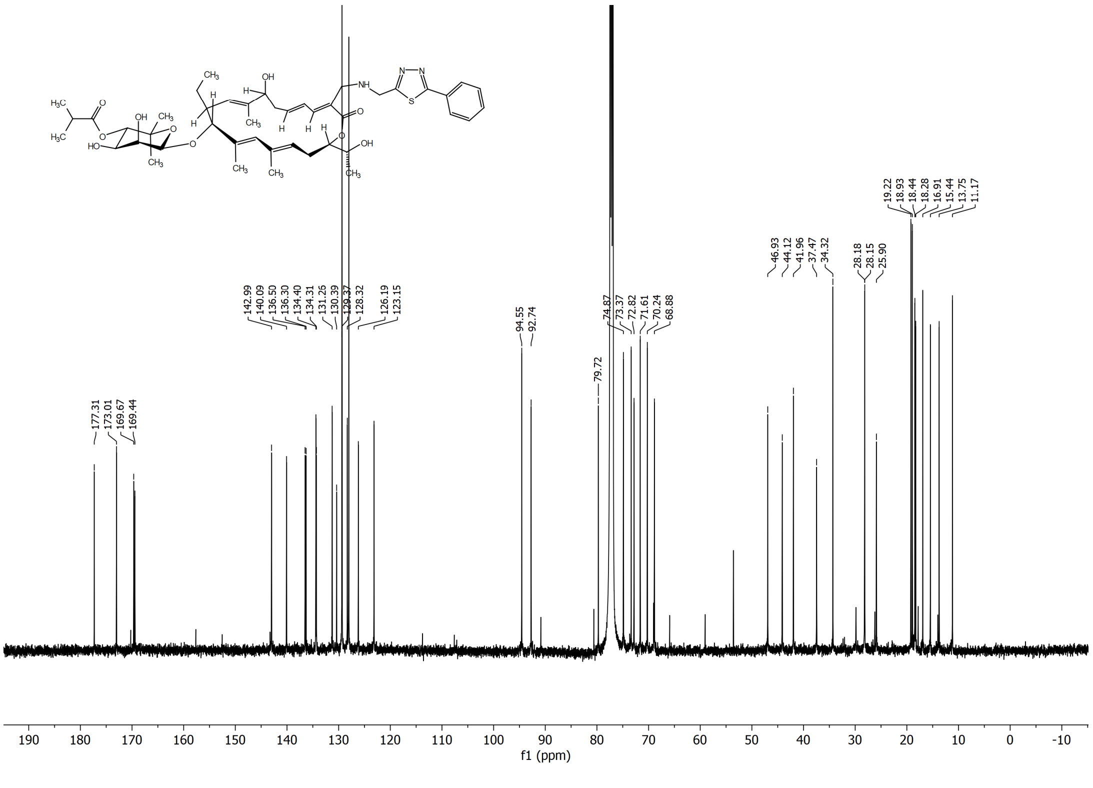

^{1}H NMR spectrum of **20a** (500 MHz, DMSO-d_6)

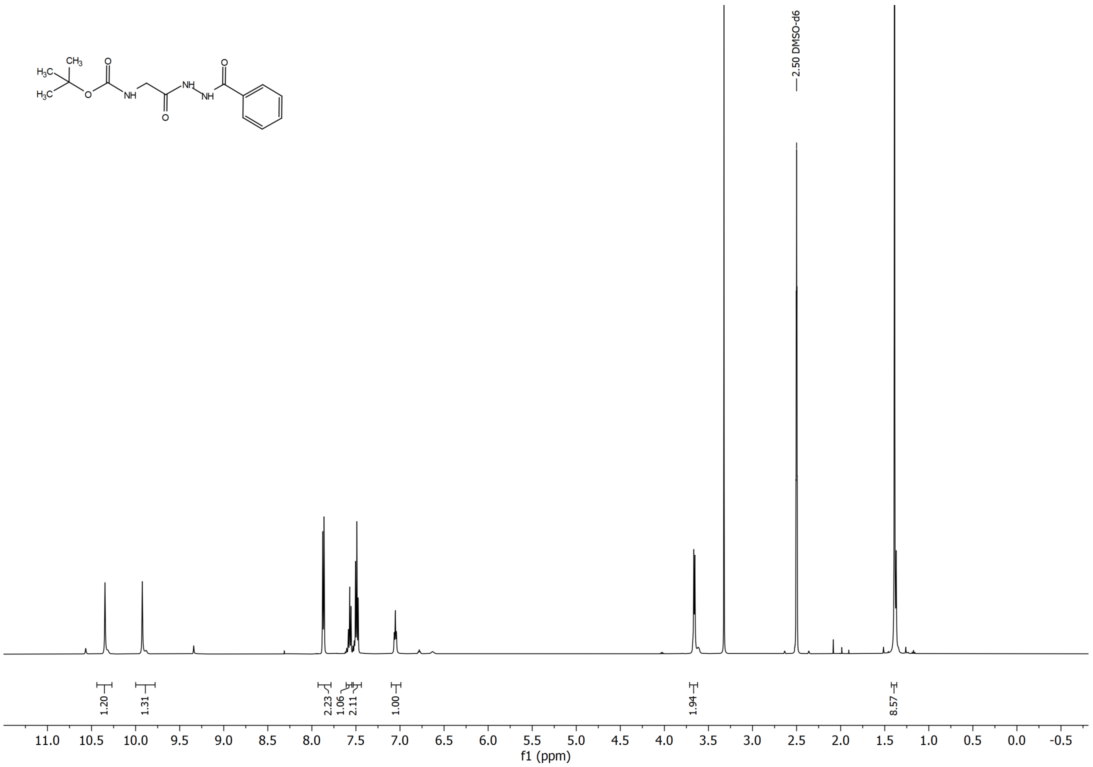

^{13}C NMR spectrum of **20a** (126 MHz, DMSO-d_6)

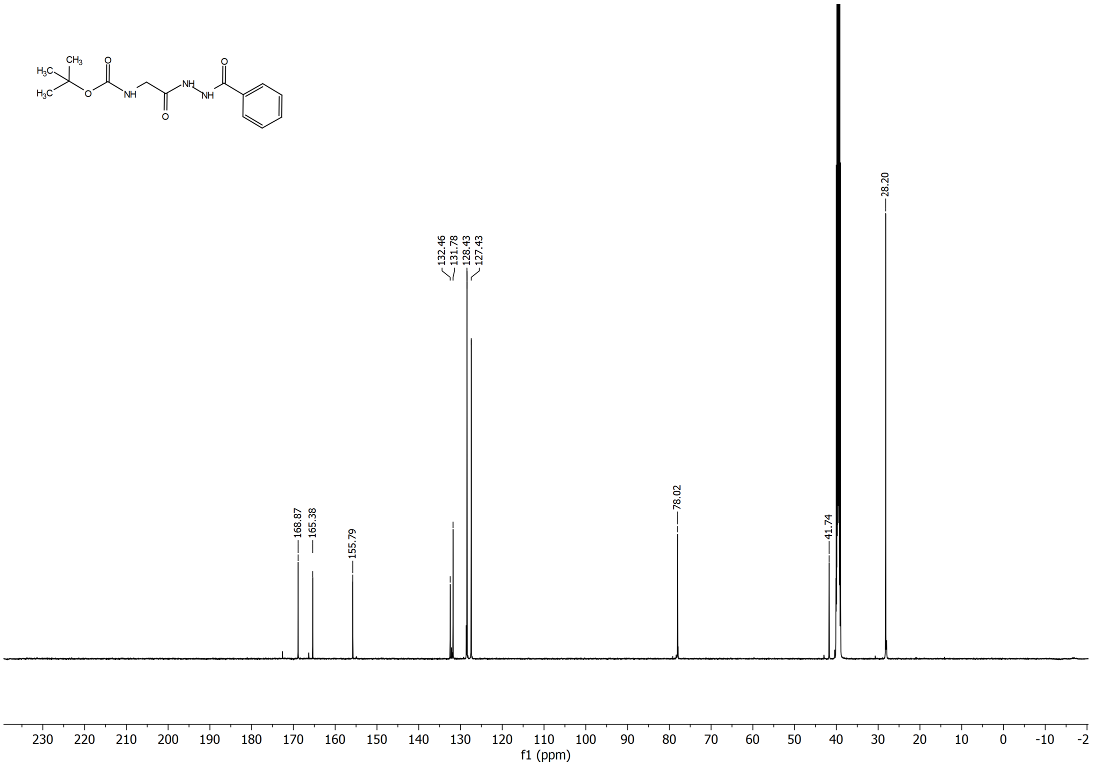

^{1}H NMR spectrum of **20b** (500 MHz, $CDCl_3$)

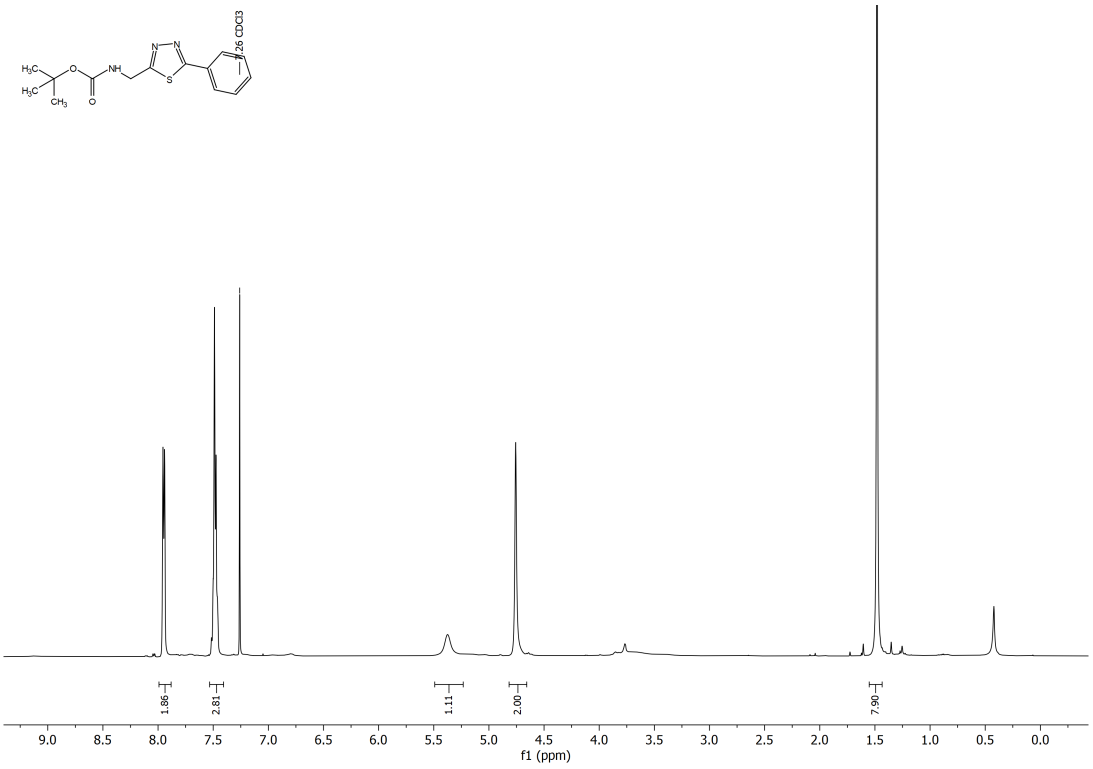

^{13}C NMR spectrum of **20b** (126 MHz, $CDCl_3$)

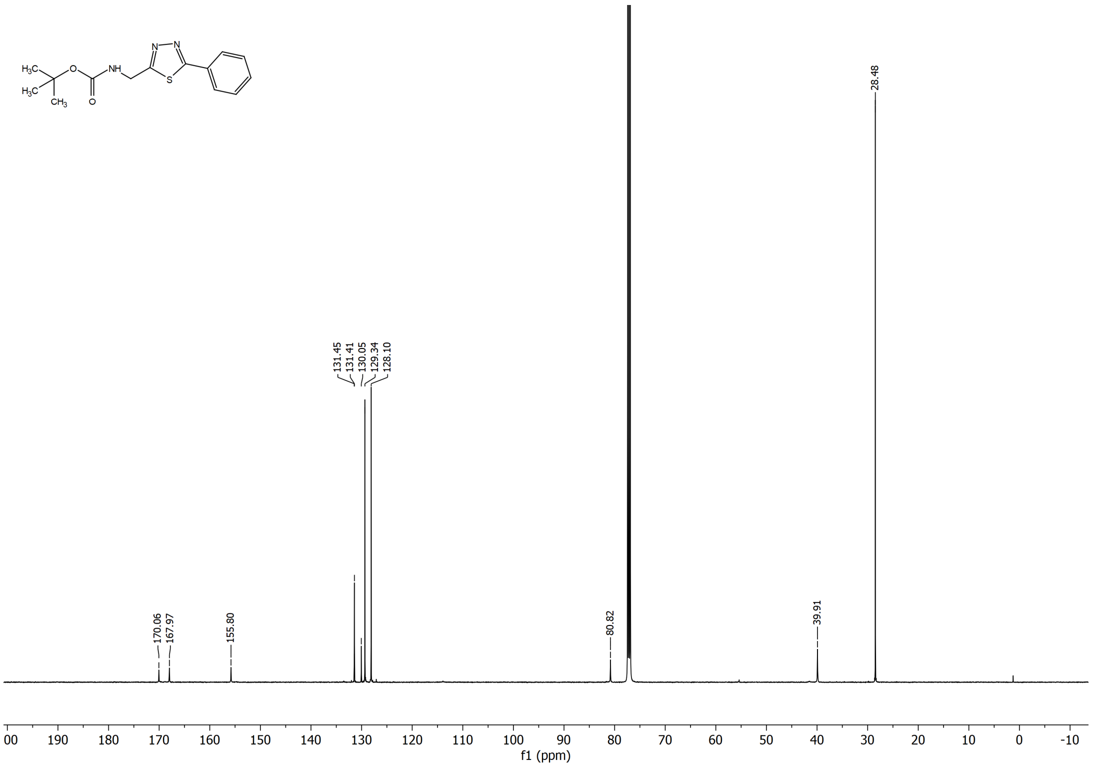

^{1}H NMR spectrum of 21 (500 MHz, $CDCl_3$)

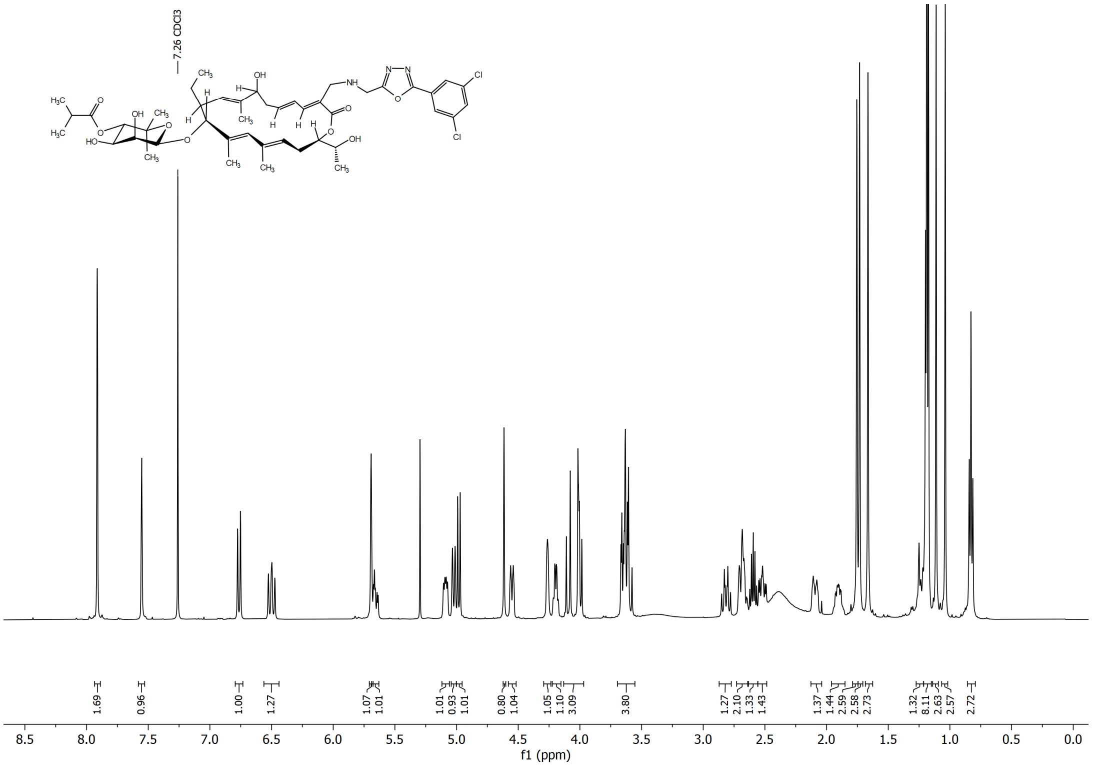

^{13}C NMR spectrum of 21 (126 MHz, $CDCl_3$)

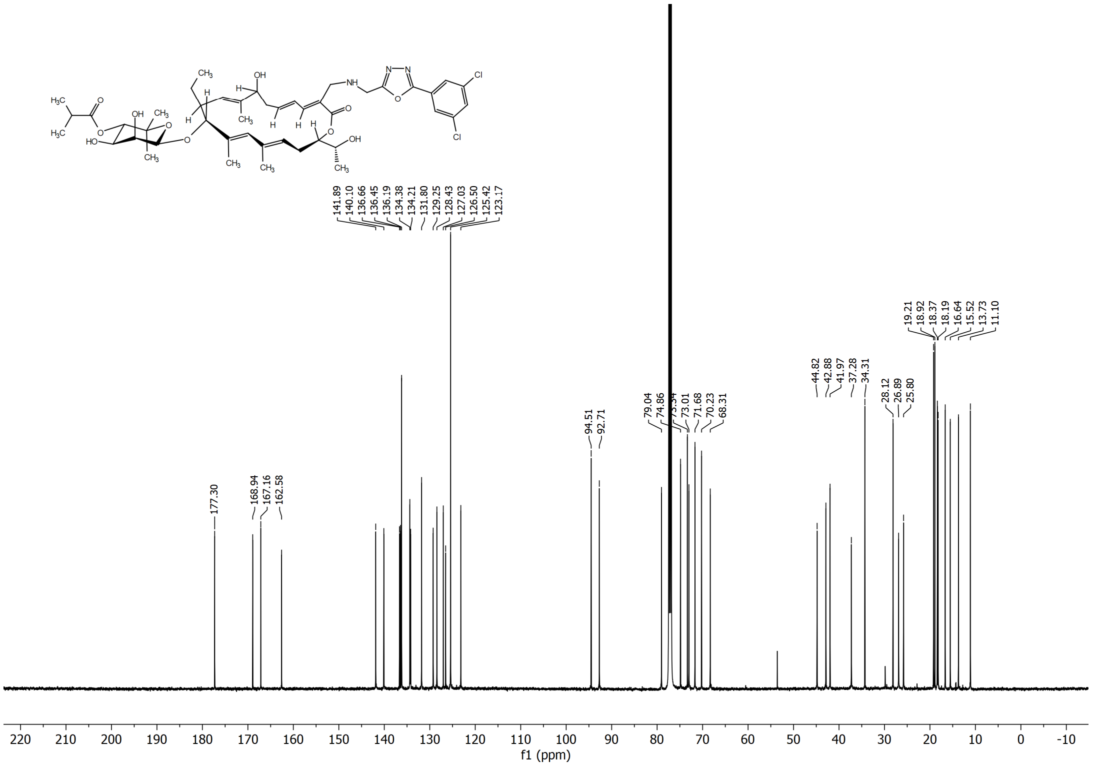

^{1}H NMR spectrum of **21a** (500 MHz, DMSO-d_6)

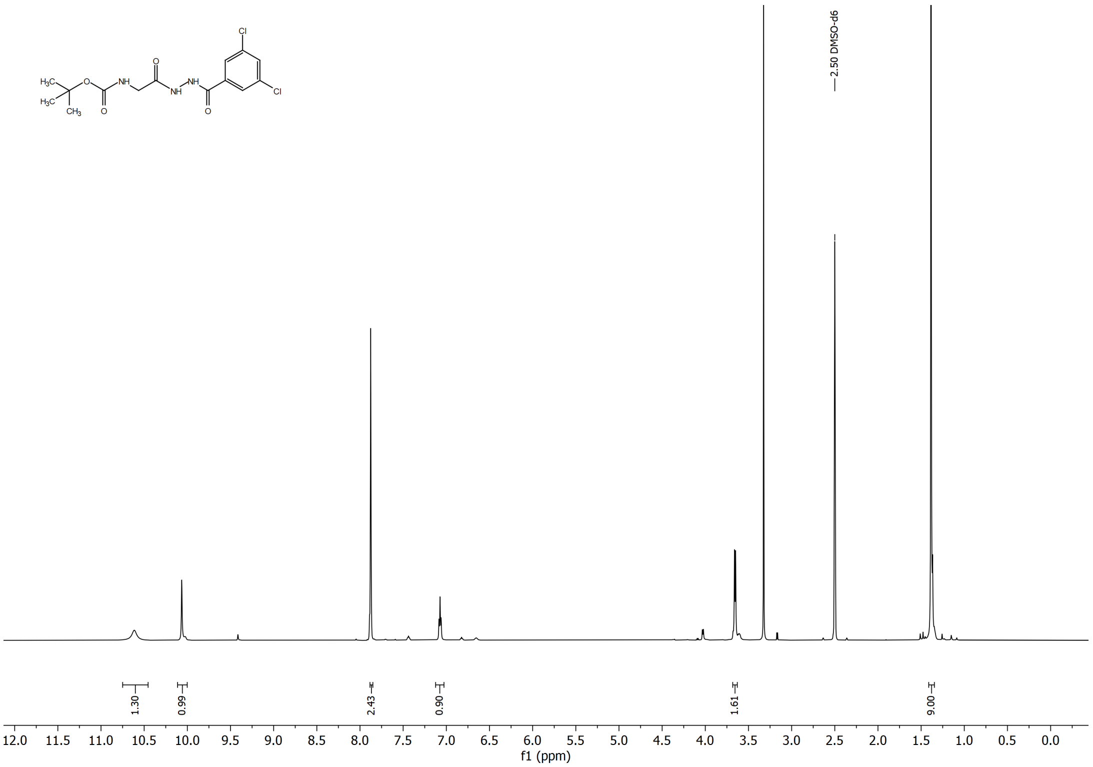

^{13}C NMR spectrum of **21a** (126 MHz, DMSO-d_6)

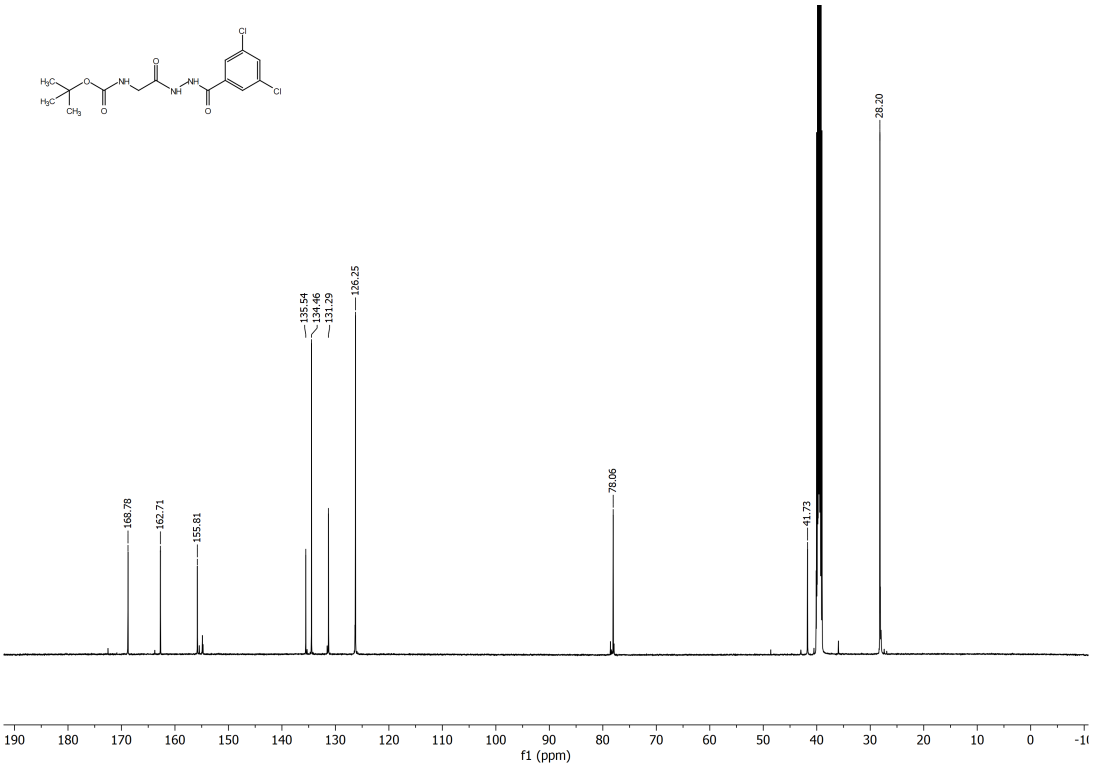

^{1}**H NMR** spectrum of **21b** (500 MHz, $CDCl_3$)

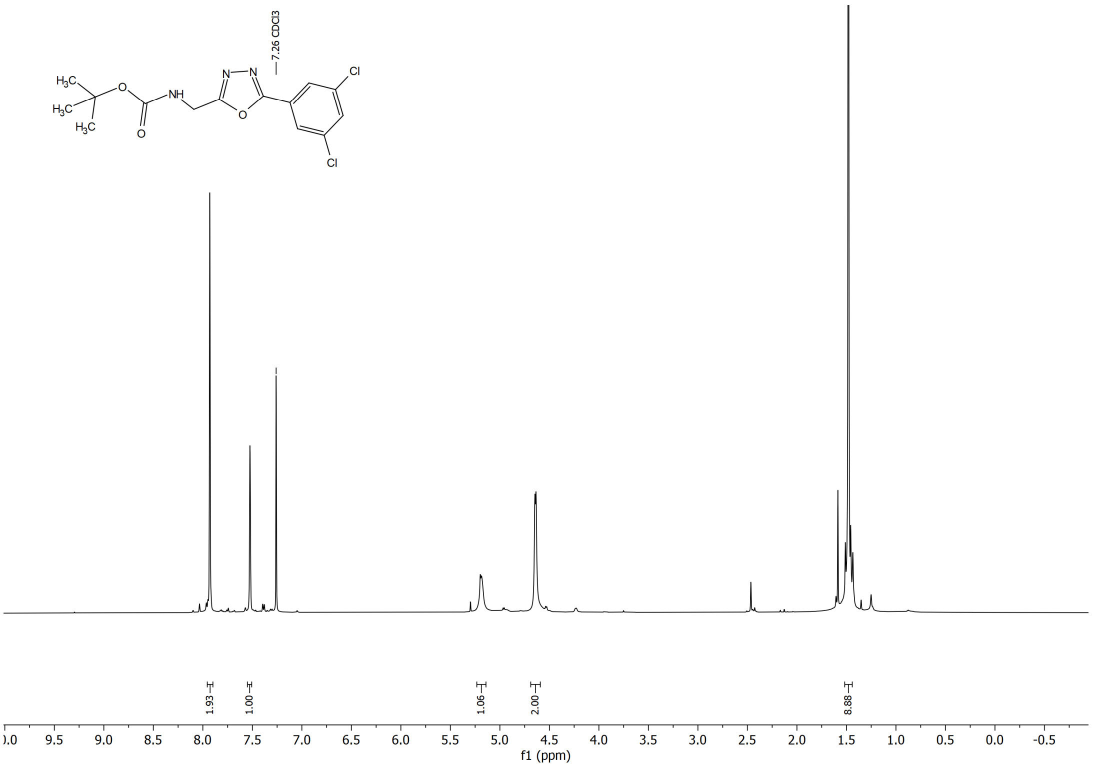

^{13}**C NMR** spectrum of **21b** (126 MHz, $CDCl_3$)

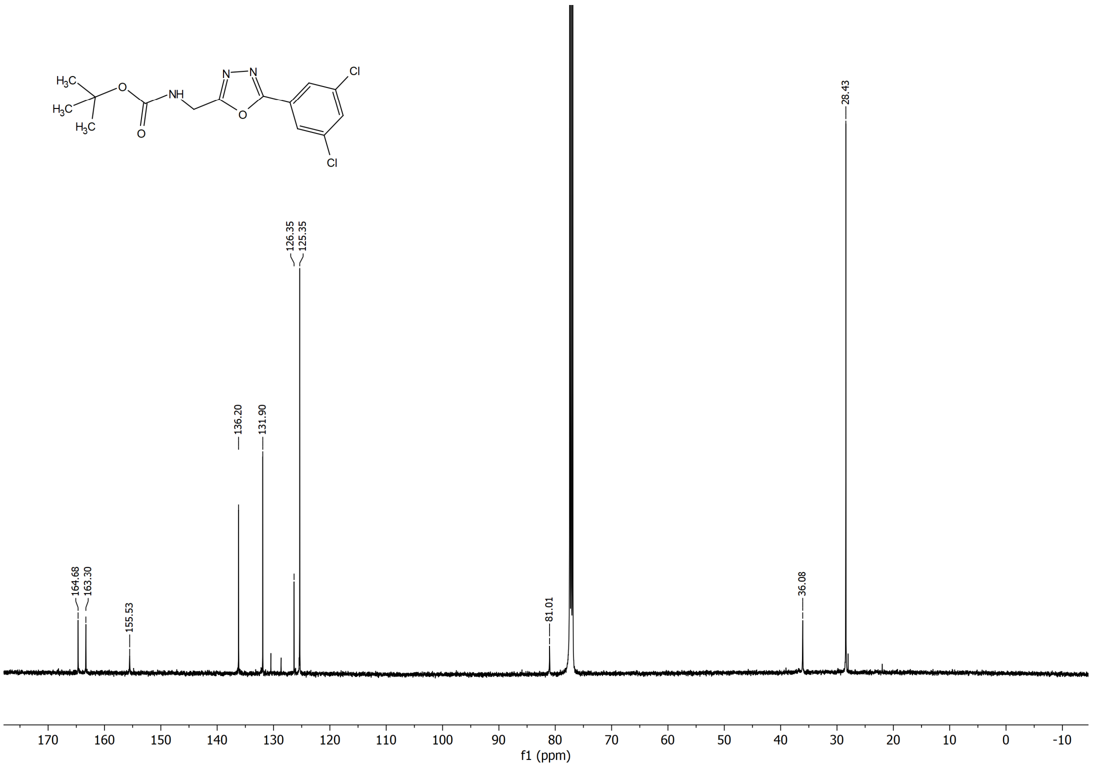

^{1}H NMR spectrum of **22** (500 MHz, $CDCl_3$)

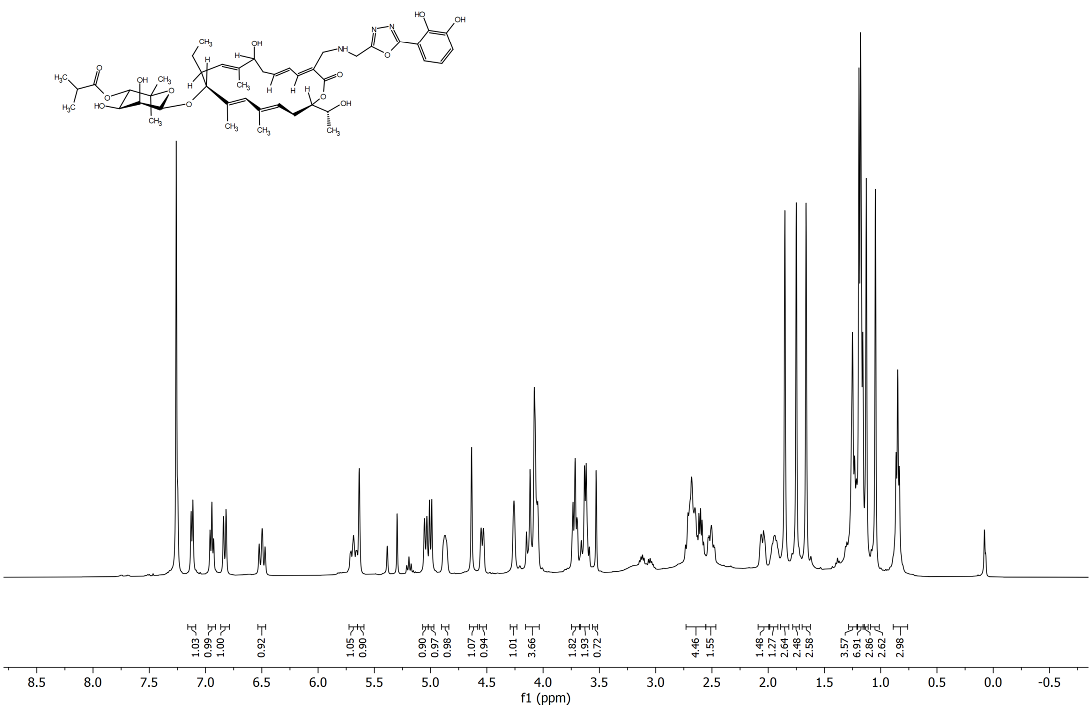

^{13}C NMR spectrum of **22** (126 MHz, $CDCl_3$)

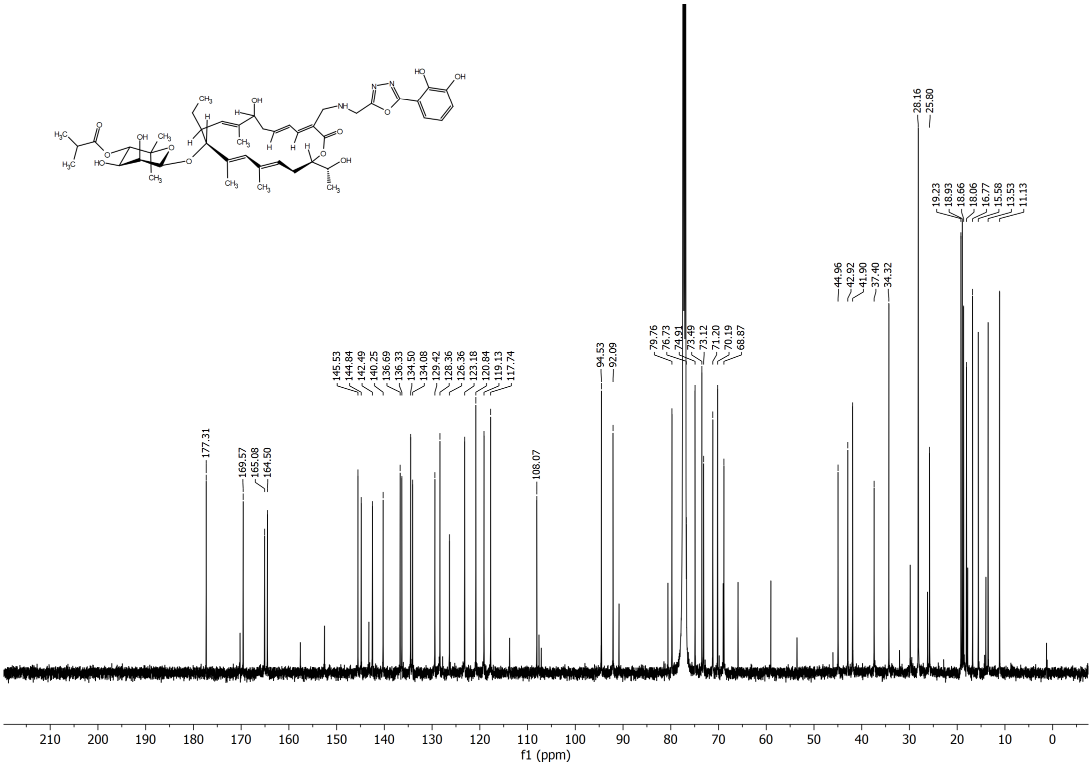

^{1}H NMR spectrum of **22a** (500 MHz, $CDCl_3$)

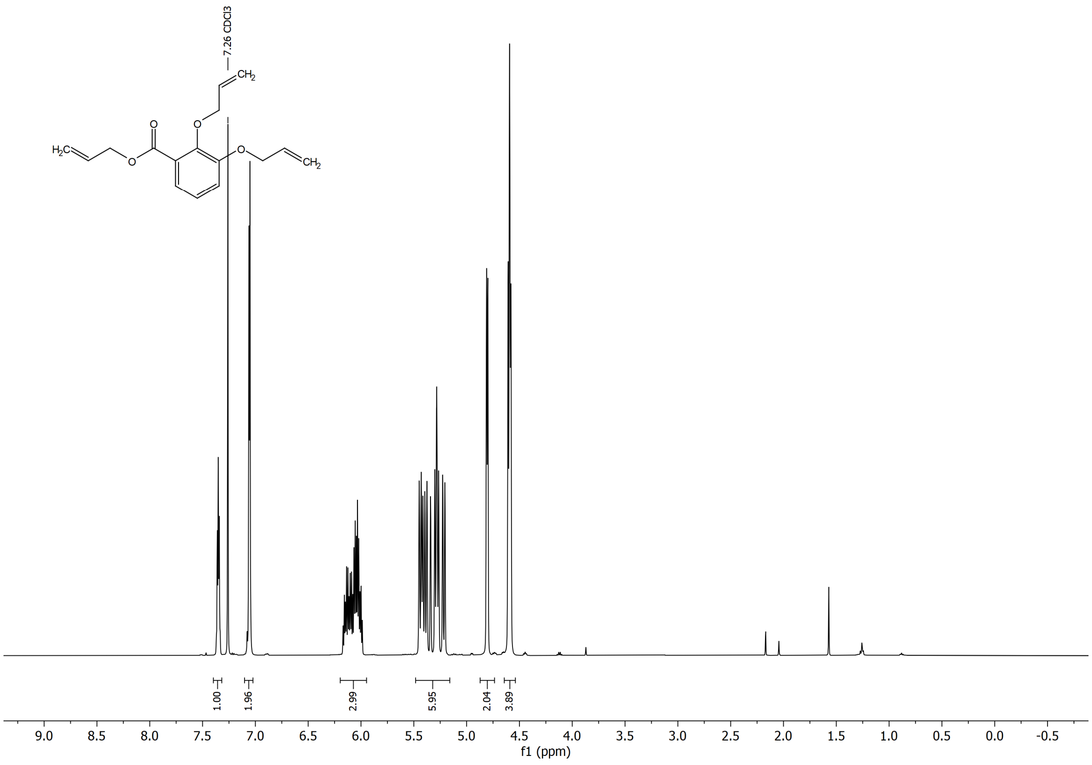

^{13}C NMR spectrum of **22a** (126 MHz, $CDCl_3$)

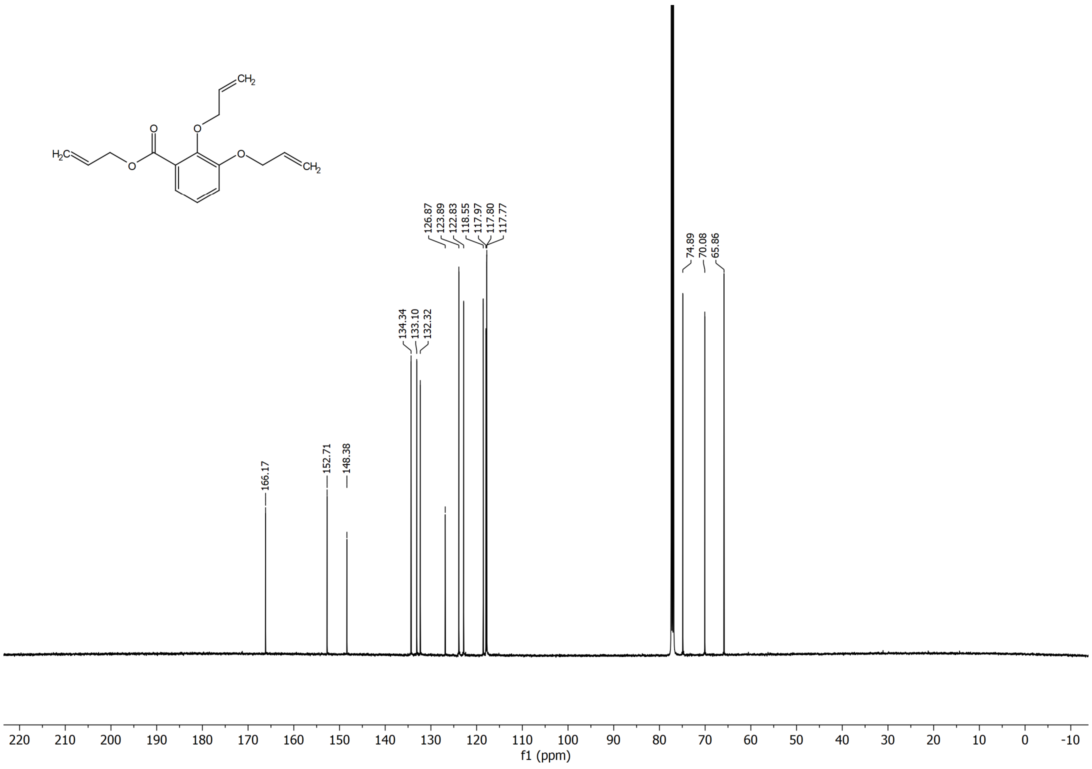

^{1}H NMR spectrum of 22b (400 MHz, $CDCl_3$)

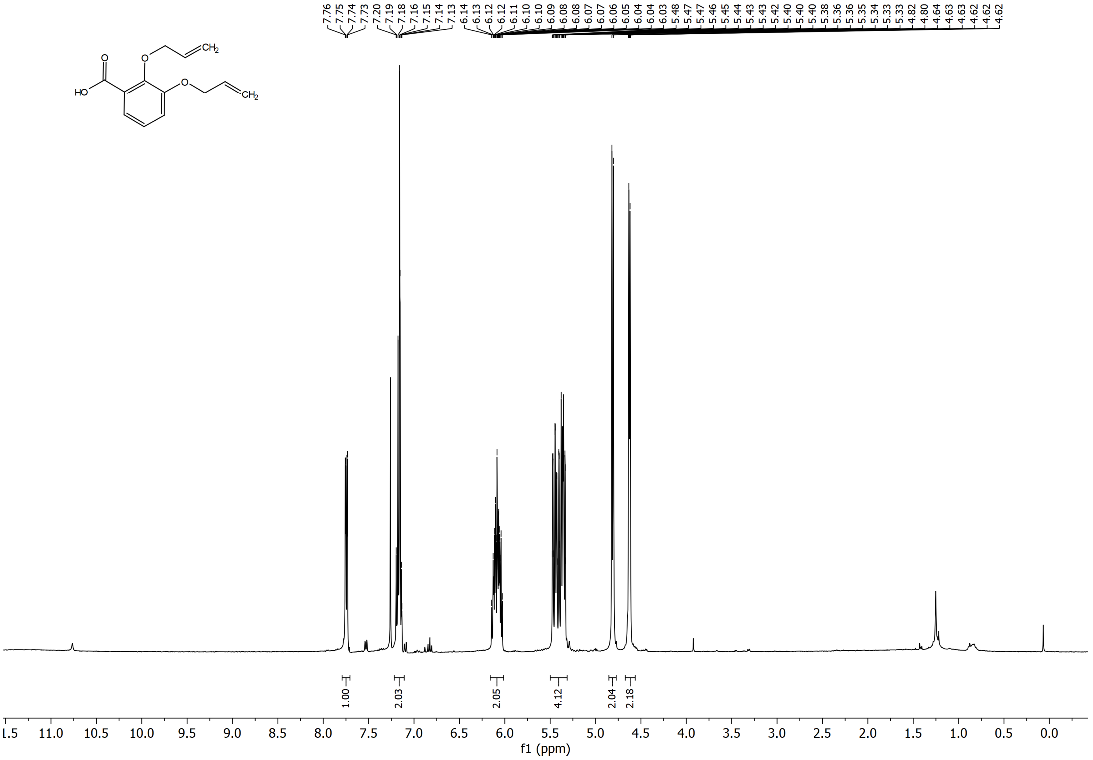

^{13}C NMR spectrum of 22b (101 MHz, $CDCl_3$)

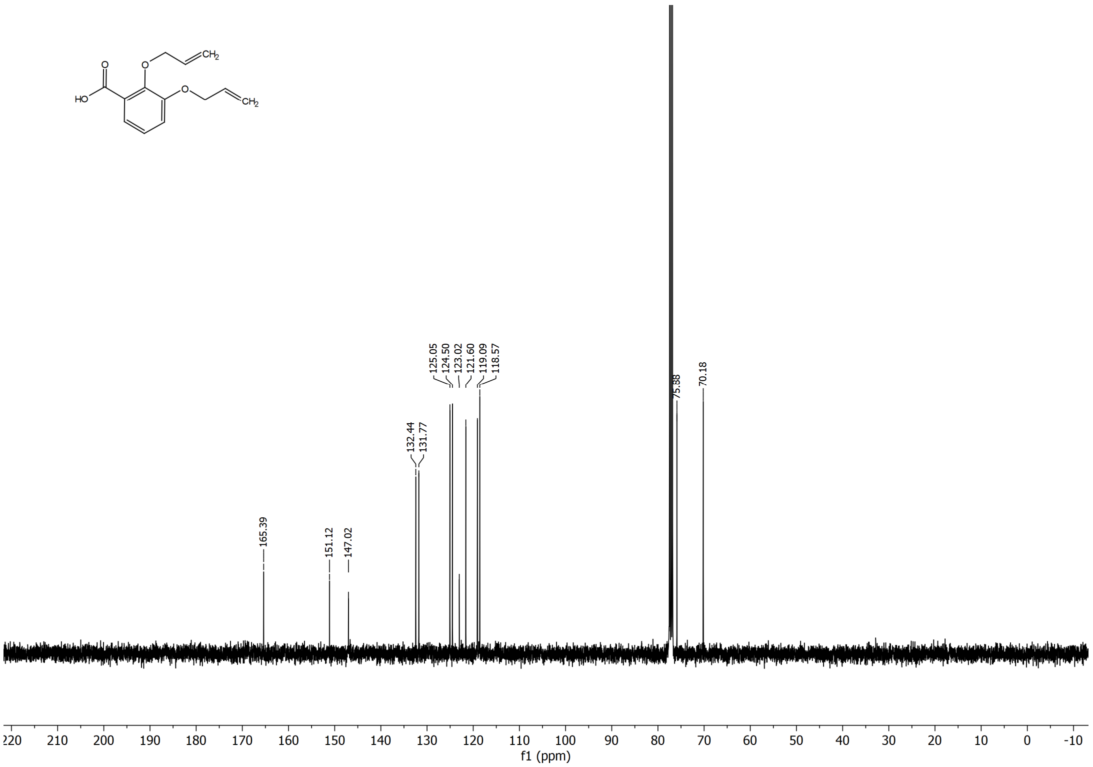

^{1}H NMR spectrum of **22c** (400 MHz, $CDCl_3$)

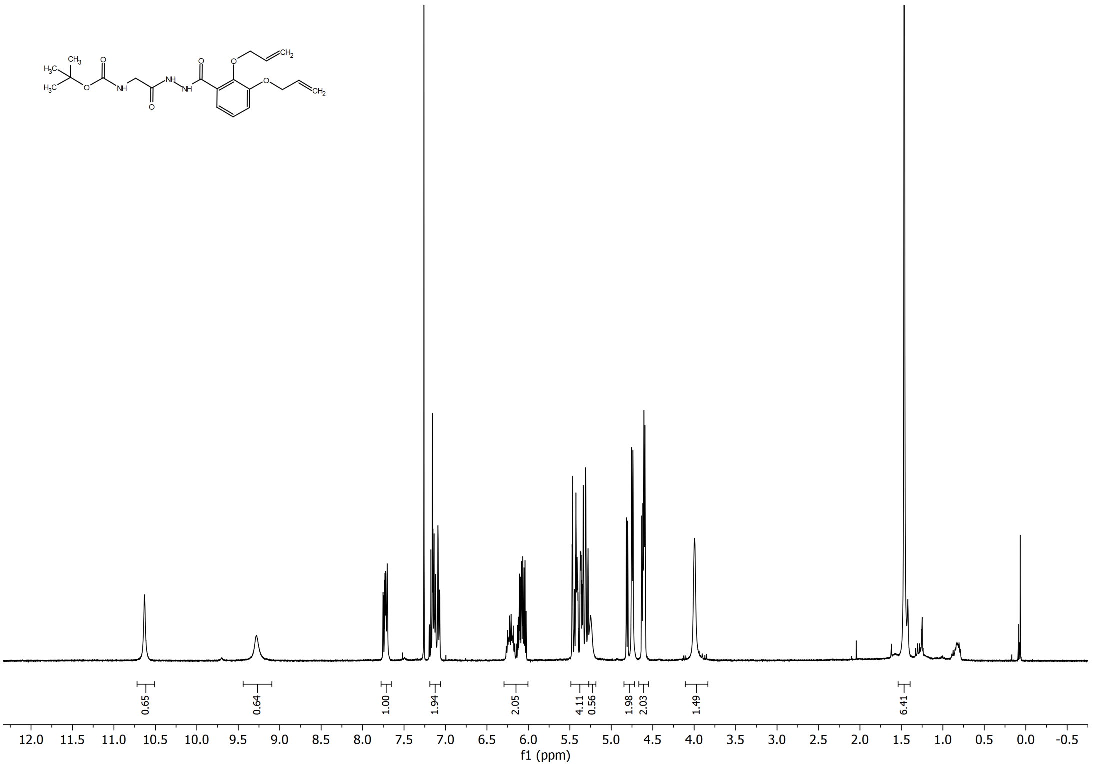

^{13}C NMR spectrum of **22c** (101 MHz, $CDCl_3$)

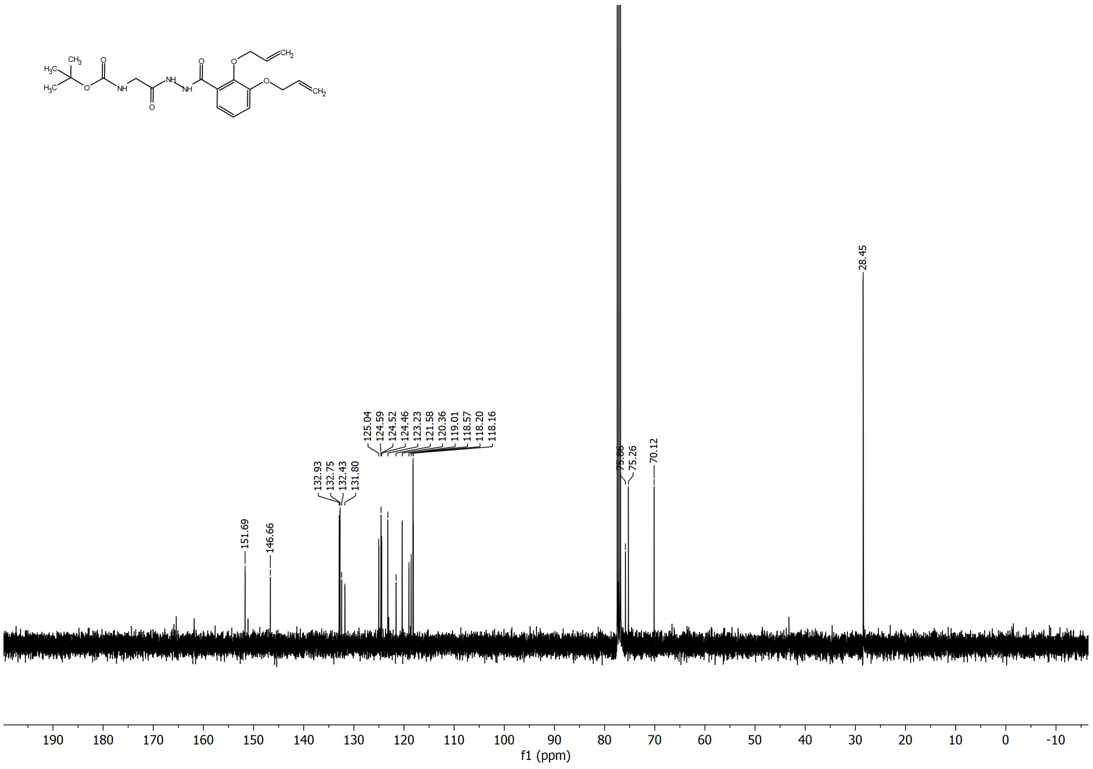

^{1}H NMR spectrum of **22d** (500 MHz, $CDCl_3$)

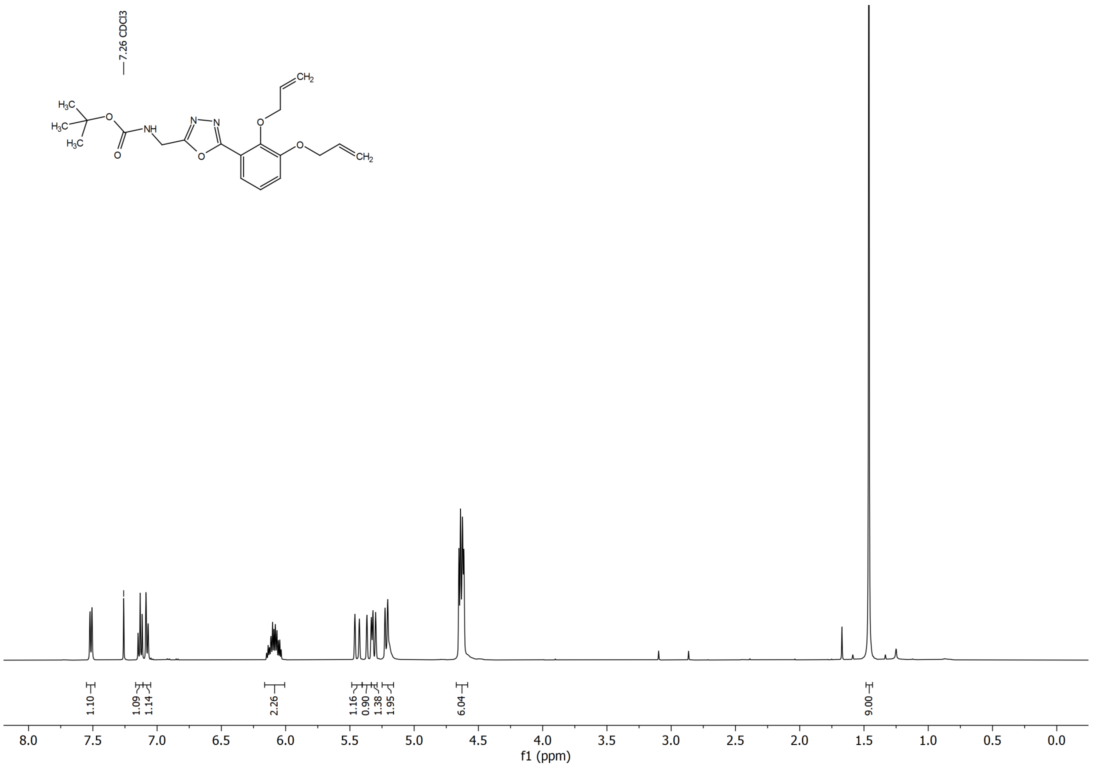

^{13}C NMR spectrum of **22d** (126 MHz, $CDCl_3$)

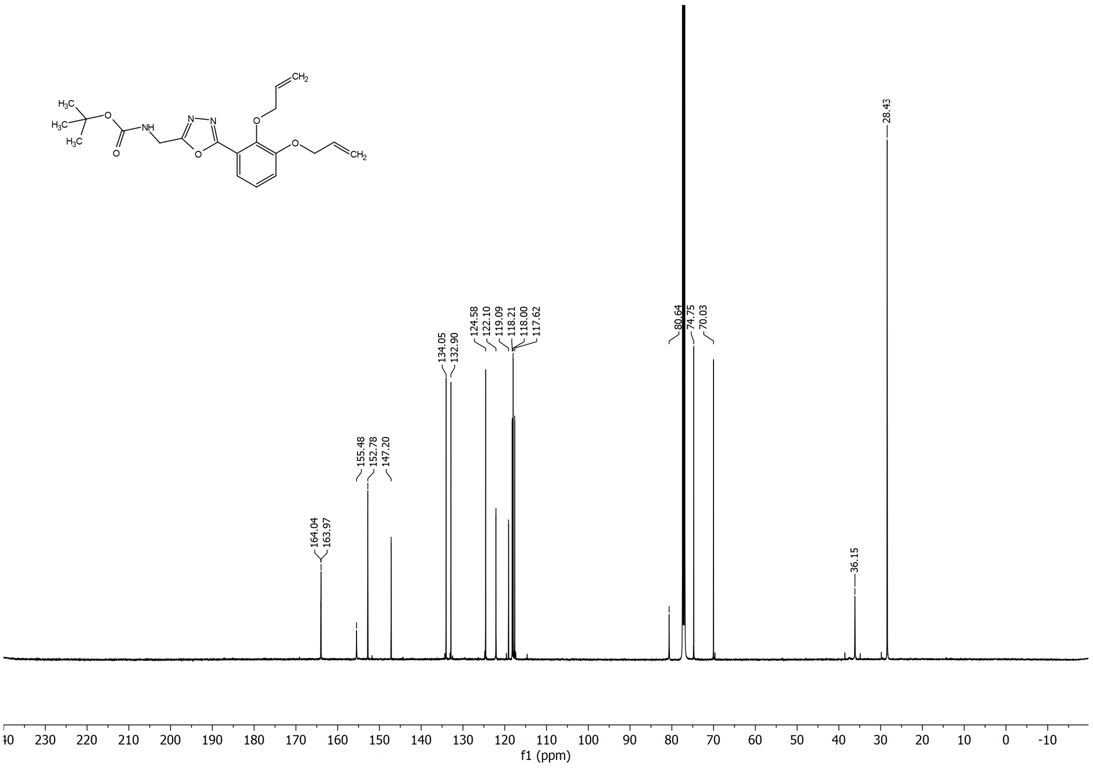

^{1}H NMR spectrum of **23** (500 MHz, $CDCl_3$)

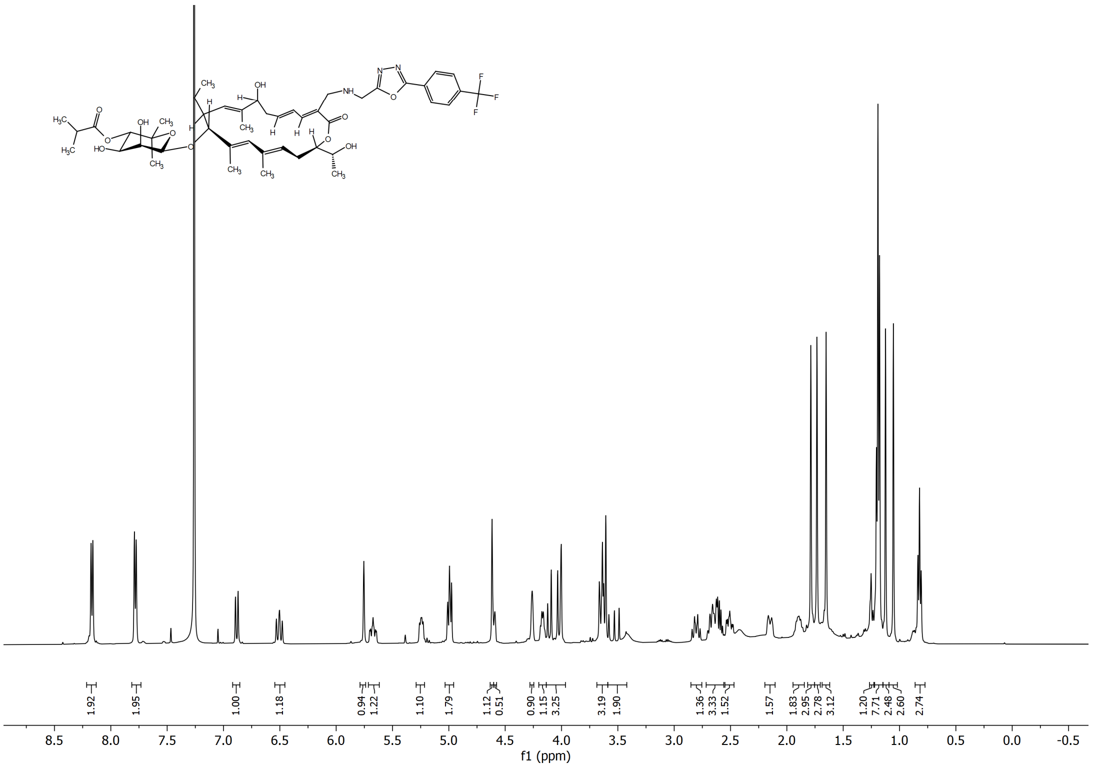

^{13}C NMR spectrum of **23** (126 MHz, $CDCl_3$)

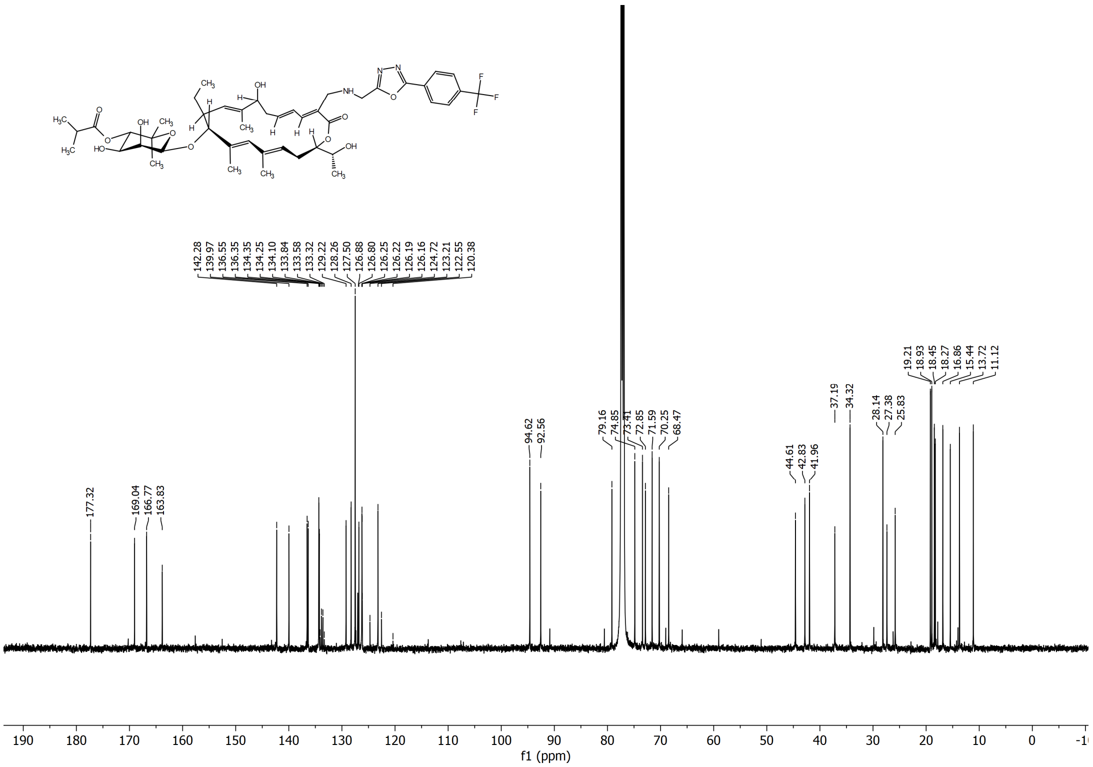

^{19}F NMR spectrum of **23** (471 MHz, $CDCl_3$)

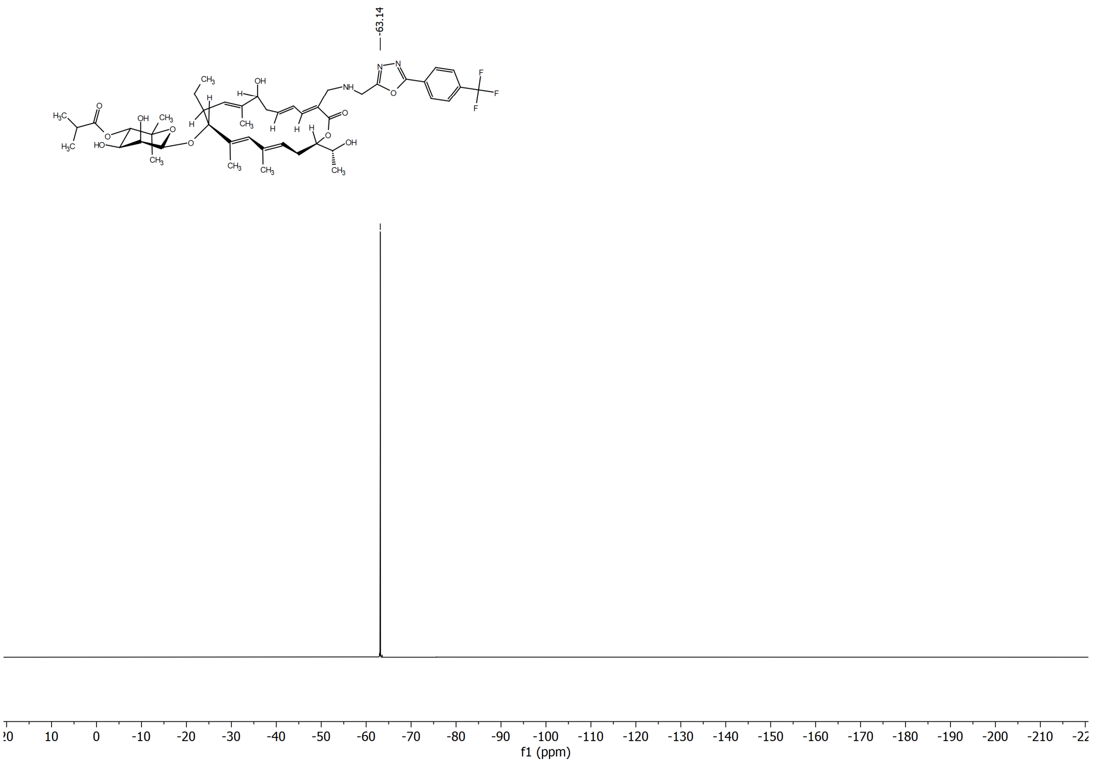

^{1}H NMR spectrum of **23a** (500 MHz, methanol-d_4)

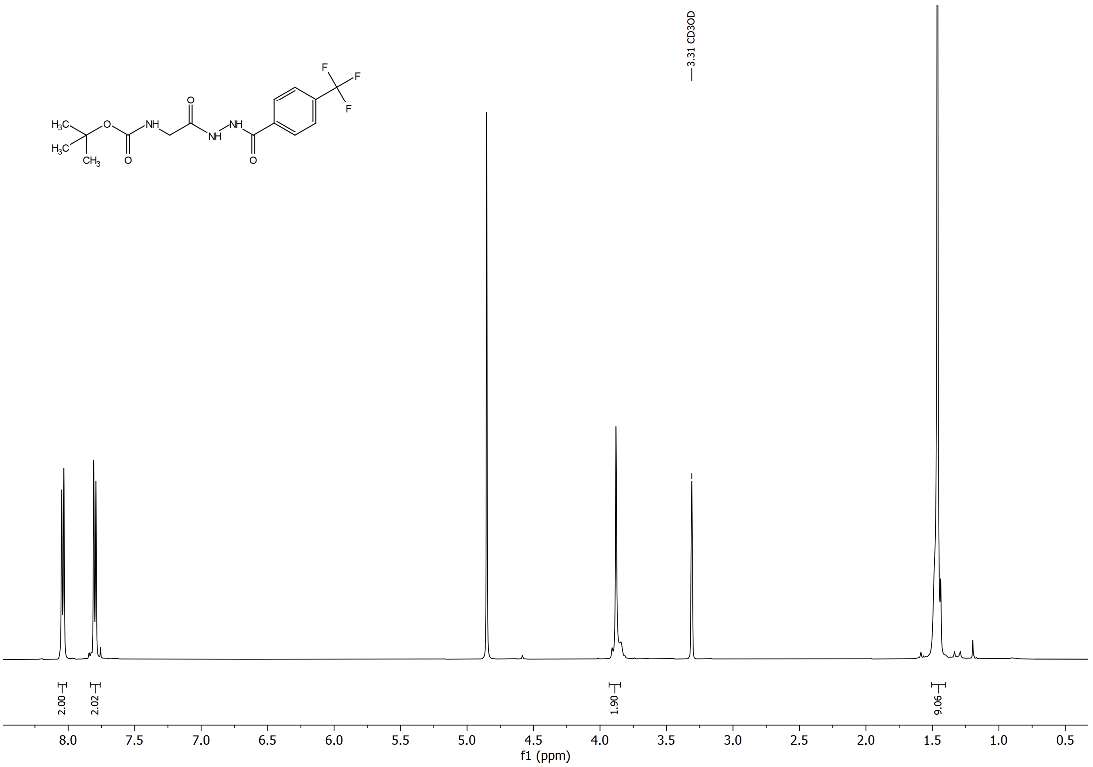

^{13}C NMR spectrum of **23a** (126 MHz, methanol-d_4)

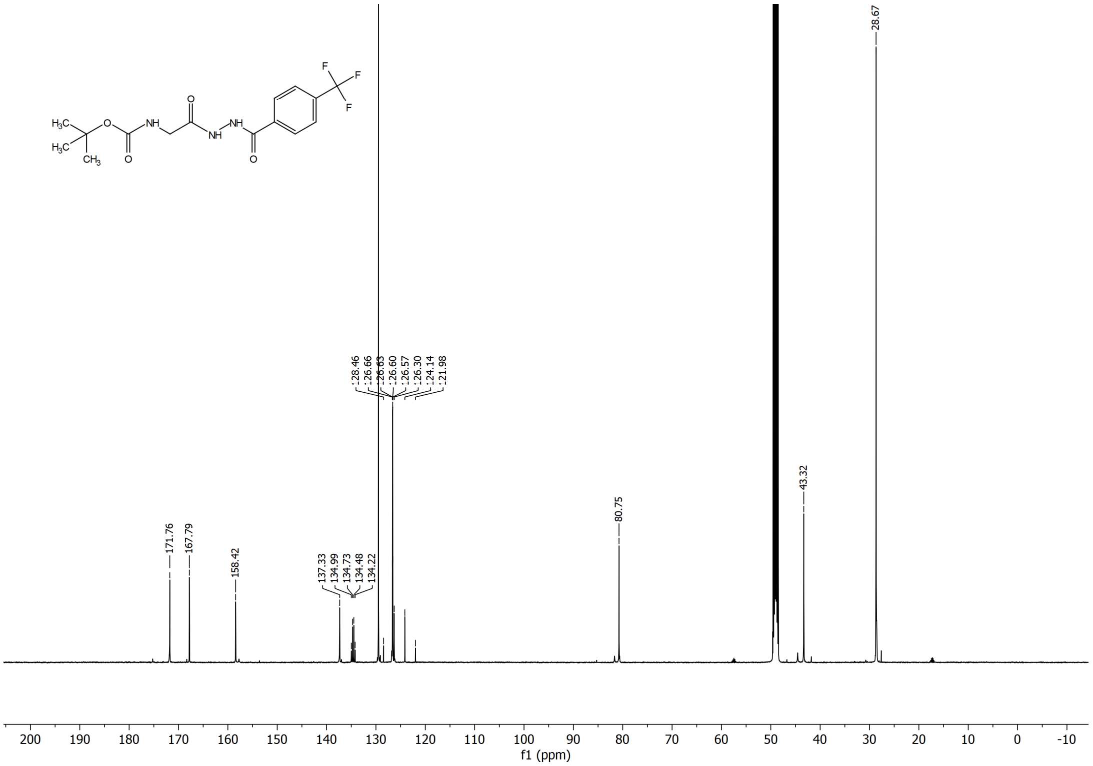

^{19}F NMR spectrum of **23a** (471 MHz, methanol-d_4)

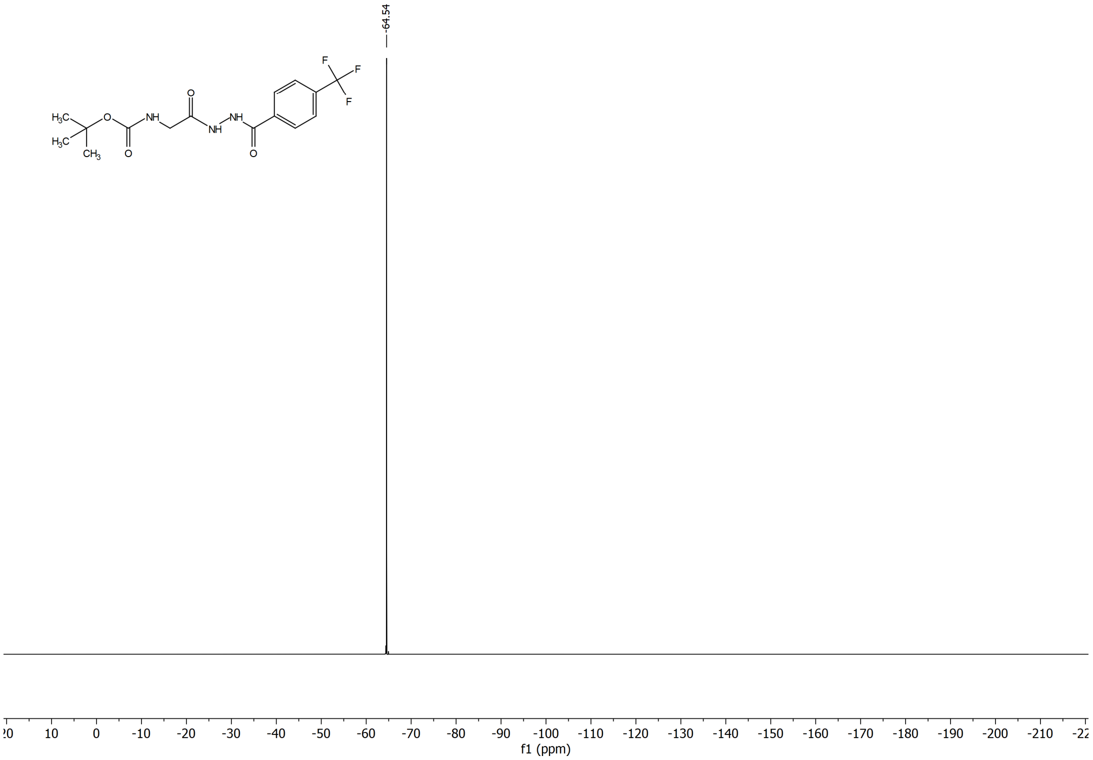

^{1}H NMR spectrum of **23b** (500 MHz, methanol-d_4)

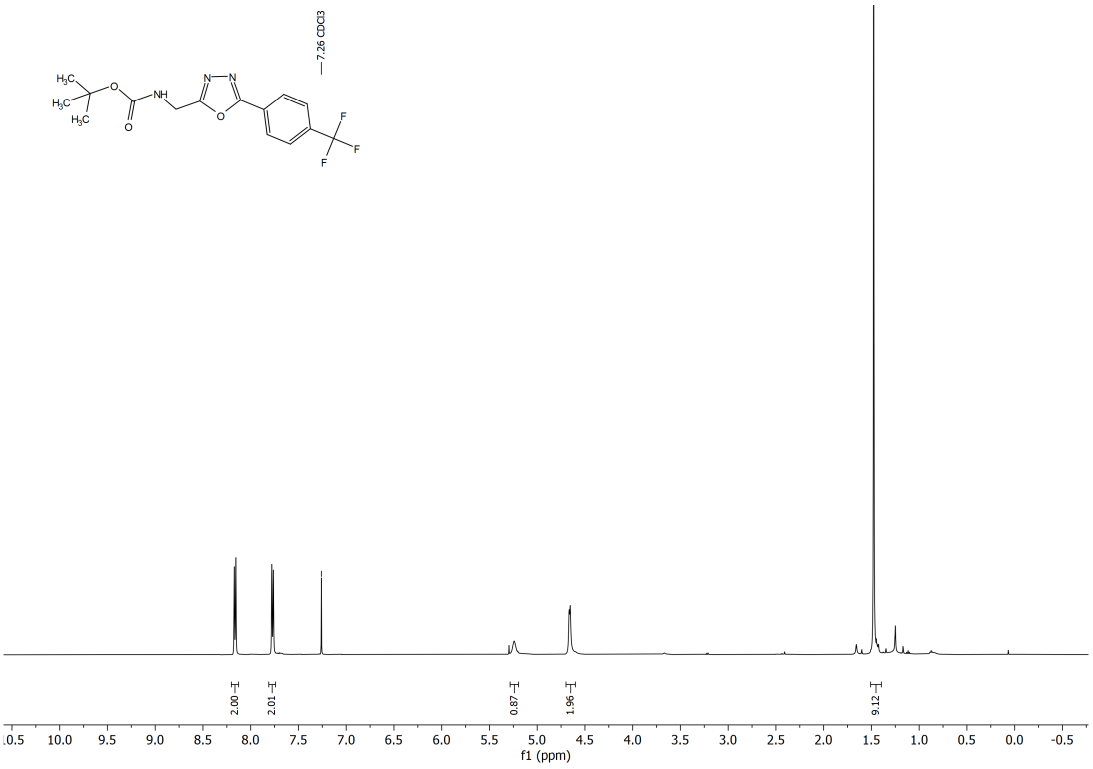

^{13}C NMR spectrum of **23b** (126 MHz, methanol-d_4)

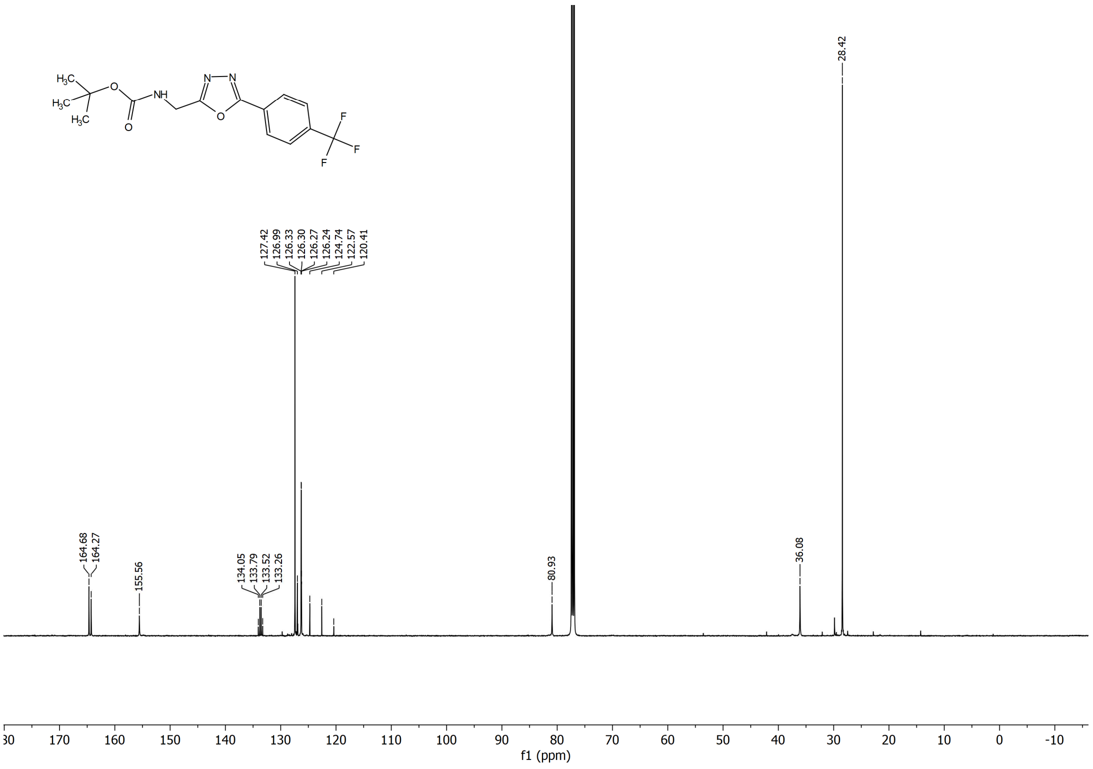

^{19}F NMR spectrum of **23b** (471 MHz, methanol-d_4)

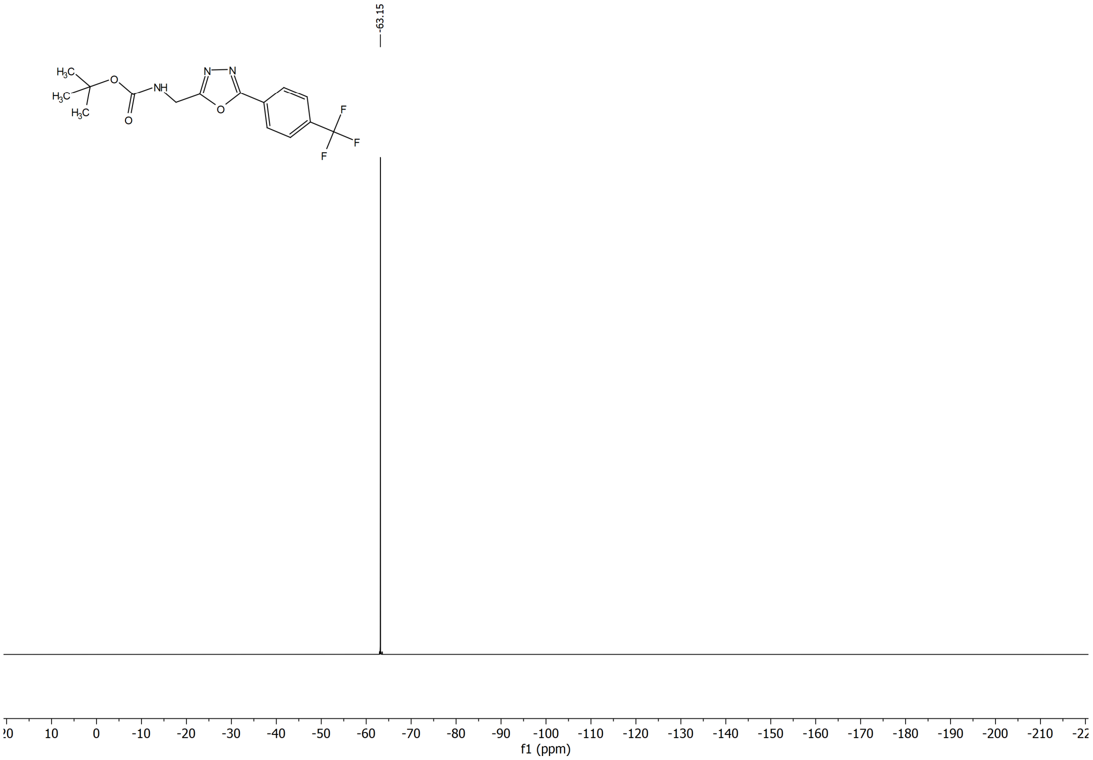

^{1}H NMR spectrum of **24** (500 MHz, acetone-d_6)

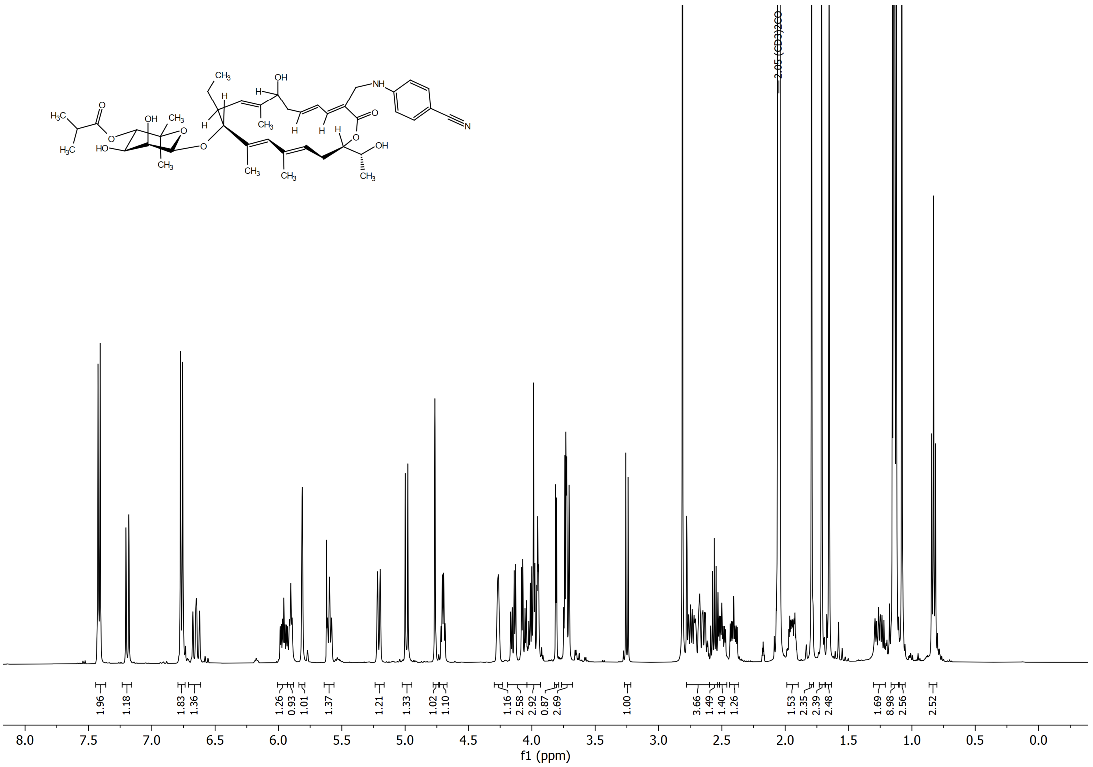

^{13}C NMR spectrum of **24** (126 MHz, acetone-d_6)

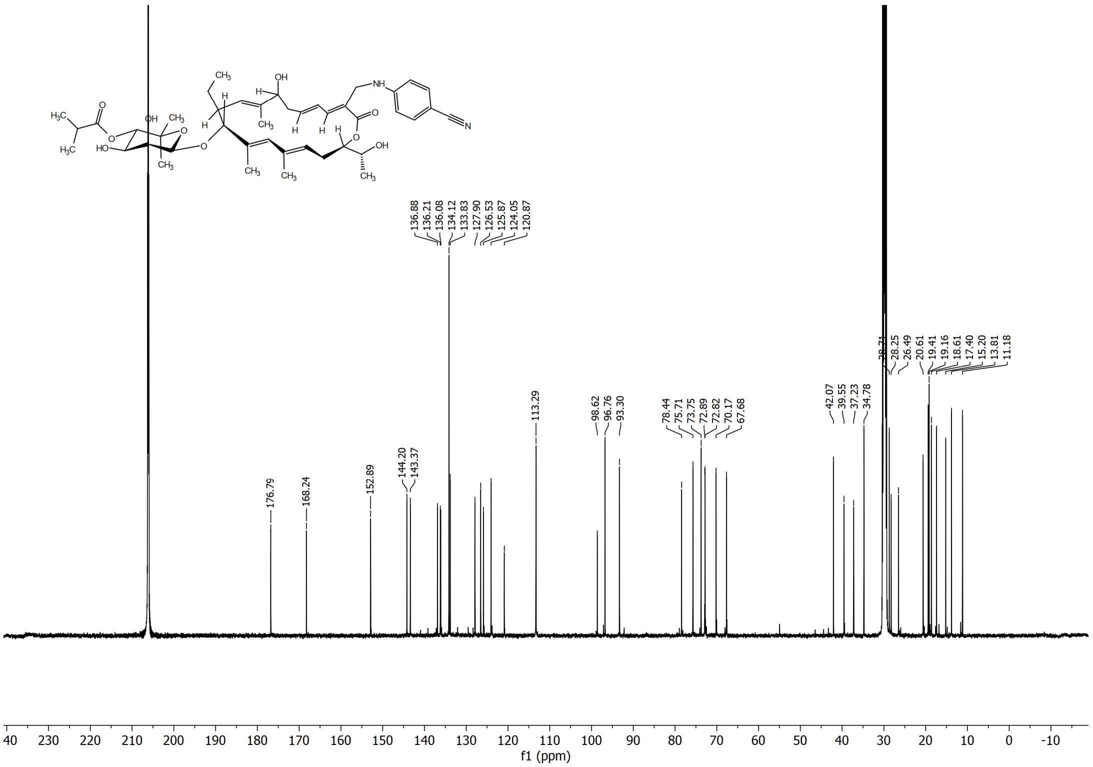

^{1}H NMR spectrum of **25** (500 MHz, acetone-d_6)

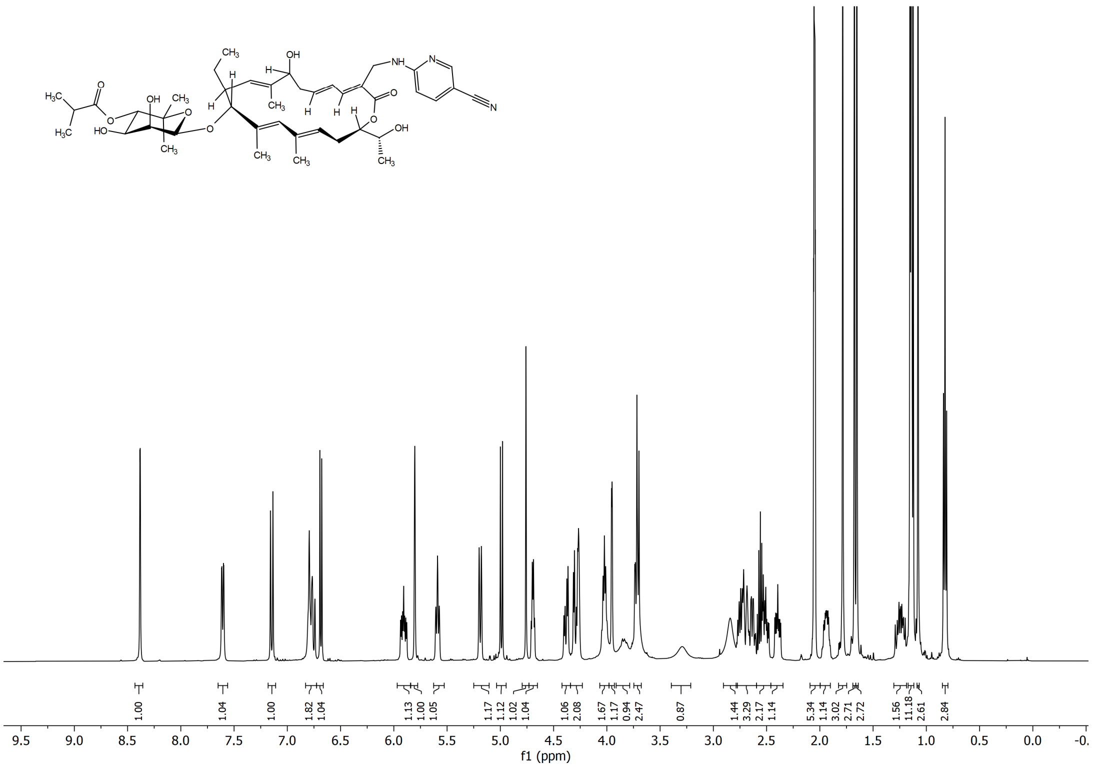

^{13}C NMR spectrum of **25** (126 MHz, acetone-d_6)

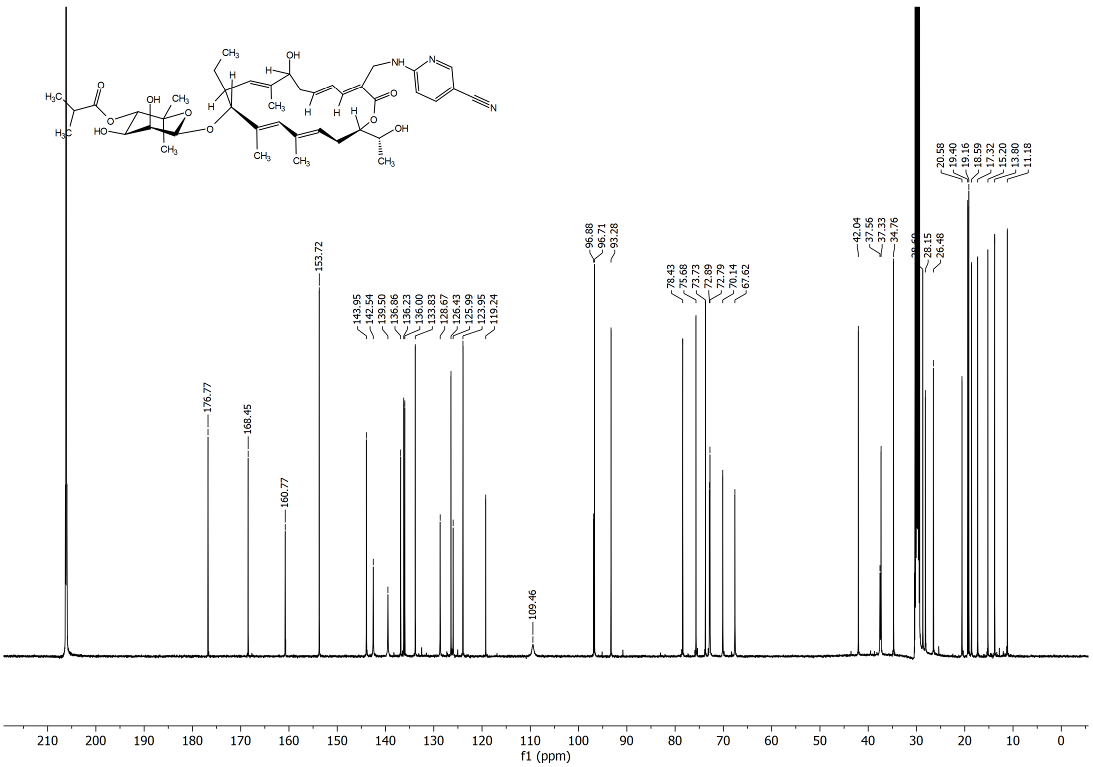

^{1}H NMR spectrum of **27** (500 MHz, $CDCl_3$)

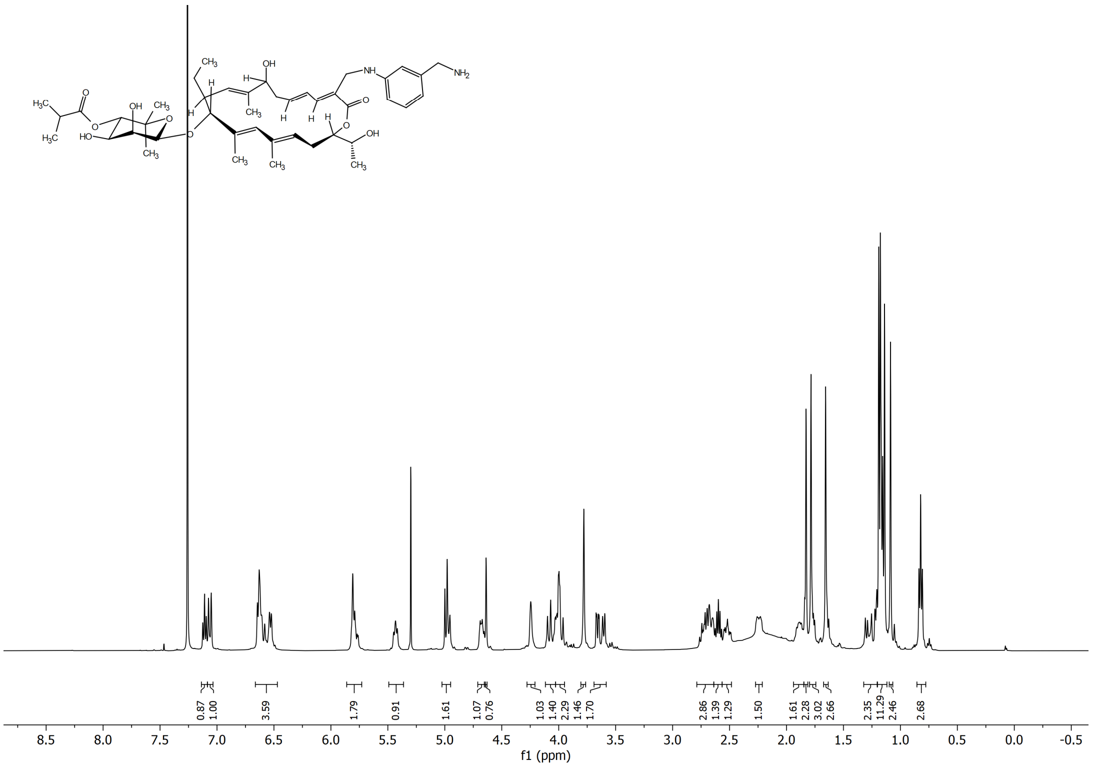

^{13}C NMR spectrum of **27** (126 MHz, $CDCl_3$)

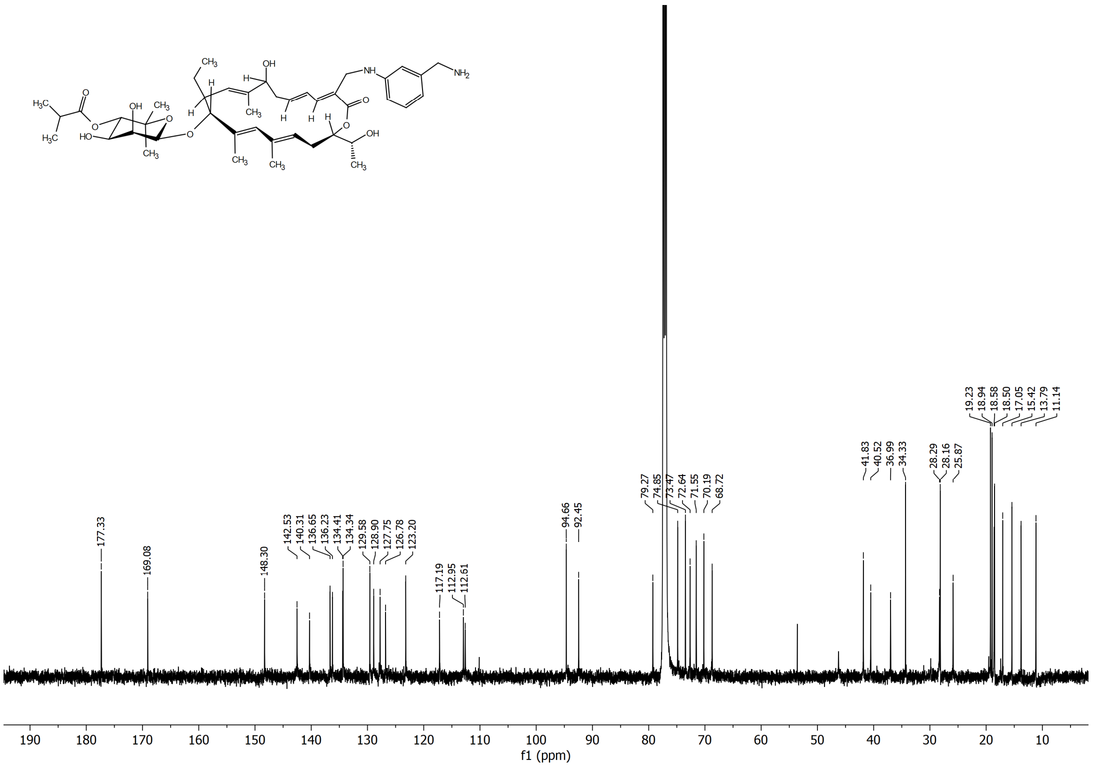

COSY spectrum of **27** (500 MHz, $CDCl_3$)

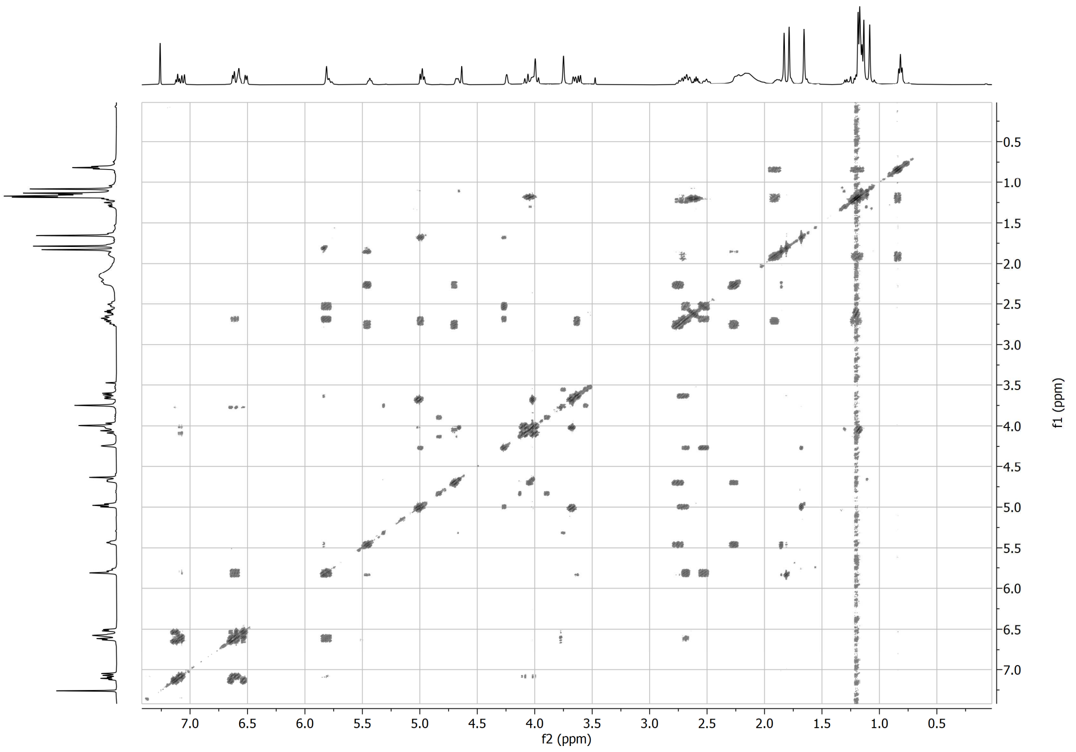

TOCSY spectrum of **27** (500 MHz, $CDCl_3$)

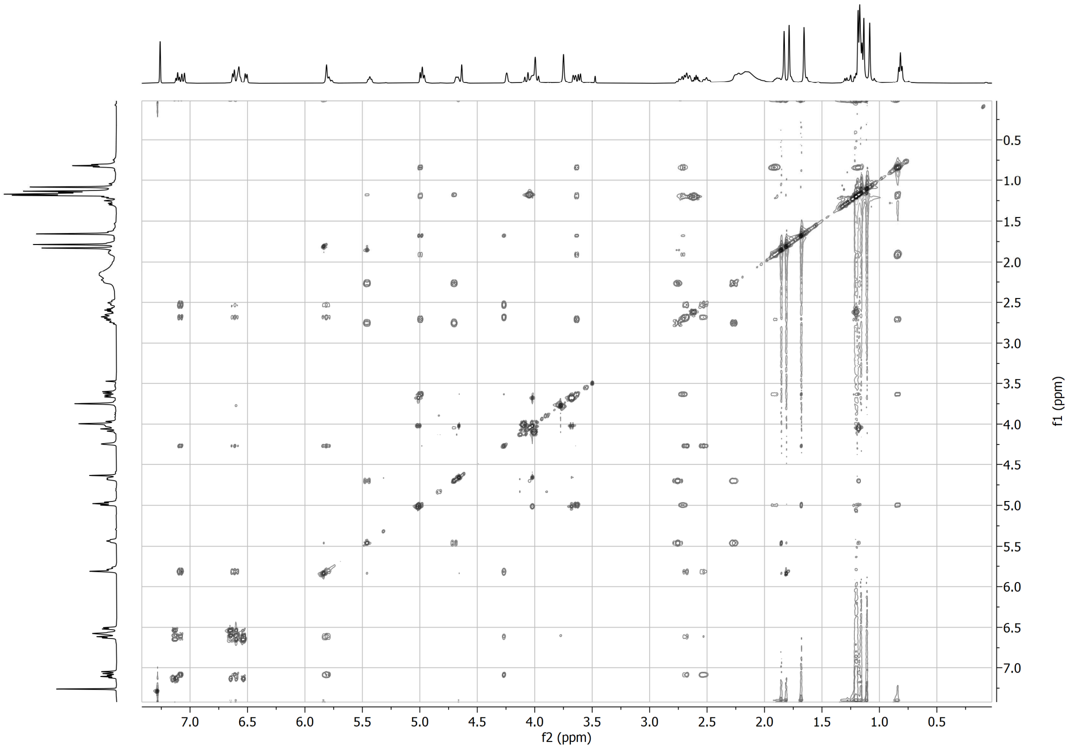

HSQC_edited spectrum of **27** (500 MHz 126 MHz, $CDCl_3$)

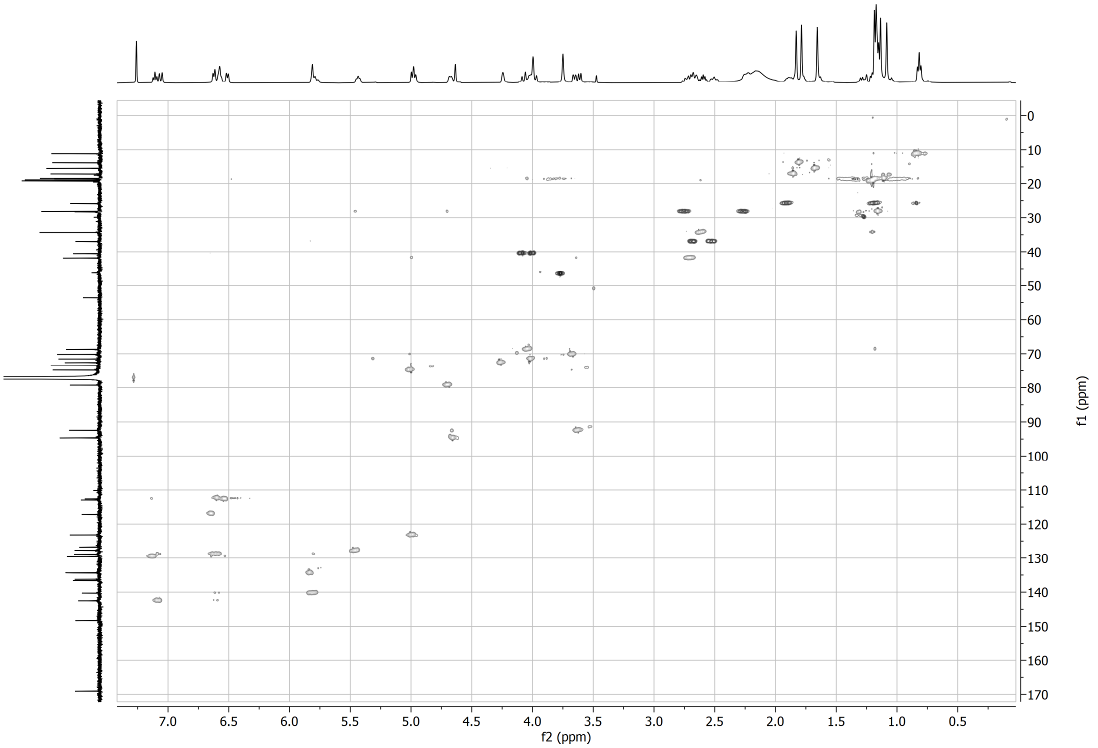

HMBC spectrum of **27** (500 MHz 126 MHz, $CDCl_3$)

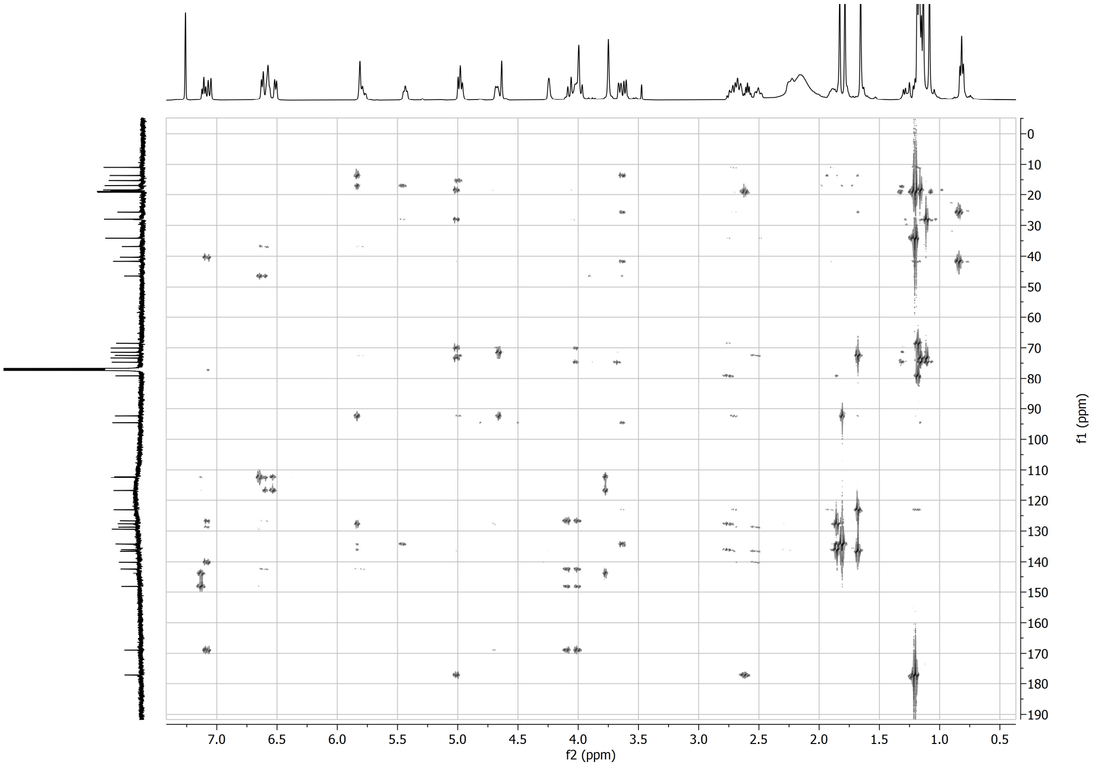

^{1}H NMR spectrum of **28** (500 MHz, $CDCl_3$)

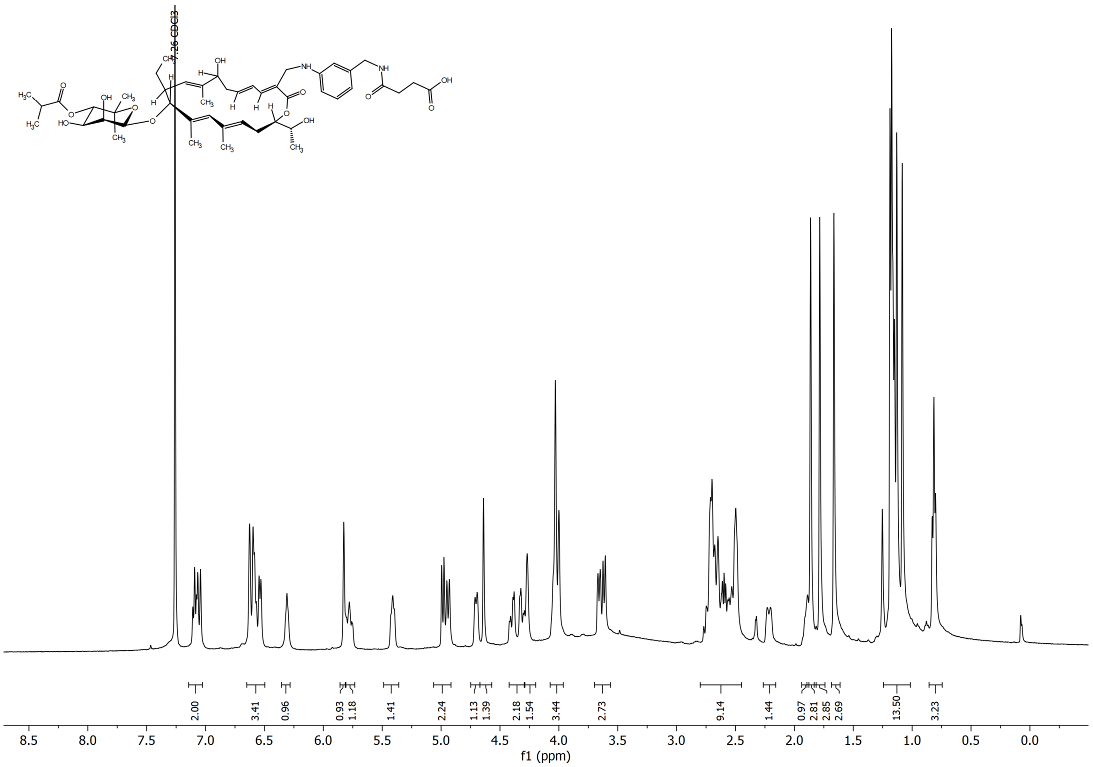

^{13}C NMR spectrum of **28** (126 MHz, $CDCl_3$)

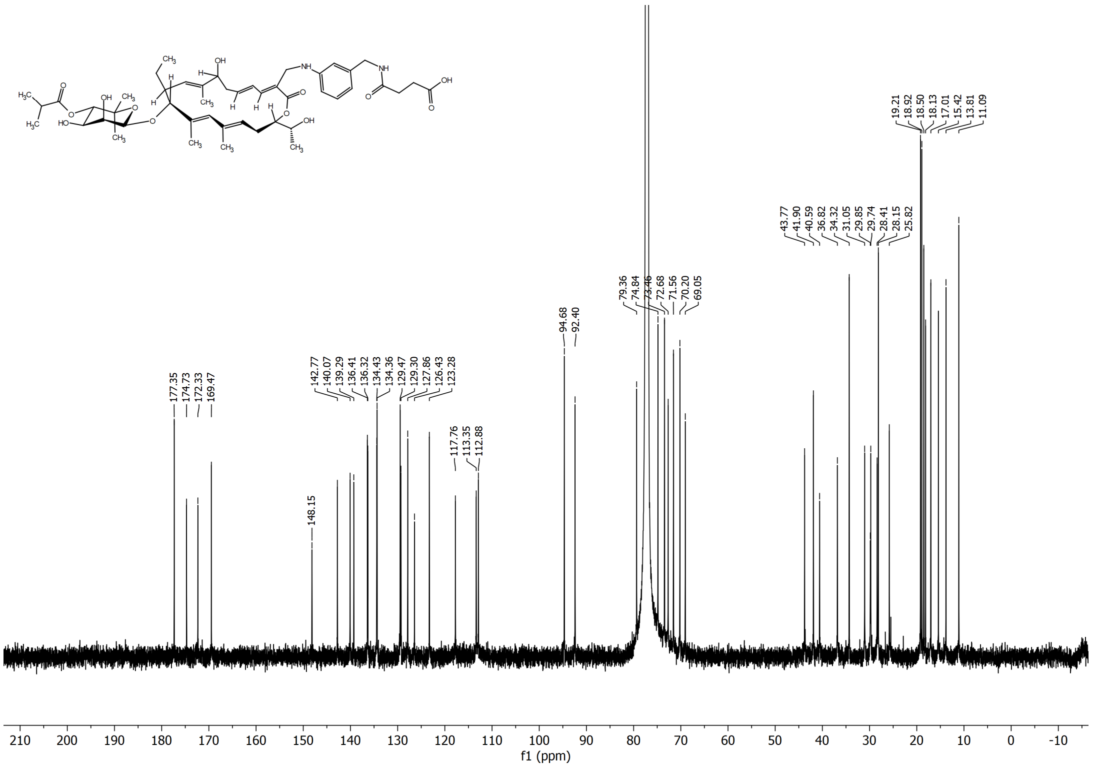

¹H NMR spectrum of **29** (500 MHz, acetone-*d*₆)

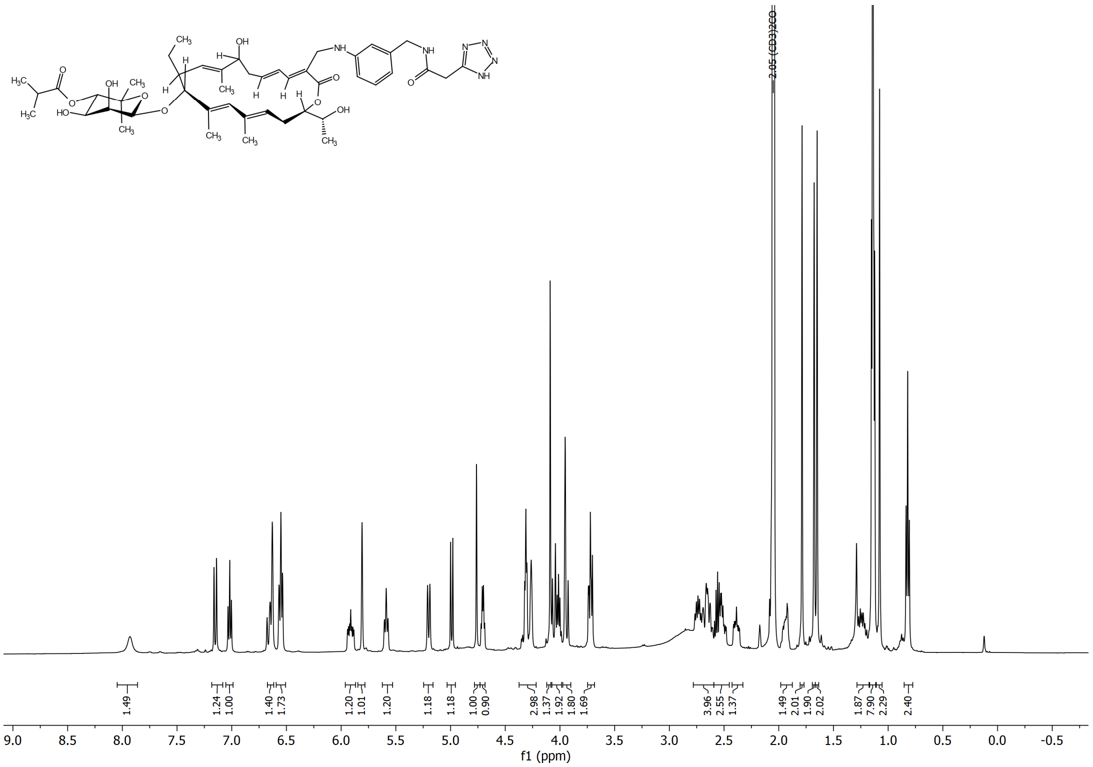

¹³C NMR spectrum of **29** (126 MHz, acetone-*d*₆)

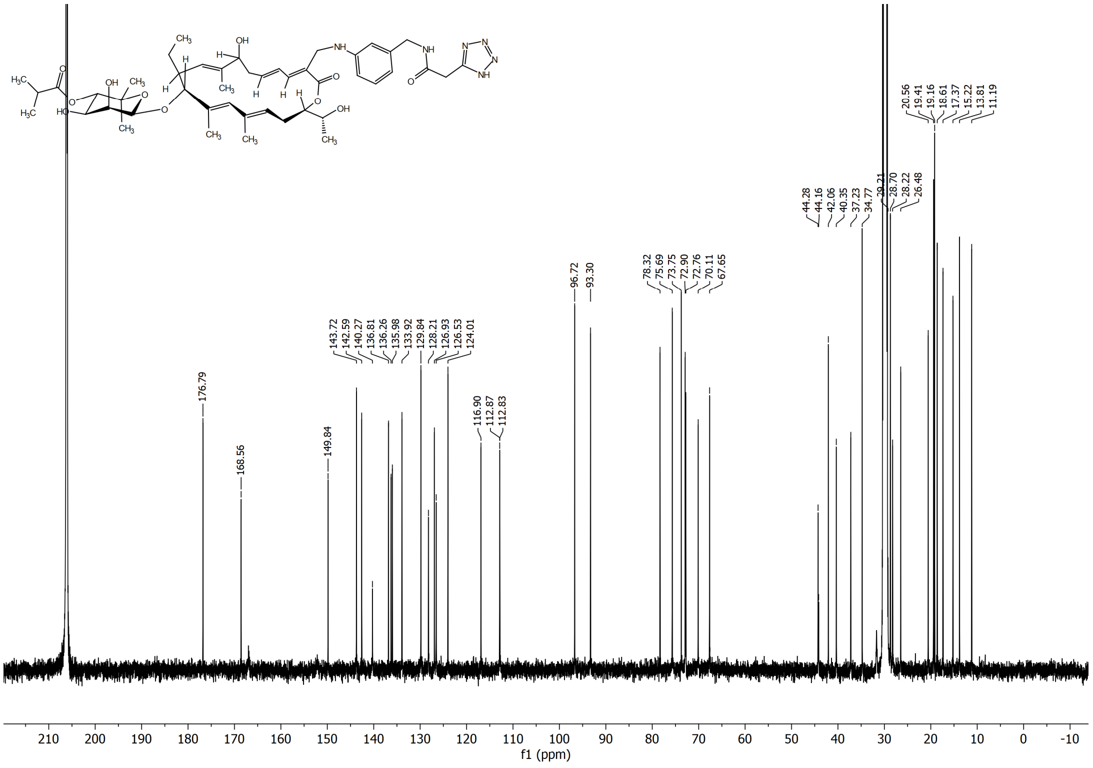

^{1}H NMR spectrum of **30** (500 MHz, $CDCl_3$)

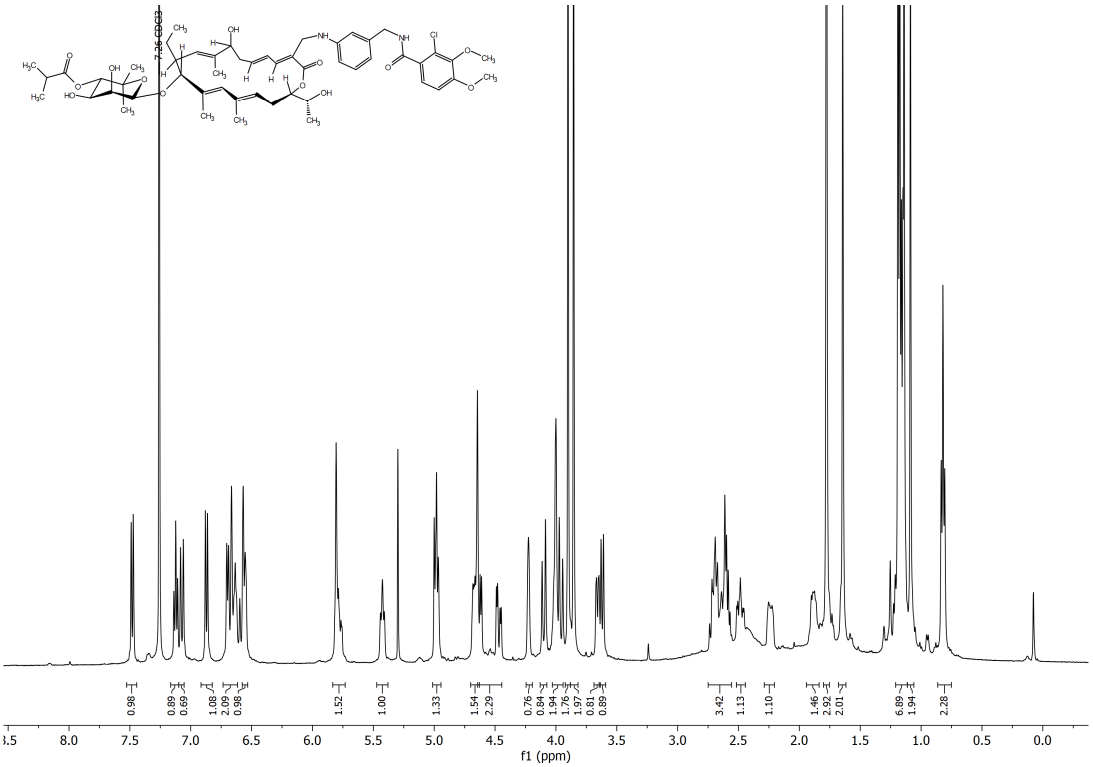

^{13}C NMR spectrum of **30** (126 MHz, $CDCl_3$)

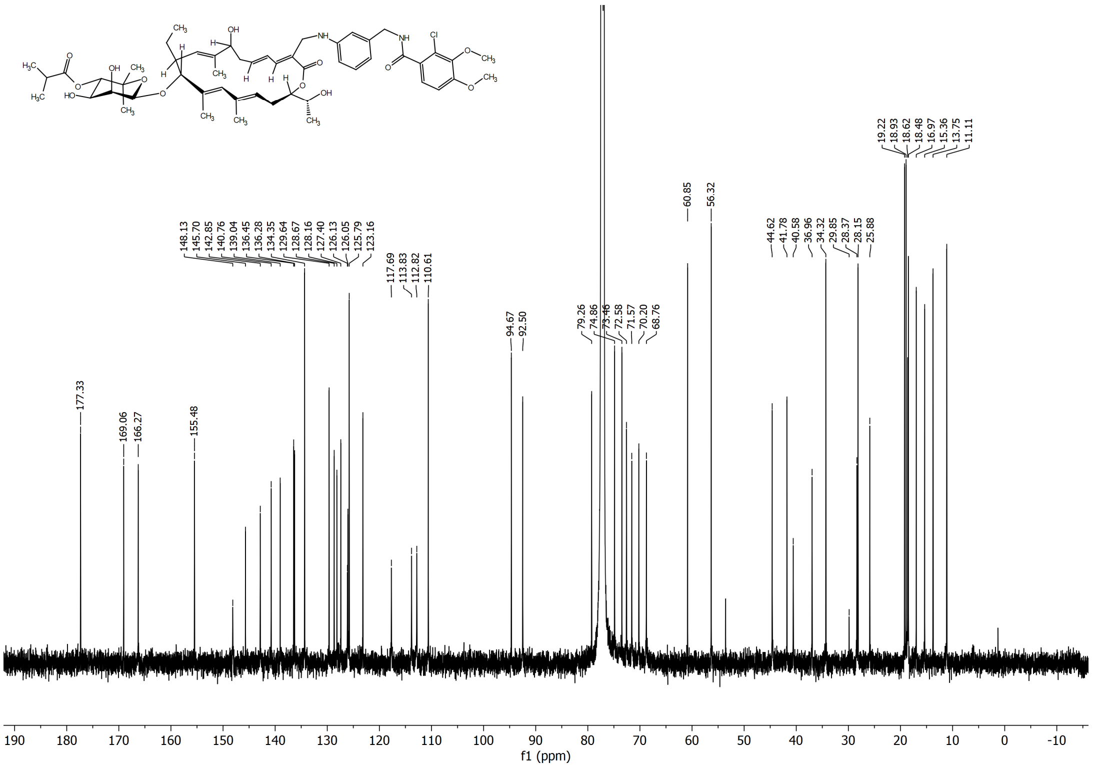

¹H NMR spectrum of **31** (500 MHz, acetone-d_6)

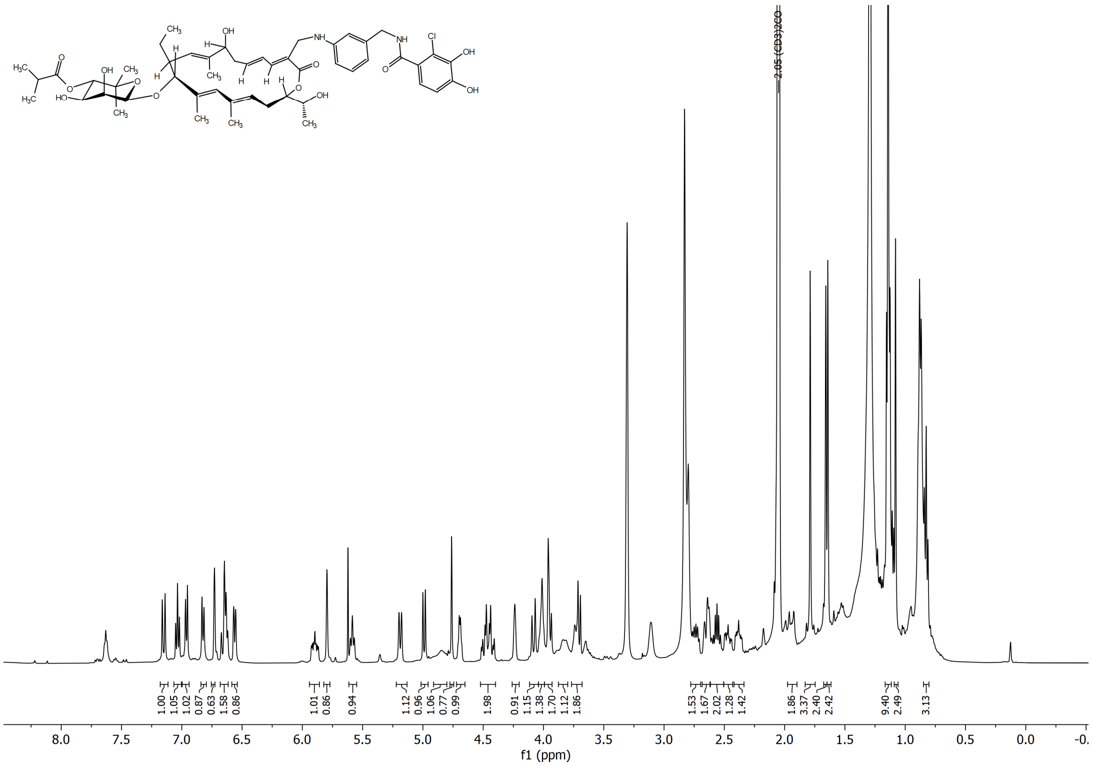

¹³C NMR spectrum of **31** (126 MHz, acetone-d_6)

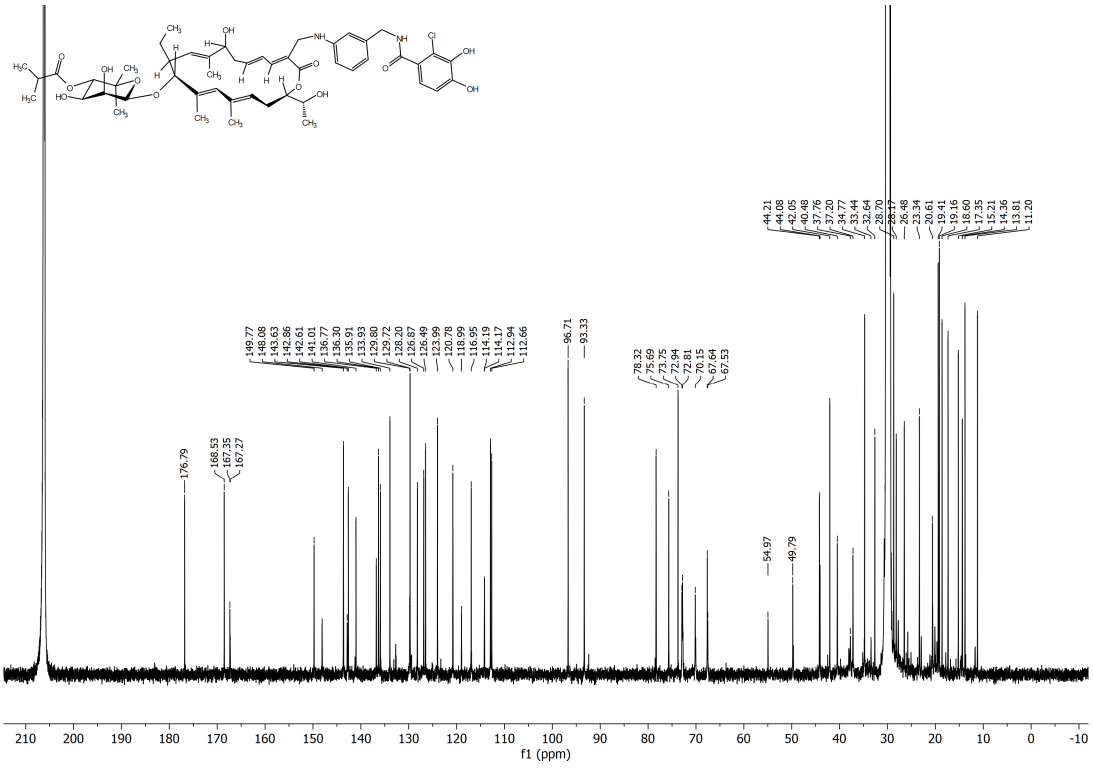

^{1}H NMR spectrum of **31a** (500 MHz, $CDCl_3$)

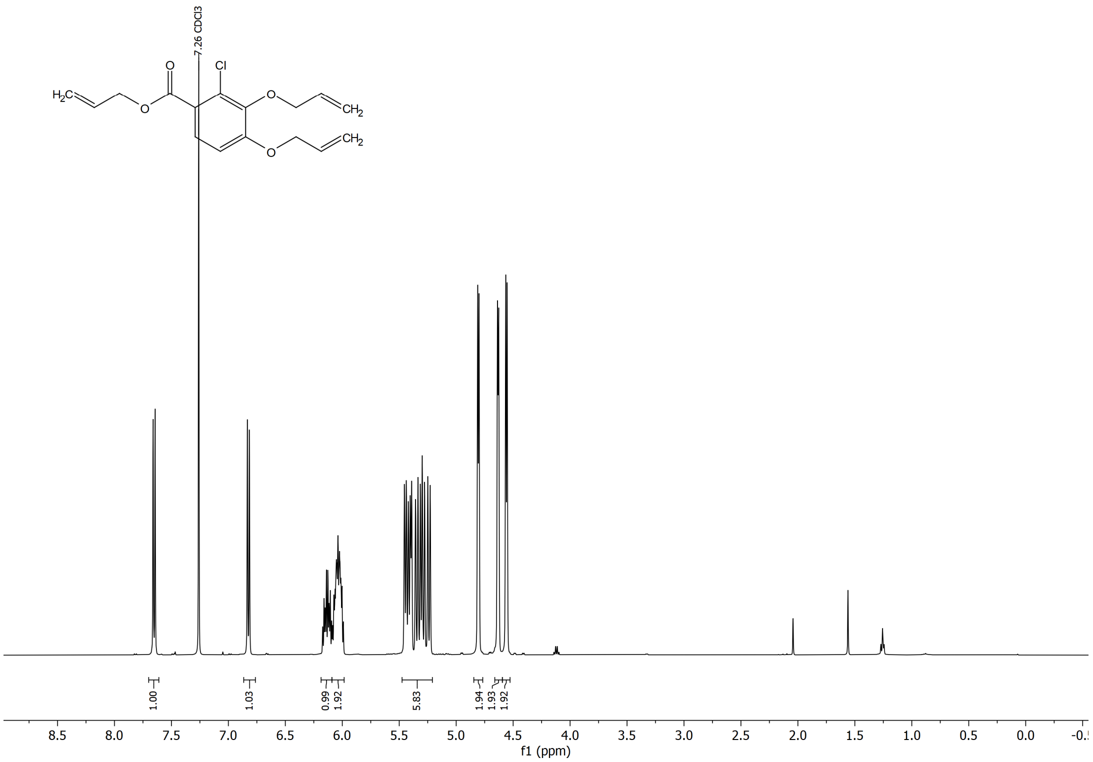

^{13}C NMR spectrum of **31a** (126 MHz, $CDCl_3$)

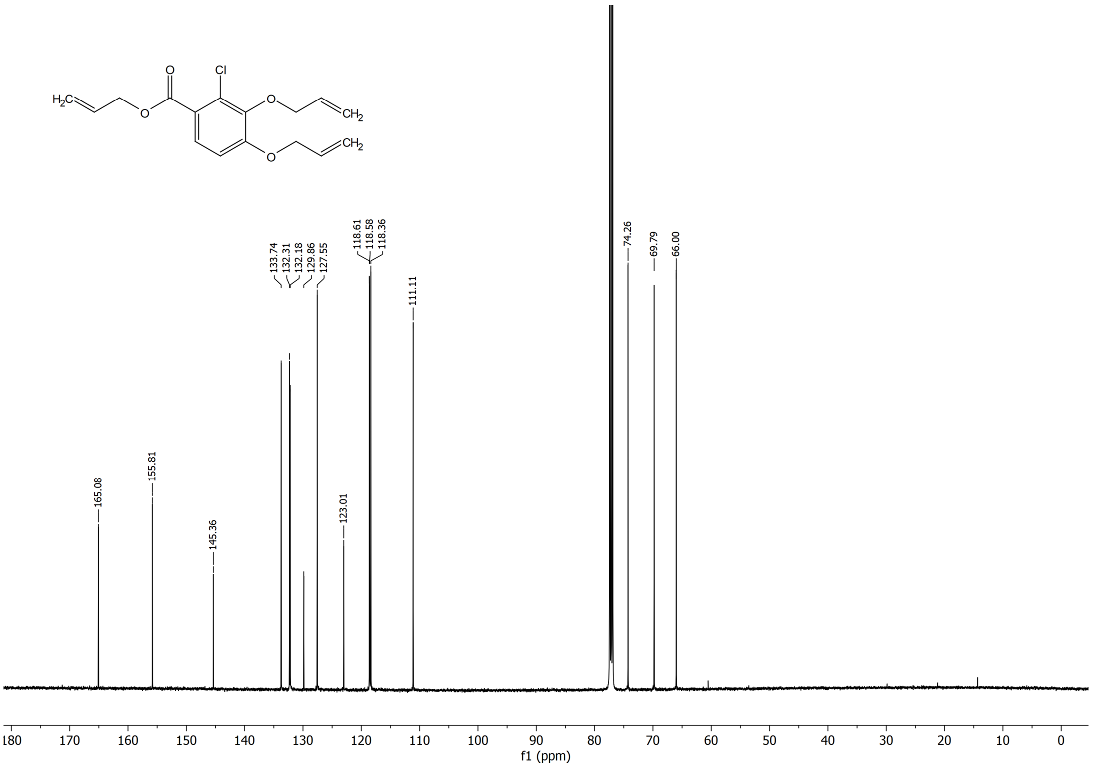

^{1}H NMR spectrum of **31b** (500 MHz, $CDCl_3$)

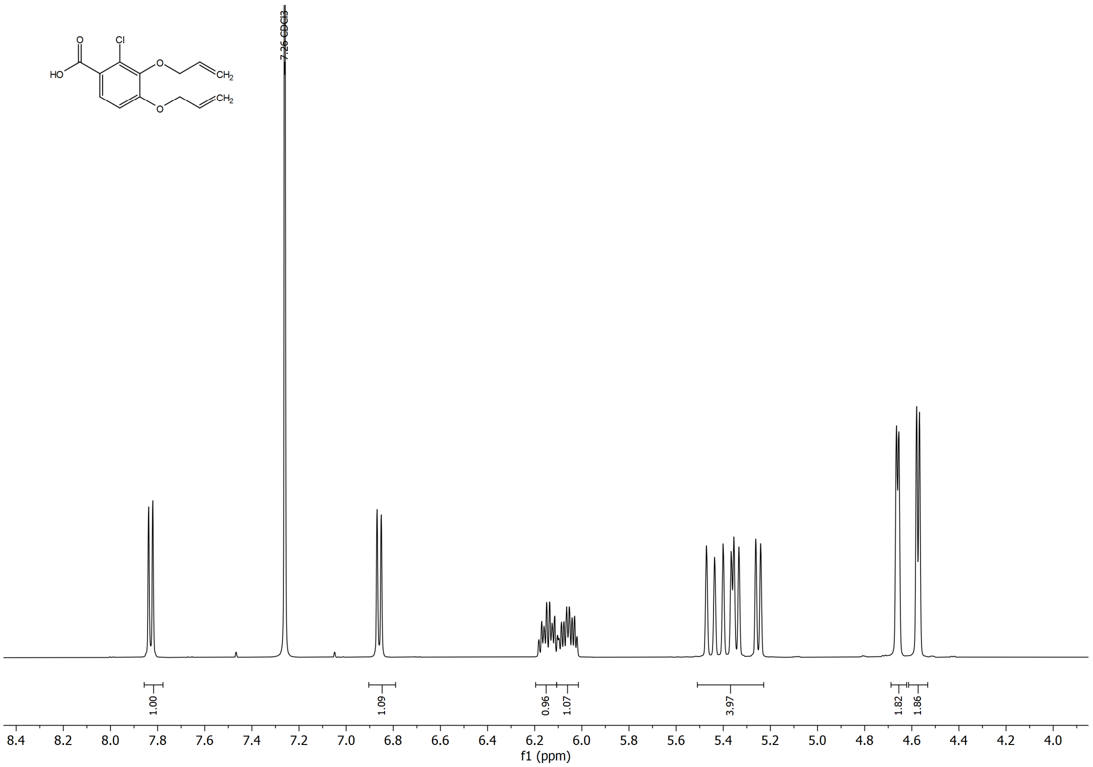

^{13}C NMR spectrum of **31b** (126 MHz, $CDCl_3$)

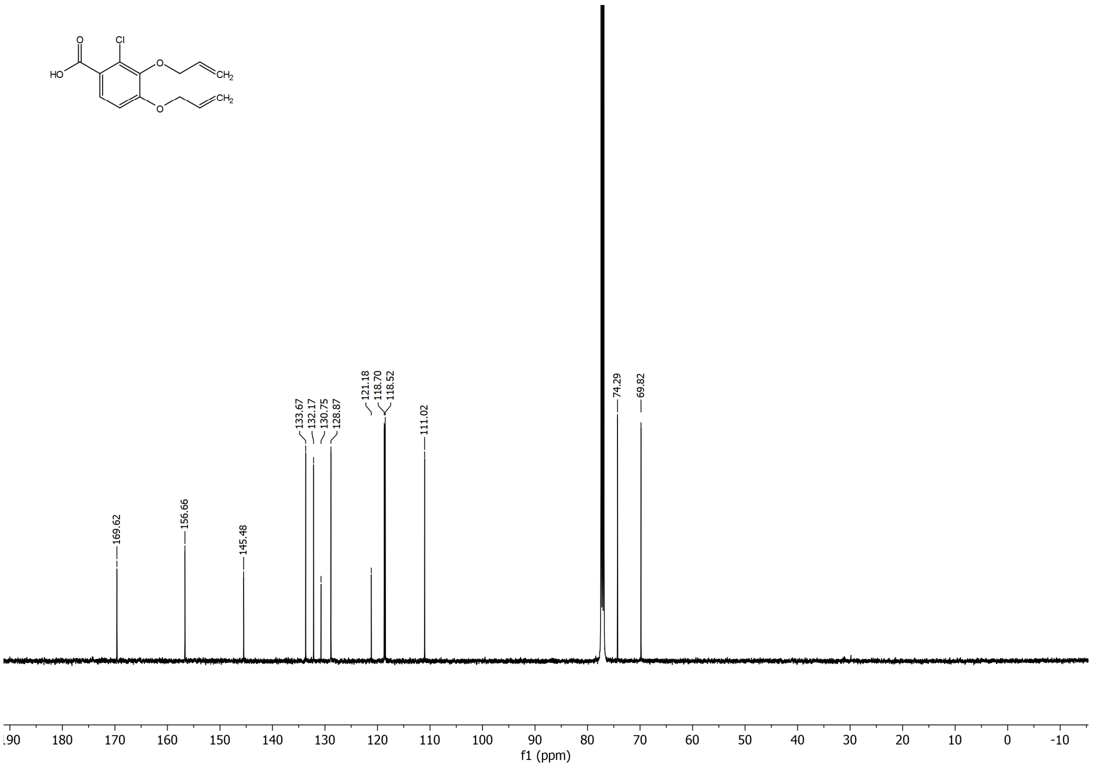

^{1}H NMR spectrum of **32** (500 MHz, acetone-d_6)

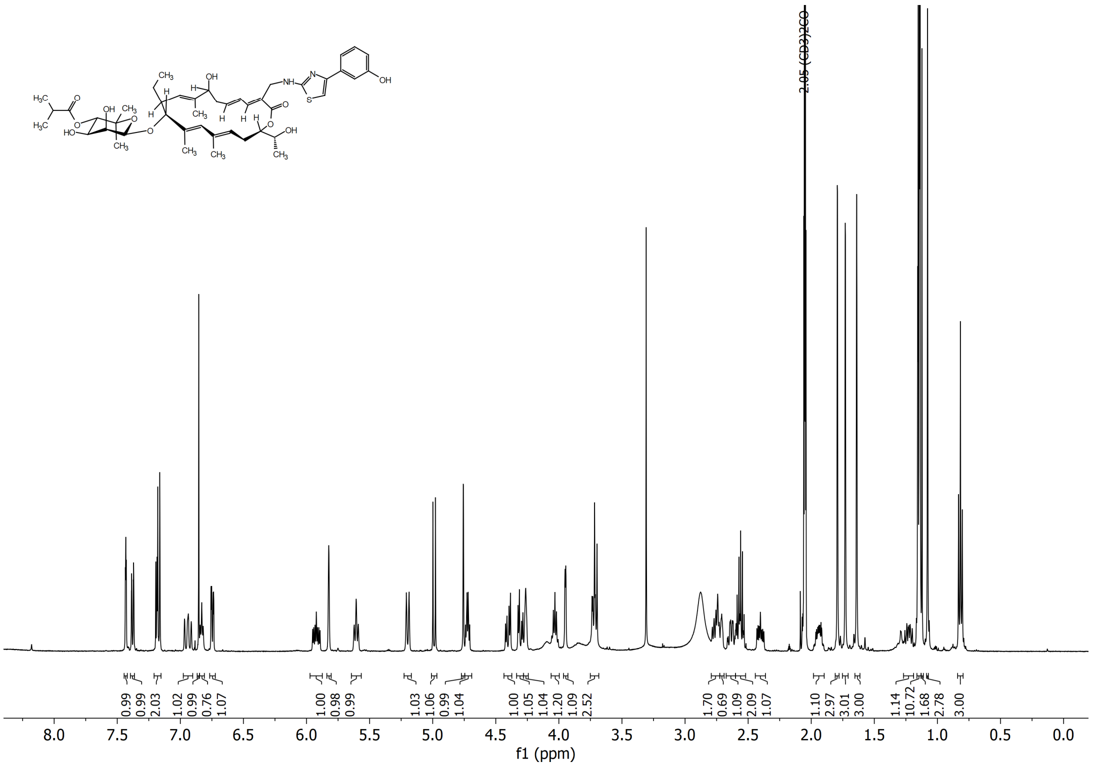

^{13}C NMR spectrum of **32** (126 MHz, acetone-d_6)

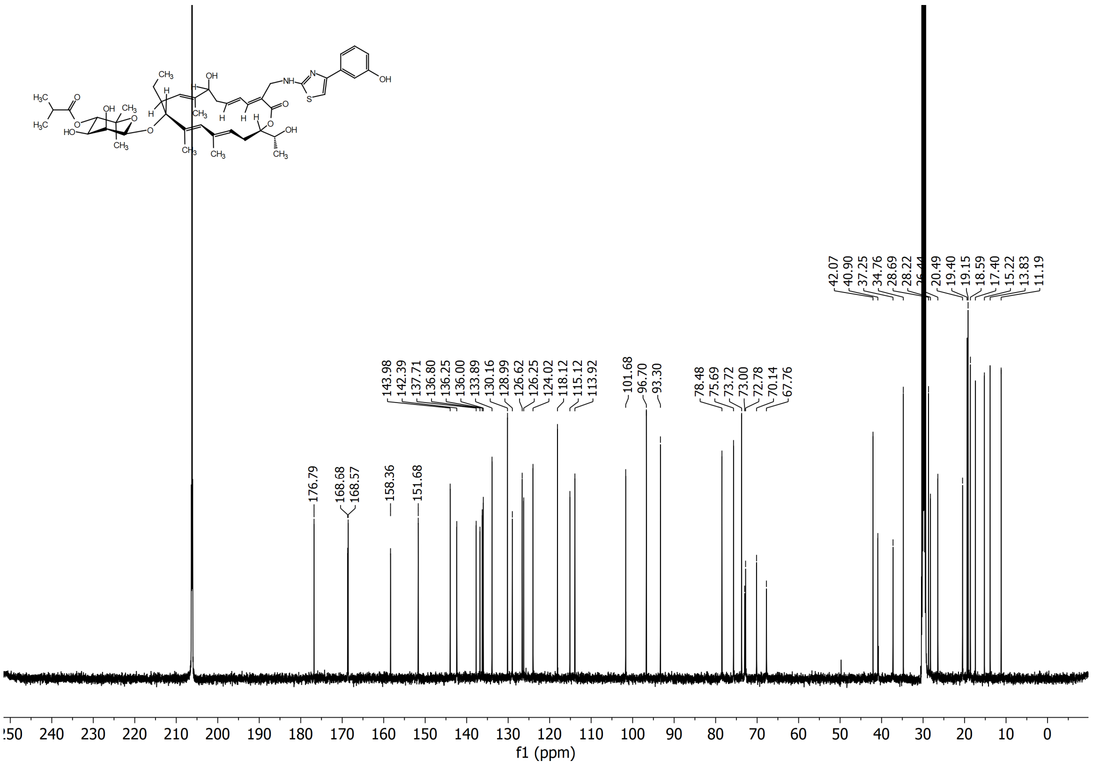

COSY spectrum of **32** (500 MHz, acetone-d_6)

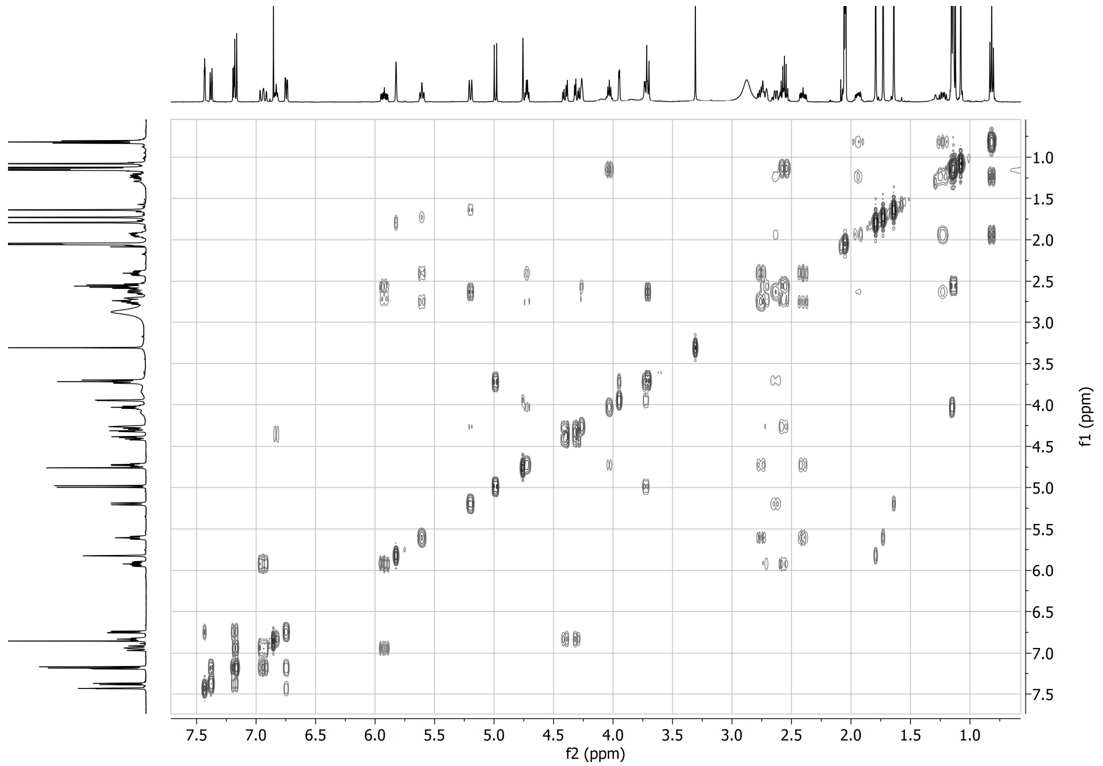

TOCSY spectrum of **32** (500 MHz, acetone-d_6)

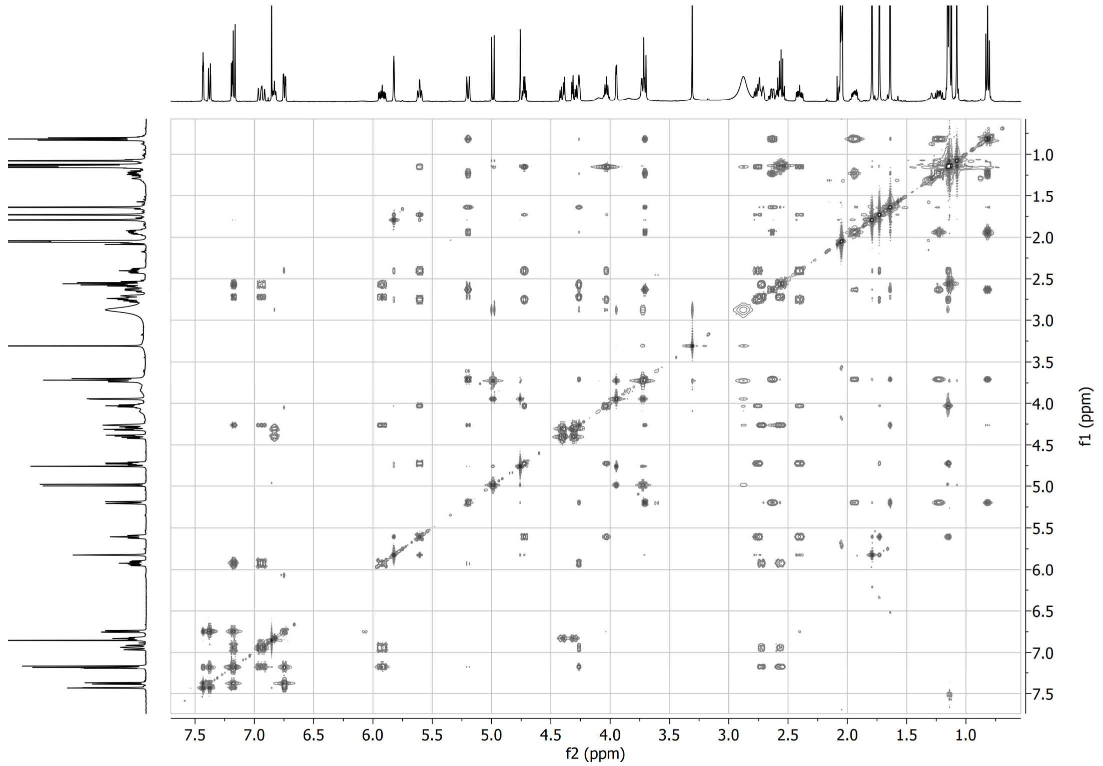

HSQC spectrum of **32** (500 MHz 126 MHz, acetone-d_6)

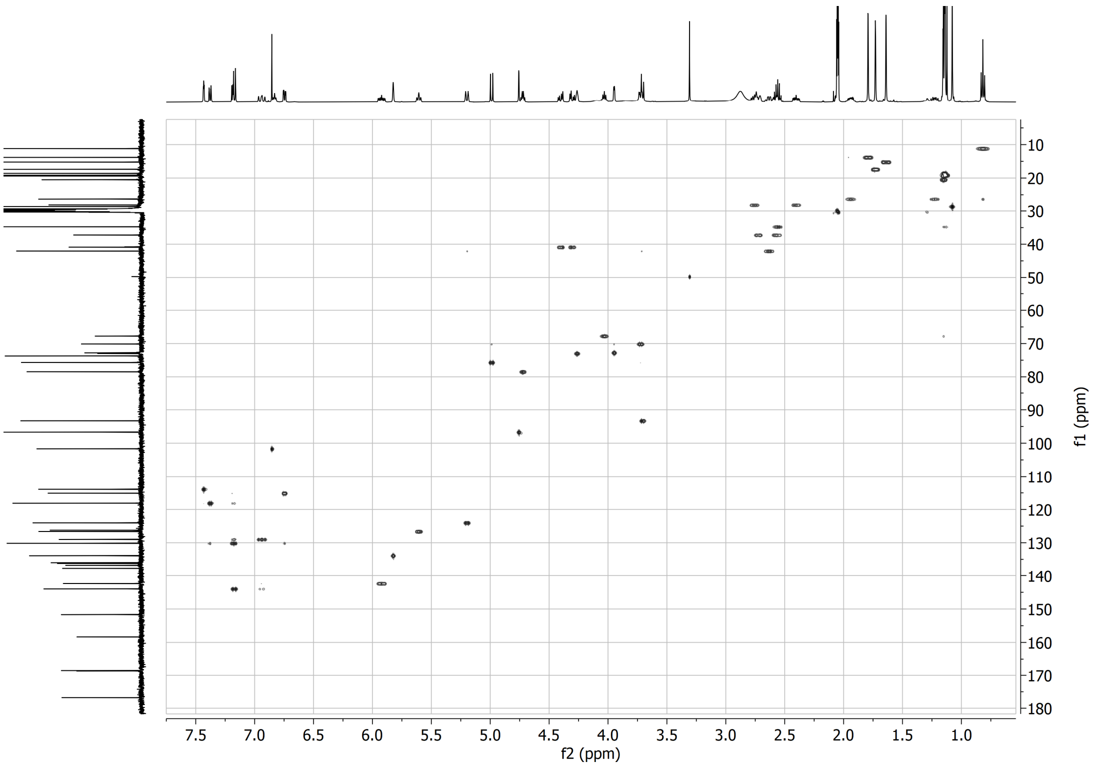

HMBC spectrum of **32** (500 MHz 126 MHz, acetone-d_6)

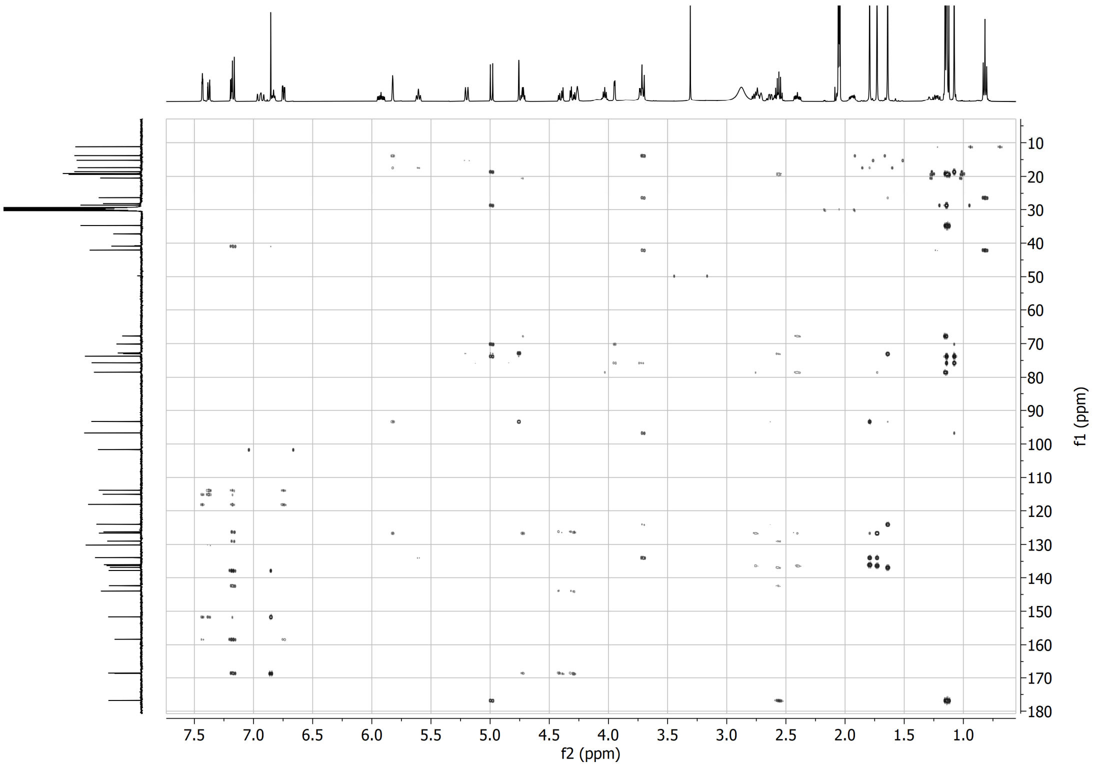

^{1}H NMR spectrum of **32a** (400 MHz, $CDCl_3$)

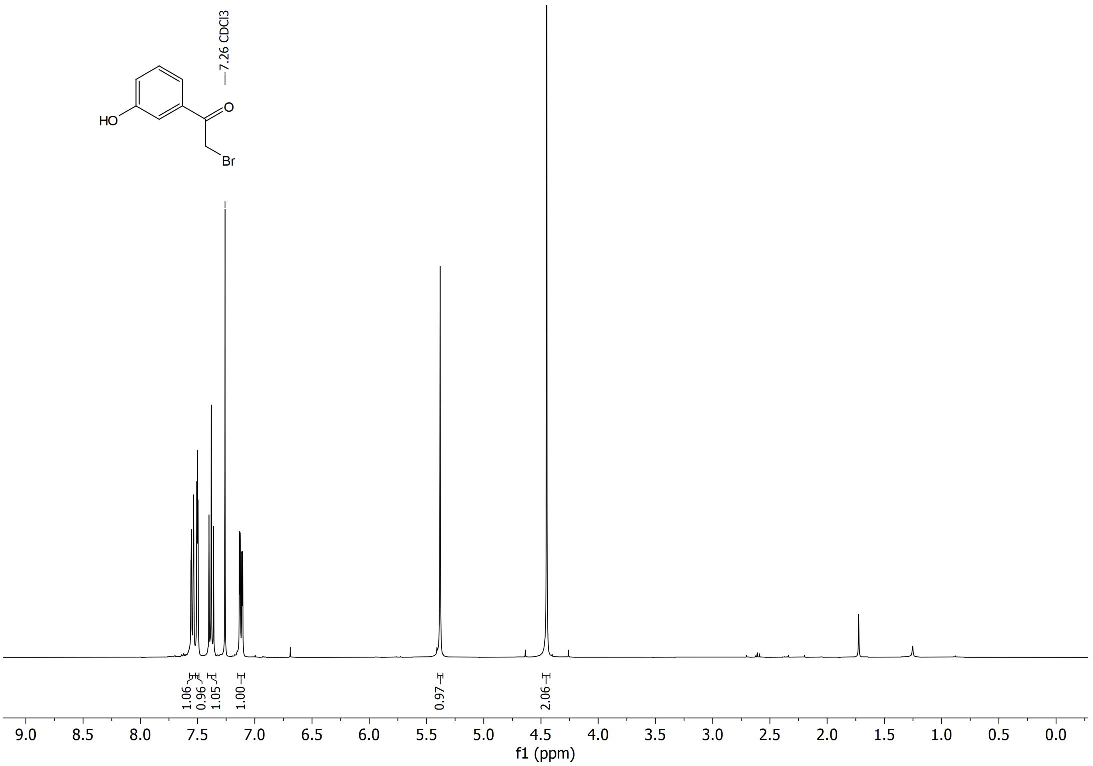

^{1}H NMR spectrum of **32b** (400 MHz, DMSO-d_6)

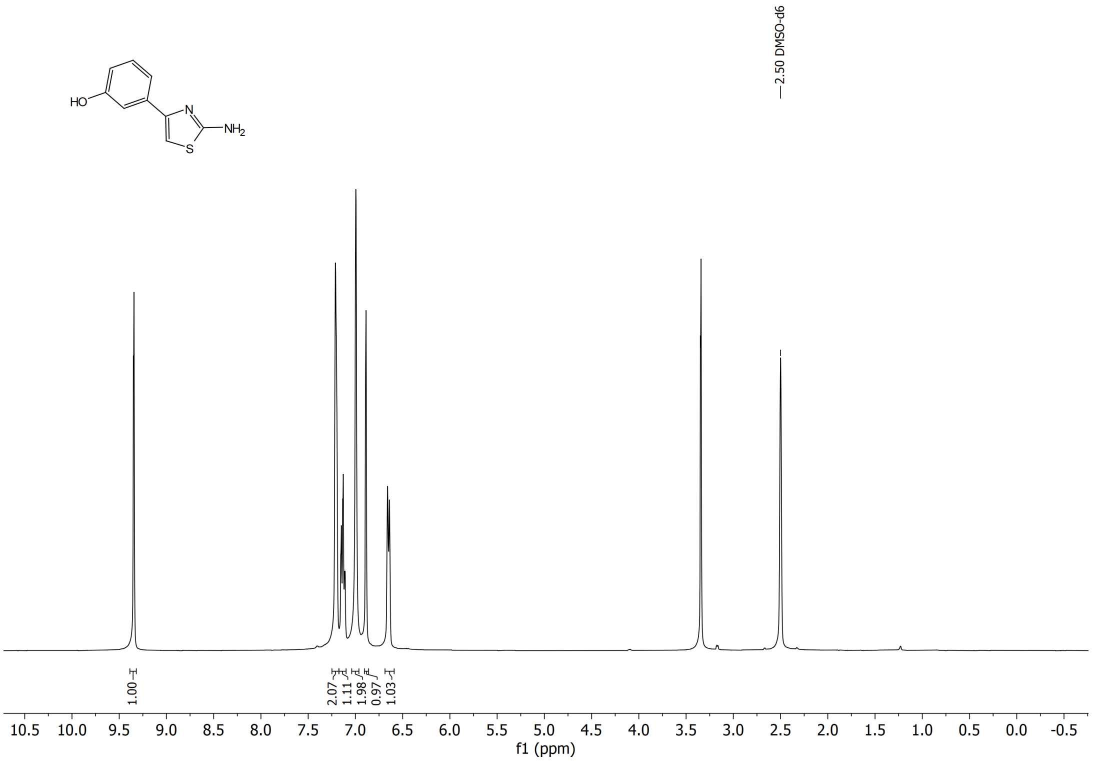

^{1}H NMR spectrum of **33** (500 MHz, acetone-d_6)

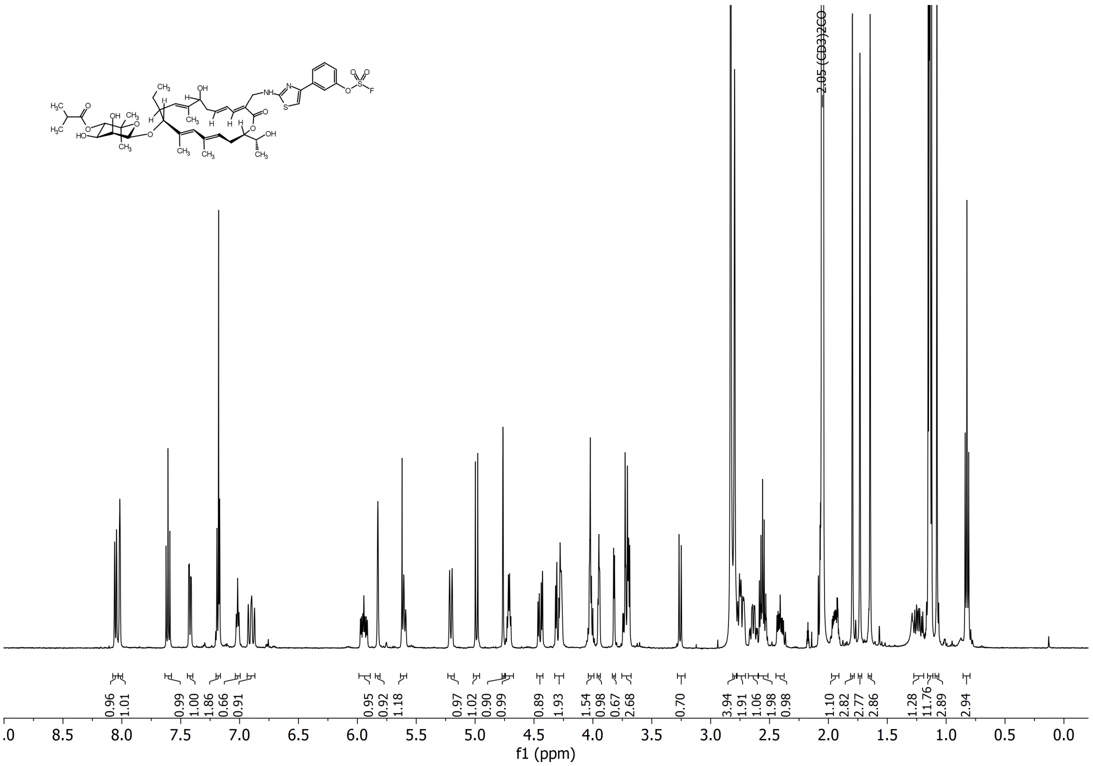

^{13}C NMR spectrum of **33** (126 MHz, acetone-d_6)

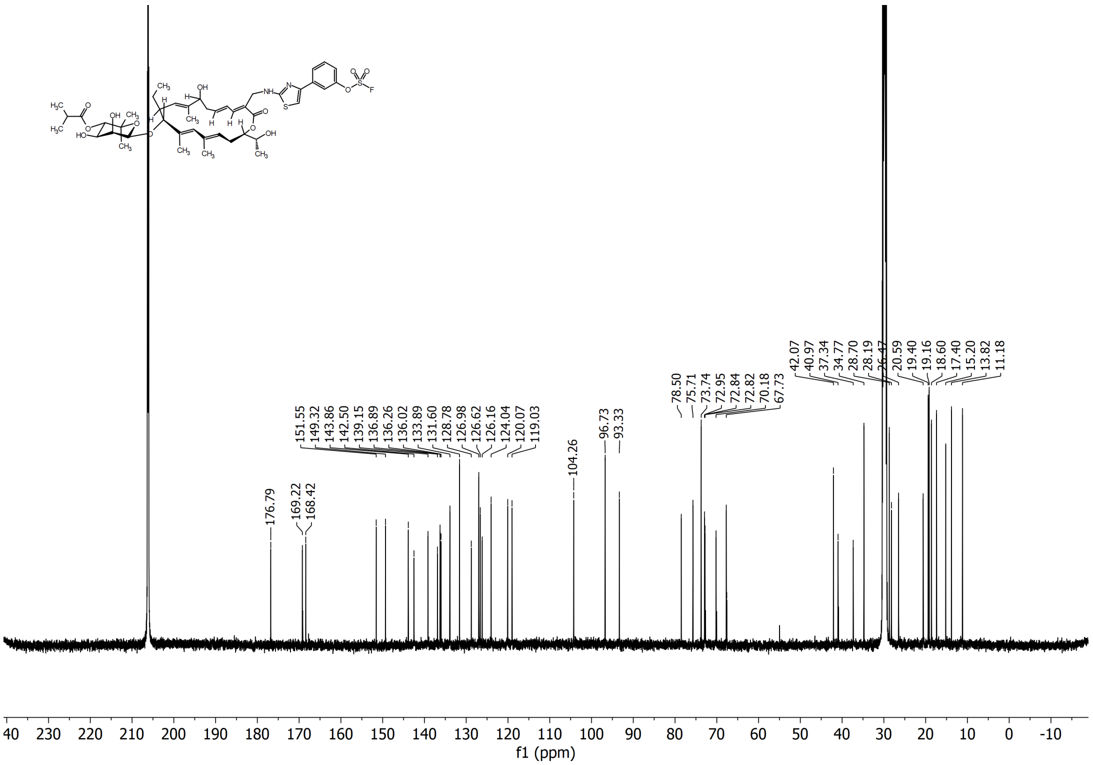

^{19}F NMR spectrum of **33** (471 MHz, acetone-d_6)

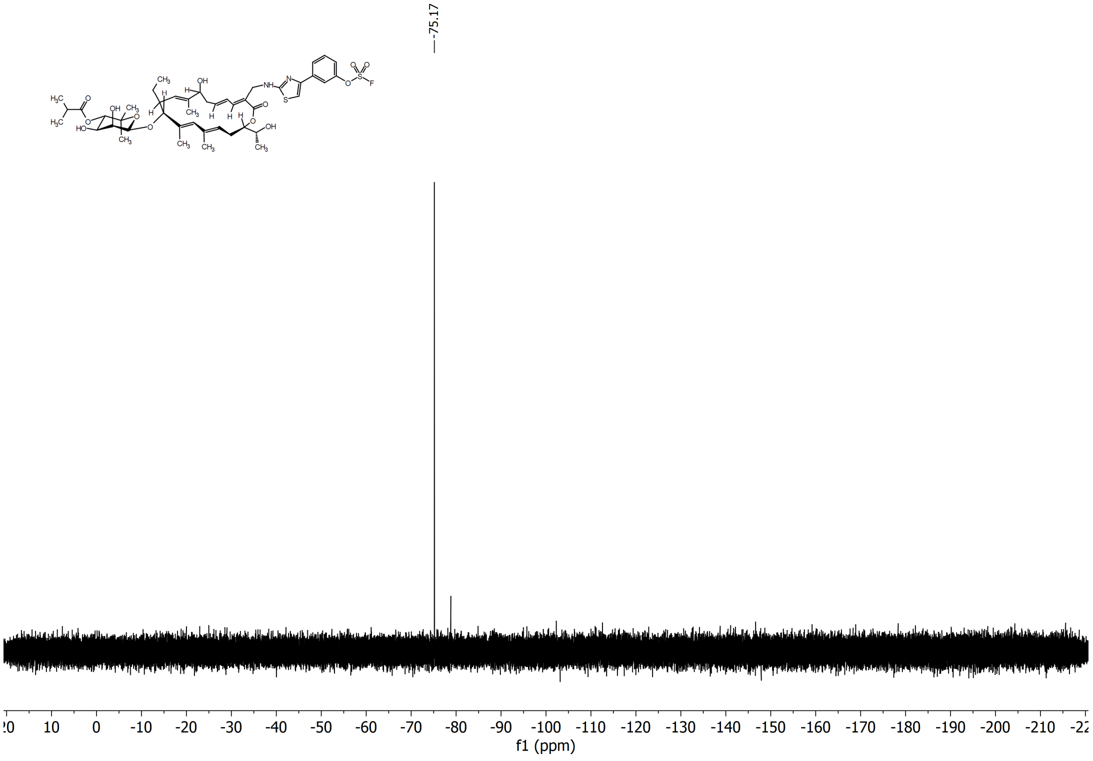

^{1}H NMR spectrum of **34** (500 MHz, acetone-d_6)

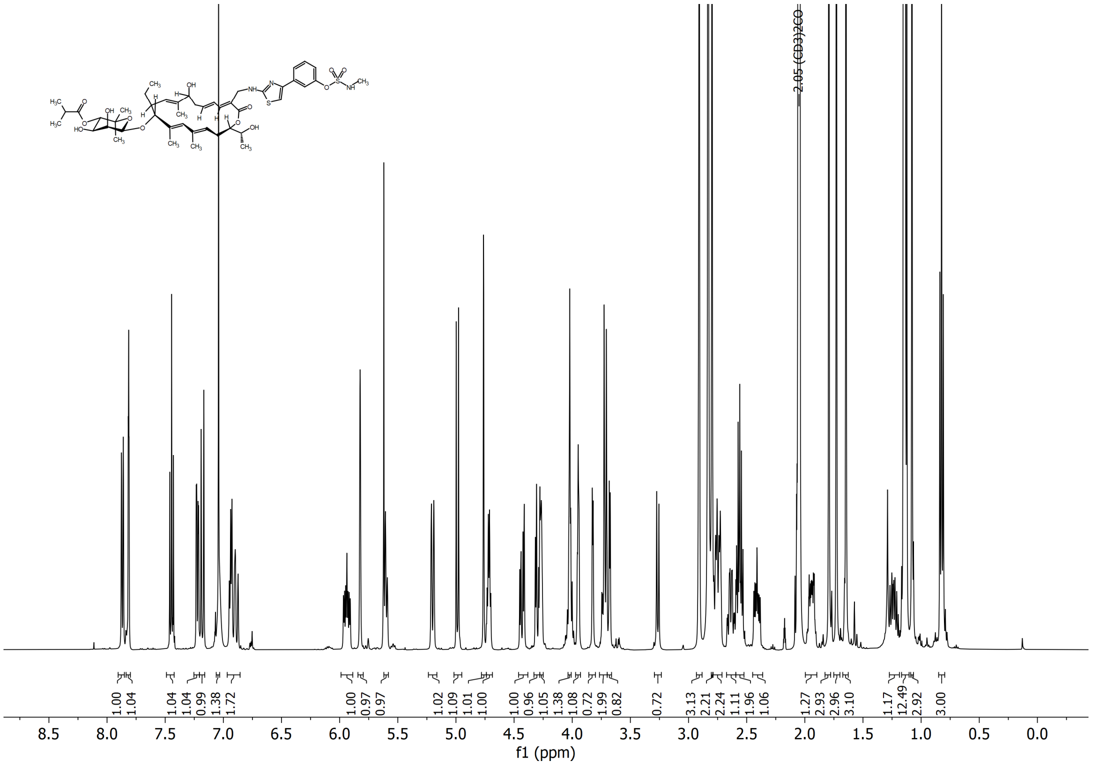

^{13}C NMR spectrum of **34** (126 MHz, acetone-d_6)

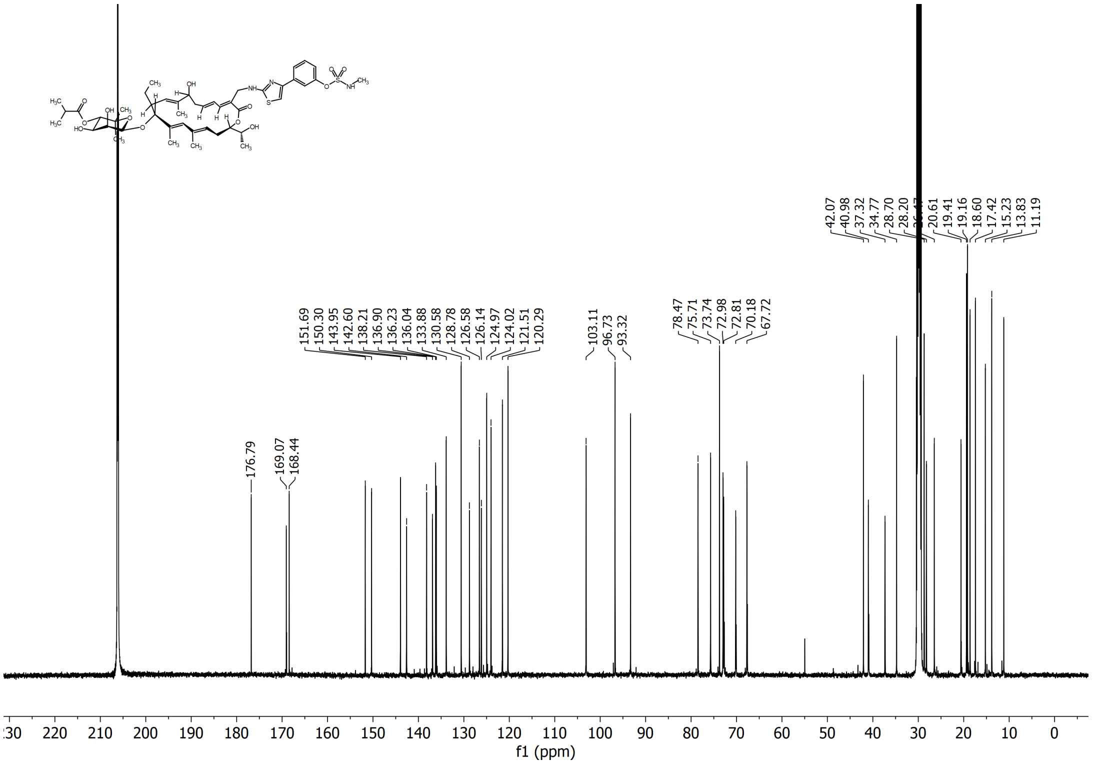

^{1}H NMR spectrum of **35** (500 MHz, acetone-d_6)

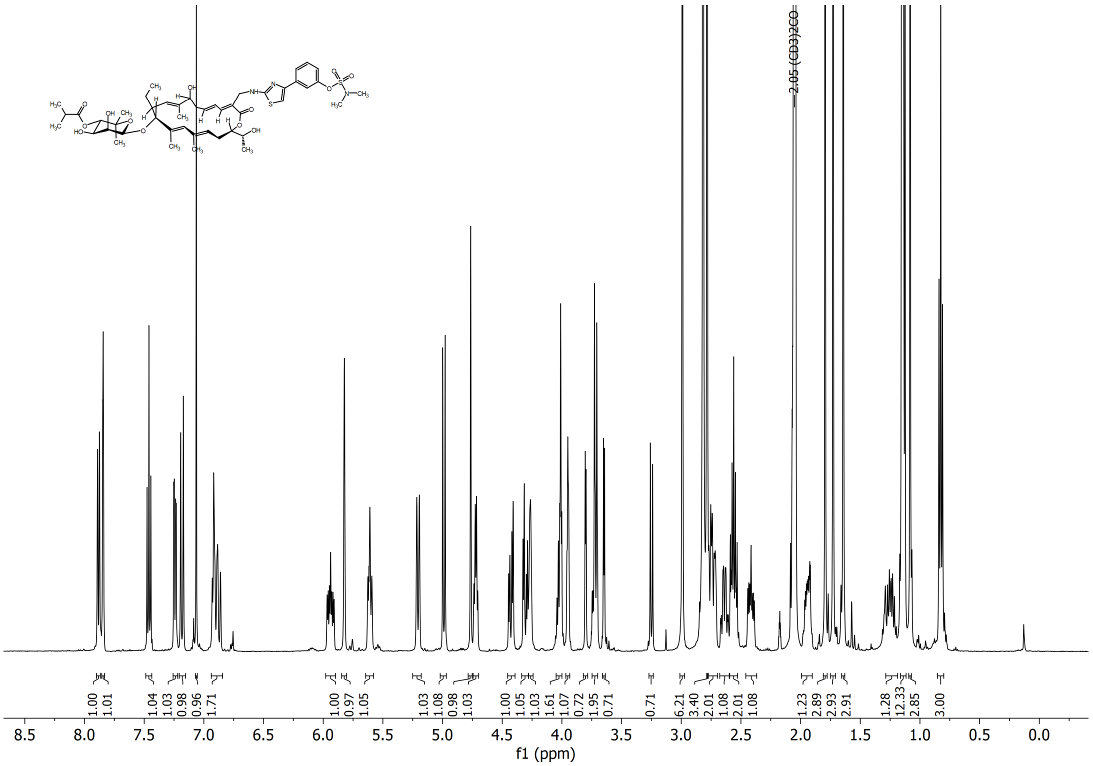

^{13}C NMR spectrum of **35** (126 MHz, acetone-d_6)

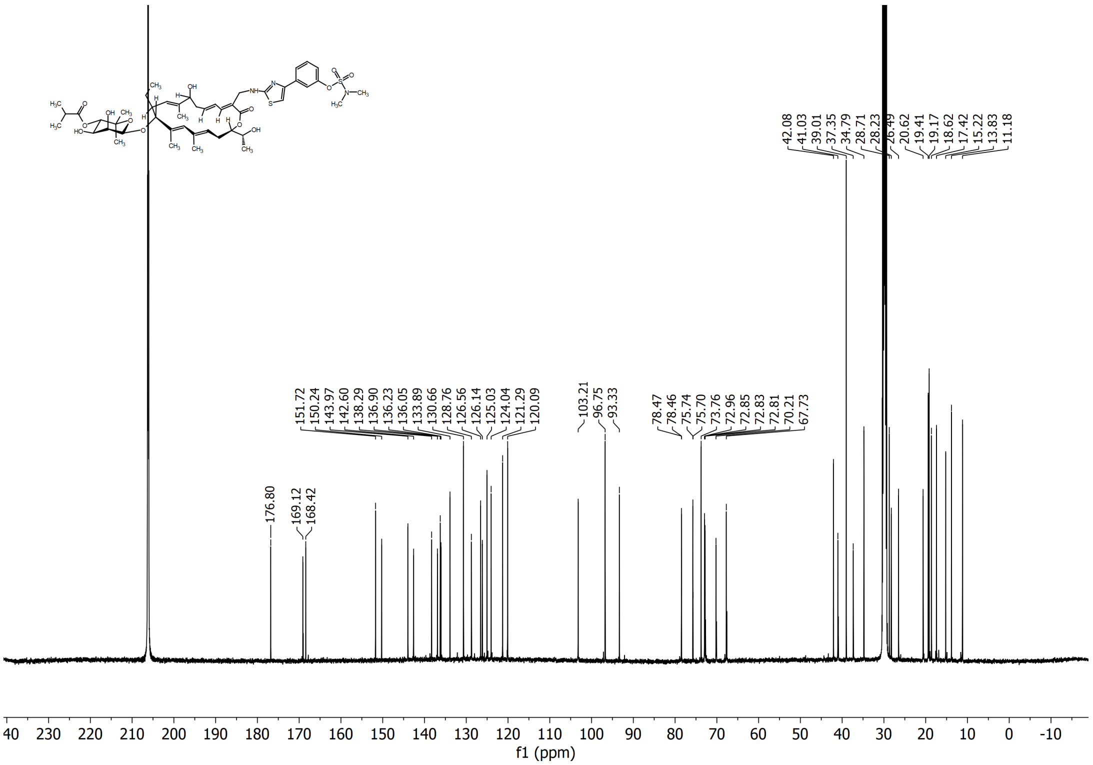

^{1}H NMR spectrum of **36** (500 MHz, acetone-d_6)

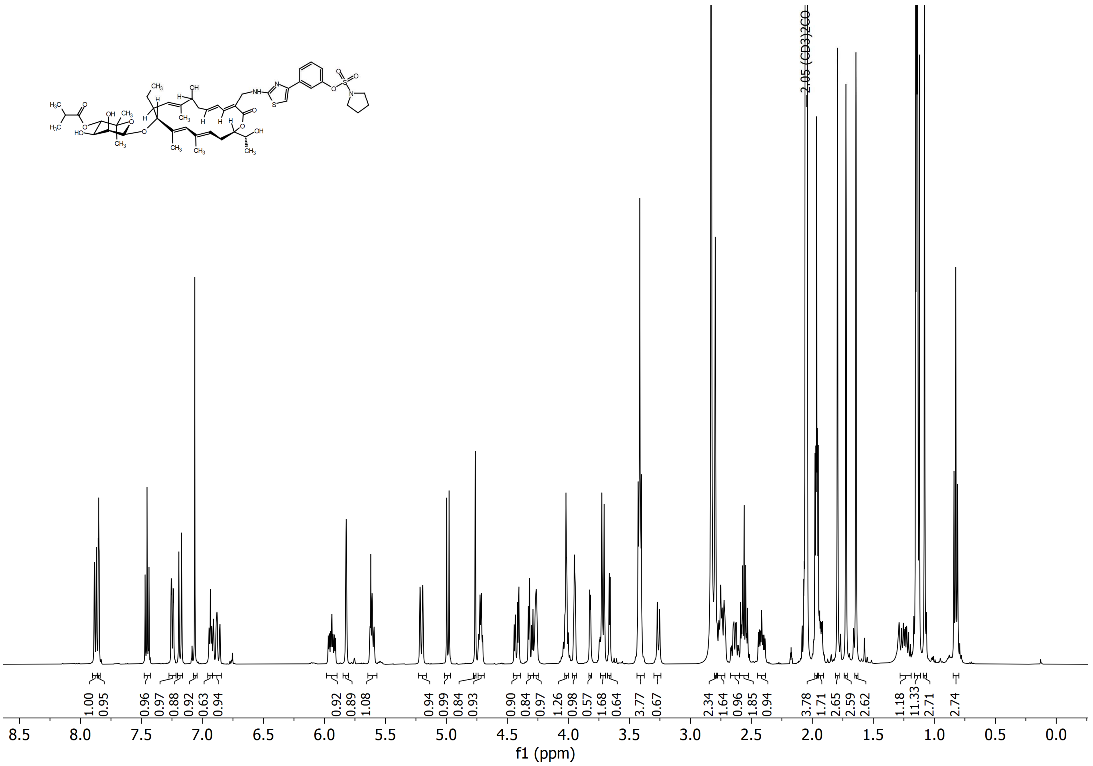

^{13}C NMR spectrum of **36** (126 MHz, acetone-d_6)

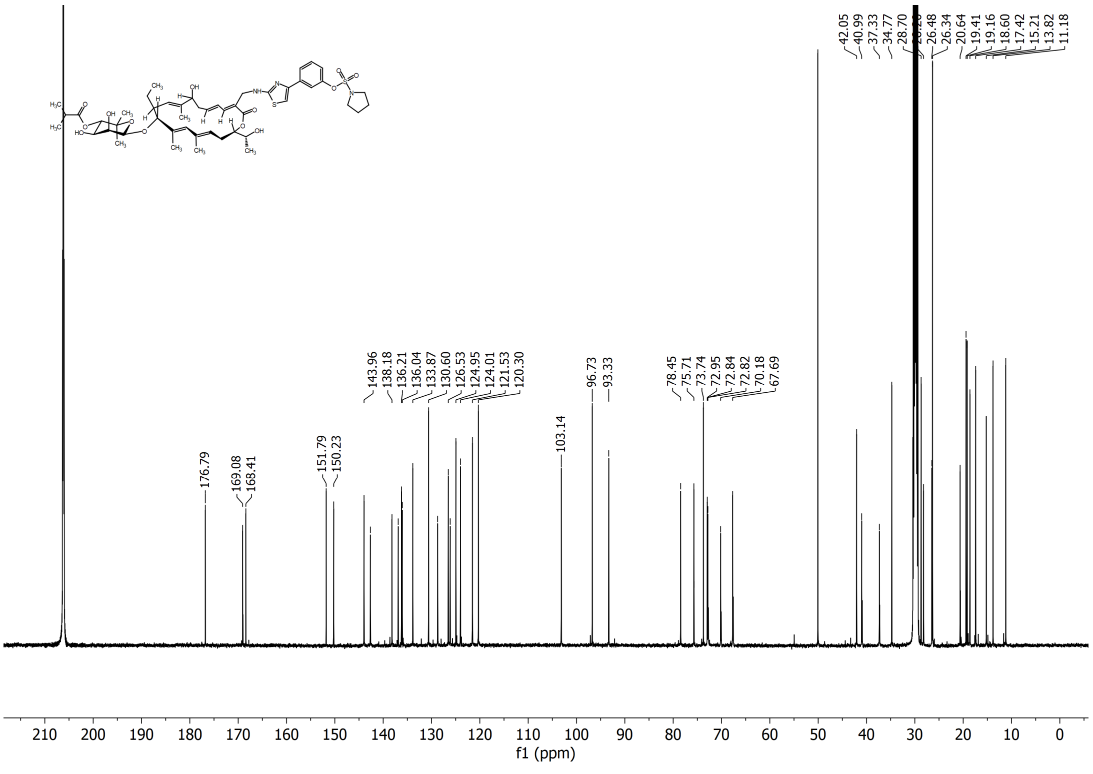

^{1}H NMR spectrum of **37** (500 MHz, acetone-*d_6*)

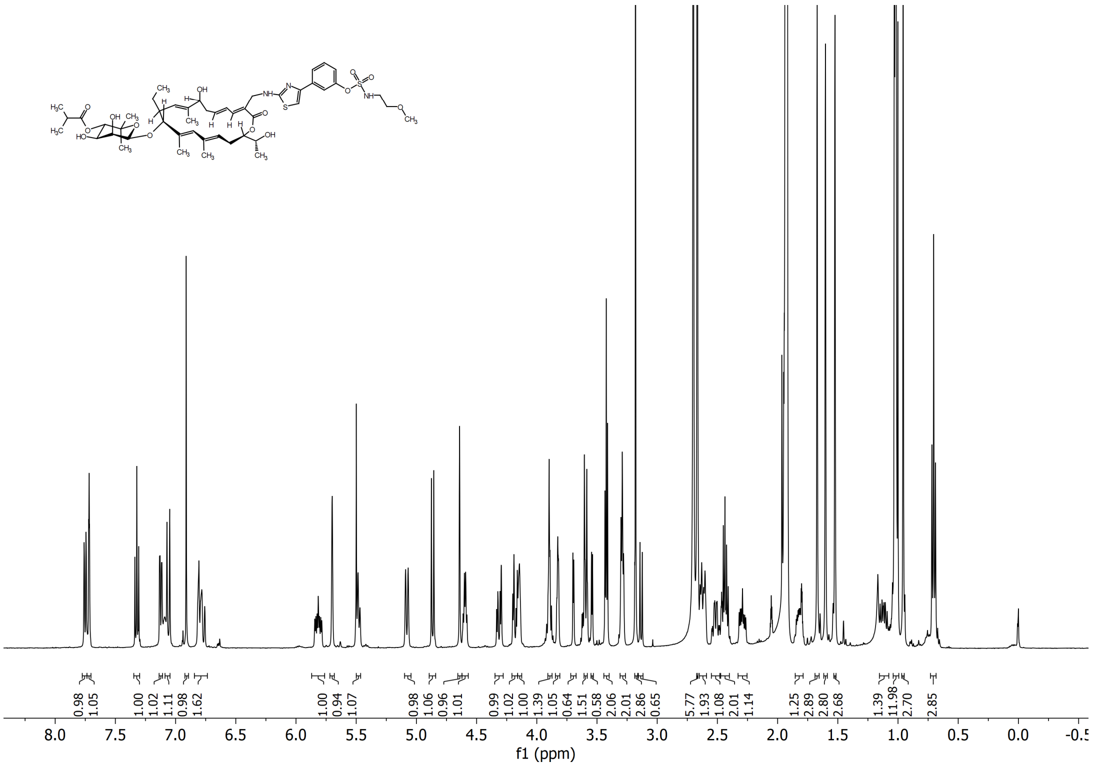

^{13}C NMR spectrum of **37** (126 MHz, acetone-*d_6*)

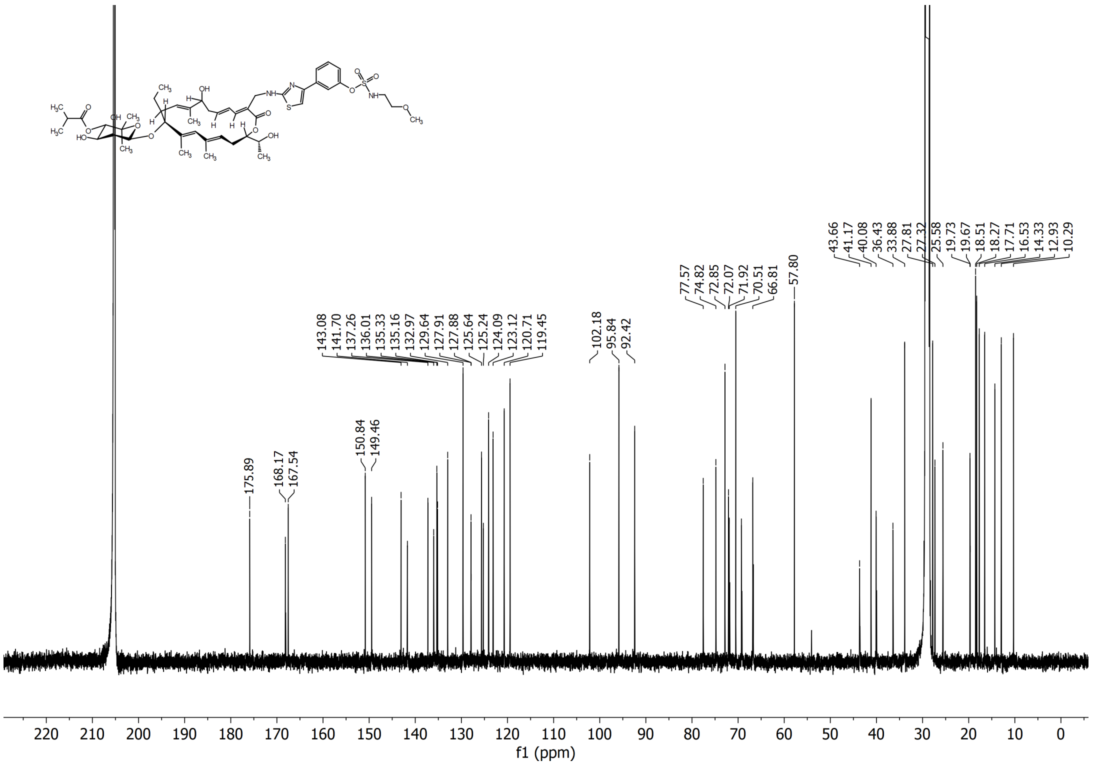

^{1}H NMR spectrum of **38** (500 MHz, acetone-d_6)

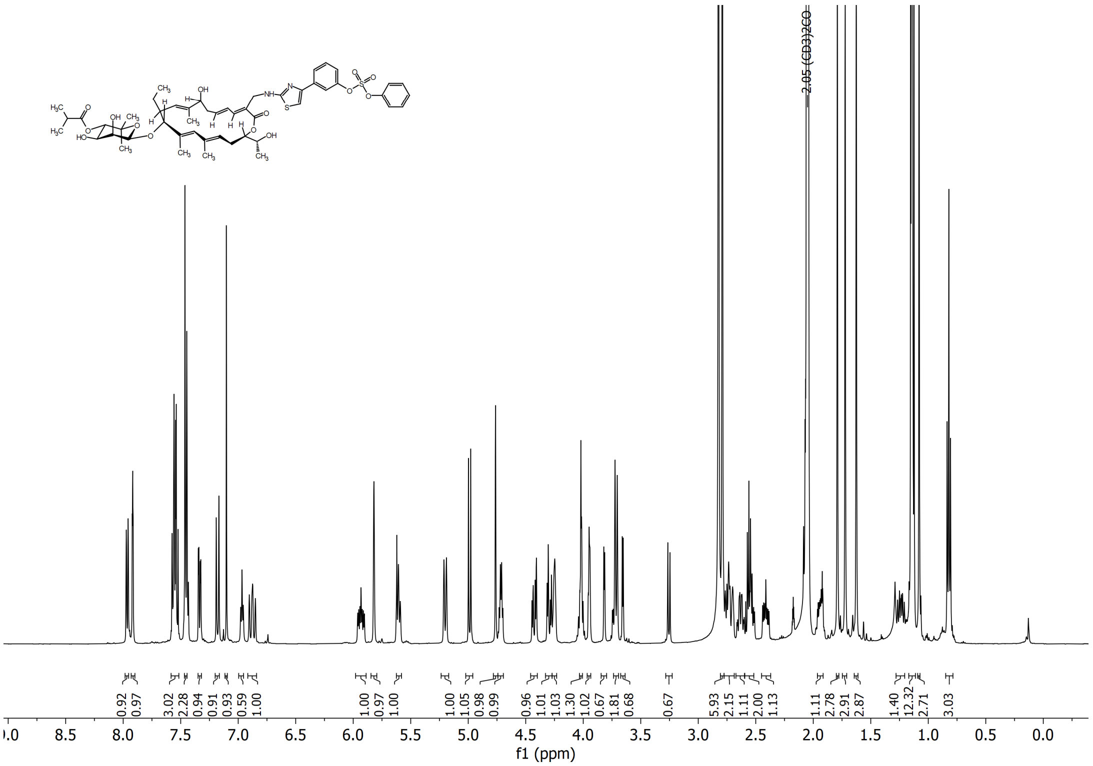

^{13}C NMR spectrum of **38** (126 MHz, acetone-d_6)

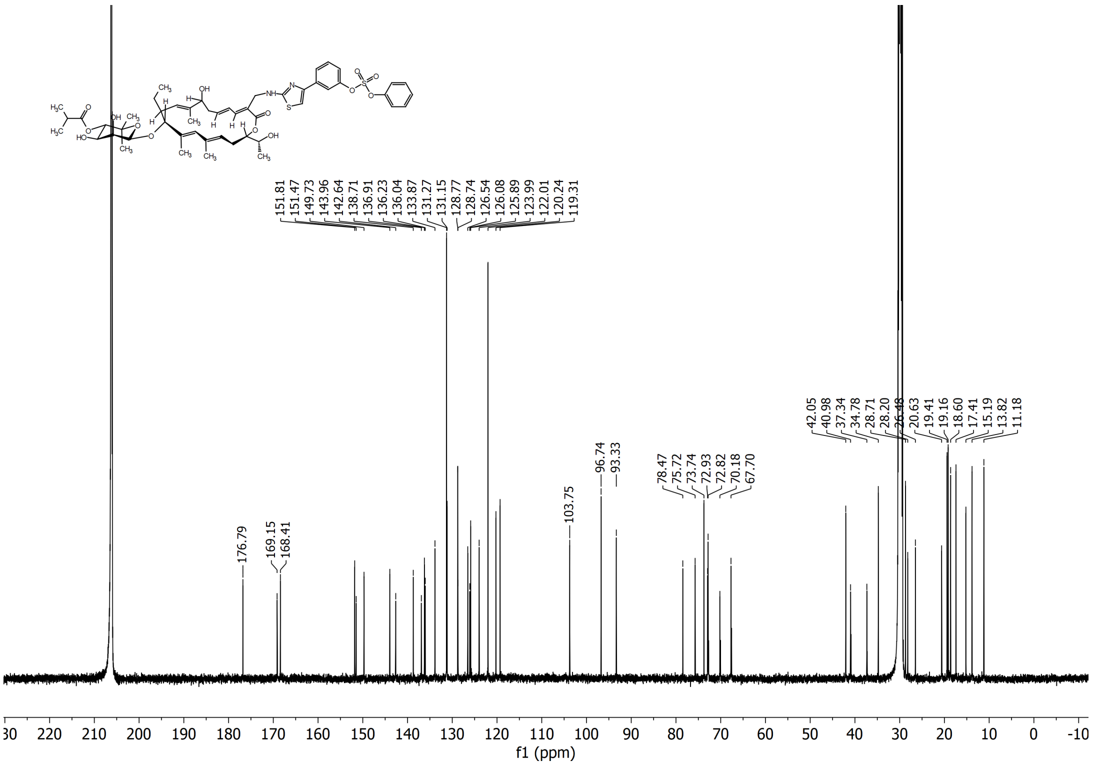

^{1}H NMR spectrum of **2-(2-bromo-5-methoxyphenyl)-1*H*-imidazole** (400 MHz, $CDCl_3$)

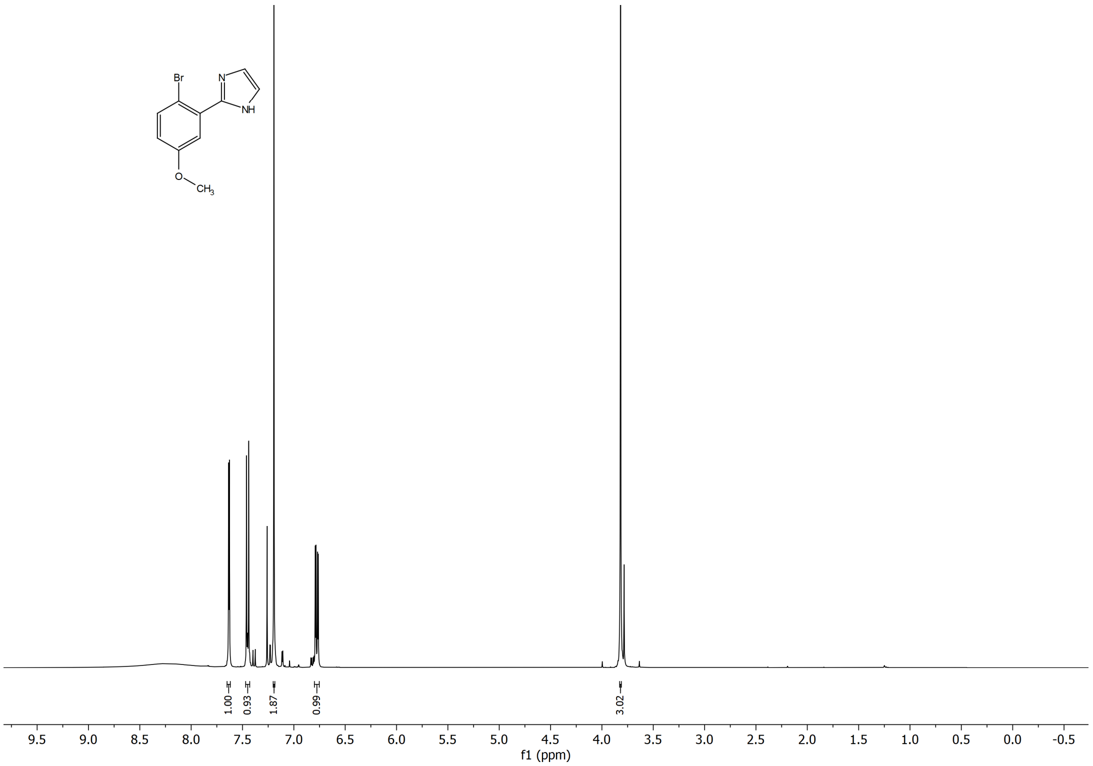

^{1}H NMR spectrum of **2-(2-bromo-5-methoxyphenyl)-1-methyl-1*H*-imidazole** (400 MHz, $CDCl_3$)

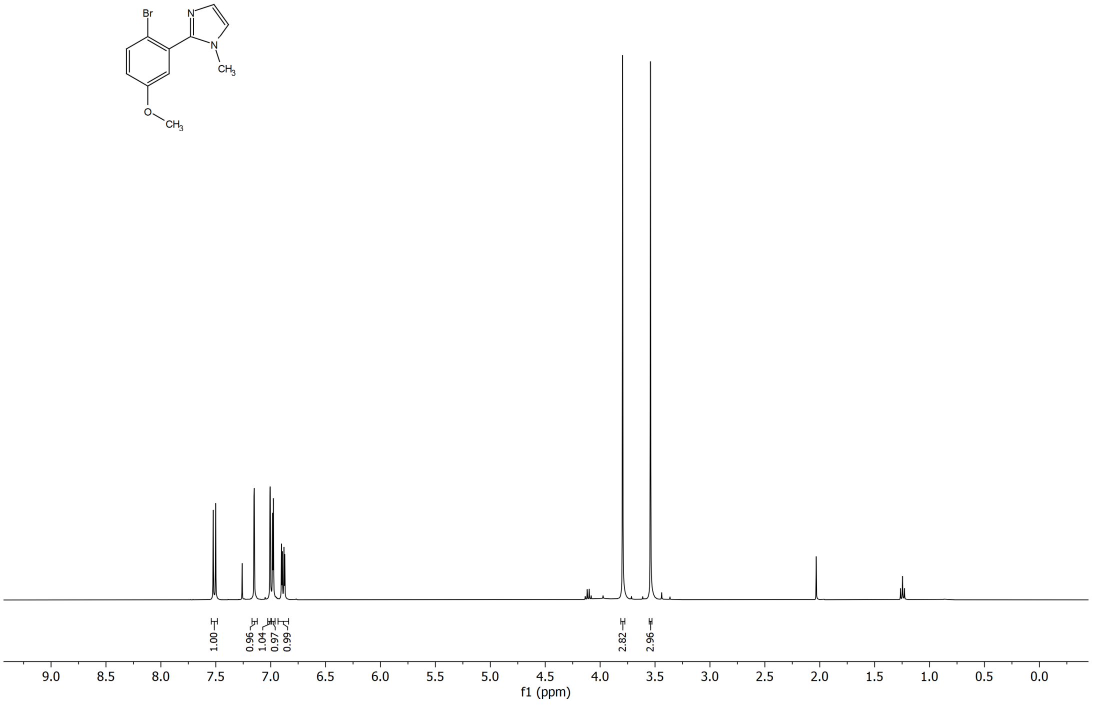

^{1}H NMR spectrum of **Shimada's catalyst** (400 MHz, methanol-d_4)

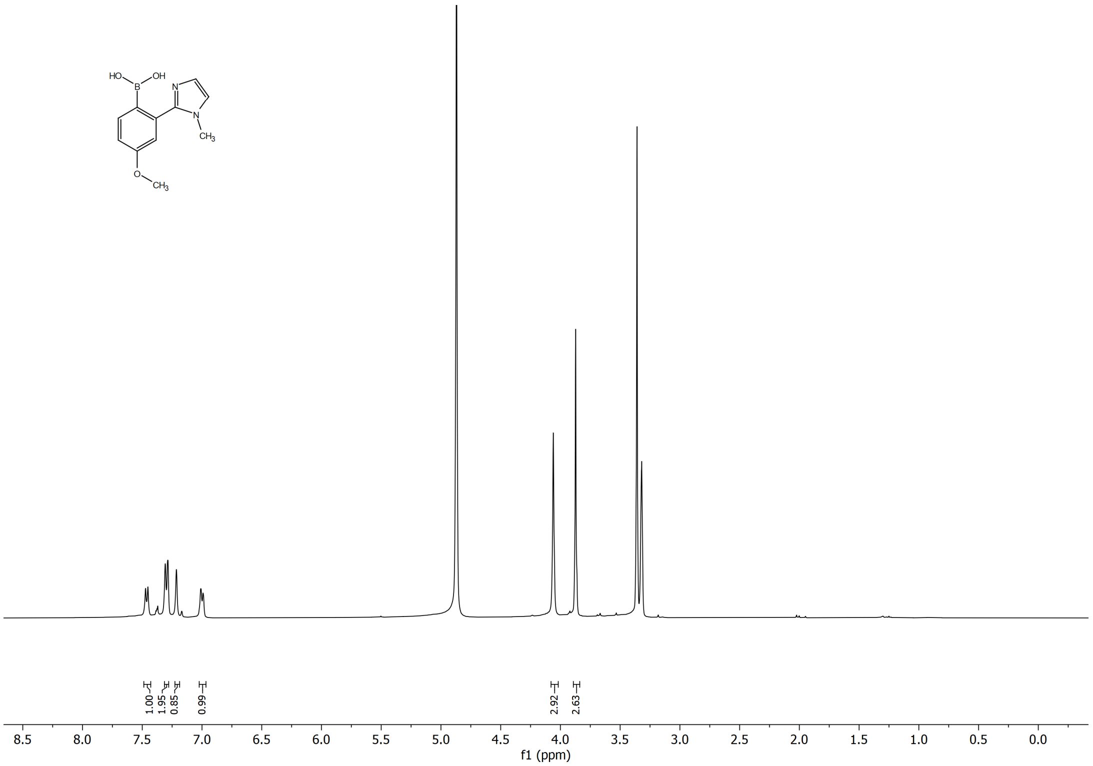

^{11}B NMR spectrum of **Shimada's catalyst** (161 MHz, methanol-d_4)

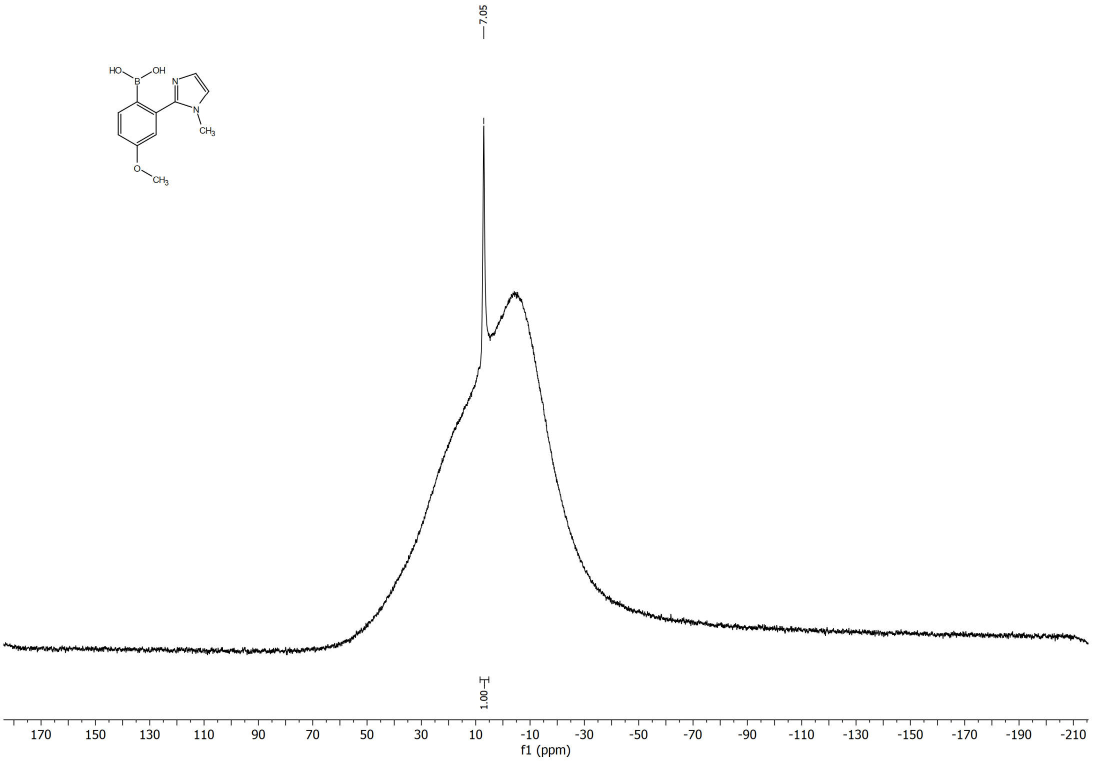

^{1}**H NMR** spectrum of **OP1118** (**39**) (400 MHz, acetone-d_6)

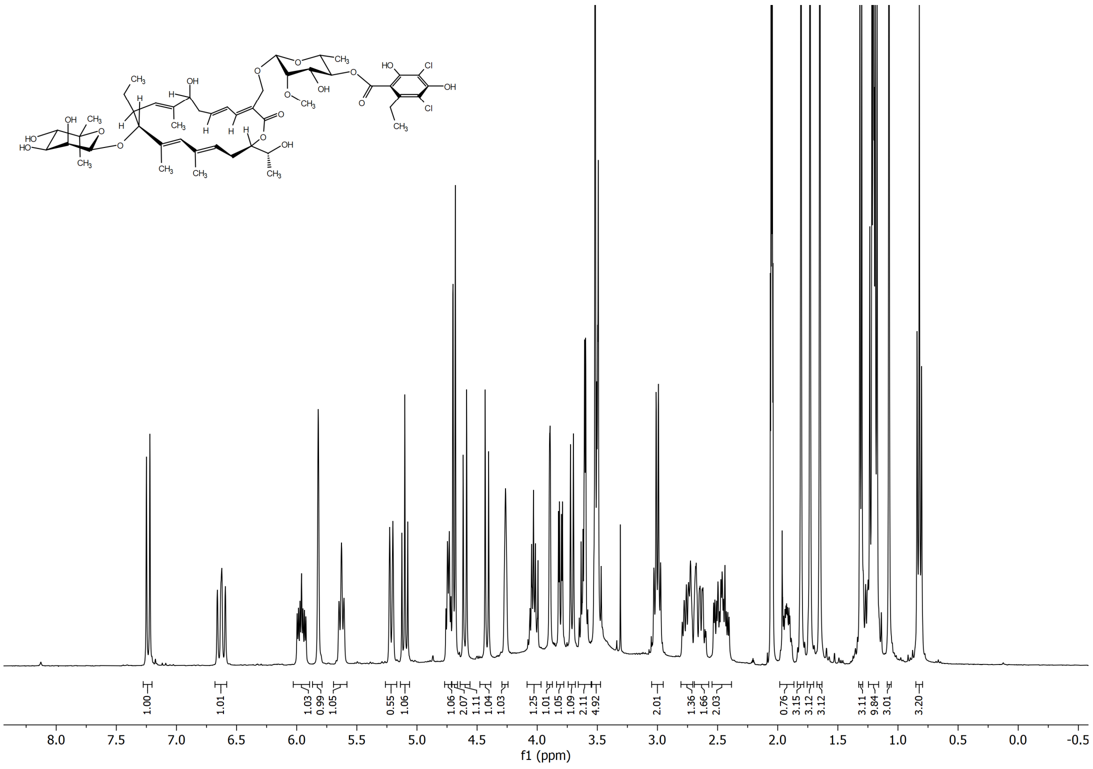

^{1}**H NMR** spectrum of **40** (500 MHz, acetone-d_6)

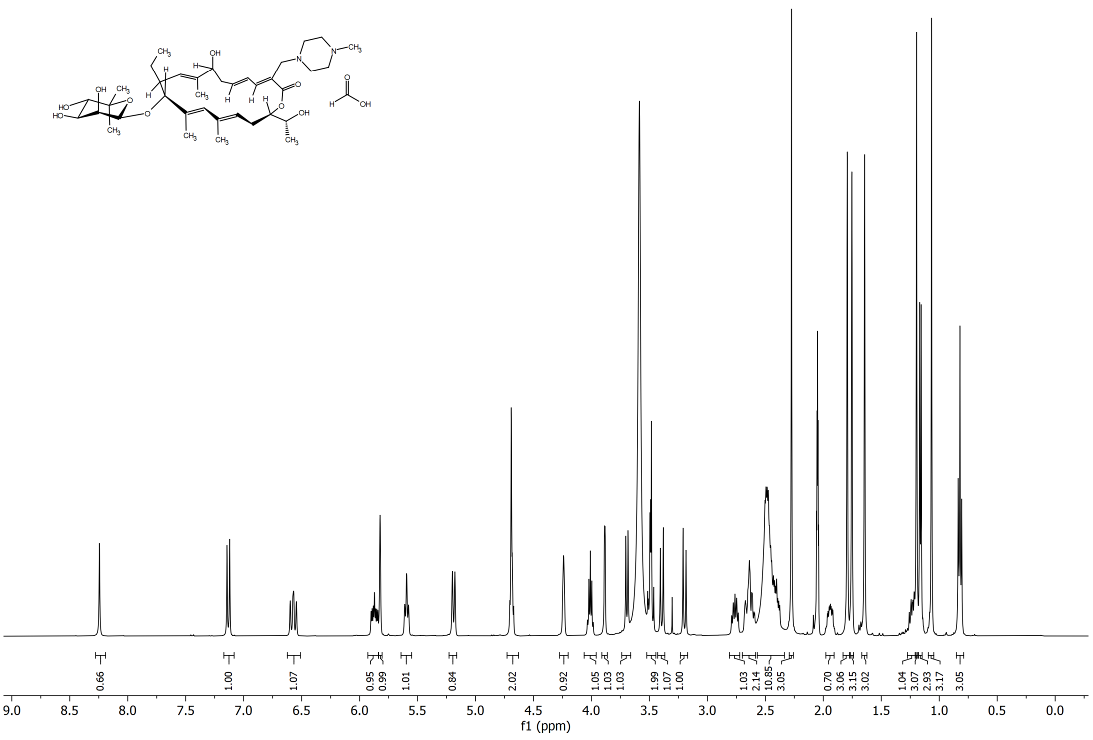

^{13}**C NMR** spectrum of **40** (126 MHz, acetone-d_6)

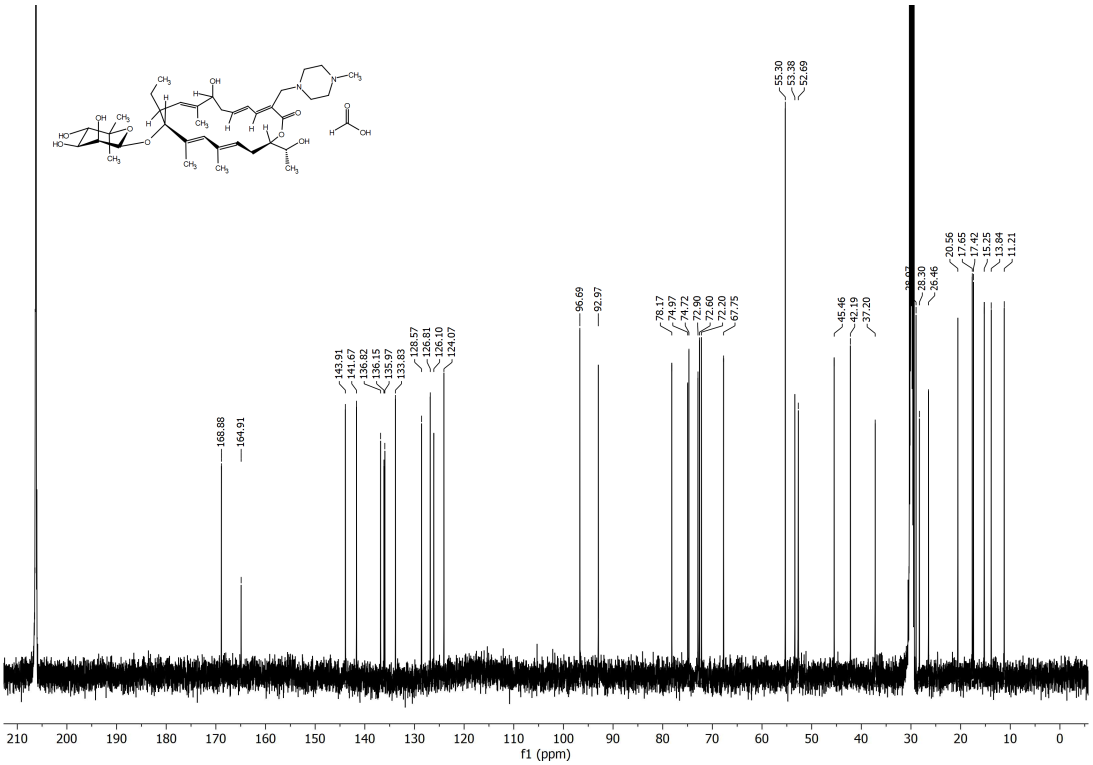

COSY spectrum of **40** (500 MHz, acetone-d_6)

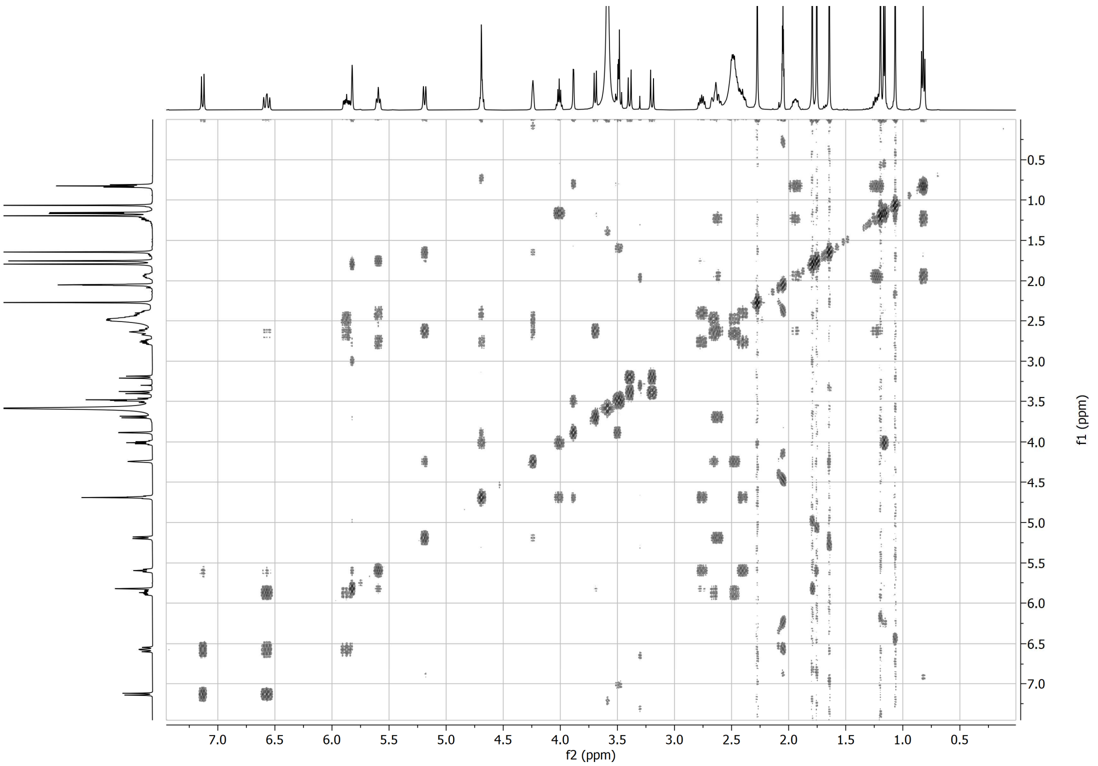

TOCSY spectrum of **40** (500 MHz, acetone-d_6)

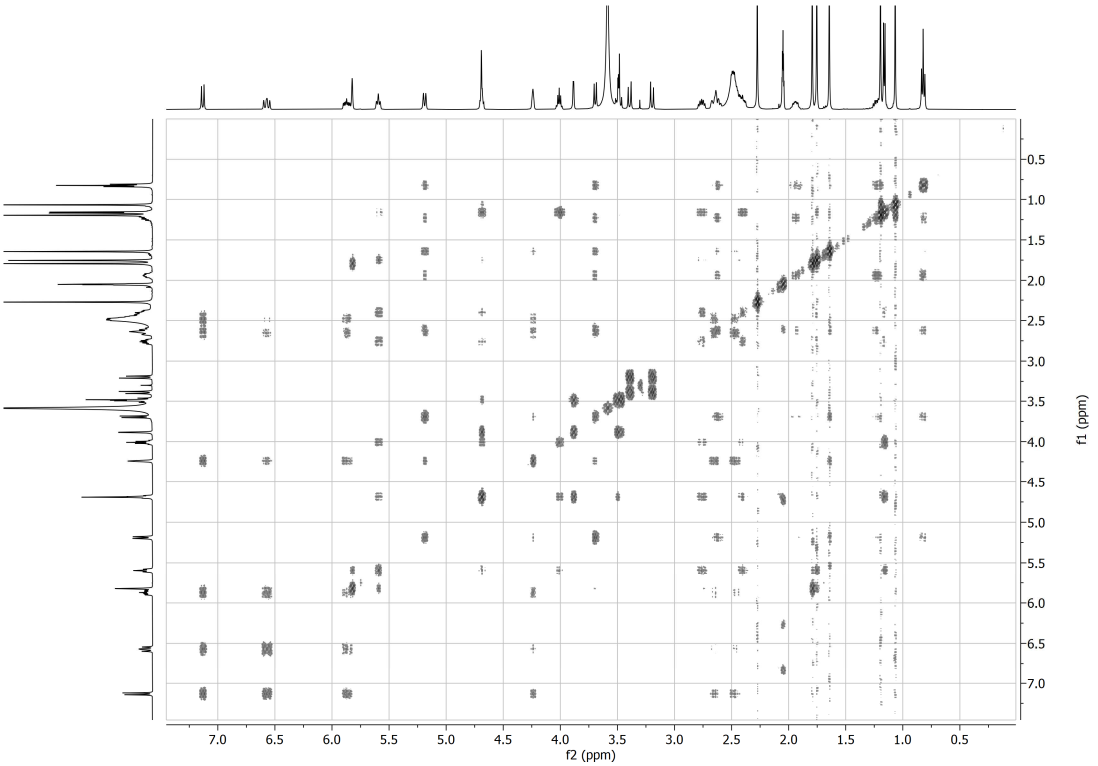

HSQC spectrum of **40** (500 MHz 126 MHz, acetone-d_6)

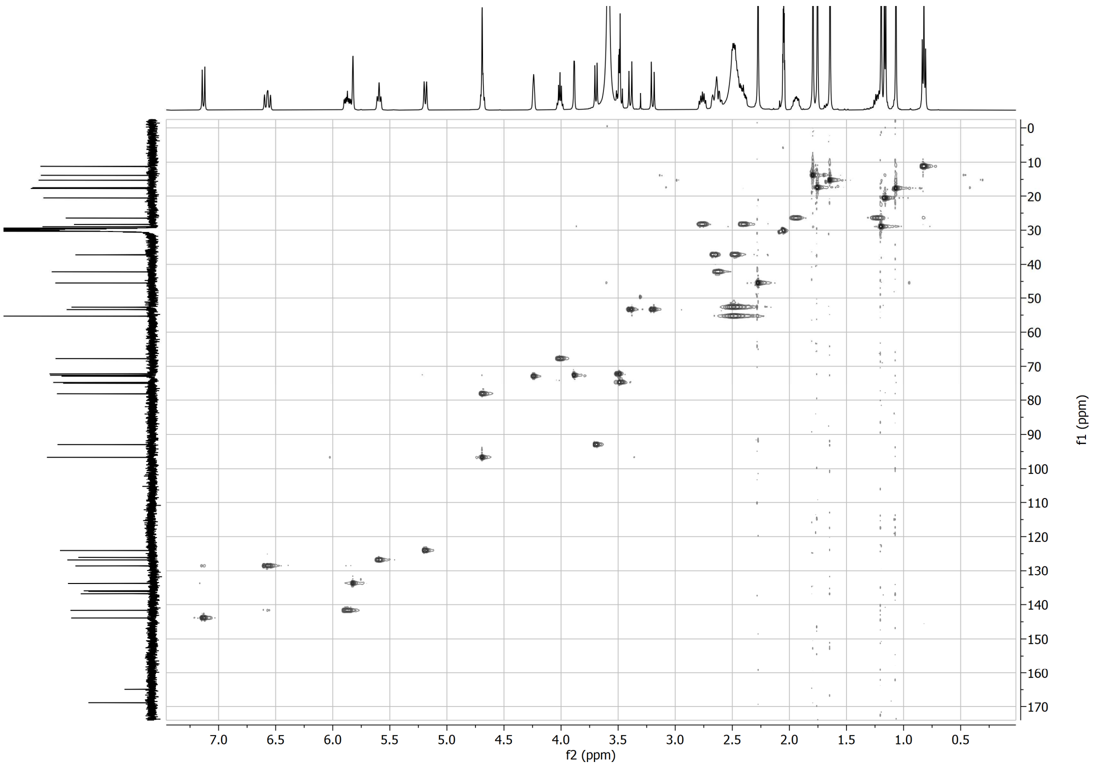

HMBC spectrum of **40** (500 MHz 126 MHz, acetone-d_6)

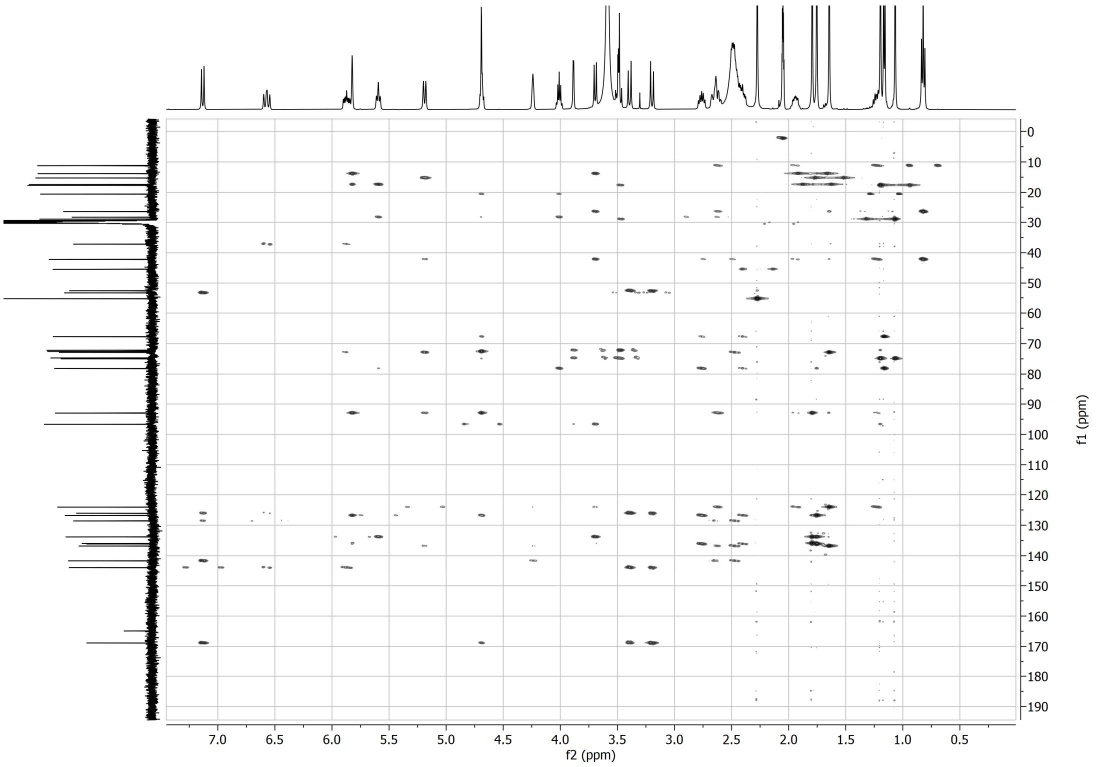

^{1}H NMR spectrum of **40i** (500 MHz, acetone-d_6)

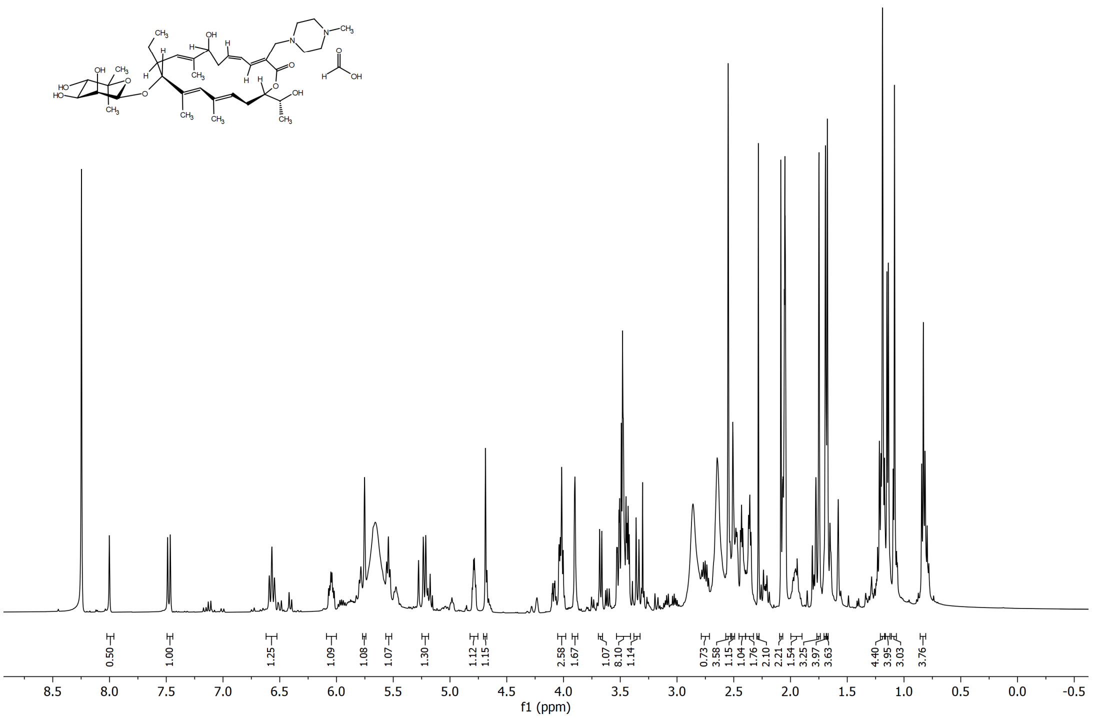

^{13}C NMR spectrum of **40i** (126 MHz, acetone-d_6)

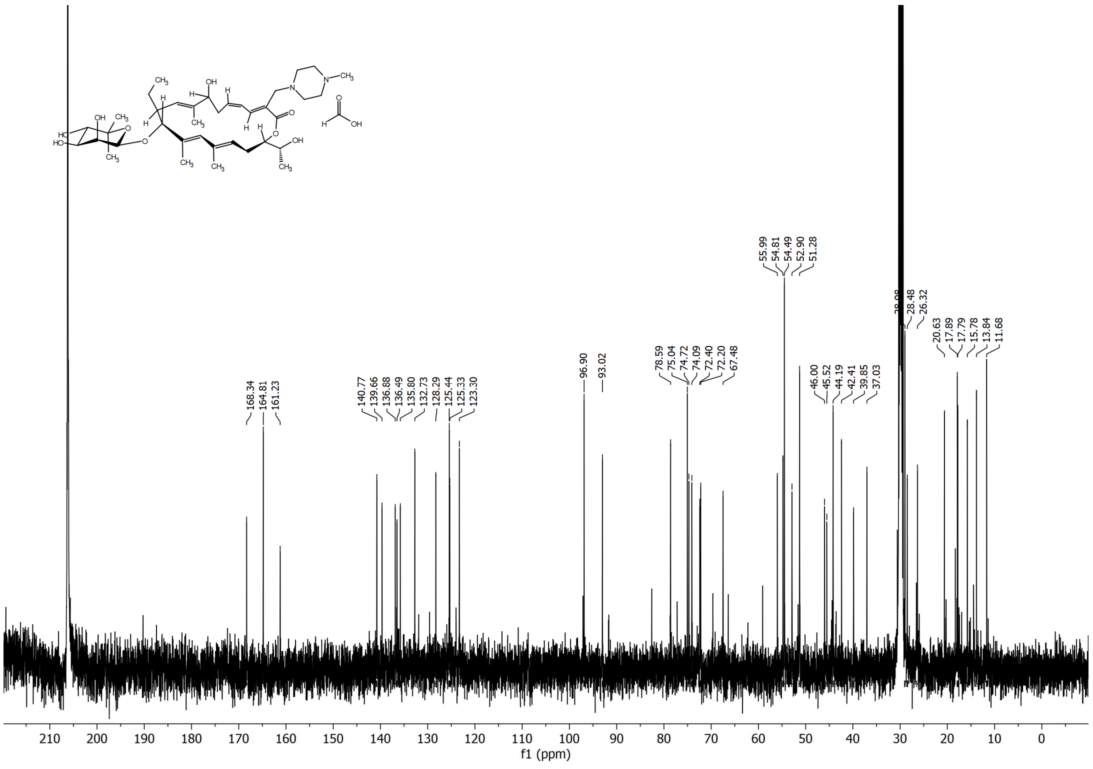

COSY spectrum of **40i** (500 MHz, acetone-d_6)

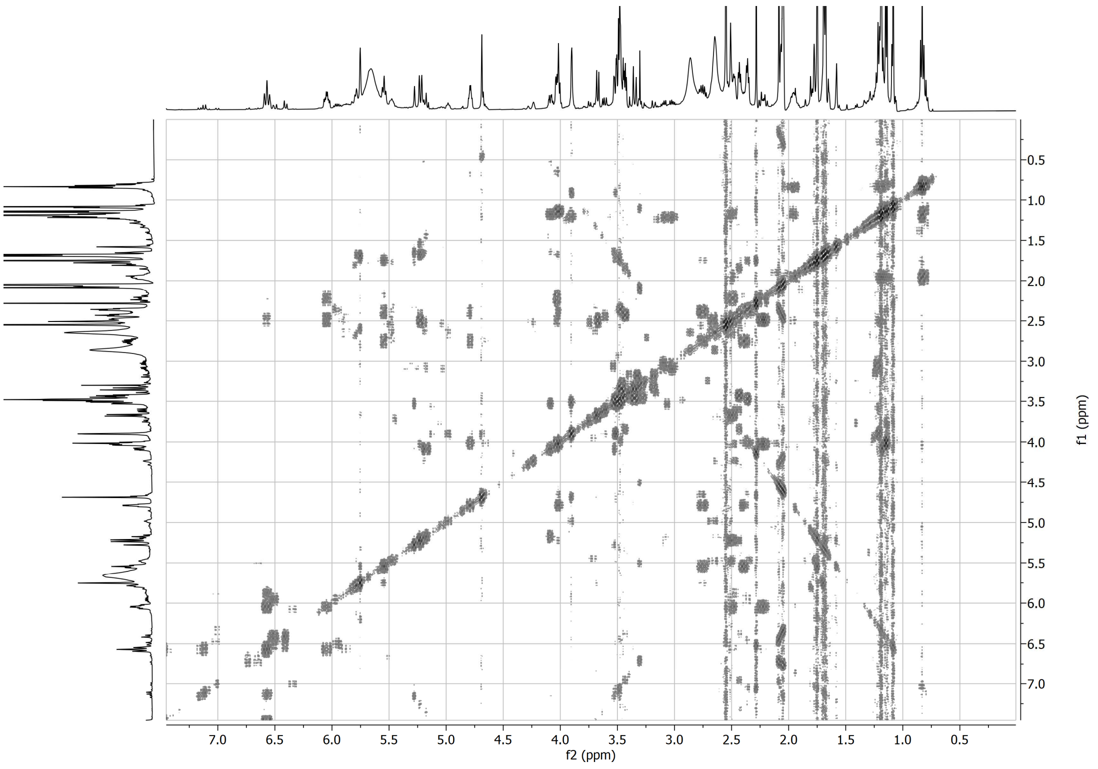

TOCSY spectrum of **40i** (500 MHz, acetone-d_6)

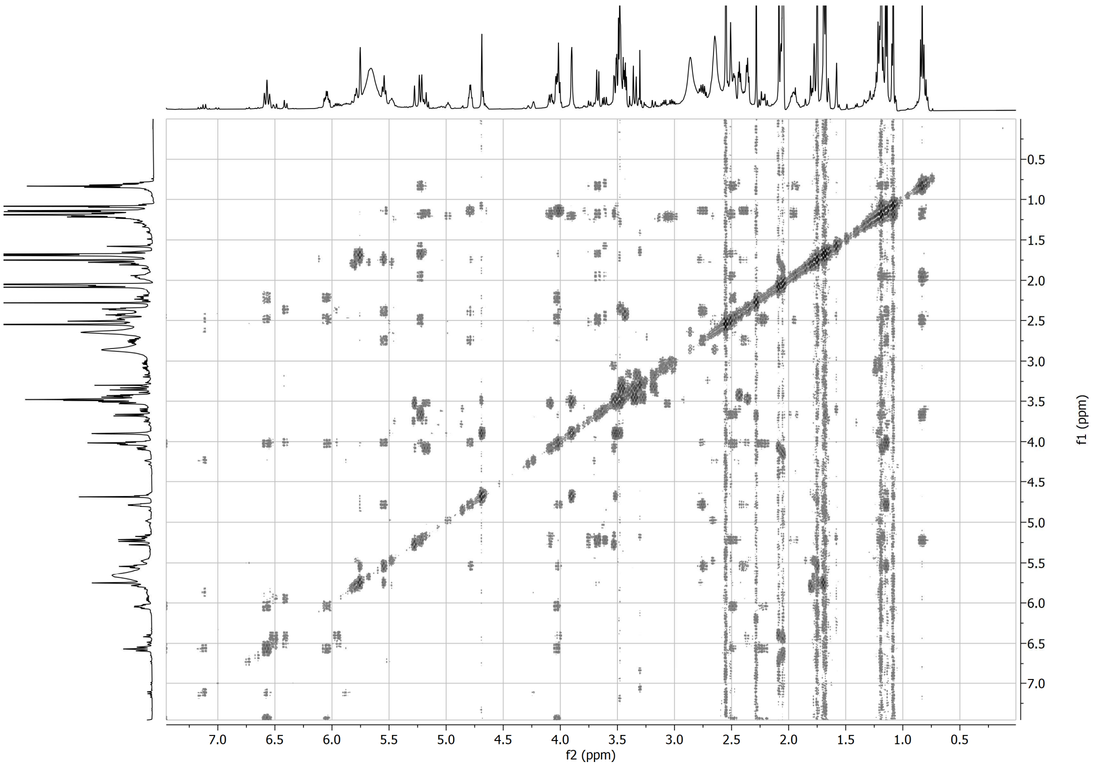

HSQC spectrum of **40i** (500 MHz 126 MHz, acetone-d_6)

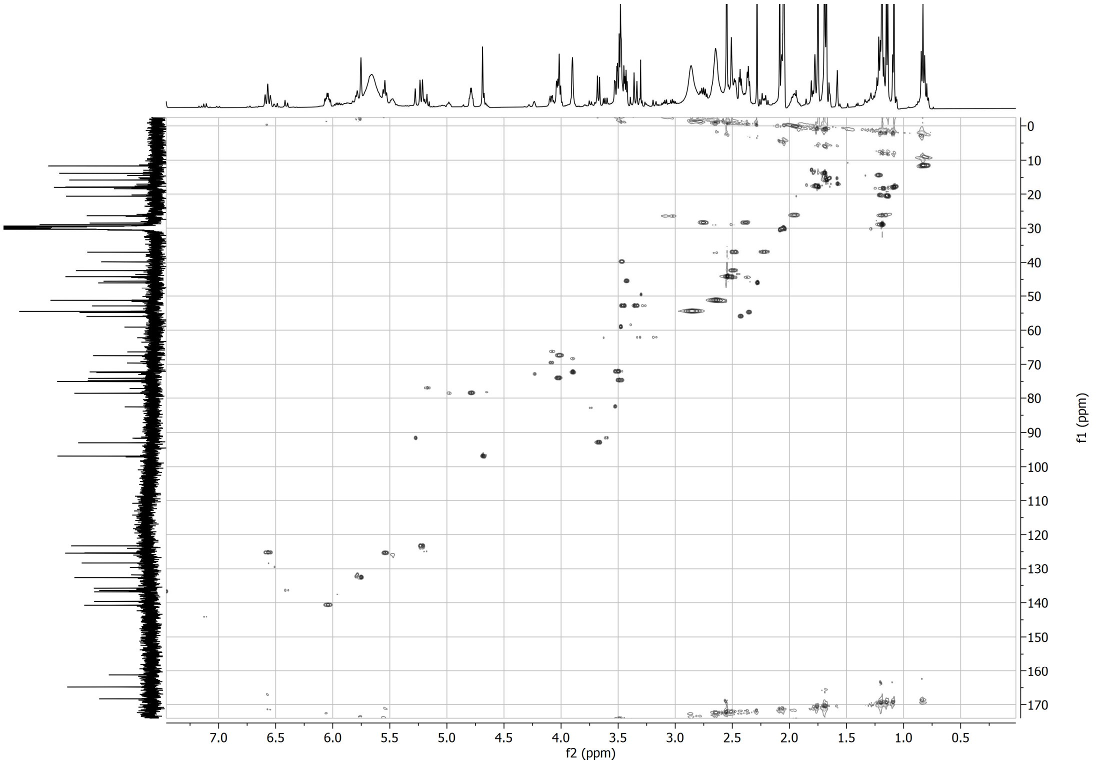

HMBC spectrum of **40i** (500 MHz 126 MHz, acetone-d_6)

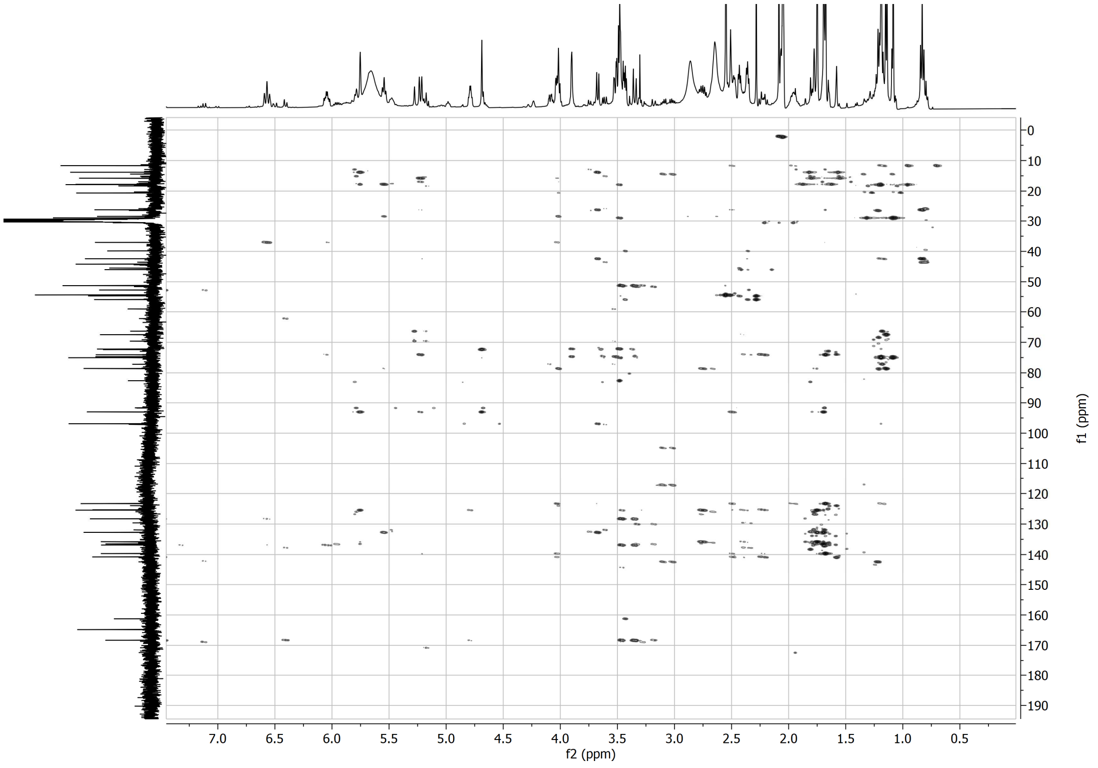

^{1}H **NMR** spectrum of **41** (500 MHz, acetone-d_6)

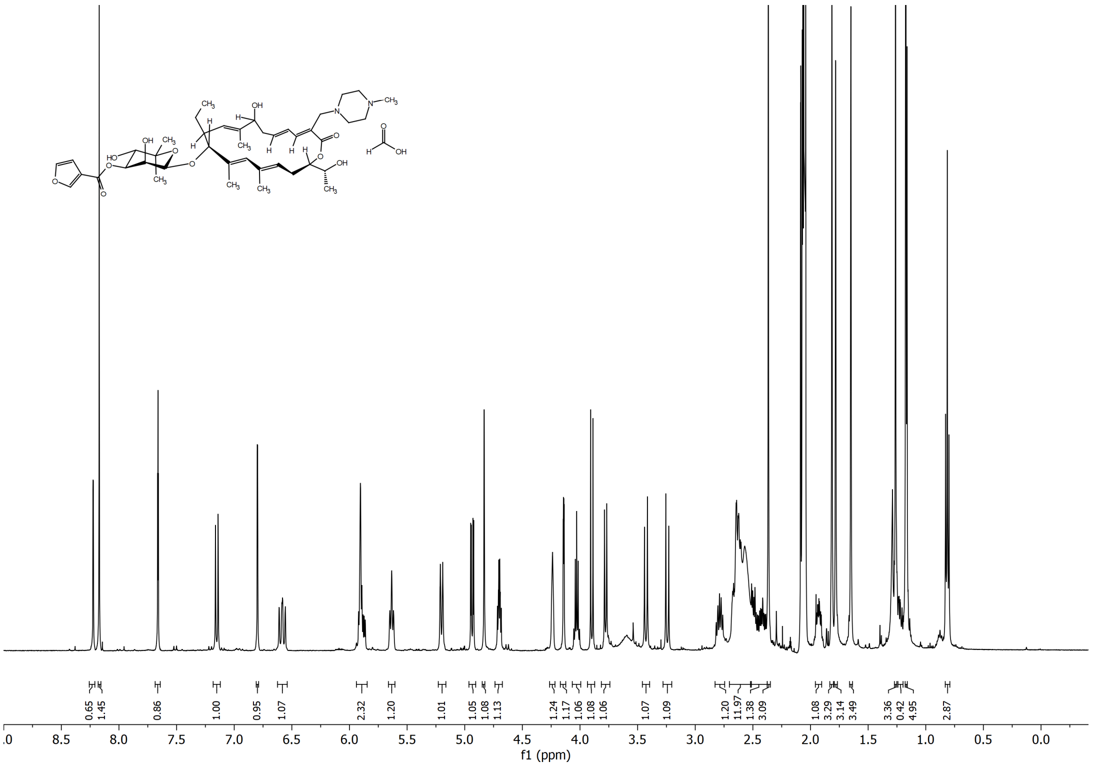

^{13}C **NMR** spectrum of **41** (101 MHz, acetone-d_6)

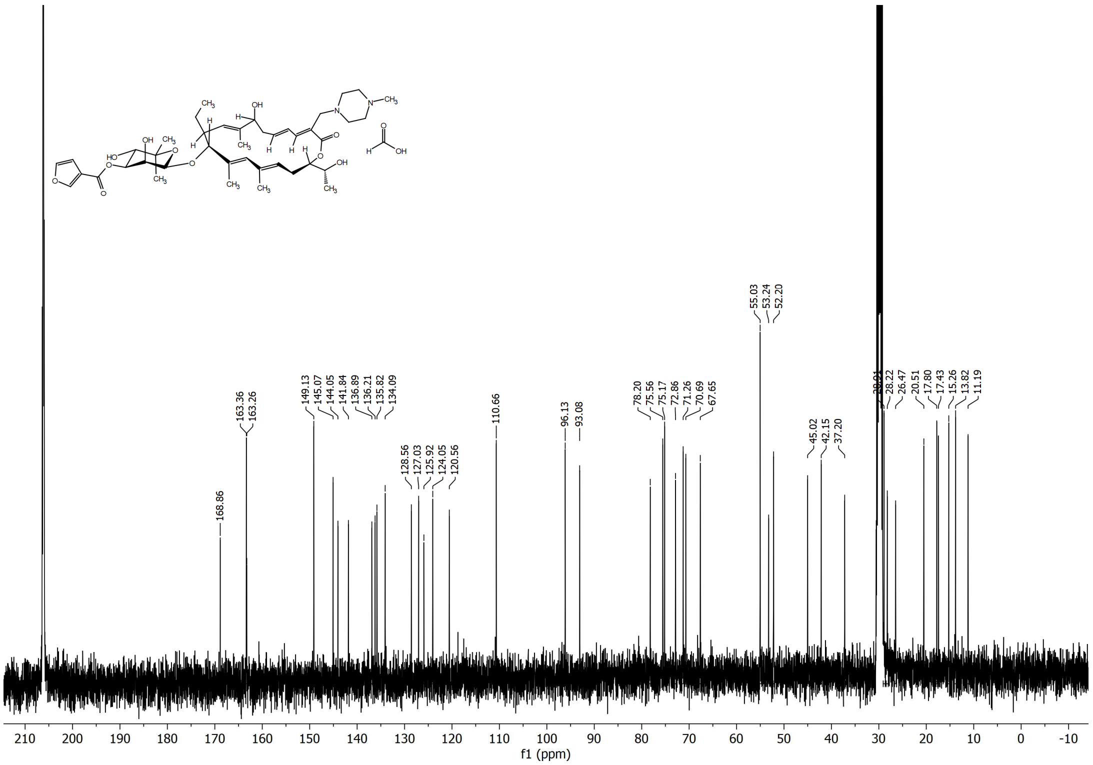

COSY spectrum of **41** (500 MHz, acetone-d_6)

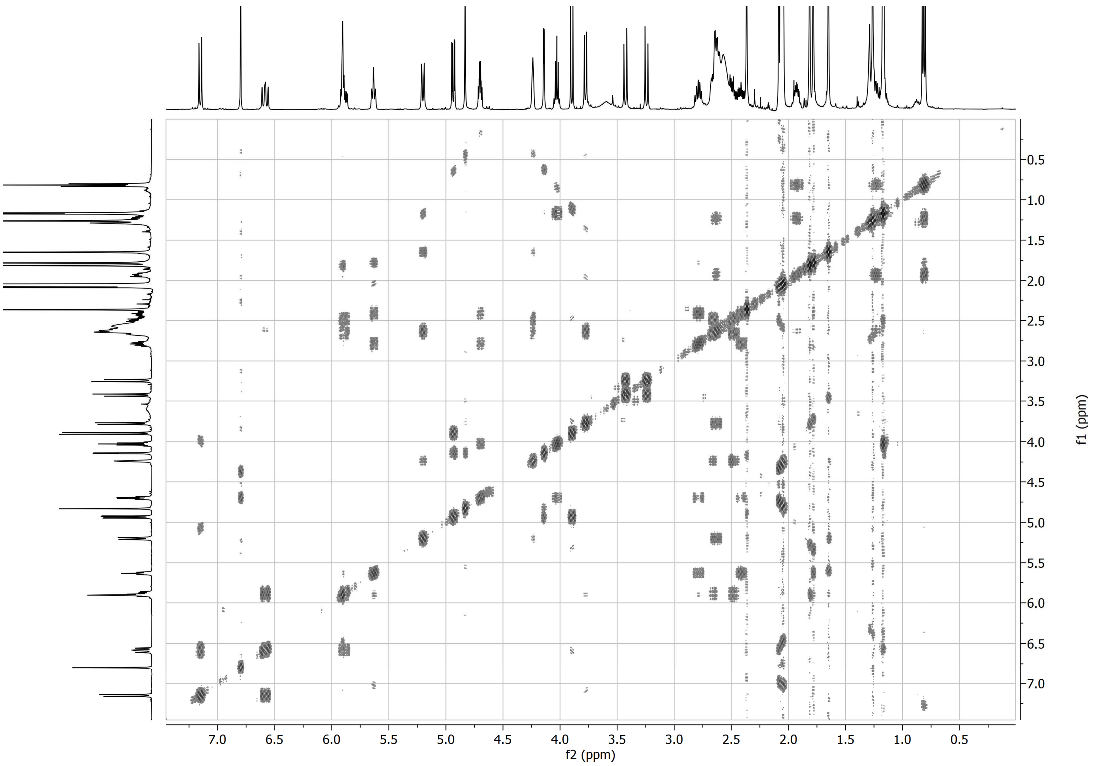

TOCSY spectrum of **41** (500 MHz, acetone-d_6)

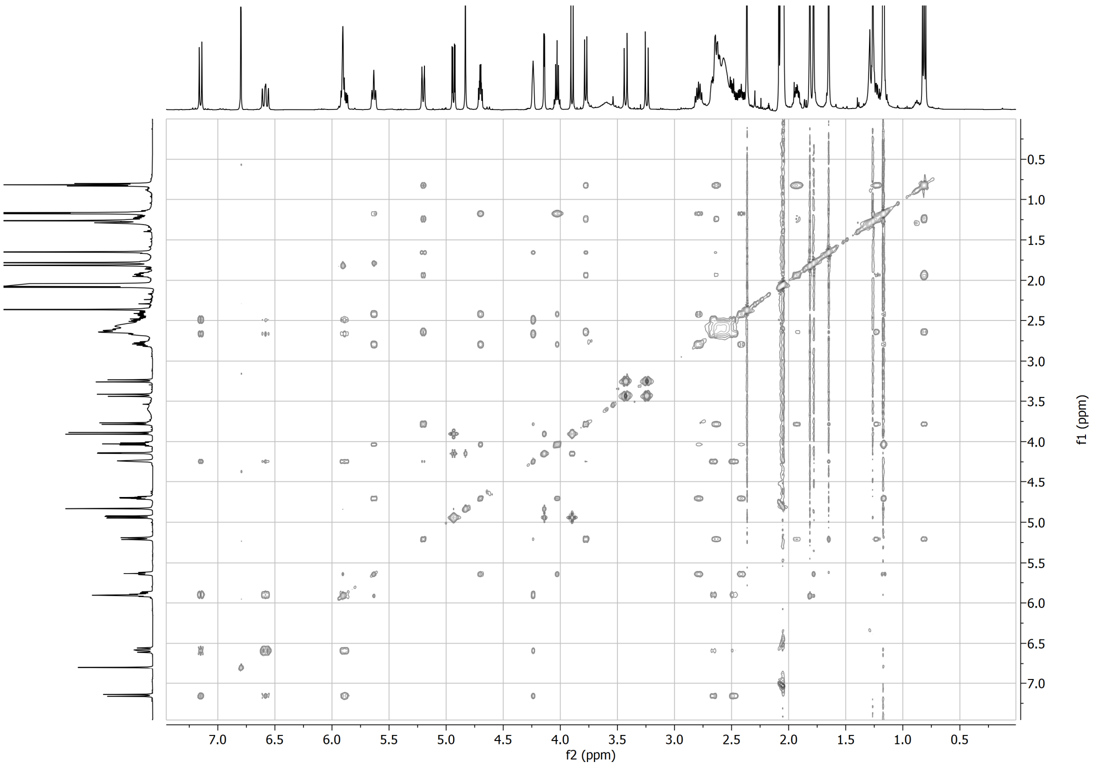

HSQC spectrum of **41** (500 MHz 126 MHz, acetone-d_6)

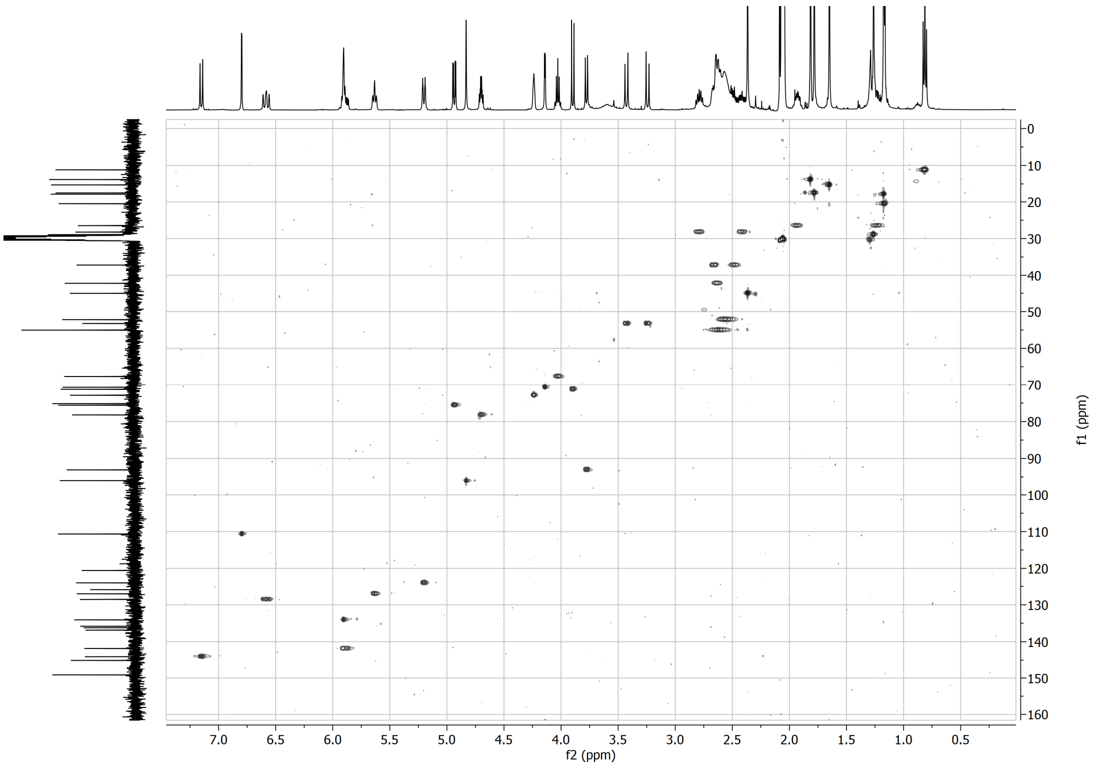

HMBC spectrum of **41** (500 MHz 126 MHz, acetone-d_6)

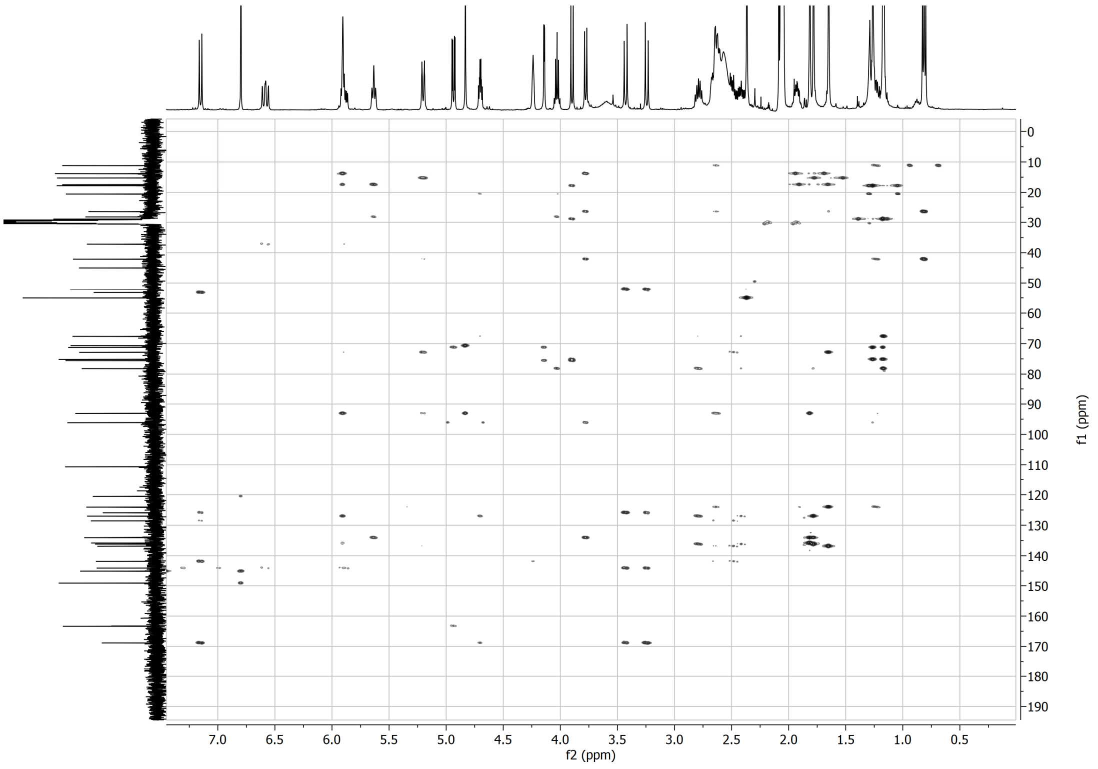

^{1}H NMR spectrum of **42** (500 MHz, acetone-d_6)

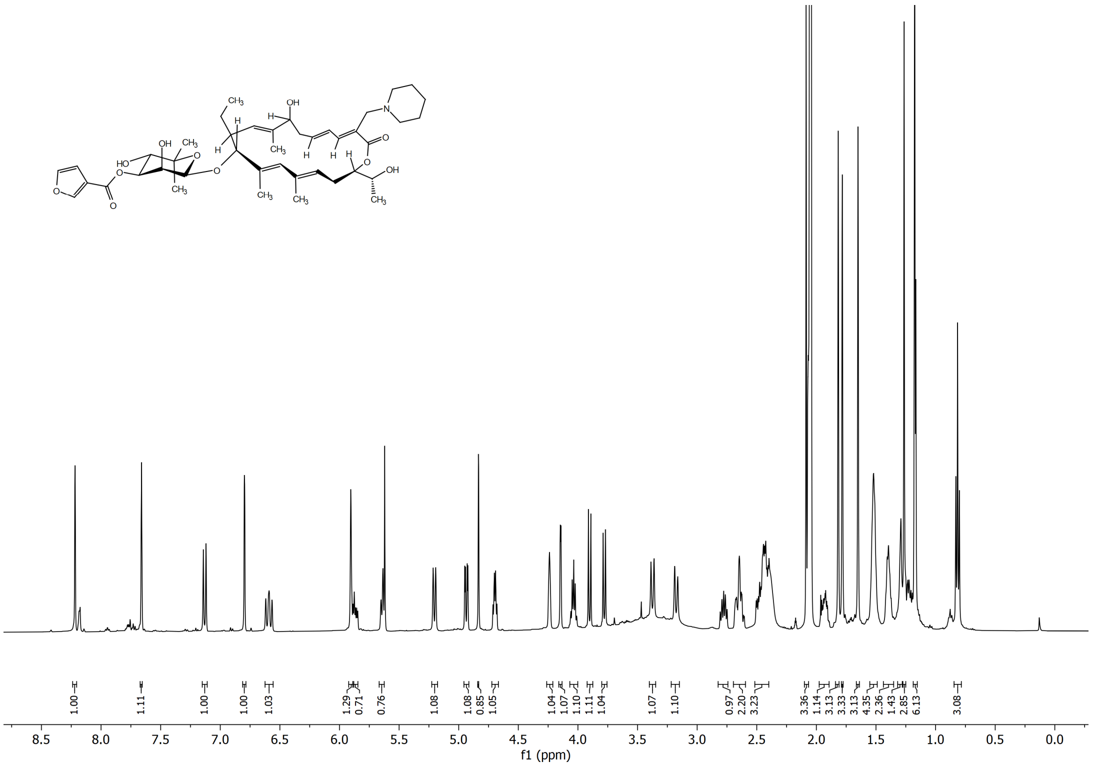

^{13}C NMR spectrum of **42** (126 MHz, acetone-d_6)

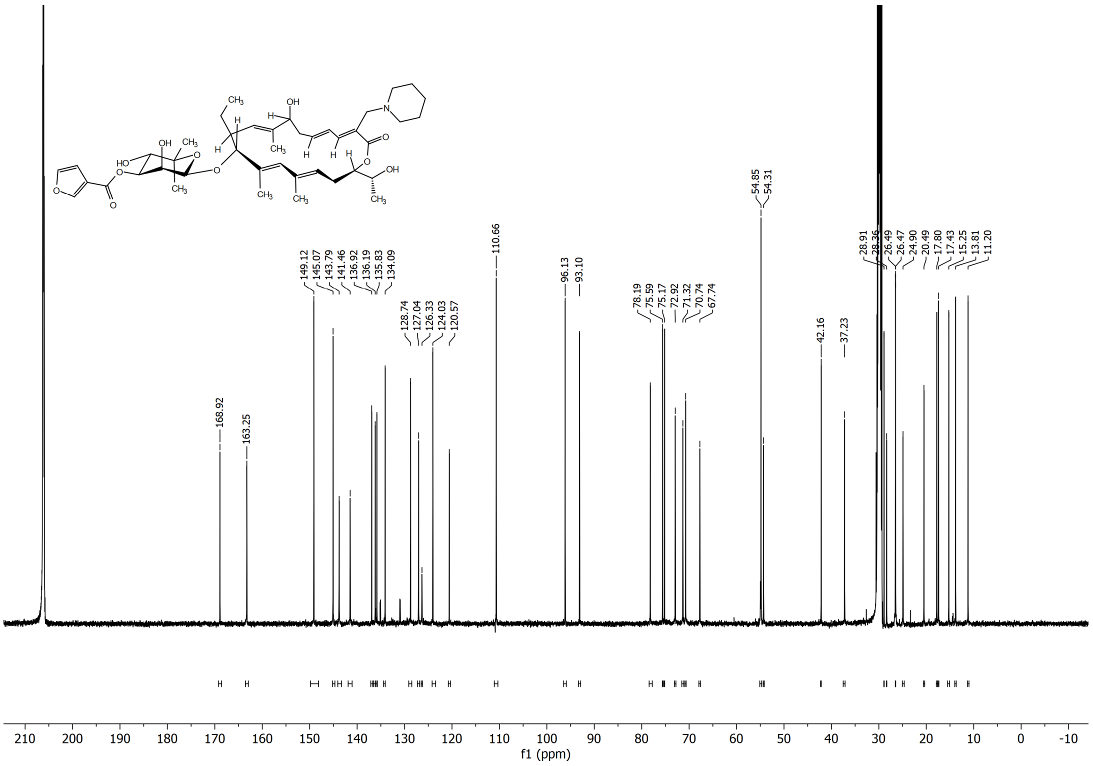

^{1}H NMR spectrum of **43** (500 MHz, acetone-d_6)

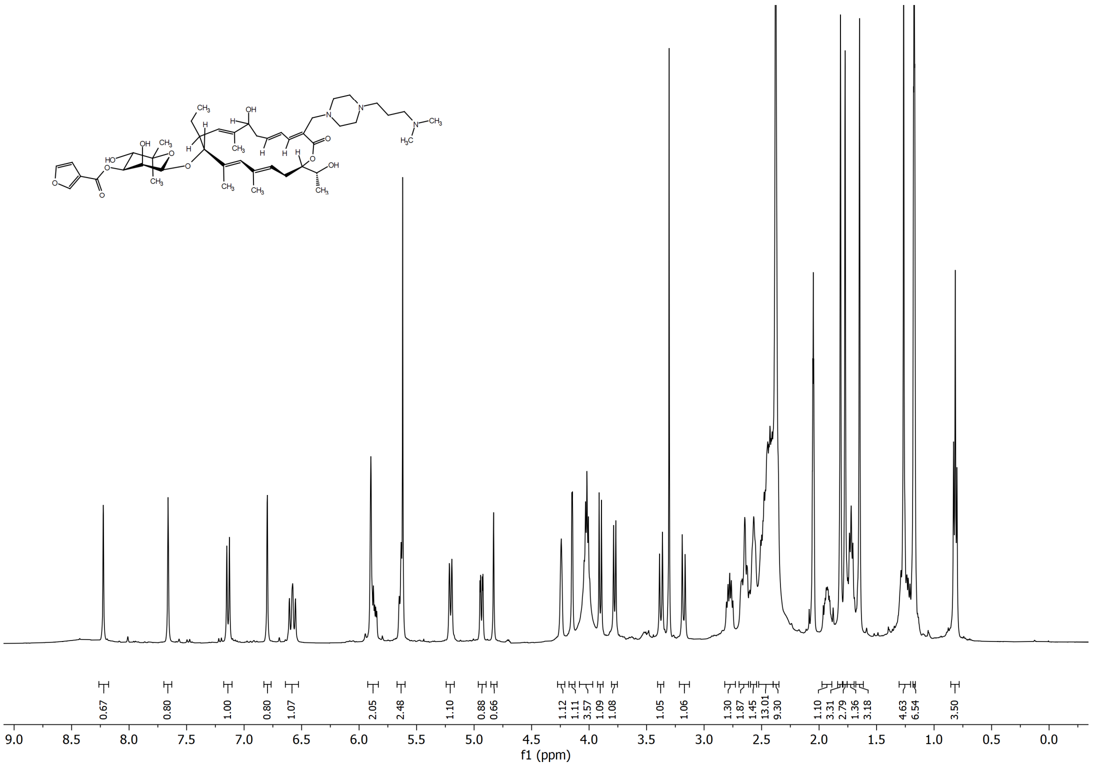

^{13}C NMR spectrum of **43** (126 MHz, acetone-d_6)

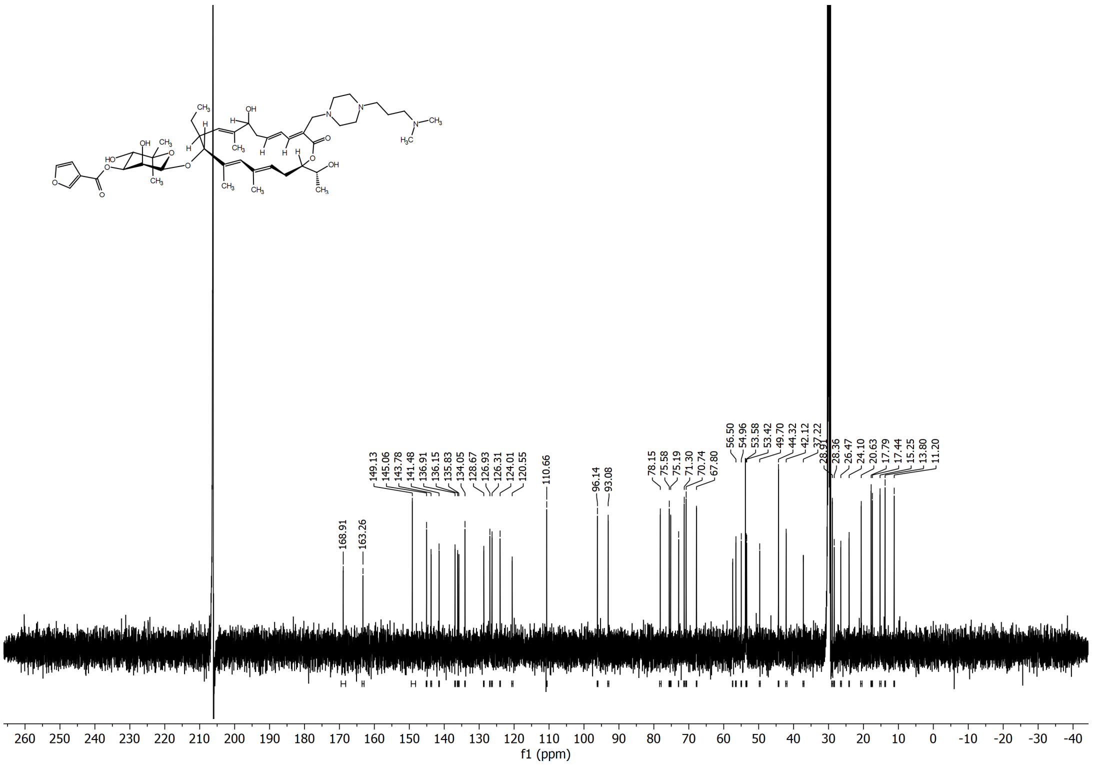

COSY spectrum of **43** (500 MHz, acetone-d_6)

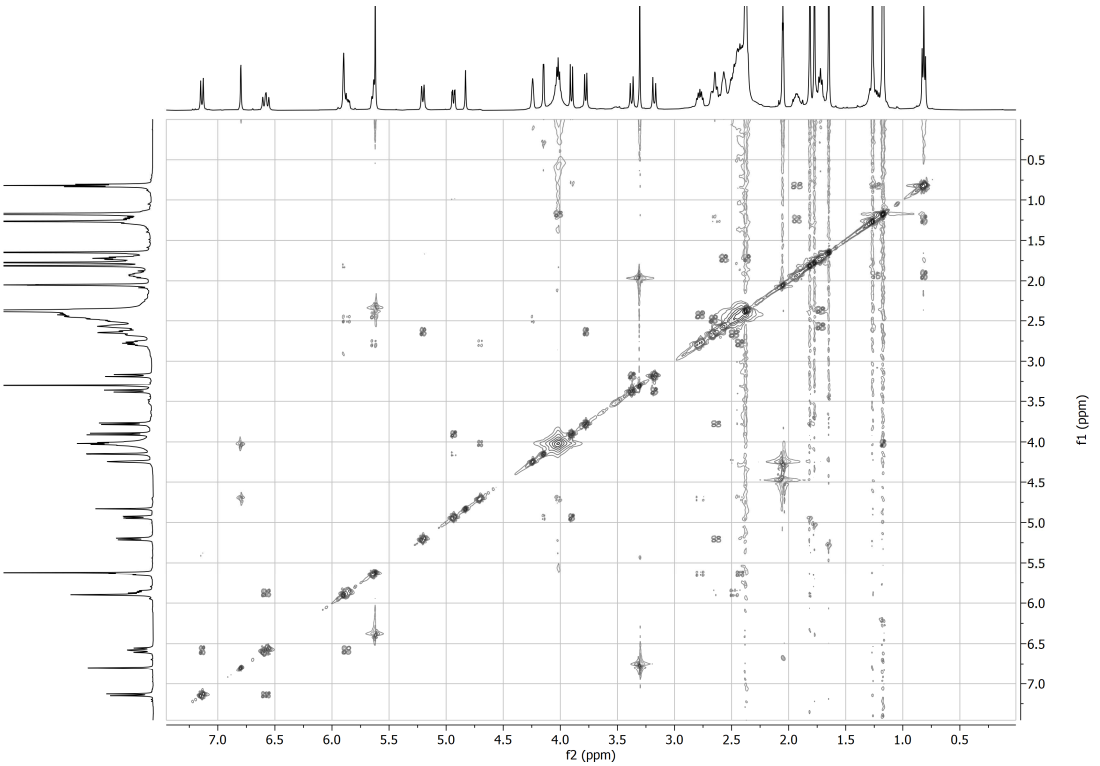

TOCSY spectrum of **43** (500 MHz, acetone-d_6)

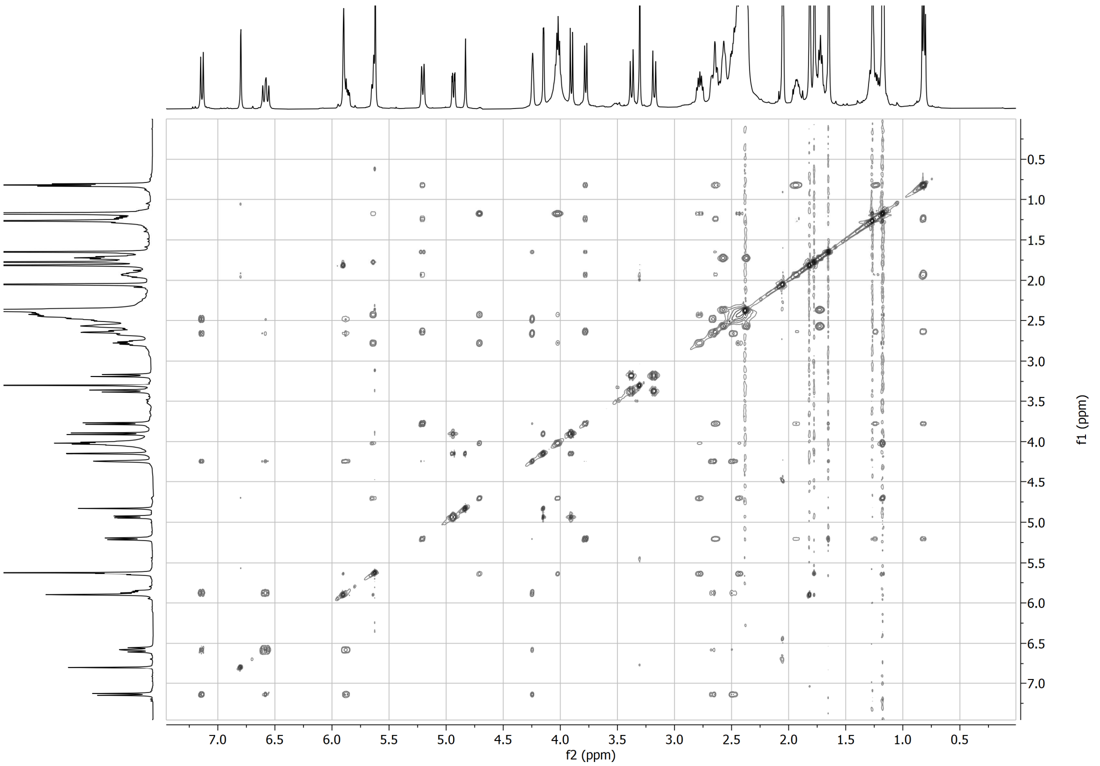

HSQC spectrum of **43** (500 MHz 126 MHz, acetone-d_6)

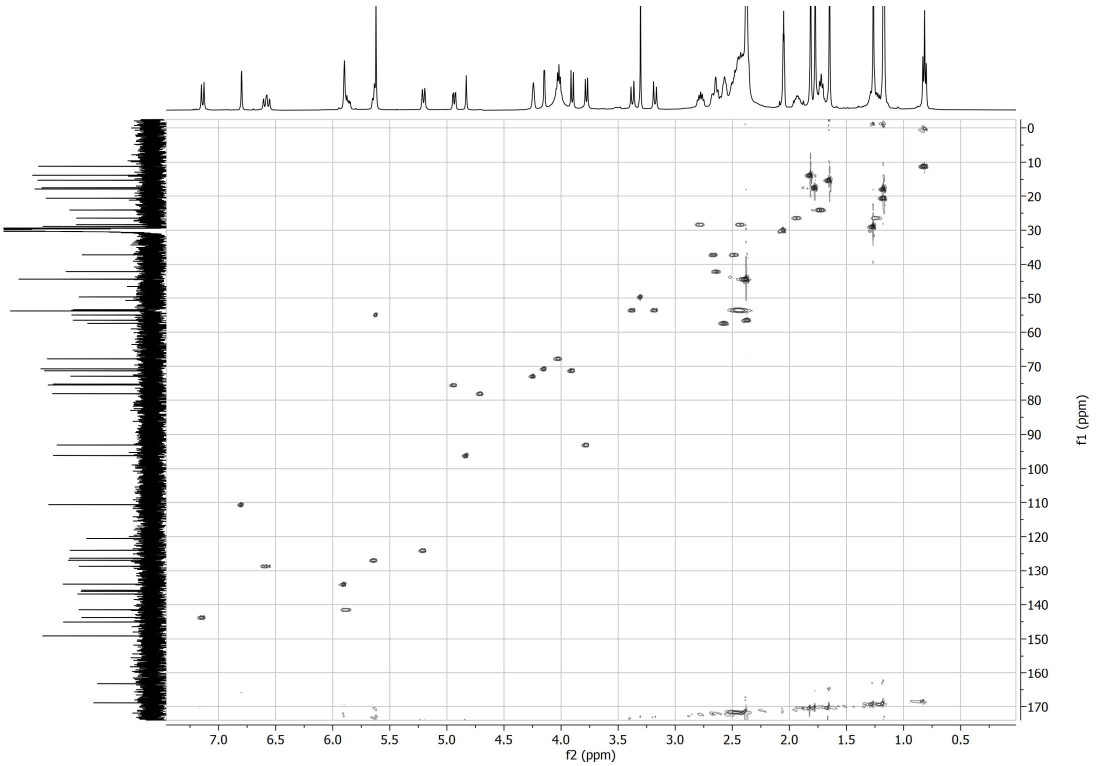

HMBC spectrum of **43** (500 MHz 126 MHz, acetone-d_6)

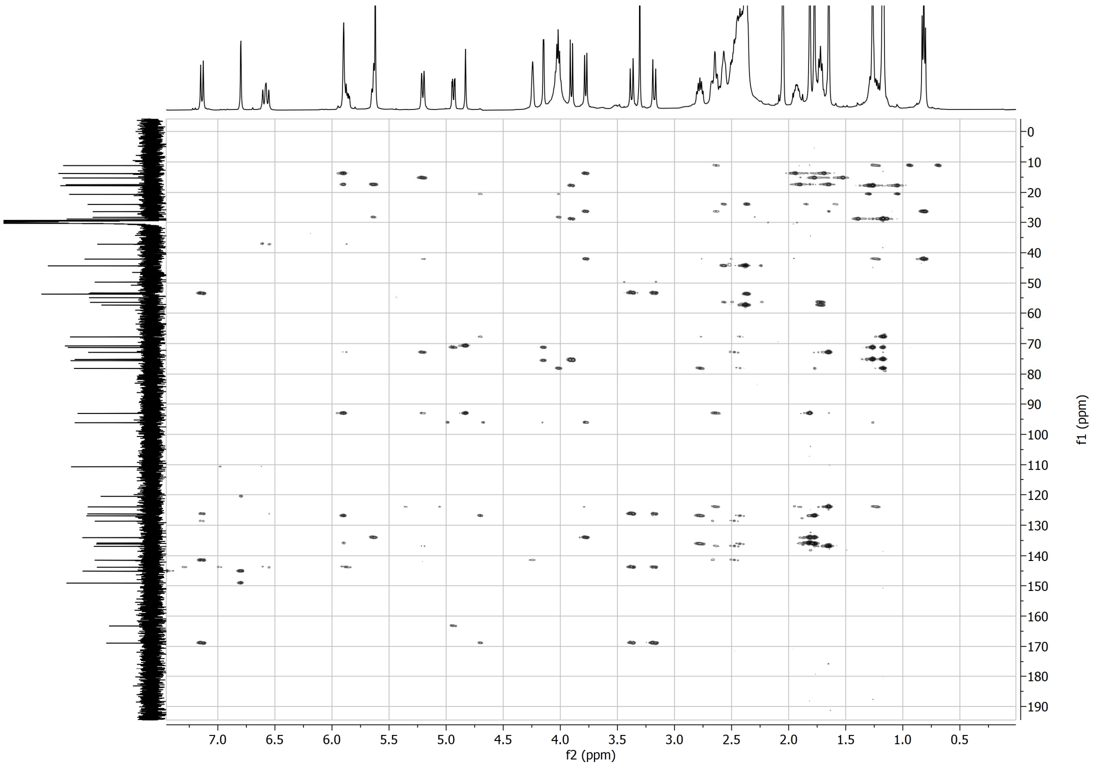

^{1}H NMR spectrum of **44** (500 MHz, acetone-d_6)

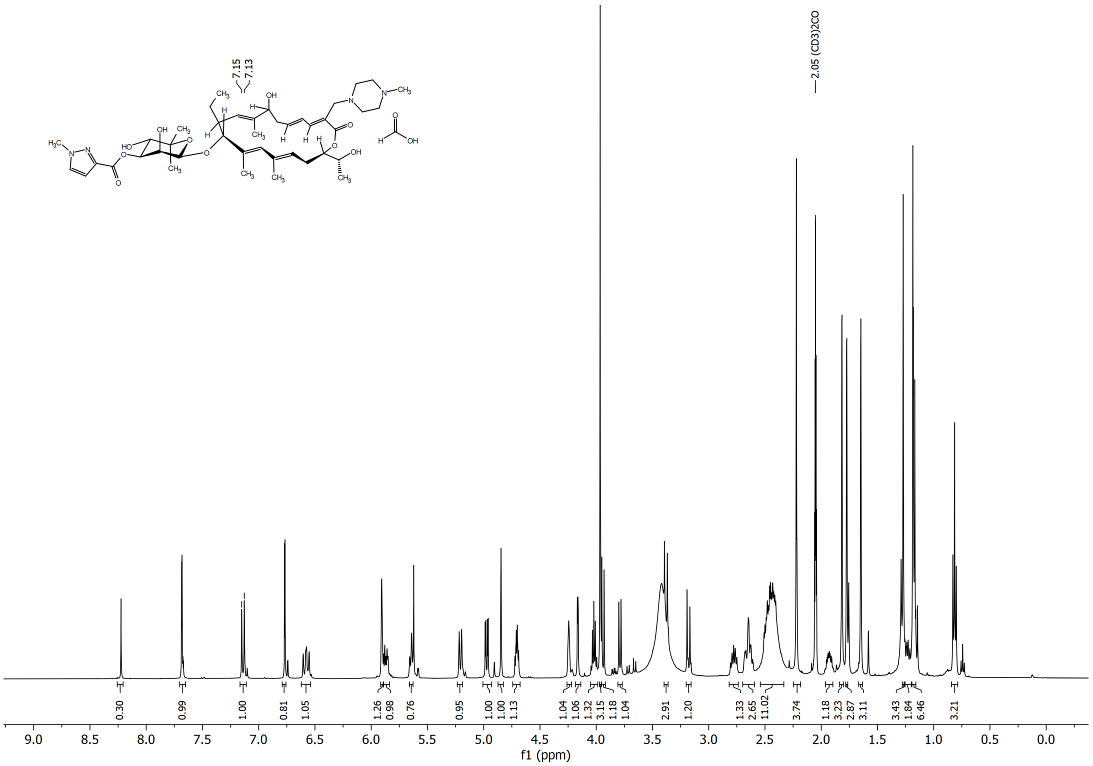

^{13}C NMR spectrum of **44** (126 MHz, acetone-d_6)

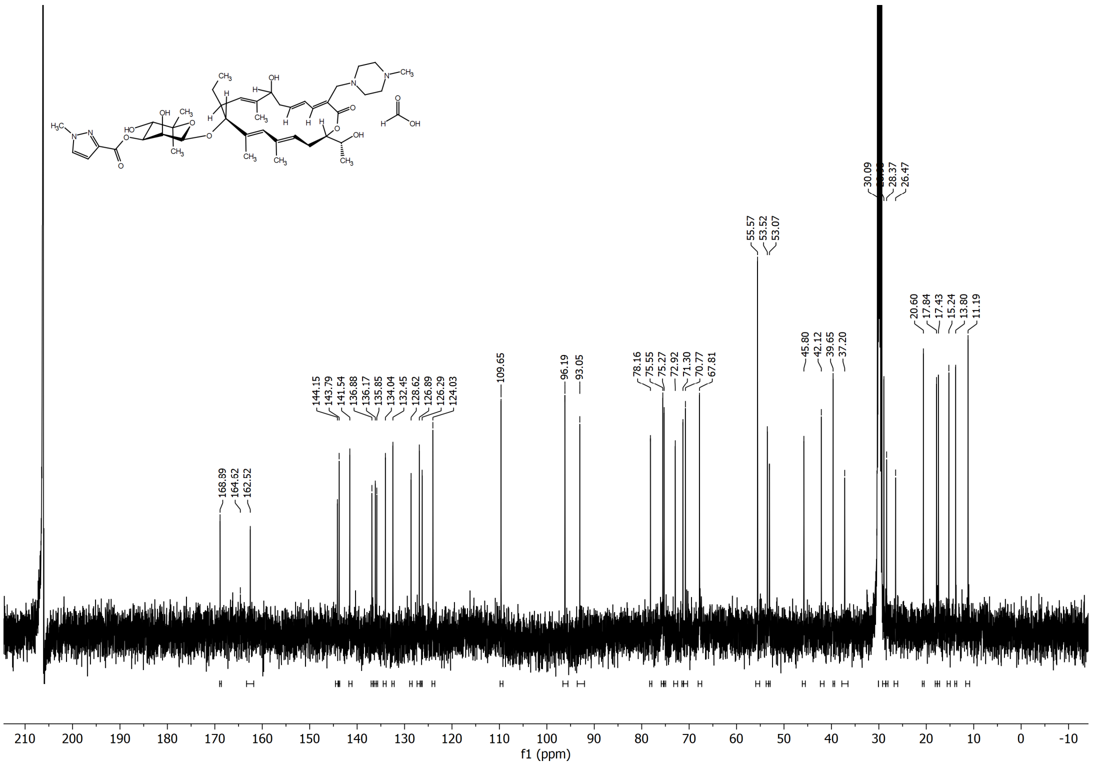

COSY spectrum of **44** (500 MHz, acetone-d_6)

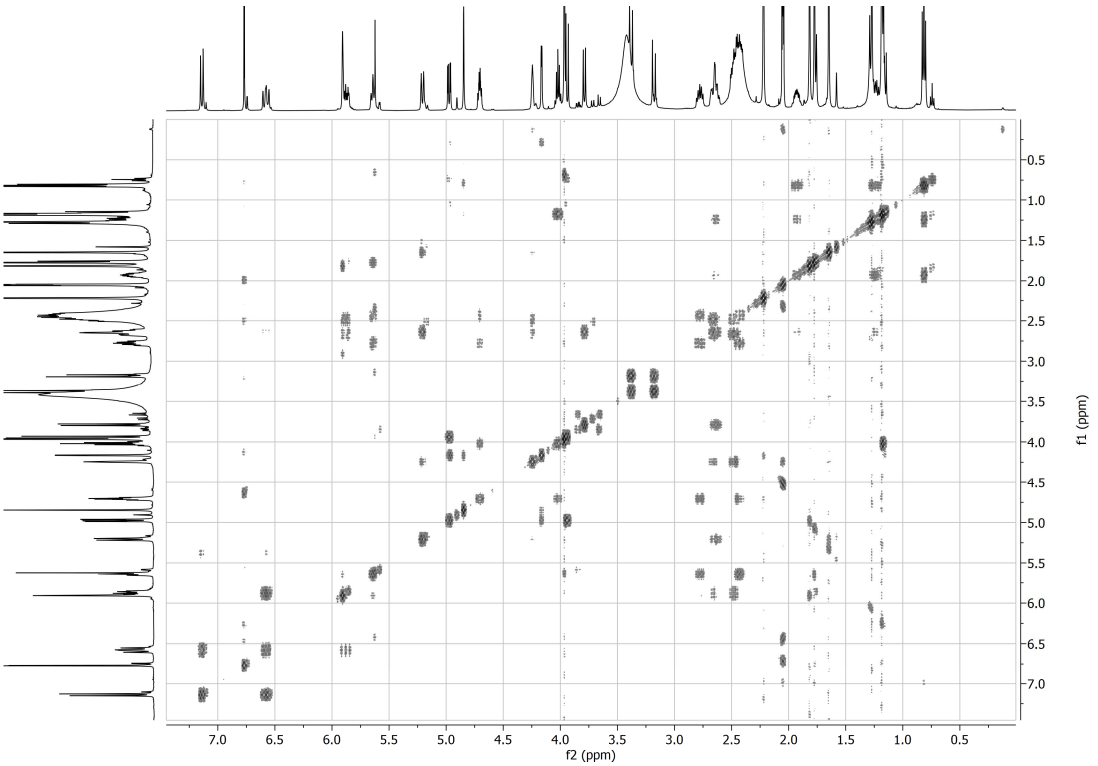

TOCSY spectrum of **44** (500 MHz, acetone-d_6)

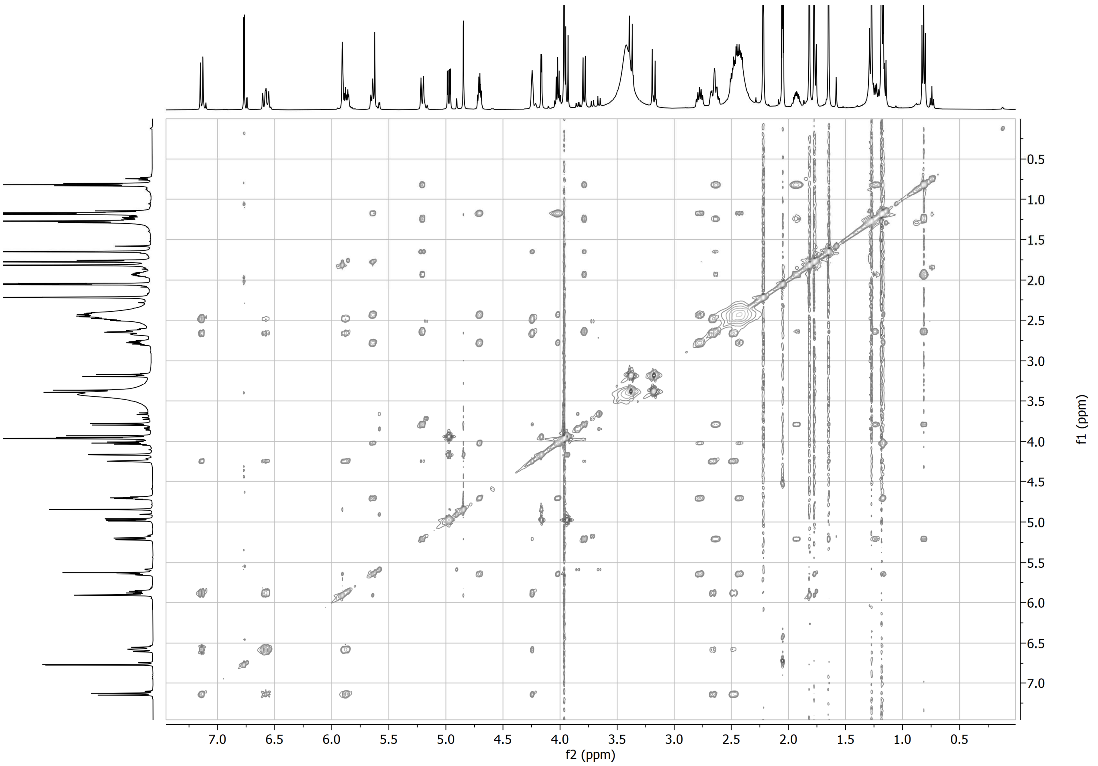

HSQC spectrum of **44** (500 MHz 126 MHz, acetone-d_6)

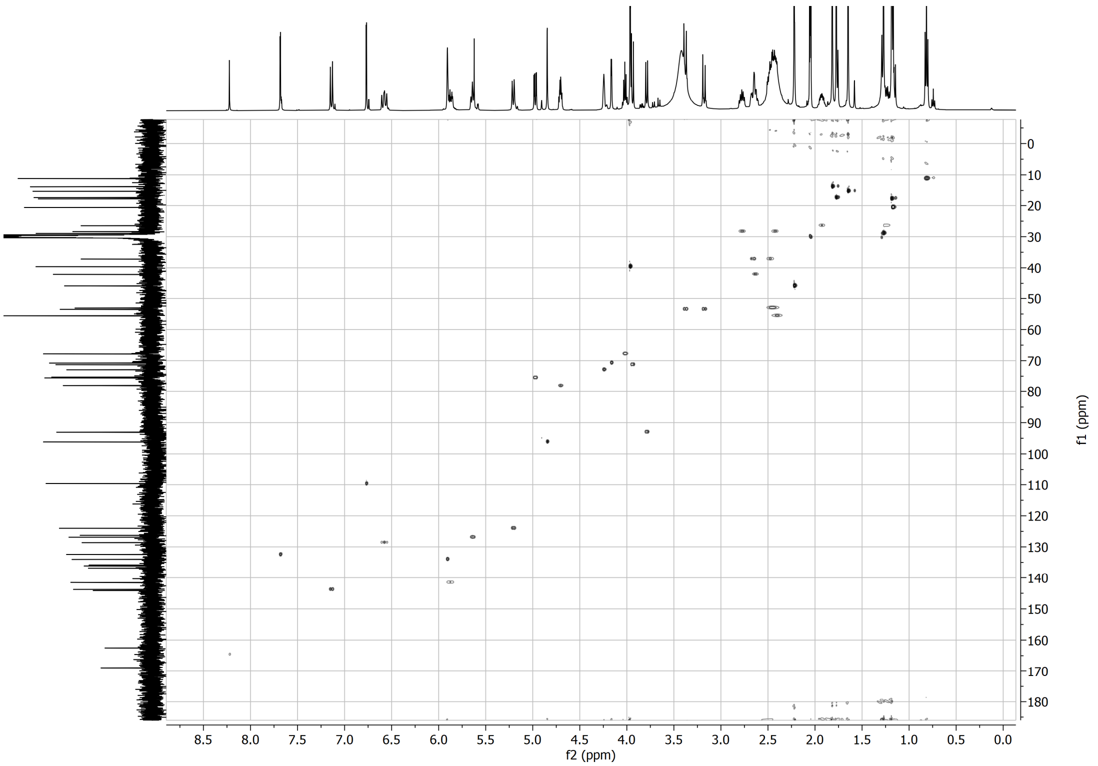

HMBC spectrum of **44** (500 MHz 126 MHz, acetone-d_6)

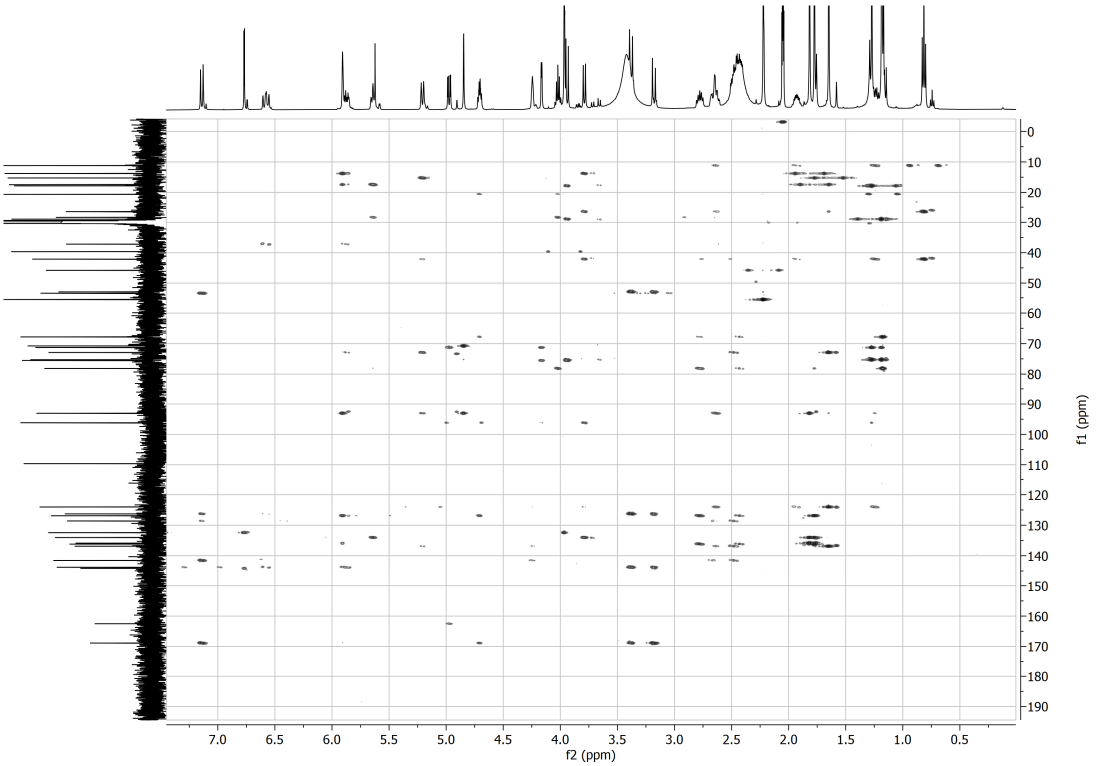

^{1}H NMR spectrum of **45** (500 MHz, acetone-d_6)

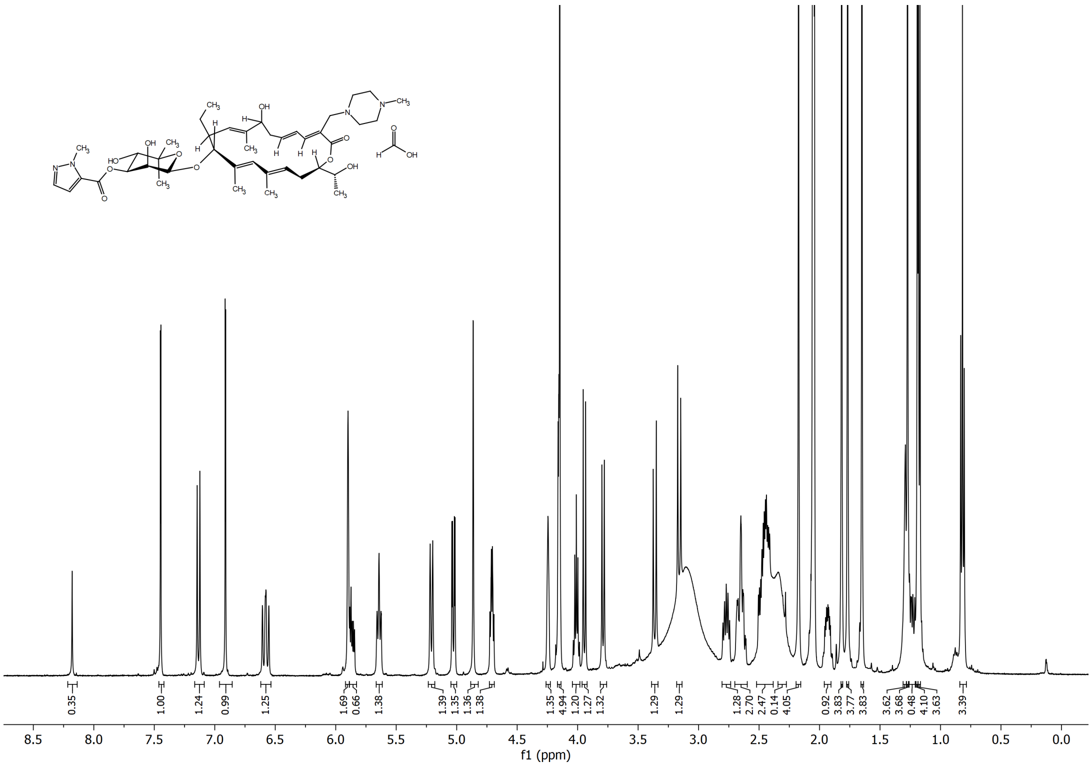

^{13}C NMR spectrum of **45** (126 MHz, acetone-d_6)

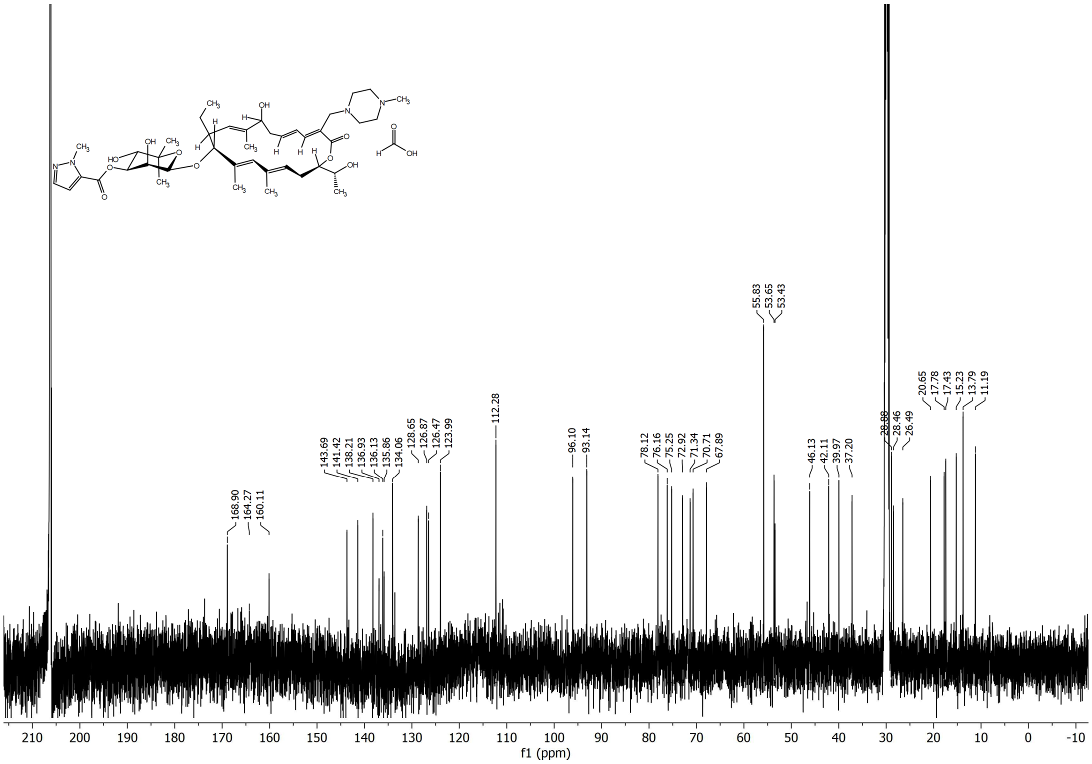

COSY spectrum of **45** (500 MHz, acetone-d_6)

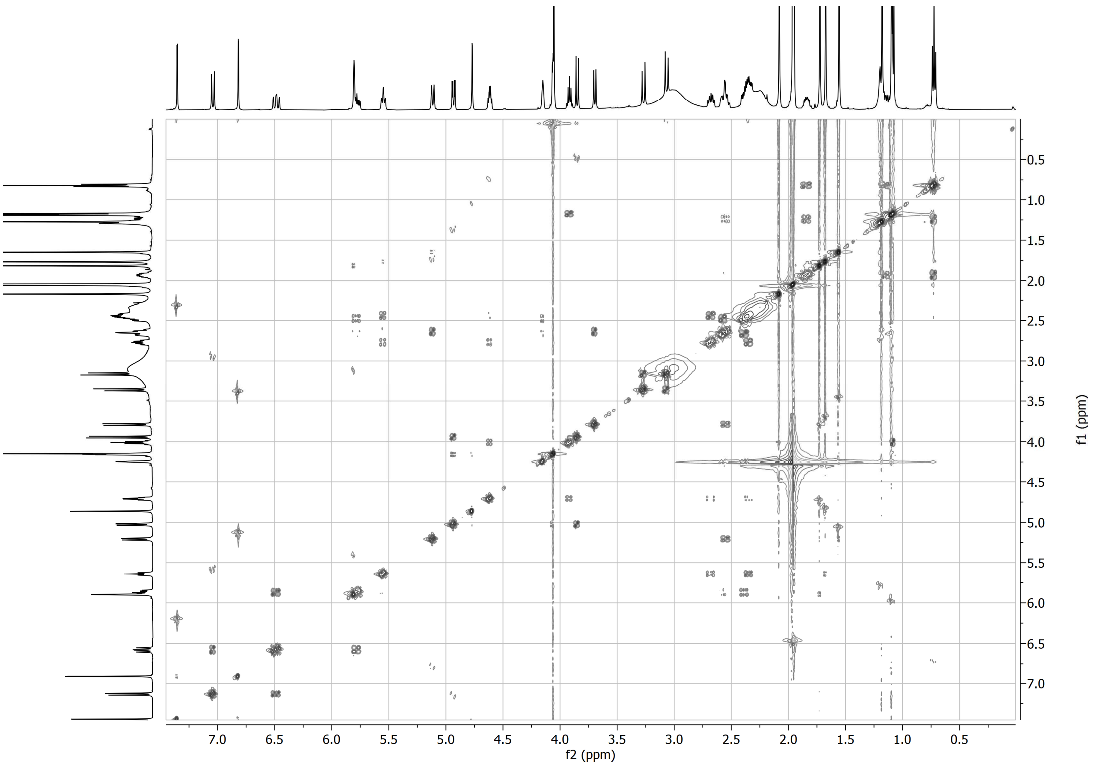

TOCSY spectrum of **45** (500 MHz, acetone-d_6)

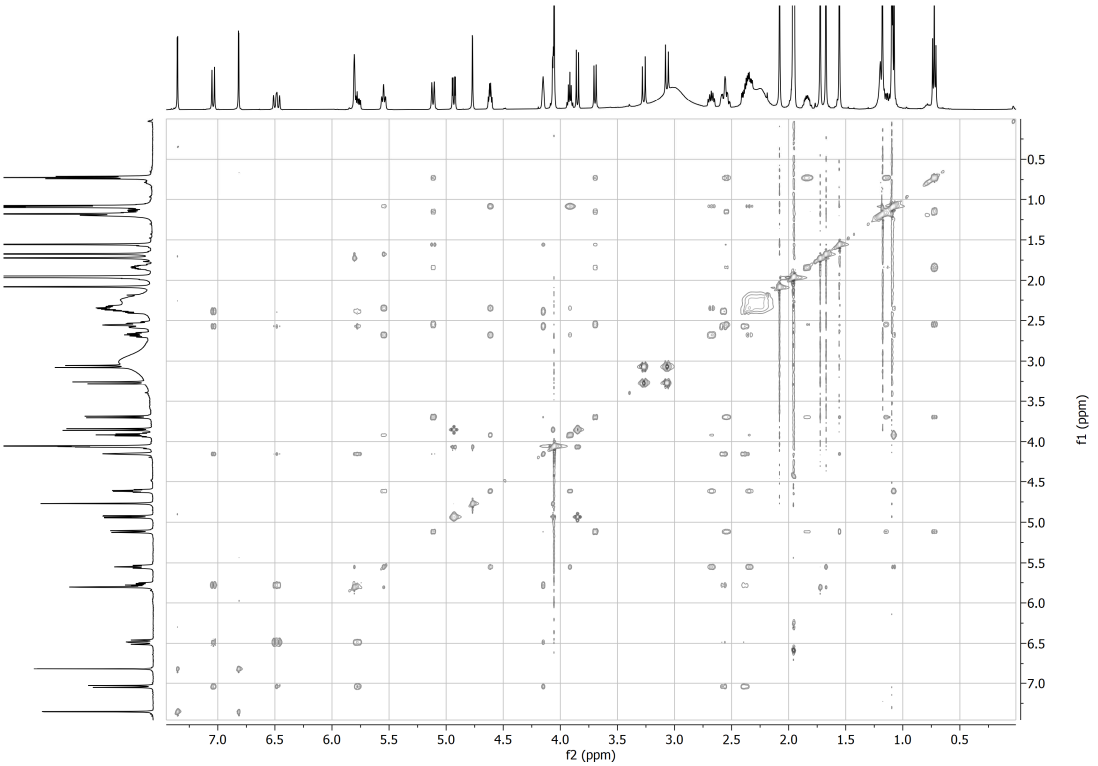

HSQC spectrum of **45** (500 MHz 126 MHz, acetone-d_6)

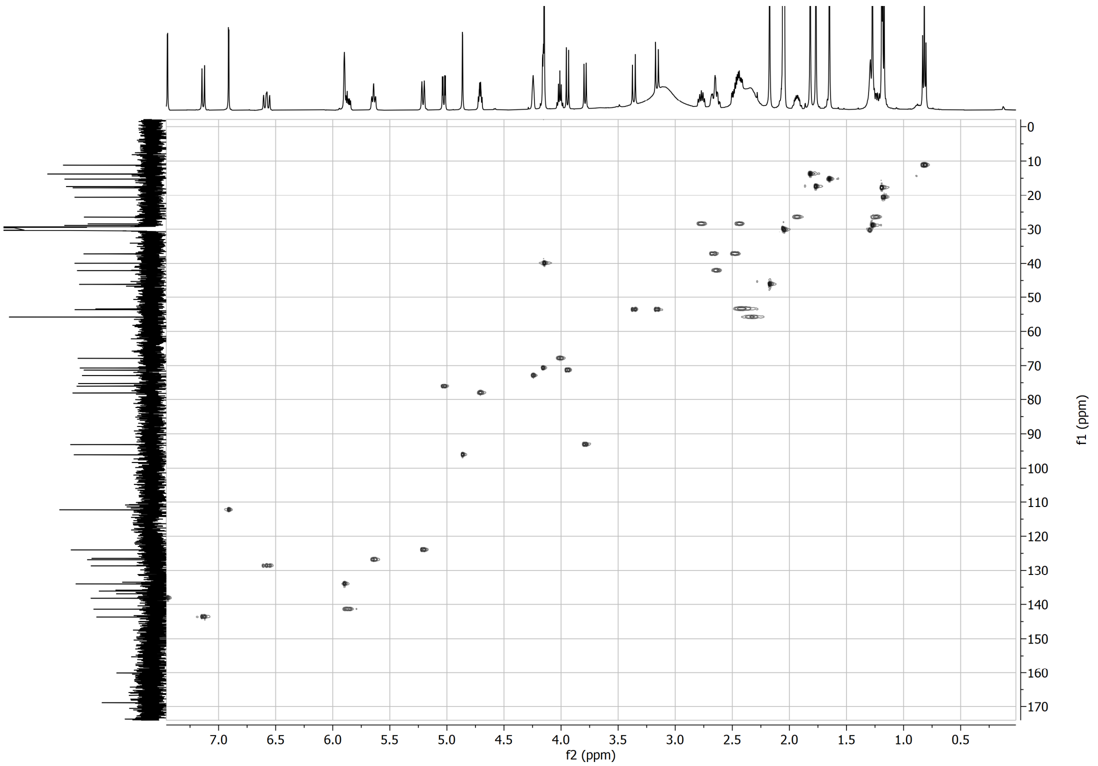

HMBC spectrum of **45** (500 MHz 126 MHz, acetone-d_6)

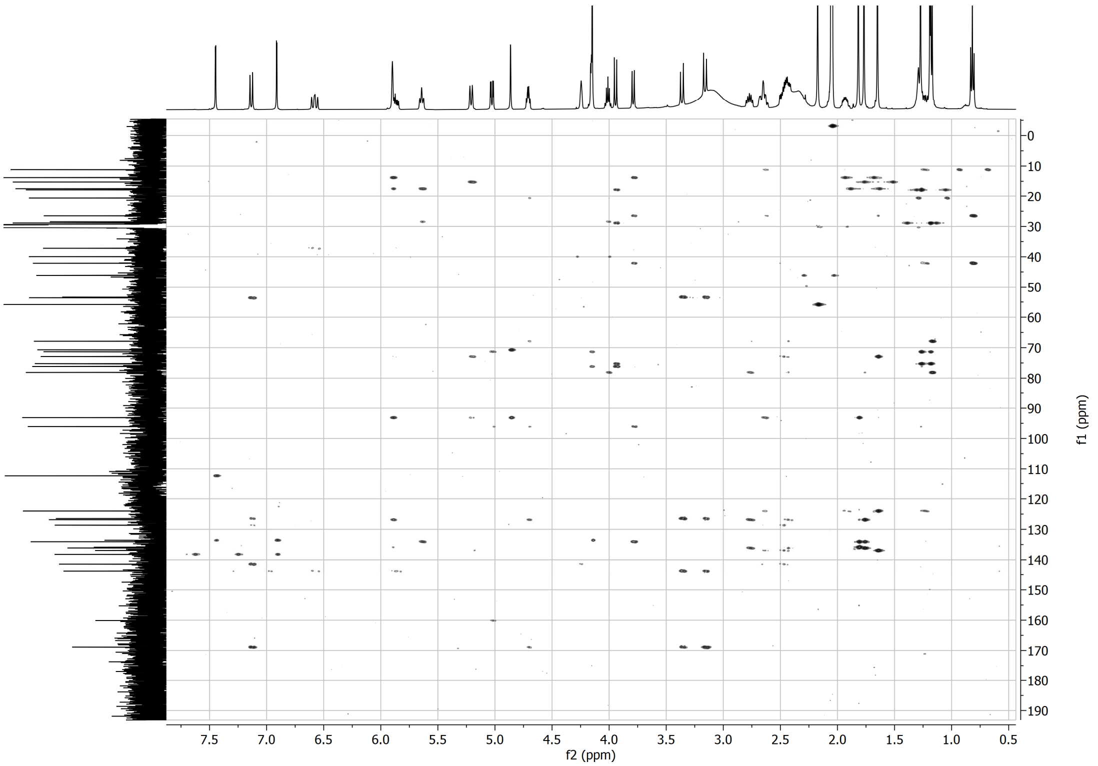

^{1}H NMR spectrum of **46** (500 MHz, acetone-d_6)

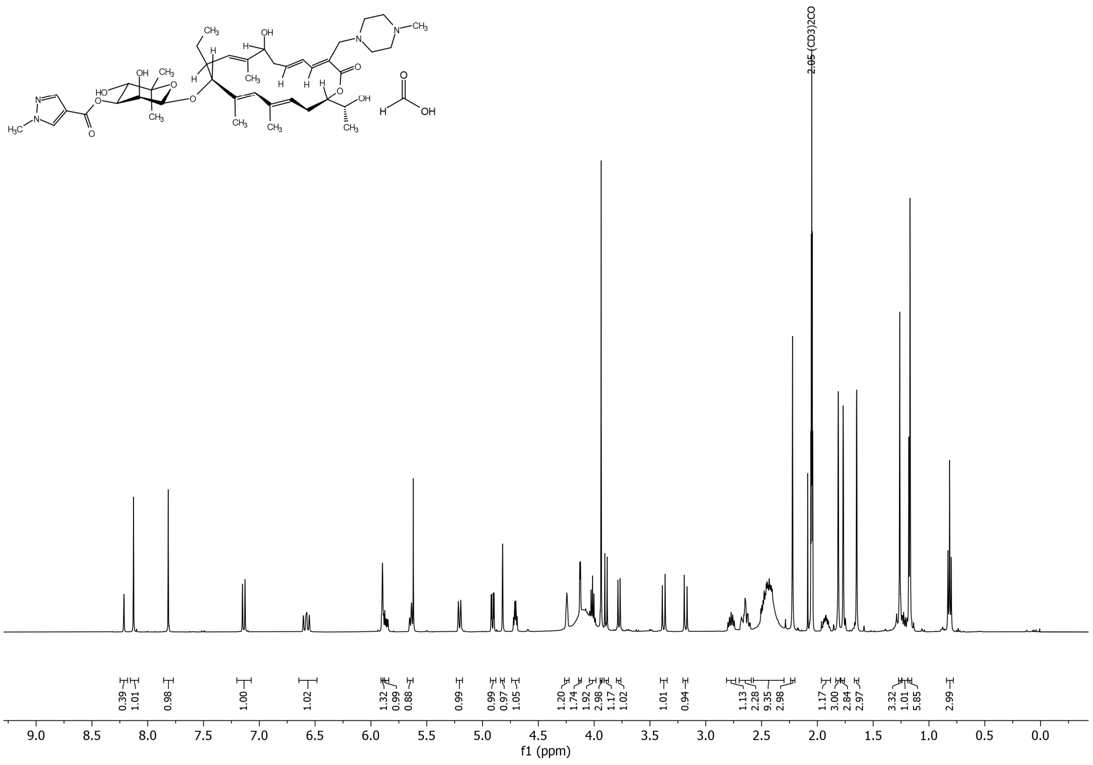

^{13}C NMR spectrum of **46** (126 MHz, acetone-d_6)

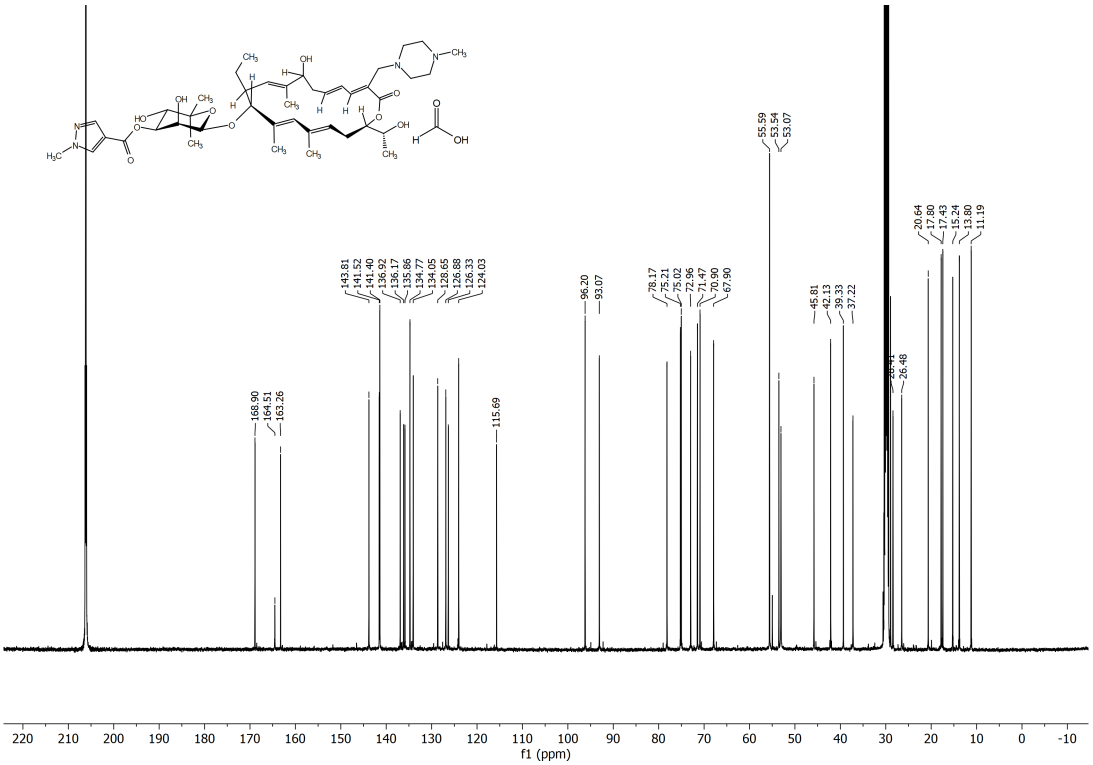

Supplement: Supplementary file 1 — Supporting Information [file ANIE-64-e202419095-s001.pdf]
